# Supplementary material for: Are perioperative interventions effective in preventing chronic pain after primary total knee replacement? A systematic review
Source: BMJ Open. 2019 Sep 6;9(9):e028093. doi: 10.1136/bmjopen-2018-028093 (PMC6731899; doi:10.1136/bmjopen-2018-028093)
Supplement: Supplementary data [file bmjopen-2018-028093supp001.pdf]

Supplementary material. Search strategy as applied in MEDLINE on Ovid

1 randomized controlled trial/ or randomized controlled trial.pt.

2 controlled clinical trial.pt.

3 randomized.ab.

4 placebo.ab.

5 randomly.ab

6 trial.ab

7 randomised.tw

8 1 or 2 or 3 or 4 or 5 or 6 or 7

9 review/

10 'systematic review$'.mp

11 9 or 10

12 8 or 11

13 Arthroplasty, Replacement, Knee/

14 Knee Prosthesis/

15 (arthoplast$ adj3 knee$).mp. [mp=title, abstract, original title, name of substance word, subject heading word, keyword heading word, protocol supplementary concept word, rare disease supplementary concept word, unique identifier]

16 (knee$ adj3 replac$).mp. [mp=title, abstract, original title, name of substance word, subject heading word, keyword heading word, protocol supplementary concept word, rare disease supplementary concept word, unique identifier]

17 (knee adj3 implant$).mp. [mp=title, abstract, original title, name of substance word, subject heading word, keyword heading word, protocol supplementary concept word, rare disease supplementary concept word, unique identifier]

18 13 or 14 or 15 or 16 or 17

19 12 and 18

Beswick AD, *et al. BMJ Open* 2019; 9:e028093. doi: 10.1136/bmjopen-2018-028093

Supplementary material. All peri-operative interventions with long-term pain or score follow up

1. Pain management

**Author**<br>**Country**<br>**Recruitment dates**<br>**Setting**	**Indication**<br>**Number randomised intervention; control**<br>**Age**<br>**% female**	**Common anaesthesia**			**Follow up**<br>**Losses to follow up intervention; control**<br>**Risk of bias issues**<br>**Key results**
		**Group 1 (intervention)**	**Group 2 (intervention)**	**Group C (control)**	
FNB single vs No FNB					
Widmer et al. 2012[34]<br>Australia<br>Before 2012<br>2 surgeons	Elective primary unilateral TKR<br>27; 28<br>Median 72.1 (IQR 64.4, 76.5); 69.4 (63.4, 75.5)<br>44.4%; 44.4%	Premedication 1-3mg i.v. midazolam. Propofol induction and sevoflurane general anaesthetic.<br>LIA with 200mg ropivacaine and 0.5mg adrenaline in 100ml saline.<br>PCA 20µg fentanyl at 5-minute intervals on demand until morning POD2. Then, oral oxycodone SR 10mg every 12 hours. Daily COX II inhibitor and paracetamol 1g every 6 hours as tolerated. For breakthrough pain, 5-10mg oxycodone immediate release every 3 hours as needed.			1 year<br>No losses to follow up reported<br>Low risk of bias<br>WOMAC pain (high score favourable) at 1 year: FNB and LIA median 2.0 (IQR 0, 2.8); LIA no FNB 1.0 (0, 2.0). p=0.74<br>No adverse events occurred in either group
		Ultrasound guided FNB with 100mg ropivacaine in 30ml saline		Sham setup for FNB. No identification or injection of femoral sheath	
FNB single vs ONB vs Control					
Bergeron et al. 2009[105]<br>Canada<br>2005-2006<br>1 centre	Elective primary unilateral TKR<br>19; 20; 20<br>Mean 65.1 (SE 2.0); 72 (1.8); 67 (1.3)<br>79%; 80%; 75%	Intraoperative sedation with iv propofol at discretion of anaesthesiologist. Lumbar spinal anaesthesia with 12mg 0.5% bupivacaine.<br>Postoperative i.v. PCA with fentanyl 50µg/ml set to deliver 25µg every 5 min as needed.<br>Celecoxib 100mg and acetaminophen 650mg on arrival in recovery room and every 12 and 6 hrs respectively. Breakthrough medication with intramuscular ketorolac 10 mg every 4 hrs.			1 year<br>Overall 32 lost to follow up<br>High risk of bias: only 27/59 patients followed up due to resource limitations.<br>No difference in HSS pain at rest or during activity at 1 year between the study groups.

Beswick AD, *et al. BMJ Open* 2019; 9:e028093. doi: 10.1136/bmjopen-2018-028093

		FNB with stimulator. 20ml 0.5% bupivacaine with 1/200,000 epinephrine.	ONB with stimulator. 20ml 0.5% bupivacaine with 1/200,000 epinephrine.	No injection but inguinal area prepared, and sham block performed behind drapes.	No long-term complications attributable to anaesthetic regimen
FNB continuous low dose vs FNB continuous high dose vs No FNB					
Shum et al. 2009[106]<br>Singapore<br>Before 2009<br>1 hospital	Unilateral TKR for osteoarthritis<br>20 (17 received treatment); 20 (18 received treatment); 20<br>Mean 66.7 (SD 8.4); 65.4 (8.4); 67.8 (5.5)<br>88%; 72%; 80%	Spinal anaesthesia induced with 2-3ml hyperbaric 0.5% bupivacaine. Intraoperative sedation with midazolam in increments of 0.5mg.<br>Intravenous PCA morphine (1mg/ml, on-demand bolus doses of 1 mg with 5 minute lockout, maximum dose 8 mg/hr)			2 years<br>16.4% of patients who received intervention lost to follow up<br>Unclear risk of bias due to differences in OKS and weight at baseline, and limited methodological details.<br>No separate pain outcome but mean OKS slightly more favourable in group with no FNB, 18.2 (SD 3.7) compared with combined FNB groups, 19.8 (5.4) but this was not significant.<br>No complications attributable to use of FNB
		Low dose continuous FNB at conclusion of TKR with ropivacaine 0.15% (10 ml/hr in the first 24 hours, followed by 5ml/hr in the next 24 hours)	High dose continuous FNB at conclusion of TKR with ropivacaine 0.2% (10 ml/hr in the first 24 hours, followed by 5 ml/hr in the next 24 hours)	No FNB	
SNB injection vs SNB continuous vs control					
Wegener et al. 2013[44]<br>The Netherlands<br>2008-2010<br>1 centre	Unilateral TKR<br>29; 30; 30 (90 randomised)<br>Median 65 (range 43-81); 66 (43-83); 62 (50-79)<br>62%; 70%; 73%	Lorazepam 1mg 2 hours and acetaminophen 2g 1 hour before surgery. FNB with stimulating catheter: loading dose 20 ml levobupivacaine 0.375% and after 45 minutes a continuous infusion of levobupivacaine 0.125% 10 ml/hr. General anaesthesia induced with 3-5 µg/ml propofol infusion and remifentanil 0.5 µg/kg/min and maintained with 2-3 µg/ml at 0.1-0.25 µg/kg/min. Postoperatively, FNB changed to patient controlled FNB, 5ml bolus, 30-minute lockout; basal rate 6 ml/hr. i.v. morphine administered if needed. Postoperative analgesia with acetaminophen 1g 4 times daily. Diclofenac 50mg or tramadol 50mg 3 times daily. Tramadol 100mg before removal of nerve catheters. Morphine pain relief as required.			12 months<br>2;7;5 lost to follow up<br>Low risk of bias<br>Median WOMAC pain scores at 12 months: SNB injection 80 (range 25-100), SNB continuous 90 (55-100) and PCA only 90 (35-100), p=0.81. No difference between groups in VAS pain at rest (p=0.90) or during mobilisation (p=0.43).<br>No information on adverse events.

		Group Fs: SNB single injection. SNB loading dose of 20 ml levobupivacaine 0.375%.	Group FCS: SNB continuous infusion. SNB loading dose of 20 ml levobupivacaine 0.375%. Continuous infusion of levobupivacaine 0.125% 10 ml/hr started 45 mins after catheter placement. SNB maintained for 36 hours postoperatively (10 ml/hr).	Group F: No SNB. PCA via femoral nerve catheter	
**General anaesthesia vs FNB single vs FNB/ SNB single**					
Gao et al. 2017[35]<br>China<br>2014-2015<br>1 centre	Primary unilateral TKR for osteoarthritis<br>50; 50; 50<br>Mean 65.8 (SD 6.7); 66.4 (7.4); 67.6 (6.3)<br>81%; 80%; 76%	Pre-operative and post-operative celecoxib 0.2g twice daily.<br>100ml intra-operative LIA with ropivacaine 200mg and epinephrine 0.25 mg.			6 months<br>2; 1; 0<br>Low risk of bias<br>Mean HSS at 6 months: 87.1 (SD 6.9); 87.4 (7.3); 88.5 (6.7). No significant difference.<br>Nausea and vomiting: 4; 2; 1, urinary retention: 3; 1; 2.
		General anaesthesia	Ultrasound guided FNB 5g/l ropivacaine 20ml plus 0.1mg epinephrine	Ultrasound guided FNB 5g/l ropivacaine 20 ml plus 0.1mg epinephrine and SNB 5g/l ropivacaine 20ml plus 0.1mg epinephrine	
LIA no corticosteroid vs No LIA/ placebo					
Wylde et al. 2015 [45]<br>UK	Primary unilateral TKR for osteoarthritis	FNB with nerve stimulator and/ or ultrasound guidance (20ml 0.25% bupivacaine). Spinal or general anaesthetic. Intra-operative analgesia provided by titration of i.v. fentanyl initially and morphine if necessary. 1g intravenous			6 and 12 months<br>24;19 at 12 months (including those who did not receive treatment)

Beswick AD, *et al. BMJ Open* 2019; 9:e028093. doi: 10.1136/bmjopen-2018-028093

2009-2012<br>1 centre	157; 159 (143; 137 received treatment)<br>Mean 69.5 (SD 9.4); 68.7 (7.9)<br>52%; 54%	paracetamol 30 minutes before the end of operation. Immediately post-operative 400mg oral ibuprofen.<br>PCA with morphine 1mg/ml, 1 mg bolus dose and a 5-minute lock-out. If necessary morphine bolus up to 0.2mg/kg as rescue analgesia. During hospital stay, visit from pain specialist nurse. Oral or i.v. paracetamol every 6 hours and ibuprofen 400mg every 8 hours. When PCA no longer needed, oral codeine phosphate 30-60mg every 6 hours, tramadol 50-100mg every 6 hours and oramorph 10-20mg as rescue analgesia.		Low risk of bias<br>At 12 months WOMAC pain score (0-100) in LIA group median 90 (IQR 30), Control 85 (35); ITT-CC linear regression coefficient 3.83 (95%CI -0.83, 8.49), p=0.107. At 6 months WOMAC pain score ITT-CC linear regression coefficient 4.10 (95%CI -0.22, 8.43), p=0.063. Mean differences lower than MCID of 8-9[77].<br>Superficial and deep wound infection rate in LIA group 3.2% and 1.9% in control group, p=0.500. No differences in serious adverse events between groups
		60ml intra-operative LIA with 0.25% bupivacaine and 1/200,000 adrenaline injected into the posterior capsule, medial and lateral capsule, fascia and muscle, and subcutaneous tissues.	No treatment other than standard care	
Williams et al. 2013[51]<br>Canada<br>Before 2013<br>1 centre, 2 surgeons	Primary unilateral TKR for osteoarthritis<br>35; 32 (26; 25 received treatment)<br>Mean 66 (SD 9.7); 67 (12.5)<br>58%; 60%	Sedation with i.v. midazolam and propofol. Intraoperative LIA loading dose of 20ml 0.25% bupivacaine/ epinephrine injection, 10ml into medial and lateral subcutaneous tissue around the incision and 10ml intra-articular after closure. Infiltrate delivered by pain pump into lateral recess of intra-articular space. Spinal anaesthetic with 10-15 mg of 0.75% or 0.5% plain bupivacaine and 20μg fentanyl.<br>Postoperative morphine PCA. 7.5mg i.v ketorolac preoperatively plus 15mg every 6 hours postoperatively for 48 hours, then oral ketorolac 10mg every 6 hours for 2 days. Gabapentin 600mg given preoperatively plus 300mg twice daily for 48 hours postoperatively. Oxycodone 10mg twice daily for 48 hours postoperative. Oral paracetamol 650mg every 4 hours for 72 hours.		6 and 12 months<br>3;1 of those who received treatment<br>Low risk of bias<br>Mean VAS pain score at 6 months 1.2 (SD 1.3); 1.2 (1.2). p=0.836. At 12 months 0.9 (1.2); 1.0 (1.1). p=0.767<br>No short-term differences in adverse events except control patients more likely to be drowsy at 48 hrs. Long-term adverse events not reported.
		Infusion of 0.5% bupivacaine at 2ml/hr for 48 hrs	Infusion of saline at 2ml/hr for 48 hrs	
Niemeläinen et al. 2014[47]<br>Finland<br>2011-2012	Primary unilateral TKR for osteoarthritis<br>30; 30 (27; 29 received treatment)	Oral paracetamol 1g given 1 hour before surgery. Spinal anaesthesia with 15mg bupivacaine in 3ml.<br>After surgery oral paracetamol 1g every 6 hours and oral meloxicam (15mg) every 24 hours.<br>PCA with oxycodone 2mg, lock-out time 8 min.		12 months<br>1; 4<br>Low risk of bias<br>No pain measure separate from OKS. Weak evidence of more favourable

<table>
<tr><td rowspan="2">1 hospital</td><td rowspan="2">Mean 65 (SD 4.9); 64 (6.7)
56%; 48%</td><td colspan="2">Rescue levobupivacaine medication through a lumbar epidural catheter</td><td rowspan="2">OKS (0-48) in the LIA group at 12 months, mean difference -2.7 (95% CI -5.48, 0.07). Difference lower than MCID of 4.0[78].
Infection: 0; 0. Severe pain treated with epidural analgesia: 0; 3. Nausea: 1; 1</td></tr>
<tr><td>Intra-operative periarticular LIA of 100ml saline with levobupivacaine (150mg) mixed with ketorolac (30mg) and adrenaline (0.5mg).</td><td>Intra-operative periarticular LIA of 100ml saline</td></tr>
<tr><td rowspan="2">Motififard et al. 2017[49]
Iran
2014-2015
1 hospital</td><td rowspan="2">Primary unilateral TKR for osteoarthritis
60; 60
Mean 66.4 (6.4); 64.5 (6.0)
86.0%; 94.3%</td><td colspan="2">Spinal anaesthesia.
No FNB or SNB.
Pain medication provided as required after surgery: meloxicam (15 mg daily), celecoxib (400 mg daily), acetaminophen (1g every 8 hours), tramadol (50 mg every 8 hours), ketorolac (30 mg slow IV every 8 hours, with a 4-dose max), and morphine (5–10 mg slow IV if needed)</td><td rowspan="2">6 months
3; 7
Low risk of bias
No separate pain measure. Weak evidence for improved KSS (0-200) in LIA group at 6 months, mean 115.55 (SD 15.506); 101.40 (16.117). P=0.07. Difference of 14.15 greater than MCID of 12.3[79]. Difference was significant at 6 weeks, p<0.001.
No complications related to TKR or LIA. Low back pain (1; 2), stroke (0; 1), CHF (1; 0)</td></tr>
<tr><td>Peri-articular injection, 15 minutes before incision, of 100ml saline containing 50 mg bupivacaine hydrochloride 0.5%, 1 ml morphine sulphate 10 mg/ml, 300 µg epinephrine (1:1000) and 30 mg ketorolac</td><td>100ml saline containing 300 µg epinephrine (1:1000)</td></tr>
<tr><td rowspan="2">McDonald et al. 2016[52]
UK
2010-2011
1 hospital</td><td rowspan="2">Primary unilateral TKR for osteoarthritis
113; 109 received common spinal anaesthesia (121; 121 randomised)
Median 68 (IQR 62, 72); 67 (62, 73)
59%; 55%</td><td colspan="2">Oral premedication with 10-20mg temazepam, 150mg ranatidine, 10mg dexamethasone, 300mg gabapentin, 1g paracetamol.
Spinal anaesthesia</td><td rowspan="2">12 months
9; 11 of those receiving treatments
Low risk of bias
No separate pain outcome. Mean OKS at 12 months: median 41 (IQR 35, 44); 41 (34;44). P=0.915
Suspected infection 2; 1. MI 0; 1. GI bleed 1; 0. renal failure 1; 0. Died 2; 0)</td></tr>
<tr><td>Intra-articular and subcutaneous infiltration during surgery of 200 ml of 2mg/ml ropivacaine without adrenalin or additives. Catheter inserted, and 20 ml infiltrate injected following wound closure. Further boluses of 40 ml 2 mg/ml ropivacaine via infusion pump 4 hours after leaving theatre and morning of POD1. Two</td><td>Epidural PCA with 4 ml of 2.5 mg/ml levobupivacaine introduced at end of surgery. Thereafter self-medication with 2 ml of 1.25 mg/ml bupivacaine with 15 minutes lockout until morning of POD1. Nurse-administered rescue of 4 ml of 2.5 mg/ml levobupivacaine.</td></tr>
</table>

		additional top ups of 40 ml 2mg/ml ropivacaine were prescribed if required.		
Celecoxib vs placebo				
Meunier et al. 2007[54]<br>Sweden<br>2004-2005<br>1 centre	Elective primary unilateral TKR for osteoarthritis<br>25; 25 (24; 20 received treatment)<br>Mean 68 (SD 6.3); 69 (7.7)<br>71%; 40%	Spinal anaesthesia with bupivacaine 17.5-20mg. i.v. midazolam or propofol sedation if needed. Paracetamol 1 g preoperatively and then with tramadol 50-100 mg 4 times a day during hospital stay. Ketobemidone (2.5-5mg i.v. or subcutaneous) on demand. Paracetamol and tramadol used as required after discharge.		12 months<br>No losses to follow up after surgery reported<br>Low risk of bias<br>No effect of celecoxib on VAS or KOOS pain at 1 year.<br>DVT: 0; 1. Deep infection: 0; 0.
		Oral celecoxib 200mg 1 hour preoperatively and twice daily for 3 weeks	Oral placebo 200mg 1 hour preoperatively and twice daily for 3 weeks	
Ketamine vs placebo				
Perrin and Purcell2009 [107]<br>Australia<br>Before 2009<br>1 centre (pilot study)	Elective unilateral TKR<br>16 (5; 7 completed study per protocol)<br>Mean 65.6 (SD 10.2); 60.3 (11.9)<br>40%; 43%	Intrathecal injection of 15mg bupivacaine and 100µg morphine. General anaesthesia. After surgery 1.5g paracetamol and then 750mg every 4 hours; PCA with morphine 2mg boluses with 10-minute lockout; morphine rescue 2.5mg intravenously as required; and rescue oral ibuprofen 800mg.		6 months<br>3 protocol breaches and 1 patient with uncontrolled pain.<br>High risk of bias due to non-ITT reporting and recruitment difficulties<br>2/5 ketamine group had mild/moderate pain on the WOMAC pain scale at 26 weeks or failed to improve compared with 5/7 controls.<br>1 adverse psycho-mimetic effect not attributed to intervention or control treatment
		Ketamine 0.5mg/kg bolus followed by 4µg/kg/min infusion. Commenced before surgical incision and continued until wound bandaged or syringe empty.	Saline infusion. Commenced before surgical incision and continued until wound bandaged or syringe empty.	
Ketamine vs Nefopam vs placebo				
Aveline et al. 2014[55]<br>France<br>2005<br>1 centre	Elective primary unilateral TKR for osteoarthritis<br>25; 25; 25<br>Mean 73 (SD 9); 72 (9); 70 (7)<br>67%; 60%; 63%	General anaesthesia induced with 1.5-2mg/kg propofol, 1µ/kg remifentanil and a single bolus of cisatracurium 0.15mg/kg. Remifentanil infusion at 0.15µg/k/min until skin closure. Anaesthesia maintained with sevoflurane 0.9-1.2% with 50% nitrogen in oxygen. 20 mins before skin closure, 0.15mg/kg i.v. morphine bolus and 0.625mg droperidol. PCA with morphine hydrochloride 1 mg i.v. bolus with 7-min lockout. On arrival in recovery room, 3 mg i.v. morphine boluses at 5 minute intervals.		6 and 12 months<br>3; 1; 2<br>Low risk of bias<br>Median DN4 at 12 months: 1 (IQR 1, 2); 1 (0, 1); 2 (1, 3). p=0.02 for difference between ketamine and placebo groups. Number of patients with VAS pain on movement score

		0.2mg/kg nefopam administered over 20 min before incision; 2mg/ml nefopam continuous infusion at 120µg/kg/hr until end of surgery and 60µg/kg/hr for 48 hours	0.2mg/kg ketamine administered over 20 min before incision; 2mg/ml ketamine continuous infusion at 120µg/kg/hr until end of surgery and 60µg/kg/hr for 48 hours	Saline administered over 20 minutes before incision; saline continuous infusion until second post-operative day	≥40mm at 12 months by group: nefopam (3/22, 13.7%), ketamine (3/24, 12.5%), and placebo group (6/23, 26.1%). Ketamine reduced DN4 pain (p=0.02) compared with placebo. At 12 months only 7/69 patients had DN4≥4 indicative of neuropathic pain.<br>Infection: 0; 0; 0. Revision: 0; 0; 0.
Pregabalin vs placebo					
Buvanendran et al. 2010[56]<br>USA<br>2006-2007<br>Single centre	Primary unilateral TKR for osteoarthritis.<br>120; 120 (9; 2 did not receive post-operative treatment but ITT analysis)<br>Mean 64.0 (SD 8.3); 63.3 (8.9)<br>76%; 70%	Sedation with midazolam and i.v. propofol. Combined spinal-epidural anaesthetic. 1.5ml 0.75% hyperbaric bupivacaine with 25µg fentanyl injected intrathecally. Catheter inserted for epidural drug administration.<br>LIA 60 ml 0.25% bupivacaine with epinephrine infiltrated into the wound at capsule closure. From completion of surgery until 32-42 hours post-operative, epidural infusion of fentanyl (5µg/ml) and bupivacaine (1mg/ml) initiated using continuous basal infusion of 6ml/hr with epidural PCA bolus doses (maximum 10ml/hr). Patients transitioned to oral opioid (morphine, oxycodone, and hydromorphone) as required. All patients received preoperative oral celecoxib 400mg 1–2 hours before surgery and 200mg twice daily for 3 days in hospital.			6 months<br>7; 5<br>Low risk of bias<br>Mean VRS pain score at 6 months: pregabalin 0.41 (SD 1.20); control 0.95 (1.80). p=0.0084. Distributions skewed but nonparametric Wilcoxon significant (p=0.0176). Difference of 0.54 less than MCID of 1.0.<br>In the pregabalin group the incidence of neuropathic pain measured using S-LANSS was 0% (0/113) and 5.2% (6/115) in the placebo group (p=0.014).<br>No clinically significant adverse events up to 6 months and no falls. Sedation, confusion and dry mouth more frequent in pregabalin than placebo group on day of surgery and first postoperative day.
		Oral pregabalin 300mg 1–2 h before surgery, 150mg twice daily for the first 10 postoperative days, 75mg twice daily on days 11 and 12, and 50mg twice daily on days 13 and 14	Oral placebo 1–2 h before surgery, twice daily for the first 10 postoperative days, twice daily on days 11 and 12, and twice daily on days 13 and 14		
FNB long duration vs FNB short duration					

<table>
<tr><td rowspan="2">Ilfeld et al. 2009[108]
USA
2005-2007
2 centres</td><td rowspan="2">Primary unilateral TKR for osteoarthritis
25; 25
Median 66 (IQR 60, 70); 64 (60, 69)
56%; 60%</td><td colspan="2">Femoral catheter inserted using nerve stimulator. FNB with 0.2% ropivacaine infusion (8ml/hr basal; 4 ml patient-controlled bolus; 30-minute lockout) from surgery until a.m. POD1.
1 week oral acetaminophen (975mg every 6 hours), sustained release oral opioid (Oxycontin, 10mg every 12 hours), and either oral aspirin (650mg daily) or celecoxib (200mg every 12 hours). Oral oxycodone 5 mg tablets and/ or i.v. morphine sulfate 2-4 mg for breakthrough pain.</td><td rowspan="2">6 and 12 months
4; 1 lost to follow up
High risk of bias: uneven loss to follow up between groups; muscle weakness resulted in lower dose of infusion on POD1 (10 continuous; 3 saline)
Groups had similar WOMAC pain scores at 6 and 12 months (p>0.05).
MI: 1; 0. PE: 1; 0. Fall: 1; 0. Catheter leak, dislodged: 1; 2</td></tr>
<tr><td>At 6 a.m. POD1, infusion pump replaced with infusion pump with 0.2% ropivacaine.
At 6 p.m. POD2 pump replaced with portable infusion pump (400ml 0.2% ropivacaine). Catheter removed evening of POD4.</td><td>At 6 a.m. POD1, infusion pump replaced but saline substituted.
At 6 p.m. POD2 pump replaced with portable infusion pump (saline). Catheter removed evening of POD4</td></tr>
<tr><td rowspan="2">Ilfeld et al. 2011[109]
USA
2007-2009
2 centres</td><td rowspan="2">Primary unilateral cemented TKR for osteoarthritis
40; 40 (39; 38 included in RCT)
Median 61 (IQR 58, 67); 66 (60, 70)
67%; 66%</td><td colspan="2">Femoral catheter inserted using nerve stimulator. FNB with 0.2% ropivacaine infusion (6ml/hr basal; 4ml patient-controlled bolus; 30-min lockout) from surgery until POD1.
1 week oral acetaminophen (975mg every 6 hours), sustained release oral opioid (Oxycontin, 10mg every 12 hours), and either oral aspirin (650mg daily) or celecoxib (200mg every 12 hours). Oral (oxycodone 5mg or 10mg tablets) and/ or i.v. opioids (morphine sulfate 2-4mg) for breakthrough pain.</td><td rowspan="2">12 months
11; 12 incomplete follow up
High risk of bias: 11;12 did not have 4 measures out of 6 up to 12 months; graph suggests WOMAC pain lower pre-intervention in continuous infusion group.
No difference in WOMAC pain scores between randomised groups (p>0.05).
Falls: 4; 0</td></tr>
<tr><td>At 6 a.m. POD2, infusion pump replaced and 0.2% ropivacaine continued.
At 6 p.m. POD2 pump replaced with portable infusion pump (400ml 0.2% ropivacaine). Catheter removed evening of POD4</td><td>At 6 a.m. POD2, infusion pump replaced but saline substituted.
At 6 p.m. POD2 pump replaced with portable infusion pump (saline). Catheter removed evening of POD4</td></tr>
<tr><td>Choy et al. 2011[42]
Korea
2006-2007</td><td>Primary unilateral TKR for osteoarthritis</td><td colspan="2">Spinal anaesthesia. Continuous FNB via catheter until POD3. Catheter inserted with use of nerve stimulator. Analgesia induced with 20ml of 1:1 0.25% bupivacaine and 2% lidocaine with 1:200,000 epinephrine. Continuous</td><td>2 years
4; 3 lost to follow up</td></tr>
</table>

<table>
<tr><td rowspan="2">1 surgeon</td><td rowspan="2">33; 30 (2 patients received GA and excluded)
Mean 66.7 (SD 10); 67.5 (11)
97%; 93%</td><td colspan="3">infusion with 0.125% bupivacaine 5.0ml/hr. i.v. PCA (butorphanol 4mg, ketorolac 150mg, saline 50ml), programmed to deliver 1 mg bolus (lockout 10 min) with maximum dose 6mg/hr. i.v. paracetamol 2g 4 times/ day and oral ibuprofen 600mg 3 times/ day for breakthrough pain</td><td rowspan="2">Low risk of bias for 2 year outcome measures.
At 2 years, intervention WOMAC pain mean 7.2 (SD 2), control 6.3 (SD 1); p=0.2
Superficial infection: 1; 1</td></tr>
<tr><td colspan="2">Continuous femoral nerve block via catheter continued from POD3 to POD7</td><td>Continuous femoral nerve block discontinued on POD3</td></tr>
<tr><td colspan="6">FNB continuous high concentration vs FNB low concentration vs FNB single</td></tr>
<tr><td rowspan="2">Albrecht et al. 2014[41]
Canada
2009-2011
1 hospital</td><td rowspan="2">Scheduled primary unilateral TKR
32; 32; 35
Mean 61 (CI 57, 64); 63 (60, 67);63 (60, 66)
46%; 44%; 52%</td><td colspan="3">Stimulating catheter inserted with ultrasound guidance. Immediately after catheter placement, 10ml mepivacaine 2% was injected through the catheter. SNB using 30 ml ropivacaine 0.2%. Spinal anaesthesia with 2.5 to 3.0 ml isobaric bupivacaine 0.5% and 0.1mg intrathecal morphine.</td><td rowspan="2">12 months
4;0;2 lost to follow up
Low risk of bias.
No separate pain outcome. Mean WOMAC score at 12 months: high concentration FNB 17 (95% CI 7, 27); 22 (14, 30); 18 (8, 27). P=0.68
Falls: 0; 0; 1</td></tr>
<tr><td>Bolus of 20ml ropivacaine 0.2% with epinephrine 1:400,000 into the femoral catheter followed by ropivacaine 0.2% at a rate of 5 ml/hr with patient-controlled boluses of 5ml available every 30minutes.</td><td>Bolus of 20 ml ropivacaine 0.2% with epinephrine 1:400,000 into femoral catheter followed by ropivacaine 0.1% at rate of 10ml/hr with patient-controlled boluses of 10 ml available every 30 minutes.</td><td>Bolus of 30ml ropivacaine 0.375% with epinephrine 1:400,000 into the femoral catheter followed by normal saline at a rate of 1 ml/hr with patient-controlled boluses of 1mL available every 30minutes.</td></tr>
<tr><td colspan="6">FNB continuous vs Psoas compartment block vs FNB continuous and psoas compartment block</td></tr>
<tr><td>Morin et al. 2005[110]
Germany
Before 2005
1 centre</td><td>Elective unilateral TKR
30; 30; 30</td><td colspan="3">Oral pre-medication with 20mg chlorazepate. General anaesthesia with intravenous propofol and 4–8µg/kg i.v. fentanyl and desflurane in N2O. 100mg diclofenac suppository after anaesthesia induction and 2.5g intravenous metamizole before end of surgery. Post-operative 3 daily doses of oral diclofenac 50mg. i.v. PCA</td><td>9–12 months
7; 6; 5
High risk of bias due to large losses to follow up, non-blinded outcome collection, and differences between</td></tr>
</table>

<table>
<tr><td rowspan="2"></td><td rowspan="2">Median 68 (IQR 62, 74); 71 (63, 74); 65 (53, 73)
50%; 70%; 59%</td><td colspan="3">with piritramide bolus 2mg as needed with lockout interval of 10 mins for 48 hours.</td><td rowspan="2">groups in BMI and anaesthetist’s opinion of difficulty of catheter placement.
No difference between groups in level of pain at the knee joint during past 4 weeks: FNB median 2.5 (IQR 1, 4), FNB and SNB 2 (1, 4), Psoas block 2 (IQR 1, 4), p=0.44
No early complications but longer term adverse events not reported.</td></tr>
<tr><td>Continuous FNB
Stimulating catheter used. Initial bolus of prilocaine 1% and ropivacaine 0.75%. 300mg prilocaine 1% (30ml) and 150mg ropivacaine 0.75% (20ml). During first 48hrs post-operative ropivacaine 0.2% infusion 14ml/hr.</td><td>Continuous FNB and continuous SNB
Stimulating catheter used. Initial bolus of prilocaine 1% and ropivacaine 0.75%. In each catheter: 200mg prilocaine 1% (20ml) and 75mg ropivacaine 0.75% (10ml). During first 48hrs post-operative infusion through each catheter of 0.2% ropivacaine 7ml/hr.</td><td>Continuous psoas compartment block
Stimulating catheter used. Initial bolus of prilocaine 1% and ropivacaine 0.75%. 300mg prilocaine 1% (30ml) and 150mg ropivacaine 0.75% (20ml). During first 48hrs post-operative ropivacaine 0.2% infusion 14ml/hr.</td></tr>
<tr><td colspan="6">ACB continuous vs FNB continuous</td></tr>
<tr><td>Davidson et al. 2016[111]
USA
2013-2014
2 studies combined from 1 centre</td><td>Primary, unilateral TKR or unicompartmental
54 (39 TKR, 16 UKR); 56 (41 TKR, 15 UKR)
TKR mean 67 (SD 8); 66 (7). UKR 70 (10); 68 (12)</td><td colspan="3">Spinal or general anaesthesia. Intra-operative i.v. fentanyl, hydromorphone, and/or morphine.
LIA with 30 ml ropivacaine (0.5%), ketorolac (30 mg), and epinephrine (5 µg/ml).
Post-operative: oral acetaminophen (975 mg every 6 hr), celecoxib (200 mg every 12 hr), and sustained release oxycodone (10 mg every 12 hr). For breakthrough pain, infusion pump bolus (4 ml, 30-min lock-out). Rescue opioid titrated to pain severity. 10 ml lidocaine (2%) bolus was given via the perineural catheter for moderate or severe pain.</td><td>12 months
31; 29
High risk of bias due to partial follow up
TKR and UKR combined. Pain score (0-10) at 12 months median 0.0 (IQR 0.0, 3.0); 0.5 (0.0, 2.0). P=0.80). Pain score >0: 35%; 32%. P=0.65. No difference at 4 months when follow up more complete (51;</td></tr>
</table>

	TKR 59%; 66%.<br>UKR 47%; 47%	Ultrasound guided ACB. Ropivacaine 0.2% at basal rate of 6 ml/hr, a 4-ml bolus, and a lockout of 30 minutes	Ultrasound guided continuous FNB. Ropivacaine 0.2% at basal rate of 6 ml/hr, a 4-ml bolus, and a lockout of 30 minutes	52) in pain score (p=0.80) or pain score >0 (p=0.48).<br>Falls in hospital: 2; 5
ACB single vs FNB single				
Macrinici et al. 2017[43]<br>USA<br>Before 2017<br>1 centre	Primary unilateral TKR, indication not specified (selected by the surgeon for TKA)<br>49; 49<br>Mean 67 (SD 8); 67 (8)<br>61%; 63%	Multimodal regimen including NSAIDs, non-opioid analgesics, opioids. LIA 40ml Marcaine 0.25%.<br>All patients received an ultrasound guided needle insertion into ACB and FNB sites.		6 months<br>3; 4 lost to follow up. 6; 3 complications<br>Low risk of bias<br>VAS pain similar between groups at 6 months. No difference in functional outcomes<br>Medical complications: 3; 0. Surgical complication: 0; 1. Temporary foot drop: 3; 2.
		Immediately after surgery, 30ml solution with 100ml Marcaine into ACB site. 30 ml saline into FNB site	Immediately after surgery, 30ml solution with 100ml Marcaine into FNB site. 30 ml saline into ACB site	
FNB continuous vs oral opioid				
Nader et al. 2012[36]<br>USA<br>2007-2008<br>1 surgeon	Elective unilateral TKR<br>31; 31<br>Median (IQR) 65 (60, 76); 64 (60, 71)<br>58%; 77%	Before surgery, patients received 1–2mg midazolam as needed. Epidural with 10mg 0.5% isobaric bupivacaine injected intrathecally. Intraoperative sedation with propofol infusion of 25-75mcg/kg/minute. In post-anesthesia recovery area, PCA epidural with basal infusion of 3 ml/hr (1 mg/ml bupivacaine and 10 mg/ml hydromorphone) with patient-activated boluses of 3 ml with a lockout interval of 15 minutes and per hour maximum of 15 ml. Infusion discontinued and epidural catheter removed on morning of POD 1. All subjects received 5 mg warfarin on evening of surgery and 40 mg enoxaparin starting on POD 1		6 and 12 months<br>1; 1 lost to follow up at 12 months<br>Low risk of bias<br>No difference in overall median NRS pain score at 6 months and 12 months: 0 (IQR 0, 1); 0 (0, 1). p=1.0. At 12 months, some evidence favouring hydrocodone for pain ascending/ descending stairs: 1 (0, 2); 0 (0, 0). p=0.01. Also, suggestion of reduced pain in hydrocodone group at night in bed (p=0.06) and sitting/ lying (p=0.07), standing upright (p=0.10). No difference walking on flat surface (p=0.41).<br>Falls in month after surgery: 1;0. Positive joint aspirate: 3; 0. VTE: 0; 4.
		Continuous FNB inserted with use of stimulator. After discontinuation of epidural anaesthesia on the morning of POD1 10mL bolus of ropivacaine 0.25% followed by 5ml/h infusion of 0.1% ropivacaine. On morning of POD 2, ropivacaine infusion	10 mg oral hydrocodone plus 325mg acetaminophen every 4-6 hours administered for pain as needed. Sustained release oxycodone 10mg for 12 hours with oral hydromorphone 2 mg over	

		discontinued. Femoral catheter removed 24 hours after previous dose of enoxaparin.	4 hours for breakthrough pain	
FNB continuous vs PCA				
Wang et al. 2015[112]<br>China<br>2012-2013<br>3 centres	Elective unilateral TKR<br>82; 86<br>No significant differences in age or sex	General anaesthesia with midazolam (0.02-0.04mg/kg), fentanyl (1µg/kg), propofol (1-2mg/kg) and cisatracurium (0.15mg/kg). Anaesthesia maintained with sevoflurane during surgery. Intramuscular injection with 10mg metoclopramide and 2.5mg droperidol 30 minutes before surgery. Post-surgery, celocoxib and parecoxib 40mg for patients with severe pain, and i.v. morphine if needed.		6 and 12 months<br>2; 4 lost to follow up at 12 months<br>Unclear risk of bias: limited reporting of randomisation methods.<br>No differences were observed between groups at 6 or 12 months for any HSS domain including pain.<br>No nerve injuries
		Continuous FNB with ultrasound stimulator. After surgery, 0.2% ropivacaine (20ml) injected through catheter. Then an analgesia pump was attached delivering 0.2% ropivacaine 8ml/hr.	Epidural PCA 0.2% ropivacaine was injected at a rate of 5 ml/hr in a 2ml pulse dose	
Peng et al. 2014[38]<br>China<br>Before 2014<br>1 centre (2 surgical teams with 4 surgeons and 2 anaesthesiologists)	Primary unilateral TKR<br>140;140<br>Mean: 66.8 (SD 9.4); 68.0 (SD 11.2)<br>73%; 65%	General intravenous and inhalational anaesthesia: midazolam 0.1-0.15mg/kg (etomidate 0.15-0.2mg/kg for patients >65 years), propofol 2.0-2.5mg/kg, sufentanil citrate 0.3-1.0µg/kg, and vecuronium 0.08-0.12mg/kg for induction of anaesthesia. Maintenance with inhalation of 1%-3% sevoflurane and continuous intravenous infusion of remifentanil 7-8µg/kg/hr and propofol 25-75µg/kg/min. After wound closure, 5-10µg intravenous sufentanil and loading dose of PCA injected. i.v. injection of 4mg ondansetron.		6 and 12 months<br>31; 38 at 12 months<br>Low risk of bias<br>Chronic post-operative pain (NRS 1+) in 38.5% of PCA group at 6 months compared with 25.7% in FNB group (p=0.021). No difference at 12 months (p=0.273).<br>Authors only reported short term adverse events associated with use of PCA.
		FNB with ultrasound guidance. Initial dose of 10ml 2% lidocaine and 10ml 1% ropivacaine. 30 minutes before end of operation, catheter connected to PCA pump; patients received loading dose of 5ml of 0.15% ropivacaine followed by infusion of 0.15% ropivacaine at 5ml/hr, with bolus of 5mL	i.v. PCA with tramadol 800mg, flurbiprofen axetil 100mg, and dexamethasone 5mg with saline to a volume of 80ml. Loading dose of 2ml followed by an infusion rate of 1 ml/hr with bolus of 2 ml. Lock time 15min.	

		and lock time of 30 min. Preoperatively, a loading dose of 30ml was injected for intraoperative analgesia.		
Wu and Wong 2014[37]<br>China<br>2009-2011<br>1 centre	Unilateral elective TKR, 98% for osteoarthritis<br>40; 39 (30; 30 after post randomisation exclusions)<br>Mean 68.8 (SD 6.4); 68.9 (7.5)<br>73%; 73%	Paracetamol, sustained release diclofenate, opioids (codeine or morphine). Spinal anaesthesia<br>Catheter inserted under nerve stimulation and ultrasound guidance. Standardised bolus of 15 mL 0.5% levobupivacaine. Continuous infusion of 8 to 12 mL/h 0.08% levobupivacaine postoperatively until POD 3	Intravenous PCA morphine after the operation	6 months<br>2; 2 not pre- and peri-operative exclusions<br>Low risk of bias<br>No separate pain outcome but improvement of KSS from pre-operative was FNB 48.73 and PCA 44.7 (p=0.513)<br>Including patients not followed up. Deaths: 0; 0. Infection: 1;1. DVT: 2; 3. Shock: 3;2. Transfusion: 2;3. Also from excluded cases. Atrial fibrillation and confusion: 0; 1. PE: 0; 1. Sepsis: 1;0. ICU admission for shock: 1; 0.
FNB and SNB continuous vs epidural PCA				
Anastase et al. 2014[113]<br>Germany<br>2010-2011<br>1 centre	Primary unilateral TKR<br>55; 50<br>Mean 68.2 (SD 9.2); 69.7 (SD 8.7)<br>65%; 69%	Premedication with 10 mg oral clorazepate. Spinal anaesthesia with light sedation: 12.5mg 0.5% bupivacaine. Supplemental postoperative analgesia available with i.v. piritramid<br>After spinal anaesthesia installed, SNB and FNB catheters inserted with ultrasound guidance. 5 ml bolus 0.2% ropivacaine. FNB with an hourly rate of 5 ml, bolus administration of 5 ml by the patient and the lock-out interval of 20 mins. SNB 5 ml/h to a maximum of 8 ml/h, 5 ml bolus administered by the patient	Epidural catheter installed at the same time as spinal catheter. 5ml 0.2% ropivacaine and PCA performed through the epidural catheter. Hourly rate 3 ml, bolus administration of 5 ml, and lock-out period of 30 minutes.	6 and 12 months<br>15; 14<br>High risk of bias due to large loss to follow up<br>Pain during previous 4 weeks: 1 no pain, 2 very little, 3 little, 4 moderate, 5 loud, 6 very loud (translation from German). No difference at 6 months p=0.37. At 12 months, FNB/SNB median 2.00 (1.00, 2.00), PCA 2.00 (2.00, 2.00) p=0.004 favouring FNB/SNB.<br>No falls associated with quadriceps weakness. 6 and 12 month adverse events not reported.

		and lock-out interval of 20 minutes.		
**FNB single vs LIA**				
Fan et al. 2016[39]<br>China<br>2012-2014<br>Single hospital, 2 surgeons	Primary unilateral TKR (75% osteoarthritis; 25% rheumatoid arthritis)<br>80; 80 (78; 79 in analysis)<br>Mean 68.4 (SD 8.8); 67.6 (6.3)<br>79%; 86%	General anaesthesia in all but 1 in each group. After surgery, i.v. morphine, PCA and parecoxib 40mg<br>FNB performed pre-operatively with 20ml ropivacaine 0.5%.<br>After cementing prostheses, 50ml of saline injected into periarticular soft tissue.	Placebo equivalent of FNB with saline<br>After cementing prostheses, 50ml of LIA mixture containing morphine (1ml: 10mg), ropivacaine (10ml: 100mg), and diprospan (1ml: 5mg betamethasone dipropionate and 2mg betamethasone sodium phosphate) injected into periarticular soft tissue.	1 year<br>3 protocol violations<br>Low risk of bias<br>No separate pain outcome. Mean KSS at 1 year similar between groups: 94.2 (SD 2.6); LIA 93.9 (3.1). p=0.51<br>Infection: 0; 0. DVT: 1; 1. Femoral nerve injury: 1; 0.
**FNB single and epidural vs LIA**				
Reinhardt et al. 2014[40]<br>USA<br>2010-2012<br>Single hospital, 2 surgeons	Elective unilateral TKR for osteoarthritis<br>51; 51 (49; 45 received allocated intervention)<br>Mean 67.9 (SD 10.9); 66.6 (10.1)<br>59.2%; 57.8%	Spinal anaesthetic (2.5ml 0.5% bupivacaine). Mobic 15mg daily. Oral Perocet or Vicodin as required. Subcutaneous Dilaudid for severe breakthrough pain. Intravenous Toradol.<br>Combined spinal-epidural (500ml hydromorphone 10µg/ml and bupivacaine HCl 0.06%).<br>Single intra-operative FNB injection (30ml 0.25% bupivacaine).<br>Continuous 48-hour epidural infusion (4ml/hr with 4ml per demand dose, locked out every 10 minutes with an hourly limit of 20ml). Epidural infusion weaned to 2ml/hr on POD1 and to 0 ml/hr at 5 p.m. on POD1. Demand dose with	Intra-articular knee catheter placed intraoperatively with continuous 0.2% ropivacaine infusion at 7 ml/hr until POD2.<br>Placebo epidural catheter, no FNB, and postoperative placebo continuous epidural infusion of saline.	1 year<br>0: 0 of patients who received allocated intervention<br>Low risk of bias<br>VAS pain at 1 year similar between groups (noted in text and shown graphically)<br>No wound-related complications or infections. 1 DVT and 1 DVT plus PE in epidural group. Arthrofibrosis: 2; 1

		lockout parameters continued for 48 hours.<br>Placebo intraarticular knee catheter placed intra-operatively with continuous saline 7ml/hr infusion until POD2.		
LIA with corticosteroid vs LIA with no corticosteroid				
Seah et al. 2011[53]<br>Singapore<br>2004-2005<br>1 hospital	TKR<br>50; 50<br>Mean 65.4; 67.9<br>Sex not stated	General or spinal anaesthesia. Postoperative oral naproxen and PCA (with morphine bolus of 1mg, lock-out time 5 minutes, and maximum dose 8 mg/hr) for 48 hours.		6 months and 2 years<br>No losses to follow up reported<br>Low risk of bias<br>No separate pain outcome but no statistically significant difference in OKS between groups at 2 years<br>Deep infection: 1; 1
		Intraoperative periarticular injection of 0.5ml/kg 1:200,000 epinephrine and 0.5% bupivacaine diluted with 30ml of normal saline. 40mg of corticosteroid (triamcinolone acetonide) was added to half the mixture. The solution with the corticosteroid was injected into the deep tissues. The remaining solution was injected into the skin incision before closure.	Intraoperative periarticular injection of 0.5ml/kg 1:200,000 epinephrine and 0.5% bupivacaine diluted with 30ml of normal saline. Half the mixture was injected into the deep tissues. The remaining solution was injected into the skin incision before closure.	
Yue et al. 2013[114]<br>China<br>2011-2012<br>1 hospital	Unilateral TKR for osteoarthritis<br>36; 36<br>Mean 70.2 (SD 6.4); 69.3 (5.7)<br>89%; 89%	General anaesthesia. PCA (25 mg/100ml morphine: a 1mg bolus, 6 minutes lock-out, and 5mg/hr maximum) for 72 hours after surgery. 5-10mg intramuscular morphine as rescue. Celecoxib pre- and post-operatively		6 and 12 months<br>No loss to follow up reported<br>Unclear risk of bias.<br>No separate pain outcome. No difference in mean KSS between groups at 6 or 12 months<br>No incision infection or tendon rupture complications
		Injections with local anaesthetic agent and adrenaline (0.75% ropivacaine 30ml, 1:1000 adrenaline 0.5ml, and isotonic sodium chloride solution 70ml) plus corticosteroid (1ml betamethasone).	Injections with local anaesthetic agent and adrenaline (0.75% ropivacaine 30ml, 1:1000 adrenaline 0.5ml, and isotonic sodium chloride solution 70ml) with no added corticosteroid.	

		Another 50ml syringes fluid without corticosteroid was infiltrated into the skin	Another 50ml syringes fluid without corticosteroid was infiltrated into the skin		
LIA including ketorolac vs epidural					
Spreng et al. 2012[115], Spreng et al. 2010[116]<br>Norway<br>2007–2009<br>1 hospital	Unilateral, non-cemented TKR with no patella resurfacing<br>34; 34; 34<br>66.5 (SD 11.); 67.2 (SD 8.9); 65.8 (SD 10.1)<br>61%;61%;67%	Premedication with oral paracetamol (1-2g). Spinal anaesthesia with 13-15mg bupivacaine 5mg/ml with 20µg fentanyl. If indicated, up to 10ml/hr 10mg/ml propofol for sedation. Acetaminophen 1g every 6 hours. i.v. PCA morphine for 48 hours after surgery (2mg bolus with 10 minutes lockout time). When PCA stopped, 10mg slow release oxycodone twice daily. 5mg oxycodone as rescue analgesia.			12 months<br>13 did not provide complete data<br>Unclear risk of bias due to limited reporting (long-term outcome only reported as conference abstract).<br>Perioperative analgesic treatment did not have any significant influence on any KOOS outcomes.<br>Infection: 0; 0; 1. No long-term adverse events reported
		i.v. injection of ketorolac 1ml (30mg/ml) and morphine 5ml (1mg/ml).<br>Infiltration with ropivacaine 150mg, epinephrine 0.5mg, ketorolac 30mg and morphine 5mg in 150ml saline.<br>After closure, catheter placed into knee joint and 10ml infiltrate injected. 22-24 hours after surgery, 20ml injection through catheter of ropivacaine 19ml (7.5mg/ml) and ketorolac 1ml (30mg/ml). i.v. injection of	i.v. injection of 6ml saline.<br>Infiltration with ropivacaine 150mg, epinephrine 0.5mg, ketorolac 30mg and morphine 5mg in 150ml saline.<br>After closure, catheter placed into knee joint and 10 ml infiltrate injected. 22-24 hours after surgery, 20ml injection through catheter of ropivacaine 19ml (7.5mg/ml) and saline 1ml. i.v. injection of saline 1ml.<br>Sham epidural catheter.	Epidural catheter inserted immediately before spinal anaesthesia. When spinal anaesthesia started to wear off, epidural infusion for 48 hrs with 6-10 ml/hr fentanyl 2µg/ml, epinephrine 1µg/ml, bupivacaine 1mg/ml.<br>No knee infiltrations.<br>Sham knee catheter with no injections	

Beswick AD, *et al. BMJ Open* 2019; 9:e028093. doi: 10.1136/bmjopen-2018-028093

		ketorolac 1ml (30mg/ml).<br>Sham epidural catheter.			
**Spinal with added high dose morphine sulphate vs spinal with added low dose morphine sulphate vs spinal with no morphine sulphate**					
Foadi et al. 2017[117]<br>Germany<br>Before 2017<br>1 centre	Unilateral TKR or THR for osteoarthritis<br>16; 16; 17<br>Mean 67.63 (SE 2.45); 67.33 (2.87); 63.71 (3.14) 56%; 44%; 65%	3ml spinal anaesthesia with 0.5% bupivacaine<br>Post-operative 1 g metamizole (orally or intravenously) every 4 hours. 5 mg morphine (intravenous or subcutaneous) as rescue<br>medication			6 months<br>"only a few dropouts". >70% questionnaire return rate.<br>Unclear risk of bias due to limited reporting of pilot RCT.<br>No difference in WOMAC pain between groups at 6 months.<br>No adverse events noted
		0.2mg morphine sulphate added to spinal anaesthesia	0.1mg morphine sulphate added to spinal anaesthesia	No morphine sulphate added to spinal anaesthesia	

2. Myofascial trigger point dry needling

**Author**<br>**Country**<br>**Recruitment dates**<br>**Setting**	**Indication**<br>**Number randomised intervention; control**<br>**Age**<br>**% female**	**Common pain management**		**Follow up**<br>**Losses to follow up intervention; control**<br>**Risk of bias issues**<br>**Key results**
		**Group 1 (intervention)**	**Group C (control)**	
Mayoral et al. 2013[118]<br>Spain<br>2007-2008<br>Single centre	Unilateral TKR for osteoarthritis<br>20; 20<br>Mean 71.7 (SD 6.1); 72.9 (7.9)<br>72.5%	General or spinal anaesthesia		6 months<br>4; 5<br>High risk of bias due to large losses to follow up<br>WOMAC pain at 6 months: mean 3.24 (SD 3.03); 3.13 (2.72). Difference not statistically significant. No difference between groups in VAS pain (p=0.725) or proportion of patients reporting significant VAS pain at 6 months.
		After anaesthesia and surgery started, dry needling applied 20 times to all myofascial trigger points by a trained and experienced physical therapist.	If spinal anaesthesia used, dry needling simulated behind screen	

Beswick AD, *et al. BMJ Open* 2019; 9:e028093. doi: 10.1136/bmjopen-2018-028093

				No complications related to the dry needling intervention. Other adverse events not collected.

3. Tourniquet

Author<br>Country<br>Recruitment dates<br>Setting	Indication<br>Number randomised intervention; control<br>Age<br>% female	Common blood conservation strategies		Follow up<br>Losses to follow up intervention; control<br>Risk of bias issues<br>Key results
		Group 1 (intervention)	Group C (control)	
Ejaz et al. 2014[58]<br>Denmark<br>2011-2012<br>1 centre	Primary unilateral TKR for osteoarthritis<br>35; 35 (33; 31 received intervention)<br>Mean 68 (SD 8.0); 68 (7.8) 45.5%; 45.2%	Before surgery, oral tranexamic acid (1g). Tranexamic acid (0.5g) 3 hours after surgery and 6 and 12 hours postoperatively.		6 and 12 months<br>0; 0 of those who received intervention<br>Low risk of bias<br>Statistically significant difference in KOOS pain intensity at 2 months favouring TKR without a tourniquet (p < 0.001). Small difference between groups not statistically significant at 6 and 12 months.<br>Small number of adverse events did not suggest extra risk in the group with no tourniquet.
		Appropriately sized thigh tourniquet applied. Limb exsanguination by elevation for 2 minutes and cuff inflated to 250mm Hg.	Appropriately sized thigh tourniquet applied but not inflated. Served as safety device in case of uncontrollable bleeding.	
Liu et al. 2014[59]<br>Australia<br>Before 2014<br>1 surgeon	Unilateral TKR for osteoarthritis<br>10; 10<br>Mean 67.0; 70.0<br>30%; 10%	PCA. No CPM		6 and 12 months<br>0; 0<br>Low risk of bias<br>No separate pain measure. Total OKS not significantly different at 6 and 12 months<br>Blood transfusions: 3; 0.
		Tourniquet inflated to 300 mmHg before skin incision. Tourniquet deflated after wound closure and dressing.	Tourniquet placed but not inflated	
Mittal et al. 2012[61]<br>Australia<br>2008-2010	Primary unilateral TKR<br>31; 34	Autologous blood re-infused if required		1 year<br>5; 2
		Short duration. Tourniquet set at 300mm Hg inflated	Long-duration. Tourniquet set at 300mm Hg inflated before	

Beswick AD, *et al. BMJ Open* 2019; 9:e028093. doi: 10.1136/bmjopen-2018-028093

<table>
<tr><td>1 centre</td><td>Mean 67.5 (SD 8.9); 66.6 (8.4)
81%:74%</td><td colspan="2">prior to cement application and deflated when cement hardened</td><td colspan="2">skin incision and deflated when cement hardened</td><td>Low risk of bias. However, RCT terminated early due to increased need for blood transfusion in short duration tourniquet group.
No separate pain outcome. Total OKS (0-48) at 52 weeks higher in long-duration group reflecting better recovery than short duration group but not significantly (p=0.12). Mean difference approximately 5 which is greater than MCID of 4[78].
Transfusions: 10; 2. Patient reported adverse event: 26; 12</td></tr>
<tr><td rowspan="2">Abdel-Salam and Eyres 1995[119]
UK
Date not stated
1 surgeon</td><td rowspan="2">Primary unilateral TKR of which 91% osteoarthritis
40; 40
Mean 72 (range 65-80); 74 (64-82)
57.5%; 62.5%</td><td colspan="4">Tourniquet placed around thigh</td><td>1 and 2 years</td></tr>
<tr><td colspan="2">Limb exsanguinated for 2 minutes and tourniquet inflated to twice systolic blood pressure</td><td colspan="2">Tourniquet not inflated</td><td>0; 0
Unclear risk of bias due to limited reporting of methods. No pain measure or PROM
Surgeon recorded HSS score at 1 year 90 (78-97); 91 (80-97). Not significantly different at 1 or 2 years.
Blood loss similar between groups. Wound infections: 5;0. DVT: 4;0</td></tr>
<tr><td rowspan="2">Şükür et al.2016[120]
Turkey
2015
1 surgeon</td><td rowspan="2">Primary unilateral TKR, in women with osteoarthritis
30; 30; 30; 30
Mean 67.0 (SD 7.0); 66.9 (8.5); 68.4 (6.9); 68.4 (6.8)
100%</td><td colspan="4">Pneumatic tourniquet inflated to 125mm Hg above systolic blood pressure</td><td>6 months</td></tr>
<tr><td>Knee in 90° flexion and tourniquet deflated during wound closure</td><td>Knee in 90° flexion and tourniquet inflated during wound closure</td><td>Knee in full extension and tourniquet deflated during wound closure</td><td>Knee in full extension and tourniquet inflated during wound closure</td><td>0;0;0;0
High risk of bias. KSS outcome noted in methods but not presented in results.
KSS results not reported at 6 months but no significant differences between groups at 3 months.
Surgical and wound complications similar between groups. No infections, fractures or instability requiring revision within 6 months</td></tr>
<tr><td></td><td></td><td colspan="4">Blood transfusion if required</td><td>3-22 months, mean 12;13 months</td></tr>
</table>

Beswick AD, *et al. BMJ Open* 2019; 9:e028093. doi: 10.1136/bmjopen-2018-028093

<table>
<tr><td rowspan="1">Zhang et al.2016 [121]
China
2014-2015
1 hospital</td><td>Primary TKR for osteoarthritis
84; 82
Not reported
Not reported</td><td colspan="3">Tourniquet</td><td colspan="3">No tourniquet</td><td>Not clear
High risk of bias. Variable follow up. HSS outcome noted in methods but not presented in results.
HSS not reported.
Transfusion rates similar between groups. At mean follow up of 12 -13 months, patients operated on without a tourniquet had a lower rate of DVT (2.4%) compared with those with a tourniquet (10.7%).</td></tr>
<tr><td rowspan="2">Zhang et al. 2017[62]
China
2008-2011
1 surgeon</td><td rowspan="2">Primary unilateral cemented TKR for osteoarthritis
50; 50; 50
Mean 70.3 (SD 6.6); 71 (10.2); 68.2 (6.8)
54%; 60%; 50%</td><td colspan="6">Tourniquet inflated to 300-337mm Hg. Tranexamic acid not generally used</td><td rowspan="2">6 months
0; 0; 0
Low risk of bias
No separate pain outcome. HSS similar between groups at 6 months (p=0.839).
At 2 weeks DVT: 0; 0; 1. Intramuscular vein thrombosis: 4; 3; 3. Transfusions: 30%; 26%; 10%</td></tr>
<tr><td colspan="2">Tourniquet for entire operation</td><td colspan="2">Tourniquet removed before wound closure</td><td colspan="2">Tourniquet from first bone osteotomy until wound closure</td></tr>
<tr><td rowspan="2">Huang et al. 2017[60]
China
2015
1 centre</td><td rowspan="2">Primary unilateral TKR for osteoarthritis
50; 50
Mean 66.2 (SD 8.3); 65.1 (6.8)
64%; 68%</td><td colspan="6">Tranexamic acid</td><td rowspan="2">6 months
0; 0
Low risk of bias
VAS pain similar between groups at 6 months (p=0.728). Mean HSS score 90.3 (SD 3.2); 91.2 (2.5). P=0.151
DVT: 0; 0. PE: 0; 0. Intramuscular venous thrombosis: 6; 4. Superficial infection: 1; 0. Wound secretion: 6; 0. No significant difference in blood loss between groups.</td></tr>
<tr><td colspan="3">Tourniquet</td><td colspan="3">No tourniquet</td></tr>
</table>

4. Compression bandage

**Author**	**Indication**	**Common treatments**	**Follow up**

**Country**<br>**Recruitment dates**<br>**Setting**	**Number randomised intervention; control**<br>**Age**<br>**% female**	**Intervention**	**Control**	**Losses to follow up intervention; control**<br>**Risk of bias issues**<br>**Key results**
Brock et al. 2017[70]<br>UK<br>2013-2014<br>1 hospital	Primary unilateral TKR for osteoarthritis<br>25; 25 (24 received intervention)<br>Mean 67.3 (SD 8.2); 69.5 (6.8)<br>66.7%; 64.0%	Hydrocolloid dressing left in place until clips removed on day 10-14<br>Soft inner layer with compressive outer layer bandage. Removed after 24 hours.	Standard bandaging with soft inner layer and crepe bandage outer layer. Removed after 24 hours and cryocuff used.	6 months<br>0; 0 of patients receiving intervention<br>Low risk of bias<br>No separate pain outcome. Mean OKS similar between groups at 6 months: 35.8 (SD 7.7); 34.3 (10.6). P=0.58<br>No infections or thromboembolic events in either group

5. Blood conservation

**Author**<br>**Country**<br>**Recruitment dates**<br>**Setting**	**Indication**<br>**Number randomised intervention; control**<br>**Age**<br>**% female**	**Common blood conservation strategies**		**Follow up**<br>**Losses to follow up intervention; control**<br>**Risk of bias issues**<br>**Key results**
		**Intervention**	**Control**	
Tranexamic acid				
Sa-Ngasoongsong et al. 2011[64]<br>Thailand<br>2008-2009<br>1 hospital	Primary knee osteoarthritis with unilateral primary cemented computer assisted TKR<br>24; 24<br>69.0 (SD 8.2); 69.2 (7.6)<br>91.7%; 75%	Drain and compressive dressing<br>25ml saline solution containing 250mg tranexamic acid injected into knee joint after fascial closure	25ml saline solution injected into knee joint after fascial closure	6 months<br>0; 0<br>Low risk of bias<br>No separate pain score reported but WOMAC overall score mean 18.6 (SD 7.6); 20.8 (6.4). P=0.282<br>Lower peri-operative blood loss in tranexamic acid group and need for blood transfusion, 1/24 compared with 8/24 in control group. No DVT,

<table>
<tr><td></td><td></td><td colspan="3"></td><td colspan="3"></td><td>wound complications or infection reported in either group</td></tr>
<tr><td rowspan="2">Kim et al. 2014[63]
Korea
2009-2011
1 hospital</td><td rowspan="2">Primary unilateral TKR for osteoarthritis
90; 90
Mean 73.5 (SD 5.5); 71.9 (SD 5.9)
88%; 87%</td><td colspan="6">Tourniquet, drain, compressive dressing. Allogenic blood transfusion and intravenous iron and erythropoietin if required</td><td rowspan="2">1 year
0; 0
Low risk of bias
WOMAC pain mean 3.2 (2.6); 2.8 (2.3). Difference not statistically significant
Lower blood loss and need for allogenic transfusion in tranexamic acid group. No DVT. 1 PE in control group.</td></tr>
<tr><td colspan="3">10 mg/kg body weight tranexamic acid in 100 mL of normal saline given as slow intravenous injection 30 min before tourniquet deflation, and the same amount 3 hours later.</td><td colspan="3">No tranexamic acid and no placebo</td></tr>
<tr><td rowspan="2">Sa-Ngasoongsong et al. 2013[65]
Thailand
2010-2011
1 hospital</td><td rowspan="2">Primary unilateral cemented TKR for osteoarthritis
45; 45; 45
Mean 68.1 (SD 6.2); 67.6 (8.7); 66.2 (7.3)
88.9%; 93.3%; 95.6%</td><td colspan="6">Drain and compressive dressing</td><td rowspan="2">1 year
0; 0; 0
Low risk of bias
No separate pain outcome but WOMAC mean 14.5 (7.1); 15.1 (6.2); 15.5 (6.6). P=0.42
Total blood and Hb loss lower in intervention groups than control. Fewer transfusions in 500mg (0) than 250mg tranexamic acid group (6) and control group (10). 2 DVT in 500mg group. 1 DVT in 250mg group. 1 PE and 3 DVT in control group. No infections.</td></tr>
<tr><td colspan="2">25ml saline solution containing 500mg tranexamic acid injected into knee joint after fascial closure via drain tube.</td><td colspan="2">25ml saline solution containing 250mg tranexamic acid injected into knee joint after fascial closure via drain tube.</td><td colspan="2">25ml saline solution injected into knee joint after fascial closure</td></tr>
<tr><td rowspan="2">Hourlier et al. 2015[67]
France
2009-2010
1 hospital</td><td rowspan="2">Primary unilateral TKR for osteoarthritis
52; 54
74 (SD 6); 72 (7)
62%; 63%</td><td colspan="6">Tourniquet, electrocautery, routine haemostasis, superficial drain. No blood salvage system.</td><td rowspan="2">6 months
0; 0
Low risk of bias.
No separate pain score but KSS clinical score mean 90 (SD 6); 90 (13). P=0.90
No difference between groups in total blood loss. 1 MUA in single treatment</td></tr>
<tr><td colspan="3">10 mg/kg intra-operative tranexamic infusion. After 2 hours, continuous infusion of tranexamic acid 2 mg/kg/hr for 20 hours via electric syringe</td><td colspan="3">single bolus of 30 mg/kg tranexamic acid as an intraoperative infusion. After 2 hours, placebo saline continuous infusion via electric syringe</td></tr>
</table>

				group. No deep infections or revisions.
Huang et al. 2017[60]<br>China<br>2015<br>1 centre	Primary unilateral TKR for osteoarthritis<br>50; 50<br>Mean 66.2 (SD 8.3); 65.8 (6.3)<br>64%; 70%	Tourniquet inflated to 100mm Hg above SBP before incision and deflated after wound closure<br>Intravenous tranexamic acid 20mg/kg before incision and tranexamic acid 10mg/kg at 3, 6, 12 and 24 hours. 1g tranexamic acid in 50ml saline irrigated into wound during operation	No treatment with tranexamic acid	6 months<br>0; 0<br>Low risk of bias<br>VAS pain similar between groups at 6 months (p=0.728). Mean HSS score (0-100) better in tranexamic acid group than controls: 90.3 (SD 3.2); 88.9 (3.0). P<0.001. Mean difference 1.4 lower than MCID of 8.29[81]<br>Greater blood loss in control group than tranexamic group (p<0.001). DVT: 0; 0. PE: 0; 0. Intramuscular venous thrombosis: 6; 3. Superficial infection: 1; 3. Wound secretion: 6; 9.
Thrombin infusion				
Kusuma et al. 2013[68]<br>USA<br>Not stated<br>1 hospital	Primary unilateral TKR for osteoarthritis<br>40; 40<br>Mean 64.6 (SD 10.2); 64.5 (7.3)<br>82.5%; 67.5%	Tourniquet, drain, Esmarch bandage, electrocautery<br>20,000 IU thrombin infusion (1,000 IU/mL) through fascial defect	Closure and drain placement protocol without the thrombin infusion.	1 year (6 months and 2 years also reported)<br>0; 0<br>Low risk of bias<br>No separate pain outcome. KSS mean 95.5; 96.0. p=0.45<br>Lower drop in Hb in thrombin group. Blood transfusion in 4 intervention and 7 control patients. 1 control patient had haematoma. No hospital readmissions
Flexion vs extension				
Napier et al. 2014[69]<br>UK<br>2003-2004<br>1 hospital	Primary unilateral TKR of which 89% for osteoarthritis<br>90; 90	No drains or tranexamic acid<br>Flexion. Operated knee kept in passive flexion (120°) post-operatively for 6 hours using a jig. Wound redressed and placed in flexion over a	Extension. Operated knee kept in full passive extension	1 year<br>5; 1 (12 did not attend follow up)<br>Low risk of bias.<br>No separate pain outcome. OKS mean 20.5 (SD9.0); 22.1 (9.7). P=0.27

Beswick AD, *et al. BMJ Open* 2019; 9:e028093. doi: 10.1136/bmjopen-2018-028093

	Mean 70.4 (SD 9.9) 71.0 (7.6)<br>74%; 64%	single pillow until POD1 morning.		1 MI and 1 DVT in each group. 1 haematoma in flexion group. 1 deep infection and 1 extensor muscle weakness in extension group. More transfusions in extension group (p=0.002)
Auto-transfusion of washed blood				
Thomas et al. 2001[122]<br>UK<br>Not stated<br>1 hospital	Unilateral TKR<br>115; 116<br>Mean 69.3 (range 32-95); 70.0 (40-88)<br>62%; 53%	Allogenic transfusion if Hb fell below 9g/dl		6 months<br>Losses to follow up not reported<br>Unclear risk of bias due to limited details of methods and follow up.<br>No separate pain outcome. No significant difference in EQ-5D between groups.<br>7% of auto-transfusion group required allogenic transfusion compared with 28% in control group. Fewer infections, readmissions and GP visits in auto-transfusion group. No significant differences in other serious adverse events or mortality between groups.
		Auto-transfusion of wound drainage if volume >125ml post-operative. Blood washed and re-suspended before re-infusion using a centrifugal cell washing machine	Wound drainage discarded	

6. Platelet rich plasma

**Author**<br>**Country**<br>**Recruitment dates**<br>**Setting**	**Indication**<br>**Number randomised intervention; control**<br>**Age**<br>**% female**	**Common blood conservation strategies**		**Follow up**<br>**Losses to follow up intervention; control**<br>**Risk of bias issues**<br>**Key results**
		**Group 1 (intervention)**	**Group C (control)**	
Aggarwal et al. 2014[123]	Primary unilateral surgery or first surgery of staged	Tourniquet. No tranexamic acid or suction drain. Blood transfusion if necessary due to intraoperative blood loss or postoperative haemoglobin <8g/dl.		6 months<br>No losses to follow up reported

Beswick AD, *et al. BMJ Open* 2019; 9:e028093. doi: 10.1136/bmjopen-2018-028093

India<br>2010-2011<br>1 surgeon	bilateral TKR for osteoarthritis<br>7; 14<br>Mean 56.43 (SD 7.59); 53.79 (9.75)<br>Sex not stated	8 ml PRP, prepared from patient's blood. Calcium chloride for activation given in a separate syringe in 4:1 ratio. PRP and calcium chloride injected into the posterior recess, gutters and capsule, and repaired extensor mechanism and prepatellar fat.	No treatment	High risk of bias due to unexplained differences in numbers of patients in randomised groups.<br>No separate pain outcome. WOMAC total at 6 months PRP mean 7.14 (SE 0.69), controls 7.86 (1.23), p=0.173<br>PRP group had lower fall in haemoglobin and need for blood transfusion

7. Cryotherapy

**Author**<br>**Country**<br>**Recruitment dates**<br>**Setting**	**Indication**<br>**Number randomised intervention; control**<br>**Age**<br>**% female**	**Common treatment**		**Follow up**<br>**Losses to follow up intervention; control**<br>**Risk of bias issues**<br>**Key results**
		**Group 1 (intervention)**	**Group 1 (intervention)**	
Wang 2017[124]<br>China<br>2013-2015	Unilateral TKR for osteoarthritis<br>53; 53<br>Mean 65.23 (SD 5.41); 64.97(5.36)<br>62.3%; 58.5%	CPM for 2 weeks		6 months<br>0; 0<br>Unclear risk of bias due to limited reporting<br>No separate pain outcome. At 6 months 87% of cryotherapy patients had excellent or good knee function compared with 69% of controls (p=0.032).<br>No adverse events reported in either group during functional training
		Compression cold therapy for 48 hours	No compression cold therapy	

8. Denusomab

**Author**<br>**Country**<br>**Recruitment dates**<br>**Setting**	**Indication**<br>**Number randomised intervention; control**<br>**Age**<br>**% female**	**Common treatment**		**Follow up**<br>**Losses to follow up intervention; control**<br>**Risk of bias issues**<br>**Key results**
		**Group 1 (intervention)**	**Group 1 (intervention)**	
Ledin et al. 2017[72]<br>Sweden<br>2012-2014<br>2 centres	Elective cemented primary unilateral TKR for osteoarthritis<br>25; 25<br>Mean 66 (SD 6.3); 64 (5.5)<br>60%; 60%			12, 24 months<br>0; 2<br>Low risk of bias<br>No significant differences in KOOS pain or other KOOS domains between groups 12 12 or 24 months<br>No suspected unexpected adverse reactions in either group
		Injection of 60mg denusomab 1 day after surgery and after 6 months	Injection of placebo 1 day after surgery and after 6 months	

9. Continuous passive motion

**Author**<br>**Country**<br>**Recruitment dates**<br>**Setting**	**Indication**<br>**Number randomised intervention; control**<br>**Age**<br>**% female**	**Common treatment**			**Follow up**<br>**Losses to follow up intervention; control**<br>**Risk of bias issues**<br>**Key results**
		**Group 1 (intervention)**	**Group 2 (intervention)**	**Group C (control)**	
Leach et al. 2006[125]<br>UK<br>Before 2005<br>1 hospital	Unilateral cruciate retaining rotating platform TKR for osteoarthritis<br>85 overall<br>Mean 71.2 (range 53-84); 72.9 (52-89)	Physiotherapy protocol from POD1 including slider board exercises to improve ROM and quadriceps strengthening exercises.			6 and 12 months<br>25 patients lost to follow up<br>High risk of bias due to large loss to follow up and use of date of birth randomisation<br>No difference in mean VAS pain at 1 year, CPM 0.6; control 0.9. p=0.49
		CPM commenced on first postoperative day set at a range 0–30 and used for 1 hour twice per day. Each day,	No CPM		

	50%; 54%	range was increased by 10° with discharge at POD 5-7.			Adverse events not reported
Sahin et al. 2006[126]<br>Turkey<br>Before 2006<br>1 hospital	Primary unilateral TKR for osteoarthritis<br>15; 16<br>Mean 61 (SD 6.0); 61.6 (7.5)<br>86%; 86%	Standard physiotherapy<br>From POD 1, CPM 2.5 hours 2x/day. Initially 0-40° flexion and increased by 10° each day until POD 7	No CPM		6 months<br>3 lost to follow up<br>Unclear risk of bias as patients were followed up by treating physician.<br>Mean difference in VAS pain 0.1/10 slightly favouring no CPM group (95% CI -0.8, 0.9; P=0.87)<br>Adverse events not known
Pope et al. 1997[127]<br>Australia<br>1988-1999<br>1 hospital	Primary unilateral or bilateral TKR of which 86% for osteoarthritis<br>62 (70 knees). Authors excluded those not followed up so groups were 18; 20; 19<br>Mean 72.5 (95% CI 64.4, 74.98); 72.7 (70.4, 75.0); 69.4 (64.4, 74.98)<br>64.7%; 50%; 72.2%	Physiotherapy commenced on postoperative day 1<br>Patients had an initial CPM range of 0-40° increased by 10° twice, on day after surgery and day 2, so that 0-60° flexion achieved before removal of machine at 48 hours	Patients had an initial CPM range of 0-70° increased by 10° twice, on day after surgery and day 2, so that 0-90° flexion achieved before removal of machine at 48 hours	Knee placed in an extension splint in the recovery room	6 and 12 months<br>8 patients (12 knees) excluding 1 death<br>High risk of bias due to losses to follow up and limited reporting of methods<br>No separate pain outcome. However, "pain disability" contributed up to 50 points out of a total of 70-point functional score (70 best outcome). No difference between groups in functional score: CPM 0-40 median 56 (range 20, 70); CPM 0-70 52 (10, 70); no CPM 52 (25, 70). p=0.80<br>CPM groups had greater blood loss than controls, p=0.008). 1 manipulation under anaesthesia in no CPM group, 2 revisions due to patellar dislocation in the 0-40 CPM group, 1 PE death in the 0-70 CPM group.
Beaupré etal. 2001[128]	Primary unilateral TKR of which	Standardised exercise during hospital admission which included a slider board session.			6 months

<table>
<tr><td>Canada
1997-1998
1 hospital</td><td>92% for osteoarthritis
40; 40; 40
Mean 68 (SD 9); 68 (9); 69 (8)
52.5%; 50%; 30%</td><td>3 sessions (2 hours) with CPM machine per day from POD2. Range increased from starting range 0-30 degrees as tolerated.</td><td colspan="2">Minimum of two 10-minute slider board therapy sessions per day in addition to one in the standardised exercise. Active knee flexion and extension in sitting and lying positions performed independently as tolerated.</td><td>No intervention further than standardised exercise.</td><td>6; 8; 6
Unclear risk of bias due to losses to follow up
Mean WOMAC pain at 6 months: 76 (15); 85 (15); 79 (16). No difference over time between groups, p=0.62.
Long-term adverse events. Need for MUA: 1; 1; 0. DVT: 0; 1; 0. Cellulitis: 0; 0; 1. Infection 0; 0; 1.</td></tr>
<tr><td rowspan="2">Kumar et al. 1996[129]
USA
Before 1996
1 hospital</td><td rowspan="2">Primary unilateral TKR for osteoarthritis
40 (46 knees); 33 (37)
Mean 69 (range 52-86); 68 (42-88)
58%; 67%</td><td colspan="4">Standard physiotherapy</td><td rowspan="2">6 months
15; 13 lost to follow up
High risk of bias due to large losses to follow up
No separate pain outcome. KSS CPM 82.7; Drop and dangle 80.7. p=0.78
Haematoma 3;1. Closed manipulation 1;3. DVT 0;0. PE 0;1</td></tr>
<tr><td colspan="2">CPM from POD 0. Initially 10 hours/ day 0-90° until discharge</td><td colspan="2">No CPM. Passive range of movement (“drop and dangle”) to 90° 2x/ day initially for 20 minutes, later 30-45 minutes.</td></tr>
<tr><td rowspan="2">Worland et al. 1998[130]
USA
1996
1 hospital</td><td rowspan="2">Unilateral or bilateral TKR for osteoarthritis.
91 patients (114 knees randomised). After post-randomisation exclusions: 37 (49 knees); 43 (54 knees)
Mean 70.2 (range 44-84)
66.25%</td><td colspan="4">CPM and physiotherapy during hospital admission</td><td rowspan="2">6 months
11 patients (11 knees)
Unclear risk of bias due to post-operative exclusions not reported separately for groups and limited reporting of methods.
No separate pain outcome. At 6 months, mean HSS score CPM 95.3 (SD 2.8); physiotherapy 95.7 (3.0). P=0.49.
Adverse events not reported.</td></tr>
<tr><td colspan="2">At home after discharge, CPM machine 3 hours per day on replaced knee for l0 days.</td><td colspan="2">Physical therapist home visit 1 hour three times per week for 2 weeks</td></tr>
</table>

MacDonald et al. 2000[131]<br>Canada<br>Before 2000<br>1 hospital	Primary unilateral TKR for osteoarthritis<br>40; 40; 40<br>Age and sex not reported	Active ROM, passive ROM exercises, mobilised as tolerated using walker or crutches.			6 and 12 months<br>Not reported<br>Unclear risk of bias due to limited and selective reporting.<br>No separate pain outcome. No statistical differences between groups for KSS at 6 and 12 months.<br>Adverse events not reported
		CPM commenced POD 0. Initially 0-50 degrees. Provided for 18-24 hour/ day. Increased by 10 degrees/ hour as tolerated. Continued until POD 1	CPM commenced POD 0. Initially 70-110 degrees. Provided for 18-24 hr/ day. Not increased.<br>Continued until POD 1	No CPM	
Bennett et al. 2005[74]<br>Australia<br>1997-2000<br>1 hospital	Primary unilateral TKR for osteoarthritis<br>47; 48; 52<br>70.7; 71.4; 71.7<br>72.3%; 64.6%; 67.3%	Standard in hospital physiotherapy programme			12 months<br>1 patient excluded due to inability to achieve 90° flexion<br>Low risk of bias<br>No separate pain outcome. No significant difference in KSS between groups at 1 year.<br>No difference in wound healing between groups
		Standard CPM from 0° to 40° for 2x3 hours on POD 1 increased by 10° per day until POD 6. Extension splint applied overnight	Early flexion CPM commenced in recovery room from 90° to 50° knee flexion. Increased gradually to CPM 90° to 0° for 2x3 hours in day 4-6.	No CPM	
Ersözlü et al. 2009[73]<br>Turkey<br>2003-2004	Primary unilateral TKR for osteoarthritis<br>30; 30; 30<br>Mean 65 (range 54-73); 61 (49-80); 62 (52-78)<br>66%; 55%; 57%	Conventional physical therapy			2 years<br>1; 1; 2<br>Low risk of bias<br>No separate pain outcome. KSS scores 98; 95; 92. No significant difference between groups p=0.67.<br>Infection 0; 0; 1. Arrhythmia 0; 1; 0. No difference in complications between groups
		CPM set at 30-40° from POD1. Increased as tolerated to POD7. 1 hour CPM 3x/day.	CPM set at 60-70° from POD3. Increased by 10°/ day to POD7. 1 hour CPM 3x/day.	No CPM	

10. Electrical stimulation

Author	Indication	Common rehabilitation strategies	

**Country**<br>**Recruitment dates**<br>**Setting**	**Number randomised intervention; control**<br>**Age**<br>**% female**	**Intervention**	**Intervention**	**Common rehabilitation strategies**
Avramidis et al. 2011[75]<br>Greece<br>2005-2006<br>1 hospital	Elective primary unilateral TKR for osteoarthritis<br>38; 38<br>Mean 70.54 (SD 4.68); 70.66 (3.73)<br>80%; 82.9%	Standard physiotherapy for 6 weeks. No CPM		1 year<br>3 (intervention intolerance); 3<br>Low risk of bias<br>Improved SF-36 bodily pain at 1 year in intervention group compared with control, mean 92 (SD 10.57); 79.48 (12.72). P<0.001. Difference of 12.52 close to MCID of 16.86[82]. No difference in OKS or American KSS<br>Adverse events not reported
		Transcutaneous electric muscle stimulation of the vastus medialis muscle from POD2 2x/ day for 2 hours for 6 weeks.	No intervention	
Stevens-Lapsley et al. 2012[132]<br>USA<br>2006-2010<br>1 hospital	Primary unilateral TKR for osteoarthritis<br>35; 31<br>Mean 66.2 (SD 9.1); 64.8 (7.7)<br>57.1%; 51.6%	Standard inpatient rehabilitation, home and outpatient physical therapy		6 months and 1 year<br>5; 6<br>Unclear risk of bias due to baseline differences in WOMAC<br>No difference in resting pain (points) at 1 year intervention mean 0.6 (SD 1.4); control 0.4 (1.5). Also similar at 6 months. Mean WOMAC total score better at 1 year in intervention group compared with control, 5.7 (5.9); 10.0 (12.2) and at 6 months. However, probably explained by baseline differences. Authors state no differences for change in WOMAC.<br>DVT 1; 0. Unspecified complication 1; 0. Infection 0; 2. Revision 0; 1
		Neuromuscular electrical stimulation commenced on POD2 for 6 weeks 2x/ day.	No intervention	

Beswick AD, *et al. BMJ Open* 2019; 9:e028093. doi: 10.1136/bmjopen-2018-028093

Levine et al. 2013[133]<br>USA<br>Before 2013<br>1 surgeon	Elective unilateral TKR for osteoarthritis<br>35; 35<br>Mean 68.1; 65.1<br>76%; 62%	2 sessions of ROM exercise		6 months<br>5; 9<br>Unclear risk of bias due to large uneven losses to follow up<br>KSS pain favoured intervention at 6 months but not significantly 79.08 (SD 10.97); 75.5 (14.77); 95%CI for difference -3.78, 10.93. Similar for WOMAC total score, 95%CI for difference -3.19, 14.81.<br>Confusion 2; 0
		Neuromuscular electrical stimulation commenced 14 days pre-operatively until 1 day before surgery. Recommenced at POD1 for 60 days. After hospital discharge no direct contact with a physical therapist	Formal physical therapy programme with progressive resistive exercises and strengthening in hospital and after discharge supervised by physical therapist.	
Moretti et al. 2012[77]<br>Italy<br>2008-2010<br>1 hospital	Primary unilateral TKR for osteoarthritis<br>15; 15<br>Mean 70.0 (SD 10.6); 70.5 (8.1)<br>Not reported	Rehabilitation protocol including CPM		6 and 12 months<br>No losses to follow up<br>Low risk of bias<br>Mean VAS pain (10-point scale) lower at 12 months in intervention group compared with control, 0.5 (SD 1.3); 3.6 (3.9). $p< 0.05$. Mean difference of 2.1 (10-point scale) greater than MCID of 16.1 (100-point scale)[83]<br>Difference also at 6 months.<br>More swelling of the knee in intervention patients than controls, statistically significant at 1 and 2 months
		Pulsed electromagnetic fields (I-ONE therapy) from POD7, 4 hours/ day for 60 days	No intervention	
Adravanti et al. 2014[134]<br>Italy<br>1 hospital	Primary unilateral TKR for osteoarthritis<br>16; 17<br>Mean 66 (SD 13); 73 (5)<br>62.5%; 52.9%	Standard rehabilitation protocol: active and passive mobilisation		6 months<br>4; 3<br>High risk of bias: small study, proportionately high losses to follow up<br>At 6 months, mean VAS pain in intervention group lower than in controls ($p<0.05$). At 3 years, 1/14 intervention patients and 4/12 controls reported severe pain
		Pulsed electromagnetic fields (I-ONE therapy) by POD7 for 4 hours/ day for 60 days	No intervention	

Beswick AD, *et al. BMJ Open* 2019; 9:e028093. doi: 10.1136/bmjopen-2018-028093

				No difference between groups in swelling at 6 months.

11. Rehabilitation

Author<br>Country<br>Recruitment dates<br>Setting	Indication<br>Number randomised intervention; control<br>Age<br>% female	Common rehabilitation strategies		Follow up<br>Losses to follow up intervention; control<br>Risk of bias issues<br>Key results
		**Intervention**	**Control**	
Walking guidance and training				
Li et al. 2017[79]<br>China<br>2015-2016<br>1 hospital	Primary unilateral TKR for osteoarthritis<br>43; 43<br>Mean 76.33 (SD 5.28); 78.47 (5.50)<br>55.8%; 51.2%	Before TKR, general guidance on joint activities, quadriceps muscle strength, use of aids, diet guidance, correct walking methods and precautions.<br>Knee passive flexion and extension to 90° and quadriceps muscle strength training commenced on POD 1. POD 3-7, straight leg raising exercises. 2 weeks after replacement, increased joint activities and muscle strength training, centre of gravity transfer training, limb weight training, and walking training.		6 months<br>0; 0<br>Low risk of bias<br>Mean VAS pain at 6 months: 0.51 (SD 0.74); 2.83 (0.88) favouring walking intervention group, p<0.01. Difference of 2.42 (10 point scale) greater than the MCID of 16.1 (100-point scale)[83]. HSS scores at 6 months favoured intervention, p<0.01.<br>No infection, allergic reaction or immune reaction in either group. Intervention not associated with swelling, pain, prosthesis loosening, thrombosis, or delayed wound healing
		Standing, weight and balance exercises from POD 1. From POD 2, walking guidance and training.	No additional rehabilitation	
Aquatic therapy				
Liebs et al. 2012[81]<br>Germany<br>2003-2004<br>4 hospitals	Elective primary unilateral TKR for osteoarthritis<br>87;98	Continuous passive motion machines daily after removal of suction drains. Programme of daily physiotherapy: range of motion activities; exercises for improvement of muscle tension, venous return, balance, coordination and gait; and instruction in activities of daily living.		6, 12 and 24 months<br>13.8%; 19.4% excluding deaths and unexplained reasons<br>Low risk of bias

	Mean 68.5 (SD 8.6); 70.9 (7.5)<br>70.1%;73.5%	Aquatic therapy for 30 minutes 3 times a week up to postoperative week 5. Pool exercises aimed at training of proprioception, coordination and strengthening with aid of float cuffs, training kickboards and bar floats.			WOMAC pain at 12 months: early aquatic mean 13.2 (SD 15.0); late aquatic 17.4 (22.4) p=0.22. No difference at 6 and 24 months.<br>5 early aquatic therapy patients and 1 late aquatic therapy patient readmitted to hospital within 3 months. 2 early aquatic patients and 1 late aquatic patient readmission directly or indirectly related to the intervention.
		Aquatic therapy beginning on the 6th postoperative day with the wound covered with a waterproof adhesive dressing.	Aquatic therapy as pool exercise after the completion of wound healing on the 14th postoperative day		
Rahmann et al. 2009[135]<br>Australia<br>2003-2005<br>1 hospital with 2 surgeons	Unilateral primary TKR or THR for osteoarthritis (50% TKR)<br>18;19;17 (11 had been excluded post-randomisation due to complications in hospital<br>Mean 69.4 (SD 6.5); 69.0 (8.9); 70.4 (9.2)<br>44.4%; 63.2%; 70.6%	Standard ward-based physiotherapy until day 3. 1 ward physiotherapy treatment per day. Surgical wounds covered with an occlusive, waterproof dressing. 40 mins/ day.			6 months<br>4;2;0 for combined THR and TKR<br>Unclear risk of bias as TKR patients more likely to receive ward-based control intervention. THR and TKR analysed together<br>No difference in overall WOMAC outcome at 6 months in THR and TKR patients combined between aquatic at fast pace and ward-based (p=0.929) and aquatic at 2 paces (p=0.872).<br>No adverse events reported after intervention commenced.
		From day 4, 1 to 1 individual physiotherapy. Aquatic physiotherapy programme to maximize function and strength. 40 mins/ day. Fast pace metronome 80-88 bpm	From day 4, 1 to 1 individual physiotherapy. Water exercise programme with general exercises not targeted at specific functional retraining in the aquatic environment. Slow pace metronome 50-58 bpm	From day 4, 1 to 1 individual ward-based physiotherapy. 40 mins/ day	
Supported early discharge					
Mahomed et al. 2008[82]<br>Canada<br>2000-2002<br>2 centres	Primary unilateral TKR or THR for osteoarthritis (approximately 50% TKR)<br>119;115<br>68	Inpatient physiotherapy			12 months<br>No losses to follow up<br>Low risk of bias (analysis by actual treatment received showed similar results)<br>WOMAC pain at 12 months marginally favoured home-based
		Discharged home when able to independently transfer supine to sitting and sitting to standing, walk 30 metres and climb stairs if necessary. Physiotherapist home visit within 48 hours and	Transfer to independent rehabilitation centre for 14 day stay.		

	About 67% women	subsequent management along a multidisciplinary clinical pathway (4-16 visits). Then outpatient physiotherapy or self-directed programme.		rehabilitation mean 87 (SD 16); 83 SD (20), p=0.08 but this was not statistically significant. Mean difference of 4 less than MCID of 8-9[77]. Results did not differ between TKR and THR patients.<br>Similar rates of dislocation, DVT and readmissions between groups. 2% inpatient group developed infections compared with 0 in home group
Hill et al. 2000[136]<br>UK<br>1997-1998<br>1 centre	Unilateral, primary TKR, irrespective of diagnosis or concomitant disease<br>70 randomised, with 32;28 eligible for trial at day 5	Care pathway for medical, nursing and physiotherapy care from admission until day 5<br>Outreach team domiciliary visit prior to admission with assessment of home environment. At days 5–7, patients assessed to ensure discharge safe. Outreach team visit on day of discharge with further visits as required. 1+ physiotherapist visit linked with nurses to monitor knee performance. Discharge when skin clips removed, wound healed and specialist orthopaedic assistance not required, usually day 10–12	Inpatient care until removal of skin clips and wound healing.	1 year<br>No losses to follow up reported after commencement of intervention<br>Unclear risk of bias due to limited reporting of methods.<br>No pain outcome or patient reported outcome. Control group had better mean KSS scores, but this did not reach statistical significance at 1 year or earlier.<br>1;1 serious infection, other wound infections 1;6, painful joints 9;4, other minor complications similar between groups
Flexion or extension during knee closure				
Wang et al. 2014[80]<br>China<br>2009-2010<br>1 centre	Primary unilateral TKR for osteoarthritis<br>40; 40<br>Mean 68.34 (SD 7.09), 67.87 (6.47)<br>17.5%; 22.5%	No patellar replacement or lateral retinacular release<br>Articular capsule, soft tissue and skin enclosed in 90° flexion which was maintained for 1-2 min after wound closure.	Wound closure performed in full extension	6 months<br>No losses to follow up<br>Low risk of bias<br>Mean VAS pain in flexion group 1.15 (SD 0.73); extension group 1.12 (0.68), p=0.64

Beswick AD, *et al. BMJ Open* 2019; 9:e028093. doi: 10.1136/bmjopen-2018-028093

				No wound complications, patella fracture or infection requiring surgery in either group

12. Wound management

**Author**<br>**Country**<br>**Recruitment dates**<br>**Setting**	**Indication**<br>**Number randomised intervention; control**<br>**Age**<br>**% female**	**Common wound management strategies**		**Follow up**<br>**Losses to follow up intervention; control**<br>**Risk of bias issues**<br>**Key results**
		**Intervention**	**Control**	
Kong et al. 2014[71]<br>South Korea<br>2011<br>1 surgeon	Primary cemented unilateral TKR for osteoarthritis<br>50; 50<br>Mean 69.0 (SD 7.7); 68.0 (4.8)<br>89.6%; 87.5%	Skin staples removed on day 10 and wound closure strip applied for 5 days		6 and 12 months<br>2; 2 lost to follow up<br>Low risk of bias<br>At 12 months, VAS pain in silicone gel group mean 2.50 (SD 1.16); control 2.92 (1.90). P=0.201. No difference at 6 months, p=0.886.<br>No wound dehiscence or infection associated with application of silicone gel or petroleum
		After removal of wound closure strip, patients managed operation scars with application of silicone gel for 1 month after stitches removed	After removal of wound closure strip, patients managed operation scars with application of petroleum gel for 1 month after stitches removed	

13. Anabolic steroids

**Author**<br>**Country**<br>**Recruitment dates**<br>**Setting**	**Indication**<br>**Number randomised intervention; control**<br>**Age**<br>**% female**	**Common rehabilitation strategies**		**Follow up**<br>**Losses to follow up intervention; control**<br>**Risk of bias issues**<br>**Key results**
		**Intervention**	**Control**	
		Cold compression and CPM		6, 9 and 12 months

Beswick AD, *et al. BMJ Open* 2019; 9:e028093. doi: 10.1136/bmjopen-2018-028093

Hohmann et al. 2010[83]<br>Australia<br>Before 2010<br>1 surgeon	Primary unilateral TKR for osteoarthritis<br>5; 5<br>Mean 66.2 (range 58, 72); 65.2 (59, 72)<br>20%; 40%	On day 5, intramuscular injection of 50 mg Nandrolone decanoate solution. Patients visited every 2 weeks and injections continued for 6 months.	On day 5, intramuscular injection of saline. Patients visited every 2 weeks and injections continued for 6 months.	0; 0 lost to follow up<br>Low risk of bias (but small feasibility study)<br>No separate pain outcome. KSS at 12 months in intervention group mean 91.4 (SD 3.5); control 81.2 (SD 7.1). p=0.03. Difference also at 6 months (p=0.04), marginal at 9 months (p=0.06). Difference in means at 12 months of 10.2 close to MCID of 12.3[79].<br>Intervention group had smaller decrease in bone mineral density at 6 months than controls but not significant

14. Guided imagery

**Author**<br>**Country**<br>**Recruitment dates**<br>**Setting**	**Indication**<br>**Number randomised intervention; control**<br>**Age**<br>**% female**	**Common rehabilitation strategies**		**Follow up**<br>**Losses to follow up intervention; control**<br>**Risk of bias issues**<br>**Key results**
		**Intervention**	**Control**	
Jacobson et al. 2016[137]<br>USA<br>2011-2012<br>1 surgeon	Primary unilateral TKR for osteoarthritis<br>42; 40 (41; 39 received treatment)<br>Mean 65.0 SD 8.6)<br>62.2%	Participants listened to a 19-21-minute CD each day for 2 weeks before and 3 weeks after surgery. Content covered concerns and hopes about TKR with aim to facilitate mind–body connections to promote optimal TKR outcomes.	Participants listened to a 17-21-minute CD each day for 2 weeks before and 3 weeks after surgery. Content comprised poetry, short stories and essays	6 months<br>12; 10 of patients receiving treatments<br>High risk of bias due to large losses to follow up<br>Mean WOMAC pain 2.7 (SD 3.1); 3.5 (SD 3.3). P<0.001<br>Adverse events not reported

Beswick AD, *et al. BMJ Open* 2019; 9:e028093. doi: 10.1136/bmjopen-2018-028093

CD compact disc; CPM Continuous passive motion; DN4 Douleur Neuropathique 4; FNB Femoral nerve block; HSS Hospital for Special Surgery; i.v. intravenous; KOOS Knee injury and Osteoarthritis Outcome Score; KSS Knee Society Score; LIA local infiltration analgesia; NRS Numerical rating scale; OKS Oxford Knee Score; ONB obturator nerve block; PCA Patient controlled analgesia; PNB psoas nerve block; SF-36 Short Form 36 Health Survey; S-LANSS Leeds assessment of Neuropathic Symptoms and Signs Pain Scale; SNB Sciatic nerve block; TKR Total knee replacement; VAS Visual analogue scale; VTE Venous thromboembolism; WOMAC Western Ontario and McMaster Universities Osteoarthritis Index.

Beswick AD, *et al. BMJ Open* 2019; 9:e028093. doi: 10.1136/bmjopen-2018-028093

Supplementary material. Risk of bias assessment

Study	Random sequence generation	Allocation concealment	Blinding of participants and personnel	Blind outcome assessment	Incomplete outcome data	Selective reporting	Other bias	Summary
Pain management								
Albrecht et al. 2014[41]	Computer generated	Anaesthetist blind to allocation	Physiotherapists, surgeons, research assistants collecting data, and members of the Acute Pain Service were kept blinded to group allocation.	Physiotherapists, surgeons, research assistants collecting data, and members of the Acute Pain Service were kept blinded to group allocation.	ITT analysis low losses to follow up	No but not checked protocol	Study was terminated early with 61% of planned recruitment completed due to change in standard anaesthesia at hospital	Low
Anastase et al. 2014[113]	Method of the Ulm Institute of Statistics	Method of the Ulm Institute of Statistics	No	No	15:14 lost to follow up	No but not checked protocol	ASA comorbidities differed between groups	High
Aveline et al. 2014[55]	Computer generated	opaque sealed envelopes	Blinded syringes prepared by nurse not involved in study	Yes	Low losses to follow up	Consistent with short term follow up paper	No	Low
Bergeron et al. 2009[105]	Blocks of different sizes according to list preprepared by study epidemiologist	Not described	Anaesthetist not blind. Patients blind	Nurse observers collecting data blind to allocation	32/59 lost to follow up	No but not checked protocol	No	High
Buvanendran et al. 2010[56]	computer generated	Yes, physicians and nurses blind	Yes	Yes	ITT	Protocol not checked	No	Low

Choy et al. 2011[42]	Computer generated	sealed envelope	No, the catheter was removed at either day 3 or 7	Patient reported outcome. Other outcomes by blinded independent physician	Low losses to follow up	Protocol not checked but reasonable range of outcomes	No	Low
Davidson et al. 2016[111]	Computer generated	Sealed opaque envelopes	Subjects and investigators were not masked to treatment group	Subjects and investigators were not masked to treatment group. PROM	31; 29 lost to follow up	None apparent but protocol not checked	Combined data from 2 RCTs	High
Fan et al. 2016[39]	No details	sealed opaque envelopes	Patients and assessors blind to randomisation	Patients and assessors blind to randomisation	2% protocol violation	No but not checked protocol	No	Low
Foadi et al. 2017[117]	Computer generated	Not described	Not described	Patient reported outcome	>70% questionnaire return	None apparent but protocol not checked	Described as pilot study	Unclear
Gao et al. 2017[35]	Random number table	Not described	Blind to patients	Blind to observers	2; 1; 0	None apparent but protocol not checked	Groups similar at baseline	Low
Ilfeld et al. 2009[108]	Computer generated	Investigators, patients, and all clinical staff were unaware of treatment group assignments	Investigators, patients, and all clinical staff were unaware of treatment group assignments	Investigators, patients, and all clinical staff were unaware of treatment group assignments	4:1 lost to follow up	No but not checked protocol	Basal infusion halved on POD1 in 10 intervention patients compared with 3 controls	High
Ilfeld et al. 2011[109]	Computer generated tables	Solutions prepared by investigational pharmacist	Yes. Intervention and control solutions indistinguishable	Patient reported outcomes. Staff masked to treatment group	11;12 did not have 4 measures out	Protocol not checked	WOMAC and WOMAC domain scores	High

				assignment performed all measures and assessments	of 6 up to 12 months	but seems reasonable	somewhat lower pre-intervention in extended infusion group. Authors report change scores	
Macrinici et al. 2017[43]	Computer generated	Staff performing injections blind	Anaesthesiologist, surgeons, patients and physical therapists blind to allocation	Yes	3; 4 lost to follow up. 6; 3 complications	None apparent but protocol not checked	Groups similar at baseline	Low
McDonald et al. 2016[52]	Computerised blocked	Study coordinator independent of care and surgery	Patients blind. Surgeon aware of study	Outcome assessment blind to allocation	1; 4 unexplained	None apparent, protocol not checked	Groups similar at baseline	Low
Meunier et al. 2007[54]	Computer generated	Sealed envelope	Randomisation code broken after 1 year	Yes	ITT reported except for 12 month pain outcome	No but not checked protocol	M/F ratio differed	Low
Morin et al. 2005[110]	Allocated randomly	Sealed envelope	All patients received some form of nerve block. Anaesthesiologist not blind to intervention	Observers not blinded	Per protocol analysis	No but not checked protocol	Difference between groups in anesthetist's opinion of difficulty of catheter placement. BMI differed between groups	High
Motififard et al. 2017[49]	Computer generated	Study coordinator independent of care and surgery	Patients blind. Surgeon aware of study	Outcome assessment blind to allocation	3; 7 (1; 4 unexplained)	None apparent, protocol not checked	Groups similar at baseline	Low

Beswick AD, *et al. BMJ Open* 2019; 9:e028093. doi: 10.1136/bmjopen-2018-028093

Nader et al. 2012[36]	Computer generated	Opaque envelope	No	Patient reported outcome	1:1 lost to follow up	Protocol not checked but reasonable range of outcomes	FNB group somewhat higher BMI	Low
Niemeläinen et al. 2014[47]	No details	Opaque sealed envelopes	Only research nurse preparing infiltrate aware of randomisation. All other personnel unaware until after 1 year follow up	Only research nurse preparing infiltrate aware of randomisation. All other personnel unaware until after 1 year follow up	All patients who received intervention completed follow up	No but not checked protocol	No	Low
Peng et al. 2014[38]	Computer generated	Not possible	Not possible	Patient reported outcome	31:38 lost at 12 months but ITT and per-protocol analysis	No but not checked protocol	No	Low
Perrin and Purcell 2009[107]	No details	Sealed syringe code stored in pharmacy department	yes	Yes	4 failed to complete protocol	No but not checked protocol	Pilot investigation. High risk of bias due to recruitment difficulties leading to small trial	High
Reinhardt et al. 2014[40]	Computer generated	Maintained by pharmacy department for blinding	Patients blind to intervention	Blinded research assistant and partially physical therapist	0 reported lost to follow up of those who received interventions	No but not checked protocol	No	Low
Seah et al. 2011[53]	Randomisation tables	Sealed envelopes. Anaesthetist and surgeon blind before opening of	Blinding of patients not stated	Blind outcome assessors and PROMs	No losses to follow up reported	No but not checked protocol	No	Low

Beswick AD, *et al. BMJ Open* 2019; 9:e028093. doi: 10.1136/bmjopen-2018-028093

		sealed envelope						
Shum et al. 2009[106]	No details	No details	Anaesthetist performing the blocks was not involved in the postoperative follow-up and data collection	Patient reported	14% and 20%	No but not checked protocol	Mean patient weight lower in no FNB group. More favourable mean OKS in no FNB group. Two groups combined for 2 year outcome but not for earlier	High
Spreng et al. 2012[115], Spreng et al. 2010[116]	Hospital pharmacy	Epidural catheter or sham set-up taped along the back of the patient and connected to an infusion pump covered in an opaque bag. Also sham knee catheter	Patients blind	Blind outcome assessment	13%	Limited reporting in conference abstract	Conference abstract only so limited information additional to early follow up paper	Unclear
Wang et al. 2015[112]	No details	No details	Not stated	Not stated	2:4 lost to follow up	No but not checked protocol	No	Unclear
Wegener et al. 2013[44]	No details	Opaque envelope	Patients, surgeons and researchers not blind to intervention	Patients not blinded	2:7:5 lost to follow up	No. Protocol checked	no	Low
Widmer et al. 2012[34]	Coded envelope	Coded envelope	Except for anaesthetist and surgeon	Both the investigators and patients were blinded	None reported as incomplete	No but protocol not checked	No	Low

Williams et al. 2013[51]	Computer generated	Not stated	Patients and assessors blind	Patients and assessors blind	3:1 of those who received treatment	No but not checked protocol	No	Low
Wu and Wong 2014[37]	Computer	Sealed envelopes	No	No	Available cases	No but not checked protocol	No	Low
Wylde et al. 2015[45]	Trials unit	Trials unit	Surgeon and anaesthetist not blind to allocation, Patients blind	Patients and research nurses blind to allocation	ITT with imputed data	No as per protocol	No	Low
Yue et al. 2013[114]	No details	No details	Surgeons and patients were double-blinded to the injection administered	surgeons and patients were double-blinded to the injection administered	Losses to follow up not reported	Limited reporting	No	Unclear
Myofascial trigger point dry needling								
Mayoral et al. 2013[118]	Computerised	Not described	Patient and other researchers apart from physical therapist blind	Patient outcomes	4: 5 loss to follow up	None apparent but protocol not checked	No	High
Tourniquet								
Abdel-Salam and Eyres 1995[119]	Card system	Not described	No	No	No losses to follow up	None apparent but protocol not checked	No	Unclear
Ejaz et al. 2014[58]	Block randomised	Sealed envelopes	Patients unaware	PROM	No losses to follow up of those who received treatments	None apparent but protocol not checked	No	Low
Huang et al. 2017[60]	Computer generated	Sealed opaque envelopes	Surgeons not blind. Patients blind to allocation	Data collector blind to allocation	No losses to follow up reported	None apparent but protocol	Groups similar at baseline	Low

						not checked		
Liu et al. 2014[59]	Excel	Not described	Patients blind	PROM	No losses	None apparent but protocol not checked.	No	Low
Mittal et al. 2012[61]	Computer generated	Sealed opaque envelopes	Patient blind	Outcome assessors blind. PROM	5:2	None apparent but protocol not checked	Study stopped because of high risk of transfusion in short tourniquet duration group	Low
Şükür et al.2016[120]	Computer generated	Not described	Possibly patients	Outcome assessors blind	No losses to follow up	KSS outcome noted in methods but not presented in results	No	High
Zhang et al. 2017[62]	Excel	Randomisation by blinded researcher.	Patients and nurses on ward blind	Not clear	No losses reported	None apparent but protocol not checked	Groups similar at baseline	Low
Zhang et al.2016[121]	Randomly allocated	Not clear	Not clear	Not clear	Not clear	HSS outcome noted in methods but not presented in results	No	High
Compression bandage								

Brock et al. 2017[70]	Web-based	Not specified	Not possible	No but PROMs	4; 2 of those receiving intervention	None apparent but protocol not checked	Groups similar at baseline	Low
Blood conservation								
Hourlier et al. 2015[67]	Computer generated	Opaque envelopes	Anaethsetist, surgeon and patient blind to treatment allocation	Assessors blind	No missing data	None apparent but protocol not checked	No	Low
Huang et al. 2017[60]	Computer generated	Sealed opaque envelopes	Surgeons not blind. Patients blind to allocation	Data collector blind to allocation	No losses to follow up reported	None apparent but protocol not checked	Groups similar at baseline	Low
Kim et al. 2014[63]	Computer generated	Not stated	patients blind to allocation	Clinical investigator blind to allocation	No missing data	None apparent but protocol not checked	No	Low
Kusuma et al. 2013[68]	Computer generated	Sealed envelopes	Surgeon and patient blind	Outcome assessor blind	No missing data	None apparent but protocol not checked	No	Low
Napier et al. 2014[69]	Computer generated	Sealed envelopes	Unlikely	Not stated but PROM	low losses to follow up	None apparent but protocol not checked	No	Low
Sa-Ngasoongsong et al. 2011[64]	Computer generated	Sealed envelopes	Surgeon and patient blind	Outcome assessor blind	No missing data	None apparent but	No	Low

						protocol not checked		
Sa-Ngasoongsong et al. 2013[65]	Computer generated	Sealed envelopes	Surgeon and patient blind	Outcome assessor blind	No missing data	None apparent but protocol not checked	Some difference between groups in pre-operative Hb	Low
Thomas et al. 2001[122]	Not described	not stated	Not reported	Not reported but PROM	Not reported but ITT	None apparent but protocol not checked	No	Unclear
Platelet rich plasma								
Aggarwal et al. 2014[123]	Not described	Opaque envelopes	Patients blind	Patients and examiners blind	No losses to follow up reported	None apparent but protocol not checked	Odd numbers in groups from randomisation	High
Cryotherapy								
Wang 2017[124]	No details	No details	No details	No details	No losses to follow up	None apparent but protocol not checked	Groups similar at baseline	Unclear
Denusomab								
Ledin et al. 2017[72]	Randomisation list produced by the study monitor	Syringes prepared independently	Investigators and patients blind	Unblinding was done after all the data had been locked	0; 2	None apparent but protocol not checked	No	Low
Continuous passive motion								
Beaupré et al. 2001[128]	Computer generated	Sealed envelopes	No	Researcher unaware and PROMs	6:8:6. Results carried	No	4 controls; 1 SB	Unclear

					forward for missing data		reassigned to CPM	
Bennett et al. 2005[74]	Block	Not stated	Operating surgeon blind. Patient not	Independent assessor blind	1 not included in analyses as not able to achieve 90 degree flexion	No	No	Low
Ersözlü et al. 2009[73]	Divided into groups by random selection	Not described	No	Surgeon score	A diabetic patient from the control group was excluded because of a superficial wound infection, a patient with a cardiac problem in group II due to dysrhythmia, and two patients due to insufficient follow-up.	Not apparent	No differences baseline	Unclear
Kumar et al. 1996[129]	Random number generator	Not stated	No	Not described	Large loss to follow up	Not all data clearly reported	No	High
Leach et al. 2006[125]	Allocation by date of birth	No	No	Blinded evaluation	Large loss to follow up	No	No	High
MacDonald et al. 2000[131]	Computer generated	Allocation concealed	No	Not described	Not reported	Yes, not all outcomes reported in full	No	Unclear
Pope et al. 1997[127]	Not described	Not described	Not described	Not described	No separate reporting. 8 patients (12 knees)	None apparent but protocol	No	High

					excluding 1 death	not checked		
Sahin et al. 2006[126]	Not described	Not stated	No	Followed up by treating physician	Low loss to follow up	No	No	Unclear
Worland et al. 1998[130]	Not described	Not described	No	Researcher blind	Not reported separately	None apparent but protocol not checked	No	Unclear
Electrical stimulation								
Adravanti et al. 2014[134]	Computer generated	Not described	Research assistant not involved in patient assessment	Principal investigator and all physicians in charge of clinical controls were blinded to patient allocation	78% retained at 6 months	Not apparent but protocol not checked	No	High
Avramidis et al. 2011[75]	Computer generated	Not described	No	Independent assessors blind	3 (intolerance of intervention); 3	Not apparent	Baseline similar	Low
Levine et al. 2013[133]	Drawing papers from hat	Not described	No	Not described. WOMAC PROM	5:9 for KSS pain and WOMAC	Not apparent but protocol not checked	No	Unclear
Moretti et al. 2012[77]	Computer generated	Not described	Physicians, as well as medical assessors, were blinded to the allocation of patients in the study groups	Physicians, as well as medical assessors, were blinded to the allocation of patients in the study groups	No losses reported	Not apparent but protocol not checked	No	Low
Stevens-Lapsley et al. 2012[132]	Stratified	Concealed	No	no but standardised scripts used	5; 6	Not apparent but protocol	WOMAC, BMI unequal at baseline	Unclear

						not checked		
Rehabilitation								
Hill et al. 2000[136]	Not described	Not stated	Not possible	Not described	No losses to follow up after initial 23222	No but protocol not checked	No	Unclear
Li et al. 2017[79]	Random number table	Not stated	Not possible	Not described but PROM	No losses to follow up	No but protocol not checked	Groups similar at baseline	Low
Liebs et al. 2012[81]	Computer generated	Allocation concealed	Not possible	No but PROM	Low losses to follow up (<20% if deaths and other explained reasons not counted)	No but protocol not checked	No	Low
Mahomed et al. 2008[82]	Block randomisation	Not stated	Not possible	PROM	No loss	No but protocol not checked	ITT gave similar results to analysis according to actual discharge destination (20 inpatient group received home based)	Low
Rahmann et al. 2009[135]	Not described	Sealed numbered envelopes	Not possible	Assessor blind to intervention. Patient reported outcome	Low losses to follow up	No but protocol not checked	TKR patients more likely to receive ward-based control intervention. THR and TKR analysed together	Unclear
Wang et al. 2014[80]	Computer generated	Surgeons did not participate	Surgery was performed by the	Postoperative evaluation was	No loss to follow up	None apparent	No baseline differences	Low

		in pre-operative grouping	physicians who did not participate in the preoperative grouping and postoperative evaluation	conducted by the physicians who were unaware of the grouping.		but protocol not checked		
Wound management								
Kong et al. 2014[71]	Not described	Not described	Placebo used	Patient outcome	Low loss to follow up	None apparent but protocol not checked	Similar at baseline	Low
Anabolic steroids								
Hohmann et al. 2010[83]	Internet based	Not reported	Placebo trial	Double-blind design minimized systemic error and eliminated observer and experimenter's bias	0 loss to follow up	None apparent	None apparent but small study	Low
Guided imagery								
Jacobson et al. 2016[137]	Permuted blocks	Opaque CD holders	Personnel yes, participants no	Yes	12; 10 of patients receiving treatment	None apparent but protocol not checked	No	High

Beswick AD, *et al. BMJ Open* 2019; 9:e028093. doi: 10.1136/bmjopen-2018-028093

References to all RCTs of peri-operative interventions with long-term pain or score follow up, irrespective of risk of bias assessment (numbering consistent with main article)

34. Widmer BJ, Scholes CJ, Pattullo GG, et al. Is femoral nerve block necessary during total knee arthroplasty?: a randomized controlled trial. J Arthroplasty 2012;27:1800-5.
35. Gao WL, Li H, Liu BQ, et al. Analgesic effect of femoral and sciatic nerve block under multimodal analgesia in total knee arthroplasty. Chinese Journal of Tissue Engineering Research 2017;21:2966-72.
36. Nader A, Kendall MC, Wixson RL, et al. A randomized trial of epidural analgesia followed by continuous femoral analgesia compared with oral opioid analgesia on short- and long-term functional recovery after total knee replacement. Pain Med 2012;13:937-47.
37. Wu JWS, Wong YC. Elective unilateral total knee replacement using continuous femoral nerve blockade versus conventional patient-controlled analgesia: perioperative patient management based on a multidisciplinary pathway. Hong Kong Med J 2014;20:45-51.
38. Peng L, Ren L, Qin P, et al. Continuous femoral nerve block versus intravenous patient controlled analgesia for knee mobility and long-term pain in patients receiving total knee replacement: a randomized controlled trial. Evid Based Complement Alternat Med 2014;2014:569107.
39. Fan L, Yu X, Zan P, et al. Comparison of local infiltration analgesia with femoral nerve block for total knee arthroplasty: A prospective, randomized clinical trial. J Arthroplasty 2016;31:1361-5.
40. Reinhardt KR, Duggal S, Umunna BP, et al. Intraarticular analgesia versus epidural plus femoral nerve block after TKA: A randomized, double-blind trial. Clin Orthop Relat Res 2014;472:1400-8.
41. Albrecht E, Morfey D, Chan V, et al. Single-injection or continuous femoral nerve block for total knee arthroplasty? Clin Orthop Relat Res 2014;472:1384-93.
42. Choy WS, Lee SK, Kim KJ, et al. Two continuous femoral nerve block strategies after TKA. Knee Surg Sports Traumatol Arthrosc 2011;19:1901-8.
43. Macrinici GI, Murphy C, Christman L, et al. Prospective, double-blind, randomized study to evaluate single-injection adductor canal nerve block versus femoral nerve block: postoperative functional outcomes after total knee arthroplasty. Reg Anesth Pain Med 2017;42:10-6.
44. Wegener JT, van Ooij B, van Dijk CN, et al. Long-term pain and functional disability after total knee arthroplasty with and without single-injection or continuous sciatic nerve block in addition to continuous femoral nerve block: a prospective, 1-year follow-up of a randomized controlled trial. Reg Anesth Pain Med 2013;38:58-63.
45. Wylde V, Lenguerrand E, Gooberman-Hill R, et al. Effect of local anaesthetic infiltration on chronic postsurgical pain after total hip and knee replacement: the APEX randomised controlled trials. Pain 2015;156:1161-70.
47. Niemeläinen M, Kalliovalkama J, Aho A, et al. Single periarticular local infiltration analgesia reduces opiate consumption until 48 hours after total knee arthroplasty. A randomized placebo-controlled trial involving 56 patients. Acta Orthop 2014;85:614-9.
49. Motififard M, Omidian A, Badiei S. Pre-emptive injection of peri-articular-multimodal drug for post-operative pain management in total knee arthroplasty: a double-blind randomized clinical trial. Int Orthop 2017;41:939-47.
51. Williams D, Petruccelli D, Paul J, et al. Continuous infusion of bupivacaine following total knee arthroplasty: a randomized control trial pilot study. J Arthroplasty 2013;28:479-84.

Beswick AD, *et al. BMJ Open* 2019; 9:e028093. doi: 10.1136/bmjopen-2018-028093

52. McDonald DA, Deakin AH, Ellis BM, et al. The technique of delivery of peri-operative analgesia does not affect the rehabilitation or outcomes following total knee arthroplasty. Bone Joint J 2016;98-B:1189-96.
53. Seah VWT, Chin PL, Chia SL, et al., double-blind, randomised controlled trial. Singapore Med J 2011;52:19-23.
54. Meunier A, Lisander B, Good L. Effects of celecoxib on blood loss, pain, and recovery of function after total knee replacement: a randomized placebo-controlled trial. Acta Orthop 2007;78:661-7.
55. Aveline C, Roux AL, Hetet HL, et al. Pain and recovery after total knee arthroplasty: a 12-month follow-up after a prospective randomized study evaluating Nefopam and Ketamine for early rehabilitation. Clin J Pain 2014;30:749-54.
56. Buvanendran A, Kroin JS, Della Valle CJ, et al. Perioperative oral pregabalin reduces chronic pain after total knee arthroplasty: a prospective, randomized, controlled trial. Anesth Analg 2010;110:199-207.
58. Ejaz A, Laursen AC, Kappel A, et al. Faster recovery without the use of a tourniquet in total knee arthroplasty. Acta Orthop 2014;85:422-6.
59. Liu D, Graham D, Gillies K, et al. Effects of tourniquet use on quadriceps function and pain in total knee arthroplasty. Knee Surg Relat Res 2014;26:207-13.
60. Huang Z, Xie X, Li L, et al. Intravenous and topical tranexamic acid alone are superior to tourniquet use for primary total knee arthroplasty: A prospective, randomized controlled trial. J Bone Joint Surg (Am) 2017;99:2053-61.
61. Mittal R, Ko V, Adie S, et al. Tourniquet application only during cement fixation in total knee arthroplasty: a double-blind, randomized controlled trial. ANZ J Surg 2012;82:428-33.
62. Zhang Y, Li D, Liu P, et al. Effects of different methods of using pneumatic tourniquet in patients undergoing total knee arthroplasty: a randomized control trial. Ir Jr Med Sci 2017;186:953-9.
63. Kim TK, Chang CB, Kang YG, et al. Clinical value of tranexamic acid in unilateral and simultaneous bilateral TKAs under a contemporary blood-saving protocol: a randomized controlled trial. Knee Surg Sports Traumatol Arthrosc 2014;22:1870-8.
64. Sa-Ngasoongsong P, Channoom T, Kawinwonggowit V, et al. Postoperative blood loss reduction in computer-assisted surgery total knee replacement by low dose intra-articular tranexamic acid injection together with 2-hour clamp drain: a prospective triple-blinded randomized controlled trial. Orthop Rev 2011;3:e12.
65. Sa-Ngasoongsong P, Wongsak S, Chanplakorn P, et al. Efficacy of low-dose intra-articular tranexamic acid in total knee replacement; a prospective triple-blinded randomized controlled trial. BMC Musculoskelet Disord 2013;14:340.
67. Hourlier H, Reina N, Fennema P. Single dose intravenous tranexamic acid as effective as continuous infusion in primary total knee arthroplasty: a randomised clinical trial. Arch Orthop Trauma Surg 2015;135:465-71.
68. Kusuma SK, Sheridan KC, Wasielewski RC. Use of bovine thrombin to reduce blood loss in primary total knee arthroplasty: a controlled randomized trial. J Arthroplasty 2013;28:1278-81.
69. Napier RJ, Bennett D, McConway J, et al. The influence of immediate knee flexion on blood loss and other parameters following total knee replacement. Bone Joint J 2014;96-B:201-9.
70. Brock T, Sprowson A, Muller S, et al. STICKS study - Short-sTretch Inelastic Compression bandage in Knee Swelling following total knee arthroplasty - a feasibility study. Trials 2017;18:6.
71. Kong CG, Kim GH, Kim DW, et al. The effect of topical scar treatment on postoperative scar pain and pruritus after total knee arthroplasty. Arch Orthop Trauma Surg 2014;134:555-9.

72. Ledin H, Good L, Aspenberg P. Denosumab reduces early migration in total knee replacement. Acta Orthop 2017;88:255-8.
73. Ersözlü S, Sahin O, Ozgür A, et al. The effects of two different continuous passive motion protocols on knee range of motion after total knee arthroplasty: a prospective analysis. Acta Orthop Traumato 2009;43:412-8.
74. Bennett LA, Brearley SC, Hart JAL, et al. A comparison of 2 continuous passive motion protocols after total knee arthroplasty: a controlled and randomized study. J Arthroplasty 2005;20:225-33.
75. Avramidis K, Karachalios T, Popotonasios K, et al. Does electric stimulation of the vastus medialis muscle influence rehabilitation after total knee replacement? Orthopedics 2011;34:175.
77. Moretti B, Notarnicola A, Moretti L, et al. I-ONE therapy in patients undergoing total knee arthroplasty: a prospective, randomized and controlled study. BMC Musculoskelet Disord 2012;13:88.
79. Li L, Wang Z, Yin MH, et al. Effect of early gait training on the functional rehabilitation after total knee arthroplasty. Chinese Journal of Tissue Engineering Research 2017;21:4288-93.
80. Wang S, Xia J, Wei Y, et al. Effect of the knee position during wound closure after total knee arthroplasty on early knee function recovery. J Orthop Surg Res 2014;9:79.
81. Liebs T, Herzberg W, Rüther W, et al. Multicenter randomized controlled trial comparing early versus late aquatic therapy after total hip or knee arthroplasty. Arch Phys Med Rehabil 2012;93:192-9.
82. Mahomed NN, Davis AM, Hawker G, et al. Inpatient compared with home-based rehabilitation following primary unilateral total hip or knee replacement: a randomized controlled trial. J Bone Joint Surg (Am) 2008;90:1673-80.
83. Hohmann E, Tetsworth K, Hohmann S, et al. Anabolic steroids after total knee arthroplasty. A double blinded prospective pilot study. J Orthop Surg Res 2010;5:93.
105. Bergeron SG, Kardash KJ, Huk OL, et al. Functional outcome of femoral versus obturator nerve block after total knee arthroplasty. *Clin Orthop Relat R* 2009;467:1458-62.
106. Shum CF, Lo NN, Yeo SJ, et al. Continuous femoral nerve block in total knee arthroplasty: immediate and two-year outcomes. *J Arthroplasty* 2009;24:204-9.
107. Perrin SB, Purcell AN. Intraoperative ketamine may influence persistent pain following knee arthroplasty under combined general and spinal anaesthesia: a pilot study. *Anaesth Intensive Care* 2009;37:248-53.
108. Ilfeld BM, Meyer RS, Le LT, et al. Health-related quality of life after tricompartment knee arthroplasty with and without an extended-duration continuous femoral nerve block: a prospective, 1-year follow-up of a randomized, triple-masked, placebo-controlled study. *Anesth Analg* 2009;108:1320-5.
109. Ilfeld BM, Shuster JJ, Theriaque DW, et al. Long-term pain, stiffness, and functional disability after total knee arthroplasty with and without an extended ambulatory continuous femoral nerve block: A prospective, 1-year follow-up of a multicenter, randomized, triple-masked, placebo-controlled trial. *Reg Anesth Pain Med* 2011;36:116-20.
110. Morin AM, Kratz CD, Eberhart LH, et al. Postoperative analgesia and functional recovery after total-knee replacement: comparison of a continuous posterior lumbar plexus (psoas compartment) block, a continuous femoral nerve block, and the combination of a continuous femoral and sciatic nerve block. *Reg Anesth Pain Med* 2005;30:434-45.
111. Davidson E, Machi A, Sztain J, et al. Adductor canal versus femoral continuous peripheral nerve blocks for knee arthroplasty: A one-year follow-up pilot study of two randomized, controlled clinical trials. *Reg Anesth Pain Med* 2016;41.
112. Wang F, Zhou Y, Sun J, et al. Influences of continuous femoral nerve block on knee function and quality of life in patients following total knee arthroplasty. *Int J Clin Exp Med* 2015;8:19120-5.

113. Anastase DM, Winckelmann J, Geiger P. Effects of regional anaesthesia techniques on patients' satisfaction after total knee arthroplasty. *Jurnalul Roman de Anestezie Terapie Intensiva* 2014;21:35-43.
114. Yue D-b, Wang B-l, Liu K-p, et al. Efficacy of multimodal cocktail periarticular injection with or without steroid in total knee arthroplasty. *Chinese Med J* 2013;126:3851-5.
115. Spreng UJ, Andersson E, Dahl V. Long-term outcome after total knee arthroplasty local infiltration analgesia (LIA) vs. Epidural analgesia. *Br J Anaesth* 2012;108:ii419.
116. Spreng UJ, Dahl V, Hjall A, et al. High-volume local infiltration analgesia combined with intravenous or local ketorolac+morphine compared with epidural analgesia after total knee arthroplasty. *Br J Anaesth* 2010;105:675-82.
117. Foadi N, Karst M, Frese-Gaul A, et al. The improved quality of postoperative analgesia after intrathecal morphine does not result in improved recovery and quality of life in the first 6 months after orthopedic surgery: a randomized controlled pilot study. *J Pain Res* 2017;10:1059-69.
118. Mayoral O, Salvat I, Martin MT, et al. Efficacy of myofascial trigger point dry needling in the prevention of pain after total knee arthroplasty: A randomized, double-blinded, placebo-controlled trial. *Evid Based Complement Alternat Med* 2013;2013:Article ID 694941.
119. Abdel-Salam A, Eyres KS. Effects of tourniquet during total knee arthroplasty. A prospective randomised study. *J Bone Joint Surg (Br)* 1995;77:250-3.
120. Sükür E, Öztürkmen Y, Akman Y, et al. The effect of tourniquet and knee position during wound closure after total knee arthroplasty on early recovery of range of motion: a prospective, randomized study. *Arch Orthop Trauma Surg* 2016;136:1773-80.
121. Zhang Q, Dong J, Gong K, et al. Effects of tourniquet use on perioperative outcome in total knee arthroplasty. *Chinese Journal of Reparative and Reconstructive Surgery* 2016;30:421-5.
122. Thomas D, Wareham K, Cohen D, et al. Autologous blood transfusion in total knee replacement surgery. *Br J Anaesth* 2001;86:669-73.
123. Aggarwal AK, Shashikanth VS, Marwaha N. Platelet-rich plasma prevents blood loss and pain and enhances early functional outcome after total knee arthroplasty: a prospective randomised controlled study. *Int Orthop* 2014;38:387-95.
124. Wang X. Impact of local compression cryotherapy combined with continuous passive motion on the early functional recovery after total knee arthroplasty. *Chinese Journal of Tissue Engineering Research* 2017;21:998-1003.
125. Leach W, Reid J, Murphy F. Continuous passive motion following total knee replacement: a prospective randomized trial with follow-up to 1 year. *Knee Surg Sports Traumatol Arthrosc* 2006;14:922-6.
126. Sahin E, Akalin E, Bircan C, et al. The effects of continuous passive motion on outcome in total knee arthroplasty. *Journal of Rheumatology and Medical Rehabilitation* 2006;17:85-90.
127. Pope RO, Corcoran S, McCaul K, et al. Continuous passive motion after primary total knee arthroplasty. Does it offer any benefits? *J Bone Joint Surg (Br)* 1997;79:914-7.
128. Beaupre LA, Davies DM, Jones CA, et al. Exercise combined with continuous passive motion or slider board therapy compared with exercise only: a randomized controlled trial of patients following total knee arthroplasty. *Physical therapy* 2001;81:1029-37.
129. Kumar PJ, McPherson EJ, Dorr LD, et al. Rehabilitation after total knee arthroplasty: a comparison of 2 rehabilitation techniques. *Clin Orthop Relat R* 1996:93-101.
130. Worland RL, Arredondo J, Angles F, et al. Home continuous passive motion machine versus professional physical therapy following total knee replacement. *J Arthroplasty* 1998;13:784-7.
131. MacDonald SJ, Bourne RB, Rorabeck CH, et al. Prospective randomized clinical trial of continuous passive motion after total knee arthroplasty. *Clin Orthop Relat Res* 2000:30-5.

132. Stevens-Lapsley JE, Balter JE, Wolfe P, et al. Early neuromuscular electrical stimulation to improve quadriceps muscle strength after total knee arthroplasty: a randomized controlled trial. *Physical therapy* 2012;92:210-26.
133. Levine M, McElroy K, Stakich V, et al. Comparing conventional physical therapy rehabilitation with neuromuscular electrical stimulation after TKA. *Orthopedics* 2013;36:e319-24.
134. Adravanti P, Nicoletti S, Setti S, et al. Effect of pulsed electromagnetic field therapy in patients undergoing total knee arthroplasty: a randomised controlled trial. *Int Orthop* 2014;38:397-403.
135. Rahmann AE, Brauer SG, Nitz JC. A specific inpatient aquatic physiotherapy program improves strength after total hip or knee replacement surgery: a randomized controlled trial. *Arch Phys Med Rehabil* 2009;90:745-55.
136. Hill SP, Flynn J, Crawford EJP. Early discharge following total knee replacement -- a trial of patient satisfaction and outcomes using an orthopaedic outreach team. *J Orthop Nurs* 2000;4:121-6.
137. Jacobson AF, Umberger WA, Palmieri PA, et al. Guided imagery for total knee replacement: A randomized, placebo-controlled pilot study. *J Altern Complement Med* 2016;22:563-75.
