# Supplementary material for: Are perioperative interventions effective in preventing chronic pain after primary total knee replacement? A systematic review
Source: BMJ Open. 2019 Sep 6;9(9):e028093. doi: 10.1136/bmjopen-2018-028093 (PMC6731899; doi:10.1136/bmjopen-2018-028093)
Supplement: Author's manuscript [file bmjopen-2018-028093.draft_revisions.pdf]

BMJ Open

BMJ Open is committed to open peer review. As part of this commitment we make the peer review history of every article we publish publicly available.

When an article is published we post the peer reviewers' comments and the authors' responses online. We also post the versions of the paper that were used during peer review. These are the versions that the peer review comments apply to.

The versions of the paper that follow are the versions that were submitted during the peer review process. They are not the versions of record or the final published versions. They should not be cited or distributed as the published version of this manuscript.

BMJ Open is an open access journal and the full, final, typeset and author-corrected version of record of the manuscript is available on our site with no access controls, subscription charges or pay-per-view fees (http://bmjopen.bmj.com).

If you have any questions on BMJ Open's open peer review process please email info.bmjopen@bmj.com

BMJ Open

Are peri-operative interventions effective in preventing chronic pain after primary total knee replacement? A systematic review

Journal:	*BMJ Open*
Manuscript ID	bmjopen-2018-028093
Article Type:	Research
Date Submitted by the Author:	22-Nov-2018
Complete List of Authors:	Beswick, Andrew; University of Bristol, School of Clinical Sciences<br>Dennis, Jane; University of Bristol, Musculoskeletal Research Unit<br>Gooberman-Hill, Rachael; University of Bristol, Bristol Medical School<br>Blom, AW; University of Bristol,<br>Wylde, Vikki; University of Bristol, Musculoskeletal Research Unit
Keywords:	Knee < ORTHOPAEDIC & TRAUMA SURGERY, Pain management < ANAESTHETICS, REHABILITATION MEDICINE

SCHOLARONE™
Manuscripts

Are peri-operative interventions effective in preventing chronic pain after primary total knee replacement? A systematic review

Andrew D Beswick[1], Jane Dennis[1], Rachael Gooberman-Hill[1,2], Ashley W Blom[1,2], Vikki Wylde[1,2]

[1] Musculoskeletal Research Unit, Translational Health Sciences, Bristol Medical School, University of Bristol

[2] National Institute for Health Research Bristol Biomedical Research Centre, University Hospitals Bristol NHS Foundation Trust and University of Bristol

Corresponding author:

Andrew David Beswick
Musculoskeletal Research Unit
Translational Health Sciences
Bristol Medical School
University of Bristol
Learning and Research Building, Level 1
Southmead Hospital
Bristol
BS10 5NB

email: andy.beswick@bristol.ac.uk
Telephone: 0117 414 7870

Word count: 4000 words

For peer review only - http://bmjopen.bmj.com/site/about/guidelines.xhtml

ABSTRACT

Objectives

For many people with advanced osteoarthritis, total knee replacement (TKR) is an effective treatment for relief of pain and improvement of function. Features of peri-operative care may be associated with chronic pain six months or longer after surgery. Effects may be direct, e.g. through nerve damage or complications, or indirect by limiting mobilisation and rehabilitation. The objective of this systematic review is to evaluate whether non-surgical peri-operative interventions prevent long-term pain after TKR.

Methods

We conducted a systematic review of peri-operative interventions for adults with osteoarthritis receiving primary TKR evaluated in a randomised controlled trial (RCT). We searched major bibliographic databases up to February 2018. After screening, two reviewers evaluated articles. Studies at low risk of bias according to the Cochrane tool were included.

Interventions

Peri-operative non-surgical interventions; control receiving no intervention or alternative.

Primary and secondary outcome measures

Pain or score with pain component assessed at six months or longer post-operative

Results

44 RCTs at low risk of bias assessed long-term pain. Intervention heterogeneity precluded meta-analysis and definitive statements on effectiveness. There was encouragement for further research into local infiltration analgesia, ketamine infusion, pregabalin, and electric muscle stimulation. In the studies we identified, tranexamic acid to prevent blood loss was not associated with long-term pain. Many extensively researched interventions including venous thromboembolism prevention have not been evaluated in relation to long-term pain.

Interpretation

Our review summarises evidence on peri-operative treatments for the prevention of long-term pain after TKR and highlights aspects of care for further evaluation in well-conducted RCTs. Long-term consequences of many widely researched treatments have not been reported.

STRENGTHS AND LIMITATIONS

For peer review only - http://bmjopen.bmj.com/site/about/guidelines.xhtml

For peer review only

- For the first time, this systematic review brings together contemporary evidence on aspects of peri-operative care for people with total knee replacement and their effects on long-term pain.
- Only studies assessed to be at low risk of bias were included in the narrative synthesis.
- Intervention and outcome heterogeneity precluded meta-analysis.

KEYWORDS

Total knee replacement; Systematic review; Randomised controlled trial; Peri-operative care; Long-term pain

For peer review only

BACKGROUND

In the US about 13% of men and 19% of women will be diagnosed with knee osteoarthritis and over half will receive a total knee replacement (TKR)[1]. For people with advanced osteoarthritis unresponsive to pharmacological or conservative treatments, TKR aims to relieve pain and improve function. In the UK nearly 100,000 primary TKRs were performed in 2017[2,3] and in the USA in 2010, an estimated 4.7 million people were living with a TKR[4]. Despite good outcomes for many, some people report long-term pain and are disappointed with their surgery[5,6]. After TKR, pain levels plateau from about 6 months[7,8] after which persistent pain is considered “chronic”[9] and is reported by 10-34% of patients[10].

In the peri-operative period from hospital admission to the early stages of recovery, care focuses on acute pain management, prevention of adverse events, facilitation of early mobilisation and timely discharge. However, for people with osteoarthritis the key aim of TKR is the achievement of a long-term painless and well-functioning knee with no adverse events. All aspects of peri-operative care should work together to achieve this.

Peri-operative risk factors suggest that appropriate interventions may reduce long-term pain. For example, acute post-operative pain, which may be a direct consequence of the operation, anaesthetic protocol and subsequent analgesia, or related to particular aspects of care, is an acknowledged risk factor for chronic post-surgical pain[11]. Any treatment in the peri-operative period could potentially affect patient recovery and chronic pain, either directly or indirectly. Direct benefits may be through prevention of nerve damage[12], post-thrombotic syndrome[13], reperfusion injury[14] and articular bleeding[15]. Patients with depression and catastrophising have poor pain outcomes[16,17]. For other treatments, pathways leading to long-term pain may be indirect consequences of delayed mobilisation, rehabilitation and recovery.

Our systematic review of randomised controlled trials (RCTs) aims to evaluate the effectiveness of treatments in the peri-operative period in preventing long-term pain after TKR. By focusing on studies with low risk of bias we aim to identify interventions with robust evidence of long-term effectiveness and identify gaps in the research base.

METHODS

The systematic review protocol was registered (PROSPERO CRD42017041382) and PRISMA reporting guidelines used[18]. A checklist is included as Supplementary material.

Patient and public involvement

For peer review only - http://bmjopen.bmj.com/site/about/guidelines.xhtml

As part of the STAR programme of research (NIHR RP-PG-0613-20001), this review benefited from extensive patient and public involvement. Advice was sought from patients and stakeholders at a group discussion in March 2016 with decisions made on inclusion criteria and outcomes. Our patient advisory group comprises five patients with experience of long-term pain after TKR, supported by a dedicated co-ordinator. This group will advise on dissemination of the study results to a general audience including plain language summaries.

Eligibility criteria

Participants: adults receiving unilateral primary TKR, predominantly for osteoarthritis.

Interventions: peri-operative interventions (pharmacological or non-pharmacological) were included. "Peri-operative" reflects the time from hospital admission to early stages of recovery. Interventions relating to implant designs and surgical procedures were excluded.

Comparator: usual care, placebo or alternative intervention.

Outcomes: in preference, patient-reported joint-specific pain intensity measured by tools such as the Western Ontario and McMaster Universities Osteoarthritis Index (WOMAC) or Oxford Knee Score (OKS). If joint-specific measures were unavailable, pain dimensions from quality of life measures were used or pain rated on a visual analogue scale (VAS) or numerical rating scale (NRS). We also considered composite patient-reported outcome measures and surgeon scores which included a pain intensity component, such as the American Knee Society Score (KSS) and Hospital for Special Surgery (HSS) score. The occurrence of adverse events was summarised.

Setting: RCTs with follow up at ≥6 months after surgery and a pain outcome or score including pain. Authors of studies were contacted regarding incomplete pain outcome data.

Database searches

We established an Endnote database of all RCTs in TKR. On 14th February 2018, a final search was conducted in: *The Cochrane Library*; MEDLINE, Embase and PsycINFO on Ovid; and CINAHL on EBSCOhost. The MEDLINE search strategy is included as supplementary material. Citations of key articles were tracked in Web of Science. No language restrictions were applied, and translations made. Studies reported as abstracts or unobtainable using inter-library loans and author contact were excluded.

Screening and data extraction

For peer review only - http://bmjopen.bmj.com/site/about/guidelines.xhtml

For peer review only

We imported records into Endnote X7 (Thomson Reuters). An initial screen by one reviewer excluded clearly irrelevant articles. Subsequently, abstracts and full articles were screened independently by two reviewers and reasons for exclusion recorded.

Data were extracted onto piloted forms and an Excel spreadsheet by one reviewer, specifically: country; dates; participants (indication, age, sex); inclusion and exclusion criteria; intervention and control content; setting, timing, duration and intensity of intervention; follow up intervals; losses to follow up; pain outcome data; and serious adverse events. Data was checked against source material by a second reviewer.

Authors were contacted for missing data, and data provided for previous reviews was used[10,19].

Quality assessment

Potential sources of bias were assessed by two experienced reviewers using the Cochrane risk of bias tool[20], specifically: the randomisation process; deviations from intended interventions; missing outcome data, measurement of the outcome; and selection of the reported result. Studies with serious concerns relating to risk of bias were considered high risk and those with limited reporting unclear risk. Studies with high or unclear risk of bias were excluded from the narrative synthesis but are included in supplementary summary tables with reasons for exclusion.

Data analysis

Insufficient studies with similar interventions and outcomes were identified for meta-analysis, and a narrative synthesis is presented. Results reported with p-values ≤0.001 were considered “strong” evidence of effectiveness[21], p-values 0.001-0.05 “some” evidence, and p-values 0.05-0.1 “weak” evidence. When authors reported results “statistically significant” with no p-value, this was noted. Where possible, effect sizes were compared with published minimal clinically important differences (MCID). Concerns relating to adverse events were summarised.

RESULTS

Figure 1 shows review progress and reasons for exclusion. Peri-operative interventions with follow up of ≥six months were evaluated in 130 RCTs of which 76 reported a pain outcome or score with a pain component. Detailed intervention and study characteristics and risk of bias assessments are provided as supplementary material.

Details of 44 studies assessed to be at low risk of bias are summarised in Table 1.

For peer review only - http://bmjopen.bmj.com/site/about/guidelines.xhtml

For peer review only

Table 1. Perioperative interventions with follow up for pain or score at 6 months or later and assessed to be at low risk of bias

Study	Treatment common to randomised groups	Intervention	Number patients	Follow up<br><br>Group difference
Pain management: nerve blocks				
Albrecht et al. 2014[29]<br><br>Canada, 2009-2011,<br><br>1 hospital	SNB	1. FNB continuous high<br><br>2. FNB continuous low<br><br>3. FNB single	99	1 year<br><br>WOMAC score: no difference (p=0.68)
Choy et al. 2011[30]<br><br>Korea, 2006-2007,<br><br>1 surgeon	PCA	1. FNB continuous long<br><br>2. FNB continuous short	61	1 year<br><br>WOMAC pain: no difference (p=0.2)
Fan et al. 2016[27]<br><br>China, 2012-2014,<br><br>2 surgeons	PCA	1. FNB single<br><br>2. LIA	157	1 year<br><br>KSS: no difference (p=0.51)
Gao et al. 2017[23]<br><br>China, 2014-2015,<br><br>1 centre	LIA	1. General anaesthesia<br><br>2. FNB single<br><br>3. FNB/ SNB single	150	6 months<br><br>HSS score: no significant difference
Macrinici et al. 2017[31]<br><br>USA, Before 2017<br><br>1 centre	LIA	1. ACB single<br><br>2. FNB single	98	6 months<br><br>VAS pain: no difference
Nader et al. 2012[24]<br><br>USA, 2007-2008,<br><br>1 surgeon	PCA	1. FNB continuous<br><br>2. Oral opioid	62	1 year<br><br>NRS pain stair: some evidence favouring opioid (p=0.01) but not consistent. Overall NRS pain: no difference (p=1.0)<br><br>VTE: concern opioid
Peng et al. 2014[26]<br><br>China, Before 2014,		1. FNB continuous<br><br>2. PCA	280	6 months and 1 year<br><br>NRS pain: some evidence favouring FNB at 6 months

For peer review only - http://bmjopen.bmj.com/site/about/guidelines.xhtml

1 centre				(p=0.021); no difference at 1 year (p=0.273)
Reinhardt et al. 2014[28]<br><br>USA, 2010-2012,<br><br>2 surgeons		1. FNB single/ epidural<br><br>2. LIA 48 hours	94	1 year<br><br>VAS pain: no difference
Wegener et al. 2013[32]<br><br>The Netherlands, 2008-2010,<br><br>1 centre	FNB	1. SNB single<br><br>2. SNB continuous<br><br>3. PCA	89	1 year<br><br>WOMAC pain: no difference (p=0.81)
Widmer et al. 2012[22]<br><br>Australia, before 2012,<br><br>2 surgeons	LIA, PCA	1. FNB single<br>2. Control no FNB	55	1 year<br><br>WOMAC pain: no difference (p=0.74)
Wu and Wong 2014[25]<br><br>China, 2009-2011,<br><br>1 centre		1. FNB continuous<br><br>2. PCA	60	6 months<br><br>KSS: no difference (p=0.513)
Pain management: LIA				
McDonald et al. 2016[40]<br><br>UK, 2010-2011<br><br>1 hospital		1. LIA<br><br>2. PCA	222	1 year<br><br>OKS: no difference (p=0.915)
Motififard et al. 2017[37]<br><br>Iran, 2014-2015<br><br>1 hospital		1. LIA pre-emptive injection<br><br>2. Control saline with epinephrine	120	6 months<br><br>KSS: weak evidence favouring LIA (p=0.07). Difference between groups (14.2/200) less than MCID (12.3/200).
Niemeläinen et al. 2014[35]<br><br>Finland, 2011-2012<br><br>1 hospital	PCA	1. LIA<br><br>2. Control saline	56	1 year<br><br>OKS: weak evidence from means and confidence intervals favouring LIA. Difference (2.7/48) less than MCID (4.0/48)
Seah et al. 2011[41]<br><br>Singapore, 2004-2005	PCA	1. LIA with corticosteroid<br><br>2. LIA no corticosteroid	100	6 months and 2 years<br><br>OKS: no difference

For peer review only

For peer review only - http://bmjopen.bmj.com/site/about/guidelines.xhtml

1 hospital				
Williams et al. 2013[39]<br>Canada, Before 2013<br>2 surgeons	LIA, PCA	1. LIA 48 hours<br>2. Control saline	51	6 months and 1 year<br>VAS pain: no difference (6 months p=0.836, 1 year p=0.767)
Wylde et al. 2015[33]<br>UK, 2009-2012<br>1 centre	FNB, PCA	1. LIA<br>2. Control no LIA	280	6 months and 1 year<br>WOMAC pain: weak evidence favouring LIA at 6 months p=0.063; 1 year p=0.107. Mean difference at 1 year (3.8/100) lower than MCID (8–9/100)
Pain management: Celecoxib				
Meunier et al. 2007[42]<br>Sweden, 2004-2005<br>1 centre	PCA	1. Celecoxib<br>2. Control placebo	44	1 year<br>KOOS/VAS pain: no statistical difference
Pain management: Ketamine/ Nefopam				
Aveline et al. 2014[43]<br>France, 2005<br>1 centre	PCA	1. Ketamine infusion<br>2. Nefopam infusion<br>3. Control saline	75	6 months and 1 year<br>DN4/VAS pain: some evidence favouring ketamine (for DN4 p=0.02). Few patients had neuropathic pain at 12 months.
Pain management: Pregabalin				
Buvanendran et al. 2010[44]<br>USA, 2006-2007<br>Single centre	LIA, PCA	1. Pregabalin<br>2. Control placebo	240	6 months<br>S-LANSS pain: no neuropathic pain reported in pregabalin group compared with 5.2% of patients in control group (p=0.014)
Tourniquet				
Ejaz et al. 2014[45]<br>Denmark, 2011-2012<br>1 centre	Tranexamic acid	1. Tourniquet<br>2. Tourniquet not inflated	64	6 months and 1 year<br>KOOS pain: no significant difference

For peer review only - http://bmjopen.bmj.com/site/about/guidelines.xhtml

Huang et al. 2017[47]<br><br>China, 2015<br><br>1 centre	Tranexamic acid	1. Tourniquet<br><br>2. No tourniquet	100	6 months<br><br>VAS pain: no difference (p=0.728)<br><br>Wound: concern tourniquet
Liu et al. 2014[46]<br><br>Australia, Before 2014<br><br>1 surgeon		1. Tourniquet<br><br>2. Tourniquet not inflated	20	6 months and 1 year<br><br>OKS: no significant difference<br><br>Transfusion: concern tourniquet
Mittal et al. 2012[48]<br><br>Australia, 2008-2010<br><br>1 centre		1. Tourniquet short duration<br><br>2. Tourniquet long duration	65	1 year<br><br>OKS: weak evidence from means and Cis on graph favouring long duration at 1 year. Mean difference (5) greater than MCID (4)<br><br>Transfusions/ adverse events: concern short
Zhang et al. 2017[49]<br><br>China, 2008-2011<br><br>1 surgeon		1. Tourniquet for entire operation<br><br>2. Tourniquet removed before wound closure<br><br>3. Tourniquet from first bone osteotomy until closure	150	6 months<br><br>HSS score: no difference (p=0.839)<br><br>Transfusions: concern late tourniquet start in groups 1 and 2
Compression bandage				
Brock et al. 2017[57]<br><br>UK, 2013-2014<br><br>1 hospital	Hydrocolloid dressing	1. Compression bandage<br><br>2. Standard crepe bandage	49	6 months<br><br>OKS: no difference (p=0.58)
Blood conservation				
Hourlier et al. 2015[54]<br><br>France, 2009-2010<br><br>1 hospital	Drain, tourniquet, electrocautery	1. Continuous infusion tranexamic acid<br><br>2. Control saline	107	6 months<br><br>KSS: no difference (p=0.90)

For peer review only - http://bmjopen.bmj.com/site/about/guidelines.xhtml

Huang et al. 2017[47]<br><br>China, 2015<br><br>1 centre	Tourniquet	1. Intravenous and topical tranexamic acid<br><br>2. No tranexamic acid	100	6 months<br><br>VAS pain: no difference (p=0.728)<br><br>HSS score: strong evidence favouring tranexamic acid (p<0.001). Mean difference (1.4/100) lower than MCID (8.3/100)<br><br>Blood loss: control concern
Kim et al. 2014[51]<br><br>Korea, 2009-2011<br><br>1 hospital	Tourniquet, drain, compressive dressing	1. Tranexamic acid<br><br>2. No tranexamic acid	180	1 year<br><br>WOMAC pain: no significant difference<br><br>Transfusion: control concern
Kusuma et al. 2013[55]<br><br>USA, Before 2013<br><br>1 hospital	Tourniquet, Esmarch bandage, electrocautery	1. Thrombin infusion<br><br>2. No thrombin infusion	80	6 months, 1 and 2 years<br><br>KSS: no difference (p=0.45)
Napier et al. 2014[56]<br><br>UK, 2003-2004<br><br>1 hospital		1. Passive flexion<br><br>2. Passive extension	180	1 year<br><br>OKS: no difference (p=0.27)<br><br>Transfusion: extension concern
Sa-Ngasoongsong et al. 2011[50]<br><br>Thailand, 2008-2009<br><br>1 hospital	Drain and compressive dressing	1. Tranexamic acid<br><br>2. Control saline	48	6 months<br><br>WOMAC score: no difference (p=0.282)<br><br>Transfusion: control concern
Sa-Ngasoongsong et al. 2013[52]<br><br>Thailand, 2010-2011<br><br>1 hospital	Drain and compressive dressing	1. Tranexamic acid 500mg<br><br>2. Tranexamic acid 250mg<br><br>3. Control saline	135	1 year<br><br>WOMAC score: no difference (p=0.42)<br><br>Transfusions: control and 250mg group concerns
Denusomab				
Ledin et al. 2017[59]		1. Denusomab	50	1 and 2 years

For peer review only

For peer review only - http://bmjopen.bmj.com/site/about/guidelines.xhtml

Sweden, 2012-2014<br><br>2 centres		2. Placebo		KOOS pain: no significant difference
Continuous passive motion				
Bennett et al. 2005[61]<br><br>Australia, 1997-2000<br><br>1 hospital	Physiotherapy	1. Standard CPM<br><br>2. Early flexion CPM	147	1 year<br><br>KSS: no significant difference
Ersözlü et al. 2009[60]<br><br>Turkey, 2003-2004<br><br>1 hospital	Physiotherapy	1. CPM low and increasing<br><br>2. CPM high and increasing<br><br>3. No CPM	90	2 years<br><br>KSS: no difference (p=0.67)
Electrical stimulation				
Avramidis et al. 2011[62]<br><br>Greece, 2005-2006<br><br>1 hospital	Physiotherapy	1. Transcutaneous electric muscle stimulation<br><br>2. No treatment	76	1 year<br><br>SF-36 bodily pain: strong evidence favouring electrical stimulation (p<0.001). Mean difference (12.5/100) close to MCID (16.9/100).<br><br>OKS/ KSS: no difference
Moretti et al. 2012[64]<br><br>Italy, 2008-2010<br><br>1 hospital	Rehabilitation protocol	1. Pulsed electromagnetic fields<br><br>2. No treatment	30	6 months and 1 year<br><br>VAS pain: some evidence favouring electrical stimulation (p<0.05). Mean difference (2.1/10) greater than MCID (16.1/100)<br><br>Knee swelling: electrical stimulation concern
Rehabilitation				
Li et al. 2017[66]<br><br>China, 2015-2016<br><br>1 hospital	Standard rehabilitation	1. Walking guidance and training<br><br>2. No treatment	86	6 months<br><br>VAS pain/ HSS score: some evidence favouring walking (both p<0.01). Mean VAS pain

For peer review only - http://bmjopen.bmj.com/site/about/guidelines.xhtml

				difference (2.4/100) greater than MCID (16.1/100)
Liebs et al. 2012[68]<br><br>Germany, 2003-2004<br><br>4 hospitals	CPM, physiotherapy, post-discharge aquatic therapy	1. Early aquatic therapy<br><br>2. Delayed aquatic therapy	185	6 months, 1 and 2 years<br><br>WOMAC pain: no difference (p=0.22 at 12 months)
Mahomed et al. 2008[69]<br><br>Canada, 2000-2002<br><br>2 centres	Physiotherapy	1. Multidisciplinary supported early discharge and home physiotherapy<br><br>2. Transfer to rehabilitation centre	234 hip or knee replace ment	1 year<br><br>WOMAC pain: weak evidence favouring supported discharge (p=0.08). Mean difference (4) less than MCID (8-9)
Wang et al. 2014[67]<br><br>China, 2009-2010<br><br>1 centre		1. Wound closure in flexion<br><br>2. Wound closure in extension	80	6 months<br><br>VAS pain: no difference (p=0.64)
Wound management				
Kong et al. 2014[58]<br><br>South Korea, 2011<br><br>1 surgeon	Skin staples and closure strip	1. Silicone gel<br><br>2. Petroleum gel	100	6 months and 1 year<br><br>VAS pain: no difference (6 months p=0.886, 1 year p=0.201)
Anabolic steroids				
Hohmann et al. 2010[70]<br><br>Australia, Before 2010<br><br>1 surgeon	CPM. Cold compression,	1. Intramuscular nandrolone injections<br><br>2. Saline injections	10	6 and 9 months, 1 year<br><br>KSS: some evidence favouring nandrolone (6 months p=0.04, 9 months p=0.06, 12 months p=0.03). Difference at 12 months (10.2) close to MCID (12.3)<br><br>Bone mineral density: weak evidence favouring nandrolone

ACB adductor canal block; CPM Continuous passive motion; DN4 Douleur Neuropathique 4; FNB Femoral nerve block; HSS Hospital for Special Surgery; KOOS Knee injury and Osteoarthritis Outcome Score; KSS Knee Society Score; LIA local infiltration analgesia; MCID minimal clinically important difference; NRS Numerical rating scale; OKS Oxford Knee Score; PCA Patient controlled analgesia; SF-36 Short Form 36 Health Survey; S-LANSS Leeds assessment of Neuropathic Symptoms and Signs Pain

For peer review only - http://bmjopen.bmj.com/site/about/guidelines.xhtml

Scale; SNB Sciatic nerve block; VAS Visual analogue scale; VTE Venous thromboembolism; WOMAC Western Ontario and McMaster Universities Osteoarthritis Index.

For peer review only

For peer review only - http://bmjopen.bmj.com/site/about/guidelines.xhtml

Pain management

We identified 20 RCTs evaluating components of multi-modal pain management.

Femoral nerve block

Femoral nerve blocks (FNB) were studied in 10 RCTs.

Three RCTs compared FNB with no FNB. In one study with 55 patients, WOMAC pain scores at one year were similar in patients receiving single-shot FNB and untreated controls[22]. All patients received local anaesthetic infiltration (LIA) and patient-controlled analgesia (PCA). In another study with all participants receiving LIA, 150 were randomised to receive single-shot FNB with or without sciatic nerve block (SNB), or general anaesthesia[23]. There were no differences in HSS scores between groups at six months. Continuous FNB was compared with oral hydrocodone opioid in 62 patients receiving PCA[24]. There was some evidence for 'pain using stairs' favouring hydrocodone (p=0.01) but no difference in overall NRS-rated pain at one year and concern over venous thromboembolism in 4/31 participants treated with hydrocodone.

In two RCTs, continuous FNB was compared with PCA. In one study with 60 participants, the KSS at six months was similar between groups[25]. In another study with 280 participants, there was some evidence for higher incidence of NRS-rated pain at six months in the PCA group than the FNB group (p=0.021) but not at 12 months (p=0.273).[26]

Two RCTs compared FNB with LIA. In one study, all 157 participants also received PCA[27]. At one year, KSS values were similar in single-shot FNB and LIA groups. In the other study, 94 participants were randomised to receive single-shot FNB with continuous epidural infusion or LIA through an intra-articular catheter[28]. VAS-rated pain was similar between groups at one year.

In two RCTs, FNB procedures were compared. In one study with 99 patients randomised to two FNB concentrations, there was no difference in WOMAC score between groups at 12 months[29]. In another study with 61 participants allocated to two different durations of FNB, there was no difference in WOMAC pain scores at one year[30]. In these studies, all participants received either SNB[29] or PCA[30].

Single-shot FNB was compared with single adductor canal block in one RCT with 98 participants, all receiving LIA[31]. At six months there was no difference in VAS-rated pain.

Sciatic nerve block

For peer review only - http://bmjopen.bmj.com/site/about/guidelines.xhtml

In one study, 89 patients were randomised to single-shot SNB, continuous SNB, or PCA[32]. All patients received FNB. At 12 months, there were no differences in pain for single-shot SNB and continuous SNB on the WOMAC pain scale or VAS-rated pain at rest or during mobilisation. Similarly, there were no differences between single-shot SNB and PCA in WOMAC pain scale or VAS-rated pain at rest or during mobilisation, or between continuous SNB and PCA.

Local anaesthetic infiltration

Four RCTs compared LIA with placebo. In one study, all 280 participants received FNB and PCA[33]. There was weak evidence that WOMAC pain scores were better in the LIA group at six (p=0.063) but not at 12 months (p=0.107) when the difference in means of 3.8/100 was lower than the MCID of 8-9/100 reported by Ehrich and colleagues[34]. In another study, 56 patients received LIA including ketorolac, or saline placebo, and all received PCA[35]. At one year, mean differences and confidence intervals provided weak evidence that OKS scores were better in the LIA group but the difference in means of 2.7/48 was less than the MCID of 4/48 reported by Beard and colleagues[36]. LIA before surgical incision was compared with placebo in one study with 120 participants[37]. None received FNB or PCA. There was weak evidence for a better KSS (function and knee score components) at six months in those receiving LIA (p=0.07) with a difference in means of 14.2/200 exceeding the MCID of 12.3/200 reported by Lee and colleagues[38]. In another study, all 51 participants received LIA intra-operatively, followed by PCA[39]. Those randomised to post-operative catheter-delivered LIA with ketorolac, or saline placebo had similar VAS-rated pain at six and 12 months.

LIA delivered as an injection and post-operative infusion was compared with epidural PCA in one study with 222 patients[40]. There was no difference between groups in OKS at 12 months.

In one study of 100 participants, LIA with or without corticosteroid were compared[41]. All patients received PCA. At two years there was no difference in OKS between groups.

Oral celecoxib

In one RCT, 44 participants received oral celecoxib or placebo[42], as well as PCA. There were no differences between groups in KOOS or VAS-rated pain at 12 months.

Ketamine or nefopam infusion

In one RCT, ketamine infusion, nefopam infusion and saline placebo were compared in 75 patients, all of whom received PCA[43]. There was weak evidence that participants receiving ketamine or nefopam had lower VAS-rated pain on movement at 12 months. For the Douleur

For peer review only - http://bmjopen.bmj.com/site/about/guidelines.xhtml

Neuropathique 4 (DN4) measure of neuropathic pain, there was some evidence favouring ketamine over placebo at 6 and 12 months (p=0.02), but overall, few patients reported neuropathic pain at 12 months.

Pregabalin

Oral pregabalin was compared with placebo in one RCT with 240 participants[44]. All received LIA and PCA. At six months, no participants receiving pregabalin reported neuropathic pain when assessed using the Leeds assessment of Neuropathic Symptoms and Signs Pain Scale, compared with 5.2% of those receiving placebo (p=0.014) which represents some evidence favouring pregabalin.

Tourniquet

Five studies explored tourniquet use to provide a bloodless field.

In three RCTs, participants received TKR with or without a tourniquet. In one study with 64 patients, a difference in KOOS pain favouring tourniquet use was not significant at six or 12 months[45]. In another study with 20 patients, the OKS was not significantly different between groups at six or 12 months[46]. There were three blood transfusions in the tourniquet group, compared with none in the ‘no tourniquet’ group. In the third study with 100 participants, VAS-rated pain and HSS scores were similar between groups at 6 months[47]. Six cases of wound ooze occurred in the tourniquet group.

In two RCTs, short and long-duration tourniquet use were compared. In one study with 65 participants, there was weak evidence based on graphical representation of means and confidence intervals for improved OKS at 12 months in the long-duration group and the difference in means of 5/48[48] was greater than the MCID of 4/48. Adverse events were reported by 62% of participants receiving short-duration tourniquet compared with 38% in the long-duration group. The study was terminated early as 10 blood transfusions were required in the short-duration group compared with three in the long-duration group. In the second study with 150 participants, tourniquets were used in three different periods during surgery[49]. At six months, there were no differences between groups in HSS scores.

Blood conservation

Seven studies evaluated strategies to limit blood loss after TKR.

Tranexamic acid

For peer review only - http://bmjopen.bmj.com/site/about/guidelines.xhtml

Five RCTs evaluated tranexamic acid.

Tranexamic acid injections or infusions were compared with saline placebo or untreated control in four RCTs[47,50-52]. In all studies, control patients required more blood transfusions. In one study including 180 participants comparing intravenous tranexamic acid with untreated controls, there was no significant difference in WOMAC pain scores at one year[51]. In another study with 48 participants comparing intra-articular tranexamic acid injection with saline placebo, there was no significant difference in WOMAC scores at six months[50]. One study with 135 participants compared two intra-articular tranexamic acid doses and saline control[52]. There were no significant differences in WOMAC scores at one year. Intravenous and intra-articular tranexamic was compared with untreated controls in one study with 100 participants[47]. VAS-rated pain at six months was similar between groups, but there was strong evidence favouring tranexamic acid for HSS scores ($p<0.001$) although the difference in means of 1.4/100 was lower than the MCID of 8.3/100 reported by Singh and colleagues[53].

In one study, continuous tranexamic acid infusion was compared with a single bolus in 106 patients[54]. There was no difference between groups in KSS at six months or blood loss.

Thrombin infusion

In one RCT with 80 participants, thrombin infusion was compared with untreated control[55]. At one year there was no difference between groups in pain measured on the KSS.

Flexion or extension

For blood management, operated knees were kept in passive flexion or passive extension after surgery in one RCT with 180 patients[56]. At one year, OKS was similar between groups. Transfusion requirement was greater in patients with passive extension.

Compression bandage

One RCT with 49 participants compared compression bandaging to reduce post-operative knee swelling with standard bandaging. OKS was similar in randomised groups at six months[57].

Wound management

One RCT evaluated a wound care strategy to limit post-operative scar pain. Investigators compared silicone gel application to the surgical scar with placebo in 100 participants[58]. There were no significant differences in VAS-rated pain at six and 12 months.

Denusomab

For peer review only - http://bmjopen.bmj.com/site/about/guidelines.xhtml

For peer review only

One RCT evaluated use of the antiresorptive monoclonal antibody Denusomab to promote bone healing. Fifty participants were randomised and at 12 and 24 months there were no significant differences between groups in KOOS pain[59].

Continuous passive motion

Two RCTs evaluated use of continuous passive motion (CPM) to minimise joint stiffness and improve range of movement. In one study, 90 participants were randomised to no CPM, CPM at low flexion from post-operative day 1–7, or CPM at high flexion from post-operative day 3–7[60]. There was no significant difference between groups in KSS at two years. In the other study, 147 participants were randomised to CPM with increasing range of movement from day 1–6, early flexion CPM from day 0–6, or no CPM[61]. There were no significant differences between groups in KSS at 12 months.

Electrical stimulation

Two RCTs evaluated electrical stimulation which is believed to have anti-inflammatory activity and limit muscle atrophy. In one study with 76 participants receiving transcutaneous electric muscle stimulation from post-operative day two for six weeks or no intervention, Short Form 36 bodily pain showed strong evidence for greater improvement at one year in the intervention group compared to control ($p<0.001$)[62]. The difference in means of 12.5/100 was close to the MCID of 16.9/100 reported by Escobar and colleagues[63]. There were no differences in OKS or KSS scores. In another study with 30 participants, pulsed electromagnetic fields from post-operative day 7 were compared with untreated control[64]. At 12 months, there was some evidence that VAS-rated pain was lower in intervention patients compared with controls ($p<0.05$). The difference in means of 2.1/10 was greater than the MCID of 16.1/100 reported by Danoff and colleagues[65]. Knee swelling was common during the intervention.

Rehabilitation

Four RCTs evaluated features of early rehabilitation focusing on regaining range of movement, functional independence and improving mobility.

Walking guidance and training

In one study, 86 participants were randomised to walking guidance and training from post-operative day two or no intervention further to standard rehabilitation[66]. At six months, there was some evidence that those receiving intervention had lower VAS-rated pain ($p<0.01$) and

For peer review only - http://bmjopen.bmj.com/site/about/guidelines.xhtml

HSS score (p<0.01) than controls. The difference in mean VAS-rated pain of 2.4/10 was greater than the MCID of 16.1/100.

Flexion or extension during knee closure

Targeting improved functional recovery, wound closure performed in 90° flexion was compared with wound closure in full extension in one study with 80 participants[67]. There was no difference between groups in VAS-rated pain at six months.

Aquatic therapy

In one study with 185 participants, aquatic therapy commenced on post-operative day six or 14 were compared[68]. Patients reported similar WOMAC pain at 12 and 24 months.

Supported early discharge

In one study, early discharge supported by physiotherapist home visits and outpatient or self-directed physiotherapy was compared with two week rehabilitation centre-based usual care[69]. The study included 234 individuals receiving TKR or total hip replacement. Compared with usual care, there was weak evidence that patients with early discharge had lower WOMAC pain scores at 12 months (p=0.08). The difference in means of 4 was less than the MCID of 8-9/100. Results were not presented separately but did not differ between patients with TKR or total hip replacement.

Anabolic steroids

Searches identified one study of anabolic steroids to improve post-operative muscle strength. Ten participants received intramuscular nandrolone injections or saline from post-operative day five for six months. KSS results indicated some evidence for improvement in the intervention group compared with controls at 12 months (p=0.03)[70]. The difference in means of 10.2/200 was close to the MCID of 12.3/200.

Interventions with no long-term outcome

Interventions with lack of RCT evidence are summarised in Figure 1.

While 148 RCTs of deep vein thrombosis (DVT) prophylaxis were identified, only five reported long-term follow up, none of which included a pain or outcome score. Among 29 RCTs of antibiotic prophylaxis, 16 reported long-term follow up, but none included a pain or outcome score. Six RCTs evaluated the use of bisphosphonates and, although all reported long-term

For peer review only - http://bmjopen.bmj.com/site/about/guidelines.xhtml

follow up, none reported pain or an outcome score. One study reported long-term follow up of an RCT of teriparatide but included no data on pain.

For some interventions, RCTs with long-term pain outcomes were identified, but none were at low risk of bias: cold therapy; guided imagery; platelet rich plasma; and trigger point needling.

Aspects of peri-operative care evaluated in RCTs but lacking long-term pain follow up were: adenosine triphosphate; alternative and Chinese medicine; assistive devices; brain stimulation; calcium supplements; cardiovascular drugs; colloids and crystalloids; comorbidity management; constipation treatment; creatine; delirium prevention; dexmedetomidine; glucocorticoids; glucose infusion; iron; laser therapy; methylprednisolone; music therapy; nausea prevention; nutritional supplements; physiological treatments; remote ischaemic preconditioning; sleep treatments; therapy dogs; and warming.

DISCUSSION

Peri-operative care for patients with osteoarthritis receiving TKR varies widely[71,72]. To guide decisions on appropriate care, the top level of evidence in the hierarchy of primary research is the RCT[73,74]. Bringing evidence from RCTs together in systematic reviews with thorough risk of bias assessment ensures that health professionals have the information they need to deliver a high-quality patient experience with safe, clinically-effective and cost-effective treatments[75]. Furthermore, systematic reviews can identify gaps in the evidence base and promote further research.

Much research in TKR aims to identify treatments that facilitate a speedy recovery with minimal short-term pain. However, patients choose to have joint replacement for long-term pain relief and reduction in functional limitations. Thus, changes to peri-operative care, supported by short-term RCT evidence, should be backed up with evidence about long-term effectiveness for reducing pain and reassurance that there are no long-term unfavourable consequences. To this end, we synthesised evidence from RCTs evaluating peri-operative interventions which have considered their long-term effects on pain outcomes.

A major focus of research into improving long-term pain after TKR has been through prevention of acute post-operative pain using multimodal analgesia. Our review provides some encouragement for further research on long-term benefits of intra-articular LIA injections, as previously shown in short-term studies[19,76], ketamine infusion, oral pregabalin and oral opioids. Nerve blocks are effective for managing peri-operative pain[77] but we identified no long-term benefit. In single studies, there was no benefit for nefopam infusion, oral celecoxib or

For peer review only - http://bmjopen.bmj.com/site/about/guidelines.xhtml

LIA with additional corticosteroid. Regarding future studies, standardisation of the multi-modal regimen will allow evaluation of extra or alternative components in multiple studies in different settings. With such an approach, convincing evidence will accrue to guide multimodal pain management.

Tranexamic acid is highly effective in reducing blood transfusions during TKR[78]. We found no evidence that tranexamic acid affects long-term pain or, as observed in registry studies[79,80], adverse events. Single RCTs of thrombin infusion and maintenance of knee in flexion to prevent blood loss showed no effect on long-term pain. Tourniquets improve intraoperative visualisation of the joint, reduce blood loss and facilitate cement fixation but are associated with nerve damage, delayed recovery, acute pain and need for analgesics[81,82]. The RCTs we identified showed no effects of tourniquet use on long-term pain.

Consistent with a previous review[83], there was no suggestion that CPM affects long-term pain. Studies provided encouragement for further research into walking training, anabolic steroid injection, electrical stimulation and supported discharge.

For some interventions a direct mechanism is clear, but for others, reasons for long-term impact are less obvious. This may explain why no studies evaluated DVT prophylaxis with long-term follow up excepting a small number reporting adverse events. However, treatments to prevent symptomatic DVTs which occur in about 1% of treated patients[84] also reduce the incidence of asymptomatic DVT observed in about 28% of treated patients[85] and this may have long-term benefits. Conversely, new anticoagulants are associated with bleeding[86], which may increase the risk of wound complications[87] and joint infection[88] which are associated with long-term pain[89,90].

Our study is limited by the lack of meta-analysis which was not appropriate due to intervention and outcome heterogeneity. In the context of perioperative pain management, this was noted previously[76]. Our approach to assessing the evidence was a narrative synthesis of studies with low risk of bias. While this may seem overly restrictive, Cochrane risk of bias assessment allows us to screen out studies with important issues that may affect the validity of results. The main potential source of bias was incomplete outcome assessment. Although studies with long-term follow up are naturally at higher risk of missing data, we maintained a standard in this domain as it is recognised that research participants who do not complete follow up assessments differ in outcomes from those with follow up data and their inclusion could change the interpretation of results[91].

For peer review only - http://bmjopen.bmj.com/site/about/guidelines.xhtml

We summarised p-values to assess the strength of evidence but, as statistically strong evidence may not reflect clinically important results[92], where possible we also compared effect sizes with MCIDs. Our review considered a diverse range of interventions at a specific time in the TKR pathway and, as we were unable to make clinical practice recommendations, we did not adopt the GRADE system[93] for this review.

Our systematic review of peri-operative interventions brings together evidence on interventions in the peri-operative phase of the TKR pathway. Whilst not supportive of the inclusion of specific interventions in clinical practice to optimise long-term pain outcomes, there are clearly areas that merit research. High quality studies assessing long-term pain after peri-operative interventions are feasible and necessary to ensure that patients with osteoarthritis achieve good long-term outcomes after TKR.

AUTHOR CONTRIBUTIONS

All authors contributed to the conception and design of the study. ADB, JD and VW undertook the systematic review. ADB and JD carried out the risk of bias assessments. ADB drafted the article with revisions by JD and VW. All authors approved the final version for publication.

ROLE OF THE FUNDING SOURCE

This article presents independent research funded by the National Institute for Health Research (NIHR) under its Programme Grants for Applied Research programme (RP-PG-0613-20001). This study was supported by the NIHR Biomedical Research Centre at University Hospitals Bristol NHS Foundation Trust and the University of Bristol. The views expressed in this publication are those of the authors and not necessarily those of the NHS, the National Institute for Health Research or the Department of Health and Social Care.

COMPETING INTERESTS STATEMENT

The authors report no competing interests.

DATA STATEMENT

All extracted data is included in the Supplementary material.

REFERENCES

1. Weinstein AM, Rome BN, Reichmann WM, et al. Estimating the burden of total knee replacement in the United States. *J Bone Joint Surg (Am)* 2013;95:385-92.

For peer review only - http://bmjopen.bmj.com/site/about/guidelines.xhtml

2. National Joint Registry for England, Wales, Northern Ireland and the Isle of Man: 15th Annual Report. Hemel Hempstead: NJR Centre 2018.

3. Scottish Arthroplasty Project. Annual report 2018. Edinburgh: NHS National Services Scotland; 2018.

4. Maradit Kremers H, Larson DR, Crowson CS, et al. Prevalence of total hip and knee replacement in the United States. *J Bone Joint Surg (Am)* 2015;97:1386-97.

5. Nilsdotter AK, Toksvig-Larsen S, Roos EM. Knee arthroplasty: are patients' expectations fulfilled? A prospective study of pain and function in 102 patients with 5-year follow-up. *Acta Orthop* 2009;80:55-61.

6. Scott CE, Howie CR, MacDonald D, et al. Predicting dissatisfaction following total knee replacement: a prospective study of 1217 patients. *J Bone Joint Surg (Br)* 2010;92:1253-8.

7. Bachmeier CJM, March LM, Cross MJ, et al. A comparison of outcomes in osteoarthritis patients undergoing total hip and knee replacement surgery. *Osteoarthritis Cart* 2001;9:137-46.

8. Dailiana ZH, Papakostidou I, Varitimidis S, et al. Patient-reported quality of life after primary major joint arthroplasty: a prospective comparison of hip and knee arthroplasty. *BMC Musculoskelet Disord* 2015;16:366.

9. Werner MU, Kongsgaard UE. Defining persistent post-surgical pain: is an update required? *Br J Anaesth* 2014;113:1-4.

10. Blom AW, Artz N, Beswick AD, et al. Improving patients' experience and outcome of total joint replacement: The RESTORE programme. *Programme Grants Appl Res* 2016;4.

11. Kehlet H, Jensen TS, Woolf CJ. Persistent postsurgical pain: risk factors and prevention. *Lancet* 2006;367:1618-25.

12. Haroutiunian S, Nikolajsen L, Finnerup NB, et al. The neuropathic component in persistent postsurgical pain: A systematic literature review. *Pain* 2013;154:95-102.

13. Ashrani AA, Heit JA. Incidence and cost burden of post-thrombotic syndrome. *J Thromb Thrombolysis* 2009;28:465-76.

14. Dreyer HC. Tourniquet use during knee replacement surgery may contribute to muscle atrophy in older adults. *Exerc Sport Sci Rev* 2016;44:61-70.

15. Kindsfater K, Scott R. Recurrent hemarthrosis after total knee arthroplasty. *J Arthroplasty* 1995;10:S52-S5.

16. Edwards RR, Haythornthwaite JA, Smith MT, et al. Catastrophizing and depressive symptoms as prospective predictors of outcomes following total knee replacement. *Pain Res Manag* 2009;14:307-11.

For peer review only

For peer review only - http://bmjopen.bmj.com/site/about/guidelines.xhtml

17. Wylde V, Beswick AD, Dennis J, et al. Post-operative patient-related risk factors for chronic pain after total knee replacement: a systematic review. *BMJ Open* 2017;7:e018105.
18. Moher D, Liberati A, Tetzlaff J, et al. Preferred reporting items for systematic reviews and meta-analyses: the PRISMA statement. *J Clin Epidemiol* 2009;62:1006-12.
19. Marques EM, Jones HE, Elvers KT, et al. Local anaesthetic infiltration for peri-operative pain control in total hip and knee replacement: systematic review and meta-analyses of short- and long-term effectiveness. *BMC Musculoskelet Disord* 2014;15:220.
20. Higgins JPT, Green S. Cochrane Handbook for Systematic Reviews of Interventions Version 5.1.0 [Updated March 2011]: The Cochrane Collaboration; 2011. Available from: http://www.cochrane-handbook.org.
21. Sterne JA, Davey Smith G. Sifting the evidence-what's wrong with significance tests? *BMJ* 2001;322:226-31.
22. Widmer BJ, Scholes CJ, Pattullo GG, et al. Is femoral nerve block necessary during total knee arthroplasty?: a randomized controlled trial. *J Arthroplasty* 2012;27:1800-5.
23. Gao WL, Li H, Liu BQ, et al. Analgesic effect of femoral and sciatic nerve block under multimodal analgesia in total knee arthroplasty. *Chinese Journal of Tissue Engineering Research* 2017;21:2966-72.
24. Nader A, Kendall MC, Wixson RL, et al. A randomized trial of epidural analgesia followed by continuous femoral analgesia compared with oral opioid analgesia on short- and long-term functional recovery after total knee replacement. *Pain Med* 2012;13:937-47.
25. Wu JWS, Wong YC. Elective unilateral total knee replacement using continuous femoral nerve blockade versus conventional patient-controlled analgesia: perioperative patient management based on a multidisciplinary pathway. *Hong Kong Med J* 2014;20:45-51.
26. Peng L, Ren L, Qin P, et al. Continuous femoral nerve block versus intravenous patient controlled analgesia for knee mobility and long-term pain in patients receiving total knee replacement: a randomized controlled trial. *Evid Based Complement Alternat Med* 2014;2014:569107.
27. Fan L, Yu X, Zan P, et al. Comparison of local infiltration analgesia with femoral nerve block for total knee arthroplasty: A prospective, randomized clinical trial. *J Arthroplasty* 2016;31:1361-5.
28. Reinhardt KR, Duggal S, Umunna BP, et al. Intraarticular analgesia versus epidural plus femoral nerve block after TKA: A randomized, double-blind trial. *Clin Orthop Relat Res* 2014;472:1400-8.

For peer review only - http://bmjopen.bmj.com/site/about/guidelines.xhtml

29. Albrecht E, Morfey D, Chan V, et al. Single-injection or continuous femoral nerve block for total knee arthroplasty? *Clin Orthop Relat Res* 2014;472:1384-93.

30. Choy WS, Lee SK, Kim KJ, et al. Two continuous femoral nerve block strategies after TKA. *Knee Surg Sports Traumatol Arthrosc* 2011;19:1901-8.

31. Macrinici GI, Murphy C, Christman L, et al. Prospective, double-blind, randomized study to evaluate single-injection adductor canal nerve block versus femoral nerve block: postoperative functional outcomes after total knee arthroplasty. *Reg Anesth Pain Med* 2017;42:10-6.

32. Wegener JT, van Ooij B, van Dijk CN, et al. Long-term pain and functional disability after total knee arthroplasty with and without single-injection or continuous sciatic nerve block in addition to continuous femoral nerve block: a prospective, 1-year follow-up of a randomized controlled trial. *Reg Anesth Pain Med* 2013;38:58-63.

33. Wylde V, Lenguerrand E, Gooberman-Hill R, et al. Effect of local anaesthetic infiltration on chronic postsurgical pain after total hip and knee replacement: the APEX randomised controlled trials. *Pain* 2015;156:1161-70.

34. Ehrich EW, Davies GM, Watson DJ, et al. Minimal perceptible clinical improvement with the Western Ontario and McMaster Universities osteoarthritis index questionnaire and global assessments in patients with osteoarthritis. *J Rheumatol* 2000;27:2635-41.

35. Niemeläinen M, Kalliovalkama J, Aho A, et al. Single periarticular local infiltration analgesia reduces opiate consumption until 48 hours after total knee arthroplasty. A randomized placebo-controlled trial involving 56 patients. *Acta Orthop* 2014;85:614-9.

36. Beard DJ, Harris K, Dawson J, et al. Meaningful changes for the Oxford hip and knee scores after joint replacement surgery. *J Clin Epidemiol* 2015;68:73-9.

37. Motififard M, Omidian A, Badiei S. Pre-emptive injection of peri-articular-multimodal drug for post-operative pain management in total knee arthroplasty: a double-blind randomized clinical trial. *Int Orthop* 2017;41:939-47.

38. Lee WC, Kwan YH, Chong HC, et al. The minimal clinically important difference for Knee Society Clinical Rating System after total knee arthroplasty for primary osteoarthritis. *Knee Surg Sports Traumatol Arthrosc* 2017;25:3354-9.

39. Williams D, Petruccelli D, Paul J, et al. Continuous infusion of bupivacaine following total knee arthroplasty: a randomized control trial pilot study. *J Arthroplasty* 2013;28:479-84.

40. McDonald DA, Deakin AH, Ellis BM, et al. The technique of delivery of peri-operative analgesia does not affect the rehabilitation or outcomes following total knee arthroplasty. *Bone Joint J* 2016;98-B:1189-96.

For peer review only - http://bmjopen.bmj.com/site/about/guidelines.xhtml

41. Seah VWT, Chin PL, Chia SL, et al. Single-dose periarticular steroid infiltration for pain management in total knee arthroplasty: a prospective, double-blind, randomised controlled trial. *Singapore Med J* 2011;52:19-23.

42. Meunier A, Lisander B, Good L. Effects of celecoxib on blood loss, pain, and recovery of function after total knee replacement: a randomized placebo-controlled trial. *Acta Orthop* 2007;78:661-7.

43. Aveline C, Roux AL, Hetet HL, et al. Pain and recovery after total knee arthroplasty: a 12-month follow-up after a prospective randomized study evaluating Nefopam and Ketamine for early rehabilitation. *Clin J Pain* 2014;30:749-54.

44. Buvanendran A, Kroin JS, Della Valle CJ, et al. Perioperative oral pregabalin reduces chronic pain after total knee arthroplasty: a prospective, randomized, controlled trial. *Anesth Analg* 2010;110:199-207.

45. Ejaz A, Laursen AC, Kappel A, et al. Faster recovery without the use of a tourniquet in total knee arthroplasty. *Acta Orthop* 2014;85:422-6.

46. Liu D, Graham D, Gillies K, et al. Effects of tourniquet use on quadriceps function and pain in total knee arthroplasty. *Knee Surg Relat Res* 2014;26:207-13.

47. Huang Z, Xie X, Li L, et al. Intravenous and topical tranexamic acid alone are superior to tourniquet use for primary total knee arthroplasty: A prospective, randomized controlled trial. *J Bone Joint Surg (Am)* 2017;99:2053-61.

48. Mittal R, Ko V, Adie S, et al. Tourniquet application only during cement fixation in total knee arthroplasty: a double-blind, randomized controlled trial. *ANZ J Surg* 2012;82:428-33.

49. Zhang Y, Li D, Liu P, et al. Effects of different methods of using pneumatic tourniquet in patients undergoing total knee arthroplasty: a randomized control trial. *Ir Jr Med Sci* 2017;186:953-9.

50. Sa-Ngasoongsong P, Channoom T, Kawinwonggowit V, et al. Postoperative blood loss reduction in computer-assisted surgery total knee replacement by low dose intra-articular tranexamic acid injection together with 2-hour clamp drain: a prospective triple-blinded randomized controlled trial. *Orthop Rev* 2011;3:e12.

51. Kim TK, Chang CB, Kang YG, et al. Clinical value of tranexamic acid in unilateral and simultaneous bilateral TKAs under a contemporary blood-saving protocol: a randomized controlled trial. *Knee Surg Sports Traumatol Arthrosc* 2014;22:1870-8.

52. Sa-Ngasoongsong P, Wongsak S, Chanplakorn P, et al. Efficacy of low-dose intra-articular tranexamic acid in total knee replacement; a prospective triple-blinded randomized controlled trial. *BMC Musculoskelet Disord* 2013;14:340.

For peer review only - http://bmjopen.bmj.com/site/about/guidelines.xhtml

53. Singh JA, Schleck C, Harmsen WS, et al. Validation of the hospital for special surgery knee questionnaire: Convergent validity, responsiveness and sensitivity to change. ACR/ARHP; San Diego, California2013. p. 1926.

54. Hourlier H, Reina N, Fennema P. Single dose intravenous tranexamic acid as effective as continuous infusion in primary total knee arthroplasty: a randomised clinical trial. *Arch Orthop Trauma Surg* 2015;135:465-71.

55. Kusuma SK, Sheridan KC, Wasielewski RC. Use of bovine thrombin to reduce blood loss in primary total knee arthroplasty: a controlled randomized trial. *J Arthroplasty* 2013;28:1278-81.

56. Napier RJ, Bennett D, McConway J, et al. The influence of immediate knee flexion on blood loss and other parameters following total knee replacement. *Bone Joint J* 2014;96-B:201-9.

57. Brock TM, Sprowson AP, Muller S, et al. STICKS study - Short-sTretch Inelastic Compression bandage in Knee Swelling following total knee arthroplasty - a feasibility study. *Trials* 2017;18:6.

58. Kong CG, Kim GH, Kim DW, et al. The effect of topical scar treatment on postoperative scar pain and pruritus after total knee arthroplasty. *Arch Orthop Trauma Surg* 2014;134:555-9.

59. Ledin H, Good L, Aspenberg P. Denosumab reduces early migration in total knee replacement. *Acta Orthop* 2017;88:255-8.

60. Ersözlü S, Sahin O, Ozgür A, et al. The effects of two different continuous passive motion protocols on knee range of motion after total knee arthroplasty: a prospective analysis. *Acta Orthopaedica et Traumatologica Turcica* 2009;43:412-8.

61. Bennett LA, Brearley SC, Hart JAL, et al. A comparison of 2 continuous passive motion protocols after total knee arthroplasty: a controlled and randomized study. *J Arthroplasty* 2005;20:225-33.

62. Avramidis K, Karachalios T, Popotonasios K, et al. Does electric stimulation of the vastus medialis muscle influence rehabilitation after total knee replacement? *Orthopedics* 2011;34:175.

63. Escobar A, Quintana JM, Bilbao A, et al. Responsiveness and clinically important differences for the WOMAC and SF-36 after total knee replacement. *Osteoarthritis Cart* 2007;15:273-80.

64. Moretti B, Notarnicola A, Moretti L, et al. I-ONE therapy in patients undergoing total knee arthroplasty: a prospective, randomized and controlled study. *BMC Musculoskelet Disord* 2012;13:88.

For peer review only - http://bmjopen.bmj.com/site/about/guidelines.xhtml

65. Danoff JR, Goel R, Sutton R, et al. How much pain is significant? Defining the minimal clinically important difference for the visual analog scale for pain after total joint arthroplasty. *J Arthroplasty* 2018;33:S71-S5.

66. Li L, Wang Z, Yin MH, et al. Effect of early gait training on the functional rehabilitation after total knee arthroplasty. *Chinese Journal of Tissue Engineering Research* 2017;21:4288-93.

67. Wang S, Xia J, Wei Y, et al. Effect of the knee position during wound closure after total knee arthroplasty on early knee function recovery. *J Orthop Surg Res* 2014;9:79.

68. Liebs T, Herzberg W, Rüther W, et al. Multicenter randomized controlled trial comparing early versus late aquatic therapy after total hip or knee arthroplasty. *Arch Phys Med Rehabil* 2012;93:192-9.

69. Mahomed NN, Davis AM, Hawker G, et al. Inpatient compared with home-based rehabilitation following primary unilateral total hip or knee replacement: a randomized controlled trial. *J Bone Joint Surg (Am)* 2008;90:1673-80.

70. Hohmann E, Tetsworth K, Hohmann S, et al. Anabolic steroids after total knee arthroplasty. A double blinded prospective pilot study. *J Orthop Surg Res* 2010;5:93.

71. Mayer M, Naylor J, Harris I, et al. Evidence base and practice variation in acute care processes for knee and hip arthroplasty surgeries. *PLOS ONE* 2017;12:e0180090.

72. Kopp SL, Borglum J, Buvanendran A, et al. Anesthesia and analgesia practice pathway options for total knee arthroplasty: An evidence-based review by the American and European Societies of Regional Anesthesia and Pain Medicine. *Reg Anesth Pain Med* 2017;42:683-97.

73. Cochrane AL. Effectiveness and efficiency: random reflections on health services. London: The Nuffield Provincial Hospitals Trust; 1972.

74. Murad MH, Asi N, Alsawas M, et al. New evidence pyramid. *Evid Based Med* 2016;21:125-7.

75. National Institute for Health and Clinical Excellence. Patient experience in adult NHS services: improving the experience of care for people using adult NHS services. London: NICE; 2012.

76. Andersen LO, Kehlet H. Analgesic efficacy of local infiltration analgesia in hip and knee arthroplasty: a systematic review. *Br J Anaesth* 2014;113:360-74.

77. Chan E-Y, Fransen M, Parker DA, et al. Femoral nerve blocks for acute postoperative pain after knee replacement surgery. *Cochrane Database Syst Rev* 2014;5:CD009941.

78. Alshryda S, Sarda P, Sukeik M, et al. Tranexamic acid in total knee replacement: a systematic review and meta-analysis. *J Bone Joint Surg (Br)* 2011;93:1577-85.

For peer review only - http://bmjopen.bmj.com/site/about/guidelines.xhtml

79. Poeran J, Rasul R, Suzuki S, et al. Tranexamic acid use and postoperative outcomes in patients undergoing total hip or knee arthroplasty in the United States: retrospective analysis of effectiveness and safety. *BMJ* 2014;349.

80. Hallstrom B, Singal B, Cowen ME, et al. The Michigan experience with safety and effectiveness of tranexamic acid use in hip and knee arthroplasty. *J Bone Joint Surg (Am)* 2016;98:1646-55.

81. Noordin S, McEwen JA, Kragh JF, Jr., et al. Surgical tourniquets in orthopaedics. *J Bone Joint Surg (Am)* 2009;91:2958-67.

82. Horlocker TT, Hebl JR, Gali B, et al. Anesthetic, patient, and surgical risk factors for neurologic complications after prolonged total tourniquet time during total knee arthroplasty. *Anesth Analg* 2006;102:950-5.

83. Harvey LA, Brosseau L, Herbert RD. Continuous passive motion following total knee arthroplasty in people with arthritis. *Cochrane Database Syst Rev* 2014;2:CD004260.

84. Januel J, Chen G, Ruffieux C, et al. Symptomatic in-hospital deep vein thrombosis and pulmonary embolism following hip and knee arthroplasty among patients receiving recommended prophylaxis: A systematic review. *JAMA* 2012;307:294-303.

85. Schellong SM, Beyer J, Kakkar AK, et al. Ultrasound screening for asymptomatic deep vein thrombosis after major orthopaedic surgery: The VENUS study. *J Thromb Haemost* 2007;5:1431-7.

86. Gómez-Outes A, Terleira-Fernández AI, Suárez-Gea ML, et al. Dabigatran, rivaroxaban, or apixaban versus enoxaparin for thromboprophylaxis after total hip or knee replacement: systematic review, meta-analysis, and indirect treatment comparisons. *BMJ* 2012;344:e3675.

87. Freedman KB, Brookenthal KR, Fitzgerald RH, Jr., et al. A meta-analysis of thromboembolic prophylaxis following elective total hip arthroplasty. *J Bone Joint Surg (Am)* 2000;82-a:929-38.

88. Parvizi J, Ghanem E, Joshi A, et al. Does "excessive" anticoagulation predispose to periprosthetic infection? *J Arthroplasty* 2007;22:24-8.

89. Adelani MA, Johnson SR, Keeney JA, et al. Clinical outcomes following re-admission for non-infectious wound complications after primary total knee replacement. *Bone Joint J* 2014;96-b:619-21.

90. Cahill JL, Shadbolt B, Scarvell JM, et al. Quality of life after infection in total joint replacement. *J Orthop Surg* 2008;16:58-65.

For peer review only - http://bmjopen.bmj.com/site/about/guidelines.xhtml

91. Akl EA, Briel M, You JJ, et al. Potential impact on estimated treatment effects of information lost to follow-up in randomised controlled trials (LOST-IT): systematic review. *BMJ* 2012;344:e2809.

92. Leopold SS, Porcher R. Editorial: threshold p values in orthopaedic research—we know the problem. What is the solution? *Clin Orthop Relat Res* 2018;476:1689-91.

93. Guyatt GH, Oxman AD, Kunz R, et al. Going from evidence to recommendations. *BMJ* 2008;336:1049-51.

For peer review only

For peer review only - http://bmjopen.bmj.com/site/about/guidelines.xhtml

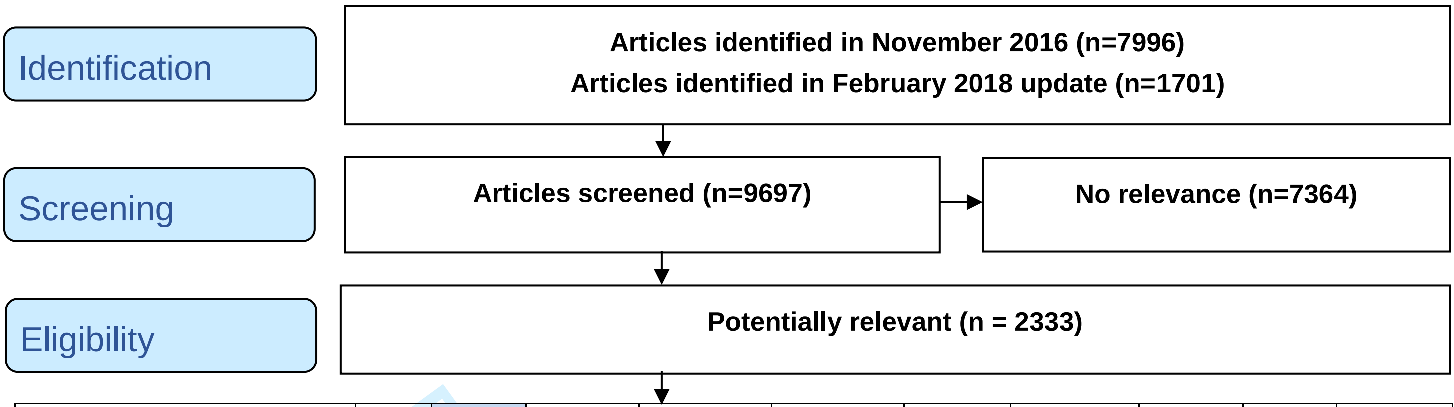

Intervention	N	In: low risk of bias	No pain/ score outcome	High/ unclear risk of bias	No long-term follow up	Abstract only	Additional publication	Protocol	Review	Retracted
Adenosine triphosphate	2	0	0	0	1	0	0	0	1	0
Alternative medicine	4	0	0	0	4	0	0	0	0	0
Anabolic steroids	2	1	0	0	0	0	0	0	1	0
Antibiotic prophylaxis	43	0	16	0	13	0	0	1	13	0
Assistive devices	2	0	0	0	2	0	0	0	0	0
Bisphosphonates	17	0	6	0	0	0	2	0	9	0
Blood management	355	7	10	1	209	0	0	4	124	0
Brain stimulation	3	0	0	0	3	0	0	0	0	0
Calcium supplement	1	0	0	0	1	0	0	0	0	0
Cardiovascular drugs	4	0	0	0	2	0	0	0	2	0
Chinese medicine	2	0	0	0	2	0	0	0	0	0
Cold therapy	30	0	0	1	24	0	0	0	5	0
Colloids and crystalloids	1	0	0	0	1	0	0	0	0	0
Comorbidity management	1	0	0	0	1	0	0	0	0	0
Compression	8	1	0	0	6	0	0	1	0	0
Constipation treatment	2	0	0	0	2	0	0	0	0	0
Continuous passive motion	56	2	8	7	23	1	0	1	14	0
Creatine monohydrate	1	0	0	0	1	0	0	0	0	0
Delirium prevention	4	0	0	0	3	0	0	0	1	0
Denusomab	1	1	0	0	0	0	0	0	0	0
Dexmedetomidine	1	0	0	0	1	0	0	0	0	0
Deep vein thrombosis prophylaxis	474	0	5	0	143	0	4	8	314	0
Electrical stimulation	37	2	0	3	20	0	2	0	10	0
Glucocorticoid	2	0	0	0	0	0	0	0	2	0
Glucose infusion	1	0	0	0	1	0	0	0	0	0
Guided imagery	5	0	0	1	4	0	0	0	0	0
Iron	7	0	0	0	6	0	0	0	1	0
Laser therapy	1	0	0	0	1	0	0	0	0	0
Methylprednisolone	3	0	0	0	3	0	0	0	0	0
Music therapy	9	0	0	0	9	0	0	0	0	0
Nausea prevention	11	0	0	0	9	0	2	0	0	0
Nutritional supplements	4	0	0	0	4	0	0	0	0	0
Pain management	987	20	5	12	711	1	20	9	207	2
Physiological	26	0	0	0	23	0	0	1	2	0
Platelet rich plasma	12	0	0	1	6	0	0	0	5	0
Rehabilitation	67	4	0	2	43	0	0	1	17	0
Remote ischaemic pre-conditioning	5	0	0	0	5	0	0	0	0	0
Sleep treatment	3	0	0	0	2	0	1	0	0	0
Teriparatide	1	0	1	0	0	0	0	0	0	0
Therapy dogs	1	0	0	0	1	0	0	0	0	0
Tourniquet use	100	5	3	3	67	0	2	1	19	0
Trigger point needling	1	0	0	1	0	0	0	0	0	0
Warming	19	0	0	0	16	0	0	0	3	0
Wound management	17	1	0	0	12	0	0	1	3	0
**Total**	**2333**	**44**	**54**	**32**	**1385**	**2**	**33**	**28**	**753**	**2**

For peer review only

Supplementary material. Search strategy as applied in MEDLINE on Ovid

1 randomized controlled trial/ or randomized controlled trial.pt.

2 controlled clinical trial.pt.

3 randomized.ab.

4 placebo.ab.

5 randomly.ab

6 trial.ab

7 randomised.tw

8 1 or 2 or 3 or 4 or 5 or 6 or 7

9 review/

10 'systematic review$'.mp

11 9 or 10

12 8 or 11

13 Arthroplasty, Replacement, Knee/

14 Knee Prosthesis/

15 (arthoplast$ adj3 knee$).mp. [mp=title, abstract, original title, name of substance word, subject heading word, keyword heading word, protocol supplementary concept word, rare disease supplementary concept word, unique identifier]

16 (knee$ adj3 replac$).mp. [mp=title, abstract, original title, name of substance word, subject heading word, keyword heading word, protocol supplementary concept word, rare disease supplementary concept word, unique identifier]

17 (knee adj3 implant$).mp. [mp=title, abstract, original title, name of substance word, subject heading word, keyword heading word, protocol supplementary concept word, rare disease supplementary concept word, unique identifier]

18 13 or 14 or 15 or 16 or 17

19 12 and 18

For peer review only - http://bmjopen.bmj.com/site/about/guidelines.xhtml

For peer review only

Supplementary material. All peri-operative interventions with long-term pain or score follow up

1. Pain management

Author<br>Country<br>Recruitment dates<br>Setting	Indication<br>Number randomised intervention; control<br>Age<br>% female	Common anaesthesia<br>Group 1 (intervention)	Group 2 (intervention)	Group C (control)	Follow up<br>Losses to follow up intervention; control<br>Risk of bias issues<br>Key results
FNB single vs No FNB					
Widmer et al. 2012[22]<br>Australia<br>Before 2012<br>2 surgeons	Elective unilateral TKR<br>27; 28<br>Median 72.1 (IQR 64.4, 76.5); 69.4 (63.4, 75.5)<br>44.4%; 44.4%	Premedication 1-3mg i.v. midazolam. Propofol induction and sevoflurane general anaesthetic.<br>LIA with 200mg ropivacaine and 0.5mg adrenaline in 100ml saline.<br>PCA 20µg fentanyl at 5-minute intervals on demand until morning POD2. Then, oral oxycodone SR 10mg every 12 hours. Daily COX II inhibitor and paracetamol 1g every 6 hours as tolerated. For breakthrough pain, 5-10mg oxycodone immediate release every 3 hours as needed.<br>Ultrasound guided FNB with 100mg ropivacaine in 30ml saline		Sham setup for FNB. No identification or injection of femoral sheath	1 year<br>No losses to follow up reported<br>Low risk of bias<br>WOMAC pain (high score favourable) at 1 year: FNB and LIA median 2.0 (IQR 0, 2.8); LIA no FNB 1.0 (0, 2.0). p=0.74<br>No adverse events occurred in either group
FNB single vs ONB vs Control					
Bergeron et al. 2009[94]<br>Canada<br>2005-2006<br>1 centre	Primary unilateral TKR<br>19; 20; 20<br>Mean 65.1 (SE 2.0); 72 (1.8); 67 (1.3)<br>79%; 80%; 75%	Intraoperative sedation with iv propofol at discretion of anaesthesiologist. Lumbar spinal anaesthesia with 12mg 0.5% bupivacaine.<br>Postoperative i.v. PCA with fentanyl 50µg/ml set to deliver 25µg every 5 min as needed.<br>Celecoxib 100mg and acetaminophen 650mg on arrival in recovery room and every 12 and 6 hrs respectively. Breakthrough medication with intramuscular ketorolac 10 mg every 4 hrs.			1 year<br>Overall 32 lost to follow up<br>High risk of bias: only 27/59 patients followed up due to resource limitations.<br>No difference in HSS pain at rest or during activity at 1 year between the study groups.

For peer review only

For peer review only - http://bmjopen.bmj.com/site/about/guidelines.xhtml

		FNB with stimulator. 20ml 0.5% bupivacaine with 1/200,000 epinephrine.	ONB with stimulator. 20ml 0.5% bupivacaine with 1/200,000 epinephrine.	No injection but inguinal area prepared, and sham block performed behind drapes.	No long-term complications attributable to anaesthetic regimen
FNB continuous low dose vs FNB continuous high dose vs No FNB					
Shum et al. 2009[95]<br>Singapore<br>Before 2009<br>1 hospital	Unilateral TKR for osteoarthritis<br>20 (17 received treatment); 20 (18 received treatment); 20<br>Mean 66.7 (SD 8.4); 65.4 (8.4); 67.8 (5.5)<br>88%; 72%; 80%	Spinal anaesthesia induced with 2-3ml hyperbaric 0.5% bupivacaine. Intraoperative sedation with midazolam in increments of 0.5mg.<br>Intravenous PCA morphine (1mg/ml, on-demand bolus doses of 1 mg with 5 minute lockout, maximum dose 8 mg/hr)			2 years<br>16.4% of patients who received intervention lost to follow up<br>Unclear risk of bias due to differences in OKS and weight at baseline, and limited methodological details.<br>No separate pain outcome but mean OKS slightly more favourable in group with no FNB, 18.2 (SD 3.7) compared with combined FNB groups, 19.8 (5.4) but this was not significant.<br>No complications attributable to use of FNB
		Low dose continuous FNB at conclusion of TKR with ropivacaine 0.15% (10 ml/hr in the first 24 hours, followed by 5ml/hr in the next 24 hours)	High dose continuous FNB at conclusion of TKR with ropivacaine 0.2% (10 ml/hr in the first 24 hours, followed by 5 ml/hr in the next 24 hours)	No FNB	
SNB injection vs SNB continuous vs control					
Wegener et al. 2013[32]<br>The Netherlands<br>2008-2010<br>1 centre	TKR<br>29; 30; 30 (90 randomised)<br>Median 65 (range 43-81); 66 (43-83); 62 (50-79)<br>62%; 70%; 73%	Lorazepam 1mg 2 hours and acetaminophen 2g 1 hour before surgery. FNB with stimulating catheter: loading dose 20 ml levobupivacaine 0.375% and after 45 minutes a continuous infusion of levobupivacaine 0.125% 10 ml/hr. General anaesthesia induced with 3-5 µg/ml propofol infusion and remifentanil 0.5 µg/kg/min and maintained with 2-3 µg/ml at 0.1-0.25 µg/kg/min. Postoperatively, FNB changed to patient controlled FNB, 5ml bolus, 30-minute lockout; basal rate 6 ml/hr. i.v. morphine administered if needed. Postoperative analgesia with acetaminophen 1g 4 times daily. Diclofenac 50mg or tramadol 50mg 3 times daily. Tramadol 100mg before removal of nerve catheters. Morphine pain relief as required.			12 months<br>2;7;5 lost to follow up<br>Low risk of bias<br>Median WOMAC pain scores at 12 months: SNB injection 80 (range 25-100), SNB continuous 90 (55-100) and PCA only 90 (35-100), p=0.81. No difference between groups in VAS pain at rest (p=0.90) or during mobilisation (p=0.43).<br>No information on adverse events.

For peer review only - http://bmjopen.bmj.com/site/about/guidelines.xhtml

		Group Fs: SNB single injection. SNB loading dose of 20 ml levobupivacaine 0.375%.	Group FCS: SNB continuous infusion. SNB loading dose of 20 ml levobupivacaine 0.375%. Continuous infusion of levobupivacaine 0.125% 10 ml/hr started 45 mins after catheter placement. SNB maintained for 36 hours postoperatively (10 ml/hr).	Group F: No SNB. PCA via femoral nerve catheter	
**General anaesthesia vs FNB single vs FNB/ SNB single**					
Gao et al. 2017[23]<br>China<br>2014-2015<br>1 centre	Primary unilateral TKR for osteoarthritis<br>50; 50; 50<br>Mean 65.8 (SD 6.7); 66.4 (7.4); 67.6 (6.3)<br>81%; 80%; 76%	Pre-operative and post-operative celecoxib 0.2g twice daily.<br>100ml intra-operative LIA with ropivacaine 200mg and epinephrine 0.25 mg.			6 months<br>2; 1; 0<br>Low risk of bias<br>Mean HSS at 6 months: 87.1 (SD 6.9); 87.4 (7.3); 88.5 (6.7). No significant difference.<br>Nausea and vomiting: 4; 2; 1, urinary retention: 3; 1; 2.
		General anaesthesia	Ultrasound guided FNB 5g/l ropivacaine 20ml plus 0.1mg epinephrine	Ultrasound guided FNB 5g/l ropivacaine 20 ml plus 0.1mg epinephrine and SNB 5g/l ropivacaine 20ml plus 0.1mg epinephrine	
LIA no corticosteroid vs No LIA/ placebo					
Wylde et al. 2015 [33]<br>UK	Primary unilateral TKR for osteoarthritis	FNB with nerve stimulator and/ or ultrasound guidance (20ml 0.25% bupivacaine). Spinal or general anaesthetic. Intra-operative analgesia provided by titration of i.v. fentanyl initially and morphine if necessary. 1g intravenous			6 and 12 months<br>24;19 at 12 months (including those who did not receive treatment)

For peer review only - http://bmjopen.bmj.com/site/about/guidelines.xhtml

2009-2012<br>1 centre	157; 159 (143; 137 received treatment)<br>Mean 69.5 (SD 9.4); 68.7 (7.9)<br>52%; 54%	paracetamol 30 minutes before the end of operation. Immediately post-operative 400mg oral ibuprofen.<br>PCA with morphine 1mg/ml, 1 mg bolus dose and a 5-minute lock-out. If necessary morphine bolus up to 0.2mg/kg as rescue analgesia. During hospital stay, visit from pain specialist nurse. Oral or i.v. paracetamol every 6 hours and ibuprofen 400mg every 8 hours. When PCA no longer needed, oral codeine phosphate 30-60mg every 6 hours, tramadol 50-100mg every 6 hours and oramorph 10-20mg as rescue analgesia.		Low risk of bias<br>At 12 months WOMAC pain score (0-100) in LIA group median 90 (IQR 30), Control 85 (35); ITT-CC linear regression coefficient 3.83 (95%CI -0.83, 8.49), p=0.107. At 6 months WOMAC pain score ITT-CC linear regression coefficient 4.10 (95%CI -0.22, 8.43), p=0.063. Mean differences lower than MCID of 8-9[34].<br>Superficial and deep wound infection rate in LIA group 3.2% and 1.9% in control group, p=0.500. No differences in serious adverse events between groups
		60ml intra-operative LIA with 0.25% bupivacaine and 1/200,000 adrenaline injected into the posterior capsule, medial and lateral capsule, fascia and muscle, and subcutaneous tissues.	No treatment other than standard care	
Williams et al. 2013[39]<br>Canada<br>Before 2013<br>1 centre, 2 surgeons	Primary unilateral TKR for osteoarthritis<br>35; 32 (26; 25 received treatment)<br>Mean 66 (SD 9.7); 67 (12.5)<br>58%; 60%	Sedation with i.v. midazolam and propofol. Intraoperative LIA loading dose of 20ml 0.25% bupivacaine/ epinephrine injection, 10ml into medial and lateral subcutaneous tissue around the incision and 10ml intra-articular after closure. Infiltrate delivered by pain pump into lateral recess of intra-articular space. Spinal anaesthetic with 10-15 mg of 0.75% or 0.5% plain bupivacaine and 20µg fentanyl.<br>Postoperative morphine PCA. 7.5mg i.v ketorolac preoperatively plus 15mg every 6 hours postoperatively for 48 hours, then oral ketorolac 10mg every 6 hours for 2 days. Gabapentin 600mg given preoperatively plus 300mg twice daily for 48 hours postoperatively. Oxycodone 10mg twice daily for 48 hours postoperative. Oral paracetamol 650mg every 4 hours for 72 hours.		6 and 12 months<br>3;1 of those who received treatment<br>Low risk of bias<br>Mean VAS pain score at 6 months 1.2 (SD 1.3); 1.2 (1.2). p=0.836. At 12 months 0.9 (1.2); 1.0 (1.1). p=0.767<br>No short-term differences in adverse events except control patients more likely to be drowsy at 48 hrs. Long-term adverse events not reported.
		Infusion of 0.5% bupivacaine at 2ml/hr for 48 hrs	Infusion of saline at 2ml/hr for 48 hrs	
Niemeläinen et al. 2014[35]<br>Finland<br>2011-2012	Primary unilateral TKR for osteoarthritis<br>30; 30 (27; 29 received treatment)	Oral paracetamol 1g given 1 hour before surgery. Spinal anaesthesia with 15mg bupivacaine in 3ml.<br>After surgery oral paracetamol 1g every 6 hours and oral meloxicam (15mg) every 24 hours.<br>PCA with oxycodone 2mg, lock-out time 8 min.		12 months<br>1; 4<br>Low risk of bias<br>No pain measure separate from OKS. Weak evidence of more favourable

For peer review only - http://bmjopen.bmj.com/site/about/guidelines.xhtml

1 hospital	Mean 65 (SD 4.9); 64 (6.7)<br>56%; 48%	Rescue levobupivacaine medication through a lumbar epidural catheter		OKS (0-48) in the LIA group at 12 months, mean difference -2.7 (95% CI -5.48, 0.07). Difference lower than MCID of 4.0[36].<br>Infection: 0; 0. Severe pain treated with epidural analgesia: 0; 3. Nausea: 1; 1
		Intra-operative periarticular LIA of 100ml saline with levobupivacaine (150mg) mixed with ketorolac (30mg) and adrenaline (0.5mg).	Intra-operative periarticular LIA of 100ml saline	
Motififard et al. 2017[37]<br>Iran<br>2014-2015<br>1 hospital	Primary unilateral TKR for osteoarthritis<br>60; 60<br>Mean 66.4 (6.4); 64.5 (6.0)<br>86.0%; 94.3%	Spinal anaesthesia.<br>No FNB or SNB.<br>Pain medication provided as required after surgery: meloxicam (15 mg daily), celecoxib (400 mg daily), acetaminophen (1g every 8 hours), tramadol (50 mg every 8 hours), ketorolac (30 mg slow IV every 8 hours, with a 4-dose max), and morphine (5–10 mg slow IV if needed)		6 months<br>3; 7<br>Low risk of bias<br>No separate pain measure. Weak evidence for improved KSS (0-200) in LIA group at 6 months, mean 115.55 (SD 15.506); 101.40 (16.117). P=0.07. Difference of 14.15 greater than MCID of 12.3[38]. Difference was significant at 6 weeks, p<0.001.<br>No complications related to TKR or LIA. Low back pain (1; 2), stroke (0; 1), CHF (1; 0)
		Peri-articular injection, 15 minutes before incision, of 100ml saline containing 50 mg bupivacaine hydrochloride 0.5%, 1 ml morphine sulphate 10 mg/ml, 300 µg epinephrine (1:1000) and 30 mg ketorolac	100ml saline containing 300 µg epinephrine (1:1000)	
McDonald et al. 2016[40]<br>UK<br>2010-2011<br>1 hospital	Primary unilateral TKR for osteoarthritis<br>113; 109 received common spinal anaesthesia (121; 121 randomised)<br>Median 68 (IQR 62, 72); 67 (62, 73)<br>59%; 55%	Oral premedication with 10-20mg temazepam, 150mg ranatidine, 10mg dexamethasone, 300mg gabapentin, 1g paracetamol.<br>Spinal anaesthesia		12 months<br>9; 11 of those receiving treatments<br>Low risk of bias<br>No separate pain outcome. Mean OKS at 12 months: median 41 (IQR 35, 44); 41 (34;44). P=0.915<br>Suspected infection 2; 1. MI 0; 1. GI bleed 1; 0. renal failure 1; 0. Died 2; 0)
		Intra-articular and subcutaneous infiltration during surgery of 200 ml of 2mg/ml ropivacaine without adrenalin or additives. Catheter inserted, and 20 ml infiltrate injected following wound closure. Further boluses of 40 ml 2 mg/ml ropivacaine via infusion pump 4 hours after leaving theatre and morning of POD1. Two	Epidural PCA with 4 ml of 2.5 mg/ml levobupivacaine introduced at end of surgery. Thereafter self-medication with 2 ml of 1.25 mg/ml bupivacaine with 15 minutes lockout until morning of POD1. Nurse-administered rescue of 4 ml of 2.5 mg/ml levobupivacaine.	

For peer review only - http://bmjopen.bmj.com/site/about/guidelines.xhtml

		additional top ups of 40 ml 2mg/ml ropivacaine were prescribed if required.		
Celecoxib vs placebo				
Meunier et al. 2007[42]<br>Sweden<br>2004-2005<br>1 centre	Elective primary unilateral TKR for osteoarthritis<br>25; 25 (24; 20 received treatment)<br>Mean 68 (SD 6.3); 69 (7.7)<br>71%; 40%	Spinal anaesthesia with bupivacaine 17.5-20mg. i.v. midazolam or propofol sedation if needed. Paracetamol 1 g preoperatively and then with tramadol 50-100 mg 4 times a day during hospital stay. Ketobemidone (2.5-5mg i.v. or subcutaneous) on demand. Paracetamol and tramadol used as required after discharge.		12 months<br>No losses to follow up after surgery reported<br>Low risk of bias<br>No effect of celecoxib on VAS or KOOS pain at 1 year.<br>DVT: 0; 1. Deep infection: 0; 0.
		Oral celecoxib 200mg 1 hour preoperatively and twice daily for 3 weeks	Oral placebo 200mg 1 hour preoperatively and twice daily for 3 weeks	
Ketamine vs placebo				
Perrin and Purcell2009 [96]<br>Australia<br>Before 2009<br>1 centre (pilot study)	Elective unilateral TKR<br>16 (5; 7 completed study per protocol)<br>Mean 65.6 (SD 10.2); 60.3 (11.9)<br>40%; 43%	Intrathecal injection of 15mg bupivacaine and 100µg morphine. General anaesthesia. After surgery 1.5g paracetamol and then 750mg every 4 hours; PCA with morphine 2mg boluses with 10-minute lockout; morphine rescue 2.5mg intravenously as required; and rescue oral ibuprofen 800mg.		6 months<br>3 protocol breaches and 1 patient with uncontrolled pain.<br>High risk of bias due to non-ITT reporting and recruitment difficulties<br>2/5 ketamine group had mild/moderate pain on the WOMAC pain scale at 26 weeks or failed to improve compared with 5/7 controls.<br>1 adverse psycho-mimetic effect not attributed to intervention or control treatment
		Ketamine 0.5mg/kg bolus followed by 4µg/kg/min infusion. Commenced before surgical incision and continued until wound bandaged or syringe empty.	Saline infusion. Commenced before surgical incision and continued until wound bandaged or syringe empty.	
Ketamine vs Nefopam vs placebo				
Aveline et al. 2014[43]<br>France<br>2005<br>1 centre	Elective unilateral TKR for osteoarthritis<br>25; 25; 25<br>Mean 73 (SD 9); 72 (9); 70 (7)<br>67%; 60%; 63%	General anaesthesia induced with 1.5-2mg/kg propofol, 1µ/kg remifentanil and a single bolus of cisatracurium 0.15mg/kg. Remifentanil infusion at 0.15µg/k/min until skin closure. Anaesthesia maintained with sevoflurane 0.9-1.2% with 50% nitrogen in oxygen. 20 mins before skin closure, 0.15mg/kg i.v. morphine bolus and 0.625mg droperidol. PCA with morphine hydrochloride 1 mg i.v. bolus with 7-min lockout. On arrival in recovery room, 3 mg i.v. morphine boluses at 5 minute intervals.		6 and 12 months<br>3; 1; 2<br>Low risk of bias<br>Median DN4 at 12 months: 1 (IQR 1, 2); 1 (0, 1); 2 (1, 3). p=0.02 for difference between ketamine and placebo groups. Number of patients with VAS pain on movement score

For peer review only - http://bmjopen.bmj.com/site/about/guidelines.xhtml

		0.2mg/kg nefopam administered over 20 min before incision; 2mg/ml nefopam continuous infusion at 120µg/kg/hr until end of surgery and 60µg/kg/hr for 48 hours	0.2mg/kg ketamine administered over 20 min before incision; 2mg/ml ketamine continuous infusion at 120µg/kg/hr until end of surgery and 60µg/kg/hr for 48 hours	Saline administered over 20 minutes before incision; saline continuous infusion until second post-operative day	≥40mm at 12 months by group: nefopam (3/22, 13.7%), ketamine (3/24, 12.5%), and placebo group (6/23, 26.1%). Ketamine reduced DN4 pain (P=0.02) compared with placebo. At 12 months only 7/69 patients had DN4≥4 indicative of neuropathic pain.<br>Infection: 0; 0; 0. Revision: 0; 0; 0.
Pregabalin vs placebo					
Buvanendran et al. 2010[44]<br>USA<br>2006-2007<br>Single centre	Primary unilateral TKR for osteoarthritis.<br>120; 120 (9; 2 did not receive post-operative treatment but ITT analysis)<br>Mean 64.0 (SD 8.3); 63.3 (8.9)<br>76%; 70%	Sedation with midazolam and i.v. propofol. Combined spinal-epidural anaesthetic. 1.5ml 0.75% hyperbaric bupivacaine with 25µg injected intrathecally. Catheter inserted for epidural drug administration.<br>LIA 60 ml 0.25% bupivacaine with epinephrine infiltrated into the wound at capsule closure. From completion of surgery until 32-42 hours post-operative, epidural infusion of fentanyl (5µg/ml) and bupivacaine (1mg/ml) initiated using continuous basal infusion of 6ml/hr with epidural PCA bolus doses (maximum 10ml/hr). Patients transitioned to oral opioid (morphine, oxycodone, and hydromorphone) as required. All patients received preoperative oral celecoxib 400mg 1–2 hours before surgery and 200mg twice daily for 3 days in hospital.			6 months<br>7; 5<br>Low risk of bias<br>In the pregabalin group the incidence of neuropathic pain measured using S-LANSS was 0% (0/113) and 5.2% (6/115) in the placebo group (p=0.014).<br>No clinically significant adverse events up to 6 months and no falls. Sedation, confusion and dry mouth more frequent in pregabalin than placebo group on day of surgery and first postoperative day.
		Oral pregabalin 300mg 1–2 h before surgery, 150mg twice daily for the first 10 postoperative days, 75mg twice daily on days 11 and 12, and 50mg twice daily on days 13 and 14	Oral placebo 1–2 h before surgery, twice daily for the first 10 postoperative days, twice daily on days 11 and 12, and twice daily on days 13 and 14		
FNB long duration vs FNB short duration					

For peer review only

For peer review only - http://bmjopen.bmj.com/site/about/guidelines.xhtml

<table>
<tr><td rowspan="2">Ilfeld et al. 2009[97]
USA
2005-2007
2 centres</td><td rowspan="2">Primary unilateral TKR for osteoarthritis
25; 25
Median 66 (IQR 60, 70); 64 (60, 69)
56%; 60%</td><td colspan="2">Femoral catheter inserted using nerve stimulator. FNB with 0.2% ropivacaine infusion (8ml/hr basal; 4 ml patient-controlled bolus; 30-minute lockout) from surgery until a.m. POD1.
1 week oral acetaminophen (975mg every 6 hours), sustained release oral opioid (Oxycontin, 10mg every 12 hours), and either oral aspirin (650mg daily) or celecoxib (200mg every 12 hours). Oral oxycodone 5 mg tablets and/ or i.v. morphine sulfate 2-4 mg for breakthrough pain.</td><td rowspan="2">6 and 12 months
4; 1 lost to follow up
High risk of bias: uneven loss to follow up between groups; muscle weakness resulted in lower dose of infusion on POD1 (10 continuous; 3 saline)
Groups had similar WOMAC pain scores at 6 and 12 months (p>0.05).
MI: 1; 0. PE: 1; 0. Fall: 1; 0. Catheter leak, dislodged: 1; 2</td></tr>
<tr><td>At 6 a.m. POD1, infusion pump replaced with infusion pump with 0.2% ropivacaine.
At 6 p.m. POD2 pump replaced with portable infusion pump (400ml 0.2% ropivacaine). Catheter removed evening of POD4.</td><td>At 6 a.m. POD1, infusion pump replaced but saline substituted.
At 6 p.m. POD2 pump replaced with portable infusion pump (saline). Catheter removed evening of POD4</td></tr>
<tr><td rowspan="2">Ilfeld et al. 2011[98]
USA
2007-2009
2 centres</td><td rowspan="2">Primary cemented TKR for osteoarthritis
40; 40 (39; 38 included in RCT)
Median 61 (IQR 58, 67); 66 (60, 70)
67%; 66%</td><td colspan="2">Femoral catheter inserted using nerve stimulator. FNB with 0.2% ropivacaine infusion (6ml/hr basal; 4ml patient-controlled bolus; 30-min lockout) from surgery until POD1.
1 week oral acetaminophen (975mg every 6 hours), sustained release oral opioid (Oxycontin, 10mg every 12 hours), and either oral aspirin (650mg daily) or celecoxib (200mg every 12 hours). Oral (oxycodone 5mg or 10mg tablets) and/ or i.v. opioids (morphine sulfate 2-4mg) for breakthrough pain.</td><td rowspan="2">12 months
11; 12 incomplete follow up
High risk of bias: 11;12 did not have 4 measures out of 6 up to 12 months; graph suggests WOMAC pain lower pre-intervention in continuous infusion group.
No difference in WOMAC pain scores between randomised groups (p>0.05).
Falls: 4; 0</td></tr>
<tr><td>At 6 a.m. POD2, infusion pump replaced and 0.2% ropivacaine continued.
At 6 p.m. POD2 pump replaced with portable infusion pump (400ml 0.2% ropivacaine). Catheter removed evening of POD4</td><td>At 6 a.m. POD2, infusion pump replaced but saline substituted.
At 6 p.m. POD2 pump replaced with portable infusion pump (saline). Catheter removed evening of POD4</td></tr>
<tr><td>Choy et al. 2011[30]
Korea
2006-2007</td><td>Primary unilateral TKR for osteoarthritis</td><td colspan="2">Spinal anaesthesia. Continuous FNB via catheter until POD3. Catheter inserted with use of nerve stimulator. Analgesia induced with 20ml of 1:1 0.25% bupivacaine and 2% lidocaine with 1:200,000 epinephrine. Continuous</td><td>2 years
4; 3 lost to follow up</td></tr>
</table>

For peer review only - http://bmjopen.bmj.com/site/about/guidelines.xhtml

<table>
<tr><td rowspan="2">1 surgeon</td><td rowspan="2">33; 30 (2 patients received GA and excluded)
Mean 66.7 (SD 10); 67.5 (11)
97%; 93%</td><td colspan="3">infusion with 0.125% bupivacaine 5.0ml/hr. i.v. PCA (butorphanol 4mg, ketorolac 150mg, saline 50ml), programmed to deliver 1 mg bolus (lockout 10 min) with maximum dose 6mg/hr. i.v. paracetamol 2g 4 times/ day and oral ibuprofen 600mg 3 times/ day for breakthrough pain</td><td rowspan="2">Low risk of bias for 2 year outcome measures.
At 2 years, intervention WOMAC pain mean 7.2 (SD 2), control 6.3 (SD 1); p=0.2
Superficial infection: 1; 1</td></tr>
<tr><td colspan="2">Continuous femoral nerve block via catheter continued from POD3 to POD7</td><td>Continuous femoral nerve block discontinued on POD3</td></tr>
<tr><td colspan="6">FNB continuous high concentration vs FNB low concentration vs FNB single</td></tr>
<tr><td rowspan="2">Albrecht et al. 2014[29]
Canada
2009-2011
1 hospital</td><td rowspan="2">Primary unilateral TKR
32; 32; 35
Mean 61 (CI 57, 64); 63 (60, 67);63 (60, 66)
46%; 44%; 52%</td><td colspan="3">Stimulating catheter inserted with ultrasound guidance. Immediately after catheter placement, 10ml mepivacaine 2% was injected through the catheter. SNB using 30 ml ropivacaine 0.2%. Spinal anaesthesia with 2.5 to 3.0 ml isobaric bupivacaine 0.5% and 0.1mg intrathecal morphine.</td><td rowspan="2">12 months
4;0;2 lost to follow up
Low risk of bias.
No separate pain outcome. Mean WOMAC score at 12 months: high concentration FNB 17 (95% CI 7, 27); 22 (14, 30); 18 (8, 27). P=0.68
Falls: 0; 0; 1</td></tr>
<tr><td>Bolus of 20ml ropivacaine 0.2% with epinephrine 1:400,000 into the femoral catheter followed by ropivacaine 0.2% at a rate of 5 ml/hr with patient-controlled boluses of 5ml available every 30minutes.</td><td>Bolus of 20 ml ropivacaine 0.2% with epinephrine 1:400,000 into femoral catheter followed by ropivacaine 0.1% at rate of 10ml/hr with patient-controlled boluses of 10 ml available every 30 minutes.</td><td>Bolus of 30ml ropivacaine 0.375% with epinephrine 1:400,000 into the femoral catheter followed by normal saline at a rate of 1 ml/hr with patient-controlled boluses of 1mL available every 30minutes.</td></tr>
<tr><td colspan="6">FNB continuous vs Psoas compartment block vs FNB continuous and psoas compartment block</td></tr>
<tr><td>Morin et al. 2005[99]
Germany
Before 2005
1 centre</td><td>Elective TKR
30; 30; 30
Median 68 (IQR 62, 74); 71 (63, 74); 65 (53, 73)</td><td colspan="3">Oral pre-medication with 20mg chlorazepate. General anaesthesia with intravenous propofol and 4–8µg/kg i.v. fentanyl and desflurane in N2O. 100mg diclofenac suppository after anaesthesia induction and 2.5g intravenous metamizole before end of surgery. Post-operative 3 daily doses of oral diclofenac 50mg. i.v. PCA</td><td>9–12 months
7; 6; 5
High risk of bias due to large losses to follow up, non-blinded outcome collection, and differences between</td></tr>
</table>

For peer review only - http://bmjopen.bmj.com/site/about/guidelines.xhtml

	50%; 70%; 59%	with piritramide bolus 2mg as needed with lockout interval of 10 mins for 48 hours.			groups in BMI and anaesthetist's opinion of difficulty of catheter placement.<br>No difference between groups in level of pain at the knee joint during past 4 weeks: FNB median 2.5 (IQR 1, 4), FNB and SNB 2 (1, 4), Psoas block 2 (IQR 1, 4), p=0.44<br>No early complications but longer term adverse events not reported.
		Continuous FNB<br>Stimulating catheter used. Initial bolus of prilocaine 1% and ropivacaine 0.75%. 300mg prilocaine 1% (30ml) and 150mg ropivacaine 0.75% (20ml). During first 48hrs post-operative ropivacaine 0.2% infusion 14ml/hr.	Continuous FNB and continuous SNB<br>Stimulating catheter used. Initial bolus of prilocaine 1% and ropivacaine 0.75%. In each catheter: 200mg prilocaine 1% (20ml) and 75mg ropivacaine 0.75% (10ml). During first 48hrs post-operative infusion through each catheter of 0.2% ropivacaine 7ml/hr.	Continuous psoas compartment block<br>Stimulating catheter used. Initial bolus of prilocaine 1% and ropivacaine 0.75%. 300mg prilocaine 1% (30ml) and 150mg ropivacaine 0.75% (20ml). During first 48hrs post-operative ropivacaine 0.2% infusion 14ml/hr.	
ACB continuous vs FNB continuous					
Davidson et al. 2016[100]<br>USA<br>2013-2014<br>2 studies combined from 1 centre	Primary, unilateral TKR or unicompartmental<br>54 (39 TKR, 16 UKR); 56 (41 TKR, 15 UKR)<br>TKR mean 67 (SD 8); 66 (7). UKR 70 (10); 68 (12)	Spinal or general anaesthesia. Intra-operative i.v. fentanyl, hydromorphone, and/or morphine.<br>LIA with 30 ml ropivacaine (0.5%), ketorolac (30 mg), and epinephrine (5 µg/ml).<br>Post-operative: oral acetaminophen (975 mg every 6 hr), celecoxib (200 mg every 12 hr), and sustained release oxycodone (10 mg every 12 hr). For breakthrough pain, infusion pump bolus (4 ml, 30-min lock-out). Rescue opioid titrated to pain severity. 10 ml lidocaine (2%) bolus was given via the perineural catheter for moderate or severe pain.			12 months<br>31; 29<br>High risk of bias due to partial follow up<br>TKR and UKR combined. Pain score (0-10) at 12 months median 0.0 (IQR 0.0, 3.0); 0.5 (0.0, 2.0). P=0.80). Pain score >0: 35%; 32%. P=0.65. No difference at 4 months when follow up more complete (51;

For peer review only

For peer review only - http://bmjopen.bmj.com/site/about/guidelines.xhtml

	TKR 59%; 66%.<br>UKR 47%; 47%	Ultrasound guided ACB. Ropivacaine 0.2% at basal rate of 6 ml/hr, a 4-ml bolus, and a lockout of 30 minutes	Ultrasound guided continuous FNB. Ropivacaine 0.2% at basal rate of 6 ml/hr, a 4-ml bolus, and a lockout of 30 minutes	52) in pain score (p=0.80) or pain score >0 (p=0.48).<br>Falls in hospital: 2; 5
ACB single vs FNB single				
Macrinici et al. 2017[31]<br>USA<br>Before 2017<br>1 centre	Primary unilateral TKR<br>49; 49<br>Mean 67 (SD 8); 67 (8)<br>61%; 63%	Multimodal regimen including NSAIDs, non-opioid analgesics, opioids. LIA 40ml Marcaine 0.25%.<br>All patients received an ultrasound guided needle insertion into ACB and FNB sites.		6 months<br>3; 4 lost to follow up. 6; 3 complications<br>Low risk of bias<br>VAS pain similar between groups at 6 months. No difference in functional outcomes<br>Medical complications: 3; 0. Surgical complication: 0; 1. Temporary foot drop: 3; 2.
		Immediately after surgery, 30ml solution with 100ml Marcaine into ACB site. 30 ml saline into FNB site	Immediately after surgery, 30ml solution with 100ml Marcaine into FNB site. 30 ml saline into ACB site	
FNB continuous vs oral opioid				
Nader et al. 2012[24]<br>USA<br>2007-2008<br>1 surgeon	Elective TKR<br>31; 31<br>Median (IQR) 65 (60, 76); 64 (60, 71)<br>58%; 77%	Before surgery, patients received 1–2mg midazolam as needed. Epidural with 10mg 0.5% isobaric bupivacaine injected intrathecally. Intraoperative sedation with propofol infusion of 25-75mcg/kg/minute. In post-anesthesia recovery area, PCA epidural with basal infusion of 3 ml/hr (1 mg/ml bupivacaine and 10 mg/ml hydromorphone) with patient-activated boluses of 3 ml with a lockout interval of 15 minutes and per hour maximum of 15 ml. Infusion discontinued and epidural catheter removed on morning of POD 1. All subjects received 5 mg warfarin on evening of surgery and 40 mg enoxaparin starting on POD 1		6 and 12 months<br>1; 1 lost to follow up at 12 months<br>Low risk of bias<br>No difference in overall median NRS pain score at 6 months and 12 months: 0 (IQR 0, 1); 0 (0, 1). p=1.0. At 12 months, some evidence favouring hydrocodone for pain ascending/ descending stairs: 1 (0, 2); 0 (0, 0). p=0.01. Also, suggestion of reduced pain in hydrocodone group at night in bed (p=0.06) and sitting/ lying (p=0.07), standing upright (p=0.10). No difference walking on flat surface (p=0.41).<br>Falls in month after surgery: 1;0. Positive joint aspirate: 3; 0. VTE: 0; 4.
		Continuous FNB inserted with use of stimulator. After discontinuation of epidural anaesthesia on the morning of POD1 10mL bolus of ropivacaine 0.25% followed by 5ml/h infusion of 0.1% ropivacaine. On morning of POD 2, ropivacaine infusion	10 mg oral hydrocodone plus 325mg acetaminophen every 4-6 hours administered for pain as needed. Sustained release oxycodone 10mg for 12 hours with oral hydromorphone 2 mg over	

For peer review only

For peer review only - http://bmjopen.bmj.com/site/about/guidelines.xhtml

		discontinued. Femoral catheter removed 24 hours after previous dose of enoxaparin.	4 hours for breakthrough pain	
FNB continuous vs PCA				
Wang et al. 2015[101]<br>China<br>2012-2013<br>3 centres	Elective TKR<br>82; 86<br>No significant differences in age or sex	General anaesthesia with midazolam (0.02-0.04mg/kg), fentanyl (1µg/kg), propofol (1-2mg/kg) and cisatracurium (0.15mg/kg). Anaesthesia maintained with sevoflurane during surgery. Intramuscular injection with 10mg metoclopramide and 2.5mg droperidol 30 minutes before surgery. Post-surgery, celocoxib and parecoxib 40mg for patients with severe pain, and i.v. morphine if needed.		6 and 12 months<br>2; 4 lost to follow up at 12 months<br>Unclear risk of bias: limited reporting of randomisation methods.<br>No differences were observed between groups at 6 or 12 months for any HSS domain including pain.<br>No nerve injuries
		Continuous FNB with ultrasound stimulator. After surgery, 0.2% ropivacaine (20ml) injected through catheter. Then an analgesia pump was attached delivering 0.2% ropivacaine 8ml/hr.	Epidural PCA 0.2% ropivacaine was injected at a rate of 5 ml/hr in a 2ml pulse dose	
Peng et al. 2014[26]<br>China<br>Before 2014<br>1 centre (2 surgical teams with 4 surgeons and 2 anaesthesiologists)	Unilateral TKR<br>140;140<br>Mean: 66.8 (SD 9.4); 68.0 (SD 11.2)<br>73%; 65%	General intravenous and inhalational anaesthesia: midazolam 0.1-0.15mg/kg (etomidate 0.15-0.2mg/kg for patients >65 years), propofol 2.0-2.5mg/kg, sufentanil citrate 0.3-1.0µg/kg, and vecuronium 0.08-0.12mg/kg for induction of anaesthesia. Maintenance with inhalation of 1%-3% sevoflurane and continuous intravenous infusion of remifentanil 7-8µg/kg/hr and propofol 25-75µg/kg/min. After wound closure, 5-10µg intravenous sufentanil and loading dose of PCA injected. i.v. injection of 4mg ondansetron.		6 and 12 months<br>31; 38 at 12 months<br>Low risk of bias<br>Chronic post-operative pain (NRS 1+) in 38.5% of PCA group at 6 months compared with 25.7% in FNB group (p=0.021). No difference at 12 months (p=0.273).<br>Authors only reported short term adverse events associated with use of PCA.
		FNB with ultrasound guidance. Initial dose of 10ml 2% lidocaine and 10ml 1% ropivacaine. 30 minutes before end of operation, catheter connected to PCA pump; patients received loading dose of 5ml of 0.15% ropivacaine followed by infusion of 0.15% ropivacaine at 5ml/hr, with bolus of 5mL	i.v. PCA with tramadol 800mg, flurbiprofen axetil 100mg, and dexamethasone 5mg with saline to a volume of 80ml. Loading dose of 2ml followed by an infusion rate of 1 ml/hr with bolus of 2 ml. Lock time 15min.	

For peer review only - http://bmjopen.bmj.com/site/about/guidelines.xhtml

		and lock time of 30 min. Preoperatively, a loading dose of 30ml was injected for intraoperative analgesia.		
Wu and Wong 2014[25]<br>China<br>2009-2011<br>1 centre	Unilateral elective TKR<br>40; 39 (30; 30 after post randomisation exclusions)<br>Mean 68.8 (SD 6.4); 68.9 (7.5)<br>73%; 73%	Paracetamol, sustained release diclofenate, opioids (codeine or morphine). Spinal anaesthesia<br>Catheter inserted under nerve stimulation and ultrasound guidance. Standardised bolus of 15 mL 0.5% levobupivacaine. Continuous infusion of 8 to 12 mL/h 0.08% levobupivacaine postoperatively until POD 3	Intravenous PCA morphine after the operation	6 months<br>2; 2 not pre- and peri-operative exclusions<br>Low risk of bias<br>No separate pain outcome but improvement of KSS from pre-operative was FNB 48.73 and PCA 44.7 (p=0.513)<br>Including patients not followed up. Deaths: 0; 0. Infection: 1;1. DVT: 2; 3. Shock: 3;2. Transfusion: 2;3. Also from excluded cases. Atrial fibrillation and confusion: 0; 1. PE: 0; 1. Sepsis: 1;0. ICU admission for shock: 1; 0.
FNB and SNB continuous vs epidural PCA				
Anastase et al. 2014[102]<br>Germany<br>2010-2011<br>1 centre	Primary TKR<br>55; 50<br>Mean 68.2 (SD 9.2); 69.7 (SD 8.7)<br>65%; 69%	Premedication with 10 mg oral clorazepate. Spinal anaesthesia with light sedation: 12.5mg 0.5% bupivacaine. Supplemental postoperative analgesia available with i.v. piritramid<br>After spinal anaesthesia installed, SNB and FNB catheters inserted with ultrasound guidance. 5 ml bolus 0.2% ropivacaine. FNB with an hourly rate of 5 ml, bolus administration of 5 ml by the patient and the lock-out interval of 20 mins. SNB 5 ml/h to a maximum of 8 ml/h, 5 ml bolus administered by the patient	Epidural catheter installed at the same time as spinal catheter. 5ml 0.2% ropivacaine and PCA performed through the epidural catheter. Hourly rate 3 ml, bolus administration of 5 ml, and lock-out period of 30 minutes.	6 and 12 months<br>15; 14<br>High risk of bias due to large loss to follow up<br>Pain during previous 4 weeks: 1 no pain, 2 very little, 3 little, 4 moderate, 5 loud, 6 very loud (translation from German). No difference at 6 months p=0.37. At 12 months, FNB/SNB median 2.00 (1.00, 2.00), PCA 2.00 (2.00, 2.00) p=0.004 favouring FNB/SNB.<br>No falls associated with quadriceps weakness. 6 and 12 month adverse events not reported.

For peer review only

For peer review only - http://bmjopen.bmj.com/site/about/guidelines.xhtml

		and lock-out interval of 20 minutes.		
**FNB single vs LIA**				
Fan et al. 2016[27]<br>China<br>2012-2014<br>2 surgeons	Primary TKR<br>80; 80 (78; 79 in analysis)<br>Mean 68.4 (SD 8.8); 67.6 (6.3)<br>79%; 86%	General anaesthesia in all but 1 in each group. After surgery, i.v. morphine, PCA and parecoxib 40mg<br>FNB performed pre-operatively with 20ml ropivacaine 0.5%.<br>After cementing prostheses, 50ml of saline injected into periarticular soft tissue.	Placebo equivalent of FNB with saline<br>After cementing prostheses, 50ml of LIA mixture containing morphine (1ml: 10mg), ropivacaine (10ml: 100mg), and diprospan (1ml: 5mg betamethasone dipropionate and 2mg betamethasone sodium phosphate) injected into periarticular soft tissue.	1 year<br>3 protocol violations<br>Low risk of bias<br>No separate pain outcome. Mean KSS at 1 year similar between groups: 94.2 (SD 2.6); LIA 93.9 (3.1). p=0.51<br>Infection: 0; 0. DVT: 1; 1. Femoral nerve injury: 1; 0.
**FNB single and epidural vs LIA**				
Reinhardt et al. 2014[28]<br>USA<br>2010-2012<br>2 surgeons	Elective unilateral TKR for osteoarthritis<br>51; 51 (49; 45 received allocated intervention)<br>Mean 67.9 (SD 10.9); 66.6 (10.1)<br>59.2%; 57.8%	Spinal anaesthetic (2.5ml 0.5% bupivacaine). Mobic 15mg daily. Oral Perocet or Vicodin as required. Subcutaneous Dilaudid for severe breakthrough pain. Intravenous Toradol.<br>Combined spinal-epidural (500ml hydromorphone 10µg/ml and bupivacaine HCl 0.06%).<br>Single intra-operative FNB injection (30ml 0.25% bupivacaine).<br>Continuous 48-hour epidural infusion (4ml/hr with 4ml per demand dose, locked out every 10 minutes with an hourly limit of 20ml). Epidural infusion weaned to 2ml/hr on POD1 and to 0 ml/hr at 5 p.m. on POD1. Demand dose with	Intra-articular knee catheter placed intraoperatively with continuous 0.2% ropivacaine infusion at 7 ml/hr until POD2.<br>Placebo epidural catheter, no FNB, and postoperative placebo continuous epidural infusion of saline.	1 year<br>0: 0 of patients who received allocated intervention<br>Low risk of bias<br>VAS pain at 1 year similar between groups (noted in text and shown graphically)<br>No wound-related complications or infections. 1 DVT and 1 DVT plus PE in epidural group. Arthrofibrosis: 2; 1

For peer review only - http://bmjopen.bmj.com/site/about/guidelines.xhtml

		lockout parameters continued for 48 hours.<br>Placebo intraarticular knee catheter placed intra-operatively with continuous saline 7ml/hr infusion until POD2.		
LIA with corticosteroid vs LIA with no corticosteroid				
Seah et al. 2011[41]<br>Singapore<br>2004-2005<br>1 hospital	TKR<br>50; 50<br>Mean 65.4; 67.9<br>Sex not stated	General or spinal anaesthesia. Postoperative oral naproxen and PCA (with morphine bolus of 1mg, lock-out time 5 minutes, and maximum dose 8 mg/hr) for 48 hours.		6 months and 2 years<br>No losses to follow up reported<br>Low risk of bias<br>No separate pain outcome but no statistically significant difference in OKS between groups at 2 years<br>Deep infection: 1; 1
		Intraoperative periarticular injection of 0.5ml/kg 1:200,000 epinephrine and 0.5% bupivacaine diluted with 30ml of normal saline. 40mg of corticosteroid (triamcinolone acetonide) was added to half the mixture. The solution with the corticosteroid was injected into the deep tissues. The remaining solution was injected into the skin incision before closure.	Intraoperative periarticular injection of 0.5ml/kg 1:200,000 epinephrine and 0.5% bupivacaine diluted with 30ml of normal saline. Half the mixture was injected into the deep tissues. The remaining solution was injected into the skin incision before closure.	
Yue et al. 2013[103]<br>China<br>2011-2012<br>1 hospital	Unilateral TKR for osteoarthritis<br>36; 36<br>Mean 70.2 (SD 6.4); 69.3 (5.7)<br>89%; 89%	General anaesthesia. PCA (25 mg/100ml morphine: a 1mg bolus, 6 minutes lock-out, and 5mg/hr maximum) for 72 hours after surgery. 5-10mg intramuscular morphine as rescue. Celecoxib pre- and post-operatively		6 and 12 months<br>No loss to follow up reported<br>Unclear risk of bias.<br>No separate pain outcome. No difference in mean KSS between groups at 6 or 12 months<br>No incision infection or tendon rupture complications
		Injections with local anaesthetic agent and adrenaline (0.75% ropivacaine 30ml, 1:1000 adrenaline 0.5ml, and isotonic sodium chloride solution 70ml) plus corticosteroid (1ml betamethasone).	Injections with local anaesthetic agent and adrenaline (0.75% ropivacaine 30ml, 1:1000 adrenaline 0.5ml, and isotonic sodium chloride solution 70ml) with no added corticosteroid.	

For peer review only

<table>
<tr><td></td><td></td><td colspan="2">Another 50ml syringes fluid without corticosteroid was infiltrated into the skin</td><td colspan="2">Another 50ml syringes fluid without corticosteroid was infiltrated into the skin</td><td></td></tr>
<tr><td colspan="7">LIA including ketorolac vs epidural</td></tr>
<tr><td rowspan="2">Spreng et al. 2012[104], Spreng et al. 2010[105]
Norway
2007–2009
1 hospital</td><td rowspan="2">Unilateral, non-cemented TKR with no patella resurfacing
34; 34; 34
66.5 (SD 11.); 67.2 (SD 8.9); 65.8 (SD 10.1)
61%;61%;67%</td><td colspan="4">Premedication with oral paracetamol (1-2g). Spinal anaesthesia with 13-15mg bupivacaine 5mg/ml with 20µg fentanyl. If indicated, up to 10ml/hr 10mg/ml propofol for sedation. Acetaminophen 1g every 6 hours. i.v. PCA morphine for 48 hours after surgery (2mg bolus with 10 minutes lockout time). When PCA stopped, 10mg slow release oxycodone twice daily. 5mg oxycodone as rescue analgesia.</td><td rowspan="2">12 months
13 did not provide complete data
Unclear risk of bias due to limited reporting (long-term outcome only reported as conference abstract).
Perioperative analgesic treatment did not have any significant influence on any KOOS outcomes.
Infection: 0; 0; 1. No long-term adverse events reported</td></tr>
<tr><td>i.v. injection of ketorolac 1ml (30mg/ml) and morphine 5ml (1mg/ml).
Infiltration with ropivacaine 150mg, epinephrine 0.5mg, ketorolac 30mg and morphine 5mg in 150ml saline.
After closure, catheter placed into knee joint and 10ml infiltrate injected. 22-24 hours after surgery, 20ml injection through catheter of ropivacaine 19ml (7.5mg/ml) and ketorolac 1ml (30mg/ml). i.v. injection of</td><td colspan="2">i.v. injection of 6ml saline.
Infiltration with ropivacaine 150mg, epinephrine 0.5mg, ketorolac 30mg and morphine 5mg in 150ml saline.
After closure, catheter placed into knee joint and 10 ml infiltrate injected. 22-24 hours after surgery, 20ml injection through catheter of ropivacaine 19ml (7.5mg/ml) and saline 1ml. i.v. injection of saline 1ml.
Sham epidural catheter.</td><td>Epidural catheter inserted immediately before spinal anaesthesia. When spinal anaesthesia started to wear off, epidural infusion for 48 hrs with 6-10 ml/hr fentanyl 2µg/ml, epinephrine 1µg/ml, bupivacaine 1mg/ml.
No knee infiltrations.
Sham knee catheter with no injections</td></tr>
</table>

For peer review only

For peer review only - http://bmjopen.bmj.com/site/about/guidelines.xhtml

		ketorolac 1ml (30mg/ml). Sham epidural catheter.			
**Spinal with added high dose morphine sulphate vs spinal with added low dose morphine sulphate vs spinal with no morphine sulphate**					
Foadi et al. 2017[106]<br>Germany<br>Before 2017<br>1 centre	TKR or THR for osteoarthritis<br>16; 16; 17<br>Mean 67.63 (SE 2.45); 67.33 (2.87); 63.71 (3.14) 56%; 44%; 65%	3ml spinal anaesthesia with 0.5% bupivacaine<br>Post-operative 1 g metamizole (orally or intravenously) every 4 hours. 5 mg morphine (intravenous or subcutaneous) as rescue medication			6 months<br>"only a few dropouts". >70% questionnaire return rate.<br>Unclear risk of bias due to limited reporting of pilot RCT.<br>No difference in WOMAC pain between groups at 6 months.<br>No adverse events noted
		0.2mg morphine sulphate added to spinal anaesthesia	0.1mg morphine sulphate added to spinal anaesthesia	No morphine sulphate added to spinal anaesthesia	

2. Myofascial trigger point dry needling

**Author**<br>**Country**<br>**Recruitment dates**<br>**Setting**	**Indication**<br>**Number randomised intervention; control**<br>**Age**<br>**% female**	**Common pain management**		**Follow up**<br>**Losses to follow up intervention; control**<br>**Risk of bias issues**<br>**Key results**
		**Group 1 (intervention)**	**Group C (control)**	
Mayoral et al. 2013[107]<br>Spain<br>2007-2008<br>Single centre	TKR for osteoarthritis<br>20; 20<br>Mean 71.7 (SD 6.1); 72.9 (7.9)<br>72.5%	General or spinal anaesthesia		6 months<br>4; 5<br>High risk of bias due to large losses to follow up<br>WOMAC pain at 6 months: mean 3.24 (SD 3.03); 3.13 (2.72). Difference not statistically significant. No difference between groups in VAS pain (p=0.725) or proportion of patients reporting significant VAS pain at 6 months.
		After anaesthesia and surgery started, dry needling applied 20 times to all myofascial trigger points by a trained and experienced physical therapist.	If spinal anaesthesia used, dry needling simulated behind screen	

For peer review only - http://bmjopen.bmj.com/site/about/guidelines.xhtml

				No complications related to the dry needling intervention. Other adverse events not collected.

3. Tourniquet

**Author**<br>**Country**<br>**Recruitment dates**<br>**Setting**	**Indication**<br>**Number randomised intervention; control**<br>**Age**<br>**% female**	**Common blood conservation strategies**		**Follow up**<br>**Losses to follow up intervention; control**<br>**Risk of bias issues**<br>**Key results**
		**Group 1 (intervention)**	**Group C (control)**	
Ejaz et al. 2014[45]<br>Denmark<br>2011-2012<br>1 centre	Primary TKR<br>35; 35 (33; 31 received intervention)<br>Mean 68 (SD 8.0); 68 (7.8)<br>45.5%; 45.2%	Before surgery, oral tranexamic acid (1g). Tranexamic acid (0.5g) 3 hours after surgery and 6 and 12 hours postoperatively.		6 and 12 months<br>0; 0 of those who received intervention<br>Low risk of bias<br>Statistically significant difference in KOOS pain intensity at 2 months favouring TKR without a tourniquet (p < 0.001). Small difference between groups not statistically significant at 6 and 12 months.<br>Small number of adverse events did not suggest extra risk in the group with no tourniquet.
		Appropriately sized thigh tourniquet applied. Limb exsanguination by elevation for 2 minutes and cuff inflated to 250mm Hg.	Appropriately sized thigh tourniquet applied but not inflated. Served as safety device in case of uncontrollable bleeding.	
Liu et al. 2014[46]<br>Australia<br>Before 2014<br>1 surgeon	TKR for osteoarthritis<br>10; 10<br>Mean 67.0; 70.0<br>30%; 10%	PCA. No CPM		6 and 12 months<br>0; 0<br>Low risk of bias<br>No separate pain measure. Total OKS not significantly different at 6 and 12 months<br>Blood transfusions: 3; 0.
		Tourniquet inflated to 300 mmHg before skin incision. Tourniquet deflated after wound closure and dressing.	Tourniquet placed but not inflated	
Mittal et al. 2012[48]<br>Australia<br>2008-2010	Primary unilateral TKR<br>31; 34	Autologous blood re-infused if required		1 year<br>5; 2
		Short duration. Tourniquet set at 300mm Hg inflated	Long-duration. Tourniquet set at 300mm Hg inflated before	

For peer review only - http://bmjopen.bmj.com/site/about/guidelines.xhtml

1 centre	Mean 67.5 (SD 8.9); 66.6 (8.4)<br>81%:74%	prior to cement application and deflated when cement hardened		skin incision and deflated when cement hardened		Low risk of bias. However, RCT terminated early due to increased need for blood transfusion in short duration tourniquet group.<br>No separate pain outcome. Total OKS (0-48) at 52 weeks higher in long-duration group reflecting better recovery than short duration group but not significantly (p=0.12). Mean difference approximately 5 which is greater than MCID of 4[36].<br>Transfusions: 10; 2. Patient reported adverse event: 26; 12
Abdel-Salam and Eyres 1995[108]<br>UK<br>Date not stated<br>1 surgeon	Primary TKR<br>40; 40<br>Mean 72 (range 65-80); 74 (64-82)<br>57.5%; 62.5%	Tourniquet placed around thigh				1 and 2 years<br>0; 0<br>Unclear risk of bias due to limited reporting of methods. No pain measure or PROM<br>Surgeon recorded HSS score at 1 year 90 (78-97); 91 (80-97). Not significantly different at 1 or 2 years.<br>Blood loss similar between groups. Wound infections: 5;0. DVT: 4;0
		Limb exsanguinated for 2 minutes and tourniquet inflated to twice systolic blood pressure		Tourniquet not inflated		
Şükür et al.2016[109]<br>Turkey<br>2015<br>1 surgeon	Primary TKR, women<br>30; 30; 30; 30<br>Mean 67.0 (SD 7.0); 66.9 (8.5); 68.4 (6.9); 68.4 (6.8)<br>100%	Pneumatic tourniquet inflated to 125mm Hg above systolic blood pressure				6 months<br>0;0;0;0<br>High risk of bias. KSS outcome noted in methods but not presented in results.<br>KSS results not reported at 6 months but no significant differences between groups at 3 months.<br>Surgical and wound complications similar between groups. No infections, fractures or instability requiring revision within 6 months
		Knee in 90° flexion and tourniquet deflated during wound closure	Knee in 90° flexion and tourniquet inflated during wound closure	Knee in full extension and tourniquet deflated during wound closure	Knee in full extension and tourniquet inflated during wound closure	
		Blood transfusion if required				3-22 months, mean 12;13 months

For peer review only

For peer review only - http://bmjopen.bmj.com/site/about/guidelines.xhtml

<table>
<tr><td>Zhang et al.2016 [110]
China
2014-2015
1 hospital</td><td>Primary TKR for osteoarthritis
84; 82
Not reported
Not reported</td><td colspan="3">Tourniquet</td><td colspan="3">No tourniquet</td><td>Not clear
High risk of bias. Variable follow up. HSS outcome noted in methods but not presented in results.
HSS not reported.
Transfusion rates similar between groups. At mean follow up of 12 -13 months, patients operated on without a tourniquet had a lower rate of DVT (2.4%) compared with those with a tourniquet (10.7%).</td></tr>
<tr><td rowspan="2">Zhang et al. 2017[49]
China
2008-2011
1 surgeon</td><td rowspan="2">Unilateral cemented KR for osteoarthritis
50; 50; 50
Mean 70.3 (SD 6.6); 71 (10.2); 68.2 (6.8)
54%; 60%; 50%</td><td colspan="6">Tourniquet inflated to 300-337mm Hg. Tranexamic acid not generally used</td><td rowspan="2">6 months
0; 0; 0
Low risk of bias
No separate pain outcome. HSS similar between groups at 6 months (p=0.839).
At 2 weeks DVT: 0; 0; 1. Intramuscular vein thrombosis: 4; 3; 3. Transfusions: 30%; 26%; 10%</td></tr>
<tr><td colspan="2">Tourniquet for entire operation</td><td colspan="2">Tourniquet removed before wound closure</td><td colspan="2">Tourniquet from first bone osteotomy until wound closure</td></tr>
<tr><td rowspan="2">Huang et al. 2017[47]
China
2015
1 centre</td><td rowspan="2">Primary unilateral TKR for osteoarthritis
50; 50
Mean 66.2 (SD 8.3); 65.1 (6.8)
64%; 68%</td><td colspan="6">Tranexamic acid</td><td rowspan="2">6 months
0; 0
Low risk of bias
VAS pain similar between groups at 6 months (p=0.728). Mean HSS score 90.3 (SD 3.2); 91.2 (2.5). P=0.151
DVT: 0; 0. PE: 0; 0. Intramuscular venous thrombosis: 6; 4. Superficial infection: 1; 0. Wound secretion: 6; 0. No significant difference in blood loss between groups.</td></tr>
<tr><td colspan="3">Tourniquet</td><td colspan="3">No tourniquet</td></tr>
</table>

4. Compression bandage

**Author**	**Indication**	**Common treatments**	**Follow up**

For peer review only

Country<br>Recruitment dates<br>Setting	Number randomised intervention; control<br>Age<br>% female	Intervention	Control	Losses to follow up intervention; control<br>Risk of bias issues<br>Key results
Brock et al. 2017[57]<br>UK<br>2013-2014<br>1 hospital	Primary TKR for osteoarthritis<br>25; 25 (24 received intervention)<br>Mean 67.3 (SD 8.2); 69.5 (6.8)<br>66.7%; 64.0%	Hydrocolloid dressing left in place until clips removed on day 10-14<br>Soft inner layer with compressive outer layer bandage. Removed after 24 hours.	Standard bandaging with soft inner layer and crepe bandage outer layer. Removed after 24 hours and cryocuff used.	6 months<br>0; 0 of patients receiving intervention<br>Low risk of bias<br>No separate pain outcome. Mean OKS similar between groups at 6 months: 35.8 (SD 7.7); 34.3 (10.6). P=0.58<br>No infections or thromboembolic events in either group

5. **Blood conservation**

Author<br>Country<br>Recruitment dates<br>Setting	Indication<br>Number randomised intervention; control<br>Age<br>% female	Common blood conservation strategies<br>Intervention	 Control	Follow up<br>Losses to follow up intervention; control<br>Risk of bias issues<br>Key results
Tranexamic acid				
Sa-Ngasoongsong et al. 2011[50]<br>Thailand<br>2008-2009<br>1 hospital	Primary knee osteoarthritis with unilateral primary cemented computer assisted TKR<br>24; 24<br>69.0 (SD 8.2); 69.2 (7.6)<br>91.7%; 75%	Drain and compressive dressing<br>25ml saline solution containing 250mg tranexamic acid injected into knee joint after fascial closure	25ml saline solution injected into knee joint after fascial closure	6 months<br>0; 0<br>Low risk of bias<br>No separate pain score reported but WOMAC overall score mean 18.6 (SD 7.6); 20.8 (6.4). P=0.282<br>Lower peri-operative blood loss in tranexamic acid group and need for blood transfusion, 1/24 compared with 8/24 in control group. No DVT,

For peer review only - http://bmjopen.bmj.com/site/about/guidelines.xhtml

					wound complications or infection reported in either group
Kim et al. 2014[51]<br>Korea<br>2009-2011<br>1 hospital	Primary unilateral TKR<br>90; 90<br>Mean 73.5 (SD 5.5); 71.9 (SD 5.9)<br>88%; 87%	Tourniquet, drain, compressive dressing. Allogenic blood transfusion and intravenous iron and erythropoietin if required			1 year<br>0; 0<br>Low risk of bias<br>WOMAC pain mean 3.2 (2.6); 2.8 (2.3). Difference not statistically significant<br>Lower blood loss and need for allogenic transfusion in tranexamic acid group. No DVT. 1 PE in control group.
		10 mg/kg body weight tranexamic acid in 100 mL of normal saline given as slow intravenous injection 30 min before tourniquet deflation, and the same amount 3 hours later.	No tranexamic acid and no placebo		
Sa-Ngasoongsong et al. 2013[52]<br>Thailand<br>2010-2011<br>1 hospital	Primary unilateral cemented TKR for osteoarthritis<br>45; 45; 45<br>Mean 68.1 (SD 6.2); 67.6 (8.7); 66.2 (7.3)<br>88.9%; 93.3%; 95.6%	Drain and compressive dressing			1 year<br>0; 0; 0<br>Low risk of bias<br>No separate pain outcome but WOMAC mean 14.5 (7.1); 15.1 (6.2); 15.5 (6.6). P=0.42<br>Total blood and Hb loss lower in intervention groups than control. Fewer transfusions in 500mg (0) than 250mg tranexamic acid group (6) and control group (10). 2 DVT in 500mg group. 1 DVT in 250mg group. 1 PE and 3 DVT in control group. No infections.
		25ml saline solution containing 500mg tranexamic acid injected into knee joint after fascial closure via drain tube.	25ml saline solution containing 250mg tranexamic acid injected into knee joint after fascial closure via drain tube.	25ml saline solution injected into knee joint after fascial closure	
Hourlier et al. 2015[54]<br>France<br>2009-2010<br>1 hospital	Primary unilateral TKR<br>52; 54<br>74 (SD 6); 72 (7)<br>62%; 63%	Tourniquet, electrocautery, routine haemostasis, superficial drain. No blood salvage system.			6 months<br>0; 0<br>Low risk of bias.<br>No separate pain score but KSS clinical score mean 90 (SD 6); 90 (13). P=0.90<br>No difference between groups in total blood loss. 1 MUA in single treatment
		10 mg/kg intra-operative tranexamic infusion. After 2 hours, continuous infusion of tranexamic acid 2 mg/kg/hr for 20 hours via electric syringe	single bolus of 30 mg/kg tranexamic acid as an intraoperative infusion. After 2 hours, placebo saline continuous infusion via electric syringe		

For peer review only

				group. No deep infections or revisions.
Huang et al. 2017[47]<br>China<br>2015<br>1 centre	Primary unilateral TKR for osteoarthritis<br>50; 50<br>Mean 66.2 (SD 8.3); 65.8 (6.3)<br>64%; 70%	Tourniquet inflated to 100mm Hg above SBP before incision and deflated after wound closure<br>Intravenous tranexamic acid 20mg/kg before incision and tranexamic acid 10mg/kg at 3, 6, 12 and 24 hours. 1g tranexamic acid in 50ml saline irrigated into wound during operation	No treatment with tranexamic acid	6 months<br>0; 0<br>Low risk of bias<br>VAS pain similar between groups at 6 months (p=0.728). Mean HSS score (0-100) better in tranexamic acid group than controls: 90.3 (SD 3.2); 88.9 (3.0). P<0.001. Mean difference 1.4 lower than MCID of 8.29[53]<br>Greater blood loss in control group than tranexamic group (p<0.001). DVT: 0; 0. PE: 0; 0. Intramuscular venous thrombosis: 6; 3. Superficial infection: 1; 3. Wound secretion: 6; 9.
Thrombin infusion				
Kusuma et al. 2013[55]<br>USA<br>Not stated<br>1 hospital	Primary TKR<br>40; 40<br>Mean 64.6 (SD 10.2); 64.5 (7.3)<br>82.5%; 67.5%	Tourniquet, drain, Esmarch bandage, electrocautery<br>20,000 IU thrombin infusion (1,000 IU/mL) through fascial defect	Closure and drain placement protocol without the thrombin infusion.	1 year (6 months and 2 years also reported)<br>0; 0<br>Low risk of bias<br>No separate pain outcome. KSS mean 95.5; 96.0. p=0.45<br>Lower drop in Hb in thrombin group. Blood transfusion in 4 intervention and 7 control patients. 1 control patient had haematoma. No hospital readmissions
Flexion vs extension				
Napier et al. 2014[56]<br>UK<br>2003-2004<br>1 hospital	Primary TKR<br>90; 90<br>Mean 70.4 (SD 9.9) 71.0 (7.6)<br>74%; 64%	No drains or tranexamic acid<br>Flexion. Operated knee kept in passive flexion (120°) post-operatively for 6 hours using a jig. Wound redressed and placed in flexion over a	Extension. Operated knee kept in full passive extension	1 year<br>5; 1 (12 did not attend follow up)<br>Low risk of bias.<br>No separate pain outcome. OKS mean 20.5 (SD9.0); 22.1 (9.7). P=0.27

For peer review only - http://bmjopen.bmj.com/site/about/guidelines.xhtml

		single pillow until POD1 morning.		1 MI and 1 DVT in each group. 1 haematoma in flexion group. 1 deep infection and 1 extensor muscle weakness in extension group. More transfusions in extension group (p=0.002)
Auto-transfusion of washed blood				
Thomas et al. 2001[111]<br>UK<br>Not stated<br>1 hospital	TKR<br>115; 116<br>Mean 69.3 (range 32-95); 70.0 (40-88)<br>62%; 53%	Allogenic transfusion if Hb fell below 9g/dl<br>Auto-transfusion of wound drainage if volume >125ml post-operative. Blood washed and re-suspended before re-infusion using a centrifugal cell washing machine	Wound drainage discarded	6 months<br>Losses to follow up not reported<br>Unclear risk of bias due to limited details of methods and follow up.<br>No separate pain outcome. No significant difference in EQ-5D between groups.<br>7% of auto-transfusion group required allogenic transfusion compared with 28% in control group. Fewer infections, readmissions and GP visits in auto-transfusion group. No significant differences in other serious adverse events or mortality between groups.

6. Platelet rich plasma

**Author**<br>**Country**<br>**Recruitment dates**<br>**Setting**	**Indication**<br>**Number randomised intervention; control**<br>**Age**<br>**% female**	**Common blood conservation strategies**		**Follow up**<br>**Losses to follow up intervention; control**<br>**Risk of bias issues**<br>**Key results**
		**Group 1 (intervention)**	**Group C (control)**	
Aggarwal et al. 2014[112]	Primary unilateral surgery or first surgery of staged	Tourniquet. No tranexamic acid or suction drain. Blood transfusion if necessary due to intraoperative blood loss or postoperative haemoglobin <8g/dl.		6 months<br>No losses to follow up reported

For peer review only - http://bmjopen.bmj.com/site/about/guidelines.xhtml

India<br>2010-2011<br>1 surgeon	bilateral TKR for osteoarthritis<br>7; 14<br>Mean 56.43 (SD 7.59); 53.79 (9.75)<br>Sex not stated	8 ml PRP, prepared from patient's blood. Calcium chloride for activation given in a separate syringe in 4:1 ratio. PRP and calcium chloride injected into the posterior recess, gutters and capsule, and repaired extensor mechanism and prepatellar fat.	No treatment	High risk of bias due to unexplained differences in numbers of patients in randomised groups.<br>No separate pain outcome. WOMAC total at 6 months PRP mean 7.14 (SE 0.69), controls 7.86 (1.23), p=0.173<br>PRP group had lower fall in haemoglobin and need for blood transfusion

7. Cryotherapy

**Author**<br>**Country**<br>**Recruitment dates**<br>**Setting**	**Indication**<br>**Number randomised intervention; control**<br>**Age**<br>**% female**	**Common treatment**		**Follow up**<br>**Losses to follow up intervention; control**<br>**Risk of bias issues**<br>**Key results**
		**Group 1 (intervention)**	**Group 1 (intervention)**	
Wang 2017[113]<br>China<br>2013-2015	Unilateral TKR for osteoarthritis<br>53; 53<br>Mean 65.23 (SD 5.41); 64.97(5.36)<br>62.3%; 58.5%	CPM for 2 weeks		6 months<br>0; 0<br>Unclear risk of bias due to limited reporting<br>No separate pain outcome. At 6 months 87% of cryotherapy patients had excellent or good knee function compared with 69% of controls (p=0.032).<br>No adverse events reported in either group during functional training
		Compression cold therapy for 48 hours	No compression cold therapy	

For peer review only - http://bmjopen.bmj.com/site/about/guidelines.xhtml

8. Denusomab

**Author**<br>**Country**<br>**Recruitment dates**<br>**Setting**	**Indication**<br>**Number randomised intervention; control**<br>**Age**<br>**% female**	**Common treatment**		**Follow up**<br>**Losses to follow up intervention; control**<br>**Risk of bias issues**<br>**Key results**
		**Group 1 (intervention)**	**Group 1 (intervention)**	
Ledin et al. 2017[59]<br>Sweden<br>2012-2014<br>2 centres	Elective cemented primary TKR for osteoarthritis<br>25; 25<br>Mean 66 (SD 6.3); 64 (5.5)<br>60%; 60%			12, 24 months<br>0; 2<br>Low risk of bias<br>No significant differences in KOOS pain or other KOOS domains between groups 12 12 or 24 months<br>No suspected unexpected adverse reactions in either group
		Injection of 60mg denusomab 1 day after surgery and after 6 months	Injection of placebo 1 day after surgery and after 6 months	

9. Continuous passive motion

**Author**<br>**Country**<br>**Recruitment dates**<br>**Setting**	**Indication**<br>**Number randomised intervention; control**<br>**Age**<br>**% female**	**Common treatment**			**Follow up**<br>**Losses to follow up intervention; control**<br>**Risk of bias issues**<br>**Key results**
		**Group 1 (intervention)**	**Group 2 (intervention)**	**Group C (control)**	
Leach et al. 2006[114]<br>UK<br>Before 2005<br>1 hospital	Cruciate retaining rotating platform TKR for osteoarthritis<br>85 overall<br>Mean 71.2 (range 53-84); 72.9 (52-89)	Physiotherapy protocol from POD1 including slider board exercises to improve ROM and quadriceps strengthening exercises.			6 and 12 months<br>25 patients lost to follow up<br>High risk of bias due to large loss to follow up and use of date of birth randomisation<br>No difference in mean VAS pain at 1 year, CPM 0.6; control 0.9. p=0.49
		CPM commenced on first postoperative day set at a range 0–30 and used for 1 hour twice per day. Each day,	No CPM		

For peer review only - http://bmjopen.bmj.com/site/about/guidelines.xhtml

	50%; 54%	range was increased by 10° with discharge at POD 5-7.			Adverse events not reported
Sahin et al. 2006[115]<br>Turkey<br>Before 2006<br>1 hospital	Primary unilateral TKR for osteoarthritis<br>15; 16<br>Mean 61 (SD 6.0); 61.6 (7.5)<br>86%; 86%	Standard physiotherapy			6 months<br>3 lost to follow up<br>Unclear risk of bias as patients were followed up by treating physician.<br>Mean difference in VAS pain 0.1/10 slightly favouring no CPM group (95% CI -0.8, 0.9; P=0.87)<br>Adverse events not known
		From POD 1, CPM 2.5 hours 2x/day. Initially 0-40° flexion and increased by 10° each day until POD 7	No CPM		
Pope et al. 1997[116]<br>Australia<br>1988-1999<br>1 hospital	Primary TKR<br>62 (70 knees). Authors excluded those not followed up so groups were 18; 20; 19<br>Mean 72.5 (95% CI 64.4, 74.98); 72.7 (70.4, 75.0); 69.4 (64.4, 74.98)<br>64.7%; 50%; 72.2%	Physiotherapy commenced on postoperative day 1			6 and 12 months<br>8 patients (12 knees) excluding 1 death<br>High risk of bias due to losses to follow up and limited reporting of methods<br>No separate pain outcome. However, "pain disability" contributed up to 50 points out of a total of 70-point functional score (70 best outcome). No difference between groups in functional score: CPM 0-40 median 56 (range 20, 70); CPM 0-70 52 (10, 70); no CPM 52 (25, 70). p=0.80<br>CPM groups had greater blood loss than controls, p=0.008). 1 manipulation under anaesthesia in no CPM group, 2 revisions due to patellar dislocation in the 0-40 CPM group, 1 PE death in the 0-70 CPM group.
		Patients had an initial CPM range of 0-40° increased by 10° twice, on day after surgery and day 2, so that 0-60° flexion achieved before removal of machine at 48 hours	Patients had an initial CPM range of 0-70° increased by 10° twice, on day after surgery and day 2, so that 0-90° flexion achieved before removal of machine at 48 hours	Knee placed in an extension splint in the recovery room	
Beaupré etal. 2001[117]	Primary TKR	Standardised exercise during hospital admission which included a slider board session.			6 months

For peer review only - http://bmjopen.bmj.com/site/about/guidelines.xhtml

Canada<br>1997-1998<br>1 hospital	40; 40; 40<br>Mean 68 (SD 9); 68 (9); 69 (8)<br>52.5%; 50%; 30%	3 sessions (2 hours) with CPM machine per day from POD2. Range increased from starting range 0-30 degrees as tolerated.	Minimum of two 10-minute slider board therapy sessions per day in addition to one in the standardised exercise. Active knee flexion and extension in sitting and lying positions performed independently as tolerated.	No intervention further than standardised exercise.	6; 8; 6<br>Unclear risk of bias due to losses to follow up<br>Mean WOMAC pain at 6 months: 76 (15); 85 (15); 79 (16). No difference over time between groups, p=0.62.<br>Long-term adverse events. Need for MUA: 1; 1; 0. DVT: 0; 1; 0. Cellulitis: 0; 0; 1. Infection 0; 0; 1.
Kumar et al. 1996[118]<br>USA<br>Before 1996<br>1 hospital	Primary TKR for osteoarthritis<br>40 (46 knees); 33 (37)<br>Mean 69 (range 52-86); 68 (42-88)<br>58%; 67%	Standard physiotherapy<br>CPM from POD 0. Initially 10 hours/ day 0-90° until discharge		No CPM. Passive range of movement ("drop and dangle") to 90° 2x/ day initially for 20 minutes, later 30-45 minutes.	6 months<br>15; 13 lost to follow up<br>High risk of bias due to large losses to follow up<br>No separate pain outcome. KSS CPM 82.7; Drop and dangle 80.7. p=0.78<br>Haematoma 3;1. Closed manipulation 1;3. DVT 0;0. PE 0;1
Worland et al. 1998[119]<br>USA<br>1996<br>1 hospital	TKR for osteoarthritis. 91 patients (114 knees randomised. After post-randomisation exclusions: 37 (49 knees); 43 (54 knees)<br>Mean 70.2 (range 44-84)<br>66.25%	CPM and physiotherapy during hospital admission<br>At home after discharge, CPM machine 3 hours per day on replaced knee for l0 days.		Physical therapist home visit 1 hour three times per week for 2 weeks	6 months<br>11 patients (11 knees)<br>Unclear risk of bias due to post-operative exclusions not reported separately for groups and limited reporting of methods.<br>No separate pain outcome. At 6 months, mean HSS score CPM 95.3 (SD 2.8); physiotherapy 95.7 (3.0). P=0.49.<br>Adverse events not reported.
MacDonald et al. 2000[120]	TKR for osteoarthritis	Active ROM, passive ROM exercises, mobilised as tolerated using walker or crutches.			6 and 12 months

For peer review only - http://bmjopen.bmj.com/site/about/guidelines.xhtml

Canada<br>Before 2000<br>1 hospital	40; 40; 40<br>Age and sex not reported	CPM commenced POD 0. Initially 0-50 degrees. Provided for 18-24 hour/ day. Increased by 10 degrees/ hour as tolerated. Continued until POD 1	CPM commenced POD 0. Initially 70-110 degrees. Provided for 18-24 hr/ day. Not increased.<br>Continued until POD 1	No CPM	Not reported<br>Unclear risk of bias due to limited and selective reporting.<br>No separate pain outcome. No statistical differences between groups for KSS at 6 and 12 months.<br>Adverse events not reported
Bennett et al. 2005[61]<br>Australia<br>1997-2000<br>1 hospital	Primary unilateral TKR for osteoarthritis<br>47; 48; 52<br>70.7; 71.4; 71.7<br>72.3%; 64.6%; 67.3%	Standard in hospital physiotherapy programme			12 months<br>1 patient excluded due to inability to achieve 90° flexion<br>Low risk of bias<br>No separate pain outcome. No significant difference in KSS between groups at 1 year.<br>No difference in wound healing between groups
		Standard CPM from 0° to 40° for 2x3 hours on POD 1 increased by 10° per day until POD 6. Extension splint applied overnight	Early flexion CPM commenced in recovery room from 90° to 50° knee flexion. Increased gradually to CPM 90° to 0° for 2x3 hours in day 4-6.	No CPM	
Ersözlü et al. 2009[60]<br>Turkey<br>2003-2004	TKR for osteoarthritis<br>30; 30; 30<br>Mean 65 (range 54-73); 61 (49-80); 62 (52-78)<br>66%; 55%; 57%	Conventional physical therapy			2 years<br>1; 1; 2<br>Low risk of bias<br>No separate pain outcome. KSS scores 98; 95; 92. No significant difference between groups p=0.67.<br>Infection 0; 0; 1. Arrhythmia 0; 1; 0. No difference in complications between groups
		CPM set at 30-40° from POD1. Increased as tolerated to POD7. 1 hour CPM 3x/day.	CPM set at 60-70° from POD3. Increased by 10°/ day to POD7. 1 hour CPM 3x/day.	No CPM	

10. Electrical stimulation

Author<br>Country<br>Recruitment dates<br>Setting	Indication<br>Number randomised intervention; control	Common rehabilitation strategies		Common rehabilitation strategies
		Intervention	Intervention	

For peer review only - http://bmjopen.bmj.com/site/about/guidelines.xhtml

For peer review only

	**Age**<br>**% female**			
Avramidis et al. 2011[62]<br>Greece<br>2005-2006<br>1 hospital	Unilateral TKR for osteoarthritis<br>38; 38<br>Mean 70.54 (SD 4.68); 70.66 (3.73)<br>80%; 82.9%	Standard physiotherapy for 6 weeks. No CPM		1 year<br>3 (intervention intolerance); 3<br>Low risk of bias<br>Improved SF-36 bodily pain at 1 year in intervention group compared with control, mean 92 (SD 10.57); 79.48 (12.72). P<0.001. Difference of 12.52 close to MCID of 16.86[63]. No difference in OKS or American KSS<br>Adverse events not reported
		Transcutaneous electric muscle stimulation of the vastus medialis muscle from POD2 2x/ day for 2 hours for 6 weeks.	No intervention	
Stevens-Lapsley et al. 2012[121]<br>USA<br>2006-2010<br>1 hospital	Primary unilateral TKR<br>35; 31<br>Mean 66.2 (SD 9.1); 64.8 (7.7)<br>57.1%; 51.6%	Standard inpatient rehabilitation, home and outpatient physical therapy		6 months and 1 year<br>5; 6<br>Unclear risk of bias due to baseline differences in WOMAC<br>No difference in resting pain (points) at 1 year intervention mean 0.6 (SD 1.4); control 0.4 (1.5). Also similar at 6 months. Mean WOMAC total score better at 1 year in intervention group compared with control, 5.7 (5.9); 10.0 (12.2) and at 6 months. However, probably explained by baseline differences. Authors state no differences for change in WOMAC.<br>DVT 1; 0. Unspecified complication 1; 0. Infection 0; 2. Revision 0; 1
		Neuromuscular electrical stimulation commenced on POD2 for 6 weeks 2x/ day.	No intervention	
		2 sessions of ROM exercise		6 months

For peer review only

<table>
<tr><td>Levine et al. 2013[122]
USA
Before 2013
1 surgeon</td><td>Elective TKR for osteoarthritis
35; 35
Mean 68.1; 65.1
76%; 62%</td><td>Neuromuscular electrical stimulation commenced 14 days pre-operatively until 1 day before surgery. Recommenced at POD1 for 60 days. After hospital discharge no direct contact with a physical therapist</td><td>Formal physical therapy programme with progressive resistive exercises and strengthening in hospital and after discharge supervised by physical therapist.</td><td>5; 9
Unclear risk of bias due to large uneven losses to follow up
KSS pain favoured intervention at 6 months but not significantly 79.08 (SD 10.97); 75.5 (14.77); 95%CI for difference -3.78, 10.93. Similar for WOMAC total score, 95%CI for difference -3.19, 14.81.
Confusion 2; 0</td></tr>
<tr><td rowspan="2">Moretti et al. 2012[64]
Italy
2008-2010
1 hospital</td><td rowspan="2">TKR for osteoarthritis
15; 15
Mean 70.0 (SD 10.6); 70.5 (8.1)
Not reported</td><td colspan="2">Rehabilitation protocol including CPM</td><td rowspan="2">6 and 12 months
No losses to follow up
Low risk of bias
Mean VAS pain (10-point scale) lower at 12 months in intervention group compared with control, 0.5 (SD 1.3); 3.6 (3.9). p< 0.05. Mean difference of 2.1 (10-point scale) greater than MCID of 16.1 (100-point scale)[65]
Difference also at 6 months.
More swelling of the knee in intervention patients than controls, statistically significant at 1 and 2 months</td></tr>
<tr><td>Pulsed electromagnetic fields (I-ONE therapy) from POD7, 4 hours/ day for 60 days</td><td>No intervention</td></tr>
<tr><td rowspan="2">Adravanti et al. 2014[123]
Italy
1 hospital</td><td rowspan="2">Primary TKR for osteoarthrosis
16; 17
Mean 66 (SD 13); 73 (5)
62.5%; 52.9%</td><td colspan="2">Standard rehabilitation protocol: active and passive mobilisation</td><td rowspan="2">6 months
4; 3
High risk of bias: small study, proportionately high losses to follow up
At 6 months, mean VAS pain in intervention group lower than in controls (p<0.05). At 3 years, 1/14 intervention patients and 4/12 controls reported severe pain</td></tr>
<tr><td>Pulsed electromagnetic fields (I-ONE therapy) by POD7 for 4 hours/ day for 60 days</td><td>No intervention</td></tr>
</table>

For peer review only - http://bmjopen.bmj.com/site/about/guidelines.xhtml

				No difference between groups in swelling at 6 months.

11. Rehabilitation

Author<br>Country<br>Recruitment dates<br>Setting	Indication<br>Number randomised intervention; control<br>Age<br>% female	Common rehabilitation strategies		Follow up<br>Losses to follow up intervention; control<br>Risk of bias issues<br>Key results
		**Intervention**	**Control**	
Walking guidance and training				
Li et al. 2017[66]<br>China<br>2015-2016<br>1 hospital	Unilateral TKR<br>43; 43<br>Mean 76.33 (SD 5.28); 78.47 (5.50)<br>55.8%; 51.2%	Before TKR, general guidance on joint activities, quadriceps muscle strength, use of aids, diet guidance, correct walking methods and precautions.<br>Knee passive flexion and extension to 90° and quadriceps muscle strength training commenced on POD 1. POD 3-7, straight leg raising exercises. 2 weeks after replacement, increased joint activities and muscle strength training, centre of gravity transfer training, limb weight training, and walking training.		6 months<br>0; 0<br>Low risk of bias<br>Mean VAS pain at 6 months: 0.51 (SD 0.74); 2.83 (0.88) favouring walking intervention group, p<0.01. Difference of 2.42 (10 point scale) greater than the MCID of 16.1 (100-point scale)[65]. HSS scores at 6 months favoured intervention, p<0.01.<br>No infection, allergic reaction or immune reaction in either group. Intervention not associated with swelling, pain, prosthesis loosening, thrombosis, or delayed wound healing
		Standing, weight and balance exercises from POD 1. From POD 2, walking guidance and training.	No additional rehabilitation	
Aquatic therapy				
Liebs et al. 2012[68]<br>Germany<br>2003-2004<br>4 hospitals	Elective unilateral TKR for osteoarthritis<br>87;98	Continuous passive motion machines daily after removal of suction drains. Programme of daily physiotherapy: range of motion activities; exercises for improvement of muscle tension, venous return, balance, coordination and gait; and instruction in activities of daily living.		6, 12 and 24 months<br>13.8%; 19.4% excluding deaths and unexplained reasons<br>Low risk of bias

For peer review only - http://bmjopen.bmj.com/site/about/guidelines.xhtml

	Mean 68.5 (SD 8.6); 70.9 (7.5)<br>70.1%;73.5%	Aquatic therapy for 30 minutes 3 times a week up to postoperative week 5. Pool exercises aimed at training of proprioception, coordination and strengthening with aid of float cuffs, training kickboards and bar floats.			WOMAC pain at 12 months: early aquatic mean 13.2 (SD 15.0); late aquatic 17.4 (22.4) p=0.22. No difference at 6 and 24 months.
		Aquatic therapy beginning on the 6th postoperative day with the wound covered with a waterproof adhesive dressing.	Aquatic therapy as pool exercise after the completion of wound healing on the 14th postoperative day		5 early aquatic therapy patients and 1 late aquatic therapy patient readmitted to hospital within 3 months. 2 early aquatic patients and 1 late aquatic patient readmission directly or indirectly related to the intervention.
Rahmann et al. 2009[124]<br>Australia<br>2003-2005<br>1 hospital with 2 surgeons	Primary TKR or THR for osteoarthritis (50% TKR)<br>18;19;17 (11 had been excluded post-randomisation due to complications in hospital<br>Mean 69.4 (SD 6.5); 69.0 (8.9); 70.4 (9.2)<br>44.4%; 63.2%; 70.6%	Standard ward-based physiotherapy until day 3. 1 ward physiotherapy treatment per day. Surgical wounds covered with an occlusive, waterproof dressing. 40 mins/ day.			6 months<br>4;2;0 for combined THR and TKR<br>Unclear risk of bias as TKR patients more likely to receive ward-based control intervention. THR and TKR analysed together<br>No difference in overall WOMAC outcome at 6 months in THR and TKR patients combined between aquatic at fast pace and ward-based (p=0.929) and aquatic at 2 paces (p=0.872).<br>No adverse events reported after intervention commenced.
		From day 4, 1 to 1 individual physiotherapy. Aquatic physiotherapy programme to maximize function and strength. 40 mins/ day. Fast pace metronome 80-88 bpm	From day 4, 1 to 1 individual physiotherapy. Water exercise programme with general exercises not targeted at specific functional retraining in the aquatic environment. Slow pace metronome 50-58 bpm	From day 4, 1 to 1 individual ward-based physiotherapy. 40 mins/ day	
Supported early discharge					
Mahomed et al. 2008[69]<br>Canada<br>2000-2002<br>2 centres	Unilateral TKR or THR for osteoarthritis (approximately 50% TKR)<br>119;115<br>68	Inpatient physiotherapy			12 months<br>No losses to follow up<br>Low risk of bias (analysis by actual treatment received showed similar results)<br>WOMAC pain at 12 months marginally favoured home-based rehabilitation mean 87 (SD 16); 83
		Discharged home when able to independently transfer supine to sitting and sitting to standing, walk 30 metres and climb stairs if necessary. Physiotherapist home visit within 48 hours and subsequent management	Transfer to independent rehabilitation centre for 14 day stay.		

For peer review only

For peer review only - http://bmjopen.bmj.com/site/about/guidelines.xhtml

	About 67% women	along a multidisciplinary clinical pathway (4-16 visits). Then outpatient physiotherapy or self-directed programme.		SD (20), p=0.08 but this was not statistically significant. Mean difference of 4 less than MCID of 8-9[34]. Results did not differ between TKR and THR patients.<br>Similar rates of dislocation, DVT and readmissions between groups. 2% inpatient group developed infections compared with 0 in home group
Hill et al. 2000[125]<br>UK<br>1997-1998<br>1 centre	Unilateral, primary TKR, irrespective of diagnosis or concomitant disease<br>70 randomised, with 32;28 eligible for trial at day 5	Care pathway for medical, nursing and physiotherapy care from admission until day 5		1 year<br>No losses to follow up reported after commencement of intervention<br>Unclear risk of bias due to limited reporting of methods.<br>No pain outcome or patient reported outcome. Control group had better mean KSS scores, but this did not reach statistical significance at 1 year or earlier.<br>1;1 serious infection, other wound infections 1;6, painful joints 9;4, other minor complications similar between groups
		Outreach team domiciliary visit prior to admission with assessment of home environment. At days 5–7, patients assessed to ensure discharge safe. Outreach team visit on day of discharge with further visits as required. 1+ physiotherapist visit linked with nurses to monitor knee performance. Discharge when skin clips removed, wound healed and specialist orthopaedic assistance not required, usually day 10–12	Inpatient care until removal of skin clips and wound healing.	
Flexion or extension during knee closure				
Wang et al. 2014[67]<br>China<br>2009-2010<br>1 centre	Primary TKR for osteoarthritis<br>40; 40<br>Mean 68.34 (SD 7.09), 67.87 (6.47)<br>17.5%; 22.5%	No patellar replacement or lateral retinacular release		6 months<br>No losses to follow up<br>Low risk of bias<br>Mean VAS pain in flexion group 1.15 (SD 0.73); extension group 1.12 (0.68), p=0.64<br>No wound complications, patella fracture or infection requiring surgery in either group
		Articular capsule, soft tissue and skin enclosed in 90° flexion which was maintained for 1-2 min after wound closure.	Wound closure performed in full extension	

For peer review only - http://bmjopen.bmj.com/site/about/guidelines.xhtml

12. Wound management

Author<br>Country<br>Recruitment dates<br>Setting	Indication<br>Number randomised intervention; control<br>Age<br>% female	Common wound management strategies		Follow up<br>Losses to follow up intervention; control<br>Risk of bias issues<br>Key results
		Intervention	Control	
Kong et al. 2014[58]<br>South Korea<br>2011<br>1 surgeon	Primary cemented unilateral TKR for osteoarthritis<br>50; 50<br>Mean 69.0 (SD 7.7); 68.0 (4.8)<br>89.6%; 87.5%	Skin staples removed on day 10 and wound closure strip applied for 5 days		6 and 12 months<br>2; 2 lost to follow up<br>Low risk of bias<br>At 12 months, VAS pain in silicone gel group mean 2.50 (SD 1.16); control 2.92 (1.90). P=0.201. No difference at 6 months, p=0.886.<br>No wound dehiscence or infection associated with application of silicone gel or petroleum
		After removal of wound closure strip, patients managed operation scars with application of silicone gel for 1 month after stitches removed	After removal of wound closure strip, patients managed operation scars with application of petroleum gel for 1 month after stitches removed	

13. Anabolic steroids

Author<br>Country<br>Recruitment dates<br>Setting	Indication<br>Number randomised intervention; control<br>Age<br>% female	Common rehabilitation strategies		Follow up<br>Losses to follow up intervention; control<br>Risk of bias issues<br>Key results
		Intervention	Control	
Hohmann et al. 2010[70]<br>Australia<br>Before 2010	TKR for osteoarthritis<br>5; 5	Cold compression and CPM		6, 9 and 12 months<br>0; 0 lost to follow up<br>Low risk of bias (but small feasibility study)
		On day 5, intramuscular injection of 50 mg Nandrolone decanoate solution. Patients visited	On day 5, intramuscular injection of saline. Patients visited every 2 weeks and	

For peer review only - http://bmjopen.bmj.com/site/about/guidelines.xhtml

1 surgeon	Mean 66.2 (range 58, 72); 65.2 (59, 72)<br>20%; 40%	every 2 weeks and injections continued for 6 months.	injections continued for 6 months.	No separate pain outcome. KSS at 12 months in intervention group mean 91.4 (SD 3.5); control 81.2 (SD 7.1). p=0.03. Difference also at 6 months (p=0.04), marginal at 9 months (p=0.06). Difference in means at 12 months of 10.2 close to MCID of 12.3[38].<br>Intervention group had smaller decrease in bone mineral density at 6 months than controls but not significant

14. Guided imagery

**Author**<br>**Country**<br>**Recruitment dates**<br>**Setting**	**Indication**<br>**Number randomised intervention; control**<br>**Age**<br>**% female**	**Common rehabilitation strategies**		**Follow up**<br>**Losses to follow up intervention; control**<br>**Risk of bias issues**<br>**Key results**
		**Intervention**	**Control**	
Jacobson et al. 2016[126]<br>USA<br>2011-2012<br>1 surgeon	Unilateral TKR<br>42; 40 (41; 39 received treatment)<br>Mean 65.0 SD 8.6)<br>62.2%	Participants listened to a 19-21-minute CD each day for 2 weeks before and 3 weeks after surgery. Content covered concerns and hopes about TKR with aim to facilitate mind–body connections to promote optimal TKR outcomes.	Participants listened to a 17-21-minute CD each day for 2 weeks before and 3 weeks after surgery. Content comprised poetry, short stories and essays	6 months<br>12; 10 of patients receiving treatments<br>High risk of bias due to large losses to follow up<br>Mean WOMAC pain 2.7 (SD 3.1); 3.5 (SD 3.3). P<0.001<br>Adverse events not reported

CD compact disc; CPM Continuous passive motion; DN4 Douleur Neuropathique 4; FNB Femoral nerve block; HSS Hospital for Special Surgery; i.v. intravenous; KOOS Knee injury and Osteoarthritis Outcome Score; KSS Knee Society Score; LIA local infiltration analgesia; NRS Numerical rating scale; OKS Oxford Knee Score; ONB obturator nerve block; PCA Patient controlled analgesia; PNB psoas nerve block; SF-36 Short Form

For peer review only - http://bmjopen.bmj.com/site/about/guidelines.xhtml

36 Health Survey; S-LANSS Leeds assessment of Neuropathic Symptoms and Signs Pain Scale; SNB Sciatic nerve block; TKR Total knee replacement; VAS Visual analogue scale; VTE Venous thromboembolism; WOMAC Western Ontario and McMaster Universities Osteoarthritis Index.

ITT, ITT CC, POD, MI, PE

For peer review only

For peer review only - http://bmjopen.bmj.com/site/about/guidelines.xhtml

Supplementary material. Risk of bias assessment

Study	Random sequence generation	Allocation concealment	Blinding of participants and personnel	Blind outcome assessment	Incomplete outcome data	Selective reporting	Other bias	Summary
Pain management								
Albrecht et al. 2014[29]	Computer generated	Anaesthetist blind to allocation	Physiotherapists, surgeons, research assistants collecting data, and members of the Acute Pain Service were kept blinded to group allocation.	Physiotherapists, surgeons, research assistants collecting data, and members of the Acute Pain Service were kept blinded to group allocation.	ITT analysis low losses to follow up	No but not checked protocol	Study was terminated early with 61% of planned recruitment completed due to change in standard anaesthesia at hospital	Low
Anastase et al. 2014[102]	Method of the Ulm Institute of Statistics	Method of the Ulm Institute of Statistics	No	No	15:14 lost to follow up	No but not checked protocol	ASA comorbidities differed between groups	High
Aveline et al. 2014[43]	Computer generated	opaque sealed envelopes	Blinded syringes prepared by nurse not involved in study	Yes	Low losses to follow up	Consistent with short term follow up paper	No	Low
Bergeron et al. 2009[94]	Blocks of different sizes according to list preprepared by study epidemiologist	Not described	Anaesthetist not blind. Patients blind	Nurse observers collecting data blind to allocation	32/59 lost to follow up	No but not checked protocol	No	High
Buvanendran et al. 2010[44]	computer generated	Yes, physicians and nurses blind	Yes	Yes	ITT	No but not checked protocol	No	Low

For peer review only - http://bmjopen.bmj.com/site/about/guidelines.xhtml

Choy et al. 2011[30]	Computer generated	sealed envelope	No, the catheter was removed at either day 3 or 7	Patient reported outcome. Other outcomes by blinded independent physician	Low losses to follow up	Protocol not checked but reasonable range of outcomes	No	Low
Davidson et al. 2016[100]	Computer generated	Sealed opaque envelopes	Subjects and investigators were not masked to treatment group	Subjects and investigators were not masked to treatment group. PROM	31; 29 lost to follow up	None apparent but protocol not checked	Combined data from 2 RCTs	High
Fan et al. 2016[27]	No details	sealed opaque envelopes	Patients and assessors blind to randomisation	Patients and assessors blind to randomisation	2% protocol violation	No but not checked protocol	No	Low
Foadi et al. 2017[106]	Computer generated	Not described	Not described	Patient reported outcome	>70% questionnaire return	None apparent but protocol not checked	Described as pilot study	Unclear
Gao et al. 2017[23]	Random number table	Not described	Blind to patients	Blind to observers	2; 1; 0	None apparent but protocol not checked	Groups similar at baseline	Low
Ilfeld et al. 2009[97]	Computer generated	Investigators, patients, and all clinical staff were unaware of treatment group assignments	Investigators, patients, and all clinical staff were unaware of treatment group assignments	Investigators, patients, and all clinical staff were unaware of treatment group assignments	4:1 lost to follow up	No but not checked protocol	Basal infusion halved on POD1 in 10 intervention patients compared with 3 controls	High
Ilfeld et al. 2011[98]	Computer generated tables	Solutions prepared by investigational pharmacist	Yes. Intervention and control solutions indistinguishable	Patient reported outcomes. Staff masked to treatment group	11;12 did not have 4 measures out	Protocol not checked	WOMAC and WOMAC domain scores	High

For peer review only - http://bmjopen.bmj.com/site/about/guidelines.xhtml

				assignment performed all measures and assessments	of 6 up to 12 months	but seems reasonable	somewhat lower pre-intervention in extended infusion group. Authors report change scores	
Macrinici et al. 2017[31]	Computer generated	Staff performing injections blind	Anaesthesiologist, surgeons, patients and physical therapists blind to allocation	Yes	3; 4 lost to follow up. 6; 3 complications	None apparent but protocol not checked	Groups similar at baseline	Low
McDonald et al. 2016[40]	Computerised blocked	Study coordinator independent of care and surgery	Patients blind. Surgeon aware of study	Outcome assessment blind to allocation	1; 4 unexplained	None apparent, protocol not checked	Groups similar at baseline	Low
Meunier et al. 2007[42]	Computer generated	Sealed envelope	Randomisation code broken after 1 year	Yes	ITT reported except for 12 month pain outcome	No but not checked protocol	M/F ratio differed	Low
Morin et al. 2005[99]	Allocated randomly	Sealed envelope		Observers not blinded	Per protocol analysis	No but not checked protocol	Difference between groups in anesthetist's opinion of difficulty of catheter placement. BMI differed between groups	High
Motififard et al. 2017[37]	Computer generated	Study coordinator independent of care and surgery	Patients blind. Surgeon aware of study	Outcome assessment blind to allocation	3; 7 (1; 4 unexplained)	None apparent, protocol not checked	Groups similar at baseline	Low

For peer review only - http://bmjopen.bmj.com/site/about/guidelines.xhtml

Nader et al. 2012[24]	Computer generated	Opaque envelope	No	Patient reported outcome	1:1 lost to follow up	Protocol not checked but reasonable range of outcomes	FNB group somewhat higher BMI	Low
Niemeläinen et al. 2014[35]	No details	Opaque sealed envelopes	Only research nurse preparing infiltrate aware of randomisation. All other personnel unaware until after 1 year follow up	Only research nurse preparing infiltrate aware of randomisation. All other personnel unaware until after 1 year follow up	All patients who received intervention completed follow up	No but not checked protocol	No	Low
Peng et al. 2014[26]	Computer generated	Not possible	Not possible	Patient reported outcome	31:38 lost at 12 months but ITT and per-protocol analysis	No but not checked protocol	No	Low
Perrin and Purcell 2009[96]	No details	Sealed syringe code stored in pharmacy department	yes	Yes	4 failed to complete protocol	No but not checked protocol	Pilot investigation. High risk of bias due to recruitment difficulties leading to small trial	High
Reinhardt et al. 2014[28]	Computer generated	Maintained by pharmacy department for blinding	Patients blind to intervention	Blinded research assistant and partially physical therapist	0 reported lost to follow up of those who received interventions	No but not checked protocol	No	Low
Seah et al. 2011[41]	Randomisation tables	Sealed envelopes. Anaesthetist and surgeon blind before opening of	Blinding of patients not stated	Blind outcome assessors and PROMs	No losses to follow up reported	No but not checked protocol	No	Low

For peer review only - http://bmjopen.bmj.com/site/about/guidelines.xhtml

		sealed envelope						
Shum et al. 2009[95]	No details	No details	Anaesthetist performing the blocks was not involved in the postoperative follow-up and data collection	Patient reported	14% and 20%	No but not checked protocol	Mean patient weight lower in no FNB group. More favourable mean OKS in no FNB group. Two groups combined for 2 year outcome but not for earlier	High
Spreng et al. 2012[104], Spreng et al. 2010[105]	Hospital pharmacy	Epidural catheter or sham set-up taped along the back of the patient and connected to an infusion pump covered in an opaque bag. Also sham knee catheter	Patients blind	Blind outcome assessment	13%	Limited reporting in conference abstract	Conference abstract only so limited information additional to early follow up paper	Unclear
Wang et al. 2015[101]	No details	No details	Not stated	Not stated	2:4 lost to follow up	No but not checked protocol	No	Unclear
Wegener et al. 2013[32]	No details	Opaque envelope	Patients, surgeons and researchers not blind to intervention	Patients not blinded	2:7:5 lost to follow up	No. Protocol checked	no	Low
Widmer et al. 2012[22]	Coded envelope	Coded envelope	Except for anaesthetist and surgeon	Both the investigators and patients were blinded	None reported as incomplete	No but protocol not checked	No	Low

For peer review only - http://bmjopen.bmj.com/site/about/guidelines.xhtml

Williams et al. 2013[39]	Computer generated	Not stated	Patients and assessors blind	Patients and assessors blind	3:1 of those who received treatment	No but not checked protocol	No	Low
Wu and Wong 2014[25]	Computer	Sealed envelopes	No	No	Available cases	No but not checked protocol	No	Low
Wylde et al. 2015[33]	Trials unit	Trials unit	Surgeon and anaesthetist not blind to allocation, Patients blind	Patients and research nurses blind to allocation	ITT with imputed data	No as per protocol	No	Low
Yue et al. 2013[103]	No details	No details	Surgeons and patients were double-blinded to the injection administered	surgeons and patients were double-blinded to the injection administered	Losses to follow up not reported	Limited reporting	No	Unclear
Myofascial trigger point dry needling								
Mayoral et al. 2013[107]	Computerised	Not described	Patient and other researchers apart from physical therapist blind	Patient outcomes	4: 5 loss to follow up	None apparent but protocol not checked	No	High
Tourniquet								
Abdel-Salam and Eyres 1995[108]	Card system	Not described	No	No	No losses to follow up	None apparent but protocol not checked	No	Unclear
Ejaz et al. 2014[45]	Block randomised	Sealed envelopes	Patients unaware	PROM	No losses to follow up of those who received treatments	None apparent but protocol not checked	No	Low
Huang et al. 2017[47]	Computer generated	Sealed opaque envelopes	Surgeons not blind. Patients blind to allocation	Data collector blind to allocation	No losses to follow up reported	None apparent but protocol	Groups similar at baseline	Low

						not checked		
Liu et al. 2014[46]	Excel	Not described	Patients blind	PROM	No losses	None apparent but protocol not checked.	No	Low
Mittal et al. 2012[48]	Computer generated	Sealed opaque envelopes	Patient blind	Outcome assessors blind. PROM	5:2	None apparent but protocol not checked	Study stopped because of high risk of transfusion in short tourniquet duration group	Low
Şükür et al.2016[109]	Computer generated	Not described	Possibly patients	Outcome assessors blind	No losses to follow up	KSS outcome noted in methods but not presented in results	No	High
Zhang et al. 2017[49]	Excel	Randomisation by blinded researcher.	Patients and nurses on ward blind	Not clear	No losses reported	None apparent but protocol not checked	Groups similar at baseline	Low
Zhang et al.2016[110]	Randomly allocated	Not clear	Not clear	Not clear	Not clear	HSS outcome noted in methods but not presented in results	No	High
Compression bandage								

For peer review only - http://bmjopen.bmj.com/site/about/guidelines.xhtml

Brock et al. 2017[57]	Web-based	Not specified	Not possible	No but PROMs	4; 2 of those receiving intervention	None apparent but protocol not checked	Groups similar at baseline	Low
Blood conservation								
Hourlier et al. 2015[54]	Computer generated	Opaque envelopes	Anaethsetist, surgeon and patient blind to treatment allocation	Assessors blind	No missing data	None apparent but protocol not checked	No	Low
	Computer generated	Sealed opaque envelopes	Surgeons not blind. Patients blind to allocation	Data collector blind to allocation	No losses to follow up reported	None apparent but protocol not checked	Groups similar at baseline	Low
Kim et al. 2014[51]	Computer generated	Not stated	patients blind to allocation	Clinical investigator blind to allocation	No missing data	None apparent but protocol not checked	No	Low
Kusuma et al. 2013[55]	Computer generated	Sealed envelopes	Surgeon and patient blind	Outcome assessor blind	No missing data	None apparent but protocol not checked	No	Low
Napier et al. 2014[56]	Computer generated	Sealed envelopes	Unlikely	Not stated but PROM	low losses to follow up	None apparent but protocol not checked	No	Low
Sa-Ngasoongsong et al. 2011[50]	Computer generated	Sealed envelopes	Surgeon and patient blind	Outcome assessor blind	No missing data	None apparent but	No	Low

For peer review only - http://bmjopen.bmj.com/site/about/guidelines.xhtml

						protocol not checked		
Sa-Ngasoongsong et al. 2013[52]	Computer generated	Sealed envelopes	Surgeon and patient blind	Outcome assessor blind	No missing data	None apparent but protocol not checked	Some difference between groups in pre-operative Hb	Low
Thomas et al. 2001[111]	Not described	not stated	Not reported	Not reported but PROM	Not reported but ITT	None apparent but protocol not checked	No	Unclear
Platelet rich plasma								
Aggarwal et al. 2014[112]	Not described	Opaque envelopes	Patients blind	Patients and examiners blind	No losses to follow up reported	None apparent but protocol not checked	Odd numbers in groups from randomisation	High
Cryotherapy								
Wang 2017[113]	No details	No details	No details	No details	No losses to follow up	None apparent but protocol not checked	Groups similar at baseline	Unclear
Denusomab								
Ledin et al. 2017[59]	Randomisation list produced by the study monitor	Syringes prepared independently	Investigators and patients blind	Unblinding was done after all the data had been locked	0; 2	None apparent but protocol not checked	No	Low
Continuous passive motion								
Beaupré etal. 2001[117]	Computer generated	Sealed envelopes	No	Researcher unaware and PROMs	6:8:6. Results carried	No	4 controls; 1 SB	Unclear

For peer review only - http://bmjopen.bmj.com/site/about/guidelines.xhtml

					forward for missing data		reassigned to CPM	
Bennett et al. 2005[61]	Block	Not stated	Operating surgeon blind. Patient not	Independent assessor blind	1 not included in analyses as not able to achieve 90 degree flexion	No	No	Low
Ersözlü et al. 2009[60]	Divided into groups by random selection	Not described	No	Surgeon score	A diabetic patient from the control group was excluded because of a superficial wound infection, a patient with a cardiac problem in group II due to dysrhythmia, and two patients due to insufficient follow-up.	Not apparent	No differences baseline	Unclear
Kumar et al. 1996[118]	Random number generator	Not stated	No	Not described	Large loss to follow up	Not all data clearly reported	No	High
Leach et al. 2006[114]	Allocation by date of birth	No	No	Blinded evaluation	Large loss to follow up	No	No	High
MacDonald et al. 2000[120]	Computer generated	Allocation concealed	No	Not described	Not reported	Yes, not all outcomes reported in full	No	Unclear
Pope et al. 1997[116]	Not described	Not described	Not described	Not described	No separate reporting. 8 patients (12 knees)	None apparent but protocol	No	High

					excluding 1 death	not checked		
Sahin et al. 2006[115]	Not described	Not stated	No	Followed up by treating physician	Low loss to follow up	No	No	Unclear
Worland et al. 1998[119]	Not described	Not described	No	Researcher blind	Not reported separately	None apparent but protocol not checked	No	Unclear
Electrical stimulation								
Adravanti et al. 2014[123]	Computer generated	Not described	Research assistant not involved in patient assessment	Principal investigator and all physicians in charge of clinical controls were blinded to patient allocation	78% retained at 6 months	Not apparent but protocol not checked	No	High
Avramidis et al. 2011[62]	Computer generated	Not described	No	Independent assessors blind	3 (intolerance of intervention); 3	Not apparent	Baseline similar	Low
Levine et al. 2013[122]	Drawing papers from hat	Not described	No	Not described. WOMAC PROM	5:9 for KSS pain and WOMAC	Not apparent but protocol not checked	No	Unclear
Moretti et al. 2012[64]	Computer generated	Not described	Physicians, as well as medical assessors, were blinded to the allocation of patients in the study groups	Physicians, as well as medical assessors, were blinded to the allocation of patients in the study groups	No losses reported	Not apparent but protocol not checked	No	Low
Stevens-Lapsley et al. 2012[121]	Stratified	Concealed	No	no but standardised scripts used	5; 6	Not apparent but protocol	WOMAC, BMI unequal at baseline	Unclear

For peer review only - http://bmjopen.bmj.com/site/about/guidelines.xhtml

						not checked		
Rehabilitation								
Hill et al. 2000[125]	Not described	Not stated	Not possible	Not described	No losses to follow up after initial 23222	No but protocol not checked	No	Unclear
Li et al. 2017[66]	Random number table	Not stated	Not possible	Not described but PROM	No losses to follow up	No but protocol not checked	Groups similar at baseline	Low
Liebs et al. 2012[68]	Computer generated	Allocation concealed	Not possible	No but PROM	Low losses to follow up (<20% if deaths and other explained reasons not counted)	No but protocol not checked	No	Low
Mahomed et al. 2008[69]	Block randomisation	Not stated	Not possible	PROM	No loss	No but protocol not checked	ITT gave similar results to analysis according to actual discharge destination (20 inpatient group received home based)	Low
Rahmann et al. 2009[124]	Not described	Sealed numbered envelopes	Not possible	Assessor blind to intervention. Patient reported outcome	Low losses to follow up	No but protocol not checked	TKR patients more likely to receive ward-based control intervention. THR and TKR analysed together	Unclear
Wang et al. 2014[67]	Computer generated	Surgeons did not participate	Surgery was performed by the	Postoperative evaluation was	No loss to follow up	None apparent	No baseline differences	Low

For peer review only - http://bmjopen.bmj.com/site/about/guidelines.xhtml

		in pre-operative grouping	physicians who did not participate in the preoperative grouping and postoperative evaluation	conducted by the physicians who were unaware of the grouping.		but protocol not checked		
Wound management								
Kong et al. 2014[58]	Not described	Not described	Placebo used	Patient outcome	Low loss to follow up	None apparent but protocol not checked	Similar at baseline	Low
Anabolic steroids								
Hohmann et al. 2010[70]	Internet based	Not reported	Placebo trial	Double-blind design minimized systemic error and eliminated observer and experimenter's bias	0 loss to follow up	None apparent	None apparent but small study	Low
Guided imagery								
Jacobson et al. 2016[126]	Permuted blocks	Opaque CD holders	Personnel yes, participants no	Yes	12; 10 of patients receiving treatment	None apparent but protocol not checked	No	High

For peer review only - http://bmjopen.bmj.com/site/about/guidelines.xhtml

References to all RCTs of peri-operative interventions with long-term pain or score follow up, irrespective of risk of bias assessment (numbering consistent with main article, also includes studies reporting minimal clinically important differences)

22. Widmer BJ, Scholes CJ, Pattullo GG, et al. Is femoral nerve block necessary during total knee arthroplasty?: a randomized controlled trial. J Arthroplasty 2012;27:1800-5.
23. Gao WL, Li H, Liu BQ, et al. Analgesic effect of femoral and sciatic nerve block under multimodal analgesia in total knee arthroplasty. Chinese Journal of Tissue Engineering Research 2017;21:2966-72.
24. Nader A, Kendall MC, Wixson RL, et al. A randomized trial of epidural analgesia followed by continuous femoral analgesia compared with oral opioid analgesia on short- and long-term functional recovery after total knee replacement. Pain Med 2012;13:937-47.
25. Wu JWS, Wong YC. Elective unilateral total knee replacement using continuous femoral nerve blockade versus conventional patient-controlled analgesia: perioperative patient management based on a multidisciplinary pathway. Hong Kong Med J 2014;20:45-51.
26. Peng L, Ren L, Qin P, et al. Continuous femoral nerve block versus intravenous patient controlled analgesia for knee mobility and long-term pain in patients receiving total knee replacement: a randomized controlled trial. Evid Based Complement Alternat Med 2014;2014:569107.
27. Fan L, Yu X, Zan P, et al. Comparison of local infiltration analgesia with femoral nerve block for total knee arthroplasty: A prospective, randomized clinical trial. J Arthroplasty 2016;31:1361-5.
28. Reinhardt KR, Duggal S, Umunna BP, et al. Intraarticular analgesia versus epidural plus femoral nerve block after TKA: A randomized, double-blind trial. Clin Orthop Relat Res 2014;472:1400-8.
29. Albrecht E, Morfey D, Chan V, et al. Single-injection or continuous femoral nerve block for total knee arthroplasty? Clin Orthop Relat Res 2014;472:1384-93.
30. Choy WS, Lee SK, Kim KJ, et al. Two continuous femoral nerve block strategies after TKA. Knee Surg Sports Traumatol Arthrosc 2011;19:1901-8.
31. Macrinici GI, Murphy C, Christman L, et al. Prospective, double-blind, randomized study to evaluate single-injection adductor canal nerve block versus femoral nerve block: postoperative functional outcomes after total knee arthroplasty. Reg Anesth Pain Med 2017;42:10-6.
32. Wegener JT, van Ooij B, van Dijk CN, et al. Long-term pain and functional disability after total knee arthroplasty with and without single-injection or continuous sciatic nerve block in addition to continuous femoral nerve block: a prospective, 1-year follow-up of a randomized controlled trial. Reg Anesth Pain Med 2013;38:58-63.
33. Wylde V, Lenguerrand E, Gooberman-Hill R, et al. Effect of local anaesthetic infiltration on chronic postsurgical pain after total hip and knee replacement: the APEX randomised controlled trials. Pain 2015;156:1161-70.

For peer review only - http://bmjopen.bmj.com/site/about/guidelines.xhtml

34. Ehrich EW, Davies GM, Watson DJ, et al. Minimal perceptible clinical improvement with the Western Ontario and McMaster Universities osteoarthritis index questionnaire and global assessments in patients with osteoarthritis. J Rheumatol 2000;27:2635-41.
35. Niemeläinen M, Kalliovalkama J, Aho A, et al. Single periarticular local infiltration analgesia reduces opiate consumption until 48 hours after total knee arthroplasty. A randomized placebo-controlled trial involving 56 patients. Acta Orthop 2014;85:614-9.
36. Beard DJ, Harris K, Dawson J, et al. Meaningful changes for the Oxford hip and knee scores after joint replacement surgery. J Clin Epidemiol 2015;68:73-9.
37. Motififard M, Omidian A, Badiei S. Pre-emptive injection of peri-articular-multimodal drug for post-operative pain management in total knee arthroplasty: a double-blind randomized clinical trial. Int Orthop 2017;41:939-47.
38. Lee WC, Kwan YH, Chong HC, et al. The minimal clinically important difference for Knee Society Clinical Rating System after total knee arthroplasty for primary osteoarthritis. Knee Surg Sports Traumatol Arthrosc 2017;25:3354-9.
39. Williams D, Petruccelli D, Paul J, et al. Continuous infusion of bupivacaine following total knee arthroplasty: a randomized control trial pilot study. J Arthroplasty 2013;28:479-84.
40. McDonald DA, Deakin AH, Ellis BM, et al. The technique of delivery of peri-operative analgesia does not affect the rehabilitation or outcomes following total knee arthroplasty. Bone Joint J 2016;98-B:1189-96.
41. Seah VWT, Chin PL, Chia SL, et al. Single-dose periarticular steroid infiltration for pain management in total knee arthroplasty: a prospective, double-blind, randomised controlled trial. Singapore Med J 2011;52:19-23.
42. Meunier A, Lisander B, Good L. Effects of celecoxib on blood loss, pain, and recovery of function after total knee replacement: a randomized placebo-controlled trial. Acta Orthop 2007;78:661-7.
43. Aveline C, Roux AL, Hetet HL, et al. Pain and recovery after total knee arthroplasty: a 12-month follow-up after a prospective randomized study evaluating Nefopam and Ketamine for early rehabilitation. Clin J Pain 2014;30:749-54.
44. Buvanendran A, Kroin JS, Della Valle CJ, et al. Perioperative oral pregabalin reduces chronic pain after total knee arthroplasty: a prospective, randomized, controlled trial. Anesth Analg 2010;110:199-207.
45. Ejaz A, Laursen AC, Kappel A, et al. Faster recovery without the use of a tourniquet in total knee arthroplasty. Acta Orthop 2014;85:422-6.
46. Liu D, Graham D, Gillies K, et al. Effects of tourniquet use on quadriceps function and pain in total knee arthroplasty. Knee Surg Relat Res 2014;26:207-13.
47. Huang Z, Xie X, Li L, et al. Intravenous and topical tranexamic acid alone are superior to tourniquet use for primary total knee arthroplasty: A prospective, randomized controlled trial. J Bone Joint Surg (Am) 2017;99:2053-61.
48. Mittal R, Ko V, Adie S, et al. Tourniquet application only during cement fixation in total knee arthroplasty: a double-blind, randomized controlled trial. ANZ J Surg 2012;82:428-33.
49. Zhang Y, Li D, Liu P, et al. Effects of different methods of using pneumatic tourniquet in patients undergoing total knee arthroplasty: a randomized control trial. Ir Jr Med Sci 2017;186:953-9.

For peer review only

For peer review only - http://bmjopen.bmj.com/site/about/guidelines.xhtml

50. Sa-Ngasoongsong P, Channoom T, Kawinwonggowit V, et al. Postoperative blood loss reduction in computer-assisted surgery total knee replacement by low dose intra-articular tranexamic acid injection together with 2-hour clamp drain: a prospective triple-blinded randomized controlled trial. Orthop Rev 2011;3:e12.
51. Kim TK, Chang CB, Kang YG, et al. Clinical value of tranexamic acid in unilateral and simultaneous bilateral TKAs under a contemporary blood-saving protocol: a randomized controlled trial. Knee Surg Sports Traumatol Arthrosc 2014;22:1870-8.
52. Sa-Ngasoongsong P, Wongsak S, Chanplakorn P, et al. Efficacy of low-dose intra-articular tranexamic acid in total knee replacement; a prospective triple-blinded randomized controlled trial. BMC Musculoskelet Disord 2013;14:340.
53. Singh JA, Schleck C, Harmsen WS, et al. Validation of the hospital for special surgery knee questionnaire: Convergent validity, responsiveness and sensitivity to change. ACR/ARHP; San Diego, California2013. p. 1926.
54. Hourlier H, Reina N, Fennema P. Single dose intravenous tranexamic acid as effective as continuous infusion in primary total knee arthroplasty: a randomised clinical trial. Arch Orthop Trauma Surg 2015;135:465-71.
55. Kusuma SK, Sheridan KC, Wasielewski RC. Use of bovine thrombin to reduce blood loss in primary total knee arthroplasty: a controlled randomized trial. J Arthroplasty 2013;28:1278-81.
56. Napier RJ, Bennett D, McConway J, et al. The influence of immediate knee flexion on blood loss and other parameters following total knee replacement. Bone Joint J 2014;96-B:201-9.
57. Brock TM, Sprowson AP, Muller S, et al. STICKS study - Short-sTretch Inelastic Compression bandage in Knee Swelling following total knee arthroplasty - a feasibility study. Trials 2017;18:6.
58. Kong CG, Kim GH, Kim DW, et al. The effect of topical scar treatment on postoperative scar pain and pruritus after total knee arthroplasty. Arch Orthop Trauma Surg 2014;134:555-9.
59. Ledin H, Good L, Aspenberg P. Denosumab reduces early migration in total knee replacement. Acta Orthop 2017;88:255-8.
60. Ersözlü S, Sahin O, Ozgür A, et al. The effects of two different continuous passive motion protocols on knee range of motion after total knee arthroplasty: a prospective analysis. Acta Orthopaedica et Traumatologica Turcica 2009;43:412-8.
61. Bennett LA, Brearley SC, Hart JAL, et al. A comparison of 2 continuous passive motion protocols after total knee arthroplasty: a controlled and randomized study. J Arthroplasty 2005;20:225-33.
62. Avramidis K, Karachalios T, Popotonasios K, et al. Does electric stimulation of the vastus medialis muscle influence rehabilitation after total knee replacement? Orthopedics 2011;34:175.
63. Escobar A, Quintana JM, Bilbao A, et al. Responsiveness and clinically important differences for the WOMAC and SF-36 after total knee replacement. Osteoarthritis Cart 2007;15:273-80.
64. Moretti B, Notarnicola A, Moretti L, et al. I-ONE therapy in patients undergoing total knee arthroplasty: a prospective, randomized and controlled study. BMC Musculoskelet Disord 2012;13:88.

For peer review only - http://bmjopen.bmj.com/site/about/guidelines.xhtml

65. Danoff JR, Goel R, Sutton R, et al. How much pain is significant? Defining the minimal clinically important difference for the visual analog scale for pain after total joint arthroplasty. J Arthroplasty 2018;33:S71-S5.
66. Li L, Wang Z, Yin MH, et al. Effect of early gait training on the functional rehabilitation after total knee arthroplasty. Chinese Journal of Tissue Engineering Research 2017;21:4288-93.
67. Wang S, Xia J, Wei Y, et al. Effect of the knee position during wound closure after total knee arthroplasty on early knee function recovery. J Orthop Surg Res 2014;9:79.
68. Liebs T, Herzberg W, Rüther W, et al. Multicenter randomized controlled trial comparing early versus late aquatic therapy after total hip or knee arthroplasty. Arch Phys Med Rehabil 2012;93:192-9.
69. Mahomed NN, Davis AM, Hawker G, et al. Inpatient compared with home-based rehabilitation following primary unilateral total hip or knee replacement: a randomized controlled trial. J Bone Joint Surg (Am) 2008;90:1673-80.
70. Hohmann E, Tetsworth K, Hohmann S, et al. Anabolic steroids after total knee arthroplasty. A double blinded prospective pilot study. J Orthop Surg Res 2010;5:93.
94. Bergeron SG, Kardash KJ, Huk OL, et al. Functional outcome of femoral versus obturator nerve block after total knee arthroplasty. Clin Orthop Relat R 2009;467:1458-62.
95. Shum CF, Lo NN, Yeo SJ, et al. Continuous femoral nerve block in total knee arthroplasty: immediate and two-year outcomes. J Arthroplasty 2009;24:204-9.
96. Perrin SB, Purcell AN. Intraoperative ketamine may influence persistent pain following knee arthroplasty under combined general and spinal anaesthesia: a pilot study. Anaesth Intensive Care 2009;37:248-53.
97. Ilfeld BM, Meyer RS, Le LT, et al. Health-related quality of life after tricompartment knee arthroplasty with and without an extended-duration continuous femoral nerve block: a prospective, 1-year follow-up of a randomized, triple-masked, placebo-controlled study. Anesth Analg 2009;108:1320-5.
98. Ilfeld BM, Shuster JJ, Theriaque DW, et al. Long-term pain, stiffness, and functional disability after total knee arthroplasty with and without an extended ambulatory continuous femoral nerve block: A prospective, 1-year follow-up of a multicenter, randomized, triple-masked, placebo-controlled trial. Reg Anesth Pain Med 2011;36:116-20.
99. Morin AM, Kratz CD, Eberhart LH, et al. Postoperative analgesia and functional recovery after total-knee replacement: comparison of a continuous posterior lumbar plexus (psoas compartment) block, a continuous femoral nerve block, and the combination of a continuous femoral and sciatic nerve block. Reg Anesth Pain Med 2005;30:434-45.
100. Davidson E, Machi A, Sztain J, et al. Adductor canal versus femoral continuous peripheral nerve blocks for knee arthroplasty: A one-year follow-up pilot study of two randomized, controlled clinical trials. Reg Anesth Pain Med 2016;41.
101. Wang F, Zhou Y, Sun J, et al. Influences of continuous femoral nerve block on knee function and quality of life in patients following total knee arthroplasty. Int J Clin Exp Med 2015;8:19120-5.
102. Anastase DM, Winckelmann J, Geiger P. Effects of regional anaesthesia techniques on patients' satisfaction after total knee arthroplasty. Jurnalul Roman de Anestezie Terapie Intensiva 2014;21:35-43.

For peer review only - http://bmjopen.bmj.com/site/about/guidelines.xhtml

103. Yue D-b, Wang B-l, Liu K-p, et al. Efficacy of multimodal cocktail periarticular injection with or without steroid in total knee arthroplasty. Chinese Med J 2013;126:3851-5.
104. Spreng UJ, Andersson E, Dahl V. Long-term outcome after total knee arthroplasty local infiltration analgesia (LIA) vs. Epidural analgesia. Br J Anaesth 2012;108:ii419.
105. Spreng UJ, Dahl V, Hjall A, et al. High-volume local infiltration analgesia combined with intravenous or local ketorolac+morphine compared with epidural analgesia after total knee arthroplasty. Br J Anaesth 2010;105:675-82.
106. Foadi N, Karst M, Frese-Gaul A, et al. The improved quality of postoperative analgesia after intrathecal morphine does not result in improved recovery and quality of life in the first 6 months after orthopedic surgery: a randomized controlled pilot study. J Pain Res 2017;10:1059-69.
107. Mayoral O, Salvat I, Martin MT, et al. Efficacy of myofascial trigger point dry needling in the prevention of pain after total knee arthroplasty: A randomized, double-blinded, placebo-controlled trial. Evid Based Complement Alternat Med 2013;2013:Article ID 694941.
108. Abdel-Salam A, Eyres KS. Effects of tourniquet during total knee arthroplasty. A prospective randomised study. J Bone Joint Surg (Br) 1995;77:250-3.
109. Sukur E, Ozturkmen Y, Akman YE, et al. The effect of tourniquet and knee position during wound closure after total knee arthroplasty on early recovery of range of motion: a prospective, randomized study. Arch Orthop Trauma Surg 2016:1-8.
110. Zhang Q, Dong J, Gong K, et al. Effects of tourniquet use on perioperative outcome in total knee arthroplasty. Chinese Journal of Reparative and Reconstructive Surgery 2016;30:421-5.
111. Thomas D, Wareham K, Cohen D, et al. Autologous blood transfusion in total knee replacement surgery. Br J Anaesth 2001;86:669-73.
112. Aggarwal AK, Shashikanth VS, Marwaha N. Platelet-rich plasma prevents blood loss and pain and enhances early functional outcome after total knee arthroplasty: a prospective randomised controlled study. Int Orthop 2014;38:387-95.
113. Wang X. Impact of local compression cryotherapy combined with continuous passive motion on the early functional recovery after total knee arthroplasty. Chinese Journal of Tissue Engineering Research 2017;21:998-1003.
114. Leach W, Reid J, Murphy F. Continuous passive motion following total knee replacement: a prospective randomized trial with follow-up to 1 year. Knee Surg Sports Traumatol Arthrosc 2006;14:922-6.
115. Sahin E, Akalin E, Bircan C, et al. The effects of continuous passive motion on outcome in total knee arthroplasty. Journal of Rheumatology and Medical Rehabilitation 2006;17:85-90.
116. Pope RO, Corcoran S, McCaul K, et al. Continuous passive motion after primary total knee arthroplasty. Does it offer any benefits? J Bone Joint Surg (Br) 1997;79:914-7.
117. Beaupre LA, Davies DM, Jones CA, et al. Exercise combined with continuous passive motion or slider board therapy compared with exercise only: a randomized controlled trial of patients following total knee arthroplasty. Physical therapy 2001;81:1029-37.
118. Kumar PJ, McPherson EJ, Dorr LD, et al. Rehabilitation after total knee arthroplasty: a comparison of 2 rehabilitation techniques. Clin Orthop Relat R 1996:93-101.

For peer review only - http://bmjopen.bmj.com/site/about/guidelines.xhtml

119. Worland RL, Arredondo J, Angles F, et al. Home continuous passive motion machine versus professional physical therapy following total knee replacement. J Arthroplasty 1998;13:784-7.
120. MacDonald SJ, Bourne RB, Rorabeck CH, et al. Prospective randomized clinical trial of continuous passive motion after total knee arthroplasty. Clin Orthop Relat R 2000:30-5.
121. Stevens-Lapsley JE, Balter JE, Wolfe P, et al. Early neuromuscular electrical stimulation to improve quadriceps muscle strength after total knee arthroplasty: a randomized controlled trial. Physical therapy 2012;92:210-26.
122. Levine M, McElroy K, Stakich V, et al. Comparing conventional physical therapy rehabilitation with neuromuscular electrical stimulation after TKA. Orthopedics 2013;36:e319-24.
123. Adravanti P, Nicoletti S, Setti S, et al. Effect of pulsed electromagnetic field therapy in patients undergoing total knee arthroplasty: a randomised controlled trial. Int Orthop 2014;38:397-403.
124. Rahmann AE, Brauer SG, Nitz JC. A specific inpatient aquatic physiotherapy program improves strength after total hip or knee replacement surgery: a randomized controlled trial. Arch Phys Med Rehabil 2009;90:745-55.
125. Hill SP, Flynn J, Crawford EJP. Early discharge following total knee replacement -- a trial of patient satisfaction and outcomes using an orthopaedic outreach team. J Orthop Nurs 2000;4:121-6.
126. Jacobson AF, Umberger WA, Palmieri PA, et al. Guided imagery for total knee replacement: A randomized, placebo-controlled pilot study. J Altern Complement Med 2016;22:563-75.

For peer review only - http://bmjopen.bmj.com/site/about/guidelines.xhtml

Reporting checklist for systematic review and meta-analysis.

Based on the PRISMA guidelines.

Instructions to authors

Complete this checklist by entering the page numbers from your manuscript where readers will find each of the items listed below.

Your article may not currently address all the items on the checklist. Please modify your text to include the missing information. If you are certain that an item does not apply, please write "n/a" and provide a short explanation.

Upload your completed checklist as an extra file when you submit to a journal.

In your methods section, say that you used the PRISMA reporting guidelines, and cite them as:

Moher D, Liberati A, Tetzlaff J, Altman DG, The PRISMA Group. Preferred Reporting Items for Systematic Reviews and Meta-Analyses: The PRISMA Statement

		Reporting Item	Page Number
	#1	Identify the report as a systematic review, meta-analysis, or both.	1
Structured summary	#2	Provide a structured summary including, as applicable: background; objectives; data sources; study eligibility criteria, participants, and interventions; study appraisal and synthesis	2

For peer review only

		methods; results; limitations; conclusions and implications of key findings; systematic review registration number	
Rationale	#3	Describe the rationale for the review in the context of what is already known.	4
Objectives	#4	Provide an explicit statement of questions being addressed with reference to participants, interventions, comparisons, outcomes, and study design (PICOS).	4
Protocol and registration	#5	Indicate if a review protocol exists, if and where it can be accessed (e.g., Web address) and, if available, provide registration information including the registration number.	4
Eligibility criteria	#6	Specify study characteristics (e.g., PICOS, length of follow-up) and report characteristics (e.g., years considered, language, publication status) used as criteria for eligibility, giving rational	5
Information sources	#7	Describe all information sources in the search (e.g., databases with dates of coverage, contact with study authors to identify additional studies) and date last searched.	5
Search	#8	Present full electronic search strategy for at least one database, including any limits used, such that it could be repeated.	See note 1
Study selection	#9	State the process for selecting studies (i.e., for screening, for determining eligibility, for inclusion in the systematic review, and, if applicable, for inclusion in the meta-analysis).	5,6

For peer review only

Data collection process	#10	Describe the method of data extraction from reports (e.g., piloted forms, independently by two reviewers) and any processes for obtaining and confirming data from investigators.	6
Data items	#11	List and define all variables for which data were sought (e.g., PICOS, funding sources), and any assumptions and simplifications made.	6
Risk of bias in individual studies	#12	Describe methods used for assessing risk of bias in individual studies (including specification of whether this was done at the study or outcome level, or both), and how this information is to be used in any data synthesis.	6
Summary measures	#13	State the principal summary measures (e.g., risk ratio, difference in means).	6
Planned methods of analyis	#14	Describe the methods of handling data and combining results of studies, if done, including measures of consistency (e.g., I2) for each meta-analysis.	6
Risk of bias across studies	#15	Specify any assessment of risk of bias that may affect the cumulative evidence (e.g., publication bias, selective reporting within studies).	6
Additional analyses	#16	Describe methods of additional analyses (e.g., sensitivity or subgroup analyses, meta-regression), if done, indicating which were pre-specified.	6

For peer review only

Study selection	#17	Give numbers of studies screened, assessed for eligibility, and included in the review, with reasons for exclusions at each stage, ideally with a flow diagram.	See note 2
Study characteristics	#18	For each study, present characteristics for which data were extracted (e.g., study size, PICOS, follow-up period) and provide the citation.	See note 3
Risk of bias within studies	#19	Present data on risk of bias of each study and, if available, any outcome-level assessment (see Item 12).	See note 4
Results of individual studies	#20	For all outcomes considered (benefits and harms), present, for each study: (a) simple summary data for each intervention group and (b) effect estimates and confidence intervals, ideally with a forest plot.	See note 5
Synthesis of results	#21	Present the main results of the review. If meta-analyses are done, include for each, confidence intervals and measures of consistency.	15-21
Risk of bias across studies	#22	Present results of any assessment of risk of bias across studies (see Item 15).	See note 6
Additional analysis	#23	Give results of additional analyses, if done (e.g., sensitivity or subgroup analyses, meta-regression [see Item 16]).	15-21
Summary of Evidence	#24	Summarize the main findings, including the strength of evidence for each main outcome; consider their relevance to key groups (e.g., health care providers, users, and policy makers	21-23

For peer review only

Limitations	#25	Discuss limitations at study and outcome level (e.g., risk of bias), and at review level (e.g., incomplete retrieval of identified research, reporting bias).	21-23
Conclusions	#26	Provide a general interpretation of the results in the context of other evidence, and implications for future research.	21-23
Funding	#27	Describe sources of funding or other support (e.g., supply of data) for the systematic review; role of funders for the systematic review.	23

Author notes

1. 5, Supplemetary material

2. 6, Figure 1

3. 15-21, Table1, Supplementary material

4. 6, Supplementary material

5. 15-21, Table1, Supplementary material

6. 15-21, Supplementary material

The PRISMA checklist is distributed under the terms of the Creative Commons Attribution License CC-BY. This checklist was completed on 21. November 2018 using http://www.goodreports.org/, a tool made by the EQUATOR Network in collaboration with Penelope.ai

For peer review only

BMJ Open

Are peri-operative interventions effective in preventing chronic pain after primary total knee replacement? A systematic review

Journal:	*BMJ Open*
Manuscript ID	bmjopen-2018-028093.R1
Article Type:	Research
Date Submitted by the Author:	09-Apr-2019
Complete List of Authors:	Beswick, Andrew; University of Bristol, School of Clinical Sciences<br>Dennis, Jane; University of Bristol, Musculoskeletal Research Unit<br>Gooberman-Hill, Rachael; University of Bristol, Bristol Medical School<br>Blom, AW; University of Bristol,<br>Wylde, Vikki; University of Bristol, Musculoskeletal Research Unit
**Primary Subject Heading**:	Evidence based practice
Secondary Subject Heading:	Surgery
Keywords:	Knee < ORTHOPAEDIC & TRAUMA SURGERY, Pain management < ANAESTHETICS, REHABILITATION MEDICINE

SCHOLARONE™
Manuscripts

Are peri-operative interventions effective in preventing chronic pain after primary total knee replacement? A systematic review

Andrew D Beswick[1], Jane Dennis[1], Rachael Gooberman-Hill[1,2], Ashley W Blom[1,2], Vikki Wylde[1,2]

[1] Musculoskeletal Research Unit, Translational Health Sciences, Bristol Medical School, University of Bristol

[2] National Institute for Health Research Bristol Biomedical Research Centre, University Hospitals Bristol NHS Foundation Trust and University of Bristol

Corresponding author:

Andrew David Beswick

Musculoskeletal Research Unit

Translational Health Sciences

Bristol Medical School

University of Bristol

Learning and Research Building, Level 1

Southmead Hospital

Bristol

BS10 5NB

email: andy.beswick@bristol.ac.uk

Telephone: 0117 414 7870

For peer review only

For peer review only - http://bmjopen.bmj.com/site/about/guidelines.xhtml

ABSTRACT

Objectives

For many people with advanced osteoarthritis, total knee replacement (TKR) is an effective treatment for relief of pain and improvement of function. Features of peri-operative care may be associated with the adverse event of chronic pain six months or longer after surgery; effects may be direct, e.g. through nerve damage or surgical complications, or indirect through increasing risks of adverse events. The objective of this systematic review is to evaluate whether non-surgical peri-operative interventions prevent long-term pain after TKR.

Methods

We conducted a systematic review of peri-operative interventions for adults with osteoarthritis receiving primary TKR evaluated in a randomised controlled trial (RCT). We searched *The Cochrane Library*, MEDLINE, Embase, PsycINFO and CINAHL from inception to February 2018. After screening, two reviewers evaluated articles. Studies at low risk of bias according to the Cochrane tool were included.

Interventions

Peri-operative non-surgical interventions; control receiving no intervention or alternative treatment.

Primary and secondary outcome measures

Pain or score with pain component assessed at six months or longer post-operative.

Results

44 RCTs at low risk of bias assessed long-term pain. Intervention heterogeneity precluded meta-analysis and definitive statements on effectiveness. There was encouragement for further research into local infiltration analgesia, ketamine infusion, pregabalin, and electric muscle stimulation. In the studies we identified, tranexamic acid to prevent blood loss was not associated with long-term pain. Many extensively researched interventions including venous thromboembolism prevention have not been evaluated in relation to long-term pain.

Conclusions

To prevent chronic pain after TKR, peri-operative interventions including components of multimodal analgesia, early rehabilitation and supported discharge, electrical stimulation and anabolic steroids show promise that merits further research. Tranexamic use is not associated

For peer review only

For peer review only - http://bmjopen.bmj.com/site/about/guidelines.xhtml

with chronic pain but the long-term consequences of many widely researched treatments have not been reported.

STRENGTHS AND LIMITATIONS

- For the first time, this systematic review brings together contemporary evidence on aspects of peri-operative care for people with total knee replacement and their effects on long-term pain.
- Only studies assessed to be at low risk of bias were included in the narrative synthesis.
- Intervention and outcome heterogeneity precluded meta-analysis.

KEYWORDS

Total knee replacement; Systematic review; Randomised controlled trial; Peri-operative care; Long-term pain

For peer review only

For peer review only - http://bmjopen.bmj.com/site/about/guidelines.xhtml

BACKGROUND

In the US about 13% of men and 19% of women will be diagnosed with knee osteoarthritis and over half will receive a total knee replacement (TKR)[1]. For people with advanced osteoarthritis unresponsive to pharmacological or conservative treatments, TKR aims to relieve pain and improve function. In the UK nearly 100,000 primary TKRs were performed in 2017[2,3] and in the USA in 2010, an estimated 4.7 million people were living with a TKR[4]. Despite good outcomes for many, some people report long-term pain and are disappointed with their surgery[5,6]. After TKR, pain levels plateau from about 6 months[7,8] after which persistent pain is considered “chronic”[9] and is reported by 10-34% of patients[10].

The mechanisms that influence the development of chronic pain after total knee replacement may be biological, mechanical and psychosocial. Biological causes include the sensitising impact of long-term pain from osteoarthritis[11,12], inflammation, infection and localised nerve injury[13]. Mechanical causes include altered gait, prosthesis loosening, and effects on ligaments[14,15]. Psychological factors including depression and catastrophizing may also influence outcomes[16-19]. Much research has focused on pre-operative predictors of outcomes and these include pain intensity, presence of widespread pain, anxiety, depression and catastrophizing.[10,20] However, attempts to target or modify pre-operative care have, as yet, shown no benefit regarding chronic pain or other long-term patient outcomes[10,21-23].

Peri-operative risk factors suggest that appropriate interventions may reduce long-term pain. For example, acute post-operative pain, which may be a direct consequence of the operation, anaesthetic protocol and subsequent analgesia, or related to particular aspects of care, is an acknowledged risk factor for chronic post-surgical pain[24].

In the peri-operative period from hospital admission to the early stages of recovery, care focuses on acute pain management, prevention of adverse events, facilitation of early mobilisation and timely discharge. However, for people with osteoarthritis the key aim of TKR is the achievement of a long-term painless and well-functioning knee with no adverse events. All aspects of peri-operative care should work together to achieve this.

Any treatment in the peri-operative period including pain management, blood conservation, deep vein thrombosis (DVT) and infection prevention, and inpatient rehabilitation could potentially affect patient recovery and chronic pain, either directly or indirectly. Direct mechanisms may be through prevention of nerve damage[25], post-thrombotic syndrome[26], reperfusion injury[27] and articular bleeding[28]. For other treatments, pathways leading to long-

For peer review only - http://bmjopen.bmj.com/site/about/guidelines.xhtml

term pain may be indirect, possibly being mediated through increased risks of adverse events[29]. Irrespective of mechanism, chronic pain is a highly prevalent adverse event after TKR and should be considered along with infection, DVT and other complications in the safety profile of interventions.

Our systematic review of randomised controlled trials (RCTs) aims to evaluate the effectiveness of treatments in the peri-operative period in preventing long-term pain after TKR. By focusing on studies with low risk of bias we aim to identify interventions with robust evidence of long-term effectiveness and identify gaps in the research base.

For peer review only

METHODS

The systematic review protocol was registered (PROSPERO CRD42017041382) and PRISMA reporting guidelines used[30]. A checklist is included as Supplementary material.

Patient and public involvement

As part of the STAR programme of research (NIHR RP-PG-0613-20001), this review benefited from extensive patient and public involvement. Advice was sought from patients and stakeholders at a group discussion in March 2016 with decisions made on inclusion criteria and outcomes. Our patient advisory group comprises five patients with experience of long-term pain after TKR, supported by a dedicated co-ordinator. This group will advise on dissemination of the study results to a general audience including plain language summaries.

Eligibility criteria

Studies were eligible if they satisfied PICOS criteria defined in the protocol. Participants were adults receiving unilateral primary TKR with osteoarthritis in at least 75% of patients. Pharmacological or non-pharmacological interventions commenced in the peri-operative setting with “peri-operative” reflecting the time from hospital admission to immediately post-discharge. Interventions relating to implant designs and surgical procedures were excluded. The comparator was usual care, placebo or an alternative intervention. Outcomes were, in preference, patient-reported joint-specific pain intensity measured by tools such as the Western Ontario and McMaster Universities Osteoarthritis Index (WOMAC) or Oxford Knee Score (OKS). If joint-specific measures were unavailable, pain dimensions from quality of life measures were used or pain rated on a visual analogue scale (VAS) or numerical rating scale (NRS). We also considered composite patient-reported outcome measures and surgeon scores which included a pain intensity component, such as the American Knee Society Score (KSS) and Hospital for

For peer review only - http://bmjopen.bmj.com/site/about/guidelines.xhtml

Special Surgery (HSS) score. Measures specifically of neuropathic pain were also used. The occurrence of adverse events was summarised. The studies included were RCTs with follow up at ≥6 months after surgery and a pain outcome or score including pain. Authors of studies were contacted regarding incomplete pain outcome data.

Database searches

We established an Endnote database of all RCTs in TKR. On 14th February 2018, a final search from database inception was conducted in: *The Cochrane Library*; MEDLINE, Embase and PsycINFO on Ovid; and CINAHL on EBSCOhost. The MEDLINE search strategy is included as supplementary material. Citations of key articles were tracked in Web of Science. No language restrictions were applied, and translations made. Studies reported as abstracts or unobtainable using inter-library loans and author contact were excluded.

Screening and data extraction

We imported records into Endnote X7 (Thomson Reuters). An initial screen by one reviewer excluded clearly irrelevant articles. Subsequently, abstracts and full articles were screened independently by two reviewers and reasons for exclusion recorded.

Data were extracted onto piloted forms and an Excel spreadsheet by one reviewer, specifically: country; dates; participants (indication, age, sex); inclusion and exclusion criteria; intervention and control content; setting, timing, duration and intensity of intervention; follow up intervals; losses to follow up; pain outcome data; and serious adverse events. Data was checked against source material by a second reviewer.

Authors were contacted for missing data, and data provided for previous reviews was used[10,31].

Quality assessment

Potential sources of bias were assessed by two experienced reviewers using the Cochrane risk of bias tool[32], specifically: the randomisation process; deviations from intended interventions; missing outcome data (>20%), measurement of the outcome; and selection of the reported result. Studies with serious concerns relating to risk of bias were considered high risk and those with limited reporting unclear risk. Studies with high or unclear risk of bias were excluded from the narrative synthesis but are included in supplementary summary tables with reasons for exclusion.

Data analysis

For peer review only - http://bmjopen.bmj.com/site/about/guidelines.xhtml

Insufficient studies with similar interventions and outcomes were identified for meta-analysis, and a narrative synthesis is presented. Results reported with p-values ≤0.001 were considered “strong” evidence of effectiveness[33], p-values 0.001-0.05 “some” evidence, and p-values 0.05-0.1 “weak” evidence. When authors reported results “statistically significant” with no p-value, this was noted. Where possible, effect sizes were compared with published minimal clinically important differences (MCID). Concerns relating to adverse events were summarised.

RESULTS

Figure 1 shows review progress and reasons for exclusion. Of 1515 RCTs of interventions in the peri-operative setting, 1385 had no long-term follow up. Peri-operative interventions with follow up of ≥six months were evaluated in 130 RCTs of which 76 reported a pain outcome or score with a pain component. Detailed intervention and study characteristics and risk of bias assessments are provided as supplementary material. Studies excluded had concerns for risk of bias pertaining to at least one of: large baseline differences in group characteristics or numbers in groups (n=4); incomplete outcome data (n=15); limited or selective reporting (n=12); or un-blinded surgeon follow up (n=1).

Details of 44 studies assessed to be at low risk of bias are summarised in Table 1. In 34 studies, patients received TKR exclusively for osteoarthritis and in three, 75% or more patients. In seven studies there was no information on reason for surgery but there was no suggestion that patients had an indication other than osteoarthritis. Interventions focused on pain management (n=20), tourniquets (n=5), compression bandages (n=1), blood conservation (n=7), denusomab (n=1), continuous passive motion (n=2), electrical stimulation (n=2), rehabilitation (n=4), wound management (n=1) and anabolic steroids (n=1). Primary pain outcome measures reported were VAS or NRS pain (n=12), WOMAC pain (n=7), KOOS pain (n=3), Leeds assessment of Neuropathic Symptoms and Signs Pain Scale (S-LANSS) (n=1), SF-36 bodily pain (n=1), or composite scores including a pain measure, OKS or WOMAC (n=10), KSS or HSS (n=10). Latest outcomes were recorded at 6 months (n=12), 12 months (n=26) and 24 months (n=6). Reporting of adverse events covered the entire follow up period in 27 studies, short-term after surgery in 15 studies, but were not reported in two studies.

For peer review only - http://bmjopen.bmj.com/site/about/guidelines.xhtml

Table 1. Perioperative interventions with follow up for pain or score at 6 months or later and assessed to be at low risk of bias

Study	Treatment common to randomised groups	Intervention	Number patients	Follow up<br><br>Group difference
Pain management: nerve blocks				
Albrecht et al. 2014[34]<br><br>Canada, 2009-2011,<br><br>1 hospital	SNB	1. FNB continuous high<br><br>2. FNB continuous low<br><br>3. FNB single	99	1 year<br><br>WOMAC score: no difference (p=0.68)
Choy et al. 2011[35]<br><br>Korea, 2006-2007,<br><br>1 surgeon	PCA	1. FNB continuous long<br><br>2. FNB continuous short	61	1 year<br><br>WOMAC pain: no difference (p=0.2)
Fan et al. 2016[36]<br><br>China, 2012-2014,<br><br>2 surgeons	PCA	1. FNB single<br><br>2. LIA	157	1 year<br><br>KSS: no difference (p=0.51)
Gao et al. 2017[37]<br><br>China, 2014-2015,<br><br>1 centre	LIA	1. General anaesthesia<br><br>2. FNB single<br><br>3. FNB/ SNB single	150	6 months<br><br>HSS score: no significant difference (p> 0.05)
Macrinici et al. 2017[38]<br><br>USA, Before 2017<br><br>1 centre	LIA	1. ACB single<br><br>2. FNB single	98	6 months<br><br>VAS pain: no difference
Nader et al. 2012[39]<br><br>USA, 2007-2008,<br><br>1 surgeon	PCA	1. FNB continuous<br><br>2. Oral opioid	62	1 year<br><br>NRS pain stair: some evidence favouring opioid (p=0.01) but not consistent. Overall NRS pain: no difference (p=1.0)<br><br>VTE: concern opioid
Peng et al. 2014[40]<br><br>China, Before 2014,		1. FNB continuous<br><br>2. PCA	280	6 months and 1 year<br><br>NRS pain: some evidence favouring FNB at 6 months

For peer review only - http://bmjopen.bmj.com/site/about/guidelines.xhtml

1 centre				(p=0.021); no difference at 1 year (p=0.273)
Reinhardt et al. 2014[41]<br><br>USA, 2010-2012,<br><br>2 surgeons		1. FNB single/ epidural<br><br>2. LIA 48 hours	94	1 year<br><br>VAS pain: no difference
Wegener et al. 2013[42]<br><br>The Netherlands, 2008-2010,<br><br>1 centre	FNB	1. SNB single<br><br>2. SNB continuous<br><br>3. PCA	89	1 year<br><br>WOMAC pain: no difference (p=0.81)
Widmer et al. 2012[43]<br><br>Australia, before 2012,<br><br>2 surgeons	LIA, PCA	1. FNB single<br>2. Control no FNB	55	1 year<br><br>WOMAC pain: no difference (p=0.74)
Wu and Wong 2014[44]<br><br>China, 2009-2011,<br><br>1 centre		1. FNB continuous<br><br>2. PCA	60	6 months<br><br>KSS: no difference (p=0.513)
Pain management: LIA				
McDonald et al. 2016[45]<br><br>UK, 2010-2011<br><br>1 hospital		1. LIA<br><br>2. PCA	222	1 year<br><br>OKS: no difference (p=0.915)
Motififard et al. 2017[46]<br><br>Iran, 2014-2015<br><br>1 hospital		1. LIA pre-emptive injection<br><br>2. Control saline with epinephrine	120	6 months<br><br>KSS: weak evidence favouring LIA (p=0.07). Difference between groups (14.2/200) less than MCID (12.3/200).
Niemeläinen et al. 2014[47]<br><br>Finland, 2011-2012<br><br>1 hospital	PCA	1. LIA<br><br>2. Control saline	56	1 year<br><br>OKS: weak evidence from means and confidence intervals favouring LIA. Difference (2.7/48) less than MCID (4.0/48)
Seah et al. 2011[48]<br><br>Singapore, 2004-2005	PCA	1. LIA with corticosteroid<br><br>2. LIA no corticosteroid	100	6 months and 2 years<br><br>OKS: no difference

For peer review only - http://bmjopen.bmj.com/site/about/guidelines.xhtml

1 hospital				
Williams et al. 2013[49]<br>Canada, Before 2013<br>2 surgeons	LIA, PCA	1. LIA 48 hours<br>2. Control saline	51	6 months and 1 year<br>VAS pain: no difference (6 months p=0.836, 1 year p=0.767)
Wylde et al. 2015[50]<br>UK, 2009-2012<br>1 centre	FNB, PCA	1. LIA<br>2. Control no LIA	280	6 months and 1 year<br>WOMAC pain: weak evidence favouring LIA at 6 months p=0.063; 1 year p=0.107. Mean difference at 1 year (3.8/100) lower than MCID (8–9/100)
Pain management: Celecoxib				
Meunier et al. 2007[51]<br>Sweden, 2004-2005<br>1 centre	PCA	1. Celecoxib<br>2. Control placebo	44	1 year<br>KOOS/VAS pain: no difference
Pain management: Ketamine/ Nefopam				
Aveline et al. 2014[52]<br>France, 2005<br>1 centre	PCA	1. Ketamine infusion<br>2. Nefopam infusion<br>3. Control saline	75	6 months and 1 year<br>DN4/VAS pain: some evidence favouring ketamine (for DN4 p=0.02). Few patients had neuropathic pain at 12 months.
Pain management: Pregabalin				
Buvanendran et al. 2010[53]<br>USA, 2006-2007<br>Single centre	LIA, PCA	1. Pregabalin<br>2. Control placebo	240	6 months<br>NRS pain: some evidence favouring pregabalin at 6 months (p=0.0176)<br>S-LANSS pain: no neuropathic pain reported in pregabalin group compared with 5.2% of patients in control group (p=0.014)<br>Sedation and confusion day 0 and day 1: concern pregabalin

For peer review only - http://bmjopen.bmj.com/site/about/guidelines.xhtml

Tourniquet				
Ejaz et al. 2014[54]<br>Denmark, 2011-2012<br>1 centre	Tranexamic acid	1. Tourniquet<br>2. Tourniquet not inflated	64	6 months and 1 year<br>KOOS pain: no significant difference
Huang et al. 2017[55]<br>China, 2015<br>1 centre	Tranexamic acid	1. Tourniquet<br>2. No tourniquet	100	6 months<br>VAS pain: no difference (p=0.728)<br>Wound: concern tourniquet
Liu et al. 2014[56]<br>Australia, Before 2014<br>1 surgeon		1. Tourniquet<br>2. Tourniquet not inflated	20	6 months and 1 year<br>OKS: no significant difference<br>Transfusion: concern tourniquet
Mittal et al. 2012[57]<br>Australia, 2008-2010<br>1 centre		1. Tourniquet short duration<br>2. Tourniquet long duration	65	1 year<br>OKS: weak evidence from means and Cis on graph favouring long duration at 1 year. Mean difference (5) greater than MCID (4)<br>Transfusions/ adverse events: concern short
Zhang et al. 2017[58]<br>China, 2008-2011<br>1 surgeon		1. Tourniquet for entire operation<br>2. Tourniquet removed before wound closure<br>3. Tourniquet from first bone osteotomy until closure	150	6 months<br>HSS score: no difference (p=0.839)<br>Transfusions: concern late tourniquet start in groups 1 and 2
Compression bandage				
Brock et al. 2017[59]<br>UK, 2013-2014<br>1 hospital	Hydrocolloid dressing	1. Compression bandage<br>2. Standard crepe bandage	49	6 months<br>OKS: no difference (p=0.58)
Blood conservation				

For peer review only - http://bmjopen.bmj.com/site/about/guidelines.xhtml

Hourlier et al. 2015[60]<br><br>France, 2009-2010<br><br>1 hospital	Drain, tourniquet, electrocautery	1. Continuous infusion tranexamic acid<br><br>2. Control saline	106	6 months<br><br>KSS: no difference (p=0.90)
Huang et al. 2017[55]<br><br>China, 2015<br><br>1 centre	Tourniquet	1. Intravenous and topical tranexamic acid<br><br>2. No tranexamic acid	100	6 months<br><br>VAS pain: no difference (p=0.728)<br><br>HSS score: strong evidence favouring tranexamic acid (p<0.001). Mean difference (1.4/100) lower than MCID (8.3/100)<br><br>Blood loss: control concern
Kim et al. 2014[61]<br><br>Korea, 2009-2011<br><br>1 hospital	Tourniquet, drain, compressive dressing	1. Tranexamic acid<br><br>2. No tranexamic acid	180	1 year<br><br>WOMAC pain: no significant difference<br><br>Transfusion: control concern
Kusuma et al. 2013[62]<br><br>USA, Before 2013<br><br>1 hospital	Tourniquet, Esmarch bandage, electrocautery	1. Thrombin infusion<br><br>2. No thrombin infusion	80	6 months, 1 and 2 years<br><br>KSS: no difference (p=0.45)
Napier et al. 2014[63]<br><br>UK, 2003-2004<br><br>1 hospital		1. Passive flexion<br><br>2. Passive extension	180	1 year<br><br>OKS: no difference (p=0.27)<br><br>Transfusion: extension concern
Sa-Ngasoongsong et al. 2011[64]<br><br>Thailand, 2008-2009<br><br>1 hospital	Drain and compressive dressing	1. Tranexamic acid<br><br>2. Control saline	48	6 months<br><br>WOMAC score: no difference (p=0.282)<br><br>Transfusion: control concern
Sa-Ngasoongsong et al. 2013[65]<br><br>Thailand, 2010-2011<br><br>1 hospital	Drain and compressive dressing	1. Tranexamic acid 500mg<br><br>2. Tranexamic acid 250mg<br><br>3. Control saline	135	1 year<br><br>WOMAC score: no difference (p=0.42)<br><br>Transfusions: control and 250mg group concerns

For peer review only - http://bmjopen.bmj.com/site/about/guidelines.xhtml

Denusomab				
Ledin et al. 2017[66]<br><br>Sweden, 2012-2014<br><br>2 centres		1. Denusomab<br><br>2. Placebo	50	1 and 2 years<br><br>KOOS pain: no significant difference
Continuous passive motion				
Bennett et al. 2005[67]<br><br>Australia, 1997-2000<br><br>1 hospital	Physiotherapy	1. Standard CPM<br><br>2. Early flexion CPM<br><br>3. No CPM	147	1 year<br><br>KSS: no significant difference
Ersözlü et al. 2009[68]<br><br>Turkey, 2003-2004<br><br>1 hospital	Physiotherapy	1. CPM low and increasing<br><br>2. CPM high and increasing<br><br>3. No CPM	90	2 years<br><br>KSS: no difference (p=0.67)
Electrical stimulation				
Avramidis et al. 2011[69]<br><br>Greece, 2005-2006<br><br>1 hospital	Physiotherapy	1. Transcutaneous electric muscle stimulation<br><br>2. No treatment	76	1 year<br><br>SF-36 bodily pain: strong evidence favouring electrical stimulation (p<0.001). Mean difference (12.5/100) close to MCID (16.9/100).<br><br>OKS/ KSS: no difference
Moretti et al. 2012[70]<br><br>Italy, 2008-2010<br><br>1 hospital	Rehabilitation protocol	1. Pulsed electromagnetic fields<br><br>2. No treatment	30	6 months and 1 year<br><br>VAS pain: some evidence favouring electrical stimulation (p<0.05). Mean difference (2.1/10) greater than MCID (16.1/100)<br><br>Knee swelling: electrical stimulation concern
Rehabilitation				
Li et al. 2017[71]<br><br>China, 2015-2016	Standard rehabilitation	1. Walking guidance and training	86	6 months

For peer review only - http://bmjopen.bmj.com/site/about/guidelines.xhtml

1 hospital		2. No treatment		VAS pain/ HSS score: some evidence favouring walking (both p<0.01). Mean VAS pain difference (2.4/100) greater than MCID (16.1/100)
Liebs et al. 2012[72]<br>Germany, 2003-2004<br>4 hospitals	CPM, physiotherapy, post-discharge aquatic therapy	1. Early aquatic therapy<br>2. Delayed aquatic therapy	185	6 months, 1 and 2 years<br>WOMAC pain: no difference (p=0.22 at 12 months)
Mahomed et al. 2008[73]<br>Canada, 2000-2002<br>2 centres	Physiotherapy	1. Multidisciplinary supported early discharge and home physiotherapy<br>2. Transfer to rehabilitation centre	234 hip or knee replace ment	1 year<br>WOMAC pain: weak evidence favouring supported discharge (p=0.08). Mean difference (4) less than MCID (8-9)
Wang et al. 2014[74]<br>China, 2009-2010<br>1 centre		1. Wound closure in flexion<br>2. Wound closure in extension	80	6 months<br>VAS pain: no difference (p=0.64)
Wound management				
Kong et al. 2014[75]<br>South Korea, 2011<br>1 surgeon	Skin staples and closure strip	1. Silicone gel<br>2. Petroleum gel	100	6 months and 1 year<br>VAS pain: no difference (6 months p=0.886, 1 year p=0.201)
Anabolic steroids				
Hohmann et al. 2010[76]<br>Australia, Before 2010<br>1 surgeon	CPM. Cold compression,	1. Intramuscular nandrolone injections<br>2. Saline injections	10	6 and 9 months, 1 year<br>KSS: some evidence favouring nandrolone (6 months p=0.04, 9 months p=0.06, 12 months p=0.03). Difference at 12 months (10.2) close to MCID (12.3)<br>Bone mineral density: weak evidence favouring nandrolone

ACB adductor canal block; CPM Continuous passive motion; DN4 Douleur Neuropathique 4; FNB Femoral nerve block; HSS Hospital for Special Surgery; KOOS Knee injury and Osteoarthritis Outcome Score; KSS Knee Society Score; LIA local infiltration analgesia; MCID minimal clinically important

For peer review only - http://bmjopen.bmj.com/site/about/guidelines.xhtml

For peer review only

difference; NRS Numerical rating scale; OKS Oxford Knee Score; PCA Patient controlled analgesia; SF-36 Short Form 36 Health Survey; S-LANSS Leeds assessment of Neuropathic Symptoms and Signs Pain Scale; SNB Sciatic nerve block; VAS Visual analogue scale; VTE Venous thromboembolism; WOMAC Western Ontario and McMaster Universities Osteoarthritis Index.

For peer review only

For peer review only - http://bmjopen.bmj.com/site/about/guidelines.xhtml

Pain management

We identified 20 RCTs with 2393 participants evaluating components of multi-modal pain management. Four studies each were from China and the USA, two each from Canada and the UK and one each from Australia, Finland, France, Iran, Singapore, South Korea, Sweden and The Netherlands. All were conducted at a single centre and, in those with dates, participants were recruited between 2004 and 2015. Sample sizes ranged from 44 to 280 participants, with a median of 96. Four studies had three trial arms and 16 had two. The range of mean or median ages of participants in randomised groups was 61 to 73 years and, in 17/19 studies with data, a majority of participants were women.

Femoral nerve block

Femoral nerve blocks (FNB) were studied in 10 RCTs.

Three RCTs compared FNB with no FNB. In one study with 55 patients, WOMAC pain scores at one year were similar in patients receiving single-shot FNB and untreated controls[43]. All patients received local anaesthetic infiltration (LIA) and patient-controlled analgesia (PCA). In another study with all participants receiving LIA, 150 were randomised to receive single-shot FNB with or without sciatic nerve block (SNB), or general anaesthesia[37]. There were no differences in HSS scores between groups at six months. Continuous FNB was compared with oral hydrocodone opioid in 62 patients receiving PCA[39]. There was some evidence for ‘pain using stairs’ favouring hydrocodone (p=0.01) but no difference in overall NRS-rated pain at one year and concern over venous thromboembolism in 4/31 participants treated with hydrocodone.

In two RCTs, continuous FNB was compared with PCA. In one study with 60 participants, the KSS at six months was similar between groups[44]. In another study with 280 participants, there was some evidence for higher incidence of NRS-rated pain at six months in the PCA group than the FNB group (p=0.021) but not at 12 months (p=0.273).[40]

Two RCTs compared FNB with LIA. In one study, all 157 participants also received PCA[36]. At one year, KSS values were similar in single-shot FNB and LIA groups. In the other study, 94 participants were randomised to receive single-shot FNB with continuous epidural infusion or LIA through an intra-articular catheter[41]. VAS-rated pain was similar between groups at one year.

In two RCTs, FNB procedures were compared. In one study with 99 patients randomised to two FNB concentrations, there was no difference in WOMAC score between groups at 12 months[34]. In another study with 61 participants allocated to two different durations of FNB,

For peer review only - http://bmjopen.bmj.com/site/about/guidelines.xhtml

there was no difference in WOMAC pain scores at one year[35]. In these studies, all participants received either SNB[34] or PCA[35].

Single-shot FNB was compared with single adductor canal block in one RCT with 98 participants, all receiving LIA[38]. At six months there was no difference in VAS-rated pain.

Sciatic nerve block

In one study, 89 patients were randomised to single-shot SNB, continuous SNB, or PCA[42]. All patients received FNB. At 12 months, there were no differences in pain for single-shot SNB and continuous SNB on the WOMAC pain scale or VAS-rated pain at rest or during mobilisation. Similarly, there were no differences between single-shot SNB and PCA in WOMAC pain scale or VAS-rated pain at rest or during mobilisation, or between continuous SNB and PCA.

Local anaesthetic infiltration

Four RCTs compared LIA with placebo. In one study, all 280 participants received FNB and PCA[50]. There was weak evidence that WOMAC pain scores were better in the LIA group at six (p=0.063) but not at 12 months (p=0.107) when the difference in means of 3.8/100 was lower than the MCID of 8-9/100 reported by Ehrich and colleagues[77]. In another study, 56 patients received LIA including ketorolac, or saline placebo, and all received PCA[47]. At one year, mean differences and confidence intervals provided weak evidence that OKS scores were better in the LIA group but the difference in means of 2.7/48 was less than the MCID of 4/48 reported by Beard and colleagues[78]. LIA before surgical incision was compared with placebo in one study with 120 participants[46]. None received FNB or PCA. There was weak evidence for a better KSS (function and knee score components) at six months in those receiving LIA (p=0.07) with a difference in means of 14.2/200 exceeding the MCID of 12.3/200 reported by Lee and colleagues[79]. In another study, all 51 participants received LIA intra-operatively, followed by PCA[49]. Those randomised to post-operative catheter-delivered LIA with ketorolac, or saline placebo had similar VAS-rated pain at six and 12 months.

LIA delivered as an injection and post-operative infusion was compared with epidural PCA in one study with 222 patients[45]. There was no difference between groups in OKS at 12 months.

In one study of 100 participants, LIA with or without corticosteroid were compared[48]. All patients received PCA. At two years there was no difference in OKS between groups.

Oral celecoxib

For peer review only - http://bmjopen.bmj.com/site/about/guidelines.xhtml

In one RCT, 44 participants received oral celecoxib or placebo[51], as well as PCA. There were no differences between groups in KOOS or VAS-rated pain at 12 months.

Ketamine or nefopam infusion

In one RCT, ketamine infusion, nefopam infusion and saline placebo were compared in 75 patients, all of whom received PCA[52]. VAS-rated pain on movement did not differ between groups at 12 months. For the Douleur Neuropathique 4 (DN4) measure of neuropathic pain, there was some evidence favouring ketamine over placebo at six and 12 months (p=0.02), but overall, few patients reported neuropathic pain at 12 months.

Pregabalin

Oral pregabalin was compared with placebo in one RCT with 240 participants[53]. All received LIA and PCA. At six months, there was some evidence for better NRS pain in patients receiving pregabalin compared with placebo (p=0.0176) but the difference in means of 0.54/10 was less than the MCID of 1/10 reported by Salaffi and colleagues[80]. No participants receiving pregabalin reported neuropathic pain when assessed using the S-LANSS, compared with 5.2% of those receiving placebo (p=0.014). Patients receiving pregabalin were more likely to be sedated and confused in the first two days after surgery.

Tourniquet

Five studies with 399 participants explored tourniquet use to provide a bloodless field. Two studies each were from Australia and China, and one from Denmark. All were conducted at a single centre with participants recruited between 2008 and 2015. Sample sizes ranged from 20 to 150 participants, with a median of 65. The range of mean ages of participants in randomised groups was 66 to 71 years and in 3/5 studies, a majority of participants were women.

In three RCTs, participants received TKR with or without a tourniquet. In one study with 64 patients, a difference in KOOS pain favouring tourniquet use was not significant at six or 12 months[54]. In another study with 20 patients, the OKS was not significantly different between groups at six or 12 months[56]. There were three blood transfusions in the tourniquet group, compared with none in the ‘no tourniquet’ group. In the third study with 100 participants, VAS-rated pain and HSS scores were similar between groups at 6 months[55]. Six cases of wound ooze occurred in the tourniquet group.

In two RCTs, short and long-duration tourniquet use were compared. In one study with 65 participants, there was weak evidence based on graphical representation of means and

For peer review only - http://bmjopen.bmj.com/site/about/guidelines.xhtml

confidence intervals for improved OKS at 12 months in the long-duration group and the difference in means of 5/48[57] was greater than the MCID of 4/48. Adverse events were reported by 62% of participants receiving short-duration tourniquet compared with 38% in the long-duration group. The study was terminated early as 10 blood transfusions were required in the short-duration group compared with three in the long-duration group. In the second study with 150 participants, tourniquets were used in three different periods during surgery[58]. At six months, there were no differences between groups in HSS scores.

Blood conservation

Seven studies with 829 participants evaluated strategies to limit blood loss after TKR. Two studies were from Thailand, and one each from China, France, South Korea, the UK and the USA. All were conducted at a single centre with participants recruited between 2003 and 2015 when stated. Sample sizes ranged from 48 to 180 participants, with a median of 106. One study had three trial arms. The range of mean ages of participants in randomised groups was 65 to 74 years and in all studies, a majority of participants were women.

Tranexamic acid

Five RCTs evaluated tranexamic acid.

Tranexamic acid injections or infusions were compared with saline placebo or untreated control in four RCTs[55,61,64,65]. In all studies, control patients required more blood transfusions. In one study including 180 participants comparing intravenous tranexamic acid with untreated controls, there was no significant difference in WOMAC pain scores at one year[61]. In another study with 48 participants comparing intra-articular tranexamic acid injection with saline placebo, there was no significant difference in WOMAC scores at six months[64]. One study with 135 participants compared two intra-articular tranexamic acid doses and saline control[65]. There were no significant differences in WOMAC scores at one year. Intravenous and intra-articular tranexamic was compared with untreated controls in one study with 100 participants[55]. VAS-rated pain at six months was similar between groups, but there was strong evidence favouring tranexamic acid for HSS scores ($p<0.001$) although the difference in means of 1.4/100 was lower than the MCID of 8.3/100 reported by Singh and colleagues[81].

In one study, continuous tranexamic acid infusion was compared with a single bolus in 106 patients[60]. There was no difference between groups in KSS at six months or blood loss.

Thrombin infusion

For peer review only - http://bmjopen.bmj.com/site/about/guidelines.xhtml

In one RCT with 80 participants, thrombin infusion was compared with untreated control[62]. At one year there was no difference between groups in pain measured on the KSS.

Flexion or extension

For blood management, operated knees were kept in passive flexion or passive extension after surgery in one RCT with 180 patients[63]. At one year, OKS was similar between groups. Transfusion requirement was greater in patients with passive extension.

Compression bandage

One RCT conducted at a single UK centre with 49 participants recruited between 2013 and 2014 compared compression bandaging to reduce post-operative knee swelling with standard bandaging. The mean age of participants was about 69 years and a majority were women. OKS was similar in randomised groups at six months[59].

Wound management

One RCT with recruitment in 2011 at a single centre in South Korea evaluated a wound care strategy to limit post-operative scar pain. The mean age of participants was about 69 years and a majority were women. Investigators compared silicone gel application to the surgical scar with placebo in 100 participants[75]. There were no significant differences in VAS-rated pain at six and 12 months.

Denusomab

One RCT evaluated use of the antiresorptive monoclonal antibody Denusomab to promote bone healing. The study was conducted in two centres in Sweden with recruitment of 50 participants between 2012 and 2014. The mean age of participants was about 65 years and a majority were women. At 12 and 24 months there were no significant differences between groups in KOOS pain[66].

Continuous passive motion

Two RCTs with 237 participants evaluated use of continuous passive motion (CPM) to minimise joint stiffness and improve range of movement. Studies were conducted in single centres in Australia and Turkey with participant recruitment between 1997 and 2004 and both had three trial arms. Sample sizes were 90 and 147 participants. The mean ages of participants in studies were about 63 and 72 years and a majority of participants were women. In one study, 90 participants were randomised to no CPM, CPM at low flexion from post-operative day 1–7, or

For peer review only - http://bmjopen.bmj.com/site/about/guidelines.xhtml

CPM at high flexion from post-operative day 3–7[68]. There was no significant difference between groups in KSS at two years. In the other study, 147 participants were randomised to CPM with increasing range of movement from day 1–6, early flexion CPM from day 0–6, or no CPM[67]. There were no significant differences between groups in KSS at 12 months.

Electrical stimulation

Two RCTs with 106 participants conducted in single centres in Greece and Italy evaluated electrical stimulation which is believed to have anti-inflammatory activity and limit muscle atrophy. Studies included 76 and 30 participants recruited between 2005 and 2010. The mean ages of participants were 71 and 70 years and in one study that reported it, a majority of participants were female.

In one study with 76 participants receiving transcutaneous electric muscle stimulation from post-operative day two for six weeks or no intervention, Short Form 36 bodily pain showed strong evidence for greater improvement at one year in the intervention group compared to control ($p<0.001$)[69]. The difference in means of 12.5/100 was close to the MCID of 16.9/100 reported by Escobar and colleagues[82]. There were no differences in OKS or KSS scores. In another study with 30 participants, pulsed electromagnetic fields from post-operative day 7 were compared with untreated control[70]. At 12 months, there was some evidence that VAS-rated pain was lower in intervention patients compared with controls ($p<0.05$). The difference in means of 2.1/10 was greater than the MCID of 16.1/100 reported by Danoff and colleagues[83]. Knee swelling was common during the intervention.

Rehabilitation

Four RCTs with 585 participants recruited between 2000 and 2016 evaluated features of early rehabilitation focusing on regaining range of movement, functional independence and improving mobility. Two studies were conducted at single centres in China and at two and four centres in Canada and Germany respectively. Sample sizes ranged from 80 to 234 participants, with a median of 136. The range of mean ages of participants in randomised groups was 68 to 78 years and in 3/4 studies, a majority of participants were women.

Walking guidance and training

In one study, 86 participants were randomised to walking guidance and training from post-operative day two or no intervention further to standard rehabilitation[71]. At six months, there was some evidence that those receiving intervention had lower VAS-rated pain ($p<0.01$) and

For peer review only - http://bmjopen.bmj.com/site/about/guidelines.xhtml

HSS score (p<0.01) than controls. The difference in mean VAS-rated pain of 2.4/10 was greater than the MCID of 16.1/100.

Flexion or extension during knee closure

Targeting improved functional recovery, wound closure performed in 90° flexion was compared with wound closure in full extension in one study with 80 participants[74]. There was no difference between groups in VAS-rated pain at six months.

Aquatic therapy

In one study with 185 participants, aquatic therapy commencing on post-operative day six was compared with aquatic therapy commencing on day 14[72]. Patients reported similar WOMAC pain at 12 and 24 months.

Supported early discharge

In one study, early discharge supported by physiotherapist home visits and outpatient or self-directed physiotherapy was compared with two weeks of rehabilitation centre-based usual care[73]. The study included 234 individuals receiving TKR or total hip replacement. Compared with usual care, there was weak evidence that patients with early discharge had lower WOMAC pain scores at 12 months (p=0.08). The difference in means of 4 was less than the MCID of 8-9/100. Results were not presented separately but did not differ between patients with TKR or total hip replacement.

Anabolic steroids

Searches identified one study of anabolic steroids to improve post-operative muscle strength conducted in one centre in Australia with recruitment of 10 participants before 2010. The mean age of participants was about 66 years and a minority were women. Participants received intramuscular nandrolone injections or saline from post-operative day five for six months. KSS results indicated some evidence for improvement in the intervention group compared with controls at 12 months (p=0.03)[76]. The difference in means of 10.2/200 was close to the MCID of 12.3/200.

Interventions with no long-term outcome

Interventions with lack of RCT evidence are summarised in Figure 1.

While 148 RCTs of DVT prophylaxis were identified, only five reported long-term follow up, none of which included a pain or outcome score. Among 29 RCTs of antibiotic prophylaxis, 16

For peer review only - http://bmjopen.bmj.com/site/about/guidelines.xhtml

reported long-term follow up, but none included a pain or outcome score. Six RCTs evaluated the use of bisphosphonates and, although all reported long-term follow up, none reported pain or an outcome score. One study reported long-term follow up of an RCT of teriparatide but included no data on pain.

For some interventions, RCTs with long-term pain outcomes were identified, but none were at low risk of bias: cold therapy; guided imagery; platelet rich plasma; and trigger point needling.

Aspects of peri-operative care evaluated in RCTs but lacking long-term pain follow up were: adenosine triphosphate; alternative and Chinese medicine; assistive devices; brain stimulation; calcium supplements; cardiovascular drugs; colloids and crystalloids; comorbidity management; constipation treatment; creatine; delirium prevention; dexmedetomidine; glucocorticoids; glucose infusion; iron; laser therapy; methylprednisolone; music therapy; nausea prevention; nutritional supplements; physiological treatments; remote ischaemic preconditioning; sleep treatments; therapy dogs; and warming.

DISCUSSION

Peri-operative care for patients with osteoarthritis receiving TKR varies widely[84,85]. To guide decisions on appropriate care, the top level of evidence in the hierarchy of primary research is the RCT[86,87]. Bringing evidence from RCTs together in systematic reviews with thorough risk of bias assessment ensures that health professionals have the information they need to deliver a high-quality patient experience with safe, clinically-effective and cost-effective treatments[88]. Furthermore, systematic reviews can identify gaps in the evidence base and promote further research.

Much research in TKR aims to identify treatments that facilitate a speedy recovery with minimal short-term pain. However, patients choose to have joint replacement for long-term pain relief and reduction in functional limitations. Thus, changes to peri-operative care, supported by short-term RCT evidence, should be backed up with evidence about long-term effectiveness for reducing pain and reassurance that there are no long-term unfavourable consequences. To this end, we synthesised evidence from RCTs evaluating peri-operative interventions which have considered their long-term effects on pain outcomes.

A major focus of research into improving long-term pain after TKR has been through prevention of acute post-operative pain using multimodal analgesia. Our review provides some encouragement for further research on long-term benefits of intra-articular LIA injections, as previously shown in short-term studies[31,89], oral pregabalin, oral opioids, and in relation to

For peer review only - http://bmjopen.bmj.com/site/about/guidelines.xhtml

neuropathic pain, ketamine infusion. As well as potential benefits for reduced long-term pain, future studies will need to consider concerns associated with these interventions which may not have been identified in small studies including infection[31], venous thromboembolism[39] and sedation[53].

Nerve blocks are effective for managing peri-operative pain[90] but we identified no long-term benefit. In single studies, there was no benefit for nefopam infusion, oral celecoxib or LIA with additional corticosteroid. Regarding future studies, standardisation of the multi-modal regimen will allow evaluation of extra or alternative components in multiple studies in different settings. With such an approach, convincing evidence will accrue to guide multimodal pain management.

Tranexamic acid is highly effective in reducing blood transfusions during TKR[91]. We found no evidence that tranexamic acid affects long-term pain or, as observed in registry studies[92,93], adverse events. Single RCTs of thrombin infusion and maintenance of knee in flexion to prevent blood loss showed no effect on long-term pain. Tourniquets improve intraoperative visualisation of the joint, reduce blood loss and facilitate cement fixation but are associated with nerve damage, delayed recovery, acute pain and need for analgesics[94,95]. The RCTs we identified showed no effects of tourniquet use on long-term pain.

Consistent with a previous review[96], there was no suggestion that CPM affects long-term pain. Studies provided encouragement for further research into walking training, anabolic steroid injection, electrical stimulation and supported discharge.

For some interventions a direct mechanism is clear, but for others, reasons for long-term impact are less obvious. This may explain why, for example, no studies evaluated DVT prophylaxis with long-term follow up excepting a small number reporting adverse events. However, treatments to prevent symptomatic DVTs which occur in about 1% of treated patients[97] also reduce the incidence of asymptomatic DVT observed in about 28% of treated patients[98] and this may have long-term benefits. Conversely, new anticoagulants are associated with bleeding[99], which may increase the risk of wound complications[100] and joint infection[101] which are associated with long-term pain[102,103].

Our study is limited by the lack of meta-analysis which was not appropriate due to intervention and outcome heterogeneity. In the context of perioperative pain management, this was noted previously[89]. Our approach to assessing the evidence was a narrative synthesis of studies with low risk of bias. While this may seem overly restrictive, Cochrane risk of bias assessment allows us to screen out studies with important issues that may affect the validity of results. The

For peer review only - http://bmjopen.bmj.com/site/about/guidelines.xhtml

main potential source of bias was incomplete outcome assessment. Although studies with long-term follow up are naturally at higher risk of missing data, we maintained a standard in this domain as it is recognised that research participants who do not complete follow up assessments differ in outcomes from those with follow up data and their inclusion could change the interpretation of results[104].

Another limitation is that pain assessed with questionnaires does not take into account the effect of pain medications and assistive aids. About 58% of women and 40% of men report taking pain medications after TKR because of pain in the operated knee[105] and we must recognise that pain levels at follow up without this treatment might be considerably higher. Even with treatment, around 20% of patients report chronic pain after TKR[10] and in the context of a blinded RCT we should expect to be able to identify effects of peri-operative treatments.

We summarised p-values to assess the strength of evidence but, as statistically strong evidence may not reflect clinically important results[106], where possible we also compared effect sizes with MCIDs. Our review considered a diverse range of interventions at a specific time in the TKR pathway and, as we were unable to make clinical practice recommendations, we did not adopt the GRADE system[107] for this review.

An alternative approach to the prevention of chronic pain after TKR is the individualisation of care based on pain phenotype, genetic, psychosocial and other factors[108]. An example of this might be the peri-operative treatment only of individuals with neuropathic pain with pregabalin, as opposed to the non-stratified provision in the RCT of Buvanendran and colleagues[53]. In an RCT with pregabalin provided to patients with painful HIV-neuropathy, while no overall benefit was seen, a group with hyperalgesia responded to pregabalin treatment[109].

Our systematic review of peri-operative interventions brings together evidence on interventions in the peri-operative phase of the TKR pathway. Whilst not supportive of the inclusion of specific interventions in clinical practice to optimise long-term pain outcomes, there are clearly areas that merit research. High quality studies assessing long-term pain after peri-operative interventions are feasible and necessary to ensure that patients with osteoarthritis achieve good long-term outcomes after TKR.

ACKNOWLEDGEMENT

We thank Dr Mario Moric for conducting additional analyses on the study of Buvanendran and colleagues[53].

For peer review only - http://bmjopen.bmj.com/site/about/guidelines.xhtml

AUTHOR CONTRIBUTIONS

All authors, ADB, JD, RG-H and AWB, contributed to the conception and design of the study. ADB, JD and VW undertook the systematic review. ADB and JD carried out the risk of bias assessments. ADB drafted the article with revisions by JD, VW, RG-H and AWB. All authors approved the final version for publication.

ROLE OF THE FUNDING SOURCE

This article presents independent research funded by the National Institute for Health Research (NIHR) under its Programme Grants for Applied Research programme (RP-PG-0613-20001). This study was supported by the NIHR Biomedical Research Centre at University Hospitals Bristol NHS Foundation Trust and the University of Bristol. The views expressed in this publication are those of the authors and not necessarily those of the NHS, the National Institute for Health Research or the Department of Health and Social Care.

COMPETING INTERESTS STATEMENT

The authors report no competing interests.

DATA STATEMENT

All extracted data is included in the Supplementary material.

Legend

Figure 1. Systematic review flow diagram

References

1. Weinstein AM, Rome BN, Reichmann WM, et al. Estimating the burden of total knee replacement in the United States. *J Bone Joint Surg (Am)* 2013;95:385-92.
2. National Joint Registry for England, Wales, Northern Ireland and the Isle of Man: 15th Annual Report. Hemel Hempstead: NJR Centre 2018.
3. Scottish Arthroplasty Project. Annual report 2018. Edinburgh: NHS National Services Scotland; 2018.
4. Maradit Kremers H, Larson DR, Crowson CS, et al. Prevalence of total hip and knee replacement in the United States. *J Bone Joint Surg (Am)* 2015;97:1386-97.

For peer review only - http://bmjopen.bmj.com/site/about/guidelines.xhtml

5. Nilsdotter AK, Toksvig-Larsen S, Roos EM. Knee arthroplasty: are patients' expectations fulfilled? A prospective study of pain and function in 102 patients with 5-year follow-up. *Acta Orthop* 2009;80:55-61.

6. Scott CE, Howie CR, MacDonald D, et al. Predicting dissatisfaction following total knee replacement: a prospective study of 1217 patients. *J Bone Joint Surg (Br)* 2010;92:1253-8.

7. Bachmeier CJM, March LM, Cross MJ, et al. A comparison of outcomes in osteoarthritis patients undergoing total hip and knee replacement surgery. *Osteoarthritis Cart* 2001;9:137-46.

8. Dailiana ZH, Papakostidou I, Varitimidis S, et al. Patient-reported quality of life after primary major joint arthroplasty: a prospective comparison of hip and knee arthroplasty. *BMC Musculoskelet Disord* 2015;16:366.

9. Werner MU, Kongsgaard UE. Defining persistent post-surgical pain: is an update required? *Br J Anaesth* 2014;113:1-4.

10. Blom AW, Artz N, Beswick AD, et al. Improving patients' experience and outcome of total joint replacement: The RESTORE programme. *Programme Grants Appl Res* 2016;4.

11. Lundblad H, Kreicbergs A, Jansson KA. Prediction of persistent pain after total knee replacement for osteoarthritis. *J Bone Joint Surg (Br)* 2008;90-B:166-71.

12. Wylde V, Sayers A, Odutola A, et al. Central sensitization as a determinant of patients' benefit from total hip and knee replacement. *Eur J Pain* 2017;21:357-65.

13. Mandalia V, Eyres K, Schranz P, et al. Evaluation of patients with a painful total knee replacement. *J Bone Joint Surg (Br)* 2008;90:265-71.

14. Hofmann S, Seitlinger G, Djahani O, et al. The painful knee after TKA: a diagnostic algorithm for failure analysis. *Knee Surg Sports Traumatol Arthrosc* 2011;19:1442-52.

15. Toms AD, Mandalia V, Haigh R, et al. The management of patients with painful total knee replacement. *J Bone Joint Surg (Br)* 2009;91:143-50.

16. Wylde V, Trela-Larsen L, Whitehouse MR, et al. Preoperative psychosocial risk factors for poor outcomes at 1 and 5 years after total knee replacement. *Acta Orthop* 2017;88:530-6.

17. Burns LC, Ritvo SE, Ferguson MK, et al. Pain catastrophizing as a risk factor for chronic pain after total knee arthroplasty: A systematic review. *J Pain Res* 2015;8:21-32.

18. Edwards RR, Haythornthwaite JA, Smith MT, et al. Catastrophizing and depressive symptoms as prospective predictors of outcomes following total knee replacement. *Pain Res Manag* 2009;14:307-11.

19. Wylde V, Beswick AD, Dennis J, et al. Post-operative patient-related risk factors for chronic pain after total knee replacement: a systematic review. *BMJ Open* 2017;7:e018105.

For peer review only - http://bmjopen.bmj.com/site/about/guidelines.xhtml

20. Lewis GN, Rice DA, McNair PJ, et al. Predictors of persistent pain after total knee arthroplasty: a systematic review and meta-analysis. *Br J Anaesth* 2015;114:551-61.

21. Wang L, Lee M, Zhang Z, et al. Does preoperative rehabilitation for patients planning to undergo joint replacement surgery improve outcomes? A systematic review and meta-analysis of randomised controlled trials. *BMJ Open* 2016;6:e009857.

22. Bay S, Kuster L, McLean N, et al. A systematic review of psychological interventions in total hip and knee arthroplasty. *BMC Musculoskelet Disord* 2018;19:201.

23. Riddle DL, Keefe FJ, Ang DC, et al. Pain coping skills training for patients who catastrophize about pain prior to knee arthroplasty: A multisite randomized clinical trial. *J Bone Joint Surg (Am)* 2019;101:218-27.

24. Kehlet H, Jensen TS, Woolf CJ. Persistent postsurgical pain: risk factors and prevention. *Lancet* 2006;367:1618-25.

25. Haroutiunian S, Nikolajsen L, Finnerup NB, et al. The neuropathic component in persistent postsurgical pain: A systematic literature review. *Pain* 2013;154:95-102.

26. Ashrani AA, Heit JA. Incidence and cost burden of post-thrombotic syndrome. *J Thromb Thrombolysis* 2009;28:465-76.

27. Dreyer HC. Tourniquet use during knee replacement surgery may contribute to muscle atrophy in older adults. *Exerc Sport Sci Rev* 2016;44:61-70.

28. Kindsfater K, Scott R. Recurrent hemarthrosis after total knee arthroplasty. *J Arthroplasty* 1995;10:S52-S5.

29. Epstein NE. A review article on the benefits of early mobilization following spinal surgery and other medical/surgical procedures. *Surg Neurol Int* 2014;5:S66-73.

30. Moher D, Liberati A, Tetzlaff J, et al. Preferred reporting items for systematic reviews and meta-analyses: the PRISMA statement. *J Clin Epidemiol* 2009;62:1006-12.

31. Marques EM, Jones HE, Elvers KT, et al. Local anaesthetic infiltration for peri-operative pain control in total hip and knee replacement: systematic review and meta-analyses of short- and long-term effectiveness. *BMC Musculoskelet Disord* 2014;15:220.

32. Higgins JPT, Green S. Cochrane Handbook for Systematic Reviews of Interventions Version 5.1.0 [Updated March 2011]: The Cochrane Collaboration; 2011. Available from: http://www.cochrane-handbook.org.

33. Sterne JA, Davey Smith G. Sifting the evidence-what's wrong with significance tests? *BMJ* 2001;322:226-31.

34. Albrecht E, Morfey D, Chan V, et al. Single-injection or continuous femoral nerve block for total knee arthroplasty? *Clin Orthop Relat Res* 2014;472:1384-93.

For peer review only - http://bmjopen.bmj.com/site/about/guidelines.xhtml

35. Choy WS, Lee SK, Kim KJ, et al. Two continuous femoral nerve block strategies after TKA. *Knee Surg Sports Traumatol Arthrosc* 2011;19:1901-8.
36. Fan L, Yu X, Zan P, et al. Comparison of local infiltration analgesia with femoral nerve block for total knee arthroplasty: A prospective, randomized clinical trial. *J Arthroplasty* 2016;31:1361-5.
37. Gao WL, Li H, Liu BQ, et al. Analgesic effect of femoral and sciatic nerve block under multimodal analgesia in total knee arthroplasty. *Chinese Journal of Tissue Engineering Research* 2017;21:2966-72.
38. Macrinici GI, Murphy C, Christman L, et al. Prospective, double-blind, randomized study to evaluate single-injection adductor canal nerve block versus femoral nerve block: postoperative functional outcomes after total knee arthroplasty. *Reg Anesth Pain Med* 2017;42:10-6.
39. Nader A, Kendall MC, Wixson RL, et al. A randomized trial of epidural analgesia followed by continuous femoral analgesia compared with oral opioid analgesia on short- and long-term functional recovery after total knee replacement. *Pain Med* 2012;13:937-47.
40. Peng L, Ren L, Qin P, et al. Continuous femoral nerve block versus intravenous patient controlled analgesia for knee mobility and long-term pain in patients receiving total knee replacement: a randomized controlled trial. *Evid Based Complement Alternat Med* 2014;2014:569107.
41. Reinhardt KR, Duggal S, Umunna BP, et al. Intraarticular analgesia versus epidural plus femoral nerve block after TKA: A randomized, double-blind trial. *Clin Orthop Relat Res* 2014;472:1400-8.
42. Wegener JT, van Ooij B, van Dijk CN, et al. Long-term pain and functional disability after total knee arthroplasty with and without single-injection or continuous sciatic nerve block in addition to continuous femoral nerve block: a prospective, 1-year follow-up of a randomized controlled trial. *Reg Anesth Pain Med* 2013;38:58-63.
43. Widmer BJ, Scholes CJ, Pattullo GG, et al. Is femoral nerve block necessary during total knee arthroplasty?: a randomized controlled trial. *J Arthroplasty* 2012;27:1800-5.
44. Wu JWS, Wong YC. Elective unilateral total knee replacement using continuous femoral nerve blockade versus conventional patient-controlled analgesia: perioperative patient management based on a multidisciplinary pathway. *Hong Kong Med J* 2014;20:45-51.
45. McDonald DA, Deakin AH, Ellis BM, et al. The technique of delivery of peri-operative analgesia does not affect the rehabilitation or outcomes following total knee arthroplasty. *Bone Joint J* 2016;98-B:1189-96.

For peer review only - http://bmjopen.bmj.com/site/about/guidelines.xhtml

46. Motififard M, Omidian A, Badiei S. Pre-emptive injection of peri-articular-multimodal drug for post-operative pain management in total knee arthroplasty: a double-blind randomized clinical trial. *Int Orthop* 2017;41:939-47.

47. Niemeläinen M, Kalliovalkama J, Aho A, et al. Single periarticular local infiltration analgesia reduces opiate consumption until 48 hours after total knee arthroplasty. A randomized placebo-controlled trial involving 56 patients. *Acta Orthop* 2014;85:614-9.

48. Seah VWT, Chin PL, Chia SL, et al. Single-dose periarticular steroid infiltration for pain management in total knee arthroplasty: a prospective, double-blind, randomised controlled trial. *Singapore Med J* 2011;52:19-23.

49. Williams D, Petruccelli D, Paul J, et al. Continuous infusion of bupivacaine following total knee arthroplasty: a randomized control trial pilot study. *J Arthroplasty* 2013;28:479-84.

50. Wylde V, Lenguerrand E, Gooberman-Hill R, et al. Effect of local anaesthetic infiltration on chronic postsurgical pain after total hip and knee replacement: the APEX randomised controlled trials. *Pain* 2015;156:1161-70.

51. Meunier A, Lisander B, Good L. Effects of celecoxib on blood loss, pain, and recovery of function after total knee replacement: a randomized placebo-controlled trial. *Acta Orthop* 2007;78:661-7.

52. Aveline C, Roux AL, Hetet HL, et al. Pain and recovery after total knee arthroplasty: a 12-month follow-up after a prospective randomized study evaluating Nefopam and Ketamine for early rehabilitation. *Clin J Pain* 2014;30:749-54.

53. Buvanendran A, Kroin JS, Della Valle CJ, et al. Perioperative oral pregabalin reduces chronic pain after total knee arthroplasty: a prospective, randomized, controlled trial. *Anesth Analg* 2010;110:199-207.

54. Ejaz A, Laursen AC, Kappel A, et al. Faster recovery without the use of a tourniquet in total knee arthroplasty. *Acta Orthop* 2014;85:422-6.

55. Huang Z, Xie X, Li L, et al. Intravenous and topical tranexamic acid alone are superior to tourniquet use for primary total knee arthroplasty: A prospective, randomized controlled trial. *J Bone Joint Surg (Am)* 2017;99:2053-61.

56. Liu D, Graham D, Gillies K, et al. Effects of tourniquet use on quadriceps function and pain in total knee arthroplasty. *Knee Surg Relat Res* 2014;26:207-13.

57. Mittal R, Ko V, Adie S, et al. Tourniquet application only during cement fixation in total knee arthroplasty: a double-blind, randomized controlled trial. *ANZ J Surg* 2012;82:428-33.

For peer review only - http://bmjopen.bmj.com/site/about/guidelines.xhtml

58. Zhang Y, Li D, Liu P, et al. Effects of different methods of using pneumatic tourniquet in patients undergoing total knee arthroplasty: a randomized control trial. *Ir Jr Med Sci* 2017;186:953-9.

59. Brock T, Sprowson A, Muller S, et al. STICKS study - Short-sTretch Inelastic Compression bandage in Knee Swelling following total knee arthroplasty - a feasibility study. *Trials* 2017;18:6.

60. Hourlier H, Reina N, Fennema P. Single dose intravenous tranexamic acid as effective as continuous infusion in primary total knee arthroplasty: a randomised clinical trial. *Arch Orthop Trauma Surg* 2015;135:465-71.

61. Kim TK, Chang CB, Kang YG, et al. Clinical value of tranexamic acid in unilateral and simultaneous bilateral TKAs under a contemporary blood-saving protocol: a randomized controlled trial. *Knee Surg Sports Traumatol Arthrosc* 2014;22:1870-8.

62. Kusuma SK, Sheridan KC, Wasielewski RC. Use of bovine thrombin to reduce blood loss in primary total knee arthroplasty: a controlled randomized trial. *J Arthroplasty* 2013;28:1278-81.

63. Napier RJ, Bennett D, McConway J, et al. The influence of immediate knee flexion on blood loss and other parameters following total knee replacement. *Bone Joint J* 2014;96-B:201-9.

64. Sa-Ngasoongsong P, Channoom T, Kawinwonggowit V, et al. Postoperative blood loss reduction in computer-assisted surgery total knee replacement by low dose intra-articular tranexamic acid injection together with 2-hour clamp drain: a prospective triple-blinded randomized controlled trial. *Orthop Rev* 2011;3:e12.

65. Sa-Ngasoongsong P, Wongsak S, Chanplakorn P, et al. Efficacy of low-dose intra-articular tranexamic acid in total knee replacement; a prospective triple-blinded randomized controlled trial. *BMC Musculoskelet Disord* 2013;14:340.

66. Ledin H, Good L, Aspenberg P. Denosumab reduces early migration in total knee replacement. *Acta Orthop* 2017;88:255-8.

67. Bennett LA, Brearley SC, Hart JAL, et al. A comparison of 2 continuous passive motion protocols after total knee arthroplasty: a controlled and randomized study. *J Arthroplasty* 2005;20:225-33.

68. Ersözlü S, Sahin O, Ozgür A, et al. The effects of two different continuous passive motion protocols on knee range of motion after total knee arthroplasty: a prospective analysis. *Acta Orthop Traumato* 2009;43:412-8.

For peer review only

For peer review only - http://bmjopen.bmj.com/site/about/guidelines.xhtml

69. Avramidis K, Karachalios T, Popotonasios K, et al. Does electric stimulation of the vastus medialis muscle influence rehabilitation after total knee replacement? *Orthopedics* 2011;34:175.

70. Moretti B, Notarnicola A, Moretti L, et al. I-ONE therapy in patients undergoing total knee arthroplasty: a prospective, randomized and controlled study. *BMC Musculoskelet Disord* 2012;13:88.

71. Li L, Wang Z, Yin MH, et al. Effect of early gait training on the functional rehabilitation after total knee arthroplasty. *Chinese Journal of Tissue Engineering Research* 2017;21:4288-93.

72. Liebs T, Herzberg W, Rüther W, et al. Multicenter randomized controlled trial comparing early versus late aquatic therapy after total hip or knee arthroplasty. *Arch Phys Med Rehabil* 2012;93:192-9.

73. Mahomed NN, Davis AM, Hawker G, et al. Inpatient compared with home-based rehabilitation following primary unilateral total hip or knee replacement: a randomized controlled trial. *J Bone Joint Surg (Am)* 2008;90:1673-80.

74. Wang S, Xia J, Wei Y, et al. Effect of the knee position during wound closure after total knee arthroplasty on early knee function recovery. *J Orthop Surg Res* 2014;9:79.

75. Kong CG, Kim GH, Kim DW, et al. The effect of topical scar treatment on postoperative scar pain and pruritus after total knee arthroplasty. *Arch Orthop Trauma Surg* 2014;134:555-9.

76. Hohmann E, Tetsworth K, Hohmann S, et al. Anabolic steroids after total knee arthroplasty. A double blinded prospective pilot study. *J Orthop Surg Res* 2010;5:93.

77. Ehrich EW, Davies GM, Watson DJ, et al. Minimal perceptible clinical improvement with the Western Ontario and McMaster Universities osteoarthritis index questionnaire and global assessments in patients with osteoarthritis. *J Rheumatol* 2000;27:2635-41.

78. Beard DJ, Harris K, Dawson J, et al. Meaningful changes for the Oxford hip and knee scores after joint replacement surgery. *J Clin Epidemiol* 2015;68:73-9.

79. Lee WC, Kwan YH, Chong HC, et al. The minimal clinically important difference for Knee Society Clinical Rating System after total knee arthroplasty for primary osteoarthritis. *Knee Surg Sports Traumatol Arthrosc* 2017;25:3354-9.

80. Salaffi F, Stancati A, Silvestri CA, et al. Minimal clinically important changes in chronic musculoskeletal pain intensity measured on a numerical rating scale. *Eur J Pain* 2004;8:283-91.

81. Singh JA, Schleck C, Harmsen WS, et al. Validation of the hospital for special surgery knee questionnaire: Convergent validity, responsiveness and sensitivity to change. ACR/ARHP; San Diego, California2013. p. 1926.

For peer review only - http://bmjopen.bmj.com/site/about/guidelines.xhtml

82. Escobar A, Quintana JM, Bilbao A, et al. Responsiveness and clinically important differences for the WOMAC and SF-36 after total knee replacement. *Osteoarthritis Cart* 2007;15:273-80.

83. Danoff JR, Goel R, Sutton R, et al. How much pain is significant? Defining the minimal clinically important difference for the visual analog scale for pain after total joint arthroplasty. *J Arthroplasty* 2018;33:S71-S5.

84. Mayer M, Naylor J, Harris I, et al. Evidence base and practice variation in acute care processes for knee and hip arthroplasty surgeries. *PLOS ONE* 2017;12:e0180090.

85. Kopp SL, Borglum J, Buvanendran A, et al. Anesthesia and analgesia practice pathway options for total knee arthroplasty: An evidence-based review by the American and European Societies of Regional Anesthesia and Pain Medicine. *Reg Anesth Pain Med* 2017;42:683-97.

86. Cochrane AL. Effectiveness and efficiency: random reflections on health services. London: The Nuffield Provincial Hospitals Trust; 1972.

87. Murad MH, Asi N, Alsawas M, et al. New evidence pyramid. *Evid Based Med* 2016;21:125-7.

88. National Institute for Health and Clinical Excellence. Patient experience in adult NHS services: improving the experience of care for people using adult NHS services. London: NICE; 2012.

89. Andersen LO, Kehlet H. Analgesic efficacy of local infiltration analgesia in hip and knee arthroplasty: a systematic review. *Br J Anaesth* 2014;113:360-74.

90. Chan E-Y, Fransen M, Parker DA, et al. Femoral nerve blocks for acute postoperative pain after knee replacement surgery. *Cochrane Database Syst Rev* 2014;5:CD009941.

91. Alshryda S, Sarda P, Sukeik M, et al. Tranexamic acid in total knee replacement: a systematic review and meta-analysis. *J Bone Joint Surg (Br)* 2011;93:1577-85.

92. Poeran J, Rasul R, Suzuki S, et al. Tranexamic acid use and postoperative outcomes in patients undergoing total hip or knee arthroplasty in the United States: retrospective analysis of effectiveness and safety. *BMJ* 2014;349.

93. Hallstrom B, Singal B, Cowen ME, et al. The Michigan experience with safety and effectiveness of tranexamic acid use in hip and knee arthroplasty. *J Bone Joint Surg (Am)* 2016;98:1646-55.

94. Noordin S, McEwen JA, Kragh JF, Jr., et al. Surgical tourniquets in orthopaedics. *J Bone Joint Surg (Am)* 2009;91:2958-67.

For peer review only - http://bmjopen.bmj.com/site/about/guidelines.xhtml

95. Horlocker TT, Hebl JR, Gali B, et al. Anesthetic, patient, and surgical risk factors for neurologic complications after prolonged total tourniquet time during total knee arthroplasty. *Anesth Analg* 2006;102:950-5.

96. Harvey LA, Brosseau L, Herbert RD. Continuous passive motion following total knee arthroplasty in people with arthritis. *Cochrane Database Syst Rev* 2014;2:CD004260.

97. Januel J-M, Chen G, Ruffieux C, et al. Symptomatic in-hospital deep vein thrombosis and pulmonary embolism following hip and knee arthroplasty among patients receiving recommended prophylaxis: a systematic review. *JAMA* 2012;307:294-303.

98. Schellong SM, Beyer J, Kakkar AK, et al. Ultrasound screening for asymptomatic deep vein thrombosis after major orthopaedic surgery: The VENUS study. *J Thromb Haemost* 2007;5:1431-7.

99. Gómez-Outes A, Terleira-Fernández AI, Suárez-Gea ML, et al. Dabigatran, rivaroxaban, or apixaban versus enoxaparin for thromboprophylaxis after total hip or knee replacement: systematic review, meta-analysis, and indirect treatment comparisons. *BMJ* 2012;344:e3675.

100. Freedman KB, Brookenthal KR, Fitzgerald RH, Jr., et al. A meta-analysis of thromboembolic prophylaxis following elective total hip arthroplasty. *J Bone Joint Surg (Am)* 2000;82-a:929-38.

101. Parvizi J, Ghanem E, Joshi A, et al. Does "excessive" anticoagulation predispose to periprosthetic infection? *J Arthroplasty* 2007;22:24-8.

102. Adelani MA, Johnson SR, Keeney JA, et al. Clinical outcomes following re-admission for non-infectious wound complications after primary total knee replacement. *Bone Joint J* 2014;96-b:619-21.

103. Cahill JL, Shadbolt B, Scarvell JM, et al. Quality of life after infection in total joint replacement. *J Orthop Surg* 2008;16:58-65.

104. Akl EA, Briel M, You JJ, et al. Potential impact on estimated treatment effects of information lost to follow-up in randomised controlled trials (LOST-IT): systematic review. *BMJ* 2012;344:e2809.

105. Hawker G, Wright J, Coyte P, et al. Health-related quality of life after knee replacement - Results of the Knee Replacement Patient Outcomes Research Team Study. *J Bone Joint Surg (Am)* 1998;80A:163-73.

106. Leopold SS, Porcher R. Editorial: threshold p values in orthopaedic research—we know the problem. What is the solution? *Clin Orthop Relat Res* 2018;476:1689-91.

107. Guyatt GH, Oxman AD, Kunz R, et al. Going from evidence to recommendations. *BMJ* 2008;336:1049-51.

For peer review only - http://bmjopen.bmj.com/site/about/guidelines.xhtml

108. Lavand'homme P, Thienpont E. Pain after total knee arthroplasty: a narrative review focusing on the stratification of patients at risk for persistent pain. *Bone Joint J* 2015;97-B:45-8.

109. Simpson DM, Schifitto G, Clifford DB, et al. Pregabalin for painful HIV neuropathy: a randomized, double-blind, placebo-controlled trial. *Neurology* 2010;74:413-20.

For peer review only

For peer review only - http://bmjopen.bmj.com/site/about/guidelines.xhtml

Identification

Articles identified in November 2016 (n=7996)
Articles identified in February 2018 update (n=1701)

Screening

Articles screened (n=9697)

No relevance (n=7364)

Eligibility

Potentially relevant (n = 2333)

Intervention	N	In: low risk of bias	No pain/ score outcome	High/ unclear risk of bias	No long-term follow up	Abstract only	Additional publication	Protocol	Review	Retracted
Adenosine triphosphate	2	0	0	0	1	0	0	0	1	0
Alternative medicine	4	0	0	0	4	0	0	0	0	0
Anabolic steroids	2	1	0	0	0	0	0	0	1	0
Antibiotic prophylaxis	43	0	16	0	13	0	0	1	13	0
Assistive devices	2	0	0	0	2	0	0	0	0	0
Bisphosphonates	17	0	6	0	0	0	2	0	9	0
Blood management	355	7	10	1	209	0	0	4	124	0
Brain stimulation	3	0	0	0	3	0	0	0	0	0
Calcium supplement	1	0	0	0	1	0	0	0	0	0
Cardiovascular drugs	4	0	0	0	2	0	0	0	2	0
Chinese medicine	2	0	0	0	2	0	0	0	0	0
Cold therapy	30	0	0	1	24	0	0	0	5	0
Colloids and crystalloids	1	0	0	0	1	0	0	0	0	0
Comorbidity management	1	0	0	0	1	0	0	0	0	0
Compression	8	1	0	0	6	0	0	1	0	0
Constipation treatment	2	0	0	0	2	0	0	0	0	0
Continuous passive motion	56	2	8	7	23	1	0	1	14	0
Creatine monohydrate	1	0	0	0	1	0	0	0	0	0
Delirium prevention	4	0	0	0	3	0	0	0	1	0
Denusomab	1	1	0	0	0	0	0	0	0	0
Dexmedetomidine	1	0	0	0	1	0	0	0	0	0
Deep vein thrombosis prophylaxis	474	0	5	0	143	0	4	8	314	0
Electrical stimulation	37	2	0	3	20	0	2	0	10	0
Glucocorticoid	2	0	0	0	0	0	0	0	2	0
Glucose infusion	1	0	0	0	1	0	0	0	0	0
Guided imagery	5	0	0	1	4	0	0	0	0	0
Iron	7	0	0	0	6	0	0	0	1	0
Laser therapy	1	0	0	0	1	0	0	0	0	0
Methylprednisolone	3	0	0	0	3	0	0	0	0	0
Music therapy	9	0	0	0	9	0	0	0	0	0
Nausea prevention	11	0	0	0	9	0	2	0	0	0
Nutritional supplements	4	0	0	0	4	0	0	0	0	0
Pain management	987	20	5	12	711	1	20	9	207	2
Physiological	26	0	0	0	23	0	0	1	2	0
Platelet rich plasma	12	0	0	1	6	0	0	0	5	0
Rehabilitation	67	4	0	2	43	0	0	1	17	0
Remote ischaemic pre-conditioning	5	0	0	0	5	0	0	0	0	0
Sleep treatment	3	0	0	0	2	0	1	0	0	0
Teriparatide	1	0	1	0	0	0	0	0	0	0
Therapy dogs	1	0	0	0	1	0	0	0	0	0
Tourniquet use	100	5	3	3	67	0	2	1	19	0
Trigger point needling	1	0	0	1	0	0	0	0	0	0
Warming	19	0	0	0	16	0	0	0	3	0
Wound management	17	1	0	0	12	0	0	1	3	0
**Total**	**2333**	**44**	**54**	**32**	**1385**	**2**	**33**	**28**	**753**	**2**

For peer review only

Supplementary material. Search strategy as applied in MEDLINE on Ovid

1 randomized controlled trial/ or randomized controlled trial.pt.

2 controlled clinical trial.pt.

3 randomized.ab.

4 placebo.ab.

5 randomly.ab

6 trial.ab

7 randomised.tw

8 1 or 2 or 3 or 4 or 5 or 6 or 7

9 review/

10 'systematic review$'.mp

11 9 or 10

12 8 or 11

13 Arthroplasty, Replacement, Knee/

14 Knee Prosthesis/

15 (arthoplast$ adj3 knee$).mp. [mp=title, abstract, original title, name of substance word, subject heading word, keyword heading word, protocol supplementary concept word, rare disease supplementary concept word, unique identifier]

16 (knee$ adj3 replac$).mp. [mp=title, abstract, original title, name of substance word, subject heading word, keyword heading word, protocol supplementary concept word, rare disease supplementary concept word, unique identifier]

17 (knee adj3 implant$).mp. [mp=title, abstract, original title, name of substance word, subject heading word, keyword heading word, protocol supplementary concept word, rare disease supplementary concept word, unique identifier]

18 13 or 14 or 15 or 16 or 17

19 12 and 18

For peer review only - http://bmjopen.bmj.com/site/about/guidelines.xhtml

For peer review only

Supplementary material. All peri-operative interventions with long-term pain or score follow up

1. Pain management

**Author**<br>**Country**<br>**Recruitment dates**<br>**Setting**	**Indication**<br>**Number randomised intervention; control**<br>**Age**<br>**% female**	**Common anaesthesia**			**Follow up**<br>**Losses to follow up intervention; control**<br>**Risk of bias issues**<br>**Key results**
		**Group 1 (intervention)**	**Group 2 (intervention)**	**Group C (control)**	
FNB single vs No FNB					
Widmer et al. 2012[43]<br>Australia<br>Before 2012<br>2 surgeons	Elective primary unilateral TKR<br>27; 28<br>Median 72.1 (IQR 64.4, 76.5); 69.4 (63.4, 75.5)<br>44.4%; 44.4%	Premedication 1-3mg i.v. midazolam. Propofol induction and sevoflurane general anaesthetic.<br>LIA with 200mg ropivacaine and 0.5mg adrenaline in 100ml saline.<br>PCA 20µg fentanyl at 5-minute intervals on demand until morning POD2. Then, oral oxycodone SR 10mg every 12 hours. Daily COX II inhibitor and paracetamol 1g every 6 hours as tolerated. For breakthrough pain, 5-10mg oxycodone immediate release every 3 hours as needed.			1 year<br>No losses to follow up reported<br>Low risk of bias<br>WOMAC pain (high score favourable) at 1 year: FNB and LIA median 2.0 (IQR 0, 2.8); LIA no FNB 1.0 (0, 2.0). p=0.74<br>No adverse events occurred in either group
		Ultrasound guided FNB with 100mg ropivacaine in 30ml saline		Sham setup for FNB. No identification or injection of femoral sheath	
FNB single vs ONB vs Control					
Bergeron et al. 2009[110]<br>Canada<br>2005-2006<br>1 centre	Elective primary unilateral TKR<br>19; 20; 20<br>Mean 65.1 (SE 2.0); 72 (1.8); 67 (1.3)<br>79%; 80%; 75%	Intraoperative sedation with iv propofol at discretion of anaesthesiologist. Lumbar spinal anaesthesia with 12mg 0.5% bupivacaine.<br>Postoperative i.v. PCA with fentanyl 50µg/ml set to deliver 25µg every 5 min as needed.<br>Celecoxib 100mg and acetaminophen 650mg on arrival in recovery room and every 12 and 6 hrs respectively. Breakthrough medication with intramuscular ketorolac 10 mg every 4 hrs.			1 year<br>Overall 32 lost to follow up<br>High risk of bias: only 27/59 patients followed up due to resource limitations.<br>No difference in HSS pain at rest or during activity at 1 year between the study groups.

For peer review only - http://bmjopen.bmj.com/site/about/guidelines.xhtml

<table>
<tr><td></td><td></td><td>FNB with stimulator. 20ml 0.5% bupivacaine with 1/200,000 epinephrine.</td><td>ONB with stimulator. 20ml 0.5% bupivacaine with 1/200,000 epinephrine.</td><td>No injection but inguinal area prepared, and sham block performed behind drapes.</td><td>No long-term complications attributable to anaesthetic regimen</td></tr>
<tr><td colspan="6">FNB continuous low dose vs FNB continuous high dose vs No FNB</td></tr>
<tr><td rowspan="2">Shum et al. 2009[111]
Singapore
Before 2009
1 hospital</td><td rowspan="2">Unilateral TKR for osteoarthritis
20 (17 received treatment); 20 (18 received treatment); 20
Mean 66.7 (SD 8.4); 65.4 (8.4); 67.8 (5.5)
88%; 72%; 80%</td><td colspan="3">Spinal anaesthesia induced with 2-3ml hyperbaric 0.5% bupivacaine. Intraoperative sedation with midazolam in increments of 0.5mg.
Intravenous PCA morphine (1mg/ml, on-demand bolus doses of 1 mg with 5 minute lockout, maximum dose 8 mg/hr)</td><td rowspan="2">2 years
16.4% of patients who received intervention lost to follow up
Unclear risk of bias due to differences in OKS and weight at baseline, and limited methodological details.
No separate pain outcome but mean OKS slightly more favourable in group with no FNB, 18.2 (SD 3.7) compared with combined FNB groups, 19.8 (5.4) but this was not significant.
No complications attributable to use of FNB</td></tr>
<tr><td>Low dose continuous FNB at conclusion of TKR with ropivacaine 0.15% (10 ml/hr in the first 24 hours, followed by 5ml/hr in the next 24 hours)</td><td>High dose continuous FNB at conclusion of TKR with ropivacaine 0.2% (10 ml/hr in the first 24 hours, followed by 5 ml/hr in the next 24 hours)</td><td>No FNB</td></tr>
<tr><td colspan="6">SNB injection vs SNB continuous vs control</td></tr>
<tr><td>Wegener et al. 2013[42]
The Netherlands
2008-2010
1 centre</td><td>Unilateral TKR
29; 30; 30 (90 randomised)
Median 65 (range 43-81); 66 (43-83); 62 (50-79)
62%; 70%; 73%</td><td colspan="3">Lorazepam 1mg 2 hours and acetaminophen 2g 1 hour before surgery. FNB with stimulating catheter: loading dose 20 ml levobupivacaine 0.375% and after 45 minutes a continuous infusion of levobupivacaine 0.125% 10 ml/hr. General anaesthesia induced with 3-5 µg/ml propofol infusion and remifentanil 0.5 µg/kg/min and maintained with 2-3 µg/ml at 0.1-0.25 µg/kg/min. Postoperatively, FNB changed to patient controlled FNB, 5ml bolus, 30-minute lockout; basal rate 6 ml/hr. i.v. morphine administered if needed. Postoperative analgesia with acetaminophen 1g 4 times daily. Diclofenac 50mg or tramadol 50mg 3 times daily. Tramadol 100mg before removal of nerve catheters. Morphine pain relief as required.</td><td>12 months
2;7;5 lost to follow up
Low risk of bias
Median WOMAC pain scores at 12 months: SNB injection 80 (range 25-100), SNB continuous 90 (55-100) and PCA only 90 (35-100), p=0.81. No difference between groups in VAS pain at rest (p=0.90) or during mobilisation (p=0.43).
No information on adverse events.</td></tr>
</table>

For peer review only

For peer review only - http://bmjopen.bmj.com/site/about/guidelines.xhtml

		Group Fs: SNB single injection. SNB loading dose of 20 ml levobupivacaine 0.375%.	Group FCS: SNB continuous infusion. SNB loading dose of 20 ml levobupivacaine 0.375%. Continuous infusion of levobupivacaine 0.125% 10 ml/hr started 45 mins after catheter placement. SNB maintained for 36 hours postoperatively (10 ml/hr).	Group F: No SNB. PCA via femoral nerve catheter	
**General anaesthesia vs FNB single vs FNB/ SNB single**					
Gao et al. 2017[37]<br>China<br>2014-2015<br>1 centre	Primary unilateral TKR for osteoarthritis<br>50; 50; 50<br>Mean 65.8 (SD 6.7); 66.4 (7.4); 67.6 (6.3)<br>81%; 80%; 76%	Pre-operative and post-operative celecoxib 0.2g twice daily.<br>100ml intra-operative LIA with ropivacaine 200mg and epinephrine 0.25 mg.			6 months<br>2; 1; 0<br>Low risk of bias<br>Mean HSS at 6 months: 87.1 (SD 6.9); 87.4 (7.3); 88.5 (6.7). No significant difference.<br>Nausea and vomiting: 4; 2; 1, urinary retention: 3; 1; 2.
		General anaesthesia	Ultrasound guided FNB 5g/l ropivacaine 20ml plus 0.1mg epinephrine	Ultrasound guided FNB 5g/l ropivacaine 20 ml plus 0.1mg epinephrine and SNB 5g/l ropivacaine 20ml plus 0.1mg epinephrine	
LIA no corticosteroid vs No LIA/ placebo					
Wylde et al. 2015 [50]<br>UK	Primary unilateral TKR for osteoarthritis	FNB with nerve stimulator and/ or ultrasound guidance (20ml 0.25% bupivacaine). Spinal or general anaesthetic. Intra-operative analgesia provided by titration of i.v. fentanyl initially and morphine if necessary. 1g intravenous			6 and 12 months<br>24;19 at 12 months (including those who did not receive treatment)

For peer review only

For peer review only - http://bmjopen.bmj.com/site/about/guidelines.xhtml

<table>
<tr><td>2009-2012
1 centre</td><td>157; 159 (143; 137 received treatment)
Mean 69.5 (SD 9.4); 68.7 (7.9)
52%; 54%</td><td colspan="2">paracetamol 30 minutes before the end of operation. Immediately post-operative 400mg oral ibuprofen.
PCA with morphine 1mg/ml, 1 mg bolus dose and a 5-minute lock-out. If necessary morphine bolus up to 0.2mg/kg as rescue analgesia. During hospital stay, visit from pain specialist nurse. Oral or i.v. paracetamol every 6 hours and ibuprofen 400mg every 8 hours. When PCA no longer needed, oral codeine phosphate 30-60mg every 6 hours, tramadol 50-100mg every 6 hours and oramorph 10-20mg as rescue analgesia.</td><td rowspan="2">Low risk of bias
At 12 months WOMAC pain score (0-100) in LIA group median 90 (IQR 30), Control 85 (35); ITT-CC linear regression coefficient 3.83 (95%CI -0.83, 8.49), p=0.107. At 6 months WOMAC pain score ITT-CC linear regression coefficient 4.10 (95%CI -0.22, 8.43), p=0.063. Mean differences lower than MCID of 8-9[77].
Superficial and deep wound infection rate in LIA group 3.2% and 1.9% in control group, p=0.500. No differences in serious adverse events between groups</td></tr>
<tr><td></td><td></td><td>60ml intra-operative LIA with 0.25% bupivacaine and 1/200,000 adrenaline injected into the posterior capsule, medial and lateral capsule, fascia and muscle, and subcutaneous tissues.</td><td>No treatment other than standard care</td></tr>
<tr><td>Williams et al. 2013[49]
Canada
Before 2013
1 centre, 2 surgeons</td><td>Primary unilateral TKR for osteoarthritis
35; 32 (26; 25 received treatment)
Mean 66 (SD 9.7); 67 (12.5)
58%; 60%</td><td colspan="2">Sedation with i.v. midazolam and propofol. Intraoperative LIA loading dose of 20ml 0.25% bupivacaine/ epinephrine injection, 10ml into medial and lateral subcutaneous tissue around the incision and 10ml intra-articular after closure. Infiltrate delivered by pain pump into lateral recess of intra-articular space. Spinal anaesthetic with 10-15 mg of 0.75% or 0.5% plain bupivacaine and 20μg fentanyl.
Postoperative morphine PCA. 7.5mg i.v ketorolac preoperatively plus 15mg every 6 hours postoperatively for 48 hours, then oral ketorolac 10mg every 6 hours for 2 days. Gabapentin 600mg given preoperatively plus 300mg twice daily for 48 hours postoperatively. Oxycodone 10mg twice daily for 48 hours postoperative. Oral paracetamol 650mg every 4 hours for 72 hours.</td><td rowspan="2">6 and 12 months
3;1 of those who received treatment
Low risk of bias
Mean VAS pain score at 6 months 1.2 (SD 1.3); 1.2 (1.2). p=0.836. At 12 months 0.9 (1.2); 1.0 (1.1). p=0.767
No short-term differences in adverse events except control patients more likely to be drowsy at 48 hrs. Long-term adverse events not reported.</td></tr>
<tr><td></td><td></td><td>Infusion of 0.5% bupivacaine at 2ml/hr for 48 hrs</td><td>Infusion of saline at 2ml/hr for 48 hrs</td></tr>
<tr><td>Niemeläinen et al. 2014[47]
Finland
2011-2012</td><td>Primary unilateral TKR for osteoarthritis
30; 30 (27; 29 received treatment)</td><td colspan="2">Oral paracetamol 1g given 1 hour before surgery. Spinal anaesthesia with 15mg bupivacaine in 3ml.
After surgery oral paracetamol 1g every 6 hours and oral meloxicam (15mg) every 24 hours.
PCA with oxycodone 2mg, lock-out time 8 min.</td><td>12 months
1; 4
Low risk of bias
No pain measure separate from OKS. Weak evidence of more favourable</td></tr>
</table>

For peer review only

For peer review only - http://bmjopen.bmj.com/site/about/guidelines.xhtml

1 hospital	Mean 65 (SD 4.9); 64 (6.7)<br>56%; 48%	Rescue levobupivacaine medication through a lumbar epidural catheter		OKS (0-48) in the LIA group at 12 months, mean difference -2.7 (95% CI -5.48, 0.07). Difference lower than MCID of 4.0[78].<br>Infection: 0; 0. Severe pain treated with epidural analgesia: 0; 3. Nausea: 1; 1
		Intra-operative periarticular LIA of 100ml saline with levobupivacaine (150mg) mixed with ketorolac (30mg) and adrenaline (0.5mg).	Intra-operative periarticular LIA of 100ml saline	
Motififard et al. 2017[46]<br>Iran<br>2014-2015<br>1 hospital	Primary unilateral TKR for osteoarthritis<br>60; 60<br>Mean 66.4 (6.4); 64.5 (6.0)<br>86.0%; 94.3%	Spinal anaesthesia.<br>No FNB or SNB.<br>Pain medication provided as required after surgery: meloxicam (15 mg daily), celecoxib (400 mg daily), acetaminophen (1g every 8 hours), tramadol (50 mg every 8 hours), ketorolac (30 mg slow IV every 8 hours, with a 4-dose max), and morphine (5–10 mg slow IV if needed)		6 months<br>3; 7<br>Low risk of bias<br>No separate pain measure. Weak evidence for improved KSS (0-200) in LIA group at 6 months, mean 115.55 (SD 15.506); 101.40 (16.117). P=0.07. Difference of 14.15 greater than MCID of 12.3[79]. Difference was significant at 6 weeks, p<0.001.<br>No complications related to TKR or LIA. Low back pain (1; 2), stroke (0; 1), CHF (1; 0)
		Peri-articular injection, 15 minutes before incision, of 100ml saline containing 50 mg bupivacaine hydrochloride 0.5%, 1 ml morphine sulphate 10 mg/ml, 300 µg epinephrine (1:1000) and 30 mg ketorolac	100ml saline containing 300 µg epinephrine (1:1000)	
McDonald et al. 2016[45]<br>UK<br>2010-2011<br>1 hospital	Primary unilateral TKR for osteoarthritis<br>113; 109 received common spinal anaesthesia (121; 121 randomised)<br>Median 68 (IQR 62, 72); 67 (62, 73)<br>59%; 55%	Oral premedication with 10-20mg temazepam, 150mg ranatidine, 10mg dexamethasone, 300mg gabapentin, 1g paracetamol.<br>Spinal anaesthesia		12 months<br>9; 11 of those receiving treatments<br>Low risk of bias<br>No separate pain outcome. Mean OKS at 12 months: median 41 (IQR 35, 44); 41 (34;44). P=0.915<br>Suspected infection 2; 1. MI 0; 1. GI bleed 1; 0. renal failure 1; 0. Died 2; 0)
		Intra-articular and subcutaneous infiltration during surgery of 200 ml of 2mg/ml ropivacaine without adrenalin or additives. Catheter inserted, and 20 ml infiltrate injected following wound closure. Further boluses of 40 ml 2 mg/ml ropivacaine via infusion pump 4 hours after leaving theatre and morning of POD1. Two	Epidural PCA with 4 ml of 2.5 mg/ml levobupivacaine introduced at end of surgery. Thereafter self-medication with 2 ml of 1.25 mg/ml bupivacaine with 15 minutes lockout until morning of POD1. Nurse-administered rescue of 4 ml of 2.5 mg/ml levobupivacaine.	

For peer review only - http://bmjopen.bmj.com/site/about/guidelines.xhtml

		additional top ups of 40 ml 2mg/ml ropivacaine were prescribed if required.		
Celecoxib vs placebo				
Meunier et al. 2007[51]<br>Sweden<br>2004-2005<br>1 centre	Elective primary unilateral TKR for osteoarthritis<br>25; 25 (24; 20 received treatment)<br>Mean 68 (SD 6.3); 69 (7.7)<br>71%; 40%	Spinal anaesthesia with bupivacaine 17.5-20mg. i.v. midazolam or propofol sedation if needed. Paracetamol 1 g preoperatively and then with tramadol 50-100 mg 4 times a day during hospital stay. Ketobemidone (2.5-5mg i.v. or subcutaneous) on demand. Paracetamol and tramadol used as required after discharge.		12 months<br>No losses to follow up after surgery reported<br>Low risk of bias<br>No effect of celecoxib on VAS or KOOS pain at 1 year.<br>DVT: 0; 1. Deep infection: 0; 0.
		Oral celecoxib 200mg 1 hour preoperatively and twice daily for 3 weeks	Oral placebo 200mg 1 hour preoperatively and twice daily for 3 weeks	
Ketamine vs placebo				
Perrin and Purcell2009 [112]<br>Australia<br>Before 2009<br>1 centre (pilot study)	Elective unilateral TKR<br>16 (5; 7 completed study per protocol)<br>Mean 65.6 (SD 10.2); 60.3 (11.9)<br>40%; 43%	Intrathecal injection of 15mg bupivacaine and 100µg morphine. General anaesthesia. After surgery 1.5g paracetamol and then 750mg every 4 hours; PCA with morphine 2mg boluses with 10-minute lockout; morphine rescue 2.5mg intravenously as required; and rescue oral ibuprofen 800mg.		6 months<br>3 protocol breaches and 1 patient with uncontrolled pain.<br>High risk of bias due to non-ITT reporting and recruitment difficulties<br>2/5 ketamine group had mild/moderate pain on the WOMAC pain scale at 26 weeks or failed to improve compared with 5/7 controls.<br>1 adverse psycho-mimetic effect not attributed to intervention or control treatment
		Ketamine 0.5mg/kg bolus followed by 4µg/kg/min infusion. Commenced before surgical incision and continued until wound bandaged or syringe empty.	Saline infusion. Commenced before surgical incision and continued until wound bandaged or syringe empty.	
Ketamine vs Nefopam vs placebo				
Aveline et al. 2014[52]<br>France<br>2005<br>1 centre	Elective primary unilateral TKR for osteoarthritis<br>25; 25; 25<br>Mean 73 (SD 9); 72 (9); 70 (7)<br>67%; 60%; 63%	General anaesthesia induced with 1.5-2mg/kg propofol, 1µ/kg remifentanil and a single bolus of cisatracurium 0.15mg/kg. Remifentanil infusion at 0.15µg/k/min until skin closure. Anaesthesia maintained with sevoflurane 0.9-1.2% with 50% nitrogen in oxygen. 20 mins before skin closure, 0.15mg/kg i.v. morphine bolus and 0.625mg droperidol. PCA with morphine hydrochloride 1 mg i.v. bolus with 7-min lockout. On arrival in recovery room, 3 mg i.v. morphine boluses at 5 minute intervals.		6 and 12 months<br>3; 1; 2<br>Low risk of bias<br>Median DN4 at 12 months: 1 (IQR 1, 2); 1 (0, 1); 2 (1, 3). p=0.02 for difference between ketamine and placebo groups. Number of patients with VAS pain on movement score

For peer review only - http://bmjopen.bmj.com/site/about/guidelines.xhtml

		0.2mg/kg nefopam administered over 20 min before incision; 2mg/ml nefopam continuous infusion at 120µg/kg/hr until end of surgery and 60µg/kg/hr for 48 hours	0.2mg/kg ketamine administered over 20 min before incision; 2mg/ml ketamine continuous infusion at 120µg/kg/hr until end of surgery and 60µg/kg/hr for 48 hours	Saline administered over 20 minutes before incision; saline continuous infusion until second post-operative day	≥40mm at 12 months by group: nefopam (3/22, 13.7%), ketamine (3/24, 12.5%), and placebo group (6/23, 26.1%). Ketamine reduced DN4 pain (p=0.02) compared with placebo. At 12 months only 7/69 patients had DN4≥4 indicative of neuropathic pain.<br>Infection: 0; 0; 0. Revision: 0; 0; 0.
Pregabalin vs placebo					
Buvanendran et al. 2010[53]<br>USA<br>2006-2007<br>Single centre	Primary unilateral TKR for osteoarthritis.<br>120; 120 (9; 2 did not receive post-operative treatment but ITT analysis)<br>Mean 64.0 (SD 8.3); 63.3 (8.9)<br>76%; 70%	Sedation with midazolam and i.v. propofol. Combined spinal-epidural anaesthetic. 1.5ml 0.75% hyperbaric bupivacaine with 25µg fentanyl injected intrathecally. Catheter inserted for epidural drug administration.<br>LIA 60 ml 0.25% bupivacaine with epinephrine infiltrated into the wound at capsule closure. From completion of surgery until 32-42 hours post-operative, epidural infusion of fentanyl (5µg/ml) and bupivacaine (1mg/ml) initiated using continuous basal infusion of 6ml/hr with epidural PCA bolus doses (maximum 10ml/hr). Patients transitioned to oral opioid (morphine, oxycodone, and hydromorphone) as required. All patients received preoperative oral celecoxib 400mg 1–2 hours before surgery and 200mg twice daily for 3 days in hospital.			6 months<br>7; 5<br>Low risk of bias<br>Mean VRS pain score at 6 months: pregabalin 0.41 (SD 1.20); control 0.95 (1.80). p=0.0084. Distributions skewed but nonparametric Wilcoxon significant (p=0.0176). Difference of 0.54 less than MCID of 1.0.<br>In the pregabalin group the incidence of neuropathic pain measured using S-LANSS was 0% (0/113) and 5.2% (6/115) in the placebo group (p=0.014).<br>No clinically significant adverse events up to 6 months and no falls. Sedation, confusion and dry mouth more frequent in pregabalin than placebo group on day of surgery and first postoperative day.
		Oral pregabalin 300mg 1–2 h before surgery, 150mg twice daily for the first 10 postoperative days, 75mg twice daily on days 11 and 12, and 50mg twice daily on days 13 and 14	Oral placebo 1–2 h before surgery, twice daily for the first 10 postoperative days, twice daily on days 11 and 12, and twice daily on days 13 and 14		
FNB long duration vs FNB short duration					

For peer review only

For peer review only - http://bmjopen.bmj.com/site/about/guidelines.xhtml

<table>
<tr>
<td rowspan="2">Ilfeld et al. 2009[113]
USA
2005-2007
2 centres</td>
<td rowspan="2">Primary unilateral TKR for osteoarthritis
25; 25
Median 66 (IQR 60, 70); 64 (60, 69)
56%; 60%</td>
<td colspan="2">Femoral catheter inserted using nerve stimulator. FNB with 0.2% ropivacaine infusion (8ml/hr basal; 4 ml patient-controlled bolus; 30-minute lockout) from surgery until a.m. POD1.
1 week oral acetaminophen (975mg every 6 hours), sustained release oral opioid (Oxycontin, 10mg every 12 hours), and either oral aspirin (650mg daily) or celecoxib (200mg every 12 hours). Oral oxycodone 5 mg tablets and/ or i.v. morphine sulfate 2-4 mg for breakthrough pain.</td>
<td rowspan="2">6 and 12 months
4; 1 lost to follow up
High risk of bias: uneven loss to follow up between groups; muscle weakness resulted in lower dose of infusion on POD1 (10 continuous; 3 saline)
Groups had similar WOMAC pain scores at 6 and 12 months (p>0.05).
MI: 1; 0. PE: 1; 0. Fall: 1; 0. Catheter leak, dislodged: 1; 2</td>
</tr>
<tr>
<td>At 6 a.m. POD1, infusion pump replaced with infusion pump with 0.2% ropivacaine.
At 6 p.m. POD2 pump replaced with portable infusion pump (400ml 0.2% ropivacaine). Catheter removed evening of POD4.</td>
<td>At 6 a.m. POD1, infusion pump replaced but saline substituted.
At 6 p.m. POD2 pump replaced with portable infusion pump (saline). Catheter removed evening of POD4</td>
</tr>
<tr>
<td rowspan="2">Ilfeld et al. 2011[114]
USA
2007-2009
2 centres</td>
<td rowspan="2">Primary unilateral cemented TKR for osteoarthritis
40; 40 (39; 38 included in RCT)
Median 61 (IQR 58, 67); 66 (60, 70)
67%; 66%</td>
<td colspan="2">Femoral catheter inserted using nerve stimulator. FNB with 0.2% ropivacaine infusion (6ml/hr basal; 4ml patient-controlled bolus; 30-min lockout) from surgery until POD1.
1 week oral acetaminophen (975mg every 6 hours), sustained release oral opioid (Oxycontin, 10mg every 12 hours), and either oral aspirin (650mg daily) or celecoxib (200mg every 12 hours). Oral (oxycodone 5mg or 10mg tablets) and/ or i.v. opioids (morphine sulfate 2-4mg) for breakthrough pain.</td>
<td rowspan="2">12 months
11; 12 incomplete follow up
High risk of bias: 11;12 did not have 4 measures out of 6 up to 12 months; graph suggests WOMAC pain lower pre-intervention in continuous infusion group.
No difference in WOMAC pain scores between randomised groups (p>0.05).
Falls: 4; 0</td>
</tr>
<tr>
<td>At 6 a.m. POD2, infusion pump replaced and 0.2% ropivacaine continued.
At 6 p.m. POD2 pump replaced with portable infusion pump (400ml 0.2% ropivacaine). Catheter removed evening of POD4</td>
<td>At 6 a.m. POD2, infusion pump replaced but saline substituted.
At 6 p.m. POD2 pump replaced with portable infusion pump (saline). Catheter removed evening of POD4</td>
</tr>
<tr>
<td>Choy et al. 2011[35]
Korea
2006-2007</td>
<td>Primary unilateral TKR for osteoarthritis</td>
<td colspan="2">Spinal anaesthesia. Continuous FNB via catheter until POD3. Catheter inserted with use of nerve stimulator. Analgesia induced with 20ml of 1:1 0.25% bupivacaine and 2% lidocaine with 1:200,000 epinephrine. Continuous</td>
<td>2 years
4; 3 lost to follow up</td>
</tr>
</table>

For peer review only - http://bmjopen.bmj.com/site/about/guidelines.xhtml

<table>
<tr><td rowspan="2">1 surgeon</td><td rowspan="2">33; 30 (2 patients received GA and excluded)
Mean 66.7 (SD 10); 67.5 (11)
97%; 93%</td><td colspan="3">infusion with 0.125% bupivacaine 5.0ml/hr. i.v. PCA (butorphanol 4mg, ketorolac 150mg, saline 50ml), programmed to deliver 1 mg bolus (lockout 10 min) with maximum dose 6mg/hr. i.v. paracetamol 2g 4 times/ day and oral ibuprofen 600mg 3 times/ day for breakthrough pain</td><td rowspan="2">Low risk of bias for 2 year outcome measures.
At 2 years, intervention WOMAC pain mean 7.2 (SD 2), control 6.3 (SD 1); p=0.2
Superficial infection: 1; 1</td></tr>
<tr><td colspan="2">Continuous femoral nerve block via catheter continued from POD3 to POD7</td><td>Continuous femoral nerve block discontinued on POD3</td></tr>
<tr><td colspan="6">FNB continuous high concentration vs FNB low concentration vs FNB single</td></tr>
<tr><td rowspan="2">Albrecht et al. 2014[34]
Canada
2009-2011
1 hospital</td><td rowspan="2">Scheduled primary unilateral TKR
32; 32; 35
Mean 61 (CI 57, 64); 63 (60, 67);63 (60, 66)
46%; 44%; 52%</td><td colspan="3">Stimulating catheter inserted with ultrasound guidance. Immediately after catheter placement, 10ml mepivacaine 2% was injected through the catheter. SNB using 30 ml ropivacaine 0.2%. Spinal anaesthesia with 2.5 to 3.0 ml isobaric bupivacaine 0.5% and 0.1mg intrathecal morphine.</td><td rowspan="2">12 months
4;0;2 lost to follow up
Low risk of bias.
No separate pain outcome. Mean WOMAC score at 12 months: high concentration FNB 17 (95% CI 7, 27); 22 (14, 30); 18 (8, 27). P=0.68
Falls: 0; 0; 1</td></tr>
<tr><td>Bolus of 20ml ropivacaine 0.2% with epinephrine 1:400,000 into the femoral catheter followed by ropivacaine 0.2% at a rate of 5 ml/hr with patient-controlled boluses of 5ml available every 30minutes.</td><td>Bolus of 20 ml ropivacaine 0.2% with epinephrine 1:400,000 into femoral catheter followed by ropivacaine 0.1% at rate of 10ml/hr with patient-controlled boluses of 10 ml available every 30 minutes.</td><td>Bolus of 30ml ropivacaine 0.375% with epinephrine 1:400,000 into the femoral catheter followed by normal saline at a rate of 1 ml/hr with patient-controlled boluses of 1mL available every 30minutes.</td></tr>
<tr><td colspan="6">FNB continuous vs Psoas compartment block vs FNB continuous and psoas compartment block</td></tr>
<tr><td>Morin et al. 2005[115]
Germany
Before 2005
1 centre</td><td>Elective unilateral TKR
30; 30; 30</td><td colspan="3">Oral pre-medication with 20mg chlorazepate. General anaesthesia with intravenous propofol and 4–8µg/kg i.v. fentanyl and desflurane in N2O. 100mg diclofenac suppository after anaesthesia induction and 2.5g intravenous metamizole before end of surgery. Post-operative 3 daily doses of oral diclofenac 50mg. i.v. PCA</td><td>9–12 months
7; 6; 5
High risk of bias due to large losses to follow up, non-blinded outcome collection, and differences between</td></tr>
</table>

For peer review only - http://bmjopen.bmj.com/site/about/guidelines.xhtml

	Median 68 (IQR 62, 74); 71 (63, 74); 65 (53, 73)<br>50%; 70%; 59%	with piritramide bolus 2mg as needed with lockout interval of 10 mins for 48 hours.			groups in BMI and anaesthetist's opinion of difficulty of catheter placement.<br>No difference between groups in level of pain at the knee joint during past 4 weeks: FNB median 2.5 (IQR 1, 4), FNB and SNB 2 (1, 4), Psoas block 2 (IQR 1, 4), p=0.44<br>No early complications but longer term adverse events not reported.
		Continuous FNB<br>Stimulating catheter used. Initial bolus of prilocaine 1% and ropivacaine 0.75%. 300mg prilocaine 1% (30ml) and 150mg ropivacaine 0.75% (20ml). During first 48hrs post-operative ropivacaine 0.2% infusion 14ml/hr.	Continuous FNB and continuous SNB<br>Stimulating catheter used. Initial bolus of prilocaine 1% and ropivacaine 0.75%. In each catheter: 200mg prilocaine 1% (20ml) and 75mg ropivacaine 0.75% (10ml). During first 48hrs post-operative infusion through each catheter of 0.2% ropivacaine 7ml/hr.	Continuous psoas compartment block<br>Stimulating catheter used. Initial bolus of prilocaine 1% and ropivacaine 0.75%. 300mg prilocaine 1% (30ml) and 150mg ropivacaine 0.75% (20ml). During first 48hrs post-operative ropivacaine 0.2% infusion 14ml/hr.	
ACB continuous vs FNB continuous					
Davidson et al. 2016[116]<br>USA<br>2013-2014<br>2 studies combined from 1 centre	Primary, unilateral TKR or unicompartmental<br>54 (39 TKR, 16 UKR); 56 (41 TKR, 15 UKR)<br>TKR mean 67 (SD 8); 66 (7). UKR 70 (10); 68 (12)	Spinal or general anaesthesia. Intra-operative i.v. fentanyl, hydromorphone, and/or morphine.<br>LIA with 30 ml ropivacaine (0.5%), ketorolac (30 mg), and epinephrine (5 µg/ml).<br>Post-operative: oral acetaminophen (975 mg every 6 hr), celecoxib (200 mg every 12 hr), and sustained release oxycodone (10 mg every 12 hr). For breakthrough pain, infusion pump bolus (4 ml, 30-min lock-out). Rescue opioid titrated to pain severity. 10 ml lidocaine (2%) bolus was given via the perineural catheter for moderate or severe pain.			12 months<br>31; 29<br>High risk of bias due to partial follow up<br>TKR and UKR combined. Pain score (0-10) at 12 months median 0.0 (IQR 0.0, 3.0); 0.5 (0.0, 2.0). P=0.80). Pain score >0: 35%; 32%. P=0.65. No difference at 4 months when follow up more complete (51;

For peer review only

For peer review only - http://bmjopen.bmj.com/site/about/guidelines.xhtml

<table>
<tr><td></td><td>TKR 59%; 66%.
UKR 47%; 47%</td><td>Ultrasound guided ACB. Ropivacaine 0.2% at basal rate of 6 ml/hr, a 4-ml bolus, and a lockout of 30 minutes</td><td>Ultrasound guided continuous FNB. Ropivacaine 0.2% at basal rate of 6 ml/hr, a 4-ml bolus, and a lockout of 30 minutes</td><td>52) in pain score (p=0.80) or pain score >0 (p=0.48).
Falls in hospital: 2; 5</td></tr>
<tr><td colspan="5">ACB single vs FNB single</td></tr>
<tr><td rowspan="2">Macrinici et al. 2017[38]
USA
Before 2017
1 centre</td><td rowspan="2">Primary unilateral TKR, indication not specified (selected by the surgeon for TKA)
49; 49
Mean 67 (SD 8); 67 (8)
61%; 63%</td><td colspan="2">Multimodal regimen including NSAIDs, non-opioid analgesics, opioids. LIA 40ml Marcaine 0.25%.
All patients received an ultrasound guided needle insertion into ACB and FNB sites.</td><td rowspan="2">6 months
3; 4 lost to follow up. 6; 3 complications
Low risk of bias
VAS pain similar between groups at 6 months. No difference in functional outcomes
Medical complications: 3; 0. Surgical complication: 0; 1. Temporary foot drop: 3; 2.</td></tr>
<tr><td>Immediately after surgery, 30ml solution with 100ml Marcaine into ACB site. 30 ml saline into FNB site</td><td>Immediately after surgery, 30ml solution with 100ml Marcaine into FNB site. 30 ml saline into ACB site</td></tr>
<tr><td colspan="5">FNB continuous vs oral opioid</td></tr>
<tr><td rowspan="2">Nader et al. 2012[39]
USA
2007-2008
1 surgeon</td><td rowspan="2">Elective unilateral TKR
31; 31
Median (IQR) 65 (60, 76); 64 (60, 71)
58%; 77%</td><td colspan="2">Before surgery, patients received 1–2mg midazolam as needed. Epidural with 10mg 0.5% isobaric bupivacaine injected intrathecally. Intraoperative sedation with propofol infusion of 25-75mcg/kg/minute. In post-anesthesia recovery area, PCA epidural with basal infusion of 3 ml/hr (1 mg/ml bupivacaine and 10 mg/ml hydromorphone) with patient-activated boluses of 3 ml with a lockout interval of 15 minutes and per hour maximum of 15 ml. Infusion discontinued and epidural catheter removed on morning of POD 1. All subjects received 5 mg warfarin on evening of surgery and 40 mg enoxaparin starting on POD 1</td><td rowspan="2">6 and 12 months
1; 1 lost to follow up at 12 months
Low risk of bias
No difference in overall median NRS pain score at 6 months and 12 months: 0 (IQR 0, 1); 0 (0, 1). p=1.0. At 12 months, some evidence favouring hydrocodone for pain ascending/ descending stairs: 1 (0, 2); 0 (0, 0). p=0.01. Also, suggestion of reduced pain in hydrocodone group at night in bed (p=0.06) and sitting/ lying (p=0.07), standing upright (p=0.10). No difference walking on flat surface (p=0.41).
Falls in month after surgery: 1;0. Positive joint aspirate: 3; 0. VTE: 0; 4.</td></tr>
<tr><td>Continuous FNB inserted with use of stimulator. After discontinuation of epidural anaesthesia on the morning of POD1 10mL bolus of ropivacaine 0.25% followed by 5ml/h infusion of 0.1% ropivacaine. On morning of POD 2, ropivacaine infusion</td><td>10 mg oral hydrocodone plus 325mg acetaminophen every 4-6 hours administered for pain as needed. Sustained release oxycodone 10mg for 12 hours with oral hydromorphone 2 mg over</td></tr>
</table>

For peer review only - http://bmjopen.bmj.com/site/about/guidelines.xhtml

		discontinued. Femoral catheter removed 24 hours after previous dose of enoxaparin.	4 hours for breakthrough pain	
FNB continuous vs PCA				
Wang et al. 2015[117]<br>China<br>2012-2013<br>3 centres	Elective unilateral TKR<br>82; 86<br>No significant differences in age or sex	General anaesthesia with midazolam (0.02-0.04mg/kg), fentanyl (1µg/kg), propofol (1-2mg/kg) and cisatracurium (0.15mg/kg). Anaesthesia maintained with sevoflurane during surgery. Intramuscular injection with 10mg metoclopramide and 2.5mg droperidol 30 minutes before surgery. Post-surgery, celocoxib and parecoxib 40mg for patients with severe pain, and i.v. morphine if needed.		6 and 12 months<br>2; 4 lost to follow up at 12 months<br>Unclear risk of bias: limited reporting of randomisation methods.<br>No differences were observed between groups at 6 or 12 months for any HSS domain including pain.<br>No nerve injuries
		Continuous FNB with ultrasound stimulator. After surgery, 0.2% ropivacaine (20ml) injected through catheter. Then an analgesia pump was attached delivering 0.2% ropivacaine 8ml/hr.	Epidural PCA 0.2% ropivacaine was injected at a rate of 5 ml/hr in a 2ml pulse dose	
Peng et al. 2014[40]<br>China<br>Before 2014<br>1 centre (2 surgical teams with 4 surgeons and 2 anaesthesiologists)	Primary unilateral TKR<br>140;140<br>Mean: 66.8 (SD 9.4); 68.0 (SD 11.2)<br>73%; 65%	General intravenous and inhalational anaesthesia: midazolam 0.1-0.15mg/kg (etomidate 0.15-0.2mg/kg for patients >65 years), propofol 2.0-2.5mg/kg, sufentanil citrate 0.3-1.0µg/kg, and vecuronium 0.08-0.12mg/kg for induction of anaesthesia. Maintenance with inhalation of 1%-3% sevoflurane and continuous intravenous infusion of remifentanil 7-8µg/kg/hr and propofol 25-75µg/kg/min. After wound closure, 5-10µg intravenous sufentanil and loading dose of PCA injected. i.v. injection of 4mg ondansetron.		6 and 12 months<br>31; 38 at 12 months<br>Low risk of bias<br>Chronic post-operative pain (NRS 1+) in 38.5% of PCA group at 6 months compared with 25.7% in FNB group (p=0.021). No difference at 12 months (p=0.273).<br>Authors only reported short term adverse events associated with use of PCA.
		FNB with ultrasound guidance. Initial dose of 10ml 2% lidocaine and 10ml 1% ropivacaine. 30 minutes before end of operation, catheter connected to PCA pump; patients received loading dose of 5ml of 0.15% ropivacaine followed by infusion of 0.15% ropivacaine at 5ml/hr, with bolus of 5mL	i.v. PCA with tramadol 800mg, flurbiprofen axetil 100mg, and dexamethasone 5mg with saline to a volume of 80ml. Loading dose of 2ml followed by an infusion rate of 1 ml/hr with bolus of 2 ml. Lock time 15min.	

For peer review only - http://bmjopen.bmj.com/site/about/guidelines.xhtml

		and lock time of 30 min. Preoperatively, a loading dose of 30ml was injected for intraoperative analgesia.		
Wu and Wong 2014[44]<br>China<br>2009-2011<br>1 centre	Unilateral elective TKR, 98% for osteoarthritis<br>40; 39 (30; 30 after post randomisation exclusions)<br>Mean 68.8 (SD 6.4); 68.9 (7.5)<br>73%; 73%	Paracetamol, sustained release diclofenate, opioids (codeine or morphine). Spinal anaesthesia<br>Catheter inserted under nerve stimulation and ultrasound guidance. Standardised bolus of 15 mL 0.5% levobupivacaine. Continuous infusion of 8 to 12 mL/h 0.08% levobupivacaine postoperatively until POD 3	Intravenous PCA morphine after the operation	6 months<br>2; 2 not pre- and peri-operative exclusions<br>Low risk of bias<br>No separate pain outcome but improvement of KSS from pre-operative was FNB 48.73 and PCA 44.7 (p=0.513)<br>Including patients not followed up. Deaths: 0; 0. Infection: 1;1. DVT: 2; 3. Shock: 3;2. Transfusion: 2;3. Also from excluded cases. Atrial fibrillation and confusion: 0; 1. PE: 0; 1. Sepsis: 1;0. ICU admission for shock: 1; 0.
FNB and SNB continuous vs epidural PCA				
Anastase et al. 2014[118]<br>Germany<br>2010-2011<br>1 centre	Primary unilateral TKR<br>55; 50<br>Mean 68.2 (SD 9.2); 69.7 (SD 8.7)<br>65%; 69%	Premedication with 10 mg oral clorazepate. Spinal anaesthesia with light sedation: 12.5mg 0.5% bupivacaine. Supplemental postoperative analgesia available with i.v. piritramid<br>After spinal anaesthesia installed, SNB and FNB catheters inserted with ultrasound guidance. 5 ml bolus 0.2% ropivacaine. FNB with an hourly rate of 5 ml, bolus administration of 5 ml by the patient and the lock-out interval of 20 mins. SNB 5 ml/h to a maximum of 8 ml/h, 5 ml bolus administered by the patient	Epidural catheter installed at the same time as spinal catheter. 5ml 0.2% ropivacaine and PCA performed through the epidural catheter. Hourly rate 3 ml, bolus administration of 5 ml, and lock-out period of 30 minutes.	6 and 12 months<br>15; 14<br>High risk of bias due to large loss to follow up<br>Pain during previous 4 weeks: 1 no pain, 2 very little, 3 little, 4 moderate, 5 loud, 6 very loud (translation from German). No difference at 6 months p=0.37. At 12 months, FNB/SNB median 2.00 (1.00, 2.00), PCA 2.00 (2.00, 2.00) p=0.004 favouring FNB/SNB.<br>No falls associated with quadriceps weakness. 6 and 12 month adverse events not reported.

For peer review only - http://bmjopen.bmj.com/site/about/guidelines.xhtml

		and lock-out interval of 20 minutes.		
**FNB single vs LIA**				
Fan et al. 2016[36]<br>China<br>2012-2014<br>Single hospital, 2 surgeons	Primary unilateral TKR (75% osteoarthritis; 25% rheumatoid arthritis)<br>80; 80 (78; 79 in analysis)<br>Mean 68.4 (SD 8.8); 67.6 (6.3)<br>79%; 86%	General anaesthesia in all but 1 in each group. After surgery, i.v. morphine, PCA and parecoxib 40mg		1 year<br>3 protocol violations<br>Low risk of bias<br>No separate pain outcome. Mean KSS at 1 year similar between groups: 94.2 (SD 2.6); LIA 93.9 (3.1). p=0.51<br>Infection: 0; 0. DVT: 1; 1. Femoral nerve injury: 1; 0.
		FNB performed pre-operatively with 20ml ropivacaine 0.5%.<br>After cementing prostheses, 50ml of saline injected into periarticular soft tissue.	Placebo equivalent of FNB with saline<br>After cementing prostheses, 50ml of LIA mixture containing morphine (1ml: 10mg), ropivacaine (10ml: 100mg), and diprospan (1ml: 5mg betamethasone dipropionate and 2mg betamethasone sodium phosphate) injected into periarticular soft tissue.	
**FNB single and epidural vs LIA**				
Reinhardt et al. 2014[41]<br>USA<br>2010-2012<br>Single hospital, 2 surgeons	Elective unilateral TKR for osteoarthritis<br>51; 51 (49; 45 received allocated intervention)<br>Mean 67.9 (SD 10.9); 66.6 (10.1)<br>59.2%; 57.8%	Spinal anaesthetic (2.5ml 0.5% bupivacaine). Mobic 15mg daily. Oral Perocet or Vicodin as required. Subcutaneous Dilaudid for severe breakthrough pain. Intravenous Toradol.		1 year<br>0: 0 of patients who received allocated intervention<br>Low risk of bias<br>VAS pain at 1 year similar between groups (noted in text and shown graphically)<br>No wound-related complications or infections. 1 DVT and 1 DVT plus PE in epidural group. Arthrofibrosis: 2; 1
		Combined spinal-epidural (500ml hydromorphone 10µg/ml and bupivacaine HCl 0.06%).<br>Single intra-operative FNB injection (30ml 0.25% bupivacaine).<br>Continuous 48-hour epidural infusion (4ml/hr with 4ml per demand dose, locked out every 10 minutes with an hourly limit of 20ml). Epidural infusion weaned to 2ml/hr on POD1 and to 0 ml/hr at 5 p.m. on POD1. Demand dose with	Intra-articular knee catheter placed intraoperatively with continuous 0.2% ropivacaine infusion at 7 ml/hr until POD2.<br>Placebo epidural catheter, no FNB, and postoperative placebo continuous epidural infusion of saline.	

For peer review only

For peer review only - http://bmjopen.bmj.com/site/about/guidelines.xhtml

		lockout parameters continued for 48 hours.<br>Placebo intraarticular knee catheter placed intra-operatively with continuous saline 7ml/hr infusion until POD2.		
LIA with corticosteroid vs LIA with no corticosteroid				
Seah et al. 2011[48]<br>Singapore<br>2004-2005<br>1 hospital	TKR<br>50; 50<br>Mean 65.4; 67.9<br>Sex not stated	General or spinal anaesthesia. Postoperative oral naproxen and PCA (with morphine bolus of 1mg, lock-out time 5 minutes, and maximum dose 8 mg/hr) for 48 hours.		6 months and 2 years<br>No losses to follow up reported<br>Low risk of bias<br>No separate pain outcome but no statistically significant difference in OKS between groups at 2 years<br>Deep infection: 1; 1
		Intraoperative periarticular injection of 0.5ml/kg 1:200,000 epinephrine and 0.5% bupivacaine diluted with 30ml of normal saline. 40mg of corticosteroid (triamcinolone acetonide) was added to half the mixture. The solution with the corticosteroid was injected into the deep tissues. The remaining solution was injected into the skin incision before closure.	Intraoperative periarticular injection of 0.5ml/kg 1:200,000 epinephrine and 0.5% bupivacaine diluted with 30ml of normal saline. Half the mixture was injected into the deep tissues. The remaining solution was injected into the skin incision before closure.	
Yue et al. 2013[119]<br>China<br>2011-2012<br>1 hospital	Unilateral TKR for osteoarthritis<br>36; 36<br>Mean 70.2 (SD 6.4); 69.3 (5.7)<br>89%; 89%	General anaesthesia. PCA (25 mg/100ml morphine: a 1mg bolus, 6 minutes lock-out, and 5mg/hr maximum) for 72 hours after surgery. 5-10mg intramuscular morphine as rescue. Celecoxib pre- and post-operatively		6 and 12 months<br>No loss to follow up reported<br>Unclear risk of bias.<br>No separate pain outcome. No difference in mean KSS between groups at 6 or 12 months<br>No incision infection or tendon rupture complications
		Injections with local anaesthetic agent and adrenaline (0.75% ropivacaine 30ml, 1:1000 adrenaline 0.5ml, and isotonic sodium chloride solution 70ml) plus corticosteroid (1ml betamethasone).	Injections with local anaesthetic agent and adrenaline (0.75% ropivacaine 30ml, 1:1000 adrenaline 0.5ml, and isotonic sodium chloride solution 70ml) with no added corticosteroid.	

For peer review only - http://bmjopen.bmj.com/site/about/guidelines.xhtml

		Another 50ml syringes fluid without corticosteroid was infiltrated into the skin	Another 50ml syringes fluid without corticosteroid was infiltrated into the skin		
LIA including ketorolac vs epidural					
Spreng et al. 2012[120], Spreng et al. 2010[121]<br>Norway<br>2007–2009<br>1 hospital	Unilateral, non-cemented TKR with no patella resurfacing<br>34; 34; 34<br>66.5 (SD 11.); 67.2 (SD 8.9); 65.8 (SD 10.1)<br>61%;61%;67%	Premedication with oral paracetamol (1-2g). Spinal anaesthesia with 13-15mg bupivacaine 5mg/ml with 20µg fentanyl. If indicated, up to 10ml/hr 10mg/ml propofol for sedation. Acetaminophen 1g every 6 hours. i.v. PCA morphine for 48 hours after surgery (2mg bolus with 10 minutes lockout time). When PCA stopped, 10mg slow release oxycodone twice daily. 5mg oxycodone as rescue analgesia.			12 months<br>13 did not provide complete data<br>Unclear risk of bias due to limited reporting (long-term outcome only reported as conference abstract).<br>Perioperative analgesic treatment did not have any significant influence on any KOOS outcomes.<br>Infection: 0; 0; 1. No long-term adverse events reported
		i.v. injection of ketorolac 1ml (30mg/ml) and morphine 5ml (1mg/ml).<br>Infiltration with ropivacaine 150mg, epinephrine 0.5mg, ketorolac 30mg and morphine 5mg in 150ml saline.<br>After closure, catheter placed into knee joint and 10ml infiltrate injected. 22-24 hours after surgery, 20ml injection through catheter of ropivacaine 19ml (7.5mg/ml) and ketorolac 1ml (30mg/ml). i.v. injection of	i.v. injection of 6ml saline.<br>Infiltration with ropivacaine 150mg, epinephrine 0.5mg, ketorolac 30mg and morphine 5mg in 150ml saline.<br>After closure, catheter placed into knee joint and 10 ml infiltrate injected. 22-24 hours after surgery, 20ml injection through catheter of ropivacaine 19ml (7.5mg/ml) and saline 1ml. i.v. injection of saline 1ml.<br>Sham epidural catheter.	Epidural catheter inserted immediately before spinal anaesthesia. When spinal anaesthesia started to wear off, epidural infusion for 48 hrs with 6-10 ml/hr fentanyl 2µg/ml, epinephrine 1µg/ml, bupivacaine 1mg/ml.<br>No knee infiltrations.<br>Sham knee catheter with no injections	

For peer review only - http://bmjopen.bmj.com/site/about/guidelines.xhtml

		ketorolac 1ml (30mg/ml).<br>Sham epidural catheter.			
**Spinal with added high dose morphine sulphate vs spinal with added low dose morphine sulphate vs spinal with no morphine sulphate**					
Foadi et al. 2017[122]<br>Germany<br>Before 2017<br>1 centre	Unilateral TKR or THR for osteoarthritis<br>16; 16; 17<br>Mean 67.63 (SE 2.45); 67.33 (2.87); 63.71 (3.14) 56%; 44%; 65%	3ml spinal anaesthesia with 0.5% bupivacaine<br>Post-operative 1 g metamizole (orally or intravenously) every 4 hours. 5 mg morphine (intravenous or subcutaneous) as rescue medication			6 months<br>"only a few dropouts". >70% questionnaire return rate.<br>Unclear risk of bias due to limited reporting of pilot RCT.<br>No difference in WOMAC pain between groups at 6 months.<br>No adverse events noted
		0.2mg morphine sulphate added to spinal anaesthesia	0.1mg morphine sulphate added to spinal anaesthesia	No morphine sulphate added to spinal anaesthesia	

2. Myofascial trigger point dry needling

**Author**<br>**Country**<br>**Recruitment dates**<br>**Setting**	**Indication**<br>**Number randomised intervention; control**<br>**Age**<br>**% female**	**Common pain management**		**Follow up**<br>**Losses to follow up intervention; control**<br>**Risk of bias issues**<br>**Key results**
		**Group 1 (intervention)**	**Group C (control)**	
Mayoral et al. 2013[123]<br>Spain<br>2007-2008<br>Single centre	Unilateral TKR for osteoarthritis<br>20; 20<br>Mean 71.7 (SD 6.1); 72.9 (7.9)<br>72.5%	General or spinal anaesthesia		6 months<br>4; 5<br>High risk of bias due to large losses to follow up<br>WOMAC pain at 6 months: mean 3.24 (SD 3.03); 3.13 (2.72). Difference not statistically significant. No difference between groups in VAS pain (p=0.725) or proportion of patients reporting significant VAS pain at 6 months.
		After anaesthesia and surgery started, dry needling applied 20 times to all myofascial trigger points by a trained and experienced physical therapist.	If spinal anaesthesia used, dry needling simulated behind screen	

For peer review only - http://bmjopen.bmj.com/site/about/guidelines.xhtml

				No complications related to the dry needling intervention. Other adverse events not collected.

3. Tourniquet

**Author**<br>**Country**<br>**Recruitment dates**<br>**Setting**	**Indication**<br>**Number randomised intervention; control**<br>**Age**<br>**% female**	**Common blood conservation strategies**		**Follow up**<br>**Losses to follow up intervention; control**<br>**Risk of bias issues**<br>**Key results**
		**Group 1 (intervention)**	**Group C (control)**	
Ejaz et al. 2014[54]<br>Denmark<br>2011-2012<br>1 centre	Primary unilateral TKR for osteoarthritis<br>35; 35 (33; 31 received intervention)<br>Mean 68 (SD 8.0); 68 (7.8)<br>45.5%; 45.2%	Before surgery, oral tranexamic acid (1g). Tranexamic acid (0.5g) 3 hours after surgery and 6 and 12 hours postoperatively.		6 and 12 months<br>0; 0 of those who received intervention<br>Low risk of bias<br>Statistically significant difference in KOOS pain intensity at 2 months favouring TKR without a tourniquet (p < 0.001). Small difference between groups not statistically significant at 6 and 12 months.<br>Small number of adverse events did not suggest extra risk in the group with no tourniquet.
		Appropriately sized thigh tourniquet applied. Limb exsanguination by elevation for 2 minutes and cuff inflated to 250mm Hg.	Appropriately sized thigh tourniquet applied but not inflated. Served as safety device in case of uncontrollable bleeding.	
Liu et al. 2014[56]<br>Australia<br>Before 2014<br>1 surgeon	Unilateral TKR for osteoarthritis<br>10; 10<br>Mean 67.0; 70.0<br>30%; 10%	PCA. No CPM		6 and 12 months<br>0; 0<br>Low risk of bias<br>No separate pain measure. Total OKS not significantly different at 6 and 12 months<br>Blood transfusions: 3; 0.
		Tourniquet inflated to 300 mmHg before skin incision. Tourniquet deflated after wound closure and dressing.	Tourniquet placed but not inflated	
Mittal et al. 2012[57]<br>Australia<br>2008-2010	Primary unilateral TKR<br>31; 34	Autologous blood re-infused if required		1 year<br>5; 2
		Short duration. Tourniquet set at 300mm Hg inflated	Long-duration. Tourniquet set at 300mm Hg inflated before	

For peer review only - http://bmjopen.bmj.com/site/about/guidelines.xhtml

<table>
<tr><td>1 centre</td><td>Mean 67.5 (SD 8.9); 66.6 (8.4)
81%:74%</td><td colspan="2">prior to cement application and deflated when cement hardened</td><td colspan="2">skin incision and deflated when cement hardened</td><td>Low risk of bias. However, RCT terminated early due to increased need for blood transfusion in short duration tourniquet group.
No separate pain outcome. Total OKS (0-48) at 52 weeks higher in long-duration group reflecting better recovery than short duration group but not significantly (p=0.12). Mean difference approximately 5 which is greater than MCID of 4[78].
Transfusions: 10; 2. Patient reported adverse event: 26; 12</td></tr>
<tr><td rowspan="2">Abdel-Salam and Eyres 1995[124]
UK
Date not stated
1 surgeon</td><td rowspan="2">Primary unilateral TKR of which 91% osteoarthritis
40; 40
Mean 72 (range 65-80); 74 (64-82)
57.5%; 62.5%</td><td colspan="4">Tourniquet placed around thigh</td><td rowspan="2">1 and 2 years
0; 0
Unclear risk of bias due to limited reporting of methods. No pain measure or PROM
Surgeon recorded HSS score at 1 year 90 (78-97); 91 (80-97). Not significantly different at 1 or 2 years.
Blood loss similar between groups. Wound infections: 5;0. DVT: 4;0</td></tr>
<tr><td colspan="2">Limb exsanguinated for 2 minutes and tourniquet inflated to twice systolic blood pressure</td><td colspan="2">Tourniquet not inflated</td></tr>
<tr><td rowspan="2">Şükür et al.2016[125]
Turkey
2015
1 surgeon</td><td rowspan="2">Primary unilateral TKR, in women with osteoarthritis
30; 30; 30; 30
Mean 67.0 (SD 7.0); 66.9 (8.5); 68.4 (6.9); 68.4 (6.8)
100%</td><td colspan="4">Pneumatic tourniquet inflated to 125mm Hg above systolic blood pressure</td><td rowspan="2">6 months
0;0;0;0
High risk of bias. KSS outcome noted in methods but not presented in results.
KSS results not reported at 6 months but no significant differences between groups at 3 months.
Surgical and wound complications similar between groups. No infections, fractures or instability requiring revision within 6 months</td></tr>
<tr><td>Knee in 90° flexion and tourniquet deflated during wound closure</td><td>Knee in 90° flexion and tourniquet inflated during wound closure</td><td>Knee in full extension and tourniquet deflated during wound closure</td><td>Knee in full extension and tourniquet inflated during wound closure</td></tr>
<tr><td></td><td></td><td colspan="4">Blood transfusion if required</td><td>3-22 months, mean 12;13 months</td></tr>
</table>

For peer review only - http://bmjopen.bmj.com/site/about/guidelines.xhtml

Zhang et al.2016 [126]<br>China<br>2014-2015<br>1 hospital	Primary TKR for osteoarthritis<br>84; 82<br>Not reported<br>Not reported	Tourniquet		No tourniquet	Not clear<br>High risk of bias. Variable follow up. HSS outcome noted in methods but not presented in results.<br>HSS not reported.<br>Transfusion rates similar between groups. At mean follow up of 12 -13 months, patients operated on without a tourniquet had a lower rate of DVT (2.4%) compared with those with a tourniquet (10.7%).
Zhang et al. 2017[58]<br>China<br>2008-2011<br>1 surgeon	Primary unilateral cemented TKR for osteoarthritis<br>50; 50; 50<br>Mean 70.3 (SD 6.6); 71 (10.2); 68.2 (6.8)<br>54%; 60%; 50%	Tourniquet inflated to 300-337mm Hg. Tranexamic acid not generally used			6 months<br>0; 0; 0<br>Low risk of bias<br>No separate pain outcome. HSS similar between groups at 6 months (p=0.839).<br>At 2 weeks DVT: 0; 0; 1. Intramuscular vein thrombosis: 4; 3; 3. Transfusions: 30%; 26%; 10%
		Tourniquet for entire operation	Tourniquet removed before wound closure	Tourniquet from first bone osteotomy until wound closure	
Huang et al. 2017[55]<br>China<br>2015<br>1 centre	Primary unilateral TKR for osteoarthritis<br>50; 50<br>Mean 66.2 (SD 8.3); 65.1 (6.8)<br>64%; 68%	Tranexamic acid			6 months<br>0; 0<br>Low risk of bias<br>VAS pain similar between groups at 6 months (p=0.728). Mean HSS score 90.3 (SD 3.2); 91.2 (2.5). P=0.151<br>DVT: 0; 0. PE: 0; 0. Intramuscular venous thrombosis: 6; 4. Superficial infection: 1; 0. Wound secretion: 6; 0. No significant difference in blood loss between groups.
		Tourniquet		No tourniquet	

4. Compression bandage

Author	Indication	Common treatments	Follow up

For peer review only

For peer review only - http://bmjopen.bmj.com/site/about/guidelines.xhtml

Country<br>Recruitment dates<br>Setting	Number randomised intervention; control<br>Age<br>% female	Intervention	Control	Losses to follow up intervention; control<br>Risk of bias issues<br>Key results
Brock et al. 2017[127]<br>UK<br>2013-2014<br>1 hospital	Primary unilateral TKR for osteoarthritis<br>25; 25 (24 received intervention)<br>Mean 67.3 (SD 8.2); 69.5 (6.8)<br>66.7%; 64.0%	Hydrocolloid dressing left in place until clips removed on day 10-14<br>Soft inner layer with compressive outer layer bandage. Removed after 24 hours.	Standard bandaging with soft inner layer and crepe bandage outer layer. Removed after 24 hours and cryocuff used.	6 months<br>0; 0 of patients receiving intervention<br>Low risk of bias<br>No separate pain outcome. Mean OKS similar between groups at 6 months: 35.8 (SD 7.7); 34.3 (10.6). P=0.58<br>No infections or thromboembolic events in either group

5. Blood conservation

Author<br>Country<br>Recruitment dates<br>Setting	Indication<br>Number randomised intervention; control<br>Age<br>% female	Common blood conservation strategies		Follow up<br>Losses to follow up intervention; control<br>Risk of bias issues<br>Key results
		Intervention	Control	
Tranexamic acid				
Sa-Ngasoongsong et al. 2011[64]<br>Thailand<br>2008-2009<br>1 hospital	Primary knee osteoarthritis with unilateral primary cemented computer assisted TKR<br>24; 24<br>69.0 (SD 8.2); 69.2 (7.6)<br>91.7%; 75%	Drain and compressive dressing<br>25ml saline solution containing 250mg tranexamic acid injected into knee joint after fascial closure	25ml saline solution injected into knee joint after fascial closure	6 months<br>0; 0<br>Low risk of bias<br>No separate pain score reported but WOMAC overall score mean 18.6 (SD 7.6); 20.8 (6.4). P=0.282<br>Lower peri-operative blood loss in tranexamic acid group and need for blood transfusion, 1/24 compared with 8/24 in control group. No DVT,

For peer review only - http://bmjopen.bmj.com/site/about/guidelines.xhtml

					wound complications or infection reported in either group
Kim et al. 2014[61]<br>Korea<br>2009-2011<br>1 hospital	Primary unilateral TKR for osteoarthritis<br>90; 90<br>Mean 73.5 (SD 5.5); 71.9 (SD 5.9)<br>88%; 87%	Tourniquet, drain, compressive dressing. Allogenic blood transfusion and intravenous iron and erythropoietin if required			1 year<br>0; 0<br>Low risk of bias<br>WOMAC pain mean 3.2 (2.6); 2.8 (2.3). Difference not statistically significant<br>Lower blood loss and need for allogenic transfusion in tranexamic acid group. No DVT. 1 PE in control group.
		10 mg/kg body weight tranexamic acid in 100 mL of normal saline given as slow intravenous injection 30 min before tourniquet deflation, and the same amount 3 hours later.	No tranexamic acid and no placebo		
Sa-Ngasoongsong et al. 2013[65]<br>Thailand<br>2010-2011<br>1 hospital	Primary unilateral cemented TKR for osteoarthritis<br>45; 45; 45<br>Mean 68.1 (SD 6.2); 67.6 (8.7); 66.2 (7.3)<br>88.9%; 93.3%; 95.6%	Drain and compressive dressing			1 year<br>0; 0; 0<br>Low risk of bias<br>No separate pain outcome but WOMAC mean 14.5 (7.1); 15.1 (6.2); 15.5 (6.6). P=0.42<br>Total blood and Hb loss lower in intervention groups than control. Fewer transfusions in 500mg (0) than 250mg tranexamic acid group (6) and control group (10). 2 DVT in 500mg group. 1 DVT in 250mg group. 1 PE and 3 DVT in control group. No infections.
		25ml saline solution containing 500mg tranexamic acid injected into knee joint after fascial closure via drain tube.	25ml saline solution containing 250mg tranexamic acid injected into knee joint after fascial closure via drain tube.	25ml saline solution injected into knee joint after fascial closure	
Hourlier et al. 2015[60]<br>France<br>2009-2010<br>1 hospital	Primary unilateral TKR for osteoarthritis<br>52; 54<br>74 (SD 6); 72 (7)<br>62%; 63%	Tourniquet, electrocautery, routine haemostasis, superficial drain. No blood salvage system.			6 months<br>0; 0<br>Low risk of bias.<br>No separate pain score but KSS clinical score mean 90 (SD 6); 90 (13). P=0.90<br>No difference between groups in total blood loss. 1 MUA in single treatment
		10 mg/kg intra-operative tranexamic infusion. After 2 hours, continuous infusion of tranexamic acid 2 mg/kg/hr for 20 hours via electric syringe	single bolus of 30 mg/kg tranexamic acid as an intraoperative infusion. After 2 hours, placebo saline continuous infusion via electric syringe		

For peer review only - http://bmjopen.bmj.com/site/about/guidelines.xhtml

				group. No deep infections or revisions.
Huang et al. 2017[55]<br>China<br>2015<br>1 centre	Primary unilateral TKR for osteoarthritis<br>50; 50<br>Mean 66.2 (SD 8.3); 65.8 (6.3)<br>64%; 70%	Tourniquet inflated to 100mm Hg above SBP before incision and deflated after wound closure		6 months<br>0; 0<br>Low risk of bias<br>VAS pain similar between groups at 6 months (p=0.728). Mean HSS score (0-100) better in tranexamic acid group than controls: 90.3 (SD 3.2); 88.9 (3.0). P<0.001. Mean difference 1.4 lower than MCID of 8.29[81]<br>Greater blood loss in control group than tranexamic group (p<0.001). DVT: 0; 0. PE: 0; 0. Intramuscular venous thrombosis: 6; 3. Superficial infection: 1; 3. Wound secretion: 6; 9.
		Intravenous tranexamic acid 20mg/kg before incision and tranexamic acid 10mg/kg at 3, 6, 12 and 24 hours. 1g tranexamic acid in 50ml saline irrigated into wound during operation	No treatment with tranexamic acid	
Thrombin infusion				
Kusuma et al. 2013[62]<br>USA<br>Not stated<br>1 hospital	Primary unilateral TKR for osteoarthritis<br>40; 40<br>Mean 64.6 (SD 10.2); 64.5 (7.3)<br>82.5%; 67.5%	Tourniquet, drain, Esmarch bandage, electrocautery		1 year (6 months and 2 years also reported)<br>0; 0<br>Low risk of bias<br>No separate pain outcome. KSS mean 95.5; 96.0. p=0.45<br>Lower drop in Hb in thrombin group. Blood transfusion in 4 intervention and 7 control patients. 1 control patient had haematoma. No hospital readmissions
		20,000 IU thrombin infusion (1,000 IU/mL) through fascial defect	Closure and drain placement protocol without the thrombin infusion.	
Flexion vs extension				
Napier et al. 2014[63]<br>UK<br>2003-2004<br>1 hospital	Primary unilateral TKR of which 89% for osteoarthritis<br>90; 90	No drains or tranexamic acid		1 year<br>5; 1 (12 did not attend follow up)<br>Low risk of bias.<br>No separate pain outcome. OKS mean 20.5 (SD9.0); 22.1 (9.7). P=0.27
		Flexion. Operated knee kept in passive flexion (120°) post-operatively for 6 hours using a jig. Wound redressed and placed in flexion over a	Extension. Operated knee kept in full passive extension	

For peer review only - http://bmjopen.bmj.com/site/about/guidelines.xhtml

	Mean 70.4 (SD 9.9) 71.0 (7.6)<br>74%; 64%	single pillow until POD1 morning.		1 MI and 1 DVT in each group. 1 haematoma in flexion group. 1 deep infection and 1 extensor muscle weakness in extension group. More transfusions in extension group (p=0.002)
Auto-transfusion of washed blood				
Thomas et al. 2001[128]<br>UK<br>Not stated<br>1 hospital	Unilateral TKR<br>115; 116<br>Mean 69.3 (range 32-95); 70.0 (40-88)<br>62%; 53%	Allogenic transfusion if Hb fell below 9g/dl		6 months<br>Losses to follow up not reported<br>Unclear risk of bias due to limited details of methods and follow up.<br>No separate pain outcome. No significant difference in EQ-5D between groups.<br>7% of auto-transfusion group required allogenic transfusion compared with 28% in control group. Fewer infections, readmissions and GP visits in auto-transfusion group. No significant differences in other serious adverse events or mortality between groups.
		Auto-transfusion of wound drainage if volume >125ml post-operative. Blood washed and re-suspended before re-infusion using a centrifugal cell washing machine	Wound drainage discarded	

6. Platelet rich plasma

**Author**<br>**Country**<br>**Recruitment dates**<br>**Setting**	**Indication**<br>**Number randomised intervention; control**<br>**Age**<br>**% female**	**Common blood conservation strategies**		**Follow up**<br>**Losses to follow up intervention; control**<br>**Risk of bias issues**<br>**Key results**
		**Group 1 (intervention)**	**Group C (control)**	
Aggarwal et al. 2014[129]	Primary unilateral surgery or first surgery of staged	Tourniquet. No tranexamic acid or suction drain. Blood transfusion if necessary due to intraoperative blood loss or postoperative haemoglobin <8g/dl.		6 months<br>No losses to follow up reported

For peer review only - http://bmjopen.bmj.com/site/about/guidelines.xhtml

India<br>2010-2011<br>1 surgeon	bilateral TKR for osteoarthritis<br>7; 14<br>Mean 56.43 (SD 7.59); 53.79 (9.75)<br>Sex not stated	8 ml PRP, prepared from patient's blood. Calcium chloride for activation given in a separate syringe in 4:1 ratio. PRP and calcium chloride injected into the posterior recess, gutters and capsule, and repaired extensor mechanism and prepatellar fat.	No treatment	High risk of bias due to unexplained differences in numbers of patients in randomised groups.<br>No separate pain outcome. WOMAC total at 6 months PRP mean 7.14 (SE 0.69), controls 7.86 (1.23), p=0.173<br>PRP group had lower fall in haemoglobin and need for blood transfusion

7. Cryotherapy

**Author**<br>**Country**<br>**Recruitment dates**<br>**Setting**	**Indication**<br>**Number randomised intervention; control**<br>**Age**<br>**% female**	**Common treatment**		**Follow up**<br>**Losses to follow up intervention; control**<br>**Risk of bias issues**<br>**Key results**
		**Group 1 (intervention)**	**Group 1 (intervention)**	
Wang 2017[130]<br>China<br>2013-2015	Unilateral TKR for osteoarthritis<br>53; 53<br>Mean 65.23 (SD 5.41); 64.97(5.36)<br>62.3%; 58.5%	CPM for 2 weeks		6 months<br>0; 0<br>Unclear risk of bias due to limited reporting<br>No separate pain outcome. At 6 months 87% of cryotherapy patients had excellent or good knee function compared with 69% of controls (p=0.032).<br>No adverse events reported in either group during functional training
		Compression cold therapy for 48 hours	No compression cold therapy	

For peer review only

8. Denusomab

Author<br>Country<br>Recruitment dates<br>Setting	Indication<br>Number randomised intervention; control<br>Age<br>% female	Common treatment		Follow up<br>Losses to follow up intervention; control<br>Risk of bias issues<br>Key results
		Group 1 (intervention)	Group 1 (intervention)	
Ledin et al. 2017[66]<br>Sweden<br>2012-2014<br>2 centres	Elective cemented primary unilateral TKR for osteoarthritis<br>25; 25<br>Mean 66 (SD 6.3); 64 (5.5)<br>60%; 60%			12, 24 months<br>0; 2<br>Low risk of bias<br>No significant differences in KOOS pain or other KOOS domains between groups 12 12 or 24 months<br>No suspected unexpected adverse reactions in either group
		Injection of 60mg denusomab 1 day after surgery and after 6 months	Injection of placebo 1 day after surgery and after 6 months	

9. Continuous passive motion

Author<br>Country<br>Recruitment dates<br>Setting	Indication<br>Number randomised intervention; control<br>Age<br>% female	Common treatment			Follow up<br>Losses to follow up intervention; control<br>Risk of bias issues<br>Key results
		Group 1 (intervention)	Group 2 (intervention)	Group C (control)	
Leach et al. 2006[131]<br>UK<br>Before 2005<br>1 hospital	Unilateral cruciate retaining rotating platform TKR for osteoarthritis<br>85 overall<br>Mean 71.2 (range 53-84); 72.9 (52-89)	Physiotherapy protocol from POD1 including slider board exercises to improve ROM and quadriceps strengthening exercises.			6 and 12 months<br>25 patients lost to follow up<br>High risk of bias due to large loss to follow up and use of date of birth randomisation<br>No difference in mean VAS pain at 1 year, CPM 0.6; control 0.9. p=0.49
		CPM commenced on first postoperative day set at a range 0–30 and used for 1 hour twice per day. Each day,		No CPM	

For peer review only - http://bmjopen.bmj.com/site/about/guidelines.xhtml

<table>
<tr><td></td><td>50%; 54%</td><td colspan="3">range was increased by 10° with discharge at POD 5-7.</td><td colspan="2"></td><td>Adverse events not reported</td></tr>
<tr><td rowspan="2">Sahin et al. 2006[132]
Turkey
Before 2006
1 hospital</td><td rowspan="2">Primary unilateral TKR for osteoarthritis
15; 16
Mean 61 (SD 6.0); 61.6 (7.5)
86%; 86%</td><td colspan="5">Standard physiotherapy</td><td rowspan="2">6 months
3 lost to follow up
Unclear risk of bias as patients were followed up by treating physician.
Mean difference in VAS pain 0.1/10 slightly favouring no CPM group (95% CI -0.8, 0.9; P=0.87)
Adverse events not known</td></tr>
<tr><td colspan="3">From POD 1, CPM 2.5 hours 2x/day. Initially 0-40° flexion and increased by 10° each day until POD 7</td><td colspan="2">No CPM</td></tr>
<tr><td rowspan="2">Pope et al. 1997[133]
Australia
1988-1999
1 hospital</td><td rowspan="2">Primary unilateral or bilateral TKR of which 86% for osteoarthritis
62 (70 knees). Authors excluded those not followed up so groups were 18; 20; 19
Mean 72.5 (95% CI 64.4, 74.98); 72.7 (70.4, 75.0); 69.4 (64.4, 74.98)
64.7%; 50%; 72.2%</td><td colspan="5">Physiotherapy commenced on postoperative day 1</td><td rowspan="2">6 and 12 months
8 patients (12 knees) excluding 1 death
High risk of bias due to losses to follow up and limited reporting of methods
No separate pain outcome. However, "pain disability" contributed up to 50 points out of a total of 70-point functional score (70 best outcome). No difference between groups in functional score: CPM 0-40 median 56 (range 20, 70); CPM 0-70 52 (10, 70); no CPM 52 (25, 70). p=0.80
CPM groups had greater blood loss than controls, p=0.008). 1 manipulation under anaesthesia in no CPM group, 2 revisions due to patellar dislocation in the 0-40 CPM group, 1 PE death in the 0-70 CPM group.</td></tr>
<tr><td>Patients had an initial CPM range of 0-40° increased by 10° twice, on day after surgery and day 2, so that 0-60° flexion achieved before removal of machine at 48 hours</td><td colspan="2">Patients had an initial CPM range of 0-70° increased by 10° twice, on day after surgery and day 2, so that 0-90° flexion achieved before removal of machine at 48 hours</td><td colspan="2">Knee placed in an extension splint in the recovery room</td></tr>
<tr><td>Beaupré etal. 2001[134]</td><td>Primary unilateral TKR of which</td><td colspan="5">Standardised exercise during hospital admission which included a slider board session.</td><td>6 months</td></tr>
</table>

For peer review only

For peer review only - http://bmjopen.bmj.com/site/about/guidelines.xhtml

<table>
<tr><td>Canada
1997-1998
1 hospital</td><td>92% for osteoarthritis
40; 40; 40
Mean 68 (SD 9); 68 (9); 69 (8)
52.5%; 50%; 30%</td><td>3 sessions (2 hours) with CPM machine per day from POD2. Range increased from starting range 0-30 degrees as tolerated.</td><td>Minimum of two 10-minute slider board therapy sessions per day in addition to one in the standardised exercise. Active knee flexion and extension in sitting and lying positions performed independently as tolerated.</td><td>No intervention further than standardised exercise.</td><td>6; 8; 6
Unclear risk of bias due to losses to follow up
Mean WOMAC pain at 6 months: 76 (15); 85 (15); 79 (16). No difference over time between groups, p=0.62.
Long-term adverse events. Need for MUA: 1; 1; 0. DVT: 0; 1; 0. Cellulitis: 0; 0; 1. Infection 0; 0; 1.</td></tr>
<tr><td rowspan="2">Kumar et al. 1996[135]
USA
Before 1996
1 hospital</td><td rowspan="2">Primary unilateral TKR for osteoarthritis
40 (46 knees); 33 (37)
Mean 69 (range 52-86); 68 (42-88)
58%; 67%</td><td colspan="3">Standard physiotherapy</td><td rowspan="2">6 months
15; 13 lost to follow up
High risk of bias due to large losses to follow up
No separate pain outcome. KSS CPM 82.7; Drop and dangle 80.7. p=0.78
Haematoma 3;1. Closed manipulation 1;3. DVT 0;0. PE 0;1</td></tr>
<tr><td colspan="2">CPM from POD 0. Initially 10 hours/ day 0-90° until discharge</td><td>No CPM. Passive range of movement (“drop and dangle”) to 90° 2x/ day initially for 20 minutes, later 30-45 minutes.</td></tr>
<tr><td rowspan="2">Worland et al. 1998[136]
USA
1996
1 hospital</td><td rowspan="2">Unilateral or bilateral TKR for osteoarthritis.
91 patients (114 knees randomised). After post-randomisation exclusions: 37 (49 knees); 43 (54 knees)
Mean 70.2 (range 44-84)
66.25%</td><td colspan="3">CPM and physiotherapy during hospital admission</td><td rowspan="2">6 months
11 patients (11 knees)
Unclear risk of bias due to post-operative exclusions not reported separately for groups and limited reporting of methods.
No separate pain outcome. At 6 months, mean HSS score CPM 95.3 (SD 2.8); physiotherapy 95.7 (3.0). P=0.49.
Adverse events not reported.</td></tr>
<tr><td colspan="2">At home after discharge, CPM machine 3 hours per day on replaced knee for l0 days.</td><td>Physical therapist home visit 1 hour three times per week for 2 weeks</td></tr>
</table>

For peer review only

MacDonald et al. 2000[137]<br>Canada<br>Before 2000<br>1 hospital	Primary unilateral TKR for osteoarthritis<br>40; 40; 40<br>Age and sex not reported	Active ROM, passive ROM exercises, mobilised as tolerated using walker or crutches.			6 and 12 months<br>Not reported<br>Unclear risk of bias due to limited and selective reporting.<br>No separate pain outcome. No statistical differences between groups for KSS at 6 and 12 months.<br>Adverse events not reported
		CPM commenced POD 0. Initially 0-50 degrees. Provided for 18-24 hour/ day. Increased by 10 degrees/ hour as tolerated. Continued until POD 1	CPM commenced POD 0. Initially 70-110 degrees. Provided for 18-24 hr/ day. Not increased.<br>Continued until POD 1	No CPM	
Bennett et al. 2005[67]<br>Australia<br>1997-2000<br>1 hospital	Primary unilateral TKR for osteoarthritis<br>47; 48; 52<br>70.7; 71.4; 71.7<br>72.3%; 64.6%; 67.3%	Standard in hospital physiotherapy programme			12 months<br>1 patient excluded due to inability to achieve 90° flexion<br>Low risk of bias<br>No separate pain outcome. No significant difference in KSS between groups at 1 year.<br>No difference in wound healing between groups
		Standard CPM from 0° to 40° for 2x3 hours on POD 1 increased by 10° per day until POD 6. Extension splint applied overnight	Early flexion CPM commenced in recovery room from 90° to 50° knee flexion. Increased gradually to CPM 90° to 0° for 2x3 hours in day 4-6.	No CPM	
Ersözlü et al. 2009[68]<br>Turkey<br>2003-2004	Primary unilateral TKR for osteoarthritis<br>30; 30; 30<br>Mean 65 (range 54-73); 61 (49-80); 62 (52-78)<br>66%; 55%; 57%	Conventional physical therapy			2 years<br>1; 1; 2<br>Low risk of bias<br>No separate pain outcome. KSS scores 98; 95; 92. No significant difference between groups p=0.67.<br>Infection 0; 0; 1. Arrhythmia 0; 1; 0. No difference in complications between groups
		CPM set at 30-40° from POD1. Increased as tolerated to POD7. 1 hour CPM 3x/day.	CPM set at 60-70° from POD3. Increased by 10°/ day to POD7. 1 hour CPM 3x/day.	No CPM	

For peer review only

10. Electrical stimulation

**Author**	**Indication**	**Common rehabilitation strategies**	

For peer review only - http://bmjopen.bmj.com/site/about/guidelines.xhtml

Country<br>Recruitment dates<br>Setting	Number randomised intervention; control<br>Age<br>% female	Intervention	Intervention	Common rehabilitation strategies
Avramidis et al. 2011[69]<br>Greece<br>2005-2006<br>1 hospital	Elective primary unilateral TKR for osteoarthritis<br>38; 38<br>Mean 70.54 (SD 4.68); 70.66 (3.73)<br>80%; 82.9%	Standard physiotherapy for 6 weeks. No CPM		1 year<br>3 (intervention intolerance); 3<br>Low risk of bias<br>Improved SF-36 bodily pain at 1 year in intervention group compared with control, mean 92 (SD 10.57); 79.48 (12.72). P<0.001. Difference of 12.52 close to MCID of 16.86[82]. No difference in OKS or American KSS<br>Adverse events not reported
		Transcutaneous electric muscle stimulation of the vastus medialis muscle from POD2 2x/ day for 2 hours for 6 weeks.	No intervention	
Stevens-Lapsley et al. 2012[138]<br>USA<br>2006-2010<br>1 hospital	Primary unilateral TKR for osteoarthritis<br>35; 31<br>Mean 66.2 (SD 9.1); 64.8 (7.7)<br>57.1%; 51.6%	Standard inpatient rehabilitation, home and outpatient physical therapy		6 months and 1 year<br>5; 6<br>Unclear risk of bias due to baseline differences in WOMAC<br>No difference in resting pain (points) at 1 year intervention mean 0.6 (SD 1.4); control 0.4 (1.5). Also similar at 6 months. Mean WOMAC total score better at 1 year in intervention group compared with control, 5.7 (5.9); 10.0 (12.2) and at 6 months. However, probably explained by baseline differences. Authors state no differences for change in WOMAC.<br>DVT 1; 0. Unspecified complication 1; 0. Infection 0; 2. Revision 0; 1
		Neuromuscular electrical stimulation commenced on POD2 for 6 weeks 2x/ day.	No intervention	

For peer review only - http://bmjopen.bmj.com/site/about/guidelines.xhtml

Levine et al. 2013[139]<br>USA<br>Before 2013<br>1 surgeon	Elective unilateral TKR for osteoarthritis<br>35; 35<br>Mean 68.1; 65.1<br>76%; 62%	2 sessions of ROM exercise<br>Neuromuscular electrical stimulation commenced 14 days pre-operatively until 1 day before surgery. Recommenced at POD1 for 60 days. After hospital discharge no direct contact with a physical therapist	Formal physical therapy programme with progressive resistive exercises and strengthening in hospital and after discharge supervised by physical therapist.	6 months<br>5; 9<br>Unclear risk of bias due to large uneven losses to follow up<br>KSS pain favoured intervention at 6 months but not significantly 79.08 (SD 10.97); 75.5 (14.77); 95%CI for difference -3.78, 10.93. Similar for WOMAC total score, 95%CI for difference -3.19, 14.81.<br>Confusion 2; 0
Moretti et al. 2012[70]<br>Italy<br>2008-2010<br>1 hospital	Primary unilateral TKR for osteoarthritis<br>15; 15<br>Mean 70.0 (SD 10.6); 70.5 (8.1)<br>Not reported	Rehabilitation protocol including CPM<br>Pulsed electromagnetic fields (I-ONE therapy) from POD7, 4 hours/ day for 60 days	No intervention	6 and 12 months<br>No losses to follow up<br>Low risk of bias<br>Mean VAS pain (10-point scale) lower at 12 months in intervention group compared with control, 0.5 (SD 1.3); 3.6 (3.9). p< 0.05. Mean difference of 2.1 (10-point scale) greater than MCID of 16.1 (100-point scale)[83]<br>Difference also at 6 months.<br>More swelling of the knee in intervention patients than controls, statistically significant at 1 and 2 months
Adravanti et al. 2014[140]<br>Italy<br>1 hospital	Primary unilateral TKR for osteoarthritis<br>16; 17<br>Mean 66 (SD 13); 73 (5)<br>62.5%; 52.9%	Standard rehabilitation protocol: active and passive mobilisation<br>Pulsed electromagnetic fields (I-ONE therapy) by POD7 for 4 hours/ day for 60 days	No intervention	6 months<br>4; 3<br>High risk of bias: small study, proportionately high losses to follow up<br>At 6 months, mean VAS pain in intervention group lower than in controls (p<0.05). At 3 years, 1/14 intervention patients and 4/12 controls reported severe pain

For peer review only - http://bmjopen.bmj.com/site/about/guidelines.xhtml

For peer review only

				No difference between groups in swelling at 6 months.

11. Rehabilitation

**Author**<br>**Country**<br>**Recruitment dates**<br>**Setting**	**Indication**<br>**Number randomised intervention; control**<br>**Age**<br>**% female**	**Common rehabilitation strategies**		**Follow up**<br>**Losses to follow up intervention; control**<br>**Risk of bias issues**<br>**Key results**
		**Intervention**	**Control**	
Walking guidance and training				
Li et al. 2017[71]<br>China<br>2015-2016<br>1 hospital	Primary unilateral TKR for osteoarthritis<br>43; 43<br>Mean 76.33 (SD 5.28); 78.47 (5.50)<br>55.8%; 51.2%	Before TKR, general guidance on joint activities, quadriceps muscle strength, use of aids, diet guidance, correct walking methods and precautions.<br>Knee passive flexion and extension to 90° and quadriceps muscle strength training commenced on POD 1. POD 3-7, straight leg raising exercises. 2 weeks after replacement, increased joint activities and muscle strength training, centre of gravity transfer training, limb weight training, and walking training.		6 months<br>0; 0<br>Low risk of bias<br>Mean VAS pain at 6 months: 0.51 (SD 0.74); 2.83 (0.88) favouring walking intervention group, p<0.01. Difference of 2.42 (10 point scale) greater than the MCID of 16.1 (100-point scale)[83]. HSS scores at 6 months favoured intervention, p<0.01.<br>No infection, allergic reaction or immune reaction in either group. Intervention not associated with swelling, pain, prosthesis loosening, thrombosis, or delayed wound healing
		Standing, weight and balance exercises from POD 1. From POD 2, walking guidance and training.	No additional rehabilitation	
Aquatic therapy				
Liebs et al. 2012[72]<br>Germany<br>2003-2004<br>4 hospitals	Elective primary unilateral TKR for osteoarthritis<br>87;98	Continuous passive motion machines daily after removal of suction drains. Programme of daily physiotherapy: range of motion activities; exercises for improvement of muscle tension, venous return, balance, coordination and gait; and instruction in activities of daily living.		6, 12 and 24 months<br>13.8%; 19.4% excluding deaths and unexplained reasons<br>Low risk of bias

For peer review only - http://bmjopen.bmj.com/site/about/guidelines.xhtml

<table>
<tr><td rowspan="2"></td><td rowspan="2">Mean 68.5 (SD 8.6); 70.9 (7.5)
70.1%;73.5%</td><td colspan="3">Aquatic therapy for 30 minutes 3 times a week up to postoperative week 5. Pool exercises aimed at training of proprioception, coordination and strengthening with aid of float cuffs, training kickboards and bar floats.</td><td rowspan="2">WOMAC pain at 12 months: early aquatic mean 13.2 (SD 15.0); late aquatic 17.4 (22.4) p=0.22. No difference at 6 and 24 months.
5 early aquatic therapy patients and 1 late aquatic therapy patient readmitted to hospital within 3 months. 2 early aquatic patients and 1 late aquatic patient readmission directly or indirectly related to the intervention.</td></tr>
<tr><td>Aquatic therapy beginning on the 6th postoperative day with the wound covered with a waterproof adhesive dressing.</td><td colspan="2">Aquatic therapy as pool exercise after the completion of wound healing on the 14th postoperative day</td></tr>
<tr><td rowspan="2">Rahmann et al. 2009[141]
Australia
2003-2005
1 hospital with 2 surgeons</td><td rowspan="2">Unilateral primary TKR or THR for osteoarthritis (50% TKR)
18;19;17 (11 had been excluded post-randomisation due to complications in hospital
Mean 69.4 (SD 6.5); 69.0 (8.9); 70.4 (9.2)
44.4%; 63.2%; 70.6%</td><td colspan="3">Standard ward-based physiotherapy until day 3. 1 ward physiotherapy treatment per day. Surgical wounds covered with an occlusive, waterproof dressing. 40 mins/ day.</td><td rowspan="2">6 months
4;2;0 for combined THR and TKR
Unclear risk of bias as TKR patients more likely to receive ward-based control intervention. THR and TKR analysed together
No difference in overall WOMAC outcome at 6 months in THR and TKR patients combined between aquatic at fast pace and ward-based (p=0.929) and aquatic at 2 paces (p=0.872).
No adverse events reported after intervention commenced.</td></tr>
<tr><td>From day 4, 1 to 1 individual physiotherapy. Aquatic physiotherapy programme to maximize function and strength. 40 mins/ day. Fast pace metronome 80-88 bpm</td><td>From day 4, 1 to 1 individual physiotherapy. Water exercise programme with general exercises not targeted at specific functional retraining in the aquatic environment. Slow pace metronome 50-58 bpm</td><td>From day 4, 1 to 1 individual ward-based physiotherapy. 40 mins/ day</td></tr>
<tr><td colspan="6">Supported early discharge</td></tr>
<tr><td rowspan="2">Mahomed et al. 2008[142]
Canada
2000-2002
2 centres</td><td rowspan="2">Primary unilateral TKR or THR for osteoarthritis (approximately 50% TKR)
119;115
68</td><td colspan="3">Inpatient physiotherapy</td><td rowspan="2">12 months
No losses to follow up
Low risk of bias (analysis by actual treatment received showed similar results)
WOMAC pain at 12 months marginally favoured home-based</td></tr>
<tr><td>Discharged home when able to independently transfer supine to sitting and sitting to standing, walk 30 metres and climb stairs if necessary. Physiotherapist home visit within 48 hours and</td><td colspan="2">Transfer to independent rehabilitation centre for 14 day stay.</td></tr>
</table>

For peer review only - http://bmjopen.bmj.com/site/about/guidelines.xhtml

	About 67% women	subsequent management along a multidisciplinary clinical pathway (4-16 visits). Then outpatient physiotherapy or self-directed programme.		rehabilitation mean 87 (SD 16); 83 SD (20), p=0.08 but this was not statistically significant. Mean difference of 4 less than MCID of 8-9[77]. Results did not differ between TKR and THR patients.<br>Similar rates of dislocation, DVT and readmissions between groups. 2% inpatient group developed infections compared with 0 in home group
Hill et al. 2000[143]<br>UK<br>1997-1998<br>1 centre	Unilateral, primary TKR, irrespective of diagnosis or concomitant disease<br>70 randomised, with 32;28 eligible for trial at day 5	Care pathway for medical, nursing and physiotherapy care from admission until day 5<br>Outreach team domiciliary visit prior to admission with assessment of home environment. At days 5–7, patients assessed to ensure discharge safe. Outreach team visit on day of discharge with further visits as required. 1+ physiotherapist visit linked with nurses to monitor knee performance. Discharge when skin clips removed, wound healed and specialist orthopaedic assistance not required, usually day 10–12	Inpatient care until removal of skin clips and wound healing.	1 year<br>No losses to follow up reported after commencement of intervention<br>Unclear risk of bias due to limited reporting of methods.<br>No pain outcome or patient reported outcome. Control group had better mean KSS scores, but this did not reach statistical significance at 1 year or earlier.<br>1;1 serious infection, other wound infections 1;6, painful joints 9;4, other minor complications similar between groups
Flexion or extension during knee closure				
Wang et al. 2014[74]<br>China<br>2009-2010<br>1 centre	Primary unilateral TKR for osteoarthritis<br>40; 40<br>Mean 68.34 (SD 7.09), 67.87 (6.47)<br>17.5%; 22.5%	No patellar replacement or lateral retinacular release<br>Articular capsule, soft tissue and skin enclosed in 90° flexion which was maintained for 1-2 min after wound closure.	Wound closure performed in full extension	6 months<br>No losses to follow up<br>Low risk of bias<br>Mean VAS pain in flexion group 1.15 (SD 0.73); extension group 1.12 (0.68), p=0.64

For peer review only - http://bmjopen.bmj.com/site/about/guidelines.xhtml

				No wound complications, patella fracture or infection requiring surgery in either group

12. Wound management

**Author**<br>**Country**<br>**Recruitment dates**<br>**Setting**	**Indication**<br>**Number randomised intervention; control**<br>**Age**<br>**% female**	**Common wound management strategies**		**Follow up**<br>**Losses to follow up intervention; control**<br>**Risk of bias issues**<br>**Key results**
		**Intervention**	**Control**	
Kong et al. 2014[75]<br>South Korea<br>2011<br>1 surgeon	Primary cemented unilateral TKR for osteoarthritis<br>50; 50<br>Mean 69.0 (SD 7.7); 68.0 (4.8)<br>89.6%; 87.5%	Skin staples removed on day 10 and wound closure strip applied for 5 days		6 and 12 months<br>2; 2 lost to follow up<br>Low risk of bias<br>At 12 months, VAS pain in silicone gel group mean 2.50 (SD 1.16); control 2.92 (1.90). P=0.201. No difference at 6 months, p=0.886.<br>No wound dehiscence or infection associated with application of silicone gel or petroleum
		After removal of wound closure strip, patients managed operation scars with application of silicone gel for 1 month after stitches removed	After removal of wound closure strip, patients managed operation scars with application of petroleum gel for 1 month after stitches removed	

13. Anabolic steroids

**Author**<br>**Country**<br>**Recruitment dates**<br>**Setting**	**Indication**<br>**Number randomised intervention; control**<br>**Age**<br>**% female**	**Common rehabilitation strategies**		**Follow up**<br>**Losses to follow up intervention; control**<br>**Risk of bias issues**<br>**Key results**
		**Intervention**	**Control**	
		Cold compression and CPM		6, 9 and 12 months

For peer review only - http://bmjopen.bmj.com/site/about/guidelines.xhtml

Hohmann et al. 2010[76]<br>Australia<br>Before 2010<br>1 surgeon	Primary unilateral TKR for osteoarthritis<br>5; 5<br>Mean 66.2 (range 58, 72); 65.2 (59, 72)<br>20%; 40%	On day 5, intramuscular injection of 50 mg Nandrolone decanoate solution. Patients visited every 2 weeks and injections continued for 6 months.	On day 5, intramuscular injection of saline. Patients visited every 2 weeks and injections continued for 6 months.	0; 0 lost to follow up<br>Low risk of bias (but small feasibility study)<br>No separate pain outcome. KSS at 12 months in intervention group mean 91.4 (SD 3.5); control 81.2 (SD 7.1). p=0.03. Difference also at 6 months (p=0.04), marginal at 9 months (p=0.06). Difference in means at 12 months of 10.2 close to MCID of 12.3[79].<br>Intervention group had smaller decrease in bone mineral density at 6 months than controls but not significant

14. Guided imagery

**Author**<br>**Country**<br>**Recruitment dates**<br>**Setting**	**Indication**<br>**Number randomised intervention; control**<br>**Age**<br>**% female**	**Common rehabilitation strategies**		**Follow up**<br>**Losses to follow up intervention; control**<br>**Risk of bias issues**<br>**Key results**
		**Intervention**	**Control**	
Jacobson et al. 2016[144]<br>USA<br>2011-2012<br>1 surgeon	Primary unilateral TKR for osteoarthritis<br>42; 40 (41; 39 received treatment)<br>Mean 65.0 SD 8.6)<br>62.2%	Participants listened to a 19-21-minute CD each day for 2 weeks before and 3 weeks after surgery. Content covered concerns and hopes about TKR with aim to facilitate mind–body connections to promote optimal TKR outcomes.	Participants listened to a 17-21-minute CD each day for 2 weeks before and 3 weeks after surgery. Content comprised poetry, short stories and essays	6 months<br>12; 10 of patients receiving treatments<br>High risk of bias due to large losses to follow up<br>Mean WOMAC pain 2.7 (SD 3.1); 3.5 (SD 3.3). P<0.001<br>Adverse events not reported

For peer review only - http://bmjopen.bmj.com/site/about/guidelines.xhtml

CD compact disc; CPM Continuous passive motion; DN4 Douleur Neuropathique 4; FNB Femoral nerve block; HSS Hospital for Special Surgery; i.v. intravenous; KOOS Knee injury and Osteoarthritis Outcome Score; KSS Knee Society Score; LIA local infiltration analgesia; NRS Numerical rating scale; OKS Oxford Knee Score; ONB obturator nerve block; PCA Patient controlled analgesia; PNB psoas nerve block; SF-36 Short Form 36 Health Survey; S-LANSS Leeds assessment of Neuropathic Symptoms and Signs Pain Scale; SNB Sciatic nerve block; TKR Total knee replacement; VAS Visual analogue scale; VTE Venous thromboembolism; WOMAC Western Ontario and McMaster Universities Osteoarthritis Index.

ITT, ITT CC, POD, MI, PE

For peer review only

For peer review only - http://bmjopen.bmj.com/site/about/guidelines.xhtml

Supplementary material. Risk of bias assessment

Study	Random sequence generation	Allocation concealment	Blinding of participants and personnel	Blind outcome assessment	Incomplete outcome data	Selective reporting	Other bias	Summary
Pain management								
Albrecht et al. 2014[34]	Computer generated	Anaesthetist blind to allocation	Physiotherapists, surgeons, research assistants collecting data, and members of the Acute Pain Service were kept blinded to group allocation.	Physiotherapists, surgeons, research assistants collecting data, and members of the Acute Pain Service were kept blinded to group allocation.	ITT analysis low losses to follow up	No but not checked protocol	Study was terminated early with 61% of planned recruitment completed due to change in standard anaesthesia at hospital	Low
Anastase et al. 2014[118]	Method of the Ulm Institute of Statistics	Method of the Ulm Institute of Statistics	No	No	15:14 lost to follow up	No but not checked protocol	ASA comorbidities differed between groups	High
Aveline et al. 2014[52]	Computer generated	opaque sealed envelopes	Blinded syringes prepared by nurse not involved in study	Yes	Low losses to follow up	Consistent with short term follow up paper	No	Low
Bergeron et al. 2009[110]	Blocks of different sizes according to list preprepared by study epidemiologist	Not described	Anaesthetist not blind. Patients blind	Nurse observers collecting data blind to allocation	32/59 lost to follow up	No but not checked protocol	No	High
Buvanendran et al. 2010[53]	computer generated	Yes, physicians and nurses blind	Yes	Yes	ITT	Protocol not checked	No	Low

For peer review only - http://bmjopen.bmj.com/site/about/guidelines.xhtml

Choy et al. 2011[35]	Computer generated	sealed envelope	No, the catheter was removed at either day 3 or 7	Patient reported outcome. Other outcomes by blinded independent physician	Low losses to follow up	Protocol not checked but reasonable range of outcomes	No	Low
Davidson et al. 2016[116]	Computer generated	Sealed opaque envelopes	Subjects and investigators were not masked to treatment group	Subjects and investigators were not masked to treatment group. PROM	31; 29 lost to follow up	None apparent but protocol not checked	Combined data from 2 RCTs	High
Fan et al. 2016[36]	No details	sealed opaque envelopes	Patients and assessors blind to randomisation	Patients and assessors blind to randomisation	2% protocol violation	No but not checked protocol	No	Low
Foadi et al. 2017[122]	Computer generated	Not described	Not described	Patient reported outcome	>70% questionnaire return	None apparent but protocol not checked	Described as pilot study	Unclear
Gao et al. 2017[37]	Random number table	Not described	Blind to patients	Blind to observers	2; 1; 0	None apparent but protocol not checked	Groups similar at baseline	Low
Ilfeld et al. 2009[113]	Computer generated	Investigators, patients, and all clinical staff were unaware of treatment group assignments	Investigators, patients, and all clinical staff were unaware of treatment group assignments	Investigators, patients, and all clinical staff were unaware of treatment group assignments	4:1 lost to follow up	No but not checked protocol	Basal infusion halved on POD1 in 10 intervention patients compared with 3 controls	High
Ilfeld et al. 2011[114]	Computer generated tables	Solutions prepared by investigational pharmacist	Yes. Intervention and control solutions indistinguishable	Patient reported outcomes. Staff masked to treatment group	11;12 did not have 4 measures out	Protocol not checked	WOMAC and WOMAC domain scores	High

For peer review only - http://bmjopen.bmj.com/site/about/guidelines.xhtml

				assignment performed all measures and assessments	of 6 up to 12 months	but seems reasonable	somewhat lower pre-intervention in extended infusion group. Authors report change scores	
Macrinici et al. 2017[38]	Computer generated	Staff performing injections blind	Anaesthesiologist, surgeons, patients and physical therapists blind to allocation	Yes	3; 4 lost to follow up. 6; 3 complications	None apparent but protocol not checked	Groups similar at baseline	Low
McDonald et al. 2016[45]	Computerised blocked	Study coordinator independent of care and surgery	Patients blind. Surgeon aware of study	Outcome assessment blind to allocation	1; 4 unexplained	None apparent, protocol not checked	Groups similar at baseline	Low
Meunier et al. 2007[51]	Computer generated	Sealed envelope	Randomisation code broken after 1 year	Yes	ITT reported except for 12 month pain outcome	No but not checked protocol	M/F ratio differed	Low
Morin et al. 2005[115]	Allocated randomly	Sealed envelope	All patients received some form of nerve block. Anaesthesiologist not blind to intervention	Observers not blinded	Per protocol analysis	No but not checked protocol	Difference between groups in anesthetist's opinion of difficulty of catheter placement. BMI differed between groups	High
Motififard et al. 2017[46]	Computer generated	Study coordinator independent of care and surgery	Patients blind. Surgeon aware of study	Outcome assessment blind to allocation	3; 7 (1; 4 unexplained)	None apparent, protocol not checked	Groups similar at baseline	Low

For peer review only - http://bmjopen.bmj.com/site/about/guidelines.xhtml

Nader et al. 2012[39]	Computer generated	Opaque envelope	No	Patient reported outcome	1:1 lost to follow up	Protocol not checked but reasonable range of outcomes	FNB group somewhat higher BMI	Low
Niemeläinen et al. 2014[47]	No details	Opaque sealed envelopes	Only research nurse preparing infiltrate aware of randomisation. All other personnel unaware until after 1 year follow up	Only research nurse preparing infiltrate aware of randomisation. All other personnel unaware until after 1 year follow up	All patients who received intervention completed follow up	No but not checked protocol	No	Low
Peng et al. 2014[40]	Computer generated	Not possible	Not possible	Patient reported outcome	31:38 lost at 12 months but ITT and per-protocol analysis	No but not checked protocol	No	Low
Perrin and Purcell 2009[112]	No details	Sealed syringe code stored in pharmacy department	yes	Yes	4 failed to complete protocol	No but not checked protocol	Pilot investigation. High risk of bias due to recruitment difficulties leading to small trial	High
Reinhardt et al. 2014[41]	Computer generated	Maintained by pharmacy department for blinding	Patients blind to intervention	Blinded research assistant and partially physical therapist	0 reported lost to follow up of those who received interventions	No but not checked protocol	No	Low
Seah et al. 2011[48]	Randomisation tables	Sealed envelopes. Anaesthetist and surgeon blind before opening of	Blinding of patients not stated	Blind outcome assessors and PROMs	No losses to follow up reported	No but not checked protocol	No	Low

For peer review only - http://bmjopen.bmj.com/site/about/guidelines.xhtml

		sealed envelope						
Shum et al. 2009[111]	No details	No details	Anaesthetist performing the blocks was not involved in the postoperative follow-up and data collection	Patient reported	14% and 20%	No but not checked protocol	Mean patient weight lower in no FNB group. More favourable mean OKS in no FNB group. Two groups combined for 2 year outcome but not for earlier	High
Spreng et al. 2012[120], Spreng et al. 2010[121]	Hospital pharmacy	Epidural catheter or sham set-up taped along the back of the patient and connected to an infusion pump covered in an opaque bag. Also sham knee catheter	Patients blind	Blind outcome assessment	13%	Limited reporting in conference abstract	Conference abstract only so limited information additional to early follow up paper	Unclear
Wang et al. 2015[117]	No details	No details	Not stated	Not stated	2:4 lost to follow up	No but not checked protocol	No	Unclear
Wegener et al. 2013[42]	No details	Opaque envelope	Patients, surgeons and researchers not blind to intervention	Patients not blinded	2:7:5 lost to follow up	No. Protocol checked	no	Low
Widmer et al. 2012[43]	Coded envelope	Coded envelope	Except for anaesthetist and surgeon	Both the investigators and patients were blinded	None reported as incomplete	No but protocol not checked	No	Low

For peer review only - http://bmjopen.bmj.com/site/about/guidelines.xhtml

Williams et al. 2013[49]	Computer generated	Not stated	Patients and assessors blind	Patients and assessors blind	3:1 of those who received treatment	No but not checked protocol	No	Low
Wu and Wong 2014[44]	Computer	Sealed envelopes	No	No	Available cases	No but not checked protocol	No	Low
Wylde et al. 2015[50]	Trials unit	Trials unit	Surgeon and anaesthetist not blind to allocation, Patients blind	Patients and research nurses blind to allocation	ITT with imputed data	No as per protocol	No	Low
Yue et al. 2013[119]	No details	No details	Surgeons and patients were double-blinded to the injection administered	surgeons and patients were double-blinded to the injection administered	Losses to follow up not reported	Limited reporting	No	Unclear
Myofascial trigger point dry needling								
Mayoral et al. 2013[123]	Computerised	Not described	Patient and other researchers apart from physical therapist blind	Patient outcomes	4: 5 loss to follow up	None apparent but protocol not checked	No	High
Tourniquet								
Abdel-Salam and Eyres 1995[124]	Card system	Not described	No	No	No losses to follow up	None apparent but protocol not checked	No	Unclear
Ejaz et al. 2014[54]	Block randomised	Sealed envelopes	Patients unaware	PROM	No losses to follow up of those who received treatments	None apparent but protocol not checked	No	Low
Huang et al. 2017[55]	Computer generated	Sealed opaque envelopes	Surgeons not blind. Patients blind to allocation	Data collector blind to allocation	No losses to follow up reported	None apparent but protocol	Groups similar at baseline	Low

						not checked		
Liu et al. 2014[56]	Excel	Not described	Patients blind	PROM	No losses	None apparent but protocol not checked.	No	Low
Mittal et al. 2012[57]	Computer generated	Sealed opaque envelopes	Patient blind	Outcome assessors blind. PROM	5:2	None apparent but protocol not checked	Study stopped because of high risk of transfusion in short tourniquet duration group	Low
Şükür et al.2016[125]	Computer generated	Not described	Possibly patients	Outcome assessors blind	No losses to follow up	KSS outcome noted in methods but not presented in results	No	High
Zhang et al. 2017[58]	Excel	Randomisation by blinded researcher.	Patients and nurses on ward blind	Not clear	No losses reported	None apparent but protocol not checked	Groups similar at baseline	Low
Zhang et al.2016[126]	Randomly allocated	Not clear	Not clear	Not clear	Not clear	HSS outcome noted in methods but not presented in results	No	High
Compression bandage								

For peer review only - http://bmjopen.bmj.com/site/about/guidelines.xhtml

Brock et al. 2017[127]	Web-based	Not specified	Not possible	No but PROMs	4; 2 of those receiving intervention	None apparent but protocol not checked	Groups similar at baseline	Low
Blood conservation								
Hourlier et al. 2015[60]	Computer generated	Opaque envelopes	Anaethsetist, surgeon and patient blind to treatment allocation	Assessors blind	No missing data	None apparent but protocol not checked	No	Low
	Computer generated	Sealed opaque envelopes	Surgeons not blind. Patients blind to allocation	Data collector blind to allocation	No losses to follow up reported	None apparent but protocol not checked	Groups similar at baseline	Low
Kim et al. 2014[61]	Computer generated	Not stated	patients blind to allocation	Clinical investigator blind to allocation	No missing data	None apparent but protocol not checked	No	Low
Kusuma et al. 2013[62]	Computer generated	Sealed envelopes	Surgeon and patient blind	Outcome assessor blind	No missing data	None apparent but protocol not checked	No	Low
Napier et al. 2014[63]	Computer generated	Sealed envelopes	Unlikely	Not stated but PROM	low losses to follow up	None apparent but protocol not checked	No	Low
Sa-Ngasoongsong et al. 2011[64]	Computer generated	Sealed envelopes	Surgeon and patient blind	Outcome assessor blind	No missing data	None apparent but	No	Low

For peer review only - http://bmjopen.bmj.com/site/about/guidelines.xhtml

						protocol not checked		
Sa-Ngasoongsong et al. 2013[65]	Computer generated	Sealed envelopes	Surgeon and patient blind	Outcome assessor blind	No missing data	None apparent but protocol not checked	Some difference between groups in pre-operative Hb	Low
Thomas et al. 2001[128]	Not described	not stated	Not reported	Not reported but PROM	Not reported but ITT	None apparent but protocol not checked	No	Unclear
Platelet rich plasma								
Aggarwal et al. 2014[129]	Not described	Opaque envelopes	Patients blind	Patients and examiners blind	No losses to follow up reported	None apparent but protocol not checked	Odd numbers in groups from randomisation	High
Cryotherapy								
Wang 2017[130]	No details	No details	No details	No details	No losses to follow up	None apparent but protocol not checked	Groups similar at baseline	Unclear
Denusomab								
Ledin et al. 2017[66]	Randomisation list produced by the study monitor	Syringes prepared independently	Investigators and patients blind	Unblinding was done after all the data had been locked	0; 2	None apparent but protocol not checked	No	Low
Continuous passive motion								
Beaupré etal. 2001[134]	Computer generated	Sealed envelopes	No	Researcher unaware and PROMs	6:8:6. Results carried	No	4 controls; 1 SB	Unclear

For peer review only - http://bmjopen.bmj.com/site/about/guidelines.xhtml

					forward for missing data		reassigned to CPM	
Bennett et al. 2005[67]	Block	Not stated	Operating surgeon blind. Patient not	Independent assessor blind	1 not included in analyses as not able to achieve 90 degree flexion	No	No	Low
Ersözlü et al. 2009[68]	Divided into groups by random selection	Not described	No	Surgeon score	A diabetic patient from the control group was excluded because of a superficial wound infection, a patient with a cardiac problem in group II due to dysrhythmia, and two patients due to insufficient follow-up.	Not apparent	No differences baseline	Unclear
Kumar et al. 1996[135]	Random number generator	Not stated	No	Not described	Large loss to follow up	Not all data clearly reported	No	High
Leach et al. 2006[131]	Allocation by date of birth	No	No	Blinded evaluation	Large loss to follow up	No	No	High
MacDonald et al. 2000[137]	Computer generated	Allocation concealed	No	Not described	Not reported	Yes, not all outcomes reported in full	No	Unclear
Pope et al. 1997[133]	Not described	Not described	Not described	Not described	No separate reporting. 8 patients (12 knees)	None apparent but protocol	No	High

					excluding 1 death	not checked		
Sahin et al. 2006[132]	Not described	Not stated	No	Followed up by treating physician	Low loss to follow up	No	No	Unclear
Worland et al. 1998[136]	Not described	Not described	No	Researcher blind	Not reported separately	None apparent but protocol not checked	No	Unclear
Electrical stimulation								
Adravanti et al. 2014[140]	Computer generated	Not described	Research assistant not involved in patient assessment	Principal investigator and all physicians in charge of clinical controls were blinded to patient allocation	78% retained at 6 months	Not apparent but protocol not checked	No	High
Avramidis et al. 2011[69]	Computer generated	Not described	No	Independent assessors blind	3 (intolerance of intervention); 3	Not apparent	Baseline similar	Low
Levine et al. 2013[139]	Drawing papers from hat	Not described	No	Not described. WOMAC PROM	5:9 for KSS pain and WOMAC	Not apparent but protocol not checked	No	Unclear
Moretti et al. 2012[70]	Computer generated	Not described	Physicians, as well as medical assessors, were blinded to the allocation of patients in the study groups	Physicians, as well as medical assessors, were blinded to the allocation of patients in the study groups	No losses reported	Not apparent but protocol not checked	No	Low
Stevens-Lapsley et al. 2012[138]	Stratified	Concealed	No	no but standardised scripts used	5; 6	Not apparent but protocol	WOMAC, BMI unequal at baseline	Unclear

						not checked		
Rehabilitation								
Hill et al. 2000[143]	Not described	Not stated	Not possible	Not described	No losses to follow up after initial 23222	No but protocol not checked	No	Unclear
Li et al. 2017[71]	Random number table	Not stated	Not possible	Not described but PROM	No losses to follow up	No but protocol not checked	Groups similar at baseline	Low
Liebs et al. 2012[72]	Computer generated	Allocation concealed	Not possible	No but PROM	Low losses to follow up (<20% if deaths and other explained reasons not counted)	No but protocol not checked	No	Low
Mahomed et al. 2008[142]	Block randomisation	Not stated	Not possible	PROM	No loss	No but protocol not checked	ITT gave similar results to analysis according to actual discharge destination (20 inpatient group received home based)	Low
Rahmann et al. 2009[141]	Not described	Sealed numbered envelopes	Not possible	Assessor blind to intervention. Patient reported outcome	Low losses to follow up	No but protocol not checked	TKR patients more likely to receive ward-based control intervention. THR and TKR analysed together	Unclear
Wang et al. 2014[74]	Computer generated	Surgeons did not participate	Surgery was performed by the	Postoperative evaluation was	No loss to follow up	None apparent	No baseline differences	Low

For peer review only - http://bmjopen.bmj.com/site/about/guidelines.xhtml

		in pre-operative grouping	physicians who did not participate in the preoperative grouping and postoperative evaluation	conducted by the physicians who were unaware of the grouping.		but protocol not checked		
Wound management								
Kong et al. 2014[75]	Not described	Not described	Placebo used	Patient outcome	Low loss to follow up	None apparent but protocol not checked	Similar at baseline	Low
Anabolic steroids								
Hohmann et al. 2010[76]	Internet based	Not reported	Placebo trial	Double-blind design minimized systemic error and eliminated observer and experimenter's bias	0 loss to follow up	None apparent	None apparent but small study	Low
Guided imagery								
Jacobson et al. 2016[144]	Permuted blocks	Opaque CD holders	Personnel yes, participants no	Yes	12; 10 of patients receiving treatment	None apparent but protocol not checked	No	High

References to all RCTs of peri-operative interventions with long-term pain or score follow up, irrespective of risk of bias assessment (numbering consistent with main article)

34. Albrecht E, Morfey D, Chan V, et al. Single-injection or continuous femoral nerve block for total knee arthroplasty? *Clin Orthop Relat Res* 2014;472:1384-93.
35. Choy WS, Lee SK, Kim KJ, et al. Two continuous femoral nerve block strategies after TKA. *Knee Surg Sports Traumatol Arthrosc* 2011;19:1901-8.
36. Fan L, Yu X, Zan P, et al. Comparison of local infiltration analgesia with femoral nerve block for total knee arthroplasty: A prospective, randomized clinical trial. *J Arthroplasty* 2016;31:1361-5.
37. Gao WL, Li H, Liu BQ, et al. Analgesic effect of femoral and sciatic nerve block under multimodal analgesia in total knee arthroplasty. *Chinese Journal of Tissue Engineering Research* 2017;21:2966-72.
38. Macrinici GI, Murphy C, Christman L, et al. Prospective, double-blind, randomized study to evaluate single-injection adductor canal nerve block versus femoral nerve block: postoperative functional outcomes after total knee arthroplasty. *Reg Anesth Pain Med* 2017;42:10-6.
39. Nader A, Kendall MC, Wixson RL, et al. A randomized trial of epidural analgesia followed by continuous femoral analgesia compared with oral opioid analgesia on short- and long-term functional recovery after total knee replacement. *Pain Med* 2012;13:937-47.
40. Peng L, Ren L, Qin P, et al. Continuous femoral nerve block versus intravenous patient controlled analgesia for knee mobility and long-term pain in patients receiving total knee replacement: a randomized controlled trial. *Evid Based Complement Alternat Med* 2014;2014:569107.
41. Reinhardt KR, Duggal S, Umunna BP, et al. Intraarticular analgesia versus epidural plus femoral nerve block after TKA: A randomized, double-blind trial. *Clin Orthop Relat Res* 2014;472:1400-8.
42. Wegener JT, van Ooij B, van Dijk CN, et al. Long-term pain and functional disability after total knee arthroplasty with and without single-injection or continuous sciatic nerve block in addition to continuous femoral nerve block: a prospective, 1-year follow-up of a randomized controlled trial. *Reg Anesth Pain Med* 2013;38:58-63.
43. Widmer BJ, Scholes CJ, Pattullo GG, et al. Is femoral nerve block necessary during total knee arthroplasty?: a randomized controlled trial. *J Arthroplasty* 2012;27:1800-5.
44. Wu JWS, Wong YC. Elective unilateral total knee replacement using continuous femoral nerve blockade versus conventional patient-controlled analgesia: perioperative patient management based on a multidisciplinary pathway. *Hong Kong Med J* 2014;20:45-51.
45. McDonald DA, Deakin AH, Ellis BM, et al. The technique of delivery of peri-operative analgesia does not affect the rehabilitation or outcomes following total knee arthroplasty. *Bone Joint J* 2016;98-B:1189-96.
46. Motififard M, Omidian A, Badiei S. Pre-emptive injection of peri-articular-multimodal drug for post-operative pain management in total knee arthroplasty: a double-blind randomized clinical trial. *Int Orthop* 2017;41:939-47.
47. Niemeläinen M, Kalliovalkama J, Aho A, et al. Single periarticular local infiltration analgesia reduces opiate consumption until 48 hours after total knee arthroplasty. A randomized placebo-controlled trial involving 56 patients. *Acta Orthop* 2014;85:614-9.
48. Seah VWT, Chin PL, Chia SL, et al. Single-dose periarticular steroid infiltration for pain management in total knee arthroplasty: a prospective, double-blind, randomised controlled trial. *Singapore Med J* 2011;52:19-23.

For peer review only - http://bmjopen.bmj.com/site/about/guidelines.xhtml

49. Williams D, Petruccelli D, Paul J, et al. Continuous infusion of bupivacaine following total knee arthroplasty: a randomized control trial pilot study. *J Arthroplasty* 2013;28:479-84.
50. Wylde V, Lenguerrand E, Gooberman-Hill R, et al. Effect of local anaesthetic infiltration on chronic postsurgical pain after total hip and knee replacement: the APEX randomised controlled trials. *Pain* 2015;156:1161-70.
51. Meunier A, Lisander B, Good L. Effects of celecoxib on blood loss, pain, and recovery of function after total knee replacement: a randomized placebo-controlled trial. *Acta Orthop* 2007;78:661-7.
52. Aveline C, Roux AL, Hetet HL, et al. Pain and recovery after total knee arthroplasty: a 12-month follow-up after a prospective randomized study evaluating Nefopam and Ketamine for early rehabilitation. *Clin J Pain* 2014;30:749-54.
53. Buvanendran A, Kroin JS, Della Valle CJ, et al. Perioperative oral pregabalin reduces chronic pain after total knee arthroplasty: a prospective, randomized, controlled trial. *Anesth Analg* 2010;110:199-207.
54. Ejaz A, Laursen AC, Kappel A, et al. Faster recovery without the use of a tourniquet in total knee arthroplasty. *Acta Orthop* 2014;85:422-6.
55. Huang Z, Xie X, Li L, et al. Intravenous and topical tranexamic acid alone are superior to tourniquet use for primary total knee arthroplasty: A prospective, randomized controlled trial. *J Bone Joint Surg (Am)* 2017;99:2053-61.
56. Liu D, Graham D, Gillies K, et al. Effects of tourniquet use on quadriceps function and pain in total knee arthroplasty. *Knee Surg Relat Res* 2014;26:207-13.
57. Mittal R, Ko V, Adie S, et al. Tourniquet application only during cement fixation in total knee arthroplasty: a double-blind, randomized controlled trial. *ANZ J Surg* 2012;82:428-33.
58. Zhang Y, Li D, Liu P, et al. Effects of different methods of using pneumatic tourniquet in patients undergoing total knee arthroplasty: a randomized control trial. *Ir Jr Med Sci* 2017;186:953-9.
59. Brock T, Sprowson A, Muller S, et al. STICKS study - Short-sTretch Inelastic Compression bandage in Knee Swelling following total knee arthroplasty - a feasibility study. *Trials* 2017;18:6.
60. Hourlier H, Reina N, Fennema P. Single dose intravenous tranexamic acid as effective as continuous infusion in primary total knee arthroplasty: a randomised clinical trial. *Arch Orthop Trauma Surg* 2015;135:465-71.
61. Kim TK, Chang CB, Kang YG, et al. Clinical value of tranexamic acid in unilateral and simultaneous bilateral TKAs under a contemporary blood-saving protocol: a randomized controlled trial. *Knee Surg Sports Traumatol Arthrosc* 2014;22:1870-8.
62. Kusuma SK, Sheridan KC, Wasielewski RC. Use of bovine thrombin to reduce blood loss in primary total knee arthroplasty: a controlled randomized trial. *J Arthroplasty* 2013;28:1278-81.
63. Napier RJ, Bennett D, McConway J, et al. The influence of immediate knee flexion on blood loss and other parameters following total knee replacement. *Bone Joint J* 2014;96-B:201-9.
64. Sa-Ngasoongsong P, Channoom T, Kawinwonggowit V, et al. Postoperative blood loss reduction in computer-assisted surgery total knee replacement by low dose intra-articular tranexamic acid injection together with 2-hour clamp drain: a prospective triple-blinded randomized controlled trial. *Orthop Rev* 2011;3:e12.
65. Sa-Ngasoongsong P, Wongsak S, Chanplakorn P, et al. Efficacy of low-dose intra-articular tranexamic acid in total knee replacement; a prospective triple-blinded randomized controlled trial. *BMC Musculoskelet Disord* 2013;14:340.
66. Ledin H, Good L, Aspenberg P. Denosumab reduces early migration in total knee replacement. *Acta Orthop* 2017;88:255-8.

For peer review only - http://bmjopen.bmj.com/site/about/guidelines.xhtml

67. Bennett LA, Brearley SC, Hart JAL, et al. A comparison of 2 continuous passive motion protocols after total knee arthroplasty: a controlled and randomized study. *J Arthroplasty* 2005;20:225-33.
68. Ersözlü S, Sahin O, Ozgür A, et al. The effects of two different continuous passive motion protocols on knee range of motion after total knee arthroplasty: a prospective analysis. *Acta Orthop Traumato* 2009;43:412-8.
69. Avramidis K, Karachalios T, Popotonasios K, et al. Does electric stimulation of the vastus medialis muscle influence rehabilitation after total knee replacement? *Orthopedics* 2011;34:175.
70. Moretti B, Notarnicola A, Moretti L, et al. I-ONE therapy in patients undergoing total knee arthroplasty: a prospective, randomized and controlled study. *BMC Musculoskelet Disord* 2012;13:88.
71. Li L, Wang Z, Yin MH, et al. Effect of early gait training on the functional rehabilitation after total knee arthroplasty. *Chinese Journal of Tissue Engineering Research* 2017;21:4288-93.
72. Liebs T, Herzberg W, Rüther W, et al. Multicenter randomized controlled trial comparing early versus late aquatic therapy after total hip or knee arthroplasty. *Arch Phys Med Rehabil* 2012;93:192-9.
73. Mahomed NN, Davis AM, Hawker G, et al. Inpatient compared with home-based rehabilitation following primary unilateral total hip or knee replacement: a randomized controlled trial. *J Bone Joint Surg (Am)* 2008;90:1673-80.
74. Wang S, Xia J, Wei Y, et al. Effect of the knee position during wound closure after total knee arthroplasty on early knee function recovery. *J Orthop Surg Res* 2014;9:79.
75. Kong CG, Kim GH, Kim DW, et al. The effect of topical scar treatment on postoperative scar pain and pruritus after total knee arthroplasty. *Arch Orthop Trauma Surg* 2014;134:555-9.
76. Hohmann E, Tetsworth K, Hohmann S, et al. Anabolic steroids after total knee arthroplasty. A double blinded prospective pilot study. *J Orthop Surg Res* 2010;5:93.
110. Bergeron SG, Kardash KJ, Huk OL, et al. Functional outcome of femoral versus obturator nerve block after total knee arthroplasty. *Clin Orthop Relat R* 2009;467:1458-62.
111. Shum CF, Lo NN, Yeo SJ, et al. Continuous femoral nerve block in total knee arthroplasty: immediate and two-year outcomes. *J Arthroplasty* 2009;24:204-9.
112. Perrin SB, Purcell AN. Intraoperative ketamine may influence persistent pain following knee arthroplasty under combined general and spinal anaesthesia: a pilot study. *Anaesth Intensive Care* 2009;37:248-53.
113. Ilfeld BM, Meyer RS, Le LT, et al. Health-related quality of life after tricompartment knee arthroplasty with and without an extended-duration continuous femoral nerve block: a prospective, 1-year follow-up of a randomized, triple-masked, placebo-controlled study. *Anesth Analg* 2009;108:1320-5.
114. Ilfeld BM, Shuster JJ, Theriaque DW, et al. Long-term pain, stiffness, and functional disability after total knee arthroplasty with and without an extended ambulatory continuous femoral nerve block: A prospective, 1-year follow-up of a multicenter, randomized, triple-masked, placebo-controlled trial. *Reg Anesth Pain Med* 2011;36:116-20.
115. Morin AM, Kratz CD, Eberhart LH, et al. Postoperative analgesia and functional recovery after total-knee replacement: comparison of a continuous posterior lumbar plexus (psoas compartment) block, a continuous femoral nerve block, and the combination of a continuous femoral and sciatic nerve block. *Reg Anesth Pain Med* 2005;30:434-45.
116. Davidson E, Machi A, Sztain J, et al. Adductor canal versus femoral continuous peripheral nerve blocks for knee arthroplasty: A one-year follow-up pilot study of two randomized, controlled clinical trials. *Reg Anesth Pain Med* 2016;41.
117. Wang F, Zhou Y, Sun J, et al. Influences of continuous femoral nerve block on knee function and quality of life in patients following total knee arthroplasty. *Int J Clin Exp Med* 2015;8:19120-5.

For peer review only - http://bmjopen.bmj.com/site/about/guidelines.xhtml

118. Anastase DM, Winckelmann J, Geiger P. Effects of regional anaesthesia techniques on patients' satisfaction after total knee arthroplasty. *Jurnalul Roman de Anestezie Terapie Intensiva* 2014;21:35-43.
119. Yue D-b, Wang B-l, Liu K-p, et al. Efficacy of multimodal cocktail periarticular injection with or without steroid in total knee arthroplasty. *Chinese Med J* 2013;126:3851-5.
120. Spreng UJ, Andersson E, Dahl V. Long-term outcome after total knee arthroplasty local infiltration analgesia (LIA) vs. Epidural analgesia. *Br J Anaesth* 2012;108:ii419.
121. Spreng UJ, Dahl V, Hjall A, et al. High-volume local infiltration analgesia combined with intravenous or local ketorolac+morphine compared with epidural analgesia after total knee arthroplasty. *Br J Anaesth* 2010;105:675-82.
122. Foadi N, Karst M, Frese-Gaul A, et al. The improved quality of postoperative analgesia after intrathecal morphine does not result in improved recovery and quality of life in the first 6 months after orthopedic surgery: a randomized controlled pilot study. *J Pain Res* 2017;10:1059-69.
123. Mayoral O, Salvat I, Martin MT, et al. Efficacy of myofascial trigger point dry needling in the prevention of pain after total knee arthroplasty: A randomized, double-blinded, placebo-controlled trial. *Evid Based Complement Alternat Med* 2013;2013:Article ID 694941.
124. Abdel-Salam A, Eyres KS. Effects of tourniquet during total knee arthroplasty. A prospective randomised study. *J Bone Joint Surg (Br)* 1995;77:250-3.
125. Sükür E, Öztürkmen Y, Akman Y, et al. The effect of tourniquet and knee position during wound closure after total knee arthroplasty on early recovery of range of motion: a prospective, randomized study. *Arch Orthop Trauma Surg* 2016;136:1773-80.
126. Zhang Q, Dong J, Gong K, et al. Effects of tourniquet use on perioperative outcome in total knee arthroplasty. *Chinese Journal of Reparative and Reconstructive Surgery* 2016;30:421-5.
127. Brock TM, Sprowson AP, Muller S, et al. STICKS study - Short-sTretch Inelastic Compression bandage in Knee Swelling following total knee arthroplasty - a feasibility study. *Trials* 2017;18:6.
128. Thomas D, Wareham K, Cohen D, et al. Autologous blood transfusion in total knee replacement surgery. *Br J Anaesth* 2001;86:669-73.
129. Aggarwal AK, Shashikanth VS, Marwaha N. Platelet-rich plasma prevents blood loss and pain and enhances early functional outcome after total knee arthroplasty: a prospective randomised controlled study. *Int Orthop* 2014;38:387-95.
130. Wang X. Impact of local compression cryotherapy combined with continuous passive motion on the early functional recovery after total knee arthroplasty. *Chinese Journal of Tissue Engineering Research* 2017;21:998-1003.
131. Leach W, Reid J, Murphy F. Continuous passive motion following total knee replacement: a prospective randomized trial with follow-up to 1 year. *Knee Surg Sports Traumatol Arthrosc* 2006;14:922-6.
132. Sahin E, Akalin E, Bircan C, et al. The effects of continuous passive motion on outcome in total knee arthroplasty. *Journal of Rheumatology and Medical Rehabilitation* 2006;17:85-90.
133. Pope RO, Corcoran S, McCaul K, et al. Continuous passive motion after primary total knee arthroplasty. Does it offer any benefits? *J Bone Joint Surg (Br)* 1997;79:914-7.
134. Beaupre LA, Davies DM, Jones CA, et al. Exercise combined with continuous passive motion or slider board therapy compared with exercise only: a randomized controlled trial of patients following total knee arthroplasty. *Physical therapy* 2001;81:1029-37.
135. Kumar PJ, McPherson EJ, Dorr LD, et al. Rehabilitation after total knee arthroplasty: a comparison of 2 rehabilitation techniques. *Clin Orthop Relat R* 1996:93-101.
136. Worland RL, Arredondo J, Angles F, et al. Home continuous passive motion machine versus professional physical therapy following total knee replacement. *J Arthroplasty* 1998;13:784-7.

For peer review only - http://bmjopen.bmj.com/site/about/guidelines.xhtml

137. MacDonald SJ, Bourne RB, Rorabeck CH, et al. Prospective randomized clinical trial of continuous passive motion after total knee arthroplasty. *Clin Orthop Relat Res* 2000:30-5.
138. Stevens-Lapsley JE, Balter JE, Wolfe P, et al. Early neuromuscular electrical stimulation to improve quadriceps muscle strength after total knee arthroplasty: a randomized controlled trial. *Physical therapy* 2012;92:210-26.
139. Levine M, McElroy K, Stakich V, et al. Comparing conventional physical therapy rehabilitation with neuromuscular electrical stimulation after TKA. *Orthopedics* 2013;36:e319-24.
140. Adravanti P, Nicoletti S, Setti S, et al. Effect of pulsed electromagnetic field therapy in patients undergoing total knee arthroplasty: a randomised controlled trial. *Int Orthop* 2014;38:397-403.
141. Rahmann AE, Brauer SG, Nitz JC. A specific inpatient aquatic physiotherapy program improves strength after total hip or knee replacement surgery: a randomized controlled trial. *Arch Phys Med Rehabil* 2009;90:745-55.
142. Mahomed NN, Davis AM, Hawker G, et al. Inpatient compared with home-based rehabilitation following primary unilateral total hip or knee replacement: a randomized controlled trial. *J Bone Joint Surg (Am)* 2008;90:1673-80.
143. Hill SP, Flynn J, Crawford EJP. Early discharge following total knee replacement -- a trial of patient satisfaction and outcomes using an orthopaedic outreach team. *J Orthop Nurs* 2000;4:121-6.
144. Jacobson AF, Umberger WA, Palmieri PA, et al. Guided imagery for total knee replacement: A randomized, placebo-controlled pilot study. *J Altern Complement Med* 2016;22:563-75.

For peer review only

Reporting checklist for systematic review and meta-analysis.

Based on the PRISMA guidelines.

Instructions to authors

Complete this checklist by entering the page numbers from your manuscript where readers will find each of the items listed below.

Your article may not currently address all the items on the checklist. Please modify your text to include the missing information. If you are certain that an item does not apply, please write "n/a" and provide a short explanation.

Upload your completed checklist as an extra file when you submit to a journal.

In your methods section, say that you used the PRISMA reporting guidelines, and cite them as:

Moher D, Liberati A, Tetzlaff J, Altman DG, The PRISMA Group. Preferred Reporting Items for Systematic Reviews and Meta-Analyses: The PRISMA Statement

		Reporting Item	Page Number
	#1	Identify the report as a systematic review, meta-analysis, or both.	1
Structured summary	#2	Provide a structured summary including, as applicable: background; objectives; data sources; study eligibility criteria, participants, and interventions; study appraisal and synthesis methods; results; limitations; conclusions and implications of key findings; systematic review registration number	2/5
Rationale	#3	Describe the rationale for the review in the context of what is already known.	4-5
Objectives	#4	Provide an explicit statement of questions being addressed with reference to participants, interventions, comparisons, outcomes, and study design (PICOS).	5
Protocol and registration	#5	Indicate if a review protocol exists, if and where it can be accessed (e.g., Web address) and, if available, provide registration information including the registration number.	5

For peer review only

Eligibility criteria	#6	Specify study characteristics (e.g., PICOS, length of follow-up) and report characteristics (e.g., years considered, language, publication status) used as criteria for eligibility, giving rational	5
Information sources	#7	Describe all information sources in the search (e.g., databases with dates of coverage, contact with study authors to identify additional studies) and date last searched.	6
Search	#8	Present full electronic search strategy for at least one database, including any limits used, such that it could be repeated.	See note 1
Study selection	#9	State the process for selecting studies (i.e., for screening, for determining eligibility, for inclusion in the systematic review, and, if applicable, for inclusion in the meta-analysis).	5,6
Data collection process	#10	Describe the method of data extraction from reports (e.g., piloted forms, independently by two reviewers) and any processes for obtaining and confirming data from investigators.	6
Data items	#11	List and define all variables for which data were sought (e.g., PICOS, funding sources), and any assumptions and simplifications made.	5/6
Risk of bias in individual studies	#12	Describe methods used for assessing risk of bias in individual studies (including specification of whether this was done at the study or outcome level, or both), and how this information is to be used in any data synthesis.	6
Summary measures	#13	State the principal summary measures (e.g., risk ratio, difference in means).	7
Planned methods of analyis	#14	Describe the methods of handling data and combining results of studies, if done, including measures of consistency (e.g., I2) for each meta-analysis.	7
Risk of bias across studies	#15	Specify any assessment of risk of bias that may affect the cumulative evidence (e.g., publication bias, selective reporting within studies).	6
Additional analyses	#16	Describe methods of additional analyses (e.g., sensitivity or subgroup analyses, meta-regression), if done, indicating which were pre-specified.	7

For peer review only

Study selection	#17	Give numbers of studies screened, assessed for eligibility, and included in the review, with reasons for exclusions at each stage, ideally with a flow diagram.	See note 2
Study characteristics	#18	For each study, present characteristics for which data were extracted (e.g., study size, PICOS, follow-up period) and provide the citation.	See note 3
Risk of bias within studies	#19	Present data on risk of bias of each study and, if available, any outcome-level assessment (see Item 12).	See note 4
Results of individual studies	#20	For all outcomes considered (benefits and harms), present, for each study: (a) simple summary data for each intervention group and (b) effect estimates and confidence intervals, ideally with a forest plot.	See note 5
Synthesis of results	#21	Present the main results of the review. If meta-analyses are done, include for each, confidence intervals and measures of consistency.	16-23
Risk of bias across studies	#22	Present results of any assessment of risk of bias across studies (see Item 15).	See note 6
Additional analysis	#23	Give results of additional analyses, if done (e.g., sensitivity or subgroup analyses, meta-regression [see Item 16]).	16-23
Summary of Evidence	#24	Summarize the main findings, including the strength of evidence for each main outcome; consider their relevance to key groups (e.g., health care providers, users, and policy makers	16-23
Limitations	#25	Discuss limitations at study and outcome level (e.g., risk of bias), and at review level (e.g., incomplete retrieval of identified research, reporting bias).	24-25
Conclusions	#26	Provide a general interpretation of the results in the context of other evidence, and implications for future research.	23-25
Funding	#27	Describe sources of funding or other support (e.g., supply of data) for the systematic review; role of funders for the systematic review.	26

Author notes

For peer review only

1. 5, Supplemetary material

2. 7, Figure 1

3. 8-15, Table1, Supplementary material

4. 7, Supplementary material

5. 8-15, Table1, Supplementary material

6. 8-15, Supplementary material

The PRISMA checklist is distributed under the terms of the Creative Commons Attribution License CC-BY. This checklist was completed on 21. November 2018 using http://www.goodreports.org/, a tool made by the EQUATOR Network in collaboration with Penelope.ai

For peer review only

BMJ Open

Are peri-operative interventions effective in preventing chronic pain after primary total knee replacement? A systematic review

Journal:	*BMJ Open*
Manuscript ID	bmjopen-2018-028093.R2
Article Type:	Research
Date Submitted by the Author:	07-Aug-2019
Complete List of Authors:	Beswick, Andrew; University of Bristol, School of Clinical Sciences<br>Dennis, Jane; University of Bristol, Musculoskeletal Research Unit<br>Gooberman-Hill, Rachael; University of Bristol, Bristol Medical School<br>Blom, AW; University of Bristol,<br>Wylde, Vikki; University of Bristol, Musculoskeletal Research Unit
**Primary Subject Heading**:	Evidence based practice
Secondary Subject Heading:	Surgery
Keywords:	Knee < ORTHOPAEDIC & TRAUMA SURGERY, Pain management < ANAESTHETICS, REHABILITATION MEDICINE

SCHOLARONE™
Manuscripts

Are peri-operative interventions effective in preventing chronic pain after primary total knee replacement? A systematic review

Andrew D Beswick[1], Jane Dennis[1], Rachael Gooberman-Hill[1,2], Ashley W Blom[1,2], Vikki Wylde[1,2]

[1] Musculoskeletal Research Unit, Translational Health Sciences, Bristol Medical School, University of Bristol

[2] National Institute for Health Research Bristol Biomedical Research Centre, University Hospitals Bristol NHS Foundation Trust and University of Bristol

Corresponding author:

Andrew David Beswick
Musculoskeletal Research Unit
Translational Health Sciences
Bristol Medical School
University of Bristol
Learning and Research Building, Level 1
Southmead Hospital
Bristol
BS10 5NB

email: andy.beswick@bristol.ac.uk
Telephone: 0117 414 7870

For peer review only

For peer review only - http://bmjopen.bmj.com/site/about/guidelines.xhtml

ABSTRACT

Objectives

For many people with advanced osteoarthritis, total knee replacement (TKR) is an effective treatment for relieving pain and improving function. Features of peri-operative care may be associated with the adverse event of chronic pain six months or longer after surgery; effects may be direct, e.g. through nerve damage or surgical complications, or indirect through adverse events. This systematic review aims to evaluate whether non-surgical peri-operative interventions prevent long-term pain after TKR.

Methods

We conducted a systematic review of peri-operative interventions for adults with osteoarthritis receiving primary TKR evaluated in a randomised controlled trial (RCT). We searched *The Cochrane Library*, MEDLINE, Embase, PsycINFO and CINAHL to February 2018. After screening, two reviewers evaluated articles. Studies at low risk of bias according to the Cochrane tool were included.

Interventions

Peri-operative non-surgical interventions; control receiving no intervention or alternative treatment.

Primary and secondary outcome measures

Pain or score with pain component assessed at six months or longer post-operative.

Results

44 RCTs at low risk of bias assessed long-term pain. Intervention heterogeneity precluded meta-analysis and definitive statements on effectiveness. There was good-quality evidence for a small benefit for reduced long-term pain with local infiltration analgesia (3 studies), ketamine infusion (1 study), pregabalin (1 study), and electric muscle stimulation (2 studies). No concerns relating to long-term adverse events were reported. In 5 RCTs, tranexamic acid to prevent blood loss was not associated with long-term pain. Many extensively researched interventions including venous thromboembolism prevention have not been evaluated in relation to long-term pain.

Conclusions

For peer review only

To prevent chronic pain after TKR, peri-operative interventions including components of multimodal analgesia, early rehabilitation and supported discharge, electrical stimulation and anabolic steroids show small benefits meriting further research. Tranexamic acid use is not associated with chronic pain but the long-term consequences of many widely researched treatments have not been reported.

STRENGTHS AND LIMITATIONS

- For the first time, this systematic review brings together contemporary evidence on aspects of peri-operative care for people with total knee replacement and their effects on long-term pain.
- Only studies assessed to be at low risk of bias were included in the narrative synthesis.
- Intervention and outcome heterogeneity precluded meta-analysis.

KEYWORDS

Total knee replacement; Systematic review; Randomised controlled trial; Peri-operative care; Long-term pain

For peer review only - http://bmjopen.bmj.com/site/about/guidelines.xhtml

BACKGROUND

In the US about 13% of men and 19% of women will be diagnosed with knee osteoarthritis and over half will receive a total knee replacement (TKR)[1]. For people with advanced osteoarthritis unresponsive to pharmacological or conservative treatments, TKR aims to relieve pain and improve function. In the UK nearly 100,000 primary TKRs were performed in 2017[2,3] and in the USA in 2010, an estimated 4.7 million people were living with a TKR[4]. Despite good outcomes for many, some people report long-term pain and are disappointed with their surgery[5,6]. After TKR, pain levels plateau from about 6 months[7,8] after which persistent pain is considered “chronic”[9] and is reported by 10-34% of patients[10].

The mechanisms that influence the development of chronic pain after total knee replacement may be biological, mechanical and psychosocial. Biological causes include the sensitising impact of long-term pain from osteoarthritis[11,12], inflammation, infection and localised nerve injury[13]. Mechanical causes include altered gait, prosthesis loosening, and effects on ligaments[14,15]. Psychological factors including depression and catastrophizing may also influence outcomes[16-19]. Much research has focused on pre-operative predictors of outcomes and these include pain intensity, presence of widespread pain, anxiety, depression and catastrophizing.[10,20] However, attempts to target or modify pre-operative care have, as yet, shown no benefit regarding chronic pain or other long-term patient outcomes[10,21-23].

Peri-operative risk factors suggest that appropriate interventions may reduce long-term pain. For example, acute post-operative pain, which may be a direct consequence of the operation, anaesthetic protocol and subsequent analgesia, or related to particular aspects of care, is an acknowledged risk factor for chronic post-surgical pain[24].

In the peri-operative period from hospital admission to the early stages of recovery, care focuses on acute pain management, prevention of adverse events, facilitation of early mobilisation and timely discharge. However, for people with osteoarthritis the key aim of TKR is the achievement of a long-term painless and well-functioning knee with no adverse events. All aspects of peri-operative care should work together to achieve this.

Any treatment in the peri-operative period including pain management, blood conservation, deep vein thrombosis (DVT) and infection prevention, and inpatient rehabilitation could potentially affect patient recovery and chronic pain, either directly or indirectly. Direct mechanisms may be through prevention of nerve damage[25], post-thrombotic syndrome[26], reperfusion injury[27] and articular bleeding[28]. For other treatments, pathways leading to long-

For peer review only - http://bmjopen.bmj.com/site/about/guidelines.xhtml

term pain may be indirect, possibly being mediated through increased risks of adverse events[29]. Irrespective of mechanism, chronic pain is a highly prevalent adverse event after TKR and should be considered along with infection, DVT and other complications in the safety profile of interventions.

Our systematic review of randomised controlled trials (RCTs) aims to evaluate the effectiveness of treatments in the peri-operative period in preventing long-term pain after TKR. By focusing on studies with low risk of bias we aim to identify interventions with robust evidence of long-term effectiveness and identify gaps in the research base.

METHODS

The systematic review protocol was registered (PROSPERO CRD42017041382) and PRISMA reporting guidelines used[30]. A checklist is included as Supplementary material.

Patient and public involvement

As part of the STAR programme of research (NIHR RP-PG-0613-20001), this review benefited from extensive patient and public involvement. Advice was sought from patients and stakeholders at a group discussion in March 2016 with decisions made on inclusion criteria and outcomes. Our patient advisory group comprises five patients with experience of long-term pain after TKR, supported by a dedicated co-ordinator. This group will advise on dissemination of the study results to a general audience including plain language summaries.

Eligibility criteria

Studies were eligible if they satisfied PICOS criteria defined in the protocol. Participants were adults receiving unilateral primary TKR with osteoarthritis in at least 75% of patients. Pharmacological or non-pharmacological interventions commenced in the peri-operative setting with “peri-operative” reflecting the time from hospital admission to immediately post-discharge. Interventions relating to implant designs and surgical procedures were excluded. The comparator was usual care, placebo or an alternative intervention. Outcomes were, in preference, patient-reported joint-specific pain intensity measured by tools such as the Western Ontario and McMaster Universities Osteoarthritis Index (WOMAC) or Oxford Knee Score (OKS). If joint-specific measures were unavailable, pain dimensions from quality of life measures were used or pain rated on a visual analogue scale (VAS) or numerical rating scale (NRS). We also considered composite patient-reported outcome measures and surgeon scores which included a pain intensity component, such as the American Knee Society Score (KSS) and Hospital for

For peer review only - http://bmjopen.bmj.com/site/about/guidelines.xhtml

For peer review only

Special Surgery (HSS) score. Measures specifically of neuropathic pain were also used. The occurrence of adverse events was summarised. The studies included were RCTs with follow up at ≥6 months after surgery and a pain outcome or score including pain. Authors of studies were contacted regarding incomplete pain outcome data.

Database searches

We established an Endnote database of all RCTs in TKR. On 14th February 2018, a search from database inception was conducted in: *The Cochrane Library*; MEDLINE, Embase and PsycINFO on Ovid; and CINAHL on EBSCOhost. The MEDLINE search strategy is included as supplementary material. Citations of key articles were tracked in Web of Science. No language restrictions were applied, and translations made. Studies reported as abstracts or unobtainable using inter-library loans and author contact were excluded.

Screening and data extraction

We imported records into Endnote X7 (Thomson Reuters). An initial screen by one reviewer excluded clearly irrelevant articles. Subsequently, abstracts and full articles were screened independently by two reviewers and reasons for exclusion recorded.

Data were extracted onto piloted forms and an Excel spreadsheet by one reviewer, specifically: country; dates; participants (indication, age, sex); inclusion and exclusion criteria; intervention and control content; setting, timing, duration and intensity of intervention; follow up intervals; losses to follow up; pain outcome data; and serious adverse events. Data was checked against source material by a second reviewer.

Authors were contacted for missing data, and data provided for previous reviews was used[10,31].

Quality assessment

Potential sources of bias were assessed by two experienced reviewers using the Cochrane risk of bias tool[32], specifically: the randomisation process; deviations from intended interventions; missing outcome data (>20%), measurement of the outcome; and selection of the reported result. Studies with serious concerns relating to risk of bias were considered high risk and those with limited reporting unclear risk. Studies with high or unclear risk of bias were excluded from the narrative synthesis but are included in supplementary summary tables with reasons for exclusion.

Data analysis

For peer review only - http://bmjopen.bmj.com/site/about/guidelines.xhtml

Insufficient studies with similar interventions and outcomes were identified for meta-analysis, and a narrative synthesis is presented. Results reported with p-values ≤0.001 were considered “strong” evidence of effectiveness[33], p-values 0.001-0.05 “some” evidence, and p-values 0.05-0.1 “weak” evidence. When authors reported results “statistically significant” with no p-value, this was noted. Where possible, effect sizes were compared with published minimal clinically important differences (MCID). Concerns relating to adverse events were summarised.

RESULTS

Figure 1 shows review progress and reasons for exclusion. Of 1515 RCTs of interventions in the peri-operative setting, 1385 had no long-term follow up. Peri-operative interventions with follow up of ≥six months were evaluated in 130 RCTs of which 76 reported a pain outcome or score with a pain component. Detailed intervention and study characteristics and risk of bias assessments are provided as supplementary material. Studies excluded had concerns for risk of bias pertaining to at least one of: large baseline differences in group characteristics or numbers in groups (n=4); incomplete outcome data (n=15); limited or selective reporting (n=12); or un-blinded surgeon follow up (n=1).

Details of 44 studies assessed to be at low risk of bias are summarised in Table 1. In 34 studies, patients received TKR exclusively for osteoarthritis and in three, 75% or more patients. In seven studies there was no information on reason for surgery but there was no suggestion that patients had an indication other than osteoarthritis. Interventions focused on pain management (n=20), tourniquets (n=5), compression bandages (n=1), blood conservation (n=7), denusomab (n=1), continuous passive motion (n=2), electrical stimulation (n=2), rehabilitation (n=4), wound management (n=1) and anabolic steroids (n=1). Primary pain outcome measures reported were VAS or NRS pain (n=12), WOMAC pain (n=7), KOOS pain (n=3), Leeds assessment of Neuropathic Symptoms and Signs Pain Scale (S-LANSS) (n=1), SF-36 bodily pain (n=1), or composite scores including a pain measure, OKS or WOMAC (n=10), KSS or HSS (n=10). Latest outcomes were recorded at 6 months (n=12), 12 months (n=26) and 24 months (n=6). Reporting of adverse events covered the entire follow up period in 27 studies, short-term after surgery in 15 studies, but were not reported in two studies.

For peer review only - http://bmjopen.bmj.com/site/about/guidelines.xhtml

Table 1. Perioperative interventions with follow up for pain or score at 6 months or later and assessed to be at low risk of bias

Study	Treatment common to randomised groups	Intervention	Number patients	Follow up<br>Group difference
Pain management: nerve blocks				
Albrecht et al. 2014[34]<br>Canada, 2009-2011,<br>1 hospital	SNB	1. FNB continuous high<br>2. FNB continuous low<br>3. FNB single	99	1 year<br>WOMAC score: no difference (p=0.68)
Choy et al. 2011[35]<br>Korea, 2006-2007,<br>1 surgeon	PCA	1. FNB continuous long<br>2. FNB continuous short	61	1 year<br>WOMAC pain: no difference (p=0.2)
Fan et al. 2016[36]<br>China, 2012-2014,<br>2 surgeons	PCA	1. FNB single<br>2. LIA	157	1 year<br>KSS: no difference (p=0.51)
Gao et al. 2017[37]<br>China, 2014-2015,<br>1 centre	LIA	1. General anaesthesia<br>2. FNB single<br>3. FNB/ SNB single	150	6 months<br>HSS score: no significant difference (p> 0.05)
Macrinici et al. 2017[38]<br>USA, Before 2017<br>1 centre	LIA	1. ACB single<br>2. FNB single	98	6 months<br>VAS pain: no difference
Nader et al. 2012[39]<br>USA, 2007-2008,<br>1 surgeon	PCA	1. FNB continuous<br>2. Oral opioid	62	1 year<br>NRS pain stair: some evidence favouring opioid (p=0.01) but not consistent. Overall NRS pain: no difference (p=1.0)<br>VTE: concern opioid
Peng et al. 2014[40]<br>China, Before 2014,		1. FNB continuous<br>2. PCA	280	6 months and 1 year<br>NRS pain: some evidence favouring FNB at 6 months

For peer review only - http://bmjopen.bmj.com/site/about/guidelines.xhtml

1 centre				(p=0.021); no difference at 1 year (p=0.273)
Reinhardt et al. 2014[41]<br>USA, 2010-2012,<br>2 surgeons		1. FNB single/ epidural<br>2. LIA 48 hours	94	1 year<br>VAS pain: no difference
Wegener et al. 2013[42]<br>The Netherlands, 2008-2010,<br>1 centre	FNB	1. SNB single<br>2. SNB continuous<br>3. PCA	89	1 year<br>WOMAC pain: no difference (p=0.81)
Widmer et al. 2012[43]<br>Australia, before 2012,<br>2 surgeons	LIA, PCA	1. FNB single<br>2. Control no FNB	55	1 year<br>WOMAC pain: no difference (p=0.74)
Wu and Wong 2014[44]<br>China, 2009-2011,<br>1 centre		1. FNB continuous<br>2. PCA	60	6 months<br>KSS: no difference (p=0.513)
Pain management: LIA				
McDonald et al. 2016[45]<br>UK, 2010-2011<br>1 hospital		1. LIA<br>2. PCA	222	1 year<br>OKS: no difference (p=0.915)
Motififard et al. 2017[46]<br>Iran, 2014-2015<br>1 hospital		1. LIA pre-emptive injection<br>2. Control saline with epinephrine	120	6 months<br>KSS: weak evidence favouring LIA (p=0.07). Difference between groups (14.2/200) less than MCID (12.3/200).
Niemeläinen et al. 2014[47]<br>Finland, 2011-2012<br>1 hospital	PCA	1. LIA<br>2. Control saline	56	1 year<br>OKS: weak evidence from means and confidence intervals favouring LIA. Difference (2.7/48) less than MCID (4.0/48)
Seah et al. 2011[48]<br>Singapore, 2004-2005	PCA	1. LIA with corticosteroid<br>2. LIA no corticosteroid	100	6 months and 2 years<br>OKS: no difference

For peer review only
For peer review only - http://bmjopen.bmj.com/site/about/guidelines.xhtml

1 hospital				
Williams et al. 2013[49]<br><br>Canada, Before 2013<br><br>2 surgeons	LIA, PCA	1. LIA 48 hours<br><br>2. Control saline	51	6 months and 1 year<br><br>VAS pain: no difference (6 months p=0.836, 1 year p=0.767)
Wylde et al. 2015[50]<br><br>UK, 2009-2012<br><br>1 centre	FNB, PCA	1. LIA<br><br>2. Control no LIA	280	6 months and 1 year<br><br>WOMAC pain: weak evidence favouring LIA at 6 months p=0.063; 1 year p=0.107. Mean difference at 1 year (3.8/100) lower than MCID (8–9/100)
Pain management: Celecoxib				
Meunier et al. 2007[51]<br><br>Sweden, 2004-2005<br><br>1 centre	PCA	1. Celecoxib<br><br>2. Control placebo	44	1 year<br><br>KOOS/VAS pain: no difference
Pain management: Ketamine/ Nefopam				
Aveline et al. 2014[52]<br><br>France, 2005<br><br>1 centre	PCA	1. Ketamine infusion<br><br>2. Nefopam infusion<br><br>3. Control saline	75	6 months and 1 year<br><br>DN4/VAS pain: some evidence favouring ketamine (for DN4 p=0.02). Few patients had neuropathic pain at 12 months.
Pain management: Pregabalin				
Buvanendran et al. 2010[53]<br><br>USA, 2006-2007<br><br>Single centre	LIA, PCA	1. Pregabalin<br><br>2. Control placebo	240	6 months<br><br>NRS pain: some evidence favouring pregabalin at 6 months (p=0.0176)<br><br>S-LANSS pain: no neuropathic pain reported in pregabalin group compared with 5.2% of patients in control group (p=0.014)<br><br>Sedation and confusion day 0 and day 1: concern pregabalin

For peer review only - http://bmjopen.bmj.com/site/about/guidelines.xhtml

Tourniquet				
Ejaz et al. 2014[54]<br>Denmark, 2011-2012<br>1 centre	Tranexamic acid	1. Tourniquet<br>2. Tourniquet not inflated	64	6 months and 1 year<br>KOOS pain: no significant difference
Huang et al. 2017[55]<br>China, 2015<br>1 centre	Tranexamic acid	1. Tourniquet<br>2. No tourniquet	100	6 months<br>VAS pain: no difference (p=0.728)<br>Wound: concern tourniquet
Liu et al. 2014[56]<br>Australia, Before 2014<br>1 surgeon		1. Tourniquet<br>2. Tourniquet not inflated	20	6 months and 1 year<br>OKS: no significant difference<br>Transfusion: concern tourniquet
Mittal et al. 2012[57]<br>Australia, 2008-2010<br>1 centre		1. Tourniquet short duration<br>2. Tourniquet long duration	65	1 year<br>OKS: weak evidence from means and Cis on graph favouring long duration at 1 year. Mean difference (5) greater than MCID (4)<br>Transfusions/ adverse events: concern short
Zhang et al. 2017[58]<br>China, 2008-2011<br>1 surgeon		1. Tourniquet for entire operation<br>2. Tourniquet removed before wound closure<br>3. Tourniquet from first bone osteotomy until closure	150	6 months<br>HSS score: no difference (p=0.839)<br>Transfusions: concern late tourniquet start in groups 1 and 2
Compression bandage				
Brock et al. 2017[59]<br>UK, 2013-2014<br>1 hospital	Hydrocolloid dressing	1. Compression bandage<br>2. Standard crepe bandage	49	6 months<br>OKS: no difference (p=0.58)
Blood conservation				

For peer review only

For peer review only - http://bmjopen.bmj.com/site/about/guidelines.xhtml

Hourlier et al. 2015[60]<br><br>France, 2009-2010<br><br>1 hospital	Drain, tourniquet, electrocautery	1. Continuous infusion tranexamic acid<br><br>2. Control saline	106	6 months<br><br>KSS: no difference (p=0.90)
Huang et al. 2017[55]<br><br>China, 2015<br><br>1 centre	Tourniquet	1. Intravenous and topical tranexamic acid<br><br>2. No tranexamic acid	100	6 months<br><br>VAS pain: no difference (p=0.728)<br><br>HSS score: strong evidence favouring tranexamic acid (p<0.001). Mean difference (1.4/100) lower than MCID (8.3/100)<br><br>Blood loss: control concern
Kim et al. 2014[61]<br><br>Korea, 2009-2011<br><br>1 hospital	Tourniquet, drain, compressive dressing	1. Tranexamic acid<br><br>2. No tranexamic acid	180	1 year<br><br>WOMAC pain: no significant difference<br><br>Transfusion: control concern
Kusuma et al. 2013[62]<br><br>USA, Before 2013<br><br>1 hospital	Tourniquet, Esmarch bandage, electrocautery	1. Thrombin infusion<br><br>2. No thrombin infusion	80	6 months, 1 and 2 years<br><br>KSS: no difference (p=0.45)
Napier et al. 2014[63]<br><br>UK, 2003-2004<br><br>1 hospital		1. Passive flexion<br><br>2. Passive extension	180	1 year<br><br>OKS: no difference (p=0.27)<br><br>Transfusion: extension concern
Sa-Ngasoongsong et al. 2011[64]<br><br>Thailand, 2008-2009<br><br>1 hospital	Drain and compressive dressing	1. Tranexamic acid<br><br>2. Control saline	48	6 months<br><br>WOMAC score: no difference (p=0.282)<br><br>Transfusion: control concern
Sa-Ngasoongsong et al. 2013[65]<br><br>Thailand, 2010-2011<br><br>1 hospital	Drain and compressive dressing	1. Tranexamic acid 500mg<br><br>2. Tranexamic acid 250mg<br><br>3. Control saline	135	1 year<br><br>WOMAC score: no difference (p=0.42)<br><br>Transfusions: control and 250mg group concerns

For peer review only - http://bmjopen.bmj.com/site/about/guidelines.xhtml

Denusomab				
Ledin et al. 2017[66]<br><br>Sweden, 2012-2014<br><br>2 centres		1. Denusomab<br><br>2. Placebo	50	1 and 2 years<br><br>KOOS pain: no significant difference
Continuous passive motion				
Bennett et al. 2005[67]<br><br>Australia, 1997-2000<br><br>1 hospital	Physiotherapy	1. Standard CPM<br><br>2. Early flexion CPM<br><br>3. No CPM	147	1 year<br><br>KSS: no significant difference
Ersözlü et al. 2009[68]<br><br>Turkey, 2003-2004<br><br>1 hospital	Physiotherapy	1. CPM low and increasing<br><br>2. CPM high and increasing<br><br>3. No CPM	90	2 years<br><br>KSS: no difference (p=0.67)
Electrical stimulation				
Avramidis et al. 2011[69]<br><br>Greece, 2005-2006<br><br>1 hospital	Physiotherapy	1. Transcutaneous electric muscle stimulation<br><br>2. No treatment	76	1 year<br><br>SF-36 bodily pain: strong evidence favouring electrical stimulation (p<0.001). Mean difference (12.5/100) close to MCID (16.9/100).<br><br>OKS/ KSS: no difference
Moretti et al. 2012[70]<br><br>Italy, 2008-2010<br><br>1 hospital	Rehabilitation protocol	1. Pulsed electromagnetic fields<br><br>2. No treatment	30	6 months and 1 year<br><br>VAS pain: some evidence favouring electrical stimulation (p<0.05). Mean difference (2.1/10) greater than MCID (16.1/100)<br><br>Knee swelling: electrical stimulation concern
Rehabilitation				
Li et al. 2017[71]<br><br>China, 2015-2016	Standard rehabilitation	1. Walking guidance and training	86	6 months

For peer review only - http://bmjopen.bmj.com/site/about/guidelines.xhtml

1 hospital		2. No treatment		VAS pain/ HSS score: some evidence favouring walking (both p<0.01). Mean VAS pain difference (2.4/100) greater than MCID (16.1/100)
Liebs et al. 2012[72]<br><br>Germany, 2003-2004<br><br>4 hospitals	CPM, physiotherapy, post-discharge aquatic therapy	1. Early aquatic therapy<br><br>2. Delayed aquatic therapy	185	6 months, 1 and 2 years<br><br>WOMAC pain: no difference (p=0.22 at 12 months)
Mahomed et al. 2008[73]<br><br>Canada, 2000-2002<br><br>2 centres	Physiotherapy	1. Multidisciplinary supported early discharge and home physiotherapy<br><br>2. Transfer to rehabilitation centre	234 hip or knee replace ment	1 year<br><br>WOMAC pain: weak evidence favouring supported discharge (p=0.08). Mean difference (4) less than MCID (8-9)
Wang et al. 2014[74]<br><br>China, 2009-2010<br><br>1 centre		1. Wound closure in flexion<br><br>2. Wound closure in extension	80	6 months<br><br>VAS pain: no difference (p=0.64)
Wound management				
Kong et al. 2014[75]<br><br>South Korea, 2011<br><br>1 surgeon	Skin staples and closure strip	1. Silicone gel<br><br>2. Petroleum gel	100	6 months and 1 year<br><br>VAS pain: no difference (6 months p=0.886, 1 year p=0.201)
Anabolic steroids				
Hohmann et al. 2010[76]<br><br>Australia, Before 2010<br><br>1 surgeon	CPM. Cold compression,	1. Intramuscular nandrolone injections<br><br>2. Saline injections	10	6 and 9 months, 1 year<br><br>KSS: some evidence favouring nandrolone (6 months p=0.04, 9 months p=0.06, 12 months p=0.03). Difference at 12 months (10.2) close to MCID (12.3)<br><br>Bone mineral density: weak evidence favouring nandrolone

ACB adductor canal block; CPM Continuous passive motion; DN4 Douleur Neuropathique 4; FNB Femoral nerve block; HSS Hospital for Special Surgery; KOOS Knee injury and Osteoarthritis Outcome Score; KSS Knee Society Score; LIA local infiltration analgesia; MCID minimal clinically important

For peer review only - http://bmjopen.bmj.com/site/about/guidelines.xhtml

difference; NRS Numerical rating scale; OKS Oxford Knee Score; PCA Patient controlled analgesia; SF-36 Short Form 36 Health Survey; S-LANSS Leeds assessment of Neuropathic Symptoms and Signs Pain Scale; SNB Sciatic nerve block; VAS Visual analogue scale; VTE Venous thromboembolism; WOMAC Western Ontario and McMaster Universities Osteoarthritis Index.

For peer review only

Pain management

We identified 20 RCTs with 2393 participants evaluating components of multi-modal pain management. Four studies each were from China and the USA, two each from Canada and the UK and one each from Australia, Finland, France, Iran, Singapore, South Korea, Sweden and The Netherlands. All were conducted at a single centre and, in those with dates, participants were recruited between 2004 and 2015. Sample sizes ranged from 44 to 280 participants, with a median of 96. Four studies had three trial arms and 16 had two. The range of mean or median ages of participants in randomised groups was 61 to 73 years and, in 17/19 studies with data, a majority of participants were women.

Femoral nerve block

Femoral nerve blocks (FNB) were studied in 10 RCTs.

Three RCTs compared FNB with no FNB. In one study with 55 patients, WOMAC pain scores at one year were similar in patients receiving single-shot FNB and untreated controls[43]. All patients received local anaesthetic infiltration (LIA) and patient-controlled analgesia (PCA). In another study with all participants receiving LIA, 150 were randomised to receive single-shot FNB with or without sciatic nerve block (SNB), or general anaesthesia[37]. There were no differences in HSS scores between groups at six months. Continuous FNB was compared with oral hydrocodone opioid in 62 patients receiving PCA[39]. There was some evidence for ‘pain using stairs’ favouring hydrocodone (p=0.01) but no difference in overall NRS-rated pain at one year and concern over venous thromboembolism in 4/31 participants treated with hydrocodone.

In two RCTs, continuous FNB was compared with PCA. In one study with 60 participants, the KSS at six months was similar between groups[44]. In another study with 280 participants, there was some evidence for higher incidence of NRS-rated pain at six months in the PCA group than the FNB group (p=0.021) but not at 12 months (p=0.273).[40]

Two RCTs compared FNB with LIA. In one study, all 157 participants also received PCA[36]. At one year, KSS values were similar in single-shot FNB and LIA groups. In the other study, 94 participants were randomised to receive single-shot FNB with continuous epidural infusion or LIA through an intra-articular catheter[41]. VAS-rated pain was similar between groups at one year.

In two RCTs, FNB procedures were compared. In one study with 99 patients randomised to two FNB concentrations, there was no difference in WOMAC score between groups at 12 months[34]. In another study with 61 participants allocated to two different durations of FNB,

For peer review only - http://bmjopen.bmj.com/site/about/guidelines.xhtml

there was no difference in WOMAC pain scores at one year[35]. In these studies, all participants received either SNB[34] or PCA[35].

Single-shot FNB was compared with single adductor canal block in one RCT with 98 participants, all receiving LIA[38]. At six months there was no difference in VAS-rated pain.

Sciatic nerve block

In one study, 89 patients were randomised to single-shot SNB, continuous SNB, or PCA[42]. All patients received FNB. At 12 months, there were no differences in pain for single-shot SNB and continuous SNB on the WOMAC pain scale or VAS-rated pain at rest or during mobilisation. Similarly, there were no differences between single-shot SNB and PCA in WOMAC pain scale or VAS-rated pain at rest or during mobilisation, or between continuous SNB and PCA.

Local anaesthetic infiltration

Four RCTs compared LIA with placebo. In one study, all 280 participants received FNB and PCA[50]. There was weak evidence that WOMAC pain scores were better in the LIA group at six (p=0.063) but not at 12 months (p=0.107) when the difference in means of 3.8/100 was lower than the MCID of 8-9/100 reported by Ehrich and colleagues[77]. In another study, 56 patients received LIA including ketorolac, or saline placebo, and all received PCA[47]. At one year, mean differences and confidence intervals provided weak evidence that OKS scores were better in the LIA group but the difference in means of 2.7/48 was less than the MCID of 4/48 reported by Beard and colleagues[78]. LIA before surgical incision was compared with placebo in one study with 120 participants[46]. None received FNB or PCA. There was weak evidence for a better KSS (function and knee score components) at six months in those receiving LIA (p=0.07) with a difference in means of 14.2/200 exceeding the MCID of 12.3/200 reported by Lee and colleagues[79]. In another study, all 51 participants received LIA intra-operatively, followed by PCA[49]. Those randomised to post-operative catheter-delivered LIA with ketorolac, or saline placebo had similar VAS-rated pain at six and 12 months.

LIA delivered as an injection and post-operative infusion was compared with epidural PCA in one study with 222 patients[45]. There was no difference between groups in OKS at 12 months.

In one study of 100 participants, LIA with or without corticosteroid were compared[48]. All patients received PCA. At two years there was no difference in OKS between groups.

Oral celecoxib

For peer review only - http://bmjopen.bmj.com/site/about/guidelines.xhtml

In one RCT, 44 participants received oral celecoxib or placebo[51], as well as PCA. There were no differences between groups in KOOS or VAS-rated pain at 12 months.

Ketamine or nefopam infusion

In one RCT, ketamine infusion, nefopam infusion and saline placebo were compared in 75 patients, all of whom received PCA[52]. VAS-rated pain on movement did not differ between groups at 12 months. For the Douleur Neuropathique 4 (DN4) measure of neuropathic pain, there was some evidence favouring ketamine over placebo at six and 12 months (p=0.02), but overall, few patients reported neuropathic pain at 12 months.

Pregabalin

Oral pregabalin was compared with placebo in one RCT with 240 participants[53]. All received LIA and PCA. At six months, there was some evidence for better NRS pain in patients receiving pregabalin compared with placebo (p=0.0176) but the difference in means of 0.54/10 was less than the MCID of 1/10 reported by Salaffi and colleagues[80]. No participants receiving pregabalin reported neuropathic pain when assessed using the S-LANSS, compared with 5.2% of those receiving placebo (p=0.014). Patients receiving pregabalin were more likely to be sedated and confused in the first two days after surgery.

Tourniquet

Five studies with 399 participants explored tourniquet use to provide a bloodless field. Two studies each were from Australia and China, and one from Denmark. All were conducted at a single centre with participants recruited between 2008 and 2015. Sample sizes ranged from 20 to 150 participants, with a median of 65. The range of mean ages of participants in randomised groups was 66 to 71 years and in 3/5 studies, a majority of participants were women.

In three RCTs, participants received TKR with or without a tourniquet. In one study with 64 patients, a difference in KOOS pain favouring tourniquet use was not significant at six or 12 months[54]. In another study with 20 patients, the OKS was not significantly different between groups at six or 12 months[56]. There were three blood transfusions in the tourniquet group, compared with none in the ‘no tourniquet’ group. In the third study with 100 participants, VAS-rated pain and HSS scores were similar between groups at 6 months[55]. Six cases of wound ooze occurred in the tourniquet group.

In two RCTs, short and long-duration tourniquet use were compared. In one study with 65 participants, there was weak evidence based on graphical representation of means and

For peer review only - http://bmjopen.bmj.com/site/about/guidelines.xhtml

confidence intervals for improved OKS at 12 months in the long-duration group and the difference in means of 5/48[57] was greater than the MCID of 4/48. Adverse events were reported by 62% of participants receiving short-duration tourniquet compared with 38% in the long-duration group. The study was terminated early as 10 blood transfusions were required in the short-duration group compared with three in the long-duration group. In the second study with 150 participants, tourniquets were used in three different periods during surgery[58]. At six months, there were no differences between groups in HSS scores.

Blood conservation

Seven studies with 829 participants evaluated strategies to limit blood loss after TKR. Two studies were from Thailand, and one each from China, France, South Korea, the UK and the USA. All were conducted at a single centre with participants recruited between 2003 and 2015 when stated. Sample sizes ranged from 48 to 180 participants, with a median of 106. One study had three trial arms. The range of mean ages of participants in randomised groups was 65 to 74 years and in all studies, a majority of participants were women.

Tranexamic acid

Five RCTs evaluated tranexamic acid.

Tranexamic acid injections or infusions were compared with saline placebo or untreated control in four RCTs[55,61,64,65]. In all studies, control patients required more blood transfusions. In one study including 180 participants comparing intravenous tranexamic acid with untreated controls, there was no significant difference in WOMAC pain scores at one year[61]. In another study with 48 participants comparing intra-articular tranexamic acid injection with saline placebo, there was no significant difference in WOMAC scores at six months[64]. One study with 135 participants compared two intra-articular tranexamic acid doses and saline control[65]. There were no significant differences in WOMAC scores at one year. Intravenous and intra-articular tranexamic was compared with untreated controls in one study with 100 participants[55]. VAS-rated pain at six months was similar between groups, but there was strong evidence favouring tranexamic acid for HSS scores ($p<0.001$) although the difference in means of 1.4/100 was lower than the MCID of 8.3/100 reported by Singh and colleagues[81].

In one study, continuous tranexamic acid infusion was compared with a single bolus in 106 patients[60]. There was no difference between groups in KSS at six months or blood loss.

Thrombin infusion

For peer review only - http://bmjopen.bmj.com/site/about/guidelines.xhtml

In one RCT with 80 participants, thrombin infusion was compared with untreated control[62]. At one year there was no difference between groups in pain measured on the KSS.

Flexion or extension

For blood management, operated knees were kept in passive flexion or passive extension after surgery in one RCT with 180 patients[63]. At one year, OKS was similar between groups. Transfusion requirement was greater in patients with passive extension.

Compression bandage

One RCT conducted at a single UK centre with 49 participants recruited between 2013 and 2014 compared compression bandaging to reduce post-operative knee swelling with standard bandaging. The mean age of participants was about 69 years and a majority were women. OKS was similar in randomised groups at six months[59].

Wound management

One RCT with recruitment in 2011 at a single centre in South Korea evaluated a wound care strategy to limit post-operative scar pain. The mean age of participants was about 69 years and a majority were women. Investigators compared silicone gel application to the surgical scar with placebo in 100 participants[75]. There were no significant differences in VAS-rated pain at six and 12 months.

Denusomab

One RCT evaluated use of the antiresorptive monoclonal antibody Denusomab to promote bone healing. The study was conducted in two centres in Sweden with recruitment of 50 participants between 2012 and 2014. The mean age of participants was about 65 years and a majority were women. At 12 and 24 months there were no significant differences between groups in KOOS pain[66].

Continuous passive motion

Two RCTs with 237 participants evaluated use of continuous passive motion (CPM) to minimise joint stiffness and improve range of movement. Studies were conducted in single centres in Australia and Turkey with participant recruitment between 1997 and 2004 and both had three trial arms. Sample sizes were 90 and 147 participants. The mean ages of participants in studies were about 63 and 72 years and a majority of participants were women. In one study, 90 participants were randomised to no CPM, CPM at low flexion from post-operative day 1–7, or

For peer review only - http://bmjopen.bmj.com/site/about/guidelines.xhtml

CPM at high flexion from post-operative day 3–7[68]. There was no significant difference between groups in KSS at two years. In the other study, 147 participants were randomised to CPM with increasing range of movement from day 1–6, early flexion CPM from day 0–6, or no CPM[67]. There were no significant differences between groups in KSS at 12 months.

Electrical stimulation

Two RCTs with 106 participants conducted in single centres in Greece and Italy evaluated electrical stimulation which is believed to have anti-inflammatory activity and limit muscle atrophy. Studies included 76 and 30 participants recruited between 2005 and 2010. The mean ages of participants were 71 and 70 years and in one study that reported it, a majority of participants were female.

In one study with 76 participants receiving transcutaneous electric muscle stimulation from post-operative day two for six weeks or no intervention, Short Form 36 bodily pain showed strong evidence for greater improvement at one year in the intervention group compared to control (p<0.001)[69]. The difference in means of 12.5/100 was close to the MCID of 16.9/100 reported by Escobar and colleagues[82]. There were no differences in OKS or KSS scores. In another study with 30 participants, pulsed electromagnetic fields from post-operative day 7 were compared with untreated control[70]. At 12 months, there was some evidence that VAS-rated pain was lower in intervention patients compared with controls (p<0.05). The difference in means of 2.1/10 was greater than the MCID of 16.1/100 reported by Danoff and colleagues[83]. Knee swelling was common during the intervention.

Rehabilitation

Four RCTs with 585 participants recruited between 2000 and 2016 evaluated features of early rehabilitation focusing on regaining range of movement, functional independence and improving mobility. Two studies were conducted at single centres in China and at two and four centres in Canada and Germany respectively. Sample sizes ranged from 80 to 234 participants, with a median of 136. The range of mean ages of participants in randomised groups was 68 to 78 years and in 3/4 studies, a majority of participants were women.

Walking guidance and training

In one study, 86 participants were randomised to walking guidance and training from post-operative day two or no intervention further to standard rehabilitation[71]. At six months, there was some evidence that those receiving intervention had lower VAS-rated pain (p<0.01) and

For peer review only - http://bmjopen.bmj.com/site/about/guidelines.xhtml

HSS score (p<0.01) than controls. The difference in mean VAS-rated pain of 2.4/10 was greater than the MCID of 16.1/100.

Flexion or extension during knee closure

Targeting improved functional recovery, wound closure performed in 90° flexion was compared with wound closure in full extension in one study with 80 participants[74]. There was no difference between groups in VAS-rated pain at six months.

Aquatic therapy

In one study with 185 participants, aquatic therapy commencing on post-operative day six was compared with aquatic therapy commencing on day 14[72]. Patients reported similar WOMAC pain at 12 and 24 months.

Supported early discharge

In one study, early discharge supported by physiotherapist home visits and outpatient or self-directed physiotherapy was compared with two weeks of rehabilitation centre-based usual care[73]. The study included 234 individuals receiving TKR or total hip replacement. Compared with usual care, there was weak evidence that patients with early discharge had lower WOMAC pain scores at 12 months (p=0.08). The difference in means of 4 was less than the MCID of 8-9/100. Results were not presented separately but did not differ between patients with TKR or total hip replacement.

Anabolic steroids

Searches identified one study of anabolic steroids to improve post-operative muscle strength conducted in one centre in Australia with recruitment of 10 participants before 2010. The mean age of participants was about 66 years and a minority were women. Participants received intramuscular nandrolone injections or saline from post-operative day five for six months. KSS results indicated some evidence for improvement in the intervention group compared with controls at 12 months (p=0.03)[76]. The difference in means of 10.2/200 was close to the MCID of 12.3/200.

Interventions with no long-term outcome

Interventions with lack of RCT evidence are summarised in Figure 1.

While 148 RCTs of DVT prophylaxis were identified, only five reported long-term follow up, none of which included a pain or outcome score. Among 29 RCTs of antibiotic prophylaxis, 16

For peer review only - http://bmjopen.bmj.com/site/about/guidelines.xhtml

reported long-term follow up, but none included a pain or outcome score. Six RCTs evaluated the use of bisphosphonates and, although all reported long-term follow up, none reported pain or an outcome score. One study reported long-term follow up of an RCT of teriparatide but included no data on pain.

For some interventions, RCTs with long-term pain outcomes were identified, but none were at low risk of bias: cold therapy; guided imagery; platelet rich plasma; and trigger point needling.

Aspects of peri-operative care evaluated in RCTs but lacking long-term pain follow up were: adenosine triphosphate; alternative and Chinese medicine; assistive devices; brain stimulation; calcium supplements; cardiovascular drugs; colloids and crystalloids; comorbidity management; constipation treatment; creatine; delirium prevention; dexmedetomidine; glucocorticoids; glucose infusion; iron; laser therapy; methylprednisolone; music therapy; nausea prevention; nutritional supplements; physiological treatments; remote ischaemic preconditioning; sleep treatments; therapy dogs; and warming.

DISCUSSION

Much research in TKR aims to identify treatments that facilitate a speedy recovery with minimal short-term pain. However, patients choose to have joint replacement for long-term pain relief and reduction in functional limitations. Thus, changes to peri-operative care, supported by short-term RCT evidence, should be backed up with evidence about long-term effectiveness for reducing pain and reassurance that there are no long-term unfavourable consequences. To this end, we synthesised evidence from RCTs evaluating peri-operative interventions which have considered their long-term effects on pain outcomes.

Consistent with its status as a key peri-operative risk factor, a major focus of research into improving long-term pain after TKR has been through prevention of acute post-operative pain using multimodal analgesia. Our review provides good quality evidence for a small benefit for intra-articular LIA injections, as previously shown in short-term studies[31,84], oral pregabalin, oral opioids, and in relation to neuropathic pain, ketamine infusion. As well as potential benefits for reduced long-term pain, future studies will need to consider concerns associated with these interventions which may not have been identified in small studies including infection[31], venous thromboembolism[39] and sedation[53].

Nerve blocks are effective for managing peri-operative pain[85] but we identified no long-term benefit. In single studies, there was no benefit for nefopam infusion, oral celecoxib or LIA with additional corticosteroid. Regarding future studies, standardisation of the multi-modal regimen

For peer review only - http://bmjopen.bmj.com/site/about/guidelines.xhtml

will allow evaluation of extra or alternative components in multiple studies in different settings. With such an approach, convincing evidence will accrue to guide multimodal pain management.

Some interventions targeted the prevention of adverse events and facilitation of early mobilisation. Tranexamic acid is highly effective in reducing blood transfusions during TKR[86] and we found no evidence that tranexamic acid affects long-term pain or, consistent with registry studies[87,88], adverse events. Single RCTs of thrombin infusion and maintenance of knee in flexion to prevent blood loss showed no effect on long-term pain. Tourniquets improve intraoperative visualisation of the joint, reduce blood loss and facilitate cement fixation but are associated with nerve damage, delayed recovery, acute pain and need for analgesics[89,90]. The RCTs we identified showed no effects of tourniquet use on long-term pain.

As shown in a previous review[91], there was no suggestion that CPM affects long-term pain. There was good quality evidence for a small benefit for reduced long-term pain in patients receiving walking training, anabolic steroid injection, electrical stimulation and supported discharge.

For some interventions a direct mechanism is clear, but for others, reasons for long-term impact are less obvious. This may explain why, for example, no studies evaluated DVT prophylaxis with long-term follow up excepting a small number reporting adverse events. However, treatments to prevent symptomatic DVTs which occur in about 1% of treated patients[92] also reduce the incidence of asymptomatic DVT observed in about 28% of treated patients[93] and this may have long-term benefits. Conversely, new anticoagulants are associated with bleeding[94], which may increase the risk of wound complications[95] and joint infection[96] which are associated with long-term pain[97,98].

Our study is limited by the lack of meta-analysis which was not appropriate due to intervention and outcome heterogeneity. In the context of perioperative pain management, this was noted previously[84]. Our approach to assessing the evidence was a narrative synthesis of studies with low risk of bias. While this may seem overly restrictive, Cochrane risk of bias assessment allows us to screen out studies with important issues that may affect the validity of results. The main potential source of bias was incomplete outcome assessment. Although studies with long-term follow up are naturally at higher risk of missing data, we maintained a standard in this domain as it is recognised that research participants who do not complete follow up assessments differ in outcomes from those with follow up data and their inclusion could change the interpretation of results[99].

For peer review only - http://bmjopen.bmj.com/site/about/guidelines.xhtml

Another limitation is that pain assessed with questionnaires does not take into account the effect of pain medications and assistive aids. About 58% of women and 40% of men report taking pain medications after TKR because of pain in the operated knee[100] and we must recognise that pain levels at follow up without this treatment might be considerably higher. Even with treatment, around 20% of patients report chronic pain after TKR[10] and in the context of a blinded RCT we should expect to be able to identify effects of peri-operative treatments.

We summarised p-values to assess the strength of evidence but, as statistically strong evidence may not reflect clinically important results[101], where possible we also compared effect sizes with MCIDs. Our review considered a diverse range of interventions at a specific time in the TKR pathway and, as we were unable to make clinical practice recommendations, we did not adopt the GRADE system[102] for this review.

An alternative approach to the prevention of chronic pain after TKR is the individualisation of care based on pain phenotype, genetic, psychosocial and other factors[103]. An example of this might be the peri-operative treatment only of individuals with neuropathic pain with pregabalin, as opposed to the non-stratified provision in the RCT of Buvanendran and colleagues[53]. In an RCT with pregabalin provided to patients with painful HIV-neuropathy, while no overall benefit was seen, a group with hyperalgesia responded to pregabalin treatment[104].

Our systematic review of peri-operative interventions brings together evidence on interventions in the peri-operative phase of the TKR pathway. There was good quality evidence for some interventions of a small benefit for reduced long-term pain, and whilst not supportive of the inclusion of specific interventions in clinical practice, there are clearly areas that merit research. High quality studies assessing long-term pain after peri-operative interventions are feasible and necessary to ensure that patients with osteoarthritis achieve good long-term outcomes after TKR.

ACKNOWLEDGEMENT

We thank Dr Mario Moric for conducting additional analyses on the study of Buvanendran and colleagues.

AUTHOR CONTRIBUTIONS

All authors, ADB, JD, RG-H and AWB, contributed to the conception and design of the study. ADB, JD and VW undertook the systematic review. ADB and JD carried out the risk of bias

For peer review only - http://bmjopen.bmj.com/site/about/guidelines.xhtml

assessments. ADB drafted the article with revisions by JD, VW, RG-H and AWB. All authors approved the final version for publication.

ROLE OF THE FUNDING SOURCE

This article presents independent research funded by the National Institute for Health Research (NIHR) under its Programme Grants for Applied Research programme (RP-PG-0613-20001). This study was supported by the NIHR Biomedical Research Centre at University Hospitals Bristol NHS Foundation Trust and the University of Bristol. The views expressed in this publication are those of the authors and not necessarily those of the NHS, the National Institute for Health Research or the Department of Health and Social Care.

COMPETING INTERESTS STATEMENT

The authors report no competing interests.

DATA STATEMENT

All data relevant to the study are included in the article or uploaded as supplementary information.

Legend

Figure 1. Systematic review flow diagram

REFERENCES

1. Weinstein AM, Rome BN, Reichmann WM, et al. Estimating the burden of total knee replacement in the United States. *J Bone Joint Surg (Am)* 2013;95:385-92.
2. National Joint Registry for England, Wales, Northern Ireland and the Isle of Man: 15th Annual Report. Hemel Hempstead: NJR Centre 2018.
3. Scottish Arthroplasty Project. Annual report 2018. Edinburgh: NHS National Services Scotland; 2018.
4. Maradit Kremers H, Larson DR, Crowson CS, et al. Prevalence of total hip and knee replacement in the United States. *J Bone Joint Surg (Am)* 2015;97:1386-97.
5. Nilsdotter AK, Toksvig-Larsen S, Roos EM. Knee arthroplasty: are patients' expectations fulfilled? A prospective study of pain and function in 102 patients with 5-year follow-up. *Acta Orthop* 2009;80:55-61.

For peer review only - http://bmjopen.bmj.com/site/about/guidelines.xhtml

6. Scott CE, Howie CR, MacDonald D, et al. Predicting dissatisfaction following total knee replacement: a prospective study of 1217 patients. *J Bone Joint Surg (Br)* 2010;92:1253-8.
7. Bachmeier CJM, March LM, Cross MJ, et al. A comparison of outcomes in osteoarthritis patients undergoing total hip and knee replacement surgery. *Osteoarthritis Cart* 2001;9:137-46.
8. Dailiana ZH, Papakostidou I, Varitimidis S, et al. Patient-reported quality of life after primary major joint arthroplasty: a prospective comparison of hip and knee arthroplasty. *BMC Musculoskelet Disord* 2015;16:366.
9. Werner MU, Kongsgaard UE. Defining persistent post-surgical pain: is an update required? *Br J Anaesth* 2014;113:1-4.
10. Blom AW, Artz N, Beswick AD, et al. Improving patients' experience and outcome of total joint replacement: The RESTORE programme. *Programme Grants Appl Res* 2016;4.
11. Lundblad H, Kreicbergs A, Jansson KA. Prediction of persistent pain after total knee replacement for osteoarthritis. *J Bone Joint Surg (Br)* 2008;90-B:166-71.
12. Wylde V, Sayers A, Odutola A, et al. Central sensitization as a determinant of patients' benefit from total hip and knee replacement. *Eur J Pain* 2017;21:357-65.
13. Mandalia V, Eyres K, Schranz P, et al. Evaluation of patients with a painful total knee replacement. *J Bone Joint Surg (Br)* 2008;90:265-71.
14. Hofmann S, Seitlinger G, Djahani O, et al. The painful knee after TKA: a diagnostic algorithm for failure analysis. *Knee Surg Sports Traumatol Arthrosc* 2011;19:1442-52.
15. Toms AD, Mandalia V, Haigh R, et al. The management of patients with painful total knee replacement. *J Bone Joint Surg (Br)* 2009;91:143-50.
16. Wylde V, Trela-Larsen L, Whitehouse MR, et al. Preoperative psychosocial risk factors for poor outcomes at 1 and 5 years after total knee replacement. *Acta Orthop* 2017;88:530-6.
17. Burns LC, Ritvo SE, Ferguson MK, et al. Pain catastrophizing as a risk factor for chronic pain after total knee arthroplasty: A systematic review. *J Pain Res* 2015;8:21-32.
18. Edwards RR, Haythornthwaite JA, Smith MT, et al. Catastrophizing and depressive symptoms as prospective predictors of outcomes following total knee replacement. *Pain Res Manag* 2009;14:307-11.
19. Wylde V, Beswick AD, Dennis J, et al. Post-operative patient-related risk factors for chronic pain after total knee replacement: a systematic review. *BMJ Open* 2017;7:e018105.
20. Lewis GN, Rice DA, McNair PJ, et al. Predictors of persistent pain after total knee arthroplasty: a systematic review and meta-analysis. *Br J Anaesth* 2015;114:551-61.

For peer review only

21. Wang L, Lee M, Zhang Z, et al. Does preoperative rehabilitation for patients planning to undergo joint replacement surgery improve outcomes? A systematic review and meta-analysis of randomised controlled trials. *BMJ Open* 2016;6:e009857.

22. Bay S, Kuster L, McLean N, et al. A systematic review of psychological interventions in total hip and knee arthroplasty. *BMC Musculoskelet Disord* 2018;19:201.

23. Riddle DL, Keefe FJ, Ang DC, et al. Pain coping skills training for patients who catastrophize about pain prior to knee arthroplasty: A multisite randomized clinical trial. *J Bone Joint Surg (Am)* 2019;101:218-27.

24. Kehlet H, Jensen TS, Woolf CJ. Persistent postsurgical pain: risk factors and prevention. *Lancet* 2006;367:1618-25.

25. Haroutiunian S, Nikolajsen L, Finnerup NB, et al. The neuropathic component in persistent postsurgical pain: A systematic literature review. *Pain* 2013;154:95-102.

26. Ashrani AA, Heit JA. Incidence and cost burden of post-thrombotic syndrome. *J Thromb Thrombolysis* 2009;28:465-76.

27. Dreyer HC. Tourniquet use during knee replacement surgery may contribute to muscle atrophy in older adults. *Exerc Sport Sci Rev* 2016;44:61-70.

28. Kindsfater K, Scott R. Recurrent hemarthrosis after total knee arthroplasty. *J Arthroplasty* 1995;10:S52-S5.

29. Epstein NE. A review article on the benefits of early mobilization following spinal surgery and other medical/surgical procedures. *Surg Neurol Int* 2014;5:S66-73.

30. Moher D, Liberati A, Tetzlaff J, et al. Preferred reporting items for systematic reviews and meta-analyses: the PRISMA statement. *J Clin Epidemiol* 2009;62:1006-12.

31. Marques EM, Jones HE, Elvers KT, et al. Local anaesthetic infiltration for peri-operative pain control in total hip and knee replacement: systematic review and meta-analyses of short- and long-term effectiveness. *BMC Musculoskelet Disord* 2014;15:220.

32. Higgins JPT, Green S. Cochrane Handbook for Systematic Reviews of Interventions Version 5.1.0 [Updated March 2011]: The Cochrane Collaboration; 2011. Available from: http://www.cochrane-handbook.org.

33. Sterne JA, Davey Smith G. Sifting the evidence-what's wrong with significance tests? *BMJ* 2001;322:226-31.

34. Albrecht E, Morfey D, Chan V, et al. Single-injection or continuous femoral nerve block for total knee arthroplasty? *Clin Orthop Relat Res* 2014;472:1384-93.

35. Choy WS, Lee SK, Kim KJ, et al. Two continuous femoral nerve block strategies after TKA. *Knee Surg Sports Traumatol Arthrosc* 2011;19:1901-8.

For peer review only - http://bmjopen.bmj.com/site/about/guidelines.xhtml

36. Fan L, Yu X, Zan P, et al. Comparison of local infiltration analgesia with femoral nerve block for total knee arthroplasty: A prospective, randomized clinical trial. *J Arthroplasty* 2016;31:1361-5.

37. Gao WL, Li H, Liu BQ, et al. Analgesic effect of femoral and sciatic nerve block under multimodal analgesia in total knee arthroplasty. *Chinese Journal of Tissue Engineering Research* 2017;21:2966-72.

38. Macrinici GI, Murphy C, Christman L, et al. Prospective, double-blind, randomized study to evaluate single-injection adductor canal nerve block versus femoral nerve block: postoperative functional outcomes after total knee arthroplasty. *Reg Anesth Pain Med* 2017;42:10-6.

39. Nader A, Kendall MC, Wixson RL, et al. A randomized trial of epidural analgesia followed by continuous femoral analgesia compared with oral opioid analgesia on short- and long-term functional recovery after total knee replacement. *Pain Med* 2012;13:937-47.

40. Peng L, Ren L, Qin P, et al. Continuous femoral nerve block versus intravenous patient controlled analgesia for knee mobility and long-term pain in patients receiving total knee replacement: a randomized controlled trial. *Evid Based Complement Alternat Med* 2014;2014:569107.

41. Reinhardt KR, Duggal S, Umunna BP, et al. Intraarticular analgesia versus epidural plus femoral nerve block after TKA: A randomized, double-blind trial. *Clin Orthop Relat Res* 2014;472:1400-8.

42. Wegener JT, van Ooij B, van Dijk CN, et al. Long-term pain and functional disability after total knee arthroplasty with and without single-injection or continuous sciatic nerve block in addition to continuous femoral nerve block: a prospective, 1-year follow-up of a randomized controlled trial. *Reg Anesth Pain Med* 2013;38:58-63.

43. Widmer BJ, Scholes CJ, Pattullo GG, et al. Is femoral nerve block necessary during total knee arthroplasty?: a randomized controlled trial. *J Arthroplasty* 2012;27:1800-5.

44. Wu JWS, Wong YC. Elective unilateral total knee replacement using continuous femoral nerve blockade versus conventional patient-controlled analgesia: perioperative patient management based on a multidisciplinary pathway. *Hong Kong Med J* 2014;20:45-51.

45. McDonald DA, Deakin AH, Ellis BM, et al. The technique of delivery of peri-operative analgesia does not affect the rehabilitation or outcomes following total knee arthroplasty. *Bone Joint J* 2016;98-B:1189-96.

For peer review only

For peer review only - http://bmjopen.bmj.com/site/about/guidelines.xhtml

46. Motififard M, Omidian A, Badiei S. Pre-emptive injection of peri-articular-multimodal drug for post-operative pain management in total knee arthroplasty: a double-blind randomized clinical trial. *Int Orthop* 2017;41:939-47.

47. Niemeläinen M, Kalliovalkama J, Aho A, et al. Single periarticular local infiltration analgesia reduces opiate consumption until 48 hours after total knee arthroplasty. A randomized placebo-controlled trial involving 56 patients. *Acta Orthop* 2014;85:614-9.

48. Seah VWT, Chin PL, Chia SL, et al. Single-dose periarticular steroid infiltration for pain management in total knee arthroplasty: a prospective, double-blind, randomised controlled trial. *Singapore Med J* 2011;52:19-23.

49. Williams D, Petruccelli D, Paul J, et al. Continuous infusion of bupivacaine following total knee arthroplasty: a randomized control trial pilot study. *J Arthroplasty* 2013;28:479-84.

50. Wylde V, Lenguerrand E, Gooberman-Hill R, et al. Effect of local anaesthetic infiltration on chronic postsurgical pain after total hip and knee replacement: the APEX randomised controlled trials. *Pain* 2015;156:1161-70.

51. Meunier A, Lisander B, Good L. Effects of celecoxib on blood loss, pain, and recovery of function after total knee replacement: a randomized placebo-controlled trial. *Acta Orthop* 2007;78:661-7.

52. Aveline C, Roux AL, Hetet HL, et al. Pain and recovery after total knee arthroplasty: a 12-month follow-up after a prospective randomized study evaluating Nefopam and Ketamine for early rehabilitation. *Clin J Pain* 2014;30:749-54.

53. Buvanendran A, Kroin JS, Della Valle CJ, et al. Perioperative oral pregabalin reduces chronic pain after total knee arthroplasty: a prospective, randomized, controlled trial. *Anesth Analg* 2010;110:199-207.

54. Ejaz A, Laursen AC, Kappel A, et al. Faster recovery without the use of a tourniquet in total knee arthroplasty. *Acta Orthop* 2014;85:422-6.

55. Huang Z, Xie X, Li L, et al. Intravenous and topical tranexamic acid alone are superior to tourniquet use for primary total knee arthroplasty: A prospective, randomized controlled trial. *J Bone Joint Surg (Am)* 2017;99:2053-61.

56. Liu D, Graham D, Gillies K, et al. Effects of tourniquet use on quadriceps function and pain in total knee arthroplasty. *Knee Surg Relat Res* 2014;26:207-13.

57. Mittal R, Ko V, Adie S, et al. Tourniquet application only during cement fixation in total knee arthroplasty: a double-blind, randomized controlled trial. *ANZ J Surg* 2012;82:428-33.

For peer review only

For peer review only - http://bmjopen.bmj.com/site/about/guidelines.xhtml

58. Zhang Y, Li D, Liu P, et al. Effects of different methods of using pneumatic tourniquet in patients undergoing total knee arthroplasty: a randomized control trial. *Ir Jr Med Sci* 2017;186:953-9.

59. Brock T, Sprowson A, Muller S, et al. STICKS study - Short-sTretch Inelastic Compression bandage in Knee Swelling following total knee arthroplasty - a feasibility study. *Trials* 2017;18:6.

60. Hourlier H, Reina N, Fennema P. Single dose intravenous tranexamic acid as effective as continuous infusion in primary total knee arthroplasty: a randomised clinical trial. *Arch Orthop Trauma Surg* 2015;135:465-71.

61. Kim TK, Chang CB, Kang YG, et al. Clinical value of tranexamic acid in unilateral and simultaneous bilateral TKAs under a contemporary blood-saving protocol: a randomized controlled trial. *Knee Surg Sports Traumatol Arthrosc* 2014;22:1870-8.

62. Kusuma SK, Sheridan KC, Wasielewski RC. Use of bovine thrombin to reduce blood loss in primary total knee arthroplasty: a controlled randomized trial. *J Arthroplasty* 2013;28:1278-81.

63. Napier RJ, Bennett D, McConway J, et al. The influence of immediate knee flexion on blood loss and other parameters following total knee replacement. *Bone Joint J* 2014;96-B:201-9.

64. Sa-Ngasoongsong P, Channoom T, Kawinwonggowit V, et al. Postoperative blood loss reduction in computer-assisted surgery total knee replacement by low dose intra-articular tranexamic acid injection together with 2-hour clamp drain: a prospective triple-blinded randomized controlled trial. *Orthop Rev* 2011;3:e12.

65. Sa-Ngasoongsong P, Wongsak S, Chanplakorn P, et al. Efficacy of low-dose intra-articular tranexamic acid in total knee replacement; a prospective triple-blinded randomized controlled trial. *BMC Musculoskelet Disord* 2013;14:340.

66. Ledin H, Good L, Aspenberg P. Denosumab reduces early migration in total knee replacement. *Acta Orthop* 2017;88:255-8.

67. Bennett LA, Brearley SC, Hart JAL, et al. A comparison of 2 continuous passive motion protocols after total knee arthroplasty: a controlled and randomized study. *J Arthroplasty* 2005;20:225-33.

68. Ersözlü S, Sahin O, Ozgür A, et al. The effects of two different continuous passive motion protocols on knee range of motion after total knee arthroplasty: a prospective analysis. *Acta Orthop Traumato* 2009;43:412-8.

For peer review only - http://bmjopen.bmj.com/site/about/guidelines.xhtml

69. Avramidis K, Karachalios T, Popotonasios K, et al. Does electric stimulation of the vastus medialis muscle influence rehabilitation after total knee replacement? *Orthopedics* 2011;34:175.

70. Moretti B, Notarnicola A, Moretti L, et al. I-ONE therapy in patients undergoing total knee arthroplasty: a prospective, randomized and controlled study. *BMC Musculoskelet Disord* 2012;13:88.

71. Li L, Wang Z, Yin MH, et al. Effect of early gait training on the functional rehabilitation after total knee arthroplasty. *Chinese Journal of Tissue Engineering Research* 2017;21:4288-93.

72. Liebs T, Herzberg W, Rüther W, et al. Multicenter randomized controlled trial comparing early versus late aquatic therapy after total hip or knee arthroplasty. *Arch Phys Med Rehabil* 2012;93:192-9.

73. Mahomed NN, Davis AM, Hawker G, et al. Inpatient compared with home-based rehabilitation following primary unilateral total hip or knee replacement: a randomized controlled trial. *J Bone Joint Surg (Am)* 2008;90:1673-80.

74. Wang S, Xia J, Wei Y, et al. Effect of the knee position during wound closure after total knee arthroplasty on early knee function recovery. *J Orthop Surg Res* 2014;9:79.

75. Kong CG, Kim GH, Kim DW, et al. The effect of topical scar treatment on postoperative scar pain and pruritus after total knee arthroplasty. *Arch Orthop Trauma Surg* 2014;134:555-9.

76. Hohmann E, Tetsworth K, Hohmann S, et al. Anabolic steroids after total knee arthroplasty. A double blinded prospective pilot study. *J Orthop Surg Res* 2010;5:93.

77. Ehrich EW, Davies GM, Watson DJ, et al. Minimal perceptible clinical improvement with the Western Ontario and McMaster Universities osteoarthritis index questionnaire and global assessments in patients with osteoarthritis. *J Rheumatol* 2000;27:2635-41.

78. Beard DJ, Harris K, Dawson J, et al. Meaningful changes for the Oxford hip and knee scores after joint replacement surgery. *J Clin Epidemiol* 2015;68:73-9.

79. Lee WC, Kwan YH, Chong HC, et al. The minimal clinically important difference for Knee Society Clinical Rating System after total knee arthroplasty for primary osteoarthritis. *Knee Surg Sports Traumatol Arthrosc* 2017;25:3354-9.

80. Salaffi F, Stancati A, Silvestri CA, et al. Minimal clinically important changes in chronic musculoskeletal pain intensity measured on a numerical rating scale. *Eur J Pain* 2004;8:283-91.

81. Singh JA, Schleck C, Harmsen WS, et al. Validation of the hospital for special surgery knee questionnaire: Convergent validity, responsiveness and sensitivity to change. ACR/ARHP; San Diego, California2013. p. 1926.

For peer review only

For peer review only - http://bmjopen.bmj.com/site/about/guidelines.xhtml

82. Escobar A, Quintana JM, Bilbao A, et al. Responsiveness and clinically important differences for the WOMAC and SF-36 after total knee replacement. *Osteoarthritis Cart* 2007;15:273-80.

83. Danoff JR, Goel R, Sutton R, et al. How much pain is significant? Defining the minimal clinically important difference for the visual analog scale for pain after total joint arthroplasty. *J Arthroplasty* 2018;33:S71-S5.

84. Andersen LO, Kehlet H. Analgesic efficacy of local infiltration analgesia in hip and knee arthroplasty: a systematic review. *Br J Anaesth* 2014;113:360-74.

85. Chan E-Y, Fransen M, Parker DA, et al. Femoral nerve blocks for acute postoperative pain after knee replacement surgery. *Cochrane Database Syst Rev* 2014;5:CD009941.

86. Alshryda S, Sarda P, Sukeik M, et al. Tranexamic acid in total knee replacement: a systematic review and meta-analysis. *J Bone Joint Surg (Br)* 2011;93:1577-85.

87. Poeran J, Rasul R, Suzuki S, et al. Tranexamic acid use and postoperative outcomes in patients undergoing total hip or knee arthroplasty in the United States: retrospective analysis of effectiveness and safety. *BMJ* 2014;349.

88. Hallstrom B, Singal B, Cowen ME, et al. The Michigan experience with safety and effectiveness of tranexamic acid use in hip and knee arthroplasty. *J Bone Joint Surg (Am)* 2016;98:1646-55.

89. Noordin S, McEwen JA, Kragh JF, Jr., et al. Surgical tourniquets in orthopaedics. *J Bone Joint Surg (Am)* 2009;91:2958-67.

90. Horlocker TT, Hebl JR, Gali B, et al. Anesthetic, patient, and surgical risk factors for neurologic complications after prolonged total tourniquet time during total knee arthroplasty. *Anesth Analg* 2006;102:950-5.

91. Harvey LA, Brosseau L, Herbert RD. Continuous passive motion following total knee arthroplasty in people with arthritis. *Cochrane Database Syst Rev* 2014;2:CD004260.

92. Januel J-M, Chen G, Ruffieux C, et al. Symptomatic in-hospital deep vein thrombosis and pulmonary embolism following hip and knee arthroplasty among patients receiving recommended prophylaxis: a systematic review. *JAMA* 2012;307:294-303.

93. Schellong SM, Beyer J, Kakkar AK, et al. Ultrasound screening for asymptomatic deep vein thrombosis after major orthopaedic surgery: The VENUS study. *J Thromb Haemost* 2007;5:1431-7.

94. Gómez-Outes A, Terleira-Fernández AI, Suárez-Gea ML, et al. Dabigatran, rivaroxaban, or apixaban versus enoxaparin for thromboprophylaxis after total hip or knee replacement: systematic review, meta-analysis, and indirect treatment comparisons. *BMJ* 2012;344:e3675.

For peer review only - http://bmjopen.bmj.com/site/about/guidelines.xhtml

95. Freedman KB, Brookenthal KR, Fitzgerald RH, Jr., et al. A meta-analysis of thromboembolic prophylaxis following elective total hip arthroplasty. *J Bone Joint Surg (Am)* 2000;82-a:929-38.

96. Parvizi J, Ghanem E, Joshi A, et al. Does "excessive" anticoagulation predispose to periprosthetic infection? *J Arthroplasty* 2007;22:24-8.

97. Adelani MA, Johnson SR, Keeney JA, et al. Clinical outcomes following re-admission for non-infectious wound complications after primary total knee replacement. *Bone Joint J* 2014;96-b:619-21.

98. Cahill JL, Shadbolt B, Scarvell JM, et al. Quality of life after infection in total joint replacement. *J Orthop Surg* 2008;16:58-65.

99. Akl EA, Briel M, You JJ, et al. Potential impact on estimated treatment effects of information lost to follow-up in randomised controlled trials (LOST-IT): systematic review. *BMJ* 2012;344:e2809.

100. Hawker G, Wright J, Coyte P, et al. Health-related quality of life after knee replacement - Results of the Knee Replacement Patient Outcomes Research Team Study. *J Bone Joint Surg (Am)* 1998;80A:163-73.

101. Leopold SS, Porcher R. Editorial: threshold p values in orthopaedic research—we know the problem. What is the solution? *Clin Orthop Relat Res* 2018;476:1689-91.

102. Guyatt GH, Oxman AD, Kunz R, et al. Going from evidence to recommendations. *BMJ* 2008;336:1049-51.

103. Lavand'homme P, Thienpont E. Pain after total knee arthroplasty: a narrative review focusing on the stratification of patients at risk for persistent pain. *Bone Joint J* 2015;97-B:45-8.

104. Simpson DM, Schifitto G, Clifford DB, et al. Pregabalin for painful HIV neuropathy: a randomized, double-blind, placebo-controlled trial. *Neurology* 2010;74:413-20.

For peer review only

For peer review only - http://bmjopen.bmj.com/site/about/guidelines.xhtml

For peer review only

Identification — **Articles identified in searches of *The Cochrane Library*, MEDLINE, Embase, PsycINFO and CINAHL from inception to February 2018 (n = 9697)**

Screening — **Articles screened (n = 9697)** → **No relevance (n = 7364)**

Eligibility — **Potentially relevant (n = 2333)**

Intervention	N	In: low risk of bias	No pain/ score outcome	High/ unclear risk of bias	No long-term follow up	Abstract only	Additional publication	Protocol	Review	Retracted
Adenosine triphosphate	2	0	0	0	1	0	0	0	1	0
Alternative medicine	4	0	0	0	4	0	0	0	0	0
Anabolic steroids	2	1	0	0	0	0	0	0	1	0
Antibiotic prophylaxis	43	0	16	0	13	0	0	1	13	0
Assistive devices	2	0	0	0	2	0	0	0	0	0
Bisphosphonates	17	0	6	0	0	0	2	0	9	0
Blood management	355	7	10	1	209	0	0	4	124	0
Brain stimulation	3	0	0	0	3	0	0	0	0	0
Calcium supplement	1	0	0	0	1	0	0	0	0	0
Cardiovascular drugs	4	0	0	0	2	0	0	0	2	0
Chinese medicine	2	0	0	0	2	0	0	0	0	0
Cold therapy	30	0	0	1	24	0	0	0	5	0
Colloids and crystalloids	1	0	0	0	1	0	0	0	0	0
Comorbidity management	1	0	0	0	1	0	0	0	0	0
Compression	8	1	0	0	6	0	0	1	0	0
Constipation treatment	2	0	0	0	2	0	0	0	0	0
Continuous passive motion	56	2	8	7	23	1	0	1	14	0
Creatine monohydrate	1	0	0	0	1	0	0	0	0	0
Delirium prevention	4	0	0	0	3	0	0	0	1	0
Denusomab	1	1	0	0	0	0	0	0	0	0
Dexmedetomidine	1	0	0	0	1	0	0	0	0	0
Deep vein thrombosis prophylaxis	474	0	5	0	143	0	4	8	314	0
Electrical stimulation	37	2	0	3	20	0	2	0	10	0
Glucocorticoid	2	0	0	0	0	0	0	0	2	0
Glucose infusion	1	0	0	0	1	0	0	0	0	0
Guided imagery	5	0	0	1	4	0	0	0	0	0
Iron	7	0	0	0	6	0	0	0	1	0
Laser therapy	1	0	0	0	1	0	0	0	0	0
Methylprednisolone	3	0	0	0	3	0	0	0	0	0
Music therapy	9	0	0	0	9	0	0	0	0	0
Nausea prevention	11	0	0	0	9	0	2	0	0	0
Nutritional supplements	4	0	0	0	4	0	0	0	0	0
Pain management	987	20	5	12	711	1	20	9	207	2
Physiological	26	0	0	0	23	0	0	1	2	0
Platelet rich plasma	12	0	0	1	6	0	0	0	5	0
Rehabilitation	67	4	0	2	43	0	0	1	17	0
Remote ischaemic pre-conditioning	5	0	0	0	5	0	0	0	0	0
Sleep treatment	3	0	0	0	2	0	1	0	0	0
Teriparatide	1	0	1	0	0	0	0	0	0	0
Therapy dogs	1	0	0	0	1	0	0	0	0	0
Tourniquet use	100	5	3	3	67	0	2	1	19	0
Trigger point needling	1	0	0	1	0	0	0	0	0	0
Warming	19	0	0	0	16	0	0	0	3	0
Wound management	17	1	0	0	12	0	0	1	3	0
**Total**	**2333**	**44**	**54**	**32**	**1385**	**2**	**33**	**28**	**753**	**2**

Supplementary material. Search strategy as applied in MEDLINE on Ovid

1 randomized controlled trial/ or randomized controlled trial.pt.

2 controlled clinical trial.pt.

3 randomized.ab.

4 placebo.ab.

5 randomly.ab

6 trial.ab

7 randomised.tw

8 1 or 2 or 3 or 4 or 5 or 6 or 7

9 review/

10 'systematic review$'.mp

11 9 or 10

12 8 or 11

13 Arthroplasty, Replacement, Knee/

14 Knee Prosthesis/

15 (arthoplast$ adj3 knee$).mp. [mp=title, abstract, original title, name of substance word, subject heading word, keyword heading word, protocol supplementary concept word, rare disease supplementary concept word, unique identifier]

16 (knee$ adj3 replac$).mp. [mp=title, abstract, original title, name of substance word, subject heading word, keyword heading word, protocol supplementary concept word, rare disease supplementary concept word, unique identifier]

17 (knee adj3 implant$).mp. [mp=title, abstract, original title, name of substance word, subject heading word, keyword heading word, protocol supplementary concept word, rare disease supplementary concept word, unique identifier]

18 13 or 14 or 15 or 16 or 17

19 12 and 18

For peer review only - http://bmjopen.bmj.com/site/about/guidelines.xhtml

Supplementary material. All peri-operative interventions with long-term pain or score follow up

1. Pain management

Author<br>Country<br>Recruitment dates<br>Setting	Indication<br>Number randomised intervention; control<br>Age<br>% female	Common anaesthesia			Follow up<br>Losses to follow up intervention; control<br>Risk of bias issues<br>Key results
		Group 1 (intervention)	Group 2 (intervention)	Group C (control)	
FNB single vs No FNB					
Widmer et al. 2012[43]<br>Australia<br>Before 2012<br>2 surgeons	Elective primary unilateral TKR<br>27; 28<br>Median 72.1 (IQR 64.4, 76.5); 69.4 (63.4, 75.5)<br>44.4%; 44.4%	Premedication 1-3mg i.v. midazolam. Propofol induction and sevoflurane general anaesthetic.<br>LIA with 200mg ropivacaine and 0.5mg adrenaline in 100ml saline.<br>PCA 20µg fentanyl at 5-minute intervals on demand until morning POD2. Then, oral oxycodone SR 10mg every 12 hours. Daily COX II inhibitor and paracetamol 1g every 6 hours as tolerated. For breakthrough pain, 5-10mg oxycodone immediate release every 3 hours as needed.			1 year<br>No losses to follow up reported<br>Low risk of bias<br>WOMAC pain (high score favourable) at 1 year: FNB and LIA median 2.0 (IQR 0, 2.8); LIA no FNB 1.0 (0, 2.0). p=0.74<br>No adverse events occurred in either group
		Ultrasound guided FNB with 100mg ropivacaine in 30ml saline		Sham setup for FNB. No identification or injection of femoral sheath	
FNB single vs ONB vs Control					
Bergeron et al. 2009[105]<br>Canada<br>2005-2006<br>1 centre	Elective primary unilateral TKR<br>19; 20; 20<br>Mean 65.1 (SE 2.0); 72 (1.8); 67 (1.3)<br>79%; 80%; 75%	Intraoperative sedation with iv propofol at discretion of anaesthesiologist. Lumbar spinal anaesthesia with 12mg 0.5% bupivacaine.<br>Postoperative i.v. PCA with fentanyl 50µg/ml set to deliver 25µg every 5 min as needed.<br>Celecoxib 100mg and acetaminophen 650mg on arrival in recovery room and every 12 and 6 hrs respectively. Breakthrough medication with intramuscular ketorolac 10 mg every 4 hrs.			1 year<br>Overall 32 lost to follow up<br>High risk of bias: only 27/59 patients followed up due to resource limitations.<br>No difference in HSS pain at rest or during activity at 1 year between the study groups.

For peer review only

For peer review only - http://bmjopen.bmj.com/site/about/guidelines.xhtml

		FNB with stimulator. 20ml 0.5% bupivacaine with 1/200,000 epinephrine.	ONB with stimulator. 20ml 0.5% bupivacaine with 1/200,000 epinephrine.	No injection but inguinal area prepared, and sham block performed behind drapes.	No long-term complications attributable to anaesthetic regimen
FNB continuous low dose vs FNB continuous high dose vs No FNB					
Shum et al. 2009[106]<br>Singapore<br>Before 2009<br>1 hospital	Unilateral TKR for osteoarthritis<br>20 (17 received treatment); 20 (18 received treatment); 20<br>Mean 66.7 (SD 8.4); 65.4 (8.4); 67.8 (5.5)<br>88%; 72%; 80%	Spinal anaesthesia induced with 2-3ml hyperbaric 0.5% bupivacaine. Intraoperative sedation with midazolam in increments of 0.5mg.<br>Intravenous PCA morphine (1mg/ml, on-demand bolus doses of 1 mg with 5 minute lockout, maximum dose 8 mg/hr)			2 years<br>16.4% of patients who received intervention lost to follow up<br>Unclear risk of bias due to differences in OKS and weight at baseline, and limited methodological details.<br>No separate pain outcome but mean OKS slightly more favourable in group with no FNB, 18.2 (SD 3.7) compared with combined FNB groups, 19.8 (5.4) but this was not significant.<br>No complications attributable to use of FNB
		Low dose continuous FNB at conclusion of TKR with ropivacaine 0.15% (10 ml/hr in the first 24 hours, followed by 5ml/hr in the next 24 hours)	High dose continuous FNB at conclusion of TKR with ropivacaine 0.2% (10 ml/hr in the first 24 hours, followed by 5 ml/hr in the next 24 hours)	No FNB	
SNB injection vs SNB continuous vs control					
Wegener et al. 2013[42]<br>The Netherlands<br>2008-2010<br>1 centre	Unilateral TKR<br>29; 30; 30 (90 randomised)<br>Median 65 (range 43-81); 66 (43-83); 62 (50-79)<br>62%; 70%; 73%	Lorazepam 1mg 2 hours and acetaminophen 2g 1 hour before surgery. FNB with stimulating catheter: loading dose 20 ml levobupivacaine 0.375% and after 45 minutes a continuous infusion of levobupivacaine 0.125% 10 ml/hr. General anaesthesia induced with 3-5 µg/ml propofol infusion and remifentanil 0.5 µg/kg/min and maintained with 2-3 µg/ml at 0.1-0.25 µg/kg/min. Postoperatively, FNB changed to patient controlled FNB, 5ml bolus, 30-minute lockout; basal rate 6 ml/hr. i.v. morphine administered if needed. Postoperative analgesia with acetaminophen 1g 4 times daily. Diclofenac 50mg or tramadol 50mg 3 times daily. Tramadol 100mg before removal of nerve catheters. Morphine pain relief as required.			12 months<br>2;7;5 lost to follow up<br>Low risk of bias<br>Median WOMAC pain scores at 12 months: SNB injection 80 (range 25-100), SNB continuous 90 (55-100) and PCA only 90 (35-100), p=0.81. No difference between groups in VAS pain at rest (p=0.90) or during mobilisation (p=0.43).<br>No information on adverse events.

For peer review only

		Group Fs: SNB single injection. SNB loading dose of 20 ml levobupivacaine 0.375%.	Group FCS: SNB continuous infusion. SNB loading dose of 20 ml levobupivacaine 0.375%. Continuous infusion of levobupivacaine 0.125% 10 ml/hr started 45 mins after catheter placement. SNB maintained for 36 hours postoperatively (10 ml/hr).	Group F: No SNB. PCA via femoral nerve catheter	
**General anaesthesia vs FNB single vs FNB/ SNB single**					
Gao et al. 2017[37]<br>China<br>2014-2015<br>1 centre	Primary unilateral TKR for osteoarthritis<br>50; 50; 50<br>Mean 65.8 (SD 6.7); 66.4 (7.4); 67.6 (6.3)<br>81%; 80%; 76%	Pre-operative and post-operative celecoxib 0.2g twice daily.<br>100ml intra-operative LIA with ropivacaine 200mg and epinephrine 0.25 mg.			6 months<br>2; 1; 0<br>Low risk of bias<br>Mean HSS at 6 months: 87.1 (SD 6.9); 87.4 (7.3); 88.5 (6.7). No significant difference.<br>Nausea and vomiting: 4; 2; 1, urinary retention: 3; 1; 2.
		General anaesthesia	Ultrasound guided FNB 5g/l ropivacaine 20ml plus 0.1mg epinephrine	Ultrasound guided FNB 5g/l ropivacaine 20 ml plus 0.1mg epinephrine and SNB 5g/l ropivacaine 20ml plus 0.1mg epinephrine	
LIA no corticosteroid vs No LIA/ placebo					
Wylde et al. 2015 [50]<br>UK	Primary unilateral TKR for osteoarthritis	FNB with nerve stimulator and/ or ultrasound guidance (20ml 0.25% bupivacaine). Spinal or general anaesthetic. Intra-operative analgesia provided by titration of i.v. fentanyl initially and morphine if necessary. 1g intravenous			6 and 12 months<br>24;19 at 12 months (including those who did not receive treatment)

For peer review only

For peer review only - http://bmjopen.bmj.com/site/about/guidelines.xhtml

2009-2012<br>1 centre	157; 159 (143; 137 received treatment)<br>Mean 69.5 (SD 9.4); 68.7 (7.9)<br>52%; 54%	paracetamol 30 minutes before the end of operation. Immediately post-operative 400mg oral ibuprofen.<br>PCA with morphine 1mg/ml, 1 mg bolus dose and a 5-minute lock-out. If necessary morphine bolus up to 0.2mg/kg as rescue analgesia. During hospital stay, visit from pain specialist nurse. Oral or i.v. paracetamol every 6 hours and ibuprofen 400mg every 8 hours. When PCA no longer needed, oral codeine phosphate 30-60mg every 6 hours, tramadol 50-100mg every 6 hours and oramorph 10-20mg as rescue analgesia.		Low risk of bias<br>At 12 months WOMAC pain score (0-100) in LIA group median 90 (IQR 30), Control 85 (35); ITT-CC linear regression coefficient 3.83 (95%CI -0.83, 8.49), p=0.107. At 6 months WOMAC pain score ITT-CC linear regression coefficient 4.10 (95%CI -0.22, 8.43), p=0.063. Mean differences lower than MCID of 8-9[77].<br>Superficial and deep wound infection rate in LIA group 3.2% and 1.9% in control group, p=0.500. No differences in serious adverse events between groups
		60ml intra-operative LIA with 0.25% bupivacaine and 1/200,000 adrenaline injected into the posterior capsule, medial and lateral capsule, fascia and muscle, and subcutaneous tissues.	No treatment other than standard care	
Williams et al. 2013[49]<br>Canada<br>Before 2013<br>1 centre, 2 surgeons	Primary unilateral TKR for osteoarthritis<br>35; 32 (26; 25 received treatment)<br>Mean 66 (SD 9.7); 67 (12.5)<br>58%; 60%	Sedation with i.v. midazolam and propofol. Intraoperative LIA loading dose of 20ml 0.25% bupivacaine/ epinephrine injection, 10ml into medial and lateral subcutaneous tissue around the incision and 10ml intra-articular after closure. Infiltrate delivered by pain pump into lateral recess of intra-articular space. Spinal anaesthetic with 10-15 mg of 0.75% or 0.5% plain bupivacaine and 20µg fentanyl.<br>Postoperative morphine PCA. 7.5mg i.v ketorolac preoperatively plus 15mg every 6 hours postoperatively for 48 hours, then oral ketorolac 10mg every 6 hours for 2 days. Gabapentin 600mg given preoperatively plus 300mg twice daily for 48 hours postoperatively. Oxycodone 10mg twice daily for 48 hours postoperative. Oral paracetamol 650mg every 4 hours for 72 hours.		6 and 12 months<br>3;1 of those who received treatment<br>Low risk of bias<br>Mean VAS pain score at 6 months 1.2 (SD 1.3); 1.2 (1.2). p=0.836. At 12 months 0.9 (1.2); 1.0 (1.1). p=0.767<br>No short-term differences in adverse events except control patients more likely to be drowsy at 48 hrs. Long-term adverse events not reported.
		Infusion of 0.5% bupivacaine at 2ml/hr for 48 hrs	Infusion of saline at 2ml/hr for 48 hrs	
Niemeläinen et al. 2014[47]<br>Finland<br>2011-2012	Primary unilateral TKR for osteoarthritis<br>30; 30 (27; 29 received treatment)	Oral paracetamol 1g given 1 hour before surgery. Spinal anaesthesia with 15mg bupivacaine in 3ml.<br>After surgery oral paracetamol 1g every 6 hours and oral meloxicam (15mg) every 24 hours.<br>PCA with oxycodone 2mg, lock-out time 8 min.		12 months<br>1; 4<br>Low risk of bias<br>No pain measure separate from OKS. Weak evidence of more favourable

For peer review only - http://bmjopen.bmj.com/site/about/guidelines.xhtml

1 hospital	Mean 65 (SD 4.9); 64 (6.7)<br>56%; 48%	Rescue levobupivacaine medication through a lumbar epidural catheter		OKS (0-48) in the LIA group at 12 months, mean difference -2.7 (95% CI -5.48, 0.07). Difference lower than MCID of 4.0[78].<br>Infection: 0; 0. Severe pain treated with epidural analgesia: 0; 3. Nausea: 1; 1
		Intra-operative periarticular LIA of 100ml saline with levobupivacaine (150mg) mixed with ketorolac (30mg) and adrenaline (0.5mg).	Intra-operative periarticular LIA of 100ml saline	
Motififard et al. 2017[46]<br>Iran<br>2014-2015<br>1 hospital	Primary unilateral TKR for osteoarthritis<br>60; 60<br>Mean 66.4 (6.4); 64.5 (6.0)<br>86.0%; 94.3%	Spinal anaesthesia.<br>No FNB or SNB.<br>Pain medication provided as required after surgery: meloxicam (15 mg daily), celecoxib (400 mg daily), acetaminophen (1g every 8 hours), tramadol (50 mg every 8 hours), ketorolac (30 mg slow IV every 8 hours, with a 4-dose max), and morphine (5–10 mg slow IV if needed)		6 months<br>3; 7<br>Low risk of bias<br>No separate pain measure. Weak evidence for improved KSS (0-200) in LIA group at 6 months, mean 115.55 (SD 15.506); 101.40 (16.117). P=0.07. Difference of 14.15 greater than MCID of 12.3[79]. Difference was significant at 6 weeks, p<0.001.<br>No complications related to TKR or LIA. Low back pain (1; 2), stroke (0; 1), CHF (1; 0)
		Peri-articular injection, 15 minutes before incision, of 100ml saline containing 50 mg bupivacaine hydrochloride 0.5%, 1 ml morphine sulphate 10 mg/ml, 300 µg epinephrine (1:1000) and 30 mg ketorolac	100ml saline containing 300 µg epinephrine (1:1000)	
McDonald et al. 2016[45]<br>UK<br>2010-2011<br>1 hospital	Primary unilateral TKR for osteoarthritis<br>113; 109 received common spinal anaesthesia (121; 121 randomised)<br>Median 68 (IQR 62, 72); 67 (62, 73)<br>59%; 55%	Oral premedication with 10-20mg temazepam, 150mg ranatidine, 10mg dexamethasone, 300mg gabapentin, 1g paracetamol.<br>Spinal anaesthesia		12 months<br>9; 11 of those receiving treatments<br>Low risk of bias<br>No separate pain outcome. Mean OKS at 12 months: median 41 (IQR 35, 44); 41 (34;44). P=0.915<br>Suspected infection 2; 1. MI 0; 1. GI bleed 1; 0. renal failure 1; 0. Died 2; 0)
		Intra-articular and subcutaneous infiltration during surgery of 200 ml of 2mg/ml ropivacaine without adrenalin or additives. Catheter inserted, and 20 ml infiltrate injected following wound closure. Further boluses of 40 ml 2 mg/ml ropivacaine via infusion pump 4 hours after leaving theatre and morning of POD1. Two	Epidural PCA with 4 ml of 2.5 mg/ml levobupivacaine introduced at end of surgery. Thereafter self-medication with 2 ml of 1.25 mg/ml bupivacaine with 15 minutes lockout until morning of POD1. Nurse-administered rescue of 4 ml of 2.5 mg/ml levobupivacaine.	

For peer review only

		additional top ups of 40 ml 2mg/ml ropivacaine were prescribed if required.		
Celecoxib vs placebo				
Meunier et al. 2007[51]<br>Sweden<br>2004-2005<br>1 centre	Elective primary unilateral TKR for osteoarthritis<br>25; 25 (24; 20 received treatment)<br>Mean 68 (SD 6.3); 69 (7.7)<br>71%; 40%	Spinal anaesthesia with bupivacaine 17.5-20mg. i.v. midazolam or propofol sedation if needed. Paracetamol 1 g preoperatively and then with tramadol 50-100 mg 4 times a day during hospital stay. Ketobemidone (2.5-5mg i.v. or subcutaneous) on demand. Paracetamol and tramadol used as required after discharge.		12 months<br>No losses to follow up after surgery reported<br>Low risk of bias<br>No effect of celecoxib on VAS or KOOS pain at 1 year.<br>DVT: 0; 1. Deep infection: 0; 0.
		Oral celecoxib 200mg 1 hour preoperatively and twice daily for 3 weeks	Oral placebo 200mg 1 hour preoperatively and twice daily for 3 weeks	
Ketamine vs placebo				
Perrin and Purcell2009 [107]<br>Australia<br>Before 2009<br>1 centre (pilot study)	Elective unilateral TKR<br>16 (5; 7 completed study per protocol)<br>Mean 65.6 (SD 10.2); 60.3 (11.9)<br>40%; 43%	Intrathecal injection of 15mg bupivacaine and 100μg morphine. General anaesthesia. After surgery 1.5g paracetamol and then 750mg every 4 hours; PCA with morphine 2mg boluses with 10-minute lockout; morphine rescue 2.5mg intravenously as required; and rescue oral ibuprofen 800mg.		6 months<br>3 protocol breaches and 1 patient with uncontrolled pain.<br>High risk of bias due to non-ITT reporting and recruitment difficulties<br>2/5 ketamine group had mild/moderate pain on the WOMAC pain scale at 26 weeks or failed to improve compared with 5/7 controls.<br>1 adverse psycho-mimetic effect not attributed to intervention or control treatment
		Ketamine 0.5mg/kg bolus followed by 4μg/kg/min infusion. Commenced before surgical incision and continued until wound bandaged or syringe empty.	Saline infusion. Commenced before surgical incision and continued until wound bandaged or syringe empty.	
Ketamine vs Nefopam vs placebo				
Aveline et al. 2014[52]<br>France<br>2005<br>1 centre	Elective primary unilateral TKR for osteoarthritis<br>25; 25; 25<br>Mean 73 (SD 9); 72 (9); 70 (7)<br>67%; 60%; 63%	General anaesthesia induced with 1.5-2mg/kg propofol, 1μ/kg remifentanil and a single bolus of cisatracurium 0.15mg/kg. Remifentanil infusion at 0.15μg/k/min until skin closure. Anaesthesia maintained with sevoflurane 0.9-1.2% with 50% nitrogen in oxygen. 20 mins before skin closure, 0.15mg/kg i.v. morphine bolus and 0.625mg droperidol. PCA with morphine hydrochloride 1 mg i.v. bolus with 7-min lockout. On arrival in recovery room, 3 mg i.v. morphine boluses at 5 minute intervals.		6 and 12 months<br>3; 1; 2<br>Low risk of bias<br>Median DN4 at 12 months: 1 (IQR 1, 2); 1 (0, 1); 2 (1, 3). p=0.02 for difference between ketamine and placebo groups. Number of patients with VAS pain on movement score

For peer review only - http://bmjopen.bmj.com/site/about/guidelines.xhtml

		0.2mg/kg nefopam administered over 20 min before incision; 2mg/ml nefopam continuous infusion at 120µg/kg/hr until end of surgery and 60µg/kg/hr for 48 hours	0.2mg/kg ketamine administered over 20 min before incision; 2mg/ml ketamine continuous infusion at 120µg/kg/hr until end of surgery and 60µg/kg/hr for 48 hours	Saline administered over 20 minutes before incision; saline continuous infusion until second post-operative day	≥40mm at 12 months by group: nefopam (3/22, 13.7%), ketamine (3/24, 12.5%), and placebo group (6/23, 26.1%). Ketamine reduced DN4 pain (p=0.02) compared with placebo. At 12 months only 7/69 patients had DN4≥4 indicative of neuropathic pain.<br>Infection: 0; 0; 0. Revision: 0; 0; 0.
Pregabalin vs placebo					
Buvanendran et al. 2010[53]<br>USA<br>2006-2007<br>Single centre	Primary unilateral TKR for osteoarthritis.<br>120; 120 (9; 2 did not receive post-operative treatment but ITT analysis)<br>Mean 64.0 (SD 8.3); 63.3 (8.9)<br>76%; 70%	Sedation with midazolam and i.v. propofol. Combined spinal-epidural anaesthetic. 1.5ml 0.75% hyperbaric bupivacaine with 25µg fentanyl injected intrathecally. Catheter inserted for epidural drug administration.<br>LIA 60 ml 0.25% bupivacaine with epinephrine infiltrated into the wound at capsule closure. From completion of surgery until 32-42 hours post-operative, epidural infusion of fentanyl (5µg/ml) and bupivacaine (1mg/ml) initiated using continuous basal infusion of 6ml/hr with epidural PCA bolus doses (maximum 10ml/hr). Patients transitioned to oral opioid (morphine, oxycodone, and hydromorphone) as required. All patients received preoperative oral celecoxib 400mg 1–2 hours before surgery and 200mg twice daily for 3 days in hospital.			6 months<br>7; 5<br>Low risk of bias<br>Mean VRS pain score at 6 months: pregabalin 0.41 (SD 1.20); control 0.95 (1.80). p=0.0084. Distributions skewed but nonparametric Wilcoxon significant (p=0.0176). Difference of 0.54 less than MCID of 1.0.<br>In the pregabalin group the incidence of neuropathic pain measured using S-LANSS was 0% (0/113) and 5.2% (6/115) in the placebo group (p=0.014).<br>No clinically significant adverse events up to 6 months and no falls. Sedation, confusion and dry mouth more frequent in pregabalin than placebo group on day of surgery and first postoperative day.
		Oral pregabalin 300mg 1–2 h before surgery, 150mg twice daily for the first 10 postoperative days, 75mg twice daily on days 11 and 12, and 50mg twice daily on days 13 and 14		Oral placebo 1–2 h before surgery, twice daily for the first 10 postoperative days, twice daily on days 11 and 12, and twice daily on days 13 and 14	
FNB long duration vs FNB short duration					

For peer review only - http://bmjopen.bmj.com/site/about/guidelines.xhtml

<table>
<tr>
<td rowspan="2">Ilfeld et al. 2009[108]
USA
2005-2007
2 centres</td>
<td rowspan="2">Primary unilateral TKR for osteoarthritis
25; 25
Median 66 (IQR 60, 70); 64 (60, 69)
56%; 60%</td>
<td colspan="2">Femoral catheter inserted using nerve stimulator. FNB with 0.2% ropivacaine infusion (8ml/hr basal; 4 ml patient-controlled bolus; 30-minute lockout) from surgery until a.m. POD1.
1 week oral acetaminophen (975mg every 6 hours), sustained release oral opioid (Oxycontin, 10mg every 12 hours), and either oral aspirin (650mg daily) or celecoxib (200mg every 12 hours). Oral oxycodone 5 mg tablets and/ or i.v. morphine sulfate 2-4 mg for breakthrough pain.</td>
<td rowspan="2">6 and 12 months
4; 1 lost to follow up
High risk of bias: uneven loss to follow up between groups; muscle weakness resulted in lower dose of infusion on POD1 (10 continuous; 3 saline)
Groups had similar WOMAC pain scores at 6 and 12 months (p>0.05).
MI: 1; 0. PE: 1; 0. Fall: 1; 0. Catheter leak, dislodged: 1; 2</td>
</tr>
<tr>
<td>At 6 a.m. POD1, infusion pump replaced with infusion pump with 0.2% ropivacaine.
At 6 p.m. POD2 pump replaced with portable infusion pump (400ml 0.2% ropivacaine). Catheter removed evening of POD4.</td>
<td>At 6 a.m. POD1, infusion pump replaced but saline substituted.
At 6 p.m. POD2 pump replaced with portable infusion pump (saline). Catheter removed evening of POD4</td>
</tr>
<tr>
<td rowspan="2">Ilfeld et al. 2011[109]
USA
2007-2009
2 centres</td>
<td rowspan="2">Primary unilateral cemented TKR for osteoarthritis
40; 40 (39; 38 included in RCT)
Median 61 (IQR 58, 67); 66 (60, 70)
67%; 66%</td>
<td colspan="2">Femoral catheter inserted using nerve stimulator. FNB with 0.2% ropivacaine infusion (6ml/hr basal; 4ml patient-controlled bolus; 30-min lockout) from surgery until POD1.
1 week oral acetaminophen (975mg every 6 hours), sustained release oral opioid (Oxycontin, 10mg every 12 hours), and either oral aspirin (650mg daily) or celecoxib (200mg every 12 hours). Oral (oxycodone 5mg or 10mg tablets) and/ or i.v. opioids (morphine sulfate 2-4mg) for breakthrough pain.</td>
<td rowspan="2">12 months
11; 12 incomplete follow up
High risk of bias: 11;12 did not have 4 measures out of 6 up to 12 months; graph suggests WOMAC pain lower pre-intervention in continuous infusion group.
No difference in WOMAC pain scores between randomised groups (p>0.05).
Falls: 4; 0</td>
</tr>
<tr>
<td>At 6 a.m. POD2, infusion pump replaced and 0.2% ropivacaine continued.
At 6 p.m. POD2 pump replaced with portable infusion pump (400ml 0.2% ropivacaine). Catheter removed evening of POD4</td>
<td>At 6 a.m. POD2, infusion pump replaced but saline substituted.
At 6 p.m. POD2 pump replaced with portable infusion pump (saline). Catheter removed evening of POD4</td>
</tr>
<tr>
<td>Choy et al. 2011[35]
Korea
2006-2007</td>
<td>Primary unilateral TKR for osteoarthritis</td>
<td colspan="2">Spinal anaesthesia. Continuous FNB via catheter until POD3. Catheter inserted with use of nerve stimulator. Analgesia induced with 20ml of 1:1 0.25% bupivacaine and 2% lidocaine with 1:200,000 epinephrine. Continuous</td>
<td>2 years
4; 3 lost to follow up</td>
</tr>
</table>

For peer review only

For peer review only - http://bmjopen.bmj.com/site/about/guidelines.xhtml

1 surgeon	33; 30 (2 patients received GA and excluded)<br>Mean 66.7 (SD 10); 67.5 (11)<br>97%; 93%	infusion with 0.125% bupivacaine 5.0ml/hr. i.v. PCA (butorphanol 4mg, ketorolac 150mg, saline 50ml), programmed to deliver 1 mg bolus (lockout 10 min) with maximum dose 6mg/hr. i.v. paracetamol 2g 4 times/ day and oral ibuprofen 600mg 3 times/ day for breakthrough pain<br>Continuous femoral nerve block via catheter continued from POD3 to POD7	Continuous femoral nerve block discontinued on POD3		Low risk of bias for 2 year outcome measures.<br>At 2 years, intervention WOMAC pain mean 7.2 (SD 2), control 6.3 (SD 1); p=0.2<br>Superficial infection: 1; 1
FNB continuous high concentration vs FNB low concentration vs FNB single					
Albrecht et al. 2014[34]<br>Canada<br>2009-2011<br>1 hospital	Scheduled primary unilateral TKR<br>32; 32; 35<br>Mean 61 (CI 57, 64); 63 (60, 67);63 (60, 66)<br>46%; 44%; 52%	Stimulating catheter inserted with ultrasound guidance. Immediately after catheter placement, 10ml mepivacaine 2% was injected through the catheter. SNB using 30 ml ropivacaine 0.2%. Spinal anaesthesia with 2.5 to 3.0 ml isobaric bupivacaine 0.5% and 0.1mg intrathecal morphine.<br>Bolus of 20ml ropivacaine 0.2% with epinephrine 1:400,000 into the femoral catheter followed by ropivacaine 0.2% at a rate of 5 ml/hr with patient-controlled boluses of 5ml available every 30minutes.	Bolus of 20 ml ropivacaine 0.2% with epinephrine 1:400,000 into femoral catheter followed by ropivacaine 0.1% at rate of 10ml/hr with patient-controlled boluses of 10 ml available every 30 minutes.	Bolus of 30ml ropivacaine 0.375% with epinephrine 1:400,000 into the femoral catheter followed by normal saline at a rate of 1 ml/hr with patient-controlled boluses of 1mL available every 30minutes.	12 months<br>4;0;2 lost to follow up<br>Low risk of bias.<br>No separate pain outcome. Mean WOMAC score at 12 months: high concentration FNB 17 (95% CI 7, 27); 22 (14, 30); 18 (8, 27). P=0.68<br>Falls: 0; 0; 1
FNB continuous vs Psoas compartment block vs FNB continuous and psoas compartment block					
Morin et al. 2005[110]<br>Germany<br>Before 2005<br>1 centre	Elective unilateral TKR<br>30; 30; 30	Oral pre-medication with 20mg chlorazepate. General anaesthesia with intravenous propofol and 4–8µg/kg i.v. fentanyl and desflurane in N2O. 100mg diclofenac suppository after anaesthesia induction and 2.5g intravenous metamizole before end of surgery. Post-operative 3 daily doses of oral diclofenac 50mg. i.v. PCA			9–12 months<br>7; 6; 5<br>High risk of bias due to large losses to follow up, non-blinded outcome collection, and differences between

For peer review only - http://bmjopen.bmj.com/site/about/guidelines.xhtml

	Median 68 (IQR 62, 74); 71 (63, 74); 65 (53, 73)<br>50%; 70%; 59%	with piritramide bolus 2mg as needed with lockout interval of 10 mins for 48 hours.			groups in BMI and anaesthetist's opinion of difficulty of catheter placement.<br>No difference between groups in level of pain at the knee joint during past 4 weeks: FNB median 2.5 (IQR 1, 4), FNB and SNB 2 (1, 4), Psoas block 2 (IQR 1, 4), p=0.44<br>No early complications but longer term adverse events not reported.
		Continuous FNB<br>Stimulating catheter used. Initial bolus of prilocaine 1% and ropivacaine 0.75%. 300mg prilocaine 1% (30ml) and 150mg ropivacaine 0.75% (20ml). During first 48hrs post-operative ropivacaine 0.2% infusion 14ml/hr.	Continuous FNB and continuous SNB<br>Stimulating catheter used. Initial bolus of prilocaine 1% and ropivacaine 0.75%. In each catheter: 200mg prilocaine 1% (20ml) and 75mg ropivacaine 0.75% (10ml). During first 48hrs post-operative infusion through each catheter of 0.2% ropivacaine 7ml/hr.	Continuous psoas compartment block<br>Stimulating catheter used. Initial bolus of prilocaine 1% and ropivacaine 0.75%. 300mg prilocaine 1% (30ml) and 150mg ropivacaine 0.75% (20ml). During first 48hrs post-operative ropivacaine 0.2% infusion 14ml/hr.	
ACB continuous vs FNB continuous					
Davidson et al. 2016[111]<br>USA<br>2013-2014<br>2 studies combined from 1 centre	Primary, unilateral TKR or unicompartmental<br>54 (39 TKR, 16 UKR); 56 (41 TKR, 15 UKR)<br>TKR mean 67 (SD 8); 66 (7). UKR 70 (10); 68 (12)	Spinal or general anaesthesia. Intra-operative i.v. fentanyl, hydromorphone, and/or morphine.<br>LIA with 30 ml ropivacaine (0.5%), ketorolac (30 mg), and epinephrine (5 µg/ml).<br>Post-operative: oral acetaminophen (975 mg every 6 hr), celecoxib (200 mg every 12 hr), and sustained release oxycodone (10 mg every 12 hr). For breakthrough pain, infusion pump bolus (4 ml, 30-min lock-out). Rescue opioid titrated to pain severity. 10 ml lidocaine (2%) bolus was given via the perineural catheter for moderate or severe pain.			12 months<br>31; 29<br>High risk of bias due to partial follow up<br>TKR and UKR combined. Pain score (0-10) at 12 months median 0.0 (IQR 0.0, 3.0); 0.5 (0.0, 2.0). P=0.80). Pain score >0: 35%; 32%. P=0.65. No difference at 4 months when follow up more complete (51;

For peer review only - http://bmjopen.bmj.com/site/about/guidelines.xhtml

	TKR 59%; 66%.<br>UKR 47%; 47%	Ultrasound guided ACB. Ropivacaine 0.2% at basal rate of 6 ml/hr, a 4-ml bolus, and a lockout of 30 minutes	Ultrasound guided continuous FNB. Ropivacaine 0.2% at basal rate of 6 ml/hr, a 4-ml bolus, and a lockout of 30 minutes	52) in pain score (p=0.80) or pain score >0 (p=0.48).<br>Falls in hospital: 2; 5
ACB single vs FNB single				
Macrinici et al. 2017[38]<br>USA<br>Before 2017<br>1 centre	Primary unilateral TKR, indication not specified (selected by the surgeon for TKA)<br>49; 49<br>Mean 67 (SD 8); 67 (8)<br>61%; 63%	Multimodal regimen including NSAIDs, non-opioid analgesics, opioids. LIA 40ml Marcaine 0.25%.<br>All patients received an ultrasound guided needle insertion into ACB and FNB sites.		6 months<br>3; 4 lost to follow up. 6; 3 complications<br>Low risk of bias<br>VAS pain similar between groups at 6 months. No difference in functional outcomes<br>Medical complications: 3; 0. Surgical complication: 0; 1. Temporary foot drop: 3; 2.
		Immediately after surgery, 30ml solution with 100ml Marcaine into ACB site. 30 ml saline into FNB site	Immediately after surgery, 30ml solution with 100ml Marcaine into FNB site. 30 ml saline into ACB site	
FNB continuous vs oral opioid				
Nader et al. 2012[39]<br>USA<br>2007-2008<br>1 surgeon	Elective unilateral TKR<br>31; 31<br>Median (IQR) 65 (60, 76); 64 (60, 71)<br>58%; 77%	Before surgery, patients received 1–2mg midazolam as needed. Epidural with 10mg 0.5% isobaric bupivacaine injected intrathecally. Intraoperative sedation with propofol infusion of 25-75mcg/kg/minute. In post-anesthesia recovery area, PCA epidural with basal infusion of 3 ml/hr (1 mg/ml bupivacaine and 10 mg/ml hydromorphone) with patient-activated boluses of 3 ml with a lockout interval of 15 minutes and per hour maximum of 15 ml. Infusion discontinued and epidural catheter removed on morning of POD 1. All subjects received 5 mg warfarin on evening of surgery and 40 mg enoxaparin starting on POD 1		6 and 12 months<br>1; 1 lost to follow up at 12 months<br>Low risk of bias<br>No difference in overall median NRS pain score at 6 months and 12 months: 0 (IQR 0, 1); 0 (0, 1). p=1.0. At 12 months, some evidence favouring hydrocodone for pain ascending/ descending stairs: 1 (0, 2); 0 (0, 0). p=0.01. Also, suggestion of reduced pain in hydrocodone group at night in bed (p=0.06) and sitting/ lying (p=0.07), standing upright (p=0.10). No difference walking on flat surface (p=0.41).<br>Falls in month after surgery: 1;0. Positive joint aspirate: 3; 0. VTE: 0; 4.
		Continuous FNB inserted with use of stimulator. After discontinuation of epidural anaesthesia on the morning of POD1 10mL bolus of ropivacaine 0.25% followed by 5ml/h infusion of 0.1% ropivacaine. On morning of POD 2, ropivacaine infusion	10 mg oral hydrocodone plus 325mg acetaminophen every 4-6 hours administered for pain as needed. Sustained release oxycodone 10mg for 12 hours with oral hydromorphone 2 mg over	

For peer review only

For peer review only - http://bmjopen.bmj.com/site/about/guidelines.xhtml

		discontinued. Femoral catheter removed 24 hours after previous dose of enoxaparin.	4 hours for breakthrough pain	
FNB continuous vs PCA				
Wang et al. 2015[112]<br>China<br>2012-2013<br>3 centres	Elective unilateral TKR<br>82; 86<br>No significant differences in age or sex	General anaesthesia with midazolam (0.02-0.04mg/kg), fentanyl (1µg/kg), propofol (1-2mg/kg) and cisatracurium (0.15mg/kg). Anaesthesia maintained with sevoflurane during surgery. Intramuscular injection with 10mg metoclopramide and 2.5mg droperidol 30 minutes before surgery. Post-surgery, celocoxib and parecoxib 40mg for patients with severe pain, and i.v. morphine if needed.		6 and 12 months<br>2; 4 lost to follow up at 12 months<br>Unclear risk of bias: limited reporting of randomisation methods.<br>No differences were observed between groups at 6 or 12 months for any HSS domain including pain.<br>No nerve injuries
		Continuous FNB with ultrasound stimulator. After surgery, 0.2% ropivacaine (20ml) injected through catheter. Then an analgesia pump was attached delivering 0.2% ropivacaine 8ml/hr.	Epidural PCA 0.2% ropivacaine was injected at a rate of 5 ml/hr in a 2ml pulse dose	
Peng et al. 2014[40]<br>China<br>Before 2014<br>1 centre (2 surgical teams with 4 surgeons and 2 anaesthesiologists)	Primary unilateral TKR<br>140;140<br>Mean: 66.8 (SD 9.4); 68.0 (SD 11.2)<br>73%; 65%	General intravenous and inhalational anaesthesia: midazolam 0.1-0.15mg/kg (etomidate 0.15-0.2mg/kg for patients >65 years), propofol 2.0-2.5mg/kg, sufentanil citrate 0.3-1.0µg/kg, and vecuronium 0.08-0.12mg/kg for induction of anaesthesia. Maintenance with inhalation of 1%-3% sevoflurane and continuous intravenous infusion of remifentanil 7-8µg/kg/hr and propofol 25-75µg/kg/min. After wound closure, 5-10µg intravenous sufentanil and loading dose of PCA injected. i.v. injection of 4mg ondansetron.		6 and 12 months<br>31; 38 at 12 months<br>Low risk of bias<br>Chronic post-operative pain (NRS 1+) in 38.5% of PCA group at 6 months compared with 25.7% in FNB group (p=0.021). No difference at 12 months (p=0.273).<br>Authors only reported short term adverse events associated with use of PCA.
		FNB with ultrasound guidance. Initial dose of 10ml 2% lidocaine and 10ml 1% ropivacaine. 30 minutes before end of operation, catheter connected to PCA pump; patients received loading dose of 5ml of 0.15% ropivacaine followed by infusion of 0.15% ropivacaine at 5ml/hr, with bolus of 5mL	i.v. PCA with tramadol 800mg, flurbiprofen axetil 100mg, and dexamethasone 5mg with saline to a volume of 80ml. Loading dose of 2ml followed by an infusion rate of 1 ml/hr with bolus of 2 ml. Lock time 15min.	

For peer review only - http://bmjopen.bmj.com/site/about/guidelines.xhtml

		and lock time of 30 min. Preoperatively, a loading dose of 30ml was injected for intraoperative analgesia.		
Wu and Wong 2014[44]<br>China<br>2009-2011<br>1 centre	Unilateral elective TKR, 98% for osteoarthritis<br>40; 39 (30; 30 after post randomisation exclusions)<br>Mean 68.8 (SD 6.4); 68.9 (7.5)<br>73%; 73%	Paracetamol, sustained release diclofenate, opioids (codeine or morphine). Spinal anaesthesia		6 months<br>2; 2 not pre- and peri-operative exclusions<br>Low risk of bias<br>No separate pain outcome but improvement of KSS from pre-operative was FNB 48.73 and PCA 44.7 (p=0.513)<br>Including patients not followed up. Deaths: 0; 0. Infection: 1;1. DVT: 2; 3. Shock: 3;2. Transfusion: 2;3. Also from excluded cases. Atrial fibrillation and confusion: 0; 1. PE: 0; 1. Sepsis: 1;0. ICU admission for shock: 1; 0.
		Catheter inserted under nerve stimulation and ultrasound guidance. Standardised bolus of 15 mL 0.5% levobupivacaine. Continuous infusion of 8 to 12 mL/h 0.08% levobupivacaine postoperatively until POD 3	Intravenous PCA morphine after the operation	
FNB and SNB continuous vs epidural PCA				
Anastase et al. 2014[113]<br>Germany<br>2010-2011<br>1 centre	Primary unilateral TKR<br>55; 50<br>Mean 68.2 (SD 9.2); 69.7 (SD 8.7)<br>65%; 69%	Premedication with 10 mg oral clorazepate. Spinal anaesthesia with light sedation: 12.5mg 0.5% bupivacaine. Supplemental postoperative analgesia available with i.v. piritramid		6 and 12 months<br>15; 14<br>High risk of bias due to large loss to follow up<br>Pain during previous 4 weeks: 1 no pain, 2 very little, 3 little, 4 moderate, 5 loud, 6 very loud (translation from German). No difference at 6 months p=0.37. At 12 months, FNB/SNB median 2.00 (1.00, 2.00), PCA 2.00 (2.00, 2.00) p=0.004 favouring FNB/SNB.<br>No falls associated with quadriceps weakness. 6 and 12 month adverse events not reported.
		After spinal anaesthesia installed, SNB and FNB catheters inserted with ultrasound guidance. 5 ml bolus 0.2% ropivacaine. FNB with an hourly rate of 5 ml, bolus administration of 5 ml by the patient and the lock-out interval of 20 mins. SNB 5 ml/h to a maximum of 8 ml/h, 5 ml bolus administered by the patient	Epidural catheter installed at the same time as spinal catheter. 5ml 0.2% ropivacaine and PCA performed through the epidural catheter. Hourly rate 3 ml, bolus administration of 5 ml, and lock-out period of 30 minutes.	

For peer review only - http://bmjopen.bmj.com/site/about/guidelines.xhtml

		and lock-out interval of 20 minutes.		
**FNB single vs LIA**				
Fan et al. 2016[36]<br>China<br>2012-2014<br>Single hospital, 2 surgeons	Primary unilateral TKR (75% osteoarthritis; 25% rheumatoid arthritis)<br>80; 80 (78; 79 in analysis)<br>Mean 68.4 (SD 8.8); 67.6 (6.3)<br>79%; 86%	General anaesthesia in all but 1 in each group. After surgery, i.v. morphine, PCA and parecoxib 40mg		1 year<br>3 protocol violations<br>Low risk of bias<br>No separate pain outcome. Mean KSS at 1 year similar between groups: 94.2 (SD 2.6); LIA 93.9 (3.1). p=0.51<br>Infection: 0; 0. DVT: 1; 1. Femoral nerve injury: 1; 0.
		FNB performed pre-operatively with 20ml ropivacaine 0.5%.<br>After cementing prostheses, 50ml of saline injected into periarticular soft tissue.	Placebo equivalent of FNB with saline<br>After cementing prostheses, 50ml of LIA mixture containing morphine (1ml: 10mg), ropivacaine (10ml: 100mg), and diprospan (1ml: 5mg betamethasone dipropionate and 2mg betamethasone sodium phosphate) injected into periarticular soft tissue.	
**FNB single and epidural vs LIA**				
Reinhardt et al. 2014[41]<br>USA<br>2010-2012<br>Single hospital, 2 surgeons	Elective unilateral TKR for osteoarthritis<br>51; 51 (49; 45 received allocated intervention)<br>Mean 67.9 (SD 10.9); 66.6 (10.1)<br>59.2%; 57.8%	Spinal anaesthetic (2.5ml 0.5% bupivacaine). Mobic 15mg daily. Oral Perocet or Vicodin as required. Subcutaneous Dilaudid for severe breakthrough pain. Intravenous Toradol.		1 year<br>0: 0 of patients who received allocated intervention<br>Low risk of bias<br>VAS pain at 1 year similar between groups (noted in text and shown graphically)<br>No wound-related complications or infections. 1 DVT and 1 DVT plus PE in epidural group. Arthrofibrosis: 2; 1
		Combined spinal-epidural (500ml hydromorphone 10µg/ml and bupivacaine HCl 0.06%).<br>Single intra-operative FNB injection (30ml 0.25% bupivacaine).<br>Continuous 48-hour epidural infusion (4ml/hr with 4ml per demand dose, locked out every 10 minutes with an hourly limit of 20ml). Epidural infusion weaned to 2ml/hr on POD1 and to 0 ml/hr at 5 p.m. on POD1. Demand dose with	Intra-articular knee catheter placed intraoperatively with continuous 0.2% ropivacaine infusion at 7 ml/hr until POD2.<br>Placebo epidural catheter, no FNB, and postoperative placebo continuous epidural infusion of saline.	

For peer review only

For peer review only - http://bmjopen.bmj.com/site/about/guidelines.xhtml

		lockout parameters continued for 48 hours.<br>Placebo intraarticular knee catheter placed intra-operatively with continuous saline 7ml/hr infusion until POD2.		
LIA with corticosteroid vs LIA with no corticosteroid				
Seah et al. 2011[48]<br>Singapore<br>2004-2005<br>1 hospital	TKR<br>50; 50<br>Mean 65.4; 67.9<br>Sex not stated	General or spinal anaesthesia. Postoperative oral naproxen and PCA (with morphine bolus of 1mg, lock-out time 5 minutes, and maximum dose 8 mg/hr) for 48 hours.		6 months and 2 years<br>No losses to follow up reported<br>Low risk of bias<br>No separate pain outcome but no statistically significant difference in OKS between groups at 2 years<br>Deep infection: 1; 1
		Intraoperative periarticular injection of 0.5ml/kg 1:200,000 epinephrine and 0.5% bupivacaine diluted with 30ml of normal saline. 40mg of corticosteroid (triamcinolone acetonide) was added to half the mixture. The solution with the corticosteroid was injected into the deep tissues. The remaining solution was injected into the skin incision before closure.	Intraoperative periarticular injection of 0.5ml/kg 1:200,000 epinephrine and 0.5% bupivacaine diluted with 30ml of normal saline. Half the mixture was injected into the deep tissues. The remaining solution was injected into the skin incision before closure.	
Yue et al. 2013[114]<br>China<br>2011-2012<br>1 hospital	Unilateral TKR for osteoarthritis<br>36; 36<br>Mean 70.2 (SD 6.4); 69.3 (5.7)<br>89%; 89%	General anaesthesia. PCA (25 mg/100ml morphine: a 1mg bolus, 6 minutes lock-out, and 5mg/hr maximum) for 72 hours after surgery. 5-10mg intramuscular morphine as rescue. Celecoxib pre- and post-operatively		6 and 12 months<br>No loss to follow up reported<br>Unclear risk of bias.<br>No separate pain outcome. No difference in mean KSS between groups at 6 or 12 months<br>No incision infection or tendon rupture complications
		Injections with local anaesthetic agent and adrenaline (0.75% ropivacaine 30ml, 1:1000 adrenaline 0.5ml, and isotonic sodium chloride solution 70ml) plus corticosteroid (1ml betamethasone).	Injections with local anaesthetic agent and adrenaline (0.75% ropivacaine 30ml, 1:1000 adrenaline 0.5ml, and isotonic sodium chloride solution 70ml) with no added corticosteroid.	

For peer review only

For peer review only - http://bmjopen.bmj.com/site/about/guidelines.xhtml

		Another 50ml syringes fluid without corticosteroid was infiltrated into the skin	Another 50ml syringes fluid without corticosteroid was infiltrated into the skin		
LIA including ketorolac vs epidural					
Spreng et al. 2012[115], Spreng et al. 2010[116]<br>Norway<br>2007–2009<br>1 hospital	Unilateral, non-cemented TKR with no patella resurfacing<br>34; 34; 34<br>66.5 (SD 11.); 67.2 (SD 8.9); 65.8 (SD 10.1)<br>61%;61%;67%	Premedication with oral paracetamol (1-2g). Spinal anaesthesia with 13-15mg bupivacaine 5mg/ml with 20µg fentanyl. If indicated, up to 10ml/hr 10mg/ml propofol for sedation. Acetaminophen 1g every 6 hours. i.v. PCA morphine for 48 hours after surgery (2mg bolus with 10 minutes lockout time). When PCA stopped, 10mg slow release oxycodone twice daily. 5mg oxycodone as rescue analgesia.			12 months<br>13 did not provide complete data<br>Unclear risk of bias due to limited reporting (long-term outcome only reported as conference abstract).<br>Perioperative analgesic treatment did not have any significant influence on any KOOS outcomes.<br>Infection: 0; 0; 1. No long-term adverse events reported
		i.v. injection of ketorolac 1ml (30mg/ml) and morphine 5ml (1mg/ml).<br>Infiltration with ropivacaine 150mg, epinephrine 0.5mg, ketorolac 30mg and morphine 5mg in 150ml saline.<br>After closure, catheter placed into knee joint and 10ml infiltrate injected. 22-24 hours after surgery, 20ml injection through catheter of ropivacaine 19ml (7.5mg/ml) and ketorolac 1ml (30mg/ml). i.v. injection of	i.v. injection of 6ml saline.<br>Infiltration with ropivacaine 150mg, epinephrine 0.5mg, ketorolac 30mg and morphine 5mg in 150ml saline.<br>After closure, catheter placed into knee joint and 10 ml infiltrate injected. 22-24 hours after surgery, 20ml injection through catheter of ropivacaine 19ml (7.5mg/ml) and saline 1ml. i.v. injection of saline 1ml.<br>Sham epidural catheter.	Epidural catheter inserted immediately before spinal anaesthesia. When spinal anaesthesia started to wear off, epidural infusion for 48 hrs with 6-10 ml/hr fentanyl 2µg/ml, epinephrine 1µg/ml, bupivacaine 1mg/ml.<br>No knee infiltrations.<br>Sham knee catheter with no injections	

For peer review only - http://bmjopen.bmj.com/site/about/guidelines.xhtml

		ketorolac 1ml (30mg/ml).<br>Sham epidural catheter.			
**Spinal with added high dose morphine sulphate vs spinal with added low dose morphine sulphate vs spinal with no morphine sulphate**					
Foadi et al. 2017[117]<br>Germany<br>Before 2017<br>1 centre	Unilateral TKR or THR for osteoarthritis<br>16; 16; 17<br>Mean 67.63 (SE 2.45); 67.33 (2.87); 63.71 (3.14) 56%; 44%; 65%	3ml spinal anaesthesia with 0.5% bupivacaine<br>Post-operative 1 g metamizole (orally or intravenously) every 4 hours. 5 mg morphine (intravenous or subcutaneous) as rescue medication			6 months<br>"only a few dropouts". >70% questionnaire return rate.<br>Unclear risk of bias due to limited reporting of pilot RCT.<br>No difference in WOMAC pain between groups at 6 months.<br>No adverse events noted
		0.2mg morphine sulphate added to spinal anaesthesia	0.1mg morphine sulphate added to spinal anaesthesia	No morphine sulphate added to spinal anaesthesia	

2. Myofascial trigger point dry needling

**Author**<br>**Country**<br>**Recruitment dates**<br>**Setting**	**Indication**<br>**Number randomised intervention; control**<br>**Age**<br>**% female**	**Common pain management**		**Follow up**<br>**Losses to follow up intervention; control**<br>**Risk of bias issues**<br>**Key results**
		**Group 1 (intervention)**	**Group C (control)**	
Mayoral et al. 2013[118]<br>Spain<br>2007-2008<br>Single centre	Unilateral TKR for osteoarthritis<br>20; 20<br>Mean 71.7 (SD 6.1); 72.9 (7.9)<br>72.5%	General or spinal anaesthesia		6 months<br>4; 5<br>High risk of bias due to large losses to follow up<br>WOMAC pain at 6 months: mean 3.24 (SD 3.03); 3.13 (2.72). Difference not statistically significant. No difference between groups in VAS pain (p=0.725) or proportion of patients reporting significant VAS pain at 6 months.
		After anaesthesia and surgery started, dry needling applied 20 times to all myofascial trigger points by a trained and experienced physical therapist.	If spinal anaesthesia used, dry needling simulated behind screen	

For peer review only - http://bmjopen.bmj.com/site/about/guidelines.xhtml

				No complications related to the dry needling intervention. Other adverse events not collected.

3. Tourniquet

Author<br>Country<br>Recruitment dates<br>Setting	Indication<br>Number randomised intervention; control<br>Age<br>% female	Common blood conservation strategies		Follow up<br>Losses to follow up intervention; control<br>Risk of bias issues<br>Key results
		Group 1 (intervention)	Group C (control)	
Ejaz et al. 2014[54]<br>Denmark<br>2011-2012<br>1 centre	Primary unilateral TKR for osteoarthritis<br>35; 35 (33; 31 received intervention)<br>Mean 68 (SD 8.0); 68 (7.8)<br>45.5%; 45.2%	Before surgery, oral tranexamic acid (1g). Tranexamic acid (0.5g) 3 hours after surgery and 6 and 12 hours postoperatively.		6 and 12 months<br>0; 0 of those who received intervention<br>Low risk of bias<br>Statistically significant difference in KOOS pain intensity at 2 months favouring TKR without a tourniquet (p < 0.001). Small difference between groups not statistically significant at 6 and 12 months.<br>Small number of adverse events did not suggest extra risk in the group with no tourniquet.
		Appropriately sized thigh tourniquet applied. Limb exsanguination by elevation for 2 minutes and cuff inflated to 250mm Hg.	Appropriately sized thigh tourniquet applied but not inflated. Served as safety device in case of uncontrollable bleeding.	
Liu et al. 2014[56]<br>Australia<br>Before 2014<br>1 surgeon	Unilateral TKR for osteoarthritis<br>10; 10<br>Mean 67.0; 70.0<br>30%; 10%	PCA. No CPM		6 and 12 months<br>0; 0<br>Low risk of bias<br>No separate pain measure. Total OKS not significantly different at 6 and 12 months<br>Blood transfusions: 3; 0.
		Tourniquet inflated to 300 mmHg before skin incision. Tourniquet deflated after wound closure and dressing.	Tourniquet placed but not inflated	
Mittal et al. 2012[57]<br>Australia<br>2008-2010	Primary unilateral TKR<br>31; 34	Autologous blood re-infused if required		1 year<br>5; 2
		Short duration. Tourniquet set at 300mm Hg inflated	Long-duration. Tourniquet set at 300mm Hg inflated before	

For peer review only

For peer review only - http://bmjopen.bmj.com/site/about/guidelines.xhtml

<table>
<tr><td>1 centre</td><td>Mean 67.5 (SD 8.9); 66.6 (8.4)
81%:74%</td><td colspan="2">prior to cement application and deflated when cement hardened</td><td colspan="2">skin incision and deflated when cement hardened</td><td>Low risk of bias. However, RCT terminated early due to increased need for blood transfusion in short duration tourniquet group.
No separate pain outcome. Total OKS (0-48) at 52 weeks higher in long-duration group reflecting better recovery than short duration group but not significantly (p=0.12). Mean difference approximately 5 which is greater than MCID of 4[78].
Transfusions: 10; 2. Patient reported adverse event: 26; 12</td></tr>
<tr><td rowspan="2">Abdel-Salam and Eyres 1995[119]
UK
Date not stated
1 surgeon</td><td rowspan="2">Primary unilateral TKR of which 91% osteoarthritis
40; 40
Mean 72 (range 65-80); 74 (64-82)
57.5%; 62.5%</td><td colspan="4">Tourniquet placed around thigh</td><td rowspan="2">1 and 2 years
0; 0
Unclear risk of bias due to limited reporting of methods. No pain measure or PROM
Surgeon recorded HSS score at 1 year 90 (78-97); 91 (80-97). Not significantly different at 1 or 2 years.
Blood loss similar between groups. Wound infections: 5;0. DVT: 4;0</td></tr>
<tr><td colspan="2">Limb exsanguinated for 2 minutes and tourniquet inflated to twice systolic blood pressure</td><td colspan="2">Tourniquet not inflated</td></tr>
<tr><td rowspan="2">Şükür et al.2016[120]
Turkey
2015
1 surgeon</td><td rowspan="2">Primary unilateral TKR, in women with osteoarthritis
30; 30; 30; 30
Mean 67.0 (SD 7.0); 66.9 (8.5); 68.4 (6.9); 68.4 (6.8)
100%</td><td colspan="4">Pneumatic tourniquet inflated to 125mm Hg above systolic blood pressure</td><td rowspan="2">6 months
0;0;0;0
High risk of bias. KSS outcome noted in methods but not presented in results.
KSS results not reported at 6 months but no significant differences between groups at 3 months.
Surgical and wound complications similar between groups. No infections, fractures or instability requiring revision within 6 months</td></tr>
<tr><td>Knee in 90° flexion and tourniquet deflated during wound closure</td><td>Knee in 90° flexion and tourniquet inflated during wound closure</td><td>Knee in full extension and tourniquet deflated during wound closure</td><td>Knee in full extension and tourniquet inflated during wound closure</td></tr>
<tr><td></td><td></td><td colspan="4">Blood transfusion if required</td><td>3-22 months, mean 12;13 months</td></tr>
</table>

For peer review only - http://bmjopen.bmj.com/site/about/guidelines.xhtml

<table>
<tr><td>Zhang et al.2016 [121]
China
2014-2015
1 hospital</td><td>Primary TKR for osteoarthritis
84; 82
Not reported
Not reported</td><td colspan="3">Tourniquet</td><td colspan="3">No tourniquet</td><td>Not clear
High risk of bias. Variable follow up. HSS outcome noted in methods but not presented in results.
HSS not reported.
Transfusion rates similar between groups. At mean follow up of 12 -13 months, patients operated on without a tourniquet had a lower rate of DVT (2.4%) compared with those with a tourniquet (10.7%).</td></tr>
<tr><td rowspan="2">Zhang et al. 2017[58]
China
2008-2011
1 surgeon</td><td rowspan="2">Primary unilateral cemented TKR for osteoarthritis
50; 50; 50
Mean 70.3 (SD 6.6); 71 (10.2); 68.2 (6.8)
54%; 60%; 50%</td><td colspan="6">Tourniquet inflated to 300-337mm Hg. Tranexamic acid not generally used</td><td rowspan="2">6 months
0; 0; 0
Low risk of bias
No separate pain outcome. HSS similar between groups at 6 months (p=0.839).
At 2 weeks DVT: 0; 0; 1. Intramuscular vein thrombosis: 4; 3; 3. Transfusions: 30%; 26%; 10%</td></tr>
<tr><td colspan="2">Tourniquet for entire operation</td><td colspan="2">Tourniquet removed before wound closure</td><td colspan="2">Tourniquet from first bone osteotomy until wound closure</td></tr>
<tr><td rowspan="2">Huang et al. 2017[55]
China
2015
1 centre</td><td rowspan="2">Primary unilateral TKR for osteoarthritis
50; 50
Mean 66.2 (SD 8.3); 65.1 (6.8)
64%; 68%</td><td colspan="6">Tranexamic acid</td><td rowspan="2">6 months
0; 0
Low risk of bias
VAS pain similar between groups at 6 months (p=0.728). Mean HSS score 90.3 (SD 3.2); 91.2 (2.5). P=0.151
DVT: 0; 0. PE: 0; 0. Intramuscular venous thrombosis: 6; 4. Superficial infection: 1; 0. Wound secretion: 6; 0. No significant difference in blood loss between groups.</td></tr>
<tr><td colspan="3">Tourniquet</td><td colspan="3">No tourniquet</td></tr>
</table>

4. Compression bandage

**Author**	**Indication**	**Common treatments**	**Follow up**

For peer review only - http://bmjopen.bmj.com/site/about/guidelines.xhtml

Country<br>Recruitment dates<br>Setting	Number randomised intervention; control<br>Age<br>% female	Intervention	Control	Losses to follow up intervention; control<br>Risk of bias issues<br>Key results
Brock et al. 2017[122]<br>UK<br>2013-2014<br>1 hospital	Primary unilateral TKR for osteoarthritis<br>25; 25 (24 received intervention)<br>Mean 67.3 (SD 8.2); 69.5 (6.8)<br>66.7%; 64.0%	Hydrocolloid dressing left in place until clips removed on day 10-14<br>Soft inner layer with compressive outer layer bandage. Removed after 24 hours.	Standard bandaging with soft inner layer and crepe bandage outer layer. Removed after 24 hours and cryocuff used.	6 months<br>0; 0 of patients receiving intervention<br>Low risk of bias<br>No separate pain outcome. Mean OKS similar between groups at 6 months: 35.8 (SD 7.7); 34.3 (10.6). P=0.58<br>No infections or thromboembolic events in either group

5. Blood conservation

Author<br>Country<br>Recruitment dates<br>Setting	Indication<br>Number randomised intervention; control<br>Age<br>% female	Common blood conservation strategies		Follow up<br>Losses to follow up intervention; control<br>Risk of bias issues<br>Key results
		Intervention	Control	
Tranexamic acid				
Sa-Ngasoongsong et al. 2011[64]<br>Thailand<br>2008-2009<br>1 hospital	Primary knee osteoarthritis with unilateral primary cemented computer assisted TKR<br>24; 24<br>69.0 (SD 8.2); 69.2 (7.6)<br>91.7%; 75%	Drain and compressive dressing<br>25ml saline solution containing 250mg tranexamic acid injected into knee joint after fascial closure	25ml saline solution injected into knee joint after fascial closure	6 months<br>0; 0<br>Low risk of bias<br>No separate pain score reported but WOMAC overall score mean 18.6 (SD 7.6); 20.8 (6.4). P=0.282<br>Lower peri-operative blood loss in tranexamic acid group and need for blood transfusion, 1/24 compared with 8/24 in control group. No DVT,

For peer review only - http://bmjopen.bmj.com/site/about/guidelines.xhtml

					wound complications or infection reported in either group
Kim et al. 2014[61]<br>Korea<br>2009-2011<br>1 hospital	Primary unilateral TKR for osteoarthritis<br>90; 90<br>Mean 73.5 (SD 5.5); 71.9 (SD 5.9)<br>88%; 87%	Tourniquet, drain, compressive dressing. Allogenic blood transfusion and intravenous iron and erythropoietin if required			1 year<br>0; 0<br>Low risk of bias<br>WOMAC pain mean 3.2 (2.6); 2.8 (2.3). Difference not statistically significant<br>Lower blood loss and need for allogenic transfusion in tranexamic acid group. No DVT. 1 PE in control group.
		10 mg/kg body weight tranexamic acid in 100 mL of normal saline given as slow intravenous injection 30 min before tourniquet deflation, and the same amount 3 hours later.	No tranexamic acid and no placebo		
Sa-Ngasoongsong et al. 2013[65]<br>Thailand<br>2010-2011<br>1 hospital	Primary unilateral cemented TKR for osteoarthritis<br>45; 45; 45<br>Mean 68.1 (SD 6.2); 67.6 (8.7); 66.2 (7.3)<br>88.9%; 93.3%; 95.6%	Drain and compressive dressing			1 year<br>0; 0; 0<br>Low risk of bias<br>No separate pain outcome but WOMAC mean 14.5 (7.1); 15.1 (6.2); 15.5 (6.6). P=0.42<br>Total blood and Hb loss lower in intervention groups than control. Fewer transfusions in 500mg (0) than 250mg tranexamic acid group (6) and control group (10). 2 DVT in 500mg group. 1 DVT in 250mg group. 1 PE and 3 DVT in control group. No infections.
		25ml saline solution containing 500mg tranexamic acid injected into knee joint after fascial closure via drain tube.	25ml saline solution containing 250mg tranexamic acid injected into knee joint after fascial closure via drain tube.	25ml saline solution injected into knee joint after fascial closure	
Hourlier et al. 2015[60]<br>France<br>2009-2010<br>1 hospital	Primary unilateral TKR for osteoarthritis<br>52; 54<br>74 (SD 6); 72 (7)<br>62%; 63%	Tourniquet, electrocautery, routine haemostasis, superficial drain. No blood salvage system.			6 months<br>0; 0<br>Low risk of bias.<br>No separate pain score but KSS clinical score mean 90 (SD 6); 90 (13). P=0.90<br>No difference between groups in total blood loss. 1 MUA in single treatment
		10 mg/kg intra-operative tranexamic infusion. After 2 hours, continuous infusion of tranexamic acid 2 mg/kg/hr for 20 hours via electric syringe	single bolus of 30 mg/kg tranexamic acid as an intraoperative infusion. After 2 hours, placebo saline continuous infusion via electric syringe		

For peer review only - http://bmjopen.bmj.com/site/about/guidelines.xhtml

				group. No deep infections or revisions.
Huang et al. 2017[55]<br>China<br>2015<br>1 centre	Primary unilateral TKR for osteoarthritis<br>50; 50<br>Mean 66.2 (SD 8.3); 65.8 (6.3)<br>64%; 70%	Tourniquet inflated to 100mm Hg above SBP before incision and deflated after wound closure<br>Intravenous tranexamic acid 20mg/kg before incision and tranexamic acid 10mg/kg at 3, 6, 12 and 24 hours. 1g tranexamic acid in 50ml saline irrigated into wound during operation	No treatment with tranexamic acid	6 months<br>0; 0<br>Low risk of bias<br>VAS pain similar between groups at 6 months (p=0.728). Mean HSS score (0-100) better in tranexamic acid group than controls: 90.3 (SD 3.2); 88.9 (3.0). P<0.001. Mean difference 1.4 lower than MCID of 8.29[81]<br>Greater blood loss in control group than tranexamic group (p<0.001). DVT: 0; 0. PE: 0; 0. Intramuscular venous thrombosis: 6; 3. Superficial infection: 1; 3. Wound secretion: 6; 9.
Thrombin infusion				
Kusuma et al. 2013[62]<br>USA<br>Not stated<br>1 hospital	Primary unilateral TKR for osteoarthritis<br>40; 40<br>Mean 64.6 (SD 10.2); 64.5 (7.3)<br>82.5%; 67.5%	Tourniquet, drain, Esmarch bandage, electrocautery<br>20,000 IU thrombin infusion (1,000 IU/mL) through fascial defect	Closure and drain placement protocol without the thrombin infusion.	1 year (6 months and 2 years also reported)<br>0; 0<br>Low risk of bias<br>No separate pain outcome. KSS mean 95.5; 96.0. p=0.45<br>Lower drop in Hb in thrombin group. Blood transfusion in 4 intervention and 7 control patients. 1 control patient had haematoma. No hospital readmissions
Flexion vs extension				
Napier et al. 2014[63]<br>UK<br>2003-2004<br>1 hospital	Primary unilateral TKR of which 89% for osteoarthritis<br>90; 90	No drains or tranexamic acid<br>Flexion. Operated knee kept in passive flexion (120°) post-operatively for 6 hours using a jig. Wound redressed and placed in flexion over a	Extension. Operated knee kept in full passive extension	1 year<br>5; 1 (12 did not attend follow up)<br>Low risk of bias.<br>No separate pain outcome. OKS mean 20.5 (SD9.0); 22.1 (9.7). P=0.27

For peer review only - http://bmjopen.bmj.com/site/about/guidelines.xhtml

	Mean 70.4 (SD 9.9) 71.0 (7.6)<br>74%; 64%	single pillow until POD1 morning.		1 MI and 1 DVT in each group. 1 haematoma in flexion group. 1 deep infection and 1 extensor muscle weakness in extension group. More transfusions in extension group (p=0.002)
Auto-transfusion of washed blood				
Thomas et al. 2001[123]<br>UK<br>Not stated<br>1 hospital	Unilateral TKR<br>115; 116<br>Mean 69.3 (range 32-95); 70.0 (40-88)<br>62%; 53%	Allogenic transfusion if Hb fell below 9g/dl<br>Auto-transfusion of wound drainage if volume >125ml post-operative. Blood washed and re-suspended before re-infusion using a centrifugal cell washing machine	Wound drainage discarded	6 months<br>Losses to follow up not reported<br>Unclear risk of bias due to limited details of methods and follow up.<br>No separate pain outcome. No significant difference in EQ-5D between groups.<br>7% of auto-transfusion group required allogenic transfusion compared with 28% in control group. Fewer infections, readmissions and GP visits in auto-transfusion group. No significant differences in other serious adverse events or mortality between groups.

6. Platelet rich plasma

**Author**<br>**Country**<br>**Recruitment dates**<br>**Setting**	**Indication**<br>**Number randomised intervention; control**<br>**Age**<br>**% female**	**Common blood conservation strategies**		**Follow up**<br>**Losses to follow up intervention; control**<br>**Risk of bias issues**<br>**Key results**
		**Group 1 (intervention)**	**Group C (control)**	
Aggarwal et al. 2014[124]	Primary unilateral surgery or first surgery of staged	Tourniquet. No tranexamic acid or suction drain. Blood transfusion if necessary due to intraoperative blood loss or postoperative haemoglobin <8g/dl.		6 months<br>No losses to follow up reported

For peer review only - http://bmjopen.bmj.com/site/about/guidelines.xhtml

India<br>2010-2011<br>1 surgeon	bilateral TKR for osteoarthritis<br>7; 14<br>Mean 56.43 (SD 7.59); 53.79 (9.75)<br>Sex not stated	8 ml PRP, prepared from patient's blood. Calcium chloride for activation given in a separate syringe in 4:1 ratio. PRP and calcium chloride injected into the posterior recess, gutters and capsule, and repaired extensor mechanism and prepatellar fat.	No treatment	High risk of bias due to unexplained differences in numbers of patients in randomised groups.<br>No separate pain outcome. WOMAC total at 6 months PRP mean 7.14 (SE 0.69), controls 7.86 (1.23), p=0.173<br>PRP group had lower fall in haemoglobin and need for blood transfusion

7. Cryotherapy

**Author**<br>**Country**<br>**Recruitment dates**<br>**Setting**	**Indication**<br>**Number randomised intervention; control**<br>**Age**<br>**% female**	**Common treatment**		**Follow up**<br>**Losses to follow up intervention; control**<br>**Risk of bias issues**<br>**Key results**
		**Group 1 (intervention)**	**Group 1 (intervention)**	
Wang 2017[125]<br>China<br>2013-2015	Unilateral TKR for osteoarthritis<br>53; 53<br>Mean 65.23 (SD 5.41); 64.97(5.36)<br>62.3%; 58.5%	CPM for 2 weeks		6 months<br>0; 0<br>Unclear risk of bias due to limited reporting<br>No separate pain outcome. At 6 months 87% of cryotherapy patients had excellent or good knee function compared with 69% of controls (p=0.032).<br>No adverse events reported in either group during functional training
		Compression cold therapy for 48 hours	No compression cold therapy	

For peer review only - http://bmjopen.bmj.com/site/about/guidelines.xhtml

8. Denusomab

Author<br>Country<br>Recruitment dates<br>Setting	Indication<br>Number randomised intervention; control<br>Age<br>% female	Common treatment		Follow up<br>Losses to follow up intervention; control<br>Risk of bias issues<br>Key results
		Group 1 (intervention)	Group 1 (intervention)	
Ledin et al. 2017[66]<br>Sweden<br>2012-2014<br>2 centres	Elective cemented primary unilateral TKR for osteoarthritis<br>25; 25<br>Mean 66 (SD 6.3); 64 (5.5)<br>60%; 60%			12, 24 months<br>0; 2<br>Low risk of bias<br>No significant differences in KOOS pain or other KOOS domains between groups 12 12 or 24 months<br>No suspected unexpected adverse reactions in either group
		Injection of 60mg denusomab 1 day after surgery and after 6 months	Injection of placebo 1 day after surgery and after 6 months	

9. Continuous passive motion

Author<br>Country<br>Recruitment dates<br>Setting	Indication<br>Number randomised intervention; control<br>Age<br>% female	Common treatment			Follow up<br>Losses to follow up intervention; control<br>Risk of bias issues<br>Key results
		Group 1 (intervention)	Group 2 (intervention)	Group C (control)	
Leach et al. 2006[126]<br>UK<br>Before 2005<br>1 hospital	Unilateral cruciate retaining rotating platform TKR for osteoarthritis<br>85 overall<br>Mean 71.2 (range 53-84); 72.9 (52-89)	Physiotherapy protocol from POD1 including slider board exercises to improve ROM and quadriceps strengthening exercises.			6 and 12 months<br>25 patients lost to follow up<br>High risk of bias due to large loss to follow up and use of date of birth randomisation<br>No difference in mean VAS pain at 1 year, CPM 0.6; control 0.9. p=0.49
		CPM commenced on first postoperative day set at a range 0–30 and used for 1 hour twice per day. Each day,	No CPM		

For peer review only - http://bmjopen.bmj.com/site/about/guidelines.xhtml

	50%; 54%	range was increased by 10° with discharge at POD 5-7.			Adverse events not reported
Sahin et al. 2006[127]<br>Turkey<br>Before 2006<br>1 hospital	Primary unilateral TKR for osteoarthritis<br>15; 16<br>Mean 61 (SD 6.0); 61.6 (7.5)<br>86%; 86%	Standard physiotherapy			6 months<br>3 lost to follow up<br>Unclear risk of bias as patients were followed up by treating physician.<br>Mean difference in VAS pain 0.1/10 slightly favouring no CPM group (95% CI -0.8, 0.9; P=0.87)<br>Adverse events not known
		From POD 1, CPM 2.5 hours 2x/day. Initially 0-40° flexion and increased by 10° each day until POD 7	No CPM		
Pope et al. 1997[128]<br>Australia<br>1988-1999<br>1 hospital	Primary unilateral or bilateral TKR of which 86% for osteoarthritis<br>62 (70 knees). Authors excluded those not followed up so groups were 18; 20; 19<br>Mean 72.5 (95% CI 64.4, 74.98); 72.7 (70.4, 75.0); 69.4 (64.4, 74.98)<br>64.7%; 50%; 72.2%	Physiotherapy commenced on postoperative day 1			6 and 12 months<br>8 patients (12 knees) excluding 1 death<br>High risk of bias due to losses to follow up and limited reporting of methods<br>No separate pain outcome. However, "pain disability" contributed up to 50 points out of a total of 70-point functional score (70 best outcome). No difference between groups in functional score: CPM 0-40 median 56 (range 20, 70); CPM 0-70 52 (10, 70); no CPM 52 (25, 70). p=0.80<br>CPM groups had greater blood loss than controls, p=0.008). 1 manipulation under anaesthesia in no CPM group, 2 revisions due to patellar dislocation in the 0-40 CPM group, 1 PE death in the 0-70 CPM group.
		Patients had an initial CPM range of 0-40° increased by 10° twice, on day after surgery and day 2, so that 0-60° flexion achieved before removal of machine at 48 hours	Patients had an initial CPM range of 0-70° increased by 10° twice, on day after surgery and day 2, so that 0-90° flexion achieved before removal of machine at 48 hours	Knee placed in an extension splint in the recovery room	
Beaupré etal. 2001[129]	Primary unilateral TKR of which	Standardised exercise during hospital admission which included a slider board session.			6 months

For peer review only - http://bmjopen.bmj.com/site/about/guidelines.xhtml

For peer review only

Canada<br>1997-1998<br>1 hospital	92% for osteoarthritis<br>40; 40; 40<br>Mean 68 (SD 9); 68 (9); 69 (8)<br>52.5%; 50%; 30%	3 sessions (2 hours) with CPM machine per day from POD2. Range increased from starting range 0-30 degrees as tolerated.	Minimum of two 10-minute slider board therapy sessions per day in addition to one in the standardised exercise. Active knee flexion and extension in sitting and lying positions performed independently as tolerated.	No intervention further than standardised exercise.	6; 8; 6<br>Unclear risk of bias due to losses to follow up<br>Mean WOMAC pain at 6 months: 76 (15); 85 (15); 79 (16). No difference over time between groups, p=0.62.<br>Long-term adverse events. Need for MUA: 1; 1; 0. DVT: 0; 1; 0. Cellulitis: 0; 0; 1. Infection 0; 0; 1.
Kumar et al. 1996[130]<br>USA<br>Before 1996<br>1 hospital	Primary unilateral TKR for osteoarthritis<br>40 (46 knees); 33 (37)<br>Mean 69 (range 52-86); 68 (42-88)<br>58%; 67%	Standard physiotherapy<br>CPM from POD 0. Initially 10 hours/ day 0-90° until discharge	No CPM. Passive range of movement ("drop and dangle") to 90° 2x/ day initially for 20 minutes, later 30-45 minutes.		6 months<br>15; 13 lost to follow up<br>High risk of bias due to large losses to follow up<br>No separate pain outcome. KSS CPM 82.7; Drop and dangle 80.7. p=0.78<br>Haematoma 3;1. Closed manipulation 1;3. DVT 0;0. PE 0;1
Worland et al. 1998[131]<br>USA<br>1996<br>1 hospital	Unilateral or bilateral TKR for osteoarthritis.<br>91 patients (114 knees randomised). After post-randomisation exclusions: 37 (49 knees); 43 (54 knees)<br>Mean 70.2 (range 44-84)<br>66.25%	CPM and physiotherapy during hospital admission<br>At home after discharge, CPM machine 3 hours per day on replaced knee for l0 days.	Physical therapist home visit 1 hour three times per week for 2 weeks		6 months<br>11 patients (11 knees)<br>Unclear risk of bias due to post-operative exclusions not reported separately for groups and limited reporting of methods.<br>No separate pain outcome. At 6 months, mean HSS score CPM 95.3 (SD 2.8); physiotherapy 95.7 (3.0). P=0.49.<br>Adverse events not reported.

For peer review only

For peer review only - http://bmjopen.bmj.com/site/about/guidelines.xhtml

MacDonald et al. 2000[132]<br>Canada<br>Before 2000<br>1 hospital	Primary unilateral TKR for osteoarthritis<br>40; 40; 40<br>Age and sex not reported	Active ROM, passive ROM exercises, mobilised as tolerated using walker or crutches.			6 and 12 months<br>Not reported<br>Unclear risk of bias due to limited and selective reporting.<br>No separate pain outcome. No statistical differences between groups for KSS at 6 and 12 months.<br>Adverse events not reported
		CPM commenced POD 0. Initially 0-50 degrees. Provided for 18-24 hour/ day. Increased by 10 degrees/ hour as tolerated. Continued until POD 1	CPM commenced POD 0. Initially 70-110 degrees. Provided for 18-24 hr/ day. Not increased.<br>Continued until POD 1	No CPM	
Bennett et al. 2005[67]<br>Australia<br>1997-2000<br>1 hospital	Primary unilateral TKR for osteoarthritis<br>47; 48; 52<br>70.7; 71.4; 71.7<br>72.3%; 64.6%; 67.3%	Standard in hospital physiotherapy programme			12 months<br>1 patient excluded due to inability to achieve 90° flexion<br>Low risk of bias<br>No separate pain outcome. No significant difference in KSS between groups at 1 year.<br>No difference in wound healing between groups
		Standard CPM from 0° to 40° for 2x3 hours on POD 1 increased by 10° per day until POD 6. Extension splint applied overnight	Early flexion CPM commenced in recovery room from 90° to 50° knee flexion. Increased gradually to CPM 90° to 0° for 2x3 hours in day 4-6.	No CPM	
Ersözlü et al. 2009[68]<br>Turkey<br>2003-2004	Primary unilateral TKR for osteoarthritis<br>30; 30; 30<br>Mean 65 (range 54-73); 61 (49-80); 62 (52-78)<br>66%; 55%; 57%	Conventional physical therapy			2 years<br>1; 1; 2<br>Low risk of bias<br>No separate pain outcome. KSS scores 98; 95; 92. No significant difference between groups p=0.67.<br>Infection 0; 0; 1. Arrhythmia 0; 1; 0. No difference in complications between groups
		CPM set at 30-40° from POD1. Increased as tolerated to POD7. 1 hour CPM 3x/day.	CPM set at 60-70° from POD3. Increased by 10°/ day to POD7. 1 hour CPM 3x/day.	No CPM	

10. Electrical stimulation

Author	Indication	Common rehabilitation strategies	

For peer review only - http://bmjopen.bmj.com/site/about/guidelines.xhtml

For peer review only

Country<br>Recruitment dates<br>Setting	Number randomised intervention; control<br>Age<br>% female	Intervention	Intervention	Common rehabilitation strategies
Avramidis et al. 2011[69]<br>Greece<br>2005-2006<br>1 hospital	Elective primary unilateral TKR for osteoarthritis<br>38; 38<br>Mean 70.54 (SD 4.68); 70.66 (3.73)<br>80%; 82.9%	Standard physiotherapy for 6 weeks. No CPM		1 year<br>3 (intervention intolerance); 3<br>Low risk of bias<br>Improved SF-36 bodily pain at 1 year in intervention group compared with control, mean 92 (SD 10.57); 79.48 (12.72). P<0.001. Difference of 12.52 close to MCID of 16.86[82]. No difference in OKS or American KSS<br>Adverse events not reported
		Transcutaneous electric muscle stimulation of the vastus medialis muscle from POD2 2x/ day for 2 hours for 6 weeks.	No intervention	
Stevens-Lapsley et al. 2012[133]<br>USA<br>2006-2010<br>1 hospital	Primary unilateral TKR for osteoarthritis<br>35; 31<br>Mean 66.2 (SD 9.1); 64.8 (7.7)<br>57.1%; 51.6%	Standard inpatient rehabilitation, home and outpatient physical therapy		6 months and 1 year<br>5; 6<br>Unclear risk of bias due to baseline differences in WOMAC<br>No difference in resting pain (points) at 1 year intervention mean 0.6 (SD 1.4); control 0.4 (1.5). Also similar at 6 months. Mean WOMAC total score better at 1 year in intervention group compared with control, 5.7 (5.9); 10.0 (12.2) and at 6 months. However, probably explained by baseline differences. Authors state no differences for change in WOMAC.<br>DVT 1; 0. Unspecified complication 1; 0. Infection 0; 2. Revision 0; 1
		Neuromuscular electrical stimulation commenced on POD2 for 6 weeks 2x/ day.	No intervention	

For peer review only - http://bmjopen.bmj.com/site/about/guidelines.xhtml

Levine et al. 2013[134]<br>USA<br>Before 2013<br>1 surgeon	Elective unilateral TKR for osteoarthritis<br>35; 35<br>Mean 68.1; 65.1<br>76%; 62%	2 sessions of ROM exercise		6 months<br>5; 9<br>Unclear risk of bias due to large uneven losses to follow up<br>KSS pain favoured intervention at 6 months but not significantly 79.08 (SD 10.97); 75.5 (14.77); 95%CI for difference -3.78, 10.93. Similar for WOMAC total score, 95%CI for difference -3.19, 14.81.<br>Confusion 2; 0
		Neuromuscular electrical stimulation commenced 14 days pre-operatively until 1 day before surgery. Recommenced at POD1 for 60 days. After hospital discharge no direct contact with a physical therapist	Formal physical therapy programme with progressive resistive exercises and strengthening in hospital and after discharge supervised by physical therapist.	
Moretti et al. 2012[70]<br>Italy<br>2008-2010<br>1 hospital	Primary unilateral TKR for osteoarthritis<br>15; 15<br>Mean 70.0 (SD 10.6); 70.5 (8.1)<br>Not reported	Rehabilitation protocol including CPM		6 and 12 months<br>No losses to follow up<br>Low risk of bias<br>Mean VAS pain (10-point scale) lower at 12 months in intervention group compared with control, 0.5 (SD 1.3); 3.6 (3.9). p< 0.05. Mean difference of 2.1 (10-point scale) greater than MCID of 16.1 (100-point scale)[83]<br>Difference also at 6 months.<br>More swelling of the knee in intervention patients than controls, statistically significant at 1 and 2 months
		Pulsed electromagnetic fields (I-ONE therapy) from POD7, 4 hours/ day for 60 days	No intervention	
Adravanti et al. 2014[135]<br>Italy<br>1 hospital	Primary unilateral TKR for osteoarthritis<br>16; 17<br>Mean 66 (SD 13); 73 (5)<br>62.5%; 52.9%	Standard rehabilitation protocol: active and passive mobilisation		6 months<br>4; 3<br>High risk of bias: small study, proportionately high losses to follow up<br>At 6 months, mean VAS pain in intervention group lower than in controls (p<0.05). At 3 years, 1/14 intervention patients and 4/12 controls reported severe pain
		Pulsed electromagnetic fields (I-ONE therapy) by POD7 for 4 hours/ day for 60 days	No intervention	

For peer review only

For peer review only - http://bmjopen.bmj.com/site/about/guidelines.xhtml

				No difference between groups in swelling at 6 months.

11. Rehabilitation

Author<br>Country<br>Recruitment dates<br>Setting	Indication<br>Number randomised intervention; control<br>Age<br>% female	Common rehabilitation strategies		Follow up<br>Losses to follow up intervention; control<br>Risk of bias issues<br>Key results
		**Intervention**	**Control**	
Walking guidance and training				
Li et al. 2017[71]<br>China<br>2015-2016<br>1 hospital	Primary unilateral TKR for osteoarthritis<br>43; 43<br>Mean 76.33 (SD 5.28); 78.47 (5.50)<br>55.8%; 51.2%	Before TKR, general guidance on joint activities, quadriceps muscle strength, use of aids, diet guidance, correct walking methods and precautions.<br>Knee passive flexion and extension to 90° and quadriceps muscle strength training commenced on POD 1. POD 3-7, straight leg raising exercises. 2 weeks after replacement, increased joint activities and muscle strength training, centre of gravity transfer training, limb weight training, and walking training.		6 months<br>0; 0<br>Low risk of bias<br>Mean VAS pain at 6 months: 0.51 (SD 0.74); 2.83 (0.88) favouring walking intervention group, p<0.01. Difference of 2.42 (10 point scale) greater than the MCID of 16.1 (100-point scale)[83]. HSS scores at 6 months favoured intervention, p<0.01.<br>No infection, allergic reaction or immune reaction in either group. Intervention not associated with swelling, pain, prosthesis loosening, thrombosis, or delayed wound healing
		Standing, weight and balance exercises from POD 1. From POD 2, walking guidance and training.	No additional rehabilitation	
Aquatic therapy				
Liebs et al. 2012[72]<br>Germany<br>2003-2004<br>4 hospitals	Elective primary unilateral TKR for osteoarthritis<br>87;98	Continuous passive motion machines daily after removal of suction drains. Programme of daily physiotherapy: range of motion activities; exercises for improvement of muscle tension, venous return, balance, coordination and gait; and instruction in activities of daily living.		6, 12 and 24 months<br>13.8%; 19.4% excluding deaths and unexplained reasons<br>Low risk of bias

For peer review only - http://bmjopen.bmj.com/site/about/guidelines.xhtml

	Mean 68.5 (SD 8.6); 70.9 (7.5)<br>70.1%;73.5%	Aquatic therapy for 30 minutes 3 times a week up to postoperative week 5. Pool exercises aimed at training of proprioception, coordination and strengthening with aid of float cuffs, training kickboards and bar floats.			WOMAC pain at 12 months: early aquatic mean 13.2 (SD 15.0); late aquatic 17.4 (22.4) p=0.22. No difference at 6 and 24 months.<br>5 early aquatic therapy patients and 1 late aquatic therapy patient readmitted to hospital within 3 months. 2 early aquatic patients and 1 late aquatic patient readmission directly or indirectly related to the intervention.
		Aquatic therapy beginning on the 6th postoperative day with the wound covered with a waterproof adhesive dressing.	Aquatic therapy as pool exercise after the completion of wound healing on the 14th postoperative day		
Rahmann et al. 2009[136]<br>Australia<br>2003-2005<br>1 hospital with 2 surgeons	Unilateral primary TKR or THR for osteoarthritis (50% TKR)<br>18;19;17 (11 had been excluded post-randomisation due to complications in hospital<br>Mean 69.4 (SD 6.5); 69.0 (8.9); 70.4 (9.2)<br>44.4%; 63.2%; 70.6%	Standard ward-based physiotherapy until day 3. 1 ward physiotherapy treatment per day. Surgical wounds covered with an occlusive, waterproof dressing. 40 mins/ day.			6 months<br>4;2;0 for combined THR and TKR<br>Unclear risk of bias as TKR patients more likely to receive ward-based control intervention. THR and TKR analysed together<br>No difference in overall WOMAC outcome at 6 months in THR and TKR patients combined between aquatic at fast pace and ward-based (p=0.929) and aquatic at 2 paces (p=0.872).<br>No adverse events reported after intervention commenced.
		From day 4, 1 to 1 individual physiotherapy. Aquatic physiotherapy programme to maximize function and strength. 40 mins/ day. Fast pace metronome 80-88 bpm	From day 4, 1 to 1 individual physiotherapy. Water exercise programme with general exercises not targeted at specific functional retraining in the aquatic environment. Slow pace metronome 50-58 bpm	From day 4, 1 to 1 individual ward-based physiotherapy. 40 mins/ day	
Supported early discharge					
Mahomed et al. 2008[137]<br>Canada<br>2000-2002<br>2 centres	Primary unilateral TKR or THR for osteoarthritis (approximately 50% TKR)<br>119;115<br>68	Inpatient physiotherapy			12 months<br>No losses to follow up<br>Low risk of bias (analysis by actual treatment received showed similar results)<br>WOMAC pain at 12 months marginally favoured home-based
		Discharged home when able to independently transfer supine to sitting and sitting to standing, walk 30 metres and climb stairs if necessary. Physiotherapist home visit within 48 hours and	Transfer to independent rehabilitation centre for 14 day stay.		

For peer review only

For peer review only - http://bmjopen.bmj.com/site/about/guidelines.xhtml

	About 67% women	subsequent management along a multidisciplinary clinical pathway (4-16 visits). Then outpatient physiotherapy or self-directed programme.		rehabilitation mean 87 (SD 16); 83 SD (20), p=0.08 but this was not statistically significant. Mean difference of 4 less than MCID of 8-9[77]. Results did not differ between TKR and THR patients.<br>Similar rates of dislocation, DVT and readmissions between groups. 2% inpatient group developed infections compared with 0 in home group
Hill et al. 2000[138]<br>UK<br>1997-1998<br>1 centre	Unilateral, primary TKR, irrespective of diagnosis or concomitant disease<br>70 randomised, with 32;28 eligible for trial at day 5	Care pathway for medical, nursing and physiotherapy care from admission until day 5		1 year<br>No losses to follow up reported after commencement of intervention<br>Unclear risk of bias due to limited reporting of methods.<br>No pain outcome or patient reported outcome. Control group had better mean KSS scores, but this did not reach statistical significance at 1 year or earlier.<br>1;1 serious infection, other wound infections 1;6, painful joints 9;4, other minor complications similar between groups
		Outreach team domiciliary visit prior to admission with assessment of home environment. At days 5–7, patients assessed to ensure discharge safe. Outreach team visit on day of discharge with further visits as required. 1+ physiotherapist visit linked with nurses to monitor knee performance. Discharge when skin clips removed, wound healed and specialist orthopaedic assistance not required, usually day 10–12	Inpatient care until removal of skin clips and wound healing.	
Flexion or extension during knee closure				
Wang et al. 2014[74]<br>China<br>2009-2010<br>1 centre	Primary unilateral TKR for osteoarthritis<br>40; 40<br>Mean 68.34 (SD 7.09), 67.87 (6.47)<br>17.5%; 22.5%	No patellar replacement or lateral retinacular release		6 months<br>No losses to follow up<br>Low risk of bias<br>Mean VAS pain in flexion group 1.15 (SD 0.73); extension group 1.12 (0.68), p=0.64
		Articular capsule, soft tissue and skin enclosed in 90° flexion which was maintained for 1-2 min after wound closure.	Wound closure performed in full extension	

For peer review only

For peer review only - http://bmjopen.bmj.com/site/about/guidelines.xhtml

				No wound complications, patella fracture or infection requiring surgery in either group

12. Wound management

Author<br>Country<br>Recruitment dates<br>Setting	Indication<br>Number randomised intervention; control<br>Age<br>% female	Common wound management strategies		Follow up<br>Losses to follow up intervention; control<br>Risk of bias issues<br>Key results
		Intervention	Control	
Kong et al. 2014[75]<br>South Korea<br>2011<br>1 surgeon	Primary cemented unilateral TKR for osteoarthritis<br>50; 50<br>Mean 69.0 (SD 7.7); 68.0 (4.8)<br>89.6%; 87.5%	Skin staples removed on day 10 and wound closure strip applied for 5 days		6 and 12 months<br>2; 2 lost to follow up<br>Low risk of bias<br>At 12 months, VAS pain in silicone gel group mean 2.50 (SD 1.16); control 2.92 (1.90). P=0.201. No difference at 6 months, p=0.886.<br>No wound dehiscence or infection associated with application of silicone gel or petroleum
		After removal of wound closure strip, patients managed operation scars with application of silicone gel for 1 month after stitches removed	After removal of wound closure strip, patients managed operation scars with application of petroleum gel for 1 month after stitches removed	

13. Anabolic steroids

Author<br>Country<br>Recruitment dates<br>Setting	Indication<br>Number randomised intervention; control<br>Age<br>% female	Common rehabilitation strategies		Follow up<br>Losses to follow up intervention; control<br>Risk of bias issues<br>Key results
		Intervention	Control	
		Cold compression and CPM		6, 9 and 12 months

For peer review only - http://bmjopen.bmj.com/site/about/guidelines.xhtml

Hohmann et al. 2010[76]<br>Australia<br>Before 2010<br>1 surgeon	Primary unilateral TKR for osteoarthritis<br>5; 5<br>Mean 66.2 (range 58, 72); 65.2 (59, 72)<br>20%; 40%	On day 5, intramuscular injection of 50 mg Nandrolone decanoate solution. Patients visited every 2 weeks and injections continued for 6 months.	On day 5, intramuscular injection of saline. Patients visited every 2 weeks and injections continued for 6 months.	0; 0 lost to follow up<br>Low risk of bias (but small feasibility study)<br>No separate pain outcome. KSS at 12 months in intervention group mean 91.4 (SD 3.5); control 81.2 (SD 7.1). p=0.03. Difference also at 6 months (p=0.04), marginal at 9 months (p=0.06). Difference in means at 12 months of 10.2 close to MCID of 12.3[79].<br>Intervention group had smaller decrease in bone mineral density at 6 months than controls but not significant

14. Guided imagery

**Author**<br>**Country**<br>**Recruitment dates**<br>**Setting**	**Indication**<br>**Number randomised intervention; control**<br>**Age**<br>**% female**	**Common rehabilitation strategies**		**Follow up**<br>**Losses to follow up intervention; control**<br>**Risk of bias issues**<br>**Key results**
		**Intervention**	**Control**	
Jacobson et al. 2016[139]<br>USA<br>2011-2012<br>1 surgeon	Primary unilateral TKR for osteoarthritis<br>42; 40 (41; 39 received treatment)<br>Mean 65.0 SD 8.6)<br>62.2%	Participants listened to a 19-21-minute CD each day for 2 weeks before and 3 weeks after surgery. Content covered concerns and hopes about TKR with aim to facilitate mind–body connections to promote optimal TKR outcomes.	Participants listened to a 17-21-minute CD each day for 2 weeks before and 3 weeks after surgery. Content comprised poetry, short stories and essays	6 months<br>12; 10 of patients receiving treatments<br>High risk of bias due to large losses to follow up<br>Mean WOMAC pain 2.7 (SD 3.1); 3.5 (SD 3.3). P<0.001<br>Adverse events not reported

For peer review only - http://bmjopen.bmj.com/site/about/guidelines.xhtml

CD compact disc; CPM Continuous passive motion; DN4 Douleur Neuropathique 4; FNB Femoral nerve block; HSS Hospital for Special Surgery; i.v. intravenous; KOOS Knee injury and Osteoarthritis Outcome Score; KSS Knee Society Score; LIA local infiltration analgesia; NRS Numerical rating scale; OKS Oxford Knee Score; ONB obturator nerve block; PCA Patient controlled analgesia; PNB psoas nerve block; SF-36 Short Form 36 Health Survey; S-LANSS Leeds assessment of Neuropathic Symptoms and Signs Pain Scale; SNB Sciatic nerve block; TKR Total knee replacement; VAS Visual analogue scale; VTE Venous thromboembolism; WOMAC Western Ontario and McMaster Universities Osteoarthritis Index.

ITT, ITT CC, POD, MI, PE

For peer review only

For peer review only - http://bmjopen.bmj.com/site/about/guidelines.xhtml

Supplementary material. Risk of bias assessment

Study	Random sequence generation	Allocation concealment	Blinding of participants and personnel	Blind outcome assessment	Incomplete outcome data	Selective reporting	Other bias	Summary
Pain management								
Albrecht et al. 2014[34]	Computer generated	Anaesthetist blind to allocation	Physiotherapists, surgeons, research assistants collecting data, and members of the Acute Pain Service were kept blinded to group allocation.	Physiotherapists, surgeons, research assistants collecting data, and members of the Acute Pain Service were kept blinded to group allocation.	ITT analysis low losses to follow up	No but not checked protocol	Study was terminated early with 61% of planned recruitment completed due to change in standard anaesthesia at hospital	Low
Anastase et al. 2014[113]	Method of the Ulm Institute of Statistics	Method of the Ulm Institute of Statistics	No	No	15:14 lost to follow up	No but not checked protocol	ASA comorbidities differed between groups	High
Aveline et al. 2014[52]	Computer generated	opaque sealed envelopes	Blinded syringes prepared by nurse not involved in study	Yes	Low losses to follow up	Consistent with short term follow up paper	No	Low
Bergeron et al. 2009[105]	Blocks of different sizes according to list preprepared by study epidemiologist	Not described	Anaesthetist not blind. Patients blind	Nurse observers collecting data blind to allocation	32/59 lost to follow up	No but not checked protocol	No	High
Buvanendran et al. 2010[53]	computer generated	Yes, physicians and nurses blind	Yes	Yes	ITT	Protocol not checked	No	Low

For peer review only - http://bmjopen.bmj.com/site/about/guidelines.xhtml

Choy et al. 2011[35]	Computer generated	sealed envelope	No, the catheter was removed at either day 3 or 7	Patient reported outcome. Other outcomes by blinded independent physician	Low losses to follow up	Protocol not checked but reasonable range of outcomes	No	Low
Davidson et al. 2016[111]	Computer generated	Sealed opaque envelopes	Subjects and investigators were not masked to treatment group	Subjects and investigators were not masked to treatment group. PROM	31; 29 lost to follow up	None apparent but protocol not checked	Combined data from 2 RCTs	High
Fan et al. 2016[36]	No details	sealed opaque envelopes	Patients and assessors blind to randomisation	Patients and assessors blind to randomisation	2% protocol violation	No but not checked protocol	No	Low
Foadi et al. 2017[117]	Computer generated	Not described	Not described	Patient reported outcome	>70% questionnaire return	None apparent but protocol not checked	Described as pilot study	Unclear
Gao et al. 2017[37]	Random number table	Not described	Blind to patients	Blind to observers	2; 1; 0	None apparent but protocol not checked	Groups similar at baseline	Low
Ilfeld et al. 2009[108]	Computer generated	Investigators, patients, and all clinical staff were unaware of treatment group assignments	Investigators, patients, and all clinical staff were unaware of treatment group assignments	Investigators, patients, and all clinical staff were unaware of treatment group assignments	4:1 lost to follow up	No but not checked protocol	Basal infusion halved on POD1 in 10 intervention patients compared with 3 controls	High
Ilfeld et al. 2011[109]	Computer generated tables	Solutions prepared by investigational pharmacist	Yes. Intervention and control solutions indistinguishable	Patient reported outcomes. Staff masked to treatment group	11;12 did not have 4 measures out	Protocol not checked	WOMAC and WOMAC domain scores	High

For peer review only - http://bmjopen.bmj.com/site/about/guidelines.xhtml

				assignment performed all measures and assessments	of 6 up to 12 months	but seems reasonable	somewhat lower pre-intervention in extended infusion group. Authors report change scores	
Macrinici et al. 2017[38]	Computer generated	Staff performing injections blind	Anaesthesiologist, surgeons, patients and physical therapists blind to allocation	Yes	3; 4 lost to follow up. 6; 3 complications	None apparent but protocol not checked	Groups similar at baseline	Low
McDonald et al. 2016[45]	Computerised blocked	Study coordinator independent of care and surgery	Patients blind. Surgeon aware of study	Outcome assessment blind to allocation	1; 4 unexplained	None apparent, protocol not checked	Groups similar at baseline	Low
Meunier et al. 2007[51]	Computer generated	Sealed envelope	Randomisation code broken after 1 year	Yes	ITT reported except for 12 month pain outcome	No but not checked protocol	M/F ratio differed	Low
Morin et al. 2005[110]	Allocated randomly	Sealed envelope	All patients received some form of nerve block. Anaesthesiologist not blind to intervention	Observers not blinded	Per protocol analysis	No but not checked protocol	Difference between groups in anesthetist's opinion of difficulty of catheter placement. BMI differed between groups	High
Motififard et al. 2017[46]	Computer generated	Study coordinator independent of care and surgery	Patients blind. Surgeon aware of study	Outcome assessment blind to allocation	3; 7 (1; 4 unexplained)	None apparent, protocol not checked	Groups similar at baseline	Low

For peer review only - http://bmjopen.bmj.com/site/about/guidelines.xhtml

Nader et al. 2012[39]	Computer generated	Opaque envelope	No	Patient reported outcome	1:1 lost to follow up	Protocol not checked but reasonable range of outcomes	FNB group somewhat higher BMI	Low
Niemeläinen et al. 2014[47]	No details	Opaque sealed envelopes	Only research nurse preparing infiltrate aware of randomisation. All other personnel unaware until after 1 year follow up	Only research nurse preparing infiltrate aware of randomisation. All other personnel unaware until after 1 year follow up	All patients who received intervention completed follow up	No but not checked protocol	No	Low
Peng et al. 2014[40]	Computer generated	Not possible	Not possible	Patient reported outcome	31:38 lost at 12 months but ITT and per-protocol analysis	No but not checked protocol	No	Low
Perrin and Purcell 2009[107]	No details	Sealed syringe code stored in pharmacy department	yes	Yes	4 failed to complete protocol	No but not checked protocol	Pilot investigation. High risk of bias due to recruitment difficulties leading to small trial	High
Reinhardt et al. 2014[41]	Computer generated	Maintained by pharmacy department for blinding	Patients blind to intervention	Blinded research assistant and partially physical therapist	0 reported lost to follow up of those who received interventions	No but not checked protocol	No	Low
Seah et al. 2011[48]	Randomisation tables	Sealed envelopes. Anaesthetist and surgeon blind before opening of	Blinding of patients not stated	Blind outcome assessors and PROMs	No losses to follow up reported	No but not checked protocol	No	Low

For peer review only - http://bmjopen.bmj.com/site/about/guidelines.xhtml

		sealed envelope						
Shum et al. 2009[106]	No details	No details	Anaesthetist performing the blocks was not involved in the postoperative follow-up and data collection	Patient reported	14% and 20%	No but not checked protocol	Mean patient weight lower in no FNB group. More favourable mean OKS in no FNB group. Two groups combined for 2 year outcome but not for earlier	High
Spreng et al. 2012[115], Spreng et al. 2010[116]	Hospital pharmacy	Epidural catheter or sham set-up taped along the back of the patient and connected to an infusion pump covered in an opaque bag. Also sham knee catheter	Patients blind	Blind outcome assessment	13%	Limited reporting in conference abstract	Conference abstract only so limited information additional to early follow up paper	Unclear
Wang et al. 2015[112]	No details	No details	Not stated	Not stated	2:4 lost to follow up	No but not checked protocol	No	Unclear
Wegener et al. 2013[42]	No details	Opaque envelope	Patients, surgeons and researchers not blind to intervention	Patients not blinded	2:7:5 lost to follow up	No. Protocol checked	no	Low
Widmer et al. 2012[43]	Coded envelope	Coded envelope	Except for anaesthetist and surgeon	Both the investigators and patients were blinded	None reported as incomplete	No but protocol not checked	No	Low

Williams et al. 2013[49]	Computer generated	Not stated	Patients and assessors blind	Patients and assessors blind	3:1 of those who received treatment	No but not checked protocol	No	Low
Wu and Wong 2014[44]	Computer	Sealed envelopes	No	No	Available cases	No but not checked protocol	No	Low
Wylde et al. 2015[50]	Trials unit	Trials unit	Surgeon and anaesthetist not blind to allocation, Patients blind	Patients and research nurses blind to allocation	ITT with imputed data	No as per protocol	No	Low
Yue et al. 2013[114]	No details	No details	Surgeons and patients were double-blinded to the injection administered	surgeons and patients were double-blinded to the injection administered	Losses to follow up not reported	Limited reporting	No	Unclear
Myofascial trigger point dry needling								
Mayoral et al. 2013[118]	Computerised	Not described	Patient and other researchers apart from physical therapist blind	Patient outcomes	4: 5 loss to follow up	None apparent but protocol not checked	No	High
Tourniquet								
Abdel-Salam and Eyres 1995[119]	Card system	Not described	No	No	No losses to follow up	None apparent but protocol not checked	No	Unclear
Ejaz et al. 2014[54]	Block randomised	Sealed envelopes	Patients unaware	PROM	No losses to follow up of those who received treatments	None apparent but protocol not checked	No	Low
Huang et al. 2017[55]	Computer generated	Sealed opaque envelopes	Surgeons not blind. Patients blind to allocation	Data collector blind to allocation	No losses to follow up reported	None apparent but protocol	Groups similar at baseline	Low

For peer review only - http://bmjopen.bmj.com/site/about/guidelines.xhtml

						not checked		
Liu et al. 2014[56]	Excel	Not described	Patients blind	PROM	No losses	None apparent but protocol not checked.	No	Low
Mittal et al. 2012[57]	Computer generated	Sealed opaque envelopes	Patient blind	Outcome assessors blind. PROM	5:2	None apparent but protocol not checked	Study stopped because of high risk of transfusion in short tourniquet duration group	Low
Şükür et al.2016[120]	Computer generated	Not described	Possibly patients	Outcome assessors blind	No losses to follow up	KSS outcome noted in methods but not presented in results	No	High
Zhang et al. 2017[58]	Excel	Randomisation by blinded researcher.	Patients and nurses on ward blind	Not clear	No losses reported	None apparent but protocol not checked	Groups similar at baseline	Low
Zhang et al.2016[121]	Randomly allocated	Not clear	Not clear	Not clear	Not clear	HSS outcome noted in methods but not presented in results	No	High
Compression bandage								

For peer review only - http://bmjopen.bmj.com/site/about/guidelines.xhtml

Brock et al. 2017[122]	Web-based	Not specified	Not possible	No but PROMs	4; 2 of those receiving intervention	None apparent but protocol not checked	Groups similar at baseline	Low
Blood conservation								
Hourlier et al. 2015[60]	Computer generated	Opaque envelopes	Anaethsetist, surgeon and patient blind to treatment allocation	Assessors blind	No missing data	None apparent but protocol not checked	No	Low
	Computer generated	Sealed opaque envelopes	Surgeons not blind. Patients blind to allocation	Data collector blind to allocation	No losses to follow up reported	None apparent but protocol not checked	Groups similar at baseline	Low
Kim et al. 2014[61]	Computer generated	Not stated	patients blind to allocation	Clinical investigator blind to allocation	No missing data	None apparent but protocol not checked	No	Low
Kusuma et al. 2013[62]	Computer generated	Sealed envelopes	Surgeon and patient blind	Outcome assessor blind	No missing data	None apparent but protocol not checked	No	Low
Napier et al. 2014[63]	Computer generated	Sealed envelopes	Unlikely	Not stated but PROM	low losses to follow up	None apparent but protocol not checked	No	Low
Sa-Ngasoongsong et al. 2011[64]	Computer generated	Sealed envelopes	Surgeon and patient blind	Outcome assessor blind	No missing data	None apparent but	No	Low

For peer review only - http://bmjopen.bmj.com/site/about/guidelines.xhtml

						protocol not checked		
Sa-Ngasoongsong et al. 2013[65]	Computer generated	Sealed envelopes	Surgeon and patient blind	Outcome assessor blind	No missing data	None apparent but protocol not checked	Some difference between groups in pre-operative Hb	Low
Thomas et al. 2001[123]	Not described	not stated	Not reported	Not reported but PROM	Not reported but ITT	None apparent but protocol not checked	No	Unclear
Platelet rich plasma								
Aggarwal et al. 2014[124]	Not described	Opaque envelopes	Patients blind	Patients and examiners blind	No losses to follow up reported	None apparent but protocol not checked	Odd numbers in groups from randomisation	High
Cryotherapy								
Wang 2017[125]	No details	No details	No details	No details	No losses to follow up	None apparent but protocol not checked	Groups similar at baseline	Unclear
Denusomab								
Ledin et al. 2017[66]	Randomisation list produced by the study monitor	Syringes prepared independently	Investigators and patients blind	Unblinding was done after all the data had been locked	0; 2	None apparent but protocol not checked	No	Low
Continuous passive motion								
Beaupré etal. 2001[129]	Computer generated	Sealed envelopes	No	Researcher unaware and PROMs	6:8:6. Results carried	No	4 controls; 1 SB	Unclear

For peer review only - http://bmjopen.bmj.com/site/about/guidelines.xhtml

					forward for missing data		reassigned to CPM	
Bennett et al. 2005[67]	Block	Not stated	Operating surgeon blind. Patient not	Independent assessor blind	1 not included in analyses as not able to achieve 90 degree flexion	No	No	Low
Ersözlü et al. 2009[68]	Divided into groups by random selection	Not described	No	Surgeon score	A diabetic patient from the control group was excluded because of a superficial wound infection, a patient with a cardiac problem in group II due to dysrhythmia, and two patients due to insufficient follow-up.	Not apparent	No differences baseline	Unclear
Kumar et al. 1996[130]	Random number generator	Not stated	No	Not described	Large loss to follow up	Not all data clearly reported	No	High
Leach et al. 2006[126]	Allocation by date of birth	No	No	Blinded evaluation	Large loss to follow up	No	No	High
MacDonald et al. 2000[132]	Computer generated	Allocation concealed	No	Not described	Not reported	Yes, not all outcomes reported in full	No	Unclear
Pope et al. 1997[128]	Not described	Not described	Not described	Not described	No separate reporting. 8 patients (12 knees)	None apparent but protocol	No	High

For peer review only - http://bmjopen.bmj.com/site/about/guidelines.xhtml

					excluding 1 death	not checked		
Sahin et al. 2006[127]	Not described	Not stated	No	Followed up by treating physician	Low loss to follow up	No	No	Unclear
Worland et al. 1998[131]	Not described	Not described	No	Researcher blind	Not reported separately	None apparent but protocol not checked	No	Unclear
Electrical stimulation								
Adravanti et al. 2014[135]	Computer generated	Not described	Research assistant not involved in patient assessment	Principal investigator and all physicians in charge of clinical controls were blinded to patient allocation	78% retained at 6 months	Not apparent but protocol not checked	No	High
Avramidis et al. 2011[69]	Computer generated	Not described	No	Independent assessors blind	3 (intolerance of intervention); 3	Not apparent	Baseline similar	Low
Levine et al. 2013[134]	Drawing papers from hat	Not described	No	Not described. WOMAC PROM	5:9 for KSS pain and WOMAC	Not apparent but protocol not checked	No	Unclear
Moretti et al. 2012[70]	Computer generated	Not described	Physicians, as well as medical assessors, were blinded to the allocation of patients in the study groups	Physicians, as well as medical assessors, were blinded to the allocation of patients in the study groups	No losses reported	Not apparent but protocol not checked	No	Low
Stevens-Lapsley et al. 2012[133]	Stratified	Concealed	No	no but standardised scripts used	5; 6	Not apparent but protocol	WOMAC, BMI unequal at baseline	Unclear

For peer review only - http://bmjopen.bmj.com/site/about/guidelines.xhtml

						not checked		
Rehabilitation								
Hill et al. 2000[138]	Not described	Not stated	Not possible	Not described	No losses to follow up after initial 23222	No but protocol not checked	No	Unclear
Li et al. 2017[71]	Random number table	Not stated	Not possible	Not described but PROM	No losses to follow up	No but protocol not checked	Groups similar at baseline	Low
Liebs et al. 2012[72]	Computer generated	Allocation concealed	Not possible	No but PROM	Low losses to follow up (<20% if deaths and other explained reasons not counted)	No but protocol not checked	No	Low
Mahomed et al. 2008[137]	Block randomisation	Not stated	Not possible	PROM	No loss	No but protocol not checked	ITT gave similar results to analysis according to actual discharge destination (20 inpatient group received home based)	Low
Rahmann et al. 2009[136]	Not described	Sealed numbered envelopes	Not possible	Assessor blind to intervention. Patient reported outcome	Low losses to follow up	No but protocol not checked	TKR patients more likely to receive ward-based control intervention. THR and TKR analysed together	Unclear
Wang et al. 2014[74]	Computer generated	Surgeons did not participate	Surgery was performed by the	Postoperative evaluation was	No loss to follow up	None apparent	No baseline differences	Low

For peer review only - http://bmjopen.bmj.com/site/about/guidelines.xhtml

		in pre-operative grouping	physicians who did not participate in the preoperative grouping and postoperative evaluation	conducted by the physicians who were unaware of the grouping.		but protocol not checked		
Wound management								
Kong et al. 2014[75]	Not described	Not described	Placebo used	Patient outcome	Low loss to follow up	None apparent but protocol not checked	Similar at baseline	Low
Anabolic steroids								
Hohmann et al. 2010[76]	Internet based	Not reported	Placebo trial	Double-blind design minimized systemic error and eliminated observer and experimenter's bias	0 loss to follow up	None apparent	None apparent but small study	Low
Guided imagery								
Jacobson et al. 2016[139]	Permuted blocks	Opaque CD holders	Personnel yes, participants no	Yes	12; 10 of patients receiving treatment	None apparent but protocol not checked	No	High

For peer review only - http://bmjopen.bmj.com/site/about/guidelines.xhtml

References to all RCTs of peri-operative interventions with long-term pain or score follow up, irrespective of risk of bias assessment (numbering consistent with main article)

34. Albrecht E, Morfey D, Chan V, et al. Single-injection or continuous femoral nerve block for total knee arthroplasty? *Clin Orthop Relat Res* 2014;472:1384-93.

35. Choy WS, Lee SK, Kim KJ, et al. Two continuous femoral nerve block strategies after TKA. *Knee Surg Sports Traumatol Arthrosc* 2011;19:1901-8.

36. Fan L, Yu X, Zan P, et al. Comparison of local infiltration analgesia with femoral nerve block for total knee arthroplasty: A prospective, randomized clinical trial. *J Arthroplasty* 2016;31:1361-5.

37. Gao WL, Li H, Liu BQ, et al. Analgesic effect of femoral and sciatic nerve block under multimodal analgesia in total knee arthroplasty. *Chinese Journal of Tissue Engineering Research* 2017;21:2966-72.

38. Macrinici GI, Murphy C, Christman L, et al. Prospective, double-blind, randomized study to evaluate single-injection adductor canal nerve block versus femoral nerve block: postoperative functional outcomes after total knee arthroplasty. *Reg Anesth Pain Med* 2017;42:10-6.

39. Nader A, Kendall MC, Wixson RL, et al. A randomized trial of epidural analgesia followed by continuous femoral analgesia compared with oral opioid analgesia on short- and long-term functional recovery after total knee replacement. *Pain Med* 2012;13:937-47.

40. Peng L, Ren L, Qin P, et al. Continuous femoral nerve block versus intravenous patient controlled analgesia for knee mobility and long-term pain in patients receiving total knee replacement: a randomized controlled trial. *Evid Based Complement Alternat Med* 2014;2014:569107.

41. Reinhardt KR, Duggal S, Umunna BP, et al. Intraarticular analgesia versus epidural plus femoral nerve block after TKA: A randomized, double-blind trial. *Clin Orthop Relat Res* 2014;472:1400-8.

42. Wegener JT, van Ooij B, van Dijk CN, et al. Long-term pain and functional disability after total knee arthroplasty with and without single-injection or continuous sciatic nerve block in addition to continuous femoral nerve block: a prospective, 1-year follow-up of a randomized controlled trial. *Reg Anesth Pain Med* 2013;38:58-63.

43. Widmer BJ, Scholes CJ, Pattullo GG, et al. Is femoral nerve block necessary during total knee arthroplasty?: a randomized controlled trial. *J Arthroplasty* 2012;27:1800-5.

44. Wu JWS, Wong YC. Elective unilateral total knee replacement using continuous femoral nerve blockade versus conventional patient-controlled analgesia: perioperative patient management based on a multidisciplinary pathway. *Hong Kong Med J* 2014;20:45-51.

45. McDonald DA, Deakin AH, Ellis BM, et al. The technique of delivery of peri-operative analgesia does not affect the rehabilitation or outcomes following total knee arthroplasty. *Bone Joint J* 2016;98-B:1189-96.

46. Motififard M, Omidian A, Badiei S. Pre-emptive injection of peri-articular-multimodal drug for post-operative pain management in total knee arthroplasty: a double-blind randomized clinical trial. *Int Orthop* 2017;41:939-47.

47. Niemeläinen M, Kalliovalkama J, Aho A, et al. Single periarticular local infiltration analgesia reduces opiate consumption until 48 hours after total knee arthroplasty. A randomized placebo-controlled trial involving 56 patients. *Acta Orthop* 2014;85:614-9.

48. Seah VWT, Chin PL, Chia SL, et al. Single-dose periarticular steroid infiltration for pain management in total knee arthroplasty: a prospective, double-blind, randomised controlled trial. *Singapore Med J* 2011;52:19-23.

For peer review only - http://bmjopen.bmj.com/site/about/guidelines.xhtml

49. Williams D, Petruccelli D, Paul J, et al. Continuous infusion of bupivacaine following total knee arthroplasty: a randomized control trial pilot study. *J Arthroplasty* 2013;28:479-84.
50. Wylde V, Lenguerrand E, Gooberman-Hill R, et al. Effect of local anaesthetic infiltration on chronic postsurgical pain after total hip and knee replacement: the APEX randomised controlled trials. *Pain* 2015;156:1161-70.
51. Meunier A, Lisander B, Good L. Effects of celecoxib on blood loss, pain, and recovery of function after total knee replacement: a randomized placebo-controlled trial. *Acta Orthop* 2007;78:661-7.
52. Aveline C, Roux AL, Hetet HL, et al. Pain and recovery after total knee arthroplasty: a 12-month follow-up after a prospective randomized study evaluating Nefopam and Ketamine for early rehabilitation. *Clin J Pain* 2014;30:749-54.
53. Buvanendran A, Kroin JS, Della Valle CJ, et al. Perioperative oral pregabalin reduces chronic pain after total knee arthroplasty: a prospective, randomized, controlled trial. *Anesth Analg* 2010;110:199-207.
54. Ejaz A, Laursen AC, Kappel A, et al. Faster recovery without the use of a tourniquet in total knee arthroplasty. *Acta Orthop* 2014;85:422-6.
55. Huang Z, Xie X, Li L, et al. Intravenous and topical tranexamic acid alone are superior to tourniquet use for primary total knee arthroplasty: A prospective, randomized controlled trial. *J Bone Joint Surg (Am)* 2017;99:2053-61.
56. Liu D, Graham D, Gillies K, et al. Effects of tourniquet use on quadriceps function and pain in total knee arthroplasty. *Knee Surg Relat Res* 2014;26:207-13.
57. Mittal R, Ko V, Adie S, et al. Tourniquet application only during cement fixation in total knee arthroplasty: a double-blind, randomized controlled trial. *ANZ J Surg* 2012;82:428-33.
58. Zhang Y, Li D, Liu P, et al. Effects of different methods of using pneumatic tourniquet in patients undergoing total knee arthroplasty: a randomized control trial. *Ir Jr Med Sci* 2017;186:953-9.
59. Brock T, Sprowson A, Muller S, et al. STICKS study - Short-sTretch Inelastic Compression bandage in Knee Swelling following total knee arthroplasty - a feasibility study. *Trials* 2017;18:6.
60. Hourlier H, Reina N, Fennema P. Single dose intravenous tranexamic acid as effective as continuous infusion in primary total knee arthroplasty: a randomised clinical trial. *Arch Orthop Trauma Surg* 2015;135:465-71.
61. Kim TK, Chang CB, Kang YG, et al. Clinical value of tranexamic acid in unilateral and simultaneous bilateral TKAs under a contemporary blood-saving protocol: a randomized controlled trial. *Knee Surg Sports Traumatol Arthrosc* 2014;22:1870-8.
62. Kusuma SK, Sheridan KC, Wasielewski RC. Use of bovine thrombin to reduce blood loss in primary total knee arthroplasty: a controlled randomized trial. *J Arthroplasty* 2013;28:1278-81.
63. Napier RJ, Bennett D, McConway J, et al. The influence of immediate knee flexion on blood loss and other parameters following total knee replacement. *Bone Joint J* 2014;96-B:201-9.
64. Sa-Ngasoongsong P, Channoom T, Kawinwonggowit V, et al. Postoperative blood loss reduction in computer-assisted surgery total knee replacement by low dose intra-articular tranexamic acid injection together with 2-hour clamp drain: a prospective triple-blinded randomized controlled trial. *Orthop Rev* 2011;3:e12.
65. Sa-Ngasoongsong P, Wongsak S, Chanplakorn P, et al. Efficacy of low-dose intra-articular tranexamic acid in total knee replacement; a prospective triple-blinded randomized controlled trial. *BMC Musculoskelet Disord* 2013;14:340.
66. Ledin H, Good L, Aspenberg P. Denosumab reduces early migration in total knee replacement. *Acta Orthop* 2017;88:255-8.

For peer review only - http://bmjopen.bmj.com/site/about/guidelines.xhtml

67. Bennett LA, Brearley SC, Hart JAL, et al. A comparison of 2 continuous passive motion protocols after total knee arthroplasty: a controlled and randomized study. *J Arthroplasty* 2005;20:225-33.
68. Ersözlü S, Sahin O, Ozgür A, et al. The effects of two different continuous passive motion protocols on knee range of motion after total knee arthroplasty: a prospective analysis. *Acta Orthop Traumato* 2009;43:412-8.
69. Avramidis K, Karachalios T, Popotonasios K, et al. Does electric stimulation of the vastus medialis muscle influence rehabilitation after total knee replacement? *Orthopedics* 2011;34:175.
70. Moretti B, Notarnicola A, Moretti L, et al. I-ONE therapy in patients undergoing total knee arthroplasty: a prospective, randomized and controlled study. *BMC Musculoskelet Disord* 2012;13:88.
71. Li L, Wang Z, Yin MH, et al. Effect of early gait training on the functional rehabilitation after total knee arthroplasty. *Chinese Journal of Tissue Engineering Research* 2017;21:4288-93.
72. Liebs T, Herzberg W, Rüther W, et al. Multicenter randomized controlled trial comparing early versus late aquatic therapy after total hip or knee arthroplasty. *Arch Phys Med Rehabil* 2012;93:192-9.
73. Mahomed NN, Davis AM, Hawker G, et al. Inpatient compared with home-based rehabilitation following primary unilateral total hip or knee replacement: a randomized controlled trial. *J Bone Joint Surg (Am)* 2008;90:1673-80.
74. Wang S, Xia J, Wei Y, et al. Effect of the knee position during wound closure after total knee arthroplasty on early knee function recovery. *J Orthop Surg Res* 2014;9:79.
75. Kong CG, Kim GH, Kim DW, et al. The effect of topical scar treatment on postoperative scar pain and pruritus after total knee arthroplasty. *Arch Orthop Trauma Surg* 2014;134:555-9.
76. Hohmann E, Tetsworth K, Hohmann S, et al. Anabolic steroids after total knee arthroplasty. A double blinded prospective pilot study. *J Orthop Surg Res* 2010;5:93.
105. Bergeron SG, Kardash KJ, Huk OL, et al. Functional outcome of femoral versus obturator nerve block after total knee arthroplasty. *Clin Orthop Relat R* 2009;467:1458-62.
106. Shum CF, Lo NN, Yeo SJ, et al. Continuous femoral nerve block in total knee arthroplasty: immediate and two-year outcomes. *J Arthroplasty* 2009;24:204-9.
107. Perrin SB, Purcell AN. Intraoperative ketamine may influence persistent pain following knee arthroplasty under combined general and spinal anaesthesia: a pilot study. *Anaesth Intensive Care* 2009;37:248-53.
108. Ilfeld BM, Meyer RS, Le LT, et al. Health-related quality of life after tricompartment knee arthroplasty with and without an extended-duration continuous femoral nerve block: a prospective, 1-year follow-up of a randomized, triple-masked, placebo-controlled study. *Anesth Analg* 2009;108:1320-5.
109. Ilfeld BM, Shuster JJ, Theriaque DW, et al. Long-term pain, stiffness, and functional disability after total knee arthroplasty with and without an extended ambulatory continuous femoral nerve block: A prospective, 1-year follow-up of a multicenter, randomized, triple-masked, placebo-controlled trial. *Reg Anesth Pain Med* 2011;36:116-20.
110. Morin AM, Kratz CD, Eberhart LH, et al. Postoperative analgesia and functional recovery after total-knee replacement: comparison of a continuous posterior lumbar plexus (psoas compartment) block, a continuous femoral nerve block, and the combination of a continuous femoral and sciatic nerve block. *Reg Anesth Pain Med* 2005;30:434-45.
111. Davidson E, Machi A, Sztain J, et al. Adductor canal versus femoral continuous peripheral nerve blocks for knee arthroplasty: A one-year follow-up pilot study of two randomized, controlled clinical trials. *Reg Anesth Pain Med* 2016;41.
112. Wang F, Zhou Y, Sun J, et al. Influences of continuous femoral nerve block on knee function and quality of life in patients following total knee arthroplasty. *Int J Clin Exp Med* 2015;8:19120-5.

For peer review only - http://bmjopen.bmj.com/site/about/guidelines.xhtml

113. Anastase DM, Winckelmann J, Geiger P. Effects of regional anaesthesia techniques on patients' satisfaction after total knee arthroplasty. *Jurnalul Roman de Anestezie Terapie Intensiva* 2014;21:35-43.
114. Yue D-b, Wang B-l, Liu K-p, et al. Efficacy of multimodal cocktail periarticular injection with or without steroid in total knee arthroplasty. *Chinese Med J* 2013;126:3851-5.
115. Spreng UJ, Andersson E, Dahl V. Long-term outcome after total knee arthroplasty local infiltration analgesia (LIA) vs. Epidural analgesia. *Br J Anaesth* 2012;108:ii419.
116. Spreng UJ, Dahl V, Hjall A, et al. High-volume local infiltration analgesia combined with intravenous or local ketorolac+morphine compared with epidural analgesia after total knee arthroplasty. *Br J Anaesth* 2010;105:675-82.
117. Foadi N, Karst M, Frese-Gaul A, et al. The improved quality of postoperative analgesia after intrathecal morphine does not result in improved recovery and quality of life in the first 6 months after orthopedic surgery: a randomized controlled pilot study. *J Pain Res* 2017;10:1059-69.
118. Mayoral O, Salvat I, Martin MT, et al. Efficacy of myofascial trigger point dry needling in the prevention of pain after total knee arthroplasty: A randomized, double-blinded, placebo-controlled trial. *Evid Based Complement Alternat Med* 2013;2013:Article ID 694941.
119. Abdel-Salam A, Eyres KS. Effects of tourniquet during total knee arthroplasty. A prospective randomised study. *J Bone Joint Surg (Br)* 1995;77:250-3.
120. Sükür E, Öztürkmen Y, Akman Y, et al. The effect of tourniquet and knee position during wound closure after total knee arthroplasty on early recovery of range of motion: a prospective, randomized study. *Arch Orthop Trauma Surg* 2016;136:1773-80.
121. Zhang Q, Dong J, Gong K, et al. Effects of tourniquet use on perioperative outcome in total knee arthroplasty. *Chinese Journal of Reparative and Reconstructive Surgery* 2016;30:421-5.
122. Brock TM, Sprowson AP, Muller S, et al. STICKS study - Short-sTretch Inelastic Compression bandage in Knee Swelling following total knee arthroplasty - a feasibility study. *Trials* 2017;18:6.
123. Thomas D, Wareham K, Cohen D, et al. Autologous blood transfusion in total knee replacement surgery. *Br J Anaesth* 2001;86:669-73.
124. Aggarwal AK, Shashikanth VS, Marwaha N. Platelet-rich plasma prevents blood loss and pain and enhances early functional outcome after total knee arthroplasty: a prospective randomised controlled study. *Int Orthop* 2014;38:387-95.
125. Wang X. Impact of local compression cryotherapy combined with continuous passive motion on the early functional recovery after total knee arthroplasty. *Chinese Journal of Tissue Engineering Research* 2017;21:998-1003.
126. Leach W, Reid J, Murphy F. Continuous passive motion following total knee replacement: a prospective randomized trial with follow-up to 1 year. *Knee Surg Sports Traumatol Arthrosc* 2006;14:922-6.
127. Sahin E, Akalin E, Bircan C, et al. The effects of continuous passive motion on outcome in total knee arthroplasty. *Journal of Rheumatology and Medical Rehabilitation* 2006;17:85-90.
128. Pope RO, Corcoran S, McCaul K, et al. Continuous passive motion after primary total knee arthroplasty. Does it offer any benefits? *J Bone Joint Surg (Br)* 1997;79:914-7.
129. Beaupre LA, Davies DM, Jones CA, et al. Exercise combined with continuous passive motion or slider board therapy compared with exercise only: a randomized controlled trial of patients following total knee arthroplasty. *Physical therapy* 2001;81:1029-37.
130. Kumar PJ, McPherson EJ, Dorr LD, et al. Rehabilitation after total knee arthroplasty: a comparison of 2 rehabilitation techniques. *Clin Orthop Relat R* 1996:93-101.
131. Worland RL, Arredondo J, Angles F, et al. Home continuous passive motion machine versus professional physical therapy following total knee replacement. *J Arthroplasty* 1998;13:784-7.

For peer review only - http://bmjopen.bmj.com/site/about/guidelines.xhtml

132. MacDonald SJ, Bourne RB, Rorabeck CH, et al. Prospective randomized clinical trial of continuous passive motion after total knee arthroplasty. *Clin Orthop Relat Res* 2000:30-5.
133. Stevens-Lapsley JE, Balter JE, Wolfe P, et al. Early neuromuscular electrical stimulation to improve quadriceps muscle strength after total knee arthroplasty: a randomized controlled trial. *Physical therapy* 2012;92:210-26.
134. Levine M, McElroy K, Stakich V, et al. Comparing conventional physical therapy rehabilitation with neuromuscular electrical stimulation after TKA. *Orthopedics* 2013;36:e319-24.
135. Adravanti P, Nicoletti S, Setti S, et al. Effect of pulsed electromagnetic field therapy in patients undergoing total knee arthroplasty: a randomised controlled trial. *Int Orthop* 2014;38:397-403.
136. Rahmann AE, Brauer SG, Nitz JC. A specific inpatient aquatic physiotherapy program improves strength after total hip or knee replacement surgery: a randomized controlled trial. *Arch Phys Med Rehabil* 2009;90:745-55.
137. Mahomed NN, Davis AM, Hawker G, et al. Inpatient compared with home-based rehabilitation following primary unilateral total hip or knee replacement: a randomized controlled trial. *J Bone Joint Surg (Am)* 2008;90:1673-80.
138. Hill SP, Flynn J, Crawford EJP. Early discharge following total knee replacement -- a trial of patient satisfaction and outcomes using an orthopaedic outreach team. *J Orthop Nurs* 2000;4:121-6.
139. Jacobson AF, Umberger WA, Palmieri PA, et al. Guided imagery for total knee replacement: A randomized, placebo-controlled pilot study. *J Altern Complement Med* 2016;22:563-75.

For peer review only

For peer review only - http://bmjopen.bmj.com/site/about/guidelines.xhtml

Reporting checklist for systematic review and meta-analysis.

Based on the PRISMA guidelines.

Instructions to authors

Complete this checklist by entering the page numbers from your manuscript where readers will find each of the items listed below.

Your article may not currently address all the items on the checklist. Please modify your text to include the missing information. If you are certain that an item does not apply, please write "n/a" and provide a short explanation.

Upload your completed checklist as an extra file when you submit to a journal.

In your methods section, say that you used the PRISMA reporting guidelines, and cite them as:

Moher D, Liberati A, Tetzlaff J, Altman DG, The PRISMA Group. Preferred Reporting Items for Systematic Reviews and Meta-Analyses: The PRISMA Statement

		Reporting Item	Page Number
	#1	Identify the report as a systematic review, meta-analysis, or both.	1
Structured summary	#2	Provide a structured summary including, as applicable: background; objectives; data sources; study eligibility criteria, participants, and interventions; study appraisal and synthesis methods; results; limitations; conclusions and implications of key findings; systematic review registration number	2-3,5
Rationale	#3	Describe the rationale for the review in the context of what is already known.	4-5
Objectives	#4	Provide an explicit statement of questions being addressed with reference to participants, interventions, comparisons, outcomes, and study design (PICOS).	5-6
Protocol and registration	#5	Indicate if a review protocol exists, if and where it can be accessed (e.g., Web address) and, if available, provide registration information including the registration number.	5

For peer review only

Eligibility criteria	#6	Specify study characteristics (e.g., PICOS, length of follow-up) and report characteristics (e.g., years considered, language, publication status) used as criteria for eligibility, giving rational	5-6
Information sources	#7	Describe all information sources in the search (e.g., databases with dates of coverage, contact with study authors to identify additional studies) and date last searched.	6
Search	#8	Present full electronic search strategy for at least one database, including any limits used, such that it could be repeated.	See note 1
Study selection	#9	State the process for selecting studies (i.e., for screening, for determining eligibility, for inclusion in the systematic review, and, if applicable, for inclusion in the meta-analysis).	5,6
Data collection process	#10	Describe the method of data extraction from reports (e.g., piloted forms, independently by two reviewers) and any processes for obtaining and confirming data from investigators.	6
Data items	#11	List and define all variables for which data were sought (e.g., PICOS, funding sources), and any assumptions and simplifications made.	5/6
Risk of bias in individual studies	#12	Describe methods used for assessing risk of bias in individual studies (including specification of whether this was done at the study or outcome level, or both), and how this information is to be used in any data synthesis.	6
Summary measures	#13	State the principal summary measures (e.g., risk ratio, difference in means).	7
Planned methods of analyis	#14	Describe the methods of handling data and combining results of studies, if done, including measures of consistency (e.g., I2) for each meta-analysis.	7
Risk of bias across studies	#15	Specify any assessment of risk of bias that may affect the cumulative evidence (e.g., publication bias, selective reporting within studies).	6
Additional analyses	#16	Describe methods of additional analyses (e.g., sensitivity or subgroup analyses, meta-regression), if done, indicating which were pre-specified.	7

For peer review only

Study selection	#17	Give numbers of studies screened, assessed for eligibility, and included in the review, with reasons for exclusions at each stage, ideally with a flow diagram.	See note 2
Study characteristics	#18	For each study, present characteristics for which data were extracted (e.g., study size, PICOS, follow-up period) and provide the citation.	See note 3
Risk of bias within studies	#19	Present data on risk of bias of each study and, if available, any outcome-level assessment (see Item 12).	See note 4
Results of individual studies	#20	For all outcomes considered (benefits and harms), present, for each study: (a) simple summary data for each intervention group and (b) effect estimates and confidence intervals, ideally with a forest plot.	See note 5
Synthesis of results	#21	Present the main results of the review. If meta-analyses are done, include for each, confidence intervals and measures of consistency.	16-23
Risk of bias across studies	#22	Present results of any assessment of risk of bias across studies (see Item 15).	See note 6
Additional analysis	#23	Give results of additional analyses, if done (e.g., sensitivity or subgroup analyses, meta-regression [see Item 16]).	16-23
Summary of Evidence	#24	Summarize the main findings, including the strength of evidence for each main outcome; consider their relevance to key groups (e.g., health care providers, users, and policy makers	16-23
Limitations	#25	Discuss limitations at study and outcome level (e.g., risk of bias), and at review level (e.g., incomplete retrieval of identified research, reporting bias).	24-25
Conclusions	#26	Provide a general interpretation of the results in the context of other evidence, and implications for future research.	23-25
Funding	#27	Describe sources of funding or other support (e.g., supply of data) for the systematic review; role of funders for the systematic review.	26

Author notes

For peer review only

1. 5, Supplemetary material
2. 7, Figure 1
3. 8-15, Table1, Supplementary material
4. 7, Supplementary material
5. 8-15, Table1, Supplementary material
6. 8-15, Supplementary material

The PRISMA checklist is distributed under the terms of the Creative Commons Attribution License CC-BY. This checklist was completed on 21. November 2018 using http://www.goodreports.org/, a tool made by the EQUATOR Network in collaboration with Penelope.ai

For peer review only

BMJ Open

Are peri-operative interventions effective in preventing chronic pain after primary total knee replacement? A systematic review

Journal:	*BMJ Open*
Manuscript ID	bmjopen-2018-028093.R3
Article Type:	Research
Date Submitted by the Author:	14-Aug-2019
Complete List of Authors:	Beswick, Andrew; University of Bristol, School of Clinical Sciences<br>Dennis, Jane; University of Bristol, Musculoskeletal Research Unit<br>Gooberman-Hill, Rachael; University of Bristol, Bristol Medical School<br>Blom, AW; University of Bristol,<br>Wylde, Vikki; University of Bristol, Musculoskeletal Research Unit
**Primary Subject Heading**:	Evidence based practice
Secondary Subject Heading:	Surgery
Keywords:	Knee < ORTHOPAEDIC & TRAUMA SURGERY, Pain management < ANAESTHETICS, REHABILITATION MEDICINE

SCHOLARONE™
Manuscripts

Are peri-operative interventions effective in preventing chronic pain after primary total knee replacement? A systematic review

Andrew D Beswick[1], Jane Dennis[1], Rachael Gooberman-Hill[1,2], Ashley W Blom[1,2], Vikki Wylde[1,2]

[1] Musculoskeletal Research Unit, Translational Health Sciences, Bristol Medical School, University of Bristol

[2] National Institute for Health Research Bristol Biomedical Research Centre, University Hospitals Bristol NHS Foundation Trust and University of Bristol

Corresponding author:

Andrew David Beswick
Musculoskeletal Research Unit
Translational Health Sciences
Bristol Medical School
University of Bristol
Learning and Research Building, Level 1
Southmead Hospital
Bristol
BS10 5NB

email: andy.beswick@bristol.ac.uk
Telephone: 0117 414 7870

For peer review only

For peer review only - http://bmjopen.bmj.com/site/about/guidelines.xhtml

ABSTRACT

Objectives

For many people with advanced osteoarthritis, total knee replacement (TKR) is an effective treatment for relieving pain and improving function. Features of peri-operative care may be associated with the adverse event of chronic pain six months or longer after surgery; effects may be direct, e.g. through nerve damage or surgical complications, or indirect through adverse events. This systematic review aims to evaluate whether non-surgical peri-operative interventions prevent long-term pain after TKR.

Methods

We conducted a systematic review of peri-operative interventions for adults with osteoarthritis receiving primary TKR evaluated in a randomised controlled trial (RCT). We searched *The Cochrane Library*, MEDLINE, Embase, PsycINFO and CINAHL to February 2018. After screening, two reviewers evaluated articles. Studies at low risk of bias according to the Cochrane tool were included.

Interventions

Peri-operative non-surgical interventions; control receiving no intervention or alternative treatment.

Primary and secondary outcome measures

Pain or score with pain component assessed at six months or longer post-operative.

Results

44 RCTs at low risk of bias assessed long-term pain. Intervention heterogeneity precluded meta-analysis and definitive statements on effectiveness. Good-quality research provided generally weak evidence for small reductions in long-term pain with local infiltration analgesia (3 studies), ketamine infusion (1 study), pregabalin (1 study) and supported early discharge (1 study) compared with no intervention. For electric muscle stimulation (2 studies), anabolic steroids (1 study) and walking training (1 study) there was a suggestion of more clinically important benefit. No concerns relating to long-term adverse events were reported. For a range of treatments there was no evidence linking them with unfavourable pain outcomes.

Conclusions

For peer review only - http://bmjopen.bmj.com/site/about/guidelines.xhtml

To prevent chronic pain after TKR, several peri-operative interventions show benefits and merit further research. Good quality studies assessing long-term pain after peri-operative interventions are feasible and necessary to ensure that patients with osteoarthritis achieve good long-term outcomes after TKR.

STRENGTHS AND LIMITATIONS

- For the first time, this systematic review brings together contemporary evidence on aspects of peri-operative care for people with total knee replacement and their effects on long-term pain.
- Only studies assessed to be at low risk of bias were included in the narrative synthesis.
- Intervention and outcome heterogeneity precluded meta-analysis.

KEYWORDS

Total knee replacement; Systematic review; Randomised controlled trial; Peri-operative care; Long-term pain

For peer review only

For peer review only - http://bmjopen.bmj.com/site/about/guidelines.xhtml

BACKGROUND

In the US about 13% of men and 19% of women will be diagnosed with knee osteoarthritis and over half will receive a total knee replacement (TKR)[1]. For people with advanced osteoarthritis unresponsive to pharmacological or conservative treatments, TKR aims to relieve pain and improve function. In the UK nearly 100,000 primary TKRs were performed in 2017[2,3] and in the USA in 2010, an estimated 4.7 million people were living with a TKR[4]. Despite good outcomes for many, some people report long-term pain and are disappointed with their surgery[5,6]. After TKR, pain levels plateau from about 6 months[7,8] after which persistent pain is considered "chronic"[9] and is reported by 10-34% of patients[10].

The mechanisms that influence the development of chronic pain after total knee replacement may be biological, mechanical and psychosocial. Biological explanations include the sensitising impact of long-term pain from osteoarthritis[11,12], inflammation, infection and localised nerve injury[13]. Mechanical explanations include altered gait, prosthesis loosening, and effects on ligaments[14,15]. Psychological factors including depression and catastrophizing may also influence outcomes[16-19]. Much research has focused on pre-operative predictors of outcomes and these include pain intensity, presence of widespread pain, anxiety, depression and catastrophizing.[10,20] However, attempts to target or modify pre-operative care have, as yet, shown no benefit regarding chronic pain or other long-term patient outcomes[10,21-23].

Peri-operative risk factors suggest that appropriate interventions may reduce long-term pain. For example, acute post-operative pain, which may be a direct consequence of the operation, anaesthetic protocol and subsequent analgesia, or related to particular aspects of care, is an acknowledged risk factor for chronic post-surgical pain[24].

In the peri-operative period from hospital admission to the early stages of recovery, care focuses on acute pain management, prevention of adverse events, facilitation of early mobilisation and timely discharge. However, for people with osteoarthritis the key aim of TKR is the achievement of a long-term painless and well-functioning knee with no adverse events. All aspects of peri-operative care should work together to achieve this.

Any treatment in the peri-operative period including pain management, blood conservation, deep vein thrombosis (DVT) and infection prevention, and inpatient rehabilitation could potentially affect patient recovery and chronic pain, either directly or indirectly. Direct mechanisms may be through prevention of nerve damage[25], post-thrombotic syndrome[26], reperfusion injury[27] and articular bleeding[28]. For other treatments, pathways leading to long-

For peer review only - http://bmjopen.bmj.com/site/about/guidelines.xhtml

term pain may be indirect, possibly being mediated through increased risks of adverse events[29]. Irrespective of mechanism, chronic pain is a highly prevalent adverse event after TKR and should be considered along with infection, DVT and other complications in the safety profile of interventions.

Our systematic review of randomised controlled trials (RCTs) aims to evaluate the effectiveness of treatments in the peri-operative period in preventing long-term pain after TKR. By focusing on studies with low risk of bias we aim to identify interventions with robust evidence of long-term effectiveness and identify gaps in the research base.

METHODS

The systematic review protocol was registered (PROSPERO CRD42017041382) and PRISMA reporting guidelines used[30]. A checklist is included as Supplementary material.

Patient and public involvement

As part of the STAR programme of research (NIHR RP-PG-0613-20001), this review benefited from extensive patient and public involvement. Advice was sought from patients and stakeholders at a group discussion in March 2016 with decisions made on inclusion criteria and outcomes. Our patient advisory group comprises five patients with experience of long-term pain after TKR, supported by a dedicated co-ordinator. This group will advise on dissemination of the study results to a general audience including plain language summaries.

Eligibility criteria

Studies were eligible if they satisfied PICOS criteria defined in the protocol. Participants were adults receiving unilateral primary TKR with osteoarthritis in at least 75% of patients. Pharmacological or non-pharmacological interventions commenced in the peri-operative setting with “peri-operative” reflecting the time from hospital admission to immediately post-discharge. Interventions relating to implant designs and surgical procedures were excluded. The comparator was usual care, placebo or an alternative intervention. Outcomes were, in preference, patient-reported joint-specific pain intensity measured by tools such as the Western Ontario and McMaster Universities Osteoarthritis Index (WOMAC) or Oxford Knee Score (OKS). If joint-specific measures were unavailable, pain dimensions from quality of life measures were used or pain rated on a visual analogue scale (VAS) or numerical rating scale (NRS). We also considered composite patient-reported outcome measures and surgeon scores which included a pain intensity component, such as the American Knee Society Score (KSS) and Hospital for

For peer review only - http://bmjopen.bmj.com/site/about/guidelines.xhtml

For peer review only

Special Surgery (HSS) score. Measures specifically of neuropathic pain were also used. The occurrence of adverse events was summarised. The studies included were RCTs with follow up at ≥6 months after surgery and a pain outcome or score including pain. Authors of studies were contacted regarding incomplete pain outcome data.

Database searches

We established an Endnote database of all RCTs in TKR. On 14th February 2018, a search from database inception was conducted in: *The Cochrane Library*; MEDLINE, Embase and PsycINFO on Ovid; and CINAHL on EBSCOhost. The MEDLINE search strategy is included as supplementary material. Citations of key articles were tracked in Web of Science. No language restrictions were applied, and translations made. Studies reported as abstracts or unobtainable using inter-library loans and author contact were excluded.

Screening and data extraction

We imported records into Endnote X7 (Thomson Reuters). An initial screen by one reviewer excluded clearly irrelevant articles. Subsequently, abstracts and full articles were screened independently by two reviewers and reasons for exclusion recorded.

Data were extracted onto piloted forms and an Excel spreadsheet by one reviewer, specifically: country; dates; participants (indication, age, sex); inclusion and exclusion criteria; intervention and control content; setting, timing, duration and intensity of intervention; follow up intervals; losses to follow up; pain outcome data; and serious adverse events. Data was checked against source material by a second reviewer.

Authors were contacted for missing data, and data provided for previous reviews was used[10,31].

Quality assessment

Potential sources of bias were assessed by two experienced reviewers using the Cochrane risk of bias tool[32], specifically: the randomisation process; deviations from intended interventions; missing outcome data (>20%), measurement of the outcome; and selection of the reported result. Studies with serious concerns relating to risk of bias were considered high risk and those with limited reporting unclear risk. Studies with high or unclear risk of bias were excluded from the narrative synthesis but are included in supplementary summary tables with reasons for exclusion.

Data analysis

For peer review only - http://bmjopen.bmj.com/site/about/guidelines.xhtml

Insufficient studies with similar interventions and outcomes were identified for meta-analysis, and a narrative synthesis is presented. Results reported with p-values ≤0.001 were considered “strong” evidence of effectiveness[33], p-values 0.001-0.05 “some” evidence, and p-values 0.05-0.1 “weak” evidence. When authors reported results “statistically significant” with no p-value, this was noted. Where possible, effect sizes were compared with published minimal clinically important differences (MCID). Concerns relating to adverse events were summarised.

RESULTS

Figure 1 shows review progress and reasons for exclusion. Of 1515 RCTs of interventions in the peri-operative setting, 1385 had no long-term follow up. Peri-operative interventions with follow up of ≥six months were evaluated in 130 RCTs of which 76 reported a pain outcome or score with a pain component. Detailed intervention and study characteristics and risk of bias assessments are provided as supplementary material. Studies excluded had concerns for risk of bias pertaining to at least one of: large baseline differences in group characteristics or numbers in groups (n=4); incomplete outcome data (n=15); limited or selective reporting (n=12); or un-blinded surgeon follow up (n=1).

Details of 44 studies assessed to be at low risk of bias are summarised in Table 1. In 34 studies, patients received TKR exclusively for osteoarthritis and in three, 75% or more patients had osteoarthritis. In seven studies there was no information on reason for surgery but there was no suggestion that patients had an indication other than osteoarthritis. Interventions focused on pain management (n=20), tourniquets (n=5), compression bandages (n=1), blood conservation (n=7), denusomab (n=1), continuous passive motion (n=2), electrical stimulation (n=2), rehabilitation (n=4), wound management (n=1) and anabolic steroids (n=1). Primary pain outcome measures reported were VAS or NRS pain (n=12), WOMAC pain (n=7), KOOS pain (n=3), Leeds assessment of Neuropathic Symptoms and Signs Pain Scale (S-LANSS) (n=1), SF-36 bodily pain (n=1), or composite scores including a pain measure, OKS or WOMAC (n=10), KSS or HSS (n=10). Latest outcomes were recorded at 6 months (n=12), 12 months (n=26) and 24 months (n=6). Reporting of adverse events covered the entire follow up period in 27 studies, short-term after surgery in 15 studies, but were not reported in two studies.

For peer review only

For peer review only - http://bmjopen.bmj.com/site/about/guidelines.xhtml

Table 1. Perioperative interventions with follow up for pain or score at 6 months or later and assessed to be at low risk of bias

Study	Treatment common to randomised groups	Intervention	Number patients	Follow up<br><br>Group difference
Pain management: nerve blocks				
Albrecht et al. 2014[34]<br><br>Canada, 2009-2011,<br><br>1 hospital	SNB	1. FNB continuous high<br><br>2. FNB continuous low<br><br>3. FNB single	99	1 year<br><br>WOMAC score: no difference (p=0.68)
Choy et al. 2011[35]<br><br>Korea, 2006-2007,<br><br>1 surgeon	PCA	1. FNB continuous long<br><br>2. FNB continuous short	61	1 year<br><br>WOMAC pain: no difference (p=0.2)
Fan et al. 2016[36]<br><br>China, 2012-2014,<br><br>2 surgeons	PCA	1. FNB single<br><br>2. LIA	157	1 year<br><br>KSS: no difference (p=0.51)
Gao et al. 2017[37]<br><br>China, 2014-2015,<br><br>1 centre	LIA	1. General anaesthesia<br><br>2. FNB single<br><br>3. FNB/ SNB single	150	6 months<br><br>HSS score: no significant difference (p> 0.05)
Macrinici et al. 2017[38]<br><br>USA, Before 2017<br><br>1 centre	LIA	1. ACB single<br><br>2. FNB single	98	6 months<br><br>VAS pain: no difference
Nader et al. 2012[39]<br><br>USA, 2007-2008,<br><br>1 surgeon	PCA	1. FNB continuous<br><br>2. Oral opioid	62	1 year<br><br>NRS pain stair: some evidence favouring opioid (p=0.01) but not consistent. Overall NRS pain: no difference (p=1.0)<br><br>VTE: concern opioid
Peng et al. 2014[40]<br><br>China, Before 2014,		1. FNB continuous<br><br>2. PCA	280	6 months and 1 year<br><br>NRS pain: some evidence favouring FNB at 6 months

For peer review only - http://bmjopen.bmj.com/site/about/guidelines.xhtml

1 centre				(p=0.021); no difference at 1 year (p=0.273)
Reinhardt et al. 2014[41]<br><br>USA, 2010-2012,<br><br>2 surgeons		1. FNB single/ epidural<br><br>2. LIA 48 hours	94	1 year<br><br>VAS pain: no difference
Wegener et al. 2013[42]<br><br>The Netherlands, 2008-2010,<br><br>1 centre	FNB	1. SNB single<br><br>2. SNB continuous<br><br>3. PCA	89	1 year<br><br>WOMAC pain: no difference (p=0.81)
Widmer et al. 2012[43]<br><br>Australia, before 2012,<br><br>2 surgeons	LIA, PCA	1. FNB single<br>2. Control no FNB	55	1 year<br><br>WOMAC pain: no difference (p=0.74)
Wu and Wong 2014[44]<br><br>China, 2009-2011,<br><br>1 centre		1. FNB continuous<br><br>2. PCA	60	6 months<br><br>KSS: no difference (p=0.513)
Pain management: LIA				
McDonald et al. 2016[45]<br><br>UK, 2010-2011<br><br>1 hospital		1. LIA<br><br>2. PCA	222	1 year<br><br>OKS: no difference (p=0.915)
Motififard et al. 2017[46]<br><br>Iran, 2014-2015<br><br>1 hospital		1. LIA pre-emptive injection<br><br>2. Control saline with epinephrine	120	6 months<br><br>KSS: weak evidence favouring LIA (p=0.07). Difference between groups (14.2/200) less than MCID (12.3/200).
Niemeläinen et al. 2014[47]<br><br>Finland, 2011-2012<br><br>1 hospital	PCA	1. LIA<br><br>2. Control saline	56	1 year<br><br>OKS: weak evidence from means and confidence intervals favouring LIA. Difference (2.7/48) less than MCID (4.0/48)
Seah et al. 2011[48]<br><br>Singapore, 2004-2005	PCA	1. LIA with corticosteroid<br><br>2. LIA no corticosteroid	100	6 months and 2 years<br><br>OKS: no difference

For peer review only - http://bmjopen.bmj.com/site/about/guidelines.xhtml

For peer review only

1 hospital				
Williams et al. 2013[49]<br>Canada, Before 2013<br>2 surgeons	LIA, PCA	1. LIA 48 hours<br>2. Control saline	51	6 months and 1 year<br>VAS pain: no difference (6 months p=0.836, 1 year p=0.767)
Wylde et al. 2015[50]<br>UK, 2009-2012<br>1 centre	FNB, PCA	1. LIA<br>2. Control no LIA	280	6 months and 1 year<br>WOMAC pain: weak evidence favouring LIA at 6 months p=0.063; 1 year p=0.107. Mean difference at 1 year (3.8/100) lower than MCID (8–9/100)
Pain management: Celecoxib				
Meunier et al. 2007[51]<br>Sweden, 2004-2005<br>1 centre	PCA	1. Celecoxib<br>2. Control placebo	44	1 year<br>KOOS/VAS pain: no difference
Pain management: Ketamine/ Nefopam				
Aveline et al. 2014[52]<br>France, 2005<br>1 centre	PCA	1. Ketamine infusion<br>2. Nefopam infusion<br>3. Control saline	75	6 months and 1 year<br>DN4/VAS pain: some evidence favouring ketamine (for DN4 p=0.02). Few patients had neuropathic pain at 12 months.
Pain management: Pregabalin				
Buvanendran et al. 2010[53]<br>USA, 2006-2007<br>Single centre	LIA, PCA	1. Pregabalin<br>2. Control placebo	240	6 months<br>NRS pain: some evidence favouring pregabalin at 6 months (p=0.0176)<br>S-LANSS pain: no neuropathic pain reported in pregabalin group compared with 5.2% of patients in control group (p=0.014)<br>Sedation and confusion day 0 and day 1: concern pregabalin

For peer review only

For peer review only - http://bmjopen.bmj.com/site/about/guidelines.xhtml

Tourniquet				
Ejaz et al. 2014[54]<br>Denmark, 2011-2012<br>1 centre	Tranexamic acid	1. Tourniquet<br>2. Tourniquet not inflated	64	6 months and 1 year<br>KOOS pain: no significant difference
Huang et al. 2017[55]<br>China, 2015<br>1 centre	Tranexamic acid	1. Tourniquet<br>2. No tourniquet	100	6 months<br>VAS pain: no difference (p=0.728)<br>Wound: concern tourniquet
Liu et al. 2014[56]<br>Australia, Before 2014<br>1 surgeon		1. Tourniquet<br>2. Tourniquet not inflated	20	6 months and 1 year<br>OKS: no significant difference<br>Transfusion: concern tourniquet
Mittal et al. 2012[57]<br>Australia, 2008-2010<br>1 centre		1. Tourniquet short duration<br>2. Tourniquet long duration	65	1 year<br>OKS: weak evidence from means and Cis on graph favouring long duration at 1 year. Mean difference (5) greater than MCID (4)<br>Transfusions/ adverse events: concern short
Zhang et al. 2017[58]<br>China, 2008-2011<br>1 surgeon		1. Tourniquet for entire operation<br>2. Tourniquet removed before wound closure<br>3. Tourniquet from first bone osteotomy until closure	150	6 months<br>HSS score: no difference (p=0.839)<br>Transfusions: concern late tourniquet start in groups 1 and 2
Compression bandage				
Brock et al. 2017[59]<br>UK, 2013-2014<br>1 hospital	Hydrocolloid dressing	1. Compression bandage<br>2. Standard crepe bandage	49	6 months<br>OKS: no difference (p=0.58)
Blood conservation				

For peer review only - http://bmjopen.bmj.com/site/about/guidelines.xhtml

Hourlier et al. 2015[60]<br><br>France, 2009-2010<br><br>1 hospital	Drain, tourniquet, electrocautery	1. Continuous infusion tranexamic acid<br><br>2. Control saline	106	6 months<br><br>KSS: no difference (p=0.90)
Huang et al. 2017[55]<br><br>China, 2015<br><br>1 centre	Tourniquet	1. Intravenous and topical tranexamic acid<br><br>2. No tranexamic acid	100	6 months<br><br>VAS pain: no difference (p=0.728)<br><br>HSS score: strong evidence favouring tranexamic acid (p<0.001). Mean difference (1.4/100) lower than MCID (8.3/100)<br><br>Blood loss: control concern
Kim et al. 2014[61]<br><br>Korea, 2009-2011<br><br>1 hospital	Tourniquet, drain, compressive dressing	1. Tranexamic acid<br><br>2. No tranexamic acid	180	1 year<br><br>WOMAC pain: no significant difference<br><br>Transfusion: control concern
Kusuma et al. 2013[62]<br><br>USA, Before 2013<br><br>1 hospital	Tourniquet, Esmarch bandage, electrocautery	1. Thrombin infusion<br><br>2. No thrombin infusion	80	6 months, 1 and 2 years<br><br>KSS: no difference (p=0.45)
Napier et al. 2014[63]<br><br>UK, 2003-2004<br><br>1 hospital		1. Passive flexion<br><br>2. Passive extension	180	1 year<br><br>OKS: no difference (p=0.27)<br><br>Transfusion: extension concern
Sa-Ngasoongsong et al. 2011[64]<br><br>Thailand, 2008-2009<br><br>1 hospital	Drain and compressive dressing	1. Tranexamic acid<br><br>2. Control saline	48	6 months<br><br>WOMAC score: no difference (p=0.282)<br><br>Transfusion: control concern
Sa-Ngasoongsong et al. 2013[65]<br><br>Thailand, 2010-2011<br><br>1 hospital	Drain and compressive dressing	1. Tranexamic acid 500mg<br><br>2. Tranexamic acid 250mg<br><br>3. Control saline	135	1 year<br><br>WOMAC score: no difference (p=0.42)<br><br>Transfusions: control and 250mg group concerns

For peer review only - http://bmjopen.bmj.com/site/about/guidelines.xhtml

Denusomab				
Ledin et al. 2017[66]<br><br>Sweden, 2012-2014<br><br>2 centres		1. Denusomab<br><br>2. Placebo	50	1 and 2 years<br><br>KOOS pain: no significant difference
Continuous passive motion				
Bennett et al. 2005[67]<br><br>Australia, 1997-2000<br><br>1 hospital	Physiotherapy	1. Standard CPM<br><br>2. Early flexion CPM<br><br>3. No CPM	147	1 year<br><br>KSS: no significant difference
Ersözlü et al. 2009[68]<br><br>Turkey, 2003-2004<br><br>1 hospital	Physiotherapy	1. CPM low and increasing<br><br>2. CPM high and increasing<br><br>3. No CPM	90	2 years<br><br>KSS: no difference (p=0.67)
Electrical stimulation				
Avramidis et al. 2011[69]<br><br>Greece, 2005-2006<br><br>1 hospital	Physiotherapy	1. Transcutaneous electric muscle stimulation<br><br>2. No treatment	76	1 year<br><br>SF-36 bodily pain: strong evidence favouring electrical stimulation (p<0.001). Mean difference (12.5/100) close to MCID (16.9/100).<br><br>OKS/ KSS: no difference
Moretti et al. 2012[70]<br><br>Italy, 2008-2010<br><br>1 hospital	Rehabilitation protocol	1. Pulsed electromagnetic fields<br><br>2. No treatment	30	6 months and 1 year<br><br>VAS pain: some evidence favouring electrical stimulation (p<0.05). Mean difference (2.1/10) greater than MCID (16.1/100)<br><br>Knee swelling: electrical stimulation concern
Rehabilitation				
Li et al. 2017[71]<br><br>China, 2015-2016	Standard rehabilitation	1. Walking guidance and training	86	6 months

For peer review only

For peer review only - http://bmjopen.bmj.com/site/about/guidelines.xhtml

1 hospital		2. No treatment		VAS pain/ HSS score: some evidence favouring walking (both p<0.01). Mean VAS pain difference (2.4/100) greater than MCID (16.1/100)
Liebs et al. 2012[72]<br><br>Germany, 2003-2004<br><br>4 hospitals	CPM, physiotherapy, post-discharge aquatic therapy	1. Early aquatic therapy<br><br>2. Delayed aquatic therapy	185	6 months, 1 and 2 years<br><br>WOMAC pain: no difference (p=0.22 at 12 months)
Mahomed et al. 2008[73]<br><br>Canada, 2000-2002<br><br>2 centres	Physiotherapy	1. Multidisciplinary supported early discharge and home physiotherapy<br><br>2. Transfer to rehabilitation centre	234 hip or knee replace ment	1 year<br><br>WOMAC pain: weak evidence favouring supported discharge (p=0.08). Mean difference (4) less than MCID (8-9)
Wang et al. 2014[74]<br><br>China, 2009-2010<br><br>1 centre		1. Wound closure in flexion<br><br>2. Wound closure in extension	80	6 months<br><br>VAS pain: no difference (p=0.64)
Wound management				
Kong et al. 2014[75]<br><br>South Korea, 2011<br><br>1 surgeon	Skin staples and closure strip	1. Silicone gel<br><br>2. Petroleum gel	100	6 months and 1 year<br><br>VAS pain: no difference (6 months p=0.886, 1 year p=0.201)
Anabolic steroids				
Hohmann et al. 2010[76]<br><br>Australia, Before 2010<br><br>1 surgeon	CPM. Cold compression,	1. Intramuscular nandrolone injections<br><br>2. Saline injections	10	6 and 9 months, 1 year<br><br>KSS: some evidence favouring nandrolone (6 months p=0.04, 9 months p=0.06, 12 months p=0.03). Difference at 12 months (10.2) close to MCID (12.3)<br><br>Bone mineral density: weak evidence favouring nandrolone

ACB adductor canal block; CPM Continuous passive motion; DN4 Douleur Neuropathique 4; FNB Femoral nerve block; HSS Hospital for Special Surgery; KOOS Knee injury and Osteoarthritis Outcome Score; KSS Knee Society Score; LIA local infiltration analgesia; MCID minimal clinically important

For peer review only - http://bmjopen.bmj.com/site/about/guidelines.xhtml

difference; NRS Numerical rating scale; OKS Oxford Knee Score; PCA Patient controlled analgesia; SF-36 Short Form 36 Health Survey; S-LANSS Leeds assessment of Neuropathic Symptoms and Signs Pain Scale; SNB Sciatic nerve block; VAS Visual analogue scale; VTE Venous thromboembolism; WOMAC Western Ontario and McMaster Universities Osteoarthritis Index.

For peer review only

For peer review only - http://bmjopen.bmj.com/site/about/guidelines.xhtml

Pain management

We identified 20 RCTs with 2393 participants evaluating components of multi-modal pain management. Four studies each were from China and the USA, two each from Canada and the UK and one each from Australia, Finland, France, Iran, Singapore, South Korea, Sweden and The Netherlands. All were conducted at a single centre and, in those with dates, participants were recruited between 2004 and 2015. Sample sizes ranged from 44 to 280 participants, with a median of 96. Four studies had three trial arms and 16 had two. The range of mean or median ages of participants in randomised groups was 61 to 73 years and, in 17/19 studies with data, a majority of participants were women.

Femoral nerve block

Femoral nerve blocks (FNB) were studied in 10 RCTs.

Three RCTs compared FNB with no FNB. In one study with 55 patients, WOMAC pain scores at one year were similar in patients receiving single-shot FNB and untreated controls[43]. All patients received local anaesthetic infiltration (LIA) and patient-controlled analgesia (PCA). In another study with all participants receiving LIA, 150 were randomised to receive single-shot FNB with or without sciatic nerve block (SNB), or general anaesthesia[37]. There were no differences in HSS scores between groups at six months. Continuous FNB was compared with oral hydrocodone opioid in 62 patients receiving PCA[39]. There was some evidence for ‘pain using stairs’ favouring hydrocodone (p=0.01) but no difference in overall NRS-rated pain at one year and concern over venous thromboembolism in 4/31 participants treated with hydrocodone.

In two RCTs, continuous FNB was compared with PCA. In one study with 60 participants, the KSS at six months was similar between groups[44]. In another study with 280 participants, there was some evidence for higher incidence of NRS-rated pain at six months in the PCA group than the FNB group (p=0.021) but not at 12 months (p=0.273).[40]

Two RCTs compared FNB with LIA. In one study, all 157 participants also received PCA[36]. At one year, KSS values were similar in single-shot FNB and LIA groups. In the other study, 94 participants were randomised to receive single-shot FNB with continuous epidural infusion or LIA through an intra-articular catheter[41]. VAS-rated pain was similar between groups at one year.

In two RCTs, FNB procedures were compared. In one study with 99 patients randomised to two FNB concentrations, there was no difference in WOMAC score between groups at 12 months[34]. In another study with 61 participants allocated to two different durations of FNB,

For peer review only - http://bmjopen.bmj.com/site/about/guidelines.xhtml

there was no difference in WOMAC pain scores at one year[35]. In these studies, all participants received either SNB[34] or PCA[35].

Single-shot FNB was compared with single adductor canal block in one RCT with 98 participants, all receiving LIA[38]. At six months there was no difference in VAS-rated pain.

Sciatic nerve block

In one study, 89 patients were randomised to single-shot SNB, continuous SNB, or PCA[42]. All patients received FNB. At 12 months, there were no differences in pain for single-shot SNB and continuous SNB on the WOMAC pain scale or VAS-rated pain at rest or during mobilisation. Similarly, there were no differences between single-shot SNB and PCA in WOMAC pain scale or VAS-rated pain at rest or during mobilisation, or between continuous SNB and PCA.

Local anaesthetic infiltration

In six RCTs, treatment with LIA was investigated.

Three RCTs compared intra-operative LIA with placebo or no intervention. In one study, all 280 participants received FNB and PCA[50]. There was weak evidence that WOMAC pain scores were better in the LIA group at six (p=0.063) but not at 12 months (p=0.107) when the difference in means of 3.8/100 was lower than the MCID of 8-9/100 reported by Ehrich and colleagues[77]. In another study, 56 patients received LIA including ketorolac, or saline placebo, and all received PCA[47]. At one year, mean differences and confidence intervals provided weak evidence that OKS scores were better in the LIA group but the difference in means of 2.7/48 was less than the MCID of 4/48 reported by Beard and colleagues[78]. LIA before surgical incision was compared with placebo in one study with 120 participants[46]. None received FNB or PCA. There was weak evidence for a better KSS (function and knee score components) at six months in those receiving LIA (p=0.07) with a difference in means of 14.2/200 exceeding the MCID of 12.3/200 reported by Lee and colleagues[79].

In one study, 51 participants received LIA intra-operatively, followed by PCA[49]. Those randomised to further post-operative catheter-delivered LIA with ketorolac, or saline placebo had similar VAS-rated pain at six and 12 months.

LIA delivered as an injection and post-operative infusion was compared with epidural PCA in one study with 222 patients[45]. There was no difference between groups in OKS at 12 months.

In one study of 100 participants, LIA with or without corticosteroid were compared[48]. All patients received PCA. At two years there was no difference in OKS between groups.

For peer review only - http://bmjopen.bmj.com/site/about/guidelines.xhtml

Oral celecoxib

In one RCT, 44 participants received oral celecoxib or placebo[51], as well as PCA. There were no differences between groups in KOOS or VAS-rated pain at 12 months.

Ketamine or nefopam infusion

In one RCT, ketamine infusion, nefopam infusion and saline placebo were compared in 75 patients, all of whom received PCA[52]. VAS-rated pain on movement did not differ between groups at 12 months. For the Douleur Neuropathique 4 (DN4) measure of neuropathic pain, there was some evidence favouring ketamine over placebo at six and 12 months (p=0.02), but overall, few patients reported neuropathic pain at 12 months.

Pregabalin

Oral pregabalin was compared with placebo in one RCT with 240 participants[53]. All received LIA and PCA. At six months, there was some evidence for better NRS pain in patients receiving pregabalin compared with placebo (p=0.0176) but the difference in means of 0.54/10 was less than the MCID of 1/10 reported by Salaffi and colleagues[80]. No participants receiving pregabalin reported neuropathic pain when assessed using the S-LANSS, compared with 5.2% of those receiving placebo (p=0.014). Patients receiving pregabalin were more likely to be sedated and confused in the first two days after surgery.

Tourniquet

Five studies with 399 participants explored tourniquet use to provide a bloodless field. Two studies each were from Australia and China, and one from Denmark. All were conducted at a single centre with participants recruited between 2008 and 2015. Sample sizes ranged from 20 to 150 participants, with a median of 65. The range of mean ages of participants in randomised groups was 66 to 71 years and in 3/5 studies, a majority of participants were women.

In three RCTs, participants received TKR with or without a tourniquet. In one study with 64 patients, a difference in KOOS pain favouring tourniquet use was not significant at six or 12 months[54]. In another study with 20 patients, the OKS was not significantly different between groups at six or 12 months[56]. There were three blood transfusions in the tourniquet group, compared with none in the ‘no tourniquet’ group. In the third study with 100 participants, VAS-rated pain and HSS scores were similar between groups at 6 months[55]. Six cases of wound ooze occurred in the tourniquet group.

For peer review only - http://bmjopen.bmj.com/site/about/guidelines.xhtml

In two RCTs, short and long-duration tourniquet use were compared. In one study with 65 participants, there was weak evidence based on graphical representation of means and confidence intervals for improved OKS at 12 months in the long-duration group and the difference in means of 5/48[57] was greater than the MCID of 4/48. Adverse events were reported by 62% of participants receiving short-duration tourniquet compared with 38% in the long-duration group. The study was terminated early as 10 blood transfusions were required in the short-duration group compared with three in the long-duration group. In the second study with 150 participants, tourniquets were used in three different periods during surgery[58]. At six months, there were no differences between groups in HSS scores.

Blood conservation

Seven studies with 829 participants evaluated strategies to limit blood loss after TKR. Two studies were from Thailand, and one each from China, France, South Korea, the UK and the USA. All were conducted at a single centre with participants recruited between 2003 and 2015 when stated. Sample sizes ranged from 48 to 180 participants, with a median of 106. One study had three trial arms. The range of mean ages of participants in randomised groups was 65 to 74 years and in all studies, a majority of participants were women.

Tranexamic acid

Five RCTs evaluated tranexamic acid.

Tranexamic acid injections or infusions were compared with saline placebo or untreated control in four RCTs[55,61,64,65]. In all studies, control patients required more blood transfusions. In one study including 180 participants comparing intravenous tranexamic acid with untreated controls, there was no significant difference in WOMAC pain scores at one year[61]. In another study with 48 participants comparing intra-articular tranexamic acid injection with saline placebo, there was no significant difference in WOMAC scores at six months[64]. One study with 135 participants compared two intra-articular tranexamic acid doses and saline control[65]. There were no significant differences in WOMAC scores at one year. Intravenous and intra-articular tranexamic was compared with untreated controls in one study with 100 participants[55]. VAS-rated pain at six months was similar between groups, but there was strong evidence favouring tranexamic acid for HSS scores ($p<0.001$) although the difference in means of 1.4/100 was lower than the MCID of 8.3/100 reported by Singh and colleagues[81].

In one study, continuous tranexamic acid infusion was compared with a single bolus in 106 patients[60]. There was no difference between groups in KSS at six months or blood loss.

For peer review only - http://bmjopen.bmj.com/site/about/guidelines.xhtml

Thrombin infusion

In one RCT with 80 participants, thrombin infusion was compared with untreated control[62]. At one year there was no difference between groups in pain measured on the KSS.

Flexion or extension

For blood management, operated knees were kept in passive flexion or passive extension after surgery in one RCT with 180 patients[63]. At one year, OKS was similar between groups. Transfusion requirement was greater in patients with passive extension.

Compression bandage

One RCT conducted at a single UK centre with 49 participants recruited between 2013 and 2014 compared compression bandaging to reduce post-operative knee swelling with standard bandaging. The mean age of participants was about 69 years and a majority were women. OKS was similar in randomised groups at six months[59].

Wound management

One RCT with recruitment in 2011 at a single centre in South Korea evaluated a wound care strategy to limit post-operative scar pain. The mean age of participants was about 69 years and a majority were women. Investigators compared silicone gel application to the surgical scar with placebo in 100 participants[75]. There were no significant differences in VAS-rated pain at six and 12 months.

Denusomab

One RCT evaluated use of the antiresorptive monoclonal antibody Denusomab to promote bone healing. The study was conducted in two centres in Sweden with recruitment of 50 participants between 2012 and 2014. The mean age of participants was about 65 years and a majority were women. At 12 and 24 months there were no significant differences between groups in KOOS pain[66].

Continuous passive motion

Two RCTs with 237 participants evaluated use of continuous passive motion (CPM) to minimise joint stiffness and improve range of movement. Studies were conducted in single centres in Australia and Turkey with participant recruitment between 1997 and 2004 and both had three trial arms. Sample sizes were 90 and 147 participants. The mean ages of participants in studies were about 63 and 72 years and a majority of participants were women. In one study, 90

For peer review only - http://bmjopen.bmj.com/site/about/guidelines.xhtml

participants were randomised to no CPM, CPM at low flexion from post-operative day 1–7, or CPM at high flexion from post-operative day 3–7[68]. There was no significant difference between groups in KSS at two years. In the other study, 147 participants were randomised to CPM with increasing range of movement from day 1–6, early flexion CPM from day 0–6, or no CPM[67]. There were no significant differences between groups in KSS at 12 months.

Electrical stimulation

Two RCTs with 106 participants conducted in single centres in Greece and Italy evaluated electrical stimulation which is believed to have anti-inflammatory activity and limit muscle atrophy. Studies included 76 and 30 participants recruited between 2005 and 2010. The mean ages of participants were 71 and 70 years and in one study that reported it, a majority of participants were female.

In one study with 76 participants receiving transcutaneous electric muscle stimulation from post-operative day two for six weeks or no intervention, Short Form 36 bodily pain showed strong evidence for greater improvement at one year in the intervention group compared to control ($p<0.001$)[69]. The difference in means of 12.5/100 was close to the MCID of 16.9/100 reported by Escobar and colleagues[82]. There were no differences in OKS or KSS scores. In another study with 30 participants, pulsed electromagnetic fields from post-operative day 7 were compared with untreated control[70]. At 12 months, there was some evidence that VAS-rated pain was lower in intervention patients compared with controls ($p<0.05$). The difference in means of 2.1/10 was greater than the MCID of 16.1/100 reported by Danoff and colleagues[83]. Knee swelling was common during the intervention.

Rehabilitation

Four RCTs with 585 participants recruited between 2000 and 2016 evaluated features of early rehabilitation focusing on regaining range of movement, functional independence and improving mobility. Two studies were conducted at single centres in China and at two and four centres in Canada and Germany respectively. Sample sizes ranged from 80 to 234 participants, with a median of 136. The range of mean ages of participants in randomised groups was 68 to 78 years and in 3/4 studies, a majority of participants were women.

Walking guidance and training

In one study, 86 participants were randomised to walking guidance and training from post-operative day two or no intervention further to standard rehabilitation[71]. At six months, there was some evidence that those receiving intervention had lower VAS-rated pain ($p<0.01$) and

For peer review only - http://bmjopen.bmj.com/site/about/guidelines.xhtml

HSS score (p<0.01) than controls. The difference in mean VAS-rated pain of 2.4/10 was greater than the MCID of 16.1/100.

Flexion or extension during knee closure

Targeting improved functional recovery, wound closure performed in 90° flexion was compared with wound closure in full extension in one study with 80 participants[74]. There was no difference between groups in VAS-rated pain at six months.

Aquatic therapy

In one study with 185 participants, aquatic therapy commencing on post-operative day six was compared with aquatic therapy commencing on day 14[72]. Patients reported similar WOMAC pain at 12 and 24 months.

Supported early discharge

In one study, early discharge supported by physiotherapist home visits and outpatient or self-directed physiotherapy was compared with two weeks of rehabilitation centre-based usual care[73]. The study included 234 individuals receiving TKR or total hip replacement. Compared with usual care, there was weak evidence that patients with early discharge had lower WOMAC pain scores at 12 months (p=0.08). The difference in means of 4 was less than the MCID of 8-9/100. Results were not presented separately but did not differ between patients with TKR or total hip replacement.

Anabolic steroids

Searches identified one study of anabolic steroids to improve post-operative muscle strength conducted in one centre in Australia with recruitment of 10 participants before 2010. The mean age of participants was about 66 years and a minority were women. Participants received intramuscular nandrolone injections or saline from post-operative day five for six months. KSS results indicated some evidence for improvement in the intervention group compared with controls at 12 months (p=0.03)[76]. The difference in means of 10.2/200 was close to the MCID of 12.3/200.

DISCUSSION

Much research in TKR aims to identify treatments that facilitate a speedy recovery with minimal short-term pain. However, patients choose to have joint replacement for long-term pain relief and reduction in functional limitations. Thus, changes to peri-operative care, supported by short-

For peer review only - http://bmjopen.bmj.com/site/about/guidelines.xhtml

term RCT evidence, should be backed up with evidence about long-term effectiveness for reducing pain and reassurance that there are no long-term unfavourable consequences. To this end, we synthesised evidence from RCTs evaluating peri-operative interventions which have considered their long-term effects on pain outcomes.

Consistent with its status as a key peri-operative risk factor, a major focus of research into improving long-term pain after TKR has been through prevention of acute post-operative pain using multimodal analgesia. Our review provides good quality evidence for a small benefit for intra-articular LIA injections, as previously shown in short-term studies[31,84], oral pregabalin, oral opioids, and in relation to neuropathic pain, ketamine infusion. As well as potential benefits for reduced long-term pain, future studies will need to consider concerns associated with these interventions which may not have been identified in small studies including infection[31], venous thromboembolism[39] and sedation[53].

Nerve blocks are effective for managing peri-operative pain[85] but we identified no long-term benefit. In single studies, there was no benefit for nefopam infusion, oral celecoxib or LIA with additional corticosteroid. Regarding future studies, standardisation of the multi-modal regimen will allow evaluation of extra or alternative components in multiple studies in different settings. With such an approach, convincing evidence will accrue to guide multimodal pain management.

Some interventions targeted the prevention of adverse events and facilitation of early mobilisation. Tranexamic acid is highly effective in reducing blood transfusions during TKR[86] and we found no evidence that tranexamic acid affects long-term pain or, consistent with registry studies[87,88], adverse events. Single RCTs of thrombin infusion and maintenance of knee in flexion to prevent blood loss showed no effect on long-term pain. Tourniquets improve intraoperative visualisation of the joint, reduce blood loss and facilitate cement fixation but are associated with nerve damage, delayed recovery, acute pain and need for analgesics[89,90]. The RCTs we identified showed no effects of tourniquet use on long-term pain.

As shown in a previous review[91], there was no suggestion that CPM affects long-term pain. There was good quality evidence for a small benefit for reduced long-term pain in patients receiving walking training, anabolic steroid injection, electrical stimulation and supported discharge.

For some interventions a direct mechanism is clear, but for others, reasons for long-term impact are less obvious. This may explain why, for example, no studies evaluated DVT prophylaxis with long-term follow up excepting a small number reporting adverse events. However, treatments to

For peer review only - http://bmjopen.bmj.com/site/about/guidelines.xhtml

prevent symptomatic DVTs which occur in about 1% of treated patients[92] also reduce the incidence of asymptomatic DVT observed in about 28% of treated patients[93] and this may have long-term benefits. Conversely, new anticoagulants are associated with bleeding[94], which may increase the risk of wound complications[95] and joint infection[96] which are associated with long-term pain[97,98]. More peri-operative interventions with no information on long-term pain outcomes from RCTs are shown in Figure 1.

Our study is limited by the lack of meta-analysis which was not appropriate due to intervention and outcome heterogeneity. In the context of perioperative pain management, this was noted previously[84]. Our approach to assessing the evidence was a narrative synthesis of studies with low risk of bias. While this may seem overly restrictive, Cochrane risk of bias assessment allows us to screen out studies with important issues that may affect the validity of results. The main potential source of bias was incomplete outcome assessment. Although studies with long-term follow up are naturally at higher risk of missing data, we maintained a standard in this domain as it is recognised that research participants who do not complete follow up assessments differ in outcomes from those with follow up data and their inclusion could change the interpretation of results[99].

Another limitation is that pain assessed with questionnaires does not take into account the effect of pain medications and assistive aids. About 58% of women and 40% of men report taking pain medications after TKR because of pain in the operated knee[100] and we must recognise that pain levels at follow up without this treatment might be considerably higher. Even with treatment, around 20% of patients report chronic pain after TKR[10] and in the context of a blinded RCT we should expect to be able to identify effects of peri-operative treatments.

We summarised p-values to assess the strength of evidence but, as statistically strong evidence may not reflect clinically important results[101], where possible we also compared effect sizes with MCIDs. Our review considered a diverse range of interventions at a specific time in the TKR pathway and, as we were unable to make clinical practice recommendations, we did not adopt the GRADE system[102] for this review.

An alternative approach to the prevention of chronic pain after TKR is the individualisation of care based on pain phenotype, genetic, psychosocial and other factors[103]. An example of this might be the peri-operative treatment only of individuals with neuropathic pain with pregabalin, as opposed to the non-stratified provision in the RCT of Buvanendran and colleagues[53]. In an RCT with pregabalin provided to patients with painful HIV-neuropathy, while no overall benefit was seen, a group with hyperalgesia responded to pregabalin treatment[104].

For peer review only - http://bmjopen.bmj.com/site/about/guidelines.xhtml

Our systematic review of peri-operative interventions brings together evidence on interventions in the peri-operative phase of the TKR pathway. There was good quality evidence for some interventions of a small benefit for reduced long-term pain, and whilst not supportive of the inclusion of specific interventions in clinical practice, there are clearly areas that merit research. High quality studies assessing long-term pain after peri-operative interventions are feasible and necessary to ensure that patients with osteoarthritis achieve good long-term outcomes after TKR.

ACKNOWLEDGEMENT

We thank Dr Mario Moric for conducting additional analyses on the study of Buvanendran and colleagues.

AUTHOR CONTRIBUTIONS

All authors, ADB, JD, RG-H and AWB, contributed to the conception and design of the study. ADB, JD and VW undertook the systematic review. ADB and JD carried out the risk of bias assessments. ADB drafted the article with revisions by JD, VW, RG-H and AWB. All authors approved the final version for publication.

ROLE OF THE FUNDING SOURCE

This article presents independent research funded by the National Institute for Health Research (NIHR) under its Programme Grants for Applied Research programme (RP-PG-0613-20001). This study was supported by the NIHR Biomedical Research Centre at University Hospitals Bristol NHS Foundation Trust and the University of Bristol. The views expressed in this publication are those of the authors and not necessarily those of the NHS, the National Institute for Health Research or the Department of Health and Social Care.

COMPETING INTERESTS STATEMENT

The authors report no competing interests.

DATA STATEMENT

All data relevant to the study are included in the article or uploaded as supplementary information.

Legend

For peer review only

For peer review only - http://bmjopen.bmj.com/site/about/guidelines.xhtml

Figure 1. Systematic review flow diagram

REFERENCES

1. Weinstein AM, Rome BN, Reichmann WM, et al. Estimating the burden of total knee replacement in the United States. *J Bone Joint Surg (Am)* 2013;95:385-92.
2. National Joint Registry for England, Wales, Northern Ireland and the Isle of Man: 15th Annual Report. Hemel Hempstead: NJR Centre 2018.
3. Scottish Arthroplasty Project. Annual report 2018. Edinburgh: NHS National Services Scotland; 2018.
4. Maradit Kremers H, Larson DR, Crowson CS, et al. Prevalence of total hip and knee replacement in the United States. *J Bone Joint Surg (Am)* 2015;97:1386-97.
5. Nilsdotter AK, Toksvig-Larsen S, Roos EM. Knee arthroplasty: are patients' expectations fulfilled? A prospective study of pain and function in 102 patients with 5-year follow-up. *Acta Orthop* 2009;80:55-61.
6. Scott CE, Howie CR, MacDonald D, et al. Predicting dissatisfaction following total knee replacement: a prospective study of 1217 patients. *J Bone Joint Surg (Br)* 2010;92:1253-8.
7. Bachmeier CJM, March LM, Cross MJ, et al. A comparison of outcomes in osteoarthritis patients undergoing total hip and knee replacement surgery. *Osteoarthritis Cart* 2001;9:137-46.
8. Dailiana ZH, Papakostidou I, Varitimidis S, et al. Patient-reported quality of life after primary major joint arthroplasty: a prospective comparison of hip and knee arthroplasty. *BMC Musculoskelet Disord* 2015;16:366.
9. Werner MU, Kongsgaard UE. Defining persistent post-surgical pain: is an update required? *Br J Anaesth* 2014;113:1-4.
10. Blom AW, Artz N, Beswick AD, et al. Improving patients' experience and outcome of total joint replacement: The RESTORE programme. *Programme Grants Appl Res* 2016;4.
11. Lundblad H, Kreicbergs A, Jansson KA. Prediction of persistent pain after total knee replacement for osteoarthritis. *J Bone Joint Surg (Br)* 2008;90-B:166-71.
12. Wylde V, Sayers A, Odutola A, et al. Central sensitization as a determinant of patients' benefit from total hip and knee replacement. *Eur J Pain* 2017;21:357-65.
13. Mandalia V, Eyres K, Schranz P, et al. Evaluation of patients with a painful total knee replacement. *J Bone Joint Surg (Br)* 2008;90:265-71.
14. Hofmann S, Seitlinger G, Djahani O, et al. The painful knee after TKA: a diagnostic algorithm for failure analysis. *Knee Surg Sports Traumatol Arthrosc* 2011;19:1442-52.

For peer review only - http://bmjopen.bmj.com/site/about/guidelines.xhtml

15. Toms AD, Mandalia V, Haigh R, et al. The management of patients with painful total knee replacement. *J Bone Joint Surg (Br)* 2009;91:143-50.
16. Wylde V, Trela-Larsen L, Whitehouse MR, et al. Preoperative psychosocial risk factors for poor outcomes at 1 and 5 years after total knee replacement. *Acta Orthop* 2017;88:530-6.
17. Burns LC, Ritvo SE, Ferguson MK, et al. Pain catastrophizing as a risk factor for chronic pain after total knee arthroplasty: A systematic review. *J Pain Res* 2015;8:21-32.
18. Edwards RR, Haythornthwaite JA, Smith MT, et al. Catastrophizing and depressive symptoms as prospective predictors of outcomes following total knee replacement. *Pain Res Manag* 2009;14:307-11.
19. Wylde V, Beswick AD, Dennis J, et al. Post-operative patient-related risk factors for chronic pain after total knee replacement: a systematic review. *BMJ Open* 2017;7:e018105.
20. Lewis GN, Rice DA, McNair PJ, et al. Predictors of persistent pain after total knee arthroplasty: a systematic review and meta-analysis. *Br J Anaesth* 2015;114:551-61.
21. Wang L, Lee M, Zhang Z, et al. Does preoperative rehabilitation for patients planning to undergo joint replacement surgery improve outcomes? A systematic review and meta-analysis of randomised controlled trials. *BMJ Open* 2016;6:e009857.
22. Bay S, Kuster L, McLean N, et al. A systematic review of psychological interventions in total hip and knee arthroplasty. *BMC Musculoskelet Disord* 2018;19:201.
23. Riddle DL, Keefe FJ, Ang DC, et al. Pain coping skills training for patients who catastrophize about pain prior to knee arthroplasty: A multisite randomized clinical trial. *J Bone Joint Surg (Am)* 2019;101:218-27.
24. Kehlet H, Jensen TS, Woolf CJ. Persistent postsurgical pain: risk factors and prevention. *Lancet* 2006;367:1618-25.
25. Haroutiunian S, Nikolajsen L, Finnerup NB, et al. The neuropathic component in persistent postsurgical pain: A systematic literature review. *Pain* 2013;154:95-102.
26. Ashrani AA, Heit JA. Incidence and cost burden of post-thrombotic syndrome. *J Thromb Thrombolysis* 2009;28:465-76.
27. Dreyer HC. Tourniquet use during knee replacement surgery may contribute to muscle atrophy in older adults. *Exerc Sport Sci Rev* 2016;44:61-70.
28. Kindsfater K, Scott R. Recurrent hemarthrosis after total knee arthroplasty. *J Arthroplasty* 1995;10:S52-S5.
29. Epstein NE. A review article on the benefits of early mobilization following spinal surgery and other medical/surgical procedures. *Surg Neurol Int* 2014;5:S66-73.

For peer review only - http://bmjopen.bmj.com/site/about/guidelines.xhtml

30. Moher D, Liberati A, Tetzlaff J, et al. Preferred reporting items for systematic reviews and meta-analyses: the PRISMA statement. *J Clin Epidemiol* 2009;62:1006-12.

31. Marques EM, Jones HE, Elvers KT, et al. Local anaesthetic infiltration for peri-operative pain control in total hip and knee replacement: systematic review and meta-analyses of short- and long-term effectiveness. *BMC Musculoskelet Disord* 2014;15:220.

32. Higgins JPT, Green S. Cochrane Handbook for Systematic Reviews of Interventions Version 5.1.0 [Updated March 2011]: The Cochrane Collaboration; 2011. Available from: http://www.cochrane-handbook.org.

33. Sterne JA, Davey Smith G. Sifting the evidence-what's wrong with significance tests? *BMJ* 2001;322:226-31.

34. Albrecht E, Morfey D, Chan V, et al. Single-injection or continuous femoral nerve block for total knee arthroplasty? *Clin Orthop Relat Res* 2014;472:1384-93.

35. Choy WS, Lee SK, Kim KJ, et al. Two continuous femoral nerve block strategies after TKA. *Knee Surg Sports Traumatol Arthrosc* 2011;19:1901-8.

36. Fan L, Yu X, Zan P, et al. Comparison of local infiltration analgesia with femoral nerve block for total knee arthroplasty: A prospective, randomized clinical trial. *J Arthroplasty* 2016;31:1361-5.

37. Gao WL, Li H, Liu BQ, et al. Analgesic effect of femoral and sciatic nerve block under multimodal analgesia in total knee arthroplasty. *Chinese Journal of Tissue Engineering Research* 2017;21:2966-72.

38. Macrinici GI, Murphy C, Christman L, et al. Prospective, double-blind, randomized study to evaluate single-injection adductor canal nerve block versus femoral nerve block: postoperative functional outcomes after total knee arthroplasty. *Reg Anesth Pain Med* 2017;42:10-6.

39. Nader A, Kendall MC, Wixson RL, et al. A randomized trial of epidural analgesia followed by continuous femoral analgesia compared with oral opioid analgesia on short- and long-term functional recovery after total knee replacement. *Pain Med* 2012;13:937-47.

40. Peng L, Ren L, Qin P, et al. Continuous femoral nerve block versus intravenous patient controlled analgesia for knee mobility and long-term pain in patients receiving total knee replacement: a randomized controlled trial. *Evid Based Complement Alternat Med* 2014;2014:569107.

41. Reinhardt KR, Duggal S, Umunna BP, et al. Intraarticular analgesia versus epidural plus femoral nerve block after TKA: A randomized, double-blind trial. *Clin Orthop Relat Res* 2014;472:1400-8.

For peer review only - http://bmjopen.bmj.com/site/about/guidelines.xhtml

42. Wegener JT, van Ooij B, van Dijk CN, et al. Long-term pain and functional disability after total knee arthroplasty with and without single-injection or continuous sciatic nerve block in addition to continuous femoral nerve block: a prospective, 1-year follow-up of a randomized controlled trial. *Reg Anesth Pain Med* 2013;38:58-63.

43. Widmer BJ, Scholes CJ, Pattullo GG, et al. Is femoral nerve block necessary during total knee arthroplasty?: a randomized controlled trial. *J Arthroplasty* 2012;27:1800-5.

44. Wu JWS, Wong YC. Elective unilateral total knee replacement using continuous femoral nerve blockade versus conventional patient-controlled analgesia: perioperative patient management based on a multidisciplinary pathway. *Hong Kong Med J* 2014;20:45-51.

45. McDonald DA, Deakin AH, Ellis BM, et al. The technique of delivery of peri-operative analgesia does not affect the rehabilitation or outcomes following total knee arthroplasty. *Bone Joint J* 2016;98-B:1189-96.

46. Motififard M, Omidian A, Badiei S. Pre-emptive injection of peri-articular-multimodal drug for post-operative pain management in total knee arthroplasty: a double-blind randomized clinical trial. *Int Orthop* 2017;41:939-47.

47. Niemeläinen M, Kalliovalkama J, Aho A, et al. Single periarticular local infiltration analgesia reduces opiate consumption until 48 hours after total knee arthroplasty. A randomized placebo-controlled trial involving 56 patients. *Acta Orthop* 2014;85:614-9.

48. Seah VWT, Chin PL, Chia SL, et al. Single-dose periarticular steroid infiltration for pain management in total knee arthroplasty: a prospective, double-blind, randomised controlled trial. *Singapore Med J* 2011;52:19-23.

49. Williams D, Petruccelli D, Paul J, et al. Continuous infusion of bupivacaine following total knee arthroplasty: a randomized control trial pilot study. *J Arthroplasty* 2013;28:479-84.

50. Wylde V, Lenguerrand E, Gooberman-Hill R, et al. Effect of local anaesthetic infiltration on chronic postsurgical pain after total hip and knee replacement: the APEX randomised controlled trials. *Pain* 2015;156:1161-70.

51. Meunier A, Lisander B, Good L. Effects of celecoxib on blood loss, pain, and recovery of function after total knee replacement: a randomized placebo-controlled trial. *Acta Orthop* 2007;78:661-7.

52. Aveline C, Roux AL, Hetet HL, et al. Pain and recovery after total knee arthroplasty: a 12-month follow-up after a prospective randomized study evaluating Nefopam and Ketamine for early rehabilitation. *Clin J Pain* 2014;30:749-54.

For peer review only - http://bmjopen.bmj.com/site/about/guidelines.xhtml

53. Buvanendran A, Kroin JS, Della Valle CJ, et al. Perioperative oral pregabalin reduces chronic pain after total knee arthroplasty: a prospective, randomized, controlled trial. *Anesth Analg* 2010;110:199-207.

54. Ejaz A, Laursen AC, Kappel A, et al. Faster recovery without the use of a tourniquet in total knee arthroplasty. *Acta Orthop* 2014;85:422-6.

55. Huang Z, Xie X, Li L, et al. Intravenous and topical tranexamic acid alone are superior to tourniquet use for primary total knee arthroplasty: A prospective, randomized controlled trial. *J Bone Joint Surg (Am)* 2017;99:2053-61.

56. Liu D, Graham D, Gillies K, et al. Effects of tourniquet use on quadriceps function and pain in total knee arthroplasty. *Knee Surg Relat Res* 2014;26:207-13.

57. Mittal R, Ko V, Adie S, et al. Tourniquet application only during cement fixation in total knee arthroplasty: a double-blind, randomized controlled trial. *ANZ J Surg* 2012;82:428-33.

58. Zhang Y, Li D, Liu P, et al. Effects of different methods of using pneumatic tourniquet in patients undergoing total knee arthroplasty: a randomized control trial. *Ir Jr Med Sci* 2017;186:953-9.

59. Brock T, Sprowson A, Muller S, et al. STICKS study - Short-sTretch Inelastic Compression bandage in Knee Swelling following total knee arthroplasty - a feasibility study. *Trials* 2017;18:6.

60. Hourlier H, Reina N, Fennema P. Single dose intravenous tranexamic acid as effective as continuous infusion in primary total knee arthroplasty: a randomised clinical trial. *Arch Orthop Trauma Surg* 2015;135:465-71.

61. Kim TK, Chang CB, Kang YG, et al. Clinical value of tranexamic acid in unilateral and simultaneous bilateral TKAs under a contemporary blood-saving protocol: a randomized controlled trial. *Knee Surg Sports Traumatol Arthrosc* 2014;22:1870-8.

62. Kusuma SK, Sheridan KC, Wasielewski RC. Use of bovine thrombin to reduce blood loss in primary total knee arthroplasty: a controlled randomized trial. *J Arthroplasty* 2013;28:1278-81.

63. Napier RJ, Bennett D, McConway J, et al. The influence of immediate knee flexion on blood loss and other parameters following total knee replacement. *Bone Joint J* 2014;96-B:201-9.

64. Sa-Ngasoongsong P, Channoom T, Kawinwonggowit V, et al. Postoperative blood loss reduction in computer-assisted surgery total knee replacement by low dose intra-articular tranexamic acid injection together with 2-hour clamp drain: a prospective triple-blinded randomized controlled trial. *Orthop Rev* 2011;3:e12.

For peer review only

For peer review only - http://bmjopen.bmj.com/site/about/guidelines.xhtml

65. Sa-Ngasoongsong P, Wongsak S, Chanplakorn P, et al. Efficacy of low-dose intra-articular tranexamic acid in total knee replacement; a prospective triple-blinded randomized controlled trial. *BMC Musculoskelet Disord* 2013;14:340.
66. Ledin H, Good L, Aspenberg P. Denosumab reduces early migration in total knee replacement. *Acta Orthop* 2017;88:255-8.
67. Bennett LA, Brearley SC, Hart JAL, et al. A comparison of 2 continuous passive motion protocols after total knee arthroplasty: a controlled and randomized study. *J Arthroplasty* 2005;20:225-33.
68. Ersözlü S, Sahin O, Ozgür A, et al. The effects of two different continuous passive motion protocols on knee range of motion after total knee arthroplasty: a prospective analysis. *Acta Orthop Traumato* 2009;43:412-8.
69. Avramidis K, Karachalios T, Popotonasios K, et al. Does electric stimulation of the vastus medialis muscle influence rehabilitation after total knee replacement? *Orthopedics* 2011;34:175.
70. Moretti B, Notarnicola A, Moretti L, et al. I-ONE therapy in patients undergoing total knee arthroplasty: a prospective, randomized and controlled study. *BMC Musculoskelet Disord* 2012;13:88.
71. Li L, Wang Z, Yin MH, et al. Effect of early gait training on the functional rehabilitation after total knee arthroplasty. *Chinese Journal of Tissue Engineering Research* 2017;21:4288-93.
72. Liebs T, Herzberg W, Rüther W, et al. Multicenter randomized controlled trial comparing early versus late aquatic therapy after total hip or knee arthroplasty. *Arch Phys Med Rehabil* 2012;93:192-9.
73. Mahomed NN, Davis AM, Hawker G, et al. Inpatient compared with home-based rehabilitation following primary unilateral total hip or knee replacement: a randomized controlled trial. *J Bone Joint Surg (Am)* 2008;90:1673-80.
74. Wang S, Xia J, Wei Y, et al. Effect of the knee position during wound closure after total knee arthroplasty on early knee function recovery. *J Orthop Surg Res* 2014;9:79.
75. Kong CG, Kim GH, Kim DW, et al. The effect of topical scar treatment on postoperative scar pain and pruritus after total knee arthroplasty. *Arch Orthop Trauma Surg* 2014;134:555-9.
76. Hohmann E, Tetsworth K, Hohmann S, et al. Anabolic steroids after total knee arthroplasty. A double blinded prospective pilot study. *J Orthop Surg Res* 2010;5:93.
77. Ehrich EW, Davies GM, Watson DJ, et al. Minimal perceptible clinical improvement with the Western Ontario and McMaster Universities osteoarthritis index questionnaire and global assessments in patients with osteoarthritis. *J Rheumatol* 2000;27:2635-41.

For peer review only

For peer review only - http://bmjopen.bmj.com/site/about/guidelines.xhtml

78. Beard DJ, Harris K, Dawson J, et al. Meaningful changes for the Oxford hip and knee scores after joint replacement surgery. *J Clin Epidemiol* 2015;68:73-9.

79. Lee WC, Kwan YH, Chong HC, et al. The minimal clinically important difference for Knee Society Clinical Rating System after total knee arthroplasty for primary osteoarthritis. *Knee Surg Sports Traumatol Arthrosc* 2017;25:3354-9.

80. Salaffi F, Stancati A, Silvestri CA, et al. Minimal clinically important changes in chronic musculoskeletal pain intensity measured on a numerical rating scale. *Eur J Pain* 2004;8:283-91.

81. Singh JA, Schleck C, Harmsen WS, et al. Validation of the hospital for special surgery knee questionnaire: Convergent validity, responsiveness and sensitivity to change. ACR/ARHP; San Diego, California2013. p. 1926.

82. Escobar A, Quintana JM, Bilbao A, et al. Responsiveness and clinically important differences for the WOMAC and SF-36 after total knee replacement. *Osteoarthritis Cart* 2007;15:273-80.

83. Danoff JR, Goel R, Sutton R, et al. How much pain is significant? Defining the minimal clinically important difference for the visual analog scale for pain after total joint arthroplasty. *J Arthroplasty* 2018;33:S71-S5.

84. Andersen LO, Kehlet H. Analgesic efficacy of local infiltration analgesia in hip and knee arthroplasty: a systematic review. *Br J Anaesth* 2014;113:360-74.

85. Chan E-Y, Fransen M, Parker DA, et al. Femoral nerve blocks for acute postoperative pain after knee replacement surgery. *Cochrane Database Syst Rev* 2014;5:CD009941.

86. Alshryda S, Sarda P, Sukeik M, et al. Tranexamic acid in total knee replacement: a systematic review and meta-analysis. *J Bone Joint Surg (Br)* 2011;93:1577-85.

87. Poeran J, Rasul R, Suzuki S, et al. Tranexamic acid use and postoperative outcomes in patients undergoing total hip or knee arthroplasty in the United States: retrospective analysis of effectiveness and safety. *BMJ* 2014;349.

88. Hallstrom B, Singal B, Cowen ME, et al. The Michigan experience with safety and effectiveness of tranexamic acid use in hip and knee arthroplasty. *J Bone Joint Surg (Am)* 2016;98:1646-55.

89. Noordin S, McEwen JA, Kragh JF, Jr., et al. Surgical tourniquets in orthopaedics. *J Bone Joint Surg (Am)* 2009;91:2958-67.

90. Horlocker TT, Hebl JR, Gali B, et al. Anesthetic, patient, and surgical risk factors for neurologic complications after prolonged total tourniquet time during total knee arthroplasty. *Anesth Analg* 2006;102:950-5.

For peer review only

For peer review only - http://bmjopen.bmj.com/site/about/guidelines.xhtml

91. Harvey LA, Brosseau L, Herbert RD. Continuous passive motion following total knee arthroplasty in people with arthritis. *Cochrane Database Syst Rev* 2014;2:CD004260.

92. Januel J-M, Chen G, Ruffieux C, et al. Symptomatic in-hospital deep vein thrombosis and pulmonary embolism following hip and knee arthroplasty among patients receiving recommended prophylaxis: a systematic review. *JAMA* 2012;307:294-303.

93. Schellong SM, Beyer J, Kakkar AK, et al. Ultrasound screening for asymptomatic deep vein thrombosis after major orthopaedic surgery: The VENUS study. *J Thromb Haemost* 2007;5:1431-7.

94. Gómez-Outes A, Terleira-Fernández AI, Suárez-Gea ML, et al. Dabigatran, rivaroxaban, or apixaban versus enoxaparin for thromboprophylaxis after total hip or knee replacement: systematic review, meta-analysis, and indirect treatment comparisons. *BMJ* 2012;344:e3675.

95. Freedman KB, Brookenthal KR, Fitzgerald RH, Jr., et al. A meta-analysis of thromboembolic prophylaxis following elective total hip arthroplasty. *J Bone Joint Surg (Am)* 2000;82-a:929-38.

96. Parvizi J, Ghanem E, Joshi A, et al. Does "excessive" anticoagulation predispose to periprosthetic infection? *J Arthroplasty* 2007;22:24-8.

97. Adelani MA, Johnson SR, Keeney JA, et al. Clinical outcomes following re-admission for non-infectious wound complications after primary total knee replacement. *Bone Joint J* 2014;96-b:619-21.

98. Cahill JL, Shadbolt B, Scarvell JM, et al. Quality of life after infection in total joint replacement. *J Orthop Surg* 2008;16:58-65.

99. Akl EA, Briel M, You JJ, et al. Potential impact on estimated treatment effects of information lost to follow-up in randomised controlled trials (LOST-IT): systematic review. *BMJ* 2012;344:e2809.

100. Hawker G, Wright J, Coyte P, et al. Health-related quality of life after knee replacement - Results of the Knee Replacement Patient Outcomes Research Team Study. *J Bone Joint Surg (Am)* 1998;80A:163-73.

101. Leopold SS, Porcher R. Editorial: threshold p values in orthopaedic research—we know the problem. What is the solution? *Clin Orthop Relat Res* 2018;476:1689-91.

102. Guyatt GH, Oxman AD, Kunz R, et al. Going from evidence to recommendations. *BMJ* 2008;336:1049-51.

103. Lavand'homme P, Thienpont E. Pain after total knee arthroplasty: a narrative review focusing on the stratification of patients at risk for persistent pain. *Bone Joint J* 2015;97-B:45-8.

For peer review only

For peer review only - http://bmjopen.bmj.com/site/about/guidelines.xhtml

104. Simpson DM, Schifitto G, Clifford DB, et al. Pregabalin for painful HIV neuropathy: a randomized, double-blind, placebo-controlled trial. *Neurology* 2010;74:413-20.

For peer review only

For peer review only - http://bmjopen.bmj.com/site/about/guidelines.xhtml

Identification

Articles identified in searches of *The Cochrane Library*, MEDLINE, Embase, PsycINFO and CINAHL from inception to February 2018 (n = 9697)

Screening

Articles screened (n = 9697)

No relevance (n = 7364)

Eligibility

Potentially relevant (n = 2333)

Intervention	N	In: low risk of bias	No pain/ score outcome	High/ unclear risk of bias	No long-term follow up	Abstract only	Additional publication	Protocol	Review	Retracted
Adenosine triphosphate	2	0	0	0	1	0	0	0	1	0
Alternative medicine	4	0	0	0	4	0	0	0	0	0
Anabolic steroids	2	1	0	0	0	0	0	0	1	0
Antibiotic prophylaxis	43	0	16	0	13	0	0	1	13	0
Assistive devices	2	0	0	0	2	0	0	0	0	0
Bisphosphonates	17	0	6	0	0	0	2	0	9	0
Blood management	355	7	10	1	209	0	0	4	124	0
Brain stimulation	3	0	0	0	3	0	0	0	0	0
Calcium supplement	1	0	0	0	1	0	0	0	0	0
Cardiovascular drugs	4	0	0	0	2	0	0	0	2	0
Chinese medicine	2	0	0	0	2	0	0	0	0	0
Cold therapy	30	0	0	1	24	0	0	0	5	0
Colloids and crystalloids	1	0	0	0	1	0	0	0	0	0
Comorbidity management	1	0	0	0	1	0	0	0	0	0
Compression	8	1	0	0	6	0	0	1	0	0
Constipation treatment	2	0	0	0	2	0	0	0	0	0
Continuous passive motion	56	2	8	7	23	1	0	1	14	0
Creatine monohydrate	1	0	0	0	1	0	0	0	0	0
Delirium prevention	4	0	0	0	3	0	0	0	1	0
Denusomab	1	1	0	0	0	0	0	0	0	0
Dexmedetomidine	1	0	0	0	1	0	0	0	0	0
Deep vein thrombosis prophylaxis	474	0	5	0	143	0	4	8	314	0
Electrical stimulation	37	2	0	3	20	0	2	0	10	0
Glucocorticoid	2	0	0	0	0	0	0	0	2	0
Glucose infusion	1	0	0	0	1	0	0	0	0	0
Guided imagery	5	0	0	1	4	0	0	0	0	0
Iron	7	0	0	0	6	0	0	0	1	0
Laser therapy	1	0	0	0	1	0	0	0	0	0
Methylprednisolone	3	0	0	0	3	0	0	0	0	0
Music therapy	9	0	0	0	9	0	0	0	0	0
Nausea prevention	11	0	0	0	9	0	2	0	0	0
Nutritional supplements	4	0	0	0	4	0	0	0	0	0
Pain management	987	20	5	12	711	1	20	9	207	2
Physiological	26	0	0	0	23	0	0	1	2	0
Platelet rich plasma	12	0	0	1	6	0	0	0	5	0
Rehabilitation	67	4	0	2	43	0	0	1	17	0
Remote ischaemic pre-conditioning	5	0	0	0	5	0	0	0	0	0
Sleep treatment	3	0	0	0	2	0	1	0	0	0
Teriparatide	1	0	1	0	0	0	0	0	0	0
Therapy dogs	1	0	0	0	1	0	0	0	0	0
Tourniquet use	100	5	3	3	67	0	2	1	19	0
Trigger point needling	1	0	0	1	0	0	0	0	0	0
Warming	19	0	0	0	16	0	0	0	3	0
Wound management	17	1	0	0	12	0	0	1	3	0
**Total**	**2333**	**44**	**54**	**32**	**1385**	**2**	**33**	**28**	**753**	**2**

For peer review only

Reporting checklist for systematic review and meta-analysis.

Based on the PRISMA guidelines.

Instructions to authors

Complete this checklist by entering the page numbers from your manuscript where readers will find each of the items listed below.

Your article may not currently address all the items on the checklist. Please modify your text to include the missing information. If you are certain that an item does not apply, please write "n/a" and provide a short explanation.

Upload your completed checklist as an extra file when you submit to a journal.

In your methods section, say that you used the PRISMA reporting guidelines, and cite them as:

Moher D, Liberati A, Tetzlaff J, Altman DG, The PRISMA Group. Preferred Reporting Items for Systematic Reviews and Meta-Analyses: The PRISMA Statement

		Reporting Item	Page Number
	#1	Identify the report as a systematic review, meta-analysis, or both.	1
Structured summary	#2	Provide a structured summary including, as applicable: background; objectives; data sources; study eligibility criteria, participants, and interventions; study appraisal and synthesis methods; results; limitations; conclusions and implications of key findings; systematic review registration number	2-3,5
Rationale	#3	Describe the rationale for the review in the context of what is already known.	4-5
Objectives	#4	Provide an explicit statement of questions being addressed with reference to participants, interventions, comparisons, outcomes, and study design (PICOS).	5-6
Protocol and registration	#5	Indicate if a review protocol exists, if and where it can be accessed (e.g., Web address) and, if available, provide registration information including the registration number.	5

For peer review only

Eligibility criteria	#6	Specify study characteristics (e.g., PICOS, length of follow-up) and report characteristics (e.g., years considered, language, publication status) used as criteria for eligibility, giving rational	5-6
Information sources	#7	Describe all information sources in the search (e.g., databases with dates of coverage, contact with study authors to identify additional studies) and date last searched.	6
Search	#8	Present full electronic search strategy for at least one database, including any limits used, such that it could be repeated.	See note 1
Study selection	#9	State the process for selecting studies (i.e., for screening, for determining eligibility, for inclusion in the systematic review, and, if applicable, for inclusion in the meta-analysis).	5,6
Data collection process	#10	Describe the method of data extraction from reports (e.g., piloted forms, independently by two reviewers) and any processes for obtaining and confirming data from investigators.	6
Data items	#11	List and define all variables for which data were sought (e.g., PICOS, funding sources), and any assumptions and simplifications made.	5/6
Risk of bias in individual studies	#12	Describe methods used for assessing risk of bias in individual studies (including specification of whether this was done at the study or outcome level, or both), and how this information is to be used in any data synthesis.	6
Summary measures	#13	State the principal summary measures (e.g., risk ratio, difference in means).	7
Planned methods of analyis	#14	Describe the methods of handling data and combining results of studies, if done, including measures of consistency (e.g., I2) for each meta-analysis.	7
Risk of bias across studies	#15	Specify any assessment of risk of bias that may affect the cumulative evidence (e.g., publication bias, selective reporting within studies).	6
Additional analyses	#16	Describe methods of additional analyses (e.g., sensitivity or subgroup analyses, meta-regression), if done, indicating which were pre-specified.	7

For peer review only

Study selection	#17	Give numbers of studies screened, assessed for eligibility, and included in the review, with reasons for exclusions at each stage, ideally with a flow diagram.	See note 2
Study characteristics	#18	For each study, present characteristics for which data were extracted (e.g., study size, PICOS, follow-up period) and provide the citation.	See note 3
Risk of bias within studies	#19	Present data on risk of bias of each study and, if available, any outcome-level assessment (see Item 12).	See note 4
Results of individual studies	#20	For all outcomes considered (benefits and harms), present, for each study: (a) simple summary data for each intervention group and (b) effect estimates and confidence intervals, ideally with a forest plot.	See note 5
Synthesis of results	#21	Present the main results of the review. If meta-analyses are done, include for each, confidence intervals and measures of consistency.	16-23
Risk of bias across studies	#22	Present results of any assessment of risk of bias across studies (see Item 15).	See note 6
Additional analysis	#23	Give results of additional analyses, if done (e.g., sensitivity or subgroup analyses, meta-regression [see Item 16]).	16-23
Summary of Evidence	#24	Summarize the main findings, including the strength of evidence for each main outcome; consider their relevance to key groups (e.g., health care providers, users, and policy makers	16-23
Limitations	#25	Discuss limitations at study and outcome level (e.g., risk of bias), and at review level (e.g., incomplete retrieval of identified research, reporting bias).	24-25
Conclusions	#26	Provide a general interpretation of the results in the context of other evidence, and implications for future research.	23-25
Funding	#27	Describe sources of funding or other support (e.g., supply of data) for the systematic review; role of funders for the systematic review.	26

Author notes

For peer review only

1. 5, Supplemetary material

2. 7, Figure 1

3. 8-15, Table1, Supplementary material

4. 7, Supplementary material

5. 8-15, Table1, Supplementary material

6. 8-15, Supplementary material

The PRISMA checklist is distributed under the terms of the Creative Commons Attribution License CC-BY. This checklist was completed on 21. November 2018 using http://www.goodreports.org/, a tool made by the EQUATOR Network in collaboration with Penelope.ai

For peer review only

Supplementary material. Search strategy as applied in MEDLINE on Ovid

1 randomized controlled trial/ or randomized controlled trial.pt.

2 controlled clinical trial.pt.

3 randomized.ab.

4 placebo.ab.

5 randomly.ab

6 trial.ab

7 randomised.tw

8 1 or 2 or 3 or 4 or 5 or 6 or 7

9 review/

10 'systematic review$'.mp

11 9 or 10

12 8 or 11

13 Arthroplasty, Replacement, Knee/

14 Knee Prosthesis/

15 (arthoplast$ adj3 knee$).mp. [mp=title, abstract, original title, name of substance word, subject heading word, keyword heading word, protocol supplementary concept word, rare disease supplementary concept word, unique identifier]

16 (knee$ adj3 replac$).mp. [mp=title, abstract, original title, name of substance word, subject heading word, keyword heading word, protocol supplementary concept word, rare disease supplementary concept word, unique identifier]

17 (knee adj3 implant$).mp. [mp=title, abstract, original title, name of substance word, subject heading word, keyword heading word, protocol supplementary concept word, rare disease supplementary concept word, unique identifier]

18 13 or 14 or 15 or 16 or 17

19 12 and 18

For peer review only - http://bmjopen.bmj.com/site/about/guidelines.xhtml

For peer review only

Supplementary material. All peri-operative interventions with long-term pain or score follow up

1. Pain management

Author<br>Country<br>Recruitment dates<br>Setting	Indication<br>Number randomised intervention; control<br>Age<br>% female	Common anaesthesia			Follow up<br>Losses to follow up intervention; control<br>Risk of bias issues<br>Key results
		Group 1 (intervention)	Group 2 (intervention)	Group C (control)	
FNB single vs No FNB					
Widmer et al. 2012[43]<br>Australia<br>Before 2012<br>2 surgeons	Elective primary unilateral TKR<br>27; 28<br>Median 72.1 (IQR 64.4, 76.5); 69.4 (63.4, 75.5)<br>44.4%; 44.4%	Premedication 1-3mg i.v. midazolam. Propofol induction and sevoflurane general anaesthetic.<br>LIA with 200mg ropivacaine and 0.5mg adrenaline in 100ml saline.<br>PCA 20µg fentanyl at 5-minute intervals on demand until morning POD2. Then, oral oxycodone SR 10mg every 12 hours. Daily COX II inhibitor and paracetamol 1g every 6 hours as tolerated. For breakthrough pain, 5-10mg oxycodone immediate release every 3 hours as needed.			1 year<br>No losses to follow up reported<br>Low risk of bias<br>WOMAC pain (high score favourable) at 1 year: FNB and LIA median 2.0 (IQR 0, 2.8); LIA no FNB 1.0 (0, 2.0). p=0.74<br>No adverse events occurred in either group
		Ultrasound guided FNB with 100mg ropivacaine in 30ml saline		Sham setup for FNB. No identification or injection of femoral sheath	
FNB single vs ONB vs Control					
Bergeron et al. 2009[105]<br>Canada<br>2005-2006<br>1 centre	Elective primary unilateral TKR<br>19; 20; 20<br>Mean 65.1 (SE 2.0); 72 (1.8); 67 (1.3)<br>79%; 80%; 75%	Intraoperative sedation with iv propofol at discretion of anaesthesiologist. Lumbar spinal anaesthesia with 12mg 0.5% bupivacaine.<br>Postoperative i.v. PCA with fentanyl 50µg/ml set to deliver 25µg every 5 min as needed.<br>Celecoxib 100mg and acetaminophen 650mg on arrival in recovery room and every 12 and 6 hrs respectively. Breakthrough medication with intramuscular ketorolac 10 mg every 4 hrs.			1 year<br>Overall 32 lost to follow up<br>High risk of bias: only 27/59 patients followed up due to resource limitations.<br>No difference in HSS pain at rest or during activity at 1 year between the study groups.

For peer review only - http://bmjopen.bmj.com/site/about/guidelines.xhtml

		FNB with stimulator. 20ml 0.5% bupivacaine with 1/200,000 epinephrine.	ONB with stimulator. 20ml 0.5% bupivacaine with 1/200,000 epinephrine.	No injection but inguinal area prepared, and sham block performed behind drapes.	No long-term complications attributable to anaesthetic regimen
FNB continuous low dose vs FNB continuous high dose vs No FNB					
Shum et al. 2009[106]<br>Singapore<br>Before 2009<br>1 hospital	Unilateral TKR for osteoarthritis<br>20 (17 received treatment); 20 (18 received treatment); 20<br>Mean 66.7 (SD 8.4); 65.4 (8.4); 67.8 (5.5)<br>88%; 72%; 80%	Spinal anaesthesia induced with 2-3ml hyperbaric 0.5% bupivacaine. Intraoperative sedation with midazolam in increments of 0.5mg.<br>Intravenous PCA morphine (1mg/ml, on-demand bolus doses of 1 mg with 5 minute lockout, maximum dose 8 mg/hr)			2 years<br>16.4% of patients who received intervention lost to follow up<br>Unclear risk of bias due to differences in OKS and weight at baseline, and limited methodological details.<br>No separate pain outcome but mean OKS slightly more favourable in group with no FNB, 18.2 (SD 3.7) compared with combined FNB groups, 19.8 (5.4) but this was not significant.<br>No complications attributable to use of FNB
		Low dose continuous FNB at conclusion of TKR with ropivacaine 0.15% (10 ml/hr in the first 24 hours, followed by 5ml/hr in the next 24 hours)	High dose continuous FNB at conclusion of TKR with ropivacaine 0.2% (10 ml/hr in the first 24 hours, followed by 5 ml/hr in the next 24 hours)	No FNB	
SNB injection vs SNB continuous vs control					
Wegener et al. 2013[42]<br>The Netherlands<br>2008-2010<br>1 centre	Unilateral TKR<br>29; 30; 30 (90 randomised)<br>Median 65 (range 43-81); 66 (43-83); 62 (50-79)<br>62%; 70%; 73%	Lorazepam 1mg 2 hours and acetaminophen 2g 1 hour before surgery. FNB with stimulating catheter: loading dose 20 ml levobupivacaine 0.375% and after 45 minutes a continuous infusion of levobupivacaine 0.125% 10 ml/hr. General anaesthesia induced with 3-5 µg/ml propofol infusion and remifentanil 0.5 µg/kg/min and maintained with 2-3 µg/ml at 0.1-0.25 µg/kg/min. Postoperatively, FNB changed to patient controlled FNB, 5ml bolus, 30-minute lockout; basal rate 6 ml/hr. i.v. morphine administered if needed. Postoperative analgesia with acetaminophen 1g 4 times daily. Diclofenac 50mg or tramadol 50mg 3 times daily. Tramadol 100mg before removal of nerve catheters. Morphine pain relief as required.			12 months<br>2;7;5 lost to follow up<br>Low risk of bias<br>Median WOMAC pain scores at 12 months: SNB injection 80 (range 25-100), SNB continuous 90 (55-100) and PCA only 90 (35-100), p=0.81. No difference between groups in VAS pain at rest (p=0.90) or during mobilisation (p=0.43).<br>No information on adverse events.

For peer review only - http://bmjopen.bmj.com/site/about/guidelines.xhtml

		Group Fs: SNB single injection. SNB loading dose of 20 ml levobupivacaine 0.375%.	Group FCS: SNB continuous infusion. SNB loading dose of 20 ml levobupivacaine 0.375%. Continuous infusion of levobupivacaine 0.125% 10 ml/hr started 45 mins after catheter placement. SNB maintained for 36 hours postoperatively (10 ml/hr).	Group F: No SNB. PCA via femoral nerve catheter	
**General anaesthesia vs FNB single vs FNB/ SNB single**					
Gao et al. 2017[37]<br>China<br>2014-2015<br>1 centre	Primary unilateral TKR for osteoarthritis<br>50; 50; 50<br>Mean 65.8 (SD 6.7); 66.4 (7.4); 67.6 (6.3)<br>81%; 80%; 76%	Pre-operative and post-operative celecoxib 0.2g twice daily.<br>100ml intra-operative LIA with ropivacaine 200mg and epinephrine 0.25 mg.			6 months<br>2; 1; 0<br>Low risk of bias<br>Mean HSS at 6 months: 87.1 (SD 6.9); 87.4 (7.3); 88.5 (6.7). No significant difference.<br>Nausea and vomiting: 4; 2; 1, urinary retention: 3; 1; 2.
		General anaesthesia	Ultrasound guided FNB 5g/l ropivacaine 20ml plus 0.1mg epinephrine	Ultrasound guided FNB 5g/l ropivacaine 20 ml plus 0.1mg epinephrine and SNB 5g/l ropivacaine 20ml plus 0.1mg epinephrine	
LIA no corticosteroid vs No LIA/ placebo					
Wylde et al. 2015 [50]<br>UK	Primary unilateral TKR for osteoarthritis	FNB with nerve stimulator and/ or ultrasound guidance (20ml 0.25% bupivacaine). Spinal or general anaesthetic. Intra-operative analgesia provided by titration of i.v. fentanyl initially and morphine if necessary. 1g intravenous			6 and 12 months<br>24;19 at 12 months (including those who did not receive treatment)

For peer review only

For peer review only - http://bmjopen.bmj.com/site/about/guidelines.xhtml

2009-2012<br>1 centre	157; 159 (143; 137 received treatment)<br>Mean 69.5 (SD 9.4); 68.7 (7.9)<br>52%; 54%	paracetamol 30 minutes before the end of operation. Immediately post-operative 400mg oral ibuprofen.<br>PCA with morphine 1mg/ml, 1 mg bolus dose and a 5-minute lock-out. If necessary morphine bolus up to 0.2mg/kg as rescue analgesia. During hospital stay, visit from pain specialist nurse. Oral or i.v. paracetamol every 6 hours and ibuprofen 400mg every 8 hours. When PCA no longer needed, oral codeine phosphate 30-60mg every 6 hours, tramadol 50-100mg every 6 hours and oramorph 10-20mg as rescue analgesia.	60ml intra-operative LIA with 0.25% bupivacaine and 1/200,000 adrenaline injected into the posterior capsule, medial and lateral capsule, fascia and muscle, and subcutaneous tissues.	No treatment other than standard care	Low risk of bias<br>At 12 months WOMAC pain score (0-100) in LIA group median 90 (IQR 30), Control 85 (35); ITT-CC linear regression coefficient 3.83 (95%CI -0.83, 8.49), p=0.107. At 6 months WOMAC pain score ITT-CC linear regression coefficient 4.10 (95%CI -0.22, 8.43), p=0.063. Mean differences lower than MCID of 8-9[77].<br>Superficial and deep wound infection rate in LIA group 3.2% and 1.9% in control group, p=0.500. No differences in serious adverse events between groups
Williams et al. 2013[49]<br>Canada<br>Before 2013<br>1 centre, 2 surgeons	Primary unilateral TKR for osteoarthritis<br>35; 32 (26; 25 received treatment)<br>Mean 66 (SD 9.7); 67 (12.5)<br>58%; 60%	Sedation with i.v. midazolam and propofol. Intraoperative LIA loading dose of 20ml 0.25% bupivacaine/ epinephrine injection, 10ml into medial and lateral subcutaneous tissue around the incision and 10ml intra-articular after closure. Infiltrate delivered by pain pump into lateral recess of intra-articular space. Spinal anaesthetic with 10-15 mg of 0.75% or 0.5% plain bupivacaine and 20µg fentanyl.<br>Postoperative morphine PCA. 7.5mg i.v ketorolac preoperatively plus 15mg every 6 hours postoperatively for 48 hours, then oral ketorolac 10mg every 6 hours for 2 days. Gabapentin 600mg given preoperatively plus 300mg twice daily for 48 hours postoperatively. Oxycodone 10mg twice daily for 48 hours postoperative. Oral paracetamol 650mg every 4 hours for 72 hours.	Infusion of 0.5% bupivacaine at 2ml/hr for 48 hrs	Infusion of saline at 2ml/hr for 48 hrs	6 and 12 months<br>3;1 of those who received treatment<br>Low risk of bias<br>Mean VAS pain score at 6 months 1.2 (SD 1.3); 1.2 (1.2). p=0.836. At 12 months 0.9 (1.2); 1.0 (1.1). p=0.767<br>No short-term differences in adverse events except control patients more likely to be drowsy at 48 hrs. Long-term adverse events not reported.
Niemeläinen et al. 2014[47]<br>Finland<br>2011-2012	Primary unilateral TKR for osteoarthritis<br>30; 30 (27; 29 received treatment)	Oral paracetamol 1g given 1 hour before surgery. Spinal anaesthesia with 15mg bupivacaine in 3ml.<br>After surgery oral paracetamol 1g every 6 hours and oral meloxicam (15mg) every 24 hours.<br>PCA with oxycodone 2mg, lock-out time 8 min.			12 months<br>1; 4<br>Low risk of bias<br>No pain measure separate from OKS. Weak evidence of more favourable

For peer review only - http://bmjopen.bmj.com/site/about/guidelines.xhtml

1 hospital	Mean 65 (SD 4.9); 64 (6.7)<br>56%; 48%	Rescue levobupivacaine medication through a lumbar epidural catheter		OKS (0-48) in the LIA group at 12 months, mean difference -2.7 (95% CI -5.48, 0.07). Difference lower than MCID of 4.0[78].<br>Infection: 0; 0. Severe pain treated with epidural analgesia: 0; 3. Nausea: 1; 1
		Intra-operative periarticular LIA of 100ml saline with levobupivacaine (150mg) mixed with ketorolac (30mg) and adrenaline (0.5mg).	Intra-operative periarticular LIA of 100ml saline	
Motifard et al. 2017[46]<br>Iran<br>2014-2015<br>1 hospital	Primary unilateral TKR for osteoarthritis<br>60; 60<br>Mean 66.4 (6.4); 64.5 (6.0)<br>86.0%; 94.3%	Spinal anaesthesia.<br>No FNB or SNB.<br>Pain medication provided as required after surgery: meloxicam (15 mg daily), celecoxib (400 mg daily), acetaminophen (1g every 8 hours), tramadol (50 mg every 8 hours), ketorolac (30 mg slow IV every 8 hours, with a 4-dose max), and morphine (5–10 mg slow IV if needed)		6 months<br>3; 7<br>Low risk of bias<br>No separate pain measure. Weak evidence for improved KSS (0-200) in LIA group at 6 months, mean 115.55 (SD 15.506); 101.40 (16.117). P=0.07. Difference of 14.15 greater than MCID of 12.3[79]. Difference was significant at 6 weeks, p<0.001.<br>No complications related to TKR or LIA. Low back pain (1; 2), stroke (0; 1), CHF (1; 0)
		Peri-articular injection, 15 minutes before incision, of 100ml saline containing 50 mg bupivacaine hydrochloride 0.5%, 1 ml morphine sulphate 10 mg/ml, 300 µg epinephrine (1:1000) and 30 mg ketorolac	100ml saline containing 300 µg epinephrine (1:1000)	
McDonald et al. 2016[45]<br>UK<br>2010-2011<br>1 hospital	Primary unilateral TKR for osteoarthritis<br>113; 109 received common spinal anaesthesia (121; 121 randomised)<br>Median 68 (IQR 62, 72); 67 (62, 73)<br>59%; 55%	Oral premedication with 10-20mg temazepam, 150mg ranatidine, 10mg dexamethasone, 300mg gabapentin, 1g paracetamol.<br>Spinal anaesthesia		12 months<br>9; 11 of those receiving treatments<br>Low risk of bias<br>No separate pain outcome. Mean OKS at 12 months: median 41 (IQR 35, 44); 41 (34;44). P=0.915<br>Suspected infection 2; 1. MI 0; 1. GI bleed 1; 0. renal failure 1; 0. Died 2; 0)
		Intra-articular and subcutaneous infiltration during surgery of 200 ml of 2mg/ml ropivacaine without adrenalin or additives. Catheter inserted, and 20 ml infiltrate injected following wound closure. Further boluses of 40 ml 2 mg/ml ropivacaine via infusion pump 4 hours after leaving theatre and morning of POD1. Two	Epidural PCA with 4 ml of 2.5 mg/ml levobupivacaine introduced at end of surgery. Thereafter self-medication with 2 ml of 1.25 mg/ml bupivacaine with 15 minutes lockout until morning of POD1. Nurse-administered rescue of 4 ml of 2.5 mg/ml levobupivacaine.	

For peer review only - http://bmjopen.bmj.com/site/about/guidelines.xhtml

		additional top ups of 40 ml 2mg/ml ropivacaine were prescribed if required.		
Celecoxib vs placebo				
Meunier et al. 2007[51]<br>Sweden<br>2004-2005<br>1 centre	Elective primary unilateral TKR for osteoarthritis<br>25; 25 (24; 20 received treatment)<br>Mean 68 (SD 6.3); 69 (7.7)<br>71%; 40%	Spinal anaesthesia with bupivacaine 17.5-20mg. i.v. midazolam or propofol sedation if needed. Paracetamol 1 g preoperatively and then with tramadol 50-100 mg 4 times a day during hospital stay. Ketobemidone (2.5-5mg i.v. or subcutaneous) on demand. Paracetamol and tramadol used as required after discharge.		12 months<br>No losses to follow up after surgery reported<br>Low risk of bias<br>No effect of celecoxib on VAS or KOOS pain at 1 year.<br>DVT: 0; 1. Deep infection: 0; 0.
		Oral celecoxib 200mg 1 hour preoperatively and twice daily for 3 weeks	Oral placebo 200mg 1 hour preoperatively and twice daily for 3 weeks	
Ketamine vs placebo				
Perrin and Purcell2009 [107]<br>Australia<br>Before 2009<br>1 centre (pilot study)	Elective unilateral TKR<br>16 (5; 7 completed study per protocol)<br>Mean 65.6 (SD 10.2); 60.3 (11.9)<br>40%; 43%	Intrathecal injection of 15mg bupivacaine and 100µg morphine. General anaesthesia. After surgery 1.5g paracetamol and then 750mg every 4 hours; PCA with morphine 2mg boluses with 10-minute lockout; morphine rescue 2.5mg intravenously as required; and rescue oral ibuprofen 800mg.		6 months<br>3 protocol breaches and 1 patient with uncontrolled pain.<br>High risk of bias due to non-ITT reporting and recruitment difficulties<br>2/5 ketamine group had mild/moderate pain on the WOMAC pain scale at 26 weeks or failed to improve compared with 5/7 controls.<br>1 adverse psycho-mimetic effect not attributed to intervention or control treatment
		Ketamine 0.5mg/kg bolus followed by 4µg/kg/min infusion. Commenced before surgical incision and continued until wound bandaged or syringe empty.	Saline infusion. Commenced before surgical incision and continued until wound bandaged or syringe empty.	
Ketamine vs Nefopam vs placebo				
Aveline et al. 2014[52]<br>France<br>2005<br>1 centre	Elective primary unilateral TKR for osteoarthritis<br>25; 25; 25<br>Mean 73 (SD 9); 72 (9); 70 (7)<br>67%; 60%; 63%	General anaesthesia induced with 1.5-2mg/kg propofol, 1µ/kg remifentanil and a single bolus of cisatracurium 0.15mg/kg. Remifentanil infusion at 0.15µg/k/min until skin closure. Anaesthesia maintained with sevoflurane 0.9-1.2% with 50% nitrogen in oxygen. 20 mins before skin closure, 0.15mg/kg i.v. morphine bolus and 0.625mg droperidol. PCA with morphine hydrochloride 1 mg i.v. bolus with 7-min lockout. On arrival in recovery room, 3 mg i.v. morphine boluses at 5 minute intervals.		6 and 12 months<br>3; 1; 2<br>Low risk of bias<br>Median DN4 at 12 months: 1 (IQR 1, 2); 1 (0, 1); 2 (1, 3). p=0.02 for difference between ketamine and placebo groups. Number of patients with VAS pain on movement score

For peer review only - http://bmjopen.bmj.com/site/about/guidelines.xhtml

		0.2mg/kg nefopam administered over 20 min before incision; 2mg/ml nefopam continuous infusion at 120µg/kg/hr until end of surgery and 60µg/kg/hr for 48 hours	0.2mg/kg ketamine administered over 20 min before incision; 2mg/ml ketamine continuous infusion at 120µg/kg/hr until end of surgery and 60µg/kg/hr for 48 hours	Saline administered over 20 minutes before incision; saline continuous infusion until second post-operative day	≥40mm at 12 months by group: nefopam (3/22, 13.7%), ketamine (3/24, 12.5%), and placebo group (6/23, 26.1%). Ketamine reduced DN4 pain (p=0.02) compared with placebo. At 12 months only 7/69 patients had DN4≥4 indicative of neuropathic pain.<br>Infection: 0; 0; 0. Revision: 0; 0; 0.
Pregabalin vs placebo					
Buvanendran et al. 2010[53]<br>USA<br>2006-2007<br>Single centre	Primary unilateral TKR for osteoarthritis.<br>120; 120 (9; 2 did not receive post-operative treatment but ITT analysis)<br>Mean 64.0 (SD 8.3); 63.3 (8.9)<br>76%; 70%	Sedation with midazolam and i.v. propofol. Combined spinal-epidural anaesthetic. 1.5ml 0.75% hyperbaric bupivacaine with 25µg fentanyl injected intrathecally. Catheter inserted for epidural drug administration.<br>LIA 60 ml 0.25% bupivacaine with epinephrine infiltrated into the wound at capsule closure. From completion of surgery until 32-42 hours post-operative, epidural infusion of fentanyl (5µg/ml) and bupivacaine (1mg/ml) initiated using continuous basal infusion of 6ml/hr with epidural PCA bolus doses (maximum 10ml/hr). Patients transitioned to oral opioid (morphine, oxycodone, and hydromorphone) as required. All patients received preoperative oral celecoxib 400mg 1–2 hours before surgery and 200mg twice daily for 3 days in hospital.			6 months<br>7; 5<br>Low risk of bias<br>Mean VRS pain score at 6 months: pregabalin 0.41 (SD 1.20); control 0.95 (1.80). p=0.0084. Distributions skewed but nonparametric Wilcoxon significant (p=0.0176). Difference of 0.54 less than MCID of 1.0.<br>In the pregabalin group the incidence of neuropathic pain measured using S-LANSS was 0% (0/113) and 5.2% (6/115) in the placebo group (p=0.014).<br>No clinically significant adverse events up to 6 months and no falls. Sedation, confusion and dry mouth more frequent in pregabalin than placebo group on day of surgery and first postoperative day.
		Oral pregabalin 300mg 1–2 h before surgery, 150mg twice daily for the first 10 postoperative days, 75mg twice daily on days 11 and 12, and 50mg twice daily on days 13 and 14	Oral placebo 1–2 h before surgery, twice daily for the first 10 postoperative days, twice daily on days 11 and 12, and twice daily on days 13 and 14		
FNB long duration vs FNB short duration					

For peer review only

For peer review only - http://bmjopen.bmj.com/site/about/guidelines.xhtml

Ilfeld et al. 2009[108]<br>USA<br>2005-2007<br>2 centres	Primary unilateral TKR for osteoarthritis<br>25; 25<br>Median 66 (IQR 60, 70); 64 (60, 69)<br>56%; 60%	Femoral catheter inserted using nerve stimulator. FNB with 0.2% ropivacaine infusion (8ml/hr basal; 4 ml patient-controlled bolus; 30-minute lockout) from surgery until a.m. POD1.<br>1 week oral acetaminophen (975mg every 6 hours), sustained release oral opioid (Oxycontin, 10mg every 12 hours), and either oral aspirin (650mg daily) or celecoxib (200mg every 12 hours). Oral oxycodone 5 mg tablets and/ or i.v. morphine sulfate 2-4 mg for breakthrough pain.		6 and 12 months<br>4; 1 lost to follow up<br>High risk of bias: uneven loss to follow up between groups; muscle weakness resulted in lower dose of infusion on POD1 (10 continuous; 3 saline)<br>Groups had similar WOMAC pain scores at 6 and 12 months (p>0.05).<br>MI: 1; 0. PE: 1; 0. Fall: 1; 0. Catheter leak, dislodged: 1; 2
		At 6 a.m. POD1, infusion pump replaced with infusion pump with 0.2% ropivacaine.<br>At 6 p.m. POD2 pump replaced with portable infusion pump (400ml 0.2% ropivacaine). Catheter removed evening of POD4.	At 6 a.m. POD1, infusion pump replaced but saline substituted.<br>At 6 p.m. POD2 pump replaced with portable infusion pump (saline). Catheter removed evening of POD4	
Ilfeld et al. 2011[109]<br>USA<br>2007-2009<br>2 centres	Primary unilateral cemented TKR for osteoarthritis<br>40; 40 (39; 38 included in RCT)<br>Median 61 (IQR 58, 67); 66 (60, 70)<br>67%; 66%	Femoral catheter inserted using nerve stimulator. FNB with 0.2% ropivacaine infusion (6ml/hr basal; 4ml patient-controlled bolus; 30-min lockout) from surgery until POD1.<br>1 week oral acetaminophen (975mg every 6 hours), sustained release oral opioid (Oxycontin, 10mg every 12 hours), and either oral aspirin (650mg daily) or celecoxib (200mg every 12 hours). Oral (oxycodone 5mg or 10mg tablets) and/ or i.v. opioids (morphine sulfate 2-4mg) for breakthrough pain.		12 months<br>11; 12 incomplete follow up<br>High risk of bias: 11;12 did not have 4 measures out of 6 up to 12 months; graph suggests WOMAC pain lower pre-intervention in continuous infusion group.<br>No difference in WOMAC pain scores between randomised groups (p>0.05).<br>Falls: 4; 0
		At 6 a.m. POD2, infusion pump replaced and 0.2% ropivacaine continued.<br>At 6 p.m. POD2 pump replaced with portable infusion pump (400ml 0.2% ropivacaine). Catheter removed evening of POD4	At 6 a.m. POD2, infusion pump replaced but saline substituted.<br>At 6 p.m. POD2 pump replaced with portable infusion pump (saline). Catheter removed evening of POD4	
Choy et al. 2011[35]<br>Korea<br>2006-2007	Primary unilateral TKR for osteoarthritis	Spinal anaesthesia. Continuous FNB via catheter until POD3. Catheter inserted with use of nerve stimulator. Analgesia induced with 20ml of 1:1 0.25% bupivacaine and 2% lidocaine with 1:200,000 epinephrine. Continuous		2 years<br>4; 3 lost to follow up

For peer review only - http://bmjopen.bmj.com/site/about/guidelines.xhtml

1 surgeon	33; 30 (2 patients received GA and excluded)<br>Mean 66.7 (SD 10); 67.5 (11)<br>97%; 93%	infusion with 0.125% bupivacaine 5.0ml/hr. i.v. PCA (butorphanol 4mg, ketorolac 150mg, saline 50ml), programmed to deliver 1 mg bolus (lockout 10 min) with maximum dose 6mg/hr. i.v. paracetamol 2g 4 times/ day and oral ibuprofen 600mg 3 times/ day for breakthrough pain			Low risk of bias for 2 year outcome measures.<br>At 2 years, intervention WOMAC pain mean 7.2 (SD 2), control 6.3 (SD 1); p=0.2<br>Superficial infection: 1; 1
		Continuous femoral nerve block via catheter continued from POD3 to POD7	Continuous femoral nerve block discontinued on POD3		
FNB continuous high concentration vs FNB low concentration vs FNB single					
Albrecht et al. 2014[34]<br>Canada<br>2009-2011<br>1 hospital	Scheduled primary unilateral TKR<br>32; 32; 35<br>Mean 61 (CI 57, 64); 63 (60, 67);63 (60, 66)<br>46%; 44%; 52%	Stimulating catheter inserted with ultrasound guidance. Immediately after catheter placement, 10ml mepivacaine 2% was injected through the catheter. SNB using 30 ml ropivacaine 0.2%. Spinal anaesthesia with 2.5 to 3.0 ml isobaric bupivacaine 0.5% and 0.1mg intrathecal morphine.			12 months<br>4;0;2 lost to follow up<br>Low risk of bias.<br>No separate pain outcome. Mean WOMAC score at 12 months: high concentration FNB 17 (95% CI 7, 27); 22 (14, 30); 18 (8, 27). P=0.68<br>Falls: 0; 0; 1
		Bolus of 20ml ropivacaine 0.2% with epinephrine 1:400,000 into the femoral catheter followed by ropivacaine 0.2% at a rate of 5 ml/hr with patient-controlled boluses of 5ml available every 30minutes.	Bolus of 20 ml ropivacaine 0.2% with epinephrine 1:400,000 into femoral catheter followed by ropivacaine 0.1% at rate of 10ml/hr with patient-controlled boluses of 10 ml available every 30 minutes.	Bolus of 30ml ropivacaine 0.375% with epinephrine 1:400,000 into the femoral catheter followed by normal saline at a rate of 1 ml/hr with patient-controlled boluses of 1mL available every 30minutes.	
FNB continuous vs Psoas compartment block vs FNB continuous and psoas compartment block					
Morin et al. 2005[110]<br>Germany<br>Before 2005<br>1 centre	Elective unilateral TKR<br>30; 30; 30	Oral pre-medication with 20mg chlorazepate. General anaesthesia with intravenous propofol and 4–8µg/kg i.v. fentanyl and desflurane in N2O. 100mg diclofenac suppository after anaesthesia induction and 2.5g intravenous metamizole before end of surgery. Post-operative 3 daily doses of oral diclofenac 50mg. i.v. PCA			9–12 months<br>7; 6; 5<br>High risk of bias due to large losses to follow up, non-blinded outcome collection, and differences between

For peer review only - http://bmjopen.bmj.com/site/about/guidelines.xhtml

	Median 68 (IQR 62, 74); 71 (63, 74); 65 (53, 73)<br>50%; 70%; 59%	with piritramide bolus 2mg as needed with lockout interval of 10 mins for 48 hours.			groups in BMI and anaesthetist's opinion of difficulty of catheter placement.<br>No difference between groups in level of pain at the knee joint during past 4 weeks: FNB median 2.5 (IQR 1, 4), FNB and SNB 2 (1, 4), Psoas block 2 (IQR 1, 4), p=0.44<br>No early complications but longer term adverse events not reported.
		Continuous FNB<br>Stimulating catheter used. Initial bolus of prilocaine 1% and ropivacaine 0.75%. 300mg prilocaine 1% (30ml) and 150mg ropivacaine 0.75% (20ml). During first 48hrs post-operative ropivacaine 0.2% infusion 14ml/hr.	Continuous FNB and continuous SNB<br>Stimulating catheter used. Initial bolus of prilocaine 1% and ropivacaine 0.75%. In each catheter: 200mg prilocaine 1% (20ml) and 75mg ropivacaine 0.75% (10ml). During first 48hrs post-operative infusion through each catheter of 0.2% ropivacaine 7ml/hr.	Continuous psoas compartment block<br>Stimulating catheter used. Initial bolus of prilocaine 1% and ropivacaine 0.75%. 300mg prilocaine 1% (30ml) and 150mg ropivacaine 0.75% (20ml). During first 48hrs post-operative ropivacaine 0.2% infusion 14ml/hr.	
ACB continuous vs FNB continuous					
Davidson et al. 2016[111]<br>USA<br>2013-2014<br>2 studies combined from 1 centre	Primary, unilateral TKR or unicompartmental<br>54 (39 TKR, 16 UKR); 56 (41 TKR, 15 UKR)<br>TKR mean 67 (SD 8); 66 (7). UKR 70 (10); 68 (12)	Spinal or general anaesthesia. Intra-operative i.v. fentanyl, hydromorphone, and/or morphine.<br>LIA with 30 ml ropivacaine (0.5%), ketorolac (30 mg), and epinephrine (5 μg/ml).<br>Post-operative: oral acetaminophen (975 mg every 6 hr), celecoxib (200 mg every 12 hr), and sustained release oxycodone (10 mg every 12 hr). For breakthrough pain, infusion pump bolus (4 ml, 30-min lock-out). Rescue opioid titrated to pain severity. 10 ml lidocaine (2%) bolus was given via the perineural catheter for moderate or severe pain.			12 months<br>31; 29<br>High risk of bias due to partial follow up<br>TKR and UKR combined. Pain score (0-10) at 12 months median 0.0 (IQR 0.0, 3.0); 0.5 (0.0, 2.0). P=0.80). Pain score >0: 35%; 32%. P=0.65. No difference at 4 months when follow up more complete (51;

For peer review only

	TKR 59%; 66%. UKR 47%; 47%	Ultrasound guided ACB. Ropivacaine 0.2% at basal rate of 6 ml/hr, a 4-ml bolus, and a lockout of 30 minutes	Ultrasound guided continuous FNB. Ropivacaine 0.2% at basal rate of 6 ml/hr, a 4-ml bolus, and a lockout of 30 minutes	52) in pain score (p=0.80) or pain score >0 (p=0.48).<br>Falls in hospital: 2; 5
ACB single vs FNB single				
Macrinici et al. 2017[38]<br>USA<br>Before 2017<br>1 centre	Primary unilateral TKR, indication not specified (selected by the surgeon for TKA)<br>49; 49<br>Mean 67 (SD 8); 67 (8)<br>61%; 63%	Multimodal regimen including NSAIDs, non-opioid analgesics, opioids. LIA 40ml Marcaine 0.25%.<br>All patients received an ultrasound guided needle insertion into ACB and FNB sites.		6 months<br>3; 4 lost to follow up. 6; 3 complications<br>Low risk of bias<br>VAS pain similar between groups at 6 months. No difference in functional outcomes<br>Medical complications: 3; 0. Surgical complication: 0; 1. Temporary foot drop: 3; 2.
		Immediately after surgery, 30ml solution with 100ml Marcaine into ACB site. 30 ml saline into FNB site	Immediately after surgery, 30ml solution with 100ml Marcaine into FNB site. 30 ml saline into ACB site	
FNB continuous vs oral opioid				
Nader et al. 2012[39]<br>USA<br>2007-2008<br>1 surgeon	Elective unilateral TKR<br>31; 31<br>Median (IQR) 65 (60, 76); 64 (60, 71)<br>58%; 77%	Before surgery, patients received 1–2mg midazolam as needed. Epidural with 10mg 0.5% isobaric bupivacaine injected intrathecally. Intraoperative sedation with propofol infusion of 25-75mcg/kg/minute. In post-anesthesia recovery area, PCA epidural with basal infusion of 3 ml/hr (1 mg/ml bupivacaine and 10 mg/ml hydromorphone) with patient-activated boluses of 3 ml with a lockout interval of 15 minutes and per hour maximum of 15 ml. Infusion discontinued and epidural catheter removed on morning of POD 1. All subjects received 5 mg warfarin on evening of surgery and 40 mg enoxaparin starting on POD 1		6 and 12 months<br>1; 1 lost to follow up at 12 months<br>Low risk of bias<br>No difference in overall median NRS pain score at 6 months and 12 months: 0 (IQR 0, 1); 0 (0, 1). p=1.0. At 12 months, some evidence favouring hydrocodone for pain ascending/ descending stairs: 1 (0, 2); 0 (0, 0). p=0.01. Also, suggestion of reduced pain in hydrocodone group at night in bed (p=0.06) and sitting/ lying (p=0.07), standing upright (p=0.10). No difference walking on flat surface (p=0.41).<br>Falls in month after surgery: 1;0. Positive joint aspirate: 3; 0. VTE: 0; 4.
		Continuous FNB inserted with use of stimulator. After discontinuation of epidural anaesthesia on the morning of POD1 10mL bolus of ropivacaine 0.25% followed by 5ml/h infusion of 0.1% ropivacaine. On morning of POD 2, ropivacaine infusion	10 mg oral hydrocodone plus 325mg acetaminophen every 4-6 hours administered for pain as needed. Sustained release oxycodone 10mg for 12 hours with oral hydromorphone 2 mg over	

For peer review only - http://bmjopen.bmj.com/site/about/guidelines.xhtml

		discontinued. Femoral catheter removed 24 hours after previous dose of enoxaparin.	4 hours for breakthrough pain	
FNB continuous vs PCA				
Wang et al. 2015[112]<br>China<br>2012-2013<br>3 centres	Elective unilateral TKR<br>82; 86<br>No significant differences in age or sex	General anaesthesia with midazolam (0.02-0.04mg/kg), fentanyl (1µg/kg), propofol (1-2mg/kg) and cisatracurium (0.15mg/kg). Anaesthesia maintained with sevoflurane during surgery. Intramuscular injection with 10mg metoclopramide and 2.5mg droperidol 30 minutes before surgery. Post-surgery, celocoxib and parecoxib 40mg for patients with severe pain, and i.v. morphine if needed.<br>Continuous FNB with ultrasound stimulator. After surgery, 0.2% ropivacaine (20ml) injected through catheter. Then an analgesia pump was attached delivering 0.2% ropivacaine 8ml/hr.	Epidural PCA 0.2% ropivacaine was injected at a rate of 5 ml/hr in a 2ml pulse dose	6 and 12 months<br>2; 4 lost to follow up at 12 months<br>Unclear risk of bias: limited reporting of randomisation methods.<br>No differences were observed between groups at 6 or 12 months for any HSS domain including pain.<br>No nerve injuries
Peng et al. 2014[40]<br>China<br>Before 2014<br>1 centre (2 surgical teams with 4 surgeons and 2 anaesthesiologists)	Primary unilateral TKR<br>140;140<br>Mean: 66.8 (SD 9.4); 68.0 (SD 11.2)<br>73%; 65%	General intravenous and inhalational anaesthesia: midazolam 0.1-0.15mg/kg (etomidate 0.15-0.2mg/kg for patients >65 years), propofol 2.0-2.5mg/kg, sufentanil citrate 0.3-1.0µg/kg, and vecuronium 0.08-0.12mg/kg for induction of anaesthesia. Maintenance with inhalation of 1%-3% sevoflurane and continuous intravenous infusion of remifentanil 7-8µg/kg/hr and propofol 25-75µg/kg/min. After wound closure, 5-10µg intravenous sufentanil and loading dose of PCA injected. i.v. injection of 4mg ondansetron.<br>FNB with ultrasound guidance. Initial dose of 10ml 2% lidocaine and 10ml 1% ropivacaine. 30 minutes before end of operation, catheter connected to PCA pump; patients received loading dose of 5ml of 0.15% ropivacaine followed by infusion of 0.15% ropivacaine at 5ml/hr, with bolus of 5mL	i.v. PCA with tramadol 800mg, flurbiprofen axetil 100mg, and dexamethasone 5mg with saline to a volume of 80ml. Loading dose of 2ml followed by an infusion rate of 1 ml/hr with bolus of 2 ml. Lock time 15min.	6 and 12 months<br>31; 38 at 12 months<br>Low risk of bias<br>Chronic post-operative pain (NRS 1+) in 38.5% of PCA group at 6 months compared with 25.7% in FNB group (p=0.021). No difference at 12 months (p=0.273).<br>Authors only reported short term adverse events associated with use of PCA.

For peer review only

For peer review only - http://bmjopen.bmj.com/site/about/guidelines.xhtml

		and lock time of 30 min. Preoperatively, a loading dose of 30ml was injected for intraoperative analgesia.		
Wu and Wong 2014[44]<br>China<br>2009-2011<br>1 centre	Unilateral elective TKR, 98% for osteoarthritis<br>40; 39 (30; 30 after post randomisation exclusions)<br>Mean 68.8 (SD 6.4); 68.9 (7.5)<br>73%; 73%	Paracetamol, sustained release diclofenate, opioids (codeine or morphine). Spinal anaesthesia		6 months<br>2; 2 not pre- and peri-operative exclusions<br>Low risk of bias<br>No separate pain outcome but improvement of KSS from pre-operative was FNB 48.73 and PCA 44.7 (p=0.513)<br>Including patients not followed up. Deaths: 0; 0. Infection: 1;1. DVT: 2; 3. Shock: 3;2. Transfusion: 2;3. Also from excluded cases. Atrial fibrillation and confusion: 0; 1. PE: 0; 1. Sepsis: 1;0. ICU admission for shock: 1; 0.
		Catheter inserted under nerve stimulation and ultrasound guidance. Standardised bolus of 15 mL 0.5% levobupivacaine. Continuous infusion of 8 to 12 mL/h 0.08% levobupivacaine postoperatively until POD 3	Intravenous PCA morphine after the operation	
FNB and SNB continuous vs epidural PCA				
Anastase et al. 2014[113]<br>Germany<br>2010-2011<br>1 centre	Primary unilateral TKR<br>55; 50<br>Mean 68.2 (SD 9.2); 69.7 (SD 8.7)<br>65%; 69%	Premedication with 10 mg oral clorazepate. Spinal anaesthesia with light sedation: 12.5mg 0.5% bupivacaine. Supplemental postoperative analgesia available with i.v. piritramid		6 and 12 months<br>15; 14<br>High risk of bias due to large loss to follow up<br>Pain during previous 4 weeks: 1 no pain, 2 very little, 3 little, 4 moderate, 5 loud, 6 very loud (translation from German). No difference at 6 months p=0.37. At 12 months, FNB/SNB median 2.00 (1.00, 2.00), PCA 2.00 (2.00, 2.00) p=0.004 favouring FNB/SNB.<br>No falls associated with quadriceps weakness. 6 and 12 month adverse events not reported.
		After spinal anaesthesia installed, SNB and FNB catheters inserted with ultrasound guidance. 5 ml bolus 0.2% ropivacaine. FNB with an hourly rate of 5 ml, bolus administration of 5 ml by the patient and the lock-out interval of 20 mins. SNB 5 ml/h to a maximum of 8 ml/h, 5 ml bolus administered by the patient	Epidural catheter installed at the same time as spinal catheter. 5ml 0.2% ropivacaine and PCA performed through the epidural catheter. Hourly rate 3 ml, bolus administration of 5 ml, and lock-out period of 30 minutes.	

For peer review only - http://bmjopen.bmj.com/site/about/guidelines.xhtml

		and lock-out interval of 20 minutes.		
**FNB single vs LIA**				
Fan et al. 2016[36]<br>China<br>2012-2014<br>Single hospital, 2 surgeons	Primary unilateral TKR (75% osteoarthritis; 25% rheumatoid arthritis)<br>80; 80 (78; 79 in analysis)<br>Mean 68.4 (SD 8.8); 67.6 (6.3)<br>79%; 86%	General anaesthesia in all but 1 in each group. After surgery, i.v. morphine, PCA and parecoxib 40mg<br>FNB performed pre-operatively with 20ml ropivacaine 0.5%.<br>After cementing prostheses, 50ml of saline injected into periarticular soft tissue.	Placebo equivalent of FNB with saline<br>After cementing prostheses, 50ml of LIA mixture containing morphine (1ml: 10mg), ropivacaine (10ml: 100mg), and diprospan (1ml: 5mg betamethasone dipropionate and 2mg betamethasone sodium phosphate) injected into periarticular soft tissue.	1 year<br>3 protocol violations<br>Low risk of bias<br>No separate pain outcome. Mean KSS at 1 year similar between groups: 94.2 (SD 2.6); LIA 93.9 (3.1). p=0.51<br>Infection: 0; 0. DVT: 1; 1. Femoral nerve injury: 1; 0.
**FNB single and epidural vs LIA**				
Reinhardt et al. 2014[41]<br>USA<br>2010-2012<br>Single hospital, 2 surgeons	Elective unilateral TKR for osteoarthritis<br>51; 51 (49; 45 received allocated intervention)<br>Mean 67.9 (SD 10.9); 66.6 (10.1)<br>59.2%; 57.8%	Spinal anaesthetic (2.5ml 0.5% bupivacaine). Mobic 15mg daily. Oral Perocet or Vicodin as required. Subcutaneous Dilaudid for severe breakthrough pain. Intravenous Toradol.<br>Combined spinal-epidural (500ml hydromorphone 10µg/ml and bupivacaine HCl 0.06%).<br>Single intra-operative FNB injection (30ml 0.25% bupivacaine).<br>Continuous 48-hour epidural infusion (4ml/hr with 4ml per demand dose, locked out every 10 minutes with an hourly limit of 20ml). Epidural infusion weaned to 2ml/hr on POD1 and to 0 ml/hr at 5 p.m. on POD1. Demand dose with	Intra-articular knee catheter placed intraoperatively with continuous 0.2% ropivacaine infusion at 7 ml/hr until POD2.<br>Placebo epidural catheter, no FNB, and postoperative placebo continuous epidural infusion of saline.	1 year<br>0: 0 of patients who received allocated intervention<br>Low risk of bias<br>VAS pain at 1 year similar between groups (noted in text and shown graphically)<br>No wound-related complications or infections. 1 DVT and 1 DVT plus PE in epidural group. Arthrofibrosis: 2; 1

For peer review only - http://bmjopen.bmj.com/site/about/guidelines.xhtml

		lockout parameters continued for 48 hours.<br>Placebo intraarticular knee catheter placed intra-operatively with continuous saline 7ml/hr infusion until POD2.		
LIA with corticosteroid vs LIA with no corticosteroid				
Seah et al. 2011[48]<br>Singapore<br>2004-2005<br>1 hospital	TKR<br>50; 50<br>Mean 65.4; 67.9<br>Sex not stated	General or spinal anaesthesia. Postoperative oral naproxen and PCA (with morphine bolus of 1mg, lock-out time 5 minutes, and maximum dose 8 mg/hr) for 48 hours.		6 months and 2 years<br>No losses to follow up reported<br>Low risk of bias<br>No separate pain outcome but no statistically significant difference in OKS between groups at 2 years<br>Deep infection: 1; 1
		Intraoperative periarticular injection of 0.5ml/kg 1:200,000 epinephrine and 0.5% bupivacaine diluted with 30ml of normal saline. 40mg of corticosteroid (triamcinolone acetonide) was added to half the mixture. The solution with the corticosteroid was injected into the deep tissues. The remaining solution was injected into the skin incision before closure.	Intraoperative periarticular injection of 0.5ml/kg 1:200,000 epinephrine and 0.5% bupivacaine diluted with 30ml of normal saline. Half the mixture was injected into the deep tissues. The remaining solution was injected into the skin incision before closure.	
Yue et al. 2013[114]<br>China<br>2011-2012<br>1 hospital	Unilateral TKR for osteoarthritis<br>36; 36<br>Mean 70.2 (SD 6.4); 69.3 (5.7)<br>89%; 89%	General anaesthesia. PCA (25 mg/100ml morphine: a 1mg bolus, 6 minutes lock-out, and 5mg/hr maximum) for 72 hours after surgery. 5-10mg intramuscular morphine as rescue. Celecoxib pre- and post-operatively		6 and 12 months<br>No loss to follow up reported<br>Unclear risk of bias.<br>No separate pain outcome. No difference in mean KSS between groups at 6 or 12 months<br>No incision infection or tendon rupture complications
		Injections with local anaesthetic agent and adrenaline (0.75% ropivacaine 30ml, 1:1000 adrenaline 0.5ml, and isotonic sodium chloride solution 70ml) plus corticosteroid (1ml betamethasone).	Injections with local anaesthetic agent and adrenaline (0.75% ropivacaine 30ml, 1:1000 adrenaline 0.5ml, and isotonic sodium chloride solution 70ml) with no added corticosteroid.	

For peer review only

For peer review only - http://bmjopen.bmj.com/site/about/guidelines.xhtml

		Another 50ml syringes fluid without corticosteroid was infiltrated into the skin	Another 50ml syringes fluid without corticosteroid was infiltrated into the skin		
LIA including ketorolac vs epidural					
Spreng et al. 2012[115], Spreng et al. 2010[116]<br>Norway<br>2007–2009<br>1 hospital	Unilateral, non-cemented TKR with no patella resurfacing<br>34; 34; 34<br>66.5 (SD 11.); 67.2 (SD 8.9); 65.8 (SD 10.1)<br>61%;61%;67%	Premedication with oral paracetamol (1-2g). Spinal anaesthesia with 13-15mg bupivacaine 5mg/ml with 20μg fentanyl. If indicated, up to 10ml/hr 10mg/ml propofol for sedation. Acetaminophen 1g every 6 hours. i.v. PCA morphine for 48 hours after surgery (2mg bolus with 10 minutes lockout time). When PCA stopped, 10mg slow release oxycodone twice daily. 5mg oxycodone as rescue analgesia.			12 months<br>13 did not provide complete data<br>Unclear risk of bias due to limited reporting (long-term outcome only reported as conference abstract).<br>Perioperative analgesic treatment did not have any significant influence on any KOOS outcomes.<br>Infection: 0; 0; 1. No long-term adverse events reported
		i.v. injection of ketorolac 1ml (30mg/ml) and morphine 5ml (1mg/ml).<br>Infiltration with ropivacaine 150mg, epinephrine 0.5mg, ketorolac 30mg and morphine 5mg in 150ml saline.<br>After closure, catheter placed into knee joint and 10ml infiltrate injected. 22-24 hours after surgery, 20ml injection through catheter of ropivacaine 19ml (7.5mg/ml) and ketorolac 1ml (30mg/ml). i.v. injection of	i.v. injection of 6ml saline.<br>Infiltration with ropivacaine 150mg, epinephrine 0.5mg, ketorolac 30mg and morphine 5mg in 150ml saline.<br>After closure, catheter placed into knee joint and 10 ml infiltrate injected. 22-24 hours after surgery, 20ml injection through catheter of ropivacaine 19ml (7.5mg/ml) and saline 1ml. i.v. injection of saline 1ml.<br>Sham epidural catheter.	Epidural catheter inserted immediately before spinal anaesthesia. When spinal anaesthesia started to wear off, epidural infusion for 48 hrs with 6-10 ml/hr fentanyl 2μg/ml, epinephrine 1μg/ml, bupivacaine 1mg/ml.<br>No knee infiltrations.<br>Sham knee catheter with no injections	

For peer review only - http://bmjopen.bmj.com/site/about/guidelines.xhtml

		ketorolac 1ml (30mg/ml).<br>Sham epidural catheter.			
**Spinal with added high dose morphine sulphate vs spinal with added low dose morphine sulphate vs spinal with no morphine sulphate**					
Foadi et al. 2017[117]<br>Germany<br>Before 2017<br>1 centre	Unilateral TKR or THR for osteoarthritis<br>16; 16; 17<br>Mean 67.63 (SE 2.45); 67.33 (2.87); 63.71 (3.14) 56%; 44%; 65%	3ml spinal anaesthesia with 0.5% bupivacaine<br>Post-operative 1 g metamizole (orally or intravenously) every 4 hours. 5 mg morphine (intravenous or subcutaneous) as rescue medication			6 months<br>"only a few dropouts". >70% questionnaire return rate.<br>Unclear risk of bias due to limited reporting of pilot RCT.<br>No difference in WOMAC pain between groups at 6 months.<br>No adverse events noted
		0.2mg morphine sulphate added to spinal anaesthesia	0.1mg morphine sulphate added to spinal anaesthesia	No morphine sulphate added to spinal anaesthesia	

2. Myofascial trigger point dry needling

**Author**<br>**Country**<br>**Recruitment dates**<br>**Setting**	**Indication**<br>**Number randomised intervention; control**<br>**Age**<br>**% female**	**Common pain management**		**Follow up**<br>**Losses to follow up intervention; control**<br>**Risk of bias issues**<br>**Key results**
		**Group 1 (intervention)**	**Group C (control)**	
Mayoral et al. 2013[118]<br>Spain<br>2007-2008<br>Single centre	Unilateral TKR for osteoarthritis<br>20; 20<br>Mean 71.7 (SD 6.1); 72.9 (7.9)<br>72.5%	General or spinal anaesthesia		6 months<br>4; 5<br>High risk of bias due to large losses to follow up<br>WOMAC pain at 6 months: mean 3.24 (SD 3.03); 3.13 (2.72). Difference not statistically significant. No difference between groups in VAS pain (p=0.725) or proportion of patients reporting significant VAS pain at 6 months.
		After anaesthesia and surgery started, dry needling applied 20 times to all myofascial trigger points by a trained and experienced physical therapist.	If spinal anaesthesia used, dry needling simulated behind screen	

For peer review only - http://bmjopen.bmj.com/site/about/guidelines.xhtml

				No complications related to the dry needling intervention. Other adverse events not collected.

3. Tourniquet

**Author**<br>**Country**<br>**Recruitment dates**<br>**Setting**	**Indication**<br>**Number randomised intervention; control**<br>**Age**<br>**% female**	**Common blood conservation strategies**		**Follow up**<br>**Losses to follow up intervention; control**<br>**Risk of bias issues**<br>**Key results**
		**Group 1 (intervention)**	**Group C (control)**	
Ejaz et al. 2014[54]<br>Denmark<br>2011-2012<br>1 centre	Primary unilateral TKR for osteoarthritis<br>35; 35 (33; 31 received intervention)<br>Mean 68 (SD 8.0); 68 (7.8)<br>45.5%; 45.2%	Before surgery, oral tranexamic acid (1g). Tranexamic acid (0.5g) 3 hours after surgery and 6 and 12 hours postoperatively.		6 and 12 months<br>0; 0 of those who received intervention<br>Low risk of bias<br>Statistically significant difference in KOOS pain intensity at 2 months favouring TKR without a tourniquet (p < 0.001). Small difference between groups not statistically significant at 6 and 12 months.<br>Small number of adverse events did not suggest extra risk in the group with no tourniquet.
		Appropriately sized thigh tourniquet applied. Limb exsanguination by elevation for 2 minutes and cuff inflated to 250mm Hg.	Appropriately sized thigh tourniquet applied but not inflated. Served as safety device in case of uncontrollable bleeding.	
Liu et al. 2014[56]<br>Australia<br>Before 2014<br>1 surgeon	Unilateral TKR for osteoarthritis<br>10; 10<br>Mean 67.0; 70.0<br>30%; 10%	PCA. No CPM		6 and 12 months<br>0; 0<br>Low risk of bias<br>No separate pain measure. Total OKS not significantly different at 6 and 12 months<br>Blood transfusions: 3; 0.
		Tourniquet inflated to 300 mmHg before skin incision. Tourniquet deflated after wound closure and dressing.	Tourniquet placed but not inflated	
Mittal et al. 2012[57]<br>Australia<br>2008-2010	Primary unilateral TKR<br>31; 34	Autologous blood re-infused if required		1 year<br>5; 2
		Short duration. Tourniquet set at 300mm Hg inflated	Long-duration. Tourniquet set at 300mm Hg inflated before	

For peer review only - http://bmjopen.bmj.com/site/about/guidelines.xhtml

<table>
<tr><td>1 centre</td><td>Mean 67.5 (SD 8.9); 66.6 (8.4)
81%:74%</td><td colspan="2">prior to cement application and deflated when cement hardened</td><td colspan="2">skin incision and deflated when cement hardened</td><td>Low risk of bias. However, RCT terminated early due to increased need for blood transfusion in short duration tourniquet group.
No separate pain outcome. Total OKS (0-48) at 52 weeks higher in long-duration group reflecting better recovery than short duration group but not significantly (p=0.12). Mean difference approximately 5 which is greater than MCID of 4[78].
Transfusions: 10; 2. Patient reported adverse event: 26; 12</td></tr>
<tr><td rowspan="2">Abdel-Salam and Eyres 1995[119]
UK
Date not stated
1 surgeon</td><td rowspan="2">Primary unilateral TKR of which 91% osteoarthritis
40; 40
Mean 72 (range 65-80); 74 (64-82)
57.5%; 62.5%</td><td colspan="4">Tourniquet placed around thigh</td><td rowspan="2">1 and 2 years
0; 0
Unclear risk of bias due to limited reporting of methods. No pain measure or PROM
Surgeon recorded HSS score at 1 year 90 (78-97); 91 (80-97). Not significantly different at 1 or 2 years.
Blood loss similar between groups. Wound infections: 5;0. DVT: 4;0</td></tr>
<tr><td colspan="2">Limb exsanguinated for 2 minutes and tourniquet inflated to twice systolic blood pressure</td><td colspan="2">Tourniquet not inflated</td></tr>
<tr><td rowspan="2">Şükür et al.2016[120]
Turkey
2015
1 surgeon</td><td rowspan="2">Primary unilateral TKR, in women with osteoarthritis
30; 30; 30; 30
Mean 67.0 (SD 7.0); 66.9 (8.5); 68.4 (6.9); 68.4 (6.8)
100%</td><td colspan="4">Pneumatic tourniquet inflated to 125mm Hg above systolic blood pressure</td><td rowspan="2">6 months
0;0;0;0
High risk of bias. KSS outcome noted in methods but not presented in results.
KSS results not reported at 6 months but no significant differences between groups at 3 months.
Surgical and wound complications similar between groups. No infections, fractures or instability requiring revision within 6 months</td></tr>
<tr><td>Knee in 90° flexion and tourniquet deflated during wound closure</td><td>Knee in 90° flexion and tourniquet inflated during wound closure</td><td>Knee in full extension and tourniquet deflated during wound closure</td><td>Knee in full extension and tourniquet inflated during wound closure</td></tr>
<tr><td></td><td></td><td colspan="4">Blood transfusion if required</td><td>3-22 months, mean 12;13 months</td></tr>
</table>

For peer review only

For peer review only - http://bmjopen.bmj.com/site/about/guidelines.xhtml

<table>
<tr><td>Zhang et al.2016 [121]
China
2014-2015
1 hospital</td><td>Primary TKR for osteoarthritis
84; 82
Not reported
Not reported</td><td colspan="3">Tourniquet</td><td colspan="3">No tourniquet</td><td>Not clear
High risk of bias. Variable follow up. HSS outcome noted in methods but not presented in results.
HSS not reported.
Transfusion rates similar between groups. At mean follow up of 12 -13 months, patients operated on without a tourniquet had a lower rate of DVT (2.4%) compared with those with a tourniquet (10.7%).</td></tr>
<tr><td rowspan="2">Zhang et al. 2017[58]
China
2008-2011
1 surgeon</td><td rowspan="2">Primary unilateral cemented TKR for osteoarthritis
50; 50; 50
Mean 70.3 (SD 6.6); 71 (10.2); 68.2 (6.8)
54%; 60%; 50%</td><td colspan="6">Tourniquet inflated to 300-337mm Hg. Tranexamic acid not generally used</td><td rowspan="2">6 months
0; 0; 0
Low risk of bias
No separate pain outcome. HSS similar between groups at 6 months (p=0.839).
At 2 weeks DVT: 0; 0; 1. Intramuscular vein thrombosis: 4; 3; 3. Transfusions: 30%; 26%; 10%</td></tr>
<tr><td colspan="2">Tourniquet for entire operation</td><td colspan="2">Tourniquet removed before wound closure</td><td colspan="2">Tourniquet from first bone osteotomy until wound closure</td></tr>
<tr><td rowspan="2">Huang et al. 2017[55]
China
2015
1 centre</td><td rowspan="2">Primary unilateral TKR for osteoarthritis
50; 50
Mean 66.2 (SD 8.3); 65.1 (6.8)
64%; 68%</td><td colspan="6">Tranexamic acid</td><td rowspan="2">6 months
0; 0
Low risk of bias
VAS pain similar between groups at 6 months (p=0.728). Mean HSS score 90.3 (SD 3.2); 91.2 (2.5). P=0.151
DVT: 0; 0. PE: 0; 0. Intramuscular venous thrombosis: 6; 4. Superficial infection: 1; 0. Wound secretion: 6; 0. No significant difference in blood loss between groups.</td></tr>
<tr><td colspan="3">Tourniquet</td><td colspan="3">No tourniquet</td></tr>
</table>

4. Compression bandage

**Author**	**Indication**	**Common treatments**	**Follow up**

For peer review only - http://bmjopen.bmj.com/site/about/guidelines.xhtml

Country<br>Recruitment dates<br>Setting	Number randomised intervention; control<br>Age<br>% female	Intervention	Control	Losses to follow up intervention; control<br>Risk of bias issues<br>Key results
Brock et al. 2017[122]<br>UK<br>2013-2014<br>1 hospital	Primary unilateral TKR for osteoarthritis<br>25; 25 (24 received intervention)<br>Mean 67.3 (SD 8.2); 69.5 (6.8)<br>66.7%; 64.0%	Hydrocolloid dressing left in place until clips removed on day 10-14<br>Soft inner layer with compressive outer layer bandage. Removed after 24 hours.	Standard bandaging with soft inner layer and crepe bandage outer layer. Removed after 24 hours and cryocuff used.	6 months<br>0; 0 of patients receiving intervention<br>Low risk of bias<br>No separate pain outcome. Mean OKS similar between groups at 6 months: 35.8 (SD 7.7); 34.3 (10.6). P=0.58<br>No infections or thromboembolic events in either group

5. **Blood conservation**

Author<br>Country<br>Recruitment dates<br>Setting	Indication<br>Number randomised intervention; control<br>Age<br>% female	Common blood conservation strategies		Follow up<br>Losses to follow up intervention; control<br>Risk of bias issues<br>Key results
		Intervention	Control	
Tranexamic acid				
Sa-Ngasoongsong et al. 2011[64]<br>Thailand<br>2008-2009<br>1 hospital	Primary knee osteoarthritis with unilateral primary cemented computer assisted TKR<br>24; 24<br>69.0 (SD 8.2); 69.2 (7.6)<br>91.7%; 75%	Drain and compressive dressing<br>25ml saline solution containing 250mg tranexamic acid injected into knee joint after fascial closure	25ml saline solution injected into knee joint after fascial closure	6 months<br>0; 0<br>Low risk of bias<br>No separate pain score reported but WOMAC overall score mean 18.6 (SD 7.6); 20.8 (6.4). P=0.282<br>Lower peri-operative blood loss in tranexamic acid group and need for blood transfusion, 1/24 compared with 8/24 in control group. No DVT,

For peer review only - http://bmjopen.bmj.com/site/about/guidelines.xhtml

					wound complications or infection reported in either group
Kim et al. 2014[61]<br>Korea<br>2009-2011<br>1 hospital	Primary unilateral TKR for osteoarthritis<br>90; 90<br>Mean 73.5 (SD 5.5); 71.9 (SD 5.9)<br>88%; 87%	Tourniquet, drain, compressive dressing. Allogenic blood transfusion and intravenous iron and erythropoietin if required			1 year<br>0; 0<br>Low risk of bias<br>WOMAC pain mean 3.2 (2.6); 2.8 (2.3). Difference not statistically significant<br>Lower blood loss and need for allogenic transfusion in tranexamic acid group. No DVT. 1 PE in control group.
		10 mg/kg body weight tranexamic acid in 100 mL of normal saline given as slow intravenous injection 30 min before tourniquet deflation, and the same amount 3 hours later.		No tranexamic acid and no placebo	
Sa-Ngasoongsong et al. 2013[65]<br>Thailand<br>2010-2011<br>1 hospital	Primary unilateral cemented TKR for osteoarthritis<br>45; 45; 45<br>Mean 68.1 (SD 6.2); 67.6 (8.7); 66.2 (7.3)<br>88.9%; 93.3%; 95.6%	Drain and compressive dressing			1 year<br>0; 0; 0<br>Low risk of bias<br>No separate pain outcome but WOMAC mean 14.5 (7.1); 15.1 (6.2); 15.5 (6.6). P=0.42<br>Total blood and Hb loss lower in intervention groups than control. Fewer transfusions in 500mg (0) than 250mg tranexamic acid group (6) and control group (10). 2 DVT in 500mg group. 1 DVT in 250mg group. 1 PE and 3 DVT in control group. No infections.
		25ml saline solution containing 500mg tranexamic acid injected into knee joint after fascial closure via drain tube.	25ml saline solution containing 250mg tranexamic acid injected into knee joint after fascial closure via drain tube.	25ml saline solution injected into knee joint after fascial closure	
Hourlier et al. 2015[60]<br>France<br>2009-2010<br>1 hospital	Primary unilateral TKR for osteoarthritis<br>52; 54<br>74 (SD 6); 72 (7)<br>62%; 63%	Tourniquet, electrocautery, routine haemostasis, superficial drain. No blood salvage system.			6 months<br>0; 0<br>Low risk of bias.<br>No separate pain score but KSS clinical score mean 90 (SD 6); 90 (13). P=0.90<br>No difference between groups in total blood loss. 1 MUA in single treatment
		10 mg/kg intra-operative tranexamic infusion. After 2 hours, continuous infusion of tranexamic acid 2 mg/kg/hr for 20 hours via electric syringe		single bolus of 30 mg/kg tranexamic acid as an intraoperative infusion. After 2 hours, placebo saline continuous infusion via electric syringe	

For peer review only - http://bmjopen.bmj.com/site/about/guidelines.xhtml

				group. No deep infections or revisions.
Huang et al. 2017[55]<br>China<br>2015<br>1 centre	Primary unilateral TKR for osteoarthritis<br>50; 50<br>Mean 66.2 (SD 8.3); 65.8 (6.3)<br>64%; 70%	Tourniquet inflated to 100mm Hg above SBP before incision and deflated after wound closure<br>Intravenous tranexamic acid 20mg/kg before incision and tranexamic acid 10mg/kg at 3, 6, 12 and 24 hours. 1g tranexamic acid in 50ml saline irrigated into wound during operation	No treatment with tranexamic acid	6 months<br>0; 0<br>Low risk of bias<br>VAS pain similar between groups at 6 months (p=0.728). Mean HSS score (0-100) better in tranexamic acid group than controls: 90.3 (SD 3.2); 88.9 (3.0). P<0.001. Mean difference 1.4 lower than MCID of 8.29[81]<br>Greater blood loss in control group than tranexamic group (p<0.001). DVT: 0; 0. PE: 0; 0. Intramuscular venous thrombosis: 6; 3. Superficial infection: 1; 3. Wound secretion: 6; 9.
Thrombin infusion				
Kusuma et al. 2013[62]<br>USA<br>Not stated<br>1 hospital	Primary unilateral TKR for osteoarthritis<br>40; 40<br>Mean 64.6 (SD 10.2); 64.5 (7.3)<br>82.5%; 67.5%	Tourniquet, drain, Esmarch bandage, electrocautery<br>20,000 IU thrombin infusion (1,000 IU/mL) through fascial defect	Closure and drain placement protocol without the thrombin infusion.	1 year (6 months and 2 years also reported)<br>0; 0<br>Low risk of bias<br>No separate pain outcome. KSS mean 95.5; 96.0. p=0.45<br>Lower drop in Hb in thrombin group. Blood transfusion in 4 intervention and 7 control patients. 1 control patient had haematoma. No hospital readmissions
Flexion vs extension				
Napier et al. 2014[63]<br>UK<br>2003-2004<br>1 hospital	Primary unilateral TKR of which 89% for osteoarthritis<br>90; 90	No drains or tranexamic acid<br>Flexion. Operated knee kept in passive flexion (120°) post-operatively for 6 hours using a jig. Wound redressed and placed in flexion over a	Extension. Operated knee kept in full passive extension	1 year<br>5; 1 (12 did not attend follow up)<br>Low risk of bias.<br>No separate pain outcome. OKS mean 20.5 (SD9.0); 22.1 (9.7). P=0.27

For peer review only

For peer review only - http://bmjopen.bmj.com/site/about/guidelines.xhtml

	Mean 70.4 (SD 9.9) 71.0 (7.6)<br>74%; 64%	single pillow until POD1 morning.		1 MI and 1 DVT in each group. 1 haematoma in flexion group. 1 deep infection and 1 extensor muscle weakness in extension group. More transfusions in extension group (p=0.002)
Auto-transfusion of washed blood				
Thomas et al. 2001[123]<br>UK<br>Not stated<br>1 hospital	Unilateral TKR<br>115; 116<br>Mean 69.3 (range 32-95); 70.0 (40-88)<br>62%; 53%	Allogenic transfusion if Hb fell below 9g/dl<br>Auto-transfusion of wound drainage if volume >125ml post-operative. Blood washed and re-suspended before re-infusion using a centrifugal cell washing machine	Wound drainage discarded	6 months<br>Losses to follow up not reported<br>Unclear risk of bias due to limited details of methods and follow up.<br>No separate pain outcome. No significant difference in EQ-5D between groups.<br>7% of auto-transfusion group required allogenic transfusion compared with 28% in control group. Fewer infections, readmissions and GP visits in auto-transfusion group. No significant differences in other serious adverse events or mortality between groups.

6. Platelet rich plasma

Author<br>Country<br>Recruitment dates<br>Setting	Indication<br>Number randomised intervention; control<br>Age<br>% female	Common blood conservation strategies<br>Group 1 (intervention)	 Group C (control)	Follow up<br>Losses to follow up intervention; control<br>Risk of bias issues<br>Key results
Aggarwal et al. 2014[124]	Primary unilateral surgery or first surgery of staged	Tourniquet. No tranexamic acid or suction drain. Blood transfusion if necessary due to intraoperative blood loss or postoperative haemoglobin <8g/dl.		6 months<br>No losses to follow up reported

For peer review only - http://bmjopen.bmj.com/site/about/guidelines.xhtml

India<br>2010-2011<br>1 surgeon	bilateral TKR for osteoarthritis<br>7; 14<br>Mean 56.43 (SD 7.59); 53.79 (9.75)<br>Sex not stated	8 ml PRP, prepared from patient's blood. Calcium chloride for activation given in a separate syringe in 4:1 ratio. PRP and calcium chloride injected into the posterior recess, gutters and capsule, and repaired extensor mechanism and prepatellar fat.	No treatment	High risk of bias due to unexplained differences in numbers of patients in randomised groups.<br>No separate pain outcome. WOMAC total at 6 months PRP mean 7.14 (SE 0.69), controls 7.86 (1.23), p=0.173<br>PRP group had lower fall in haemoglobin and need for blood transfusion

7. Cryotherapy

**Author**<br>**Country**<br>**Recruitment dates**<br>**Setting**	**Indication**<br>**Number randomised intervention; control**<br>**Age**<br>**% female**	**Common treatment**		**Follow up**<br>**Losses to follow up intervention; control**<br>**Risk of bias issues**<br>**Key results**
		**Group 1 (intervention)**	**Group 1 (intervention)**	
Wang 2017[125]<br>China<br>2013-2015	Unilateral TKR for osteoarthritis<br>53; 53<br>Mean 65.23 (SD 5.41); 64.97(5.36)<br>62.3%; 58.5%	CPM for 2 weeks		6 months<br>0; 0<br>Unclear risk of bias due to limited reporting<br>No separate pain outcome. At 6 months 87% of cryotherapy patients had excellent or good knee function compared with 69% of controls (p=0.032).<br>No adverse events reported in either group during functional training
		Compression cold therapy for 48 hours	No compression cold therapy	

For peer review only - http://bmjopen.bmj.com/site/about/guidelines.xhtml

8. Denusomab

Author<br>Country<br>Recruitment dates<br>Setting	Indication<br>Number randomised intervention; control<br>Age<br>% female	Common treatment<br>Group 1 (intervention)	Group 1 (intervention)	Follow up<br>Losses to follow up intervention; control<br>Risk of bias issues<br>Key results
Ledin et al. 2017[66]<br>Sweden<br>2012-2014<br>2 centres	Elective cemented primary unilateral TKR for osteoarthritis<br>25; 25<br>Mean 66 (SD 6.3); 64 (5.5)<br>60%; 60%	Injection of 60mg denusomab 1 day after surgery and after 6 months	Injection of placebo 1 day after surgery and after 6 months	12, 24 months<br>0; 2<br>Low risk of bias<br>No significant differences in KOOS pain or other KOOS domains between groups 12 12 or 24 months<br>No suspected unexpected adverse reactions in either group

9. Continuous passive motion

Author<br>Country<br>Recruitment dates<br>Setting	Indication<br>Number randomised intervention; control<br>Age<br>% female	Common treatment<br>Group 1 (intervention)	Group 2 (intervention)	Group C (control)	Follow up<br>Losses to follow up intervention; control<br>Risk of bias issues<br>Key results
Leach et al. 2006[126]<br>UK<br>Before 2005<br>1 hospital	Unilateral cruciate retaining rotating platform TKR for osteoarthritis<br>85 overall<br>Mean 71.2 (range 53-84); 72.9 (52-89)	Physiotherapy protocol from POD1 including slider board exercises to improve ROM and quadriceps strengthening exercises.<br>CPM commenced on first postoperative day set at a range 0–30 and used for 1 hour twice per day. Each day,	No CPM		6 and 12 months<br>25 patients lost to follow up<br>High risk of bias due to large loss to follow up and use of date of birth randomisation<br>No difference in mean VAS pain at 1 year, CPM 0.6; control 0.9. p=0.49

For peer review only - http://bmjopen.bmj.com/site/about/guidelines.xhtml

	50%; 54%	range was increased by 10° with discharge at POD 5-7.			Adverse events not reported
Sahin et al. 2006[127]<br>Turkey<br>Before 2006<br>1 hospital	Primary unilateral TKR for osteoarthritis<br>15; 16<br>Mean 61 (SD 6.0); 61.6 (7.5)<br>86%; 86%	Standard physiotherapy<br>From POD 1, CPM 2.5 hours 2x/day. Initially 0-40° flexion and increased by 10° each day until POD 7		No CPM	6 months<br>3 lost to follow up<br>Unclear risk of bias as patients were followed up by treating physician.<br>Mean difference in VAS pain 0.1/10 slightly favouring no CPM group (95% CI -0.8, 0.9; P=0.87)<br>Adverse events not known
Pope et al. 1997[128]<br>Australia<br>1988-1999<br>1 hospital	Primary unilateral or bilateral TKR of which 86% for osteoarthritis<br>62 (70 knees). Authors excluded those not followed up so groups were 18; 20; 19<br>Mean 72.5 (95% CI 64.4, 74.98); 72.7 (70.4, 75.0); 69.4 (64.4, 74.98)<br>64.7%; 50%; 72.2%	Physiotherapy commenced on postoperative day 1<br>Patients had an initial CPM range of 0-40° increased by 10° twice, on day after surgery and day 2, so that 0-60° flexion achieved before removal of machine at 48 hours	Patients had an initial CPM range of 0-70° increased by 10° twice, on day after surgery and day 2, so that 0-90° flexion achieved before removal of machine at 48 hours	Knee placed in an extension splint in the recovery room	6 and 12 months<br>8 patients (12 knees) excluding 1 death<br>High risk of bias due to losses to follow up and limited reporting of methods<br>No separate pain outcome. However, "pain disability" contributed up to 50 points out of a total of 70-point functional score (70 best outcome). No difference between groups in functional score: CPM 0-40 median 56 (range 20, 70); CPM 0-70 52 (10, 70); no CPM 52 (25, 70). p=0.80<br>CPM groups had greater blood loss than controls, p=0.008). 1 manipulation under anaesthesia in no CPM group, 2 revisions due to patellar dislocation in the 0-40 CPM group, 1 PE death in the 0-70 CPM group.
Beaupré etal. 2001[129]	Primary unilateral TKR of which	Standardised exercise during hospital admission which included a slider board session.			6 months

For peer review only - http://bmjopen.bmj.com/site/about/guidelines.xhtml

<table>
<tr><td>Canada
1997-1998
1 hospital</td><td>92% for osteoarthritis
40; 40; 40
Mean 68 (SD 9); 68 (9); 69 (8)
52.5%; 50%; 30%</td><td>3 sessions (2 hours) with CPM machine per day from POD2. Range increased from starting range 0-30 degrees as tolerated.</td><td>Minimum of two 10-minute slider board therapy sessions per day in addition to one in the standardised exercise. Active knee flexion and extension in sitting and lying positions performed independently as tolerated.</td><td>No intervention further than standardised exercise.</td><td>6; 8; 6
Unclear risk of bias due to losses to follow up
Mean WOMAC pain at 6 months: 76 (15); 85 (15); 79 (16). No difference over time between groups, p=0.62.
Long-term adverse events. Need for MUA: 1; 1; 0. DVT: 0; 1; 0. Cellulitis: 0; 0; 1. Infection 0; 0; 1.</td></tr>
<tr><td rowspan="2">Kumar et al. 1996[130]
USA
Before 1996
1 hospital</td><td rowspan="2">Primary unilateral TKR for osteoarthritis
40 (46 knees); 33 (37)
Mean 69 (range 52-86); 68 (42-88)
58%; 67%</td><td colspan="3">Standard physiotherapy</td><td rowspan="2">6 months
15; 13 lost to follow up
High risk of bias due to large losses to follow up
No separate pain outcome. KSS CPM 82.7; Drop and dangle 80.7. p=0.78
Haematoma 3;1. Closed manipulation 1;3. DVT 0;0. PE 0;1</td></tr>
<tr><td colspan="2">CPM from POD 0. Initially 10 hours/ day 0-90° until discharge</td><td>No CPM. Passive range of movement (“drop and dangle”) to 90° 2x/ day initially for 20 minutes, later 30-45 minutes.</td></tr>
<tr><td rowspan="2">Worland et al. 1998[131]
USA
1996
1 hospital</td><td rowspan="2">Unilateral or bilateral TKR for osteoarthritis.
91 patients (114 knees randomised). After post-randomisation exclusions: 37 (49 knees); 43 (54 knees)
Mean 70.2 (range 44-84)
66.25%</td><td colspan="3">CPM and physiotherapy during hospital admission</td><td rowspan="2">6 months
11 patients (11 knees)
Unclear risk of bias due to post-operative exclusions not reported separately for groups and limited reporting of methods.
No separate pain outcome. At 6 months, mean HSS score CPM 95.3 (SD 2.8); physiotherapy 95.7 (3.0). P=0.49.
Adverse events not reported.</td></tr>
<tr><td colspan="2">At home after discharge, CPM machine 3 hours per day on replaced knee for l0 days.</td><td>Physical therapist home visit 1 hour three times per week for 2 weeks</td></tr>
</table>

For peer review only - http://bmjopen.bmj.com/site/about/guidelines.xhtml

MacDonald et al. 2000[132]<br>Canada<br>Before 2000<br>1 hospital	Primary unilateral TKR for osteoarthritis<br>40; 40; 40<br>Age and sex not reported	Active ROM, passive ROM exercises, mobilised as tolerated using walker or crutches.			6 and 12 months<br>Not reported<br>Unclear risk of bias due to limited and selective reporting.<br>No separate pain outcome. No statistical differences between groups for KSS at 6 and 12 months.<br>Adverse events not reported
		CPM commenced POD 0. Initially 0-50 degrees. Provided for 18-24 hour/ day. Increased by 10 degrees/ hour as tolerated. Continued until POD 1	CPM commenced POD 0. Initially 70-110 degrees. Provided for 18-24 hr/ day. Not increased.<br>Continued until POD 1	No CPM	
Bennett et al. 2005[67]<br>Australia<br>1997-2000<br>1 hospital	Primary unilateral TKR for osteoarthritis<br>47; 48; 52<br>70.7; 71.4; 71.7<br>72.3%; 64.6%; 67.3%	Standard in hospital physiotherapy programme			12 months<br>1 patient excluded due to inability to achieve 90° flexion<br>Low risk of bias<br>No separate pain outcome. No significant difference in KSS between groups at 1 year.<br>No difference in wound healing between groups
		Standard CPM from 0° to 40° for 2x3 hours on POD 1 increased by 10° per day until POD 6. Extension splint applied overnight	Early flexion CPM commenced in recovery room from 90° to 50° knee flexion. Increased gradually to CPM 90° to 0° for 2x3 hours in day 4-6.	No CPM	
Ersözlü et al. 2009[68]<br>Turkey<br>2003-2004	Primary unilateral TKR for osteoarthritis<br>30; 30; 30<br>Mean 65 (range 54-73); 61 (49-80); 62 (52-78)<br>66%; 55%; 57%	Conventional physical therapy			2 years<br>1; 1; 2<br>Low risk of bias<br>No separate pain outcome. KSS scores 98; 95; 92. No significant difference between groups p=0.67.<br>Infection 0; 0; 1. Arrhythmia 0; 1; 0. No difference in complications between groups
		CPM set at 30-40° from POD1. Increased as tolerated to POD7. 1 hour CPM 3x/day.	CPM set at 60-70° from POD3. Increased by 10°/ day to POD7. 1 hour CPM 3x/day.	No CPM	

10. Electrical stimulation

Author	Indication	Common rehabilitation strategies	

For peer review only - http://bmjopen.bmj.com/site/about/guidelines.xhtml

Country<br>Recruitment dates<br>Setting	Number randomised intervention; control<br>Age<br>% female	Intervention	Intervention	Common rehabilitation strategies
Avramidis et al. 2011[69]<br>Greece<br>2005-2006<br>1 hospital	Elective primary unilateral TKR for osteoarthritis<br>38; 38<br>Mean 70.54 (SD 4.68); 70.66 (3.73)<br>80%; 82.9%	Standard physiotherapy for 6 weeks. No CPM		1 year<br>3 (intervention intolerance); 3<br>Low risk of bias<br>Improved SF-36 bodily pain at 1 year in intervention group compared with control, mean 92 (SD 10.57); 79.48 (12.72). P<0.001. Difference of 12.52 close to MCID of 16.86[82]. No difference in OKS or American KSS<br>Adverse events not reported
		Transcutaneous electric muscle stimulation of the vastus medialis muscle from POD2 2x/ day for 2 hours for 6 weeks.	No intervention	
Stevens-Lapsley et al. 2012[133]<br>USA<br>2006-2010<br>1 hospital	Primary unilateral TKR for osteoarthritis<br>35; 31<br>Mean 66.2 (SD 9.1); 64.8 (7.7)<br>57.1%; 51.6%	Standard inpatient rehabilitation, home and outpatient physical therapy		6 months and 1 year<br>5; 6<br>Unclear risk of bias due to baseline differences in WOMAC<br>No difference in resting pain (points) at 1 year intervention mean 0.6 (SD 1.4); control 0.4 (1.5). Also similar at 6 months. Mean WOMAC total score better at 1 year in intervention group compared with control, 5.7 (5.9); 10.0 (12.2) and at 6 months. However, probably explained by baseline differences. Authors state no differences for change in WOMAC.<br>DVT 1; 0. Unspecified complication 1; 0. Infection 0; 2. Revision 0; 1
		Neuromuscular electrical stimulation commenced on POD2 for 6 weeks 2x/ day.	No intervention	

For peer review only

For peer review only - http://bmjopen.bmj.com/site/about/guidelines.xhtml

Levine et al. 2013[134]<br>USA<br>Before 2013<br>1 surgeon	Elective unilateral TKR for osteoarthritis<br>35; 35<br>Mean 68.1; 65.1<br>76%; 62%	2 sessions of ROM exercise		6 months<br>5; 9<br>Unclear risk of bias due to large uneven losses to follow up<br>KSS pain favoured intervention at 6 months but not significantly 79.08 (SD 10.97); 75.5 (14.77); 95%CI for difference -3.78, 10.93. Similar for WOMAC total score, 95%CI for difference -3.19, 14.81.<br>Confusion 2; 0
		Neuromuscular electrical stimulation commenced 14 days pre-operatively until 1 day before surgery. Recommenced at POD1 for 60 days. After hospital discharge no direct contact with a physical therapist	Formal physical therapy programme with progressive resistive exercises and strengthening in hospital and after discharge supervised by physical therapist.	
Moretti et al. 2012[70]<br>Italy<br>2008-2010<br>1 hospital	Primary unilateral TKR for osteoarthritis<br>15; 15<br>Mean 70.0 (SD 10.6); 70.5 (8.1)<br>Not reported	Rehabilitation protocol including CPM		6 and 12 months<br>No losses to follow up<br>Low risk of bias<br>Mean VAS pain (10-point scale) lower at 12 months in intervention group compared with control, 0.5 (SD 1.3); 3.6 (3.9). p< 0.05. Mean difference of 2.1 (10-point scale) greater than MCID of 16.1 (100-point scale)[83]<br>Difference also at 6 months.<br>More swelling of the knee in intervention patients than controls, statistically significant at 1 and 2 months
		Pulsed electromagnetic fields (I-ONE therapy) from POD7, 4 hours/ day for 60 days	No intervention	
Adravanti et al. 2014[135]<br>Italy<br>1 hospital	Primary unilateral TKR for osteoarthritis<br>16; 17<br>Mean 66 (SD 13); 73 (5)<br>62.5%; 52.9%	Standard rehabilitation protocol: active and passive mobilisation		6 months<br>4; 3<br>High risk of bias: small study, proportionately high losses to follow up<br>At 6 months, mean VAS pain in intervention group lower than in controls (p<0.05). At 3 years, 1/14 intervention patients and 4/12 controls reported severe pain
		Pulsed electromagnetic fields (I-ONE therapy) by POD7 for 4 hours/ day for 60 days	No intervention	

For peer review only

For peer review only - http://bmjopen.bmj.com/site/about/guidelines.xhtml

				No difference between groups in swelling at 6 months.

11. Rehabilitation

Author<br>Country<br>Recruitment dates<br>Setting	Indication<br>Number randomised intervention; control<br>Age<br>% female	Common rehabilitation strategies		Follow up<br>Losses to follow up intervention; control<br>Risk of bias issues<br>Key results
		**Intervention**	**Control**	
Walking guidance and training				
Li et al. 2017[71]<br>China<br>2015-2016<br>1 hospital	Primary unilateral TKR for osteoarthritis<br>43; 43<br>Mean 76.33 (SD 5.28); 78.47 (5.50)<br>55.8%; 51.2%	Before TKR, general guidance on joint activities, quadriceps muscle strength, use of aids, diet guidance, correct walking methods and precautions.<br>Knee passive flexion and extension to 90° and quadriceps muscle strength training commenced on POD 1. POD 3-7, straight leg raising exercises. 2 weeks after replacement, increased joint activities and muscle strength training, centre of gravity transfer training, limb weight training, and walking training.		6 months<br>0; 0<br>Low risk of bias<br>Mean VAS pain at 6 months: 0.51 (SD 0.74); 2.83 (0.88) favouring walking intervention group, p<0.01. Difference of 2.42 (10 point scale) greater than the MCID of 16.1 (100-point scale)[83]. HSS scores at 6 months favoured intervention, p<0.01.<br>No infection, allergic reaction or immune reaction in either group. Intervention not associated with swelling, pain, prosthesis loosening, thrombosis, or delayed wound healing
		Standing, weight and balance exercises from POD 1. From POD 2, walking guidance and training.	No additional rehabilitation	
Aquatic therapy				
Liebs et al. 2012[72]<br>Germany<br>2003-2004<br>4 hospitals	Elective primary unilateral TKR for osteoarthritis<br>87;98	Continuous passive motion machines daily after removal of suction drains. Programme of daily physiotherapy: range of motion activities; exercises for improvement of muscle tension, venous return, balance, coordination and gait; and instruction in activities of daily living.		6, 12 and 24 months<br>13.8%; 19.4% excluding deaths and unexplained reasons<br>Low risk of bias

For peer review only - http://bmjopen.bmj.com/site/about/guidelines.xhtml

<table>
<tr><td rowspan="2"></td><td rowspan="2">Mean 68.5 (SD 8.6); 70.9 (7.5)
70.1%;73.5%</td><td colspan="3">Aquatic therapy for 30 minutes 3 times a week up to postoperative week 5. Pool exercises aimed at training of proprioception, coordination and strengthening with aid of float cuffs, training kickboards and bar floats.</td><td rowspan="2">WOMAC pain at 12 months: early aquatic mean 13.2 (SD 15.0); late aquatic 17.4 (22.4) p=0.22. No difference at 6 and 24 months.
5 early aquatic therapy patients and 1 late aquatic therapy patient readmitted to hospital within 3 months. 2 early aquatic patients and 1 late aquatic patient readmission directly or indirectly related to the intervention.</td></tr>
<tr><td colspan="2">Aquatic therapy beginning on the 6th postoperative day with the wound covered with a waterproof adhesive dressing.</td><td>Aquatic therapy as pool exercise after the completion of wound healing on the 14th postoperative day</td></tr>
<tr><td rowspan="2">Rahmann et al. 2009[136]
Australia
2003-2005
1 hospital with 2 surgeons</td><td rowspan="2">Unilateral primary TKR or THR for osteoarthritis (50% TKR)
18;19;17 (11 had been excluded post-randomisation due to complications in hospital
Mean 69.4 (SD 6.5); 69.0 (8.9); 70.4 (9.2)
44.4%; 63.2%; 70.6%</td><td colspan="3">Standard ward-based physiotherapy until day 3. 1 ward physiotherapy treatment per day. Surgical wounds covered with an occlusive, waterproof dressing. 40 mins/ day.</td><td rowspan="2">6 months
4;2;0 for combined THR and TKR
Unclear risk of bias as TKR patients more likely to receive ward-based control intervention. THR and TKR analysed together
No difference in overall WOMAC outcome at 6 months in THR and TKR patients combined between aquatic at fast pace and ward-based (p=0.929) and aquatic at 2 paces (p=0.872).
No adverse events reported after intervention commenced.</td></tr>
<tr><td>From day 4, 1 to 1 individual physiotherapy. Aquatic physiotherapy programme to maximize function and strength. 40 mins/ day. Fast pace metronome 80-88 bpm</td><td>From day 4, 1 to 1 individual physiotherapy. Water exercise programme with general exercises not targeted at specific functional retraining in the aquatic environment. Slow pace metronome 50-58 bpm</td><td>From day 4, 1 to 1 individual ward-based physiotherapy. 40 mins/ day</td></tr>
<tr><td colspan="6">Supported early discharge</td></tr>
<tr><td rowspan="2">Mahomed et al. 2008[137]
Canada
2000-2002
2 centres</td><td rowspan="2">Primary unilateral TKR or THR for osteoarthritis (approximately 50% TKR)
119;115
68</td><td colspan="3">Inpatient physiotherapy</td><td rowspan="2">12 months
No losses to follow up
Low risk of bias (analysis by actual treatment received showed similar results)
WOMAC pain at 12 months marginally favoured home-based</td></tr>
<tr><td colspan="2">Discharged home when able to independently transfer supine to sitting and sitting to standing, walk 30 metres and climb stairs if necessary. Physiotherapist home visit within 48 hours and</td><td>Transfer to independent rehabilitation centre for 14 day stay.</td></tr>
</table>

For peer review only

For peer review only - http://bmjopen.bmj.com/site/about/guidelines.xhtml

	About 67% women	subsequent management along a multidisciplinary clinical pathway (4-16 visits). Then outpatient physiotherapy or self-directed programme.		rehabilitation mean 87 (SD 16); 83 SD (20), p=0.08 but this was not statistically significant. Mean difference of 4 less than MCID of 8-9[77]. Results did not differ between TKR and THR patients.<br>Similar rates of dislocation, DVT and readmissions between groups. 2% inpatient group developed infections compared with 0 in home group
Hill et al. 2000[138]<br>UK<br>1997-1998<br>1 centre	Unilateral, primary TKR, irrespective of diagnosis or concomitant disease<br>70 randomised, with 32;28 eligible for trial at day 5	Care pathway for medical, nursing and physiotherapy care from admission until day 5<br>Outreach team domiciliary visit prior to admission with assessment of home environment. At days 5–7, patients assessed to ensure discharge safe. Outreach team visit on day of discharge with further visits as required. 1+ physiotherapist visit linked with nurses to monitor knee performance. Discharge when skin clips removed, wound healed and specialist orthopaedic assistance not required, usually day 10–12	Inpatient care until removal of skin clips and wound healing.	1 year<br>No losses to follow up reported after commencement of intervention<br>Unclear risk of bias due to limited reporting of methods.<br>No pain outcome or patient reported outcome. Control group had better mean KSS scores, but this did not reach statistical significance at 1 year or earlier.<br>1;1 serious infection, other wound infections 1;6, painful joints 9;4, other minor complications similar between groups
Flexion or extension during knee closure				
Wang et al. 2014[74]<br>China<br>2009-2010<br>1 centre	Primary unilateral TKR for osteoarthritis<br>40; 40<br>Mean 68.34 (SD 7.09), 67.87 (6.47)<br>17.5%; 22.5%	No patellar replacement or lateral retinacular release<br>Articular capsule, soft tissue and skin enclosed in 90° flexion which was maintained for 1-2 min after wound closure.	Wound closure performed in full extension	6 months<br>No losses to follow up<br>Low risk of bias<br>Mean VAS pain in flexion group 1.15 (SD 0.73); extension group 1.12 (0.68), p=0.64

For peer review only - http://bmjopen.bmj.com/site/about/guidelines.xhtml

				No wound complications, patella fracture or infection requiring surgery in either group

12. Wound management

Author<br>Country<br>Recruitment dates<br>Setting	Indication<br>Number randomised intervention; control<br>Age<br>% female	Common wound management strategies		Follow up<br>Losses to follow up intervention; control<br>Risk of bias issues<br>Key results
		Intervention	Control	
Kong et al. 2014[75]<br>South Korea<br>2011<br>1 surgeon	Primary cemented unilateral TKR for osteoarthritis<br>50; 50<br>Mean 69.0 (SD 7.7); 68.0 (4.8)<br>89.6%; 87.5%	Skin staples removed on day 10 and wound closure strip applied for 5 days		6 and 12 months<br>2; 2 lost to follow up<br>Low risk of bias<br>At 12 months, VAS pain in silicone gel group mean 2.50 (SD 1.16); control 2.92 (1.90). P=0.201. No difference at 6 months, p=0.886.<br>No wound dehiscence or infection associated with application of silicone gel or petroleum
		After removal of wound closure strip, patients managed operation scars with application of silicone gel for 1 month after stitches removed	After removal of wound closure strip, patients managed operation scars with application of petroleum gel for 1 month after stitches removed	

13. Anabolic steroids

Author<br>Country<br>Recruitment dates<br>Setting	Indication<br>Number randomised intervention; control<br>Age<br>% female	Common rehabilitation strategies		Follow up<br>Losses to follow up intervention; control<br>Risk of bias issues<br>Key results
		Intervention	Control	
		Cold compression and CPM		6, 9 and 12 months

For peer review only - http://bmjopen.bmj.com/site/about/guidelines.xhtml

Hohmann et al. 2010[76]<br>Australia<br>Before 2010<br>1 surgeon	Primary unilateral TKR for osteoarthritis<br>5; 5<br>Mean 66.2 (range 58, 72); 65.2 (59, 72)<br>20%; 40%	On day 5, intramuscular injection of 50 mg Nandrolone decanoate solution. Patients visited every 2 weeks and injections continued for 6 months.	On day 5, intramuscular injection of saline. Patients visited every 2 weeks and injections continued for 6 months.	0; 0 lost to follow up<br>Low risk of bias (but small feasibility study)<br>No separate pain outcome. KSS at 12 months in intervention group mean 91.4 (SD 3.5); control 81.2 (SD 7.1). p=0.03. Difference also at 6 months (p=0.04), marginal at 9 months (p=0.06). Difference in means at 12 months of 10.2 close to MCID of 12.3[79].<br>Intervention group had smaller decrease in bone mineral density at 6 months than controls but not significant

14. Guided imagery

**Author**<br>**Country**<br>**Recruitment dates**<br>**Setting**	**Indication**<br>**Number randomised intervention; control**<br>**Age**<br>**% female**	**Common rehabilitation strategies**		**Follow up**<br>**Losses to follow up intervention; control**<br>**Risk of bias issues**<br>**Key results**
		**Intervention**	**Control**	
Jacobson et al. 2016[139]<br>USA<br>2011-2012<br>1 surgeon	Primary unilateral TKR for osteoarthritis<br>42; 40 (41; 39 received treatment)<br>Mean 65.0 SD 8.6)<br>62.2%	Participants listened to a 19-21-minute CD each day for 2 weeks before and 3 weeks after surgery. Content covered concerns and hopes about TKR with aim to facilitate mind–body connections to promote optimal TKR outcomes.	Participants listened to a 17-21-minute CD each day for 2 weeks before and 3 weeks after surgery. Content comprised poetry, short stories and essays	6 months<br>12; 10 of patients receiving treatments<br>High risk of bias due to large losses to follow up<br>Mean WOMAC pain 2.7 (SD 3.1); 3.5 (SD 3.3). P<0.001<br>Adverse events not reported

For peer review only - http://bmjopen.bmj.com/site/about/guidelines.xhtml

CD compact disc; CPM Continuous passive motion; DN4 Douleur Neuropathique 4; FNB Femoral nerve block; HSS Hospital for Special Surgery; i.v. intravenous; KOOS Knee injury and Osteoarthritis Outcome Score; KSS Knee Society Score; LIA local infiltration analgesia; NRS Numerical rating scale; OKS Oxford Knee Score; ONB obturator nerve block; PCA Patient controlled analgesia; PNB psoas nerve block; SF-36 Short Form 36 Health Survey; S-LANSS Leeds assessment of Neuropathic Symptoms and Signs Pain Scale; SNB Sciatic nerve block; TKR Total knee replacement; VAS Visual analogue scale; VTE Venous thromboembolism; WOMAC Western Ontario and McMaster Universities Osteoarthritis Index.

ITT, ITT CC, POD, MI, PE

For peer review only

For peer review only - http://bmjopen.bmj.com/site/about/guidelines.xhtml

Supplementary material. Risk of bias assessment

Study	Random sequence generation	Allocation concealment	Blinding of participants and personnel	Blind outcome assessment	Incomplete outcome data	Selective reporting	Other bias	Summary
Pain management								
Albrecht et al. 2014[34]	Computer generated	Anaesthetist blind to allocation	Physiotherapists, surgeons, research assistants collecting data, and members of the Acute Pain Service were kept blinded to group allocation.	Physiotherapists, surgeons, research assistants collecting data, and members of the Acute Pain Service were kept blinded to group allocation.	ITT analysis low losses to follow up	No but not checked protocol	Study was terminated early with 61% of planned recruitment completed due to change in standard anaesthesia at hospital	Low
Anastase et al. 2014[113]	Method of the Ulm Institute of Statistics	Method of the Ulm Institute of Statistics	No	No	15:14 lost to follow up	No but not checked protocol	ASA comorbidities differed between groups	High
Aveline et al. 2014[52]	Computer generated	opaque sealed envelopes	Blinded syringes prepared by nurse not involved in study	Yes	Low losses to follow up	Consistent with short term follow up paper	No	Low
Bergeron et al. 2009[105]	Blocks of different sizes according to list preprepared by study epidemiologist	Not described	Anaesthetist not blind. Patients blind	Nurse observers collecting data blind to allocation	32/59 lost to follow up	No but not checked protocol	No	High
Buvanendran et al. 2010[53]	computer generated	Yes, physicians and nurses blind	Yes	Yes	ITT	Protocol not checked	No	Low

Choy et al. 2011[35]	Computer generated	sealed envelope	No, the catheter was removed at either day 3 or 7	Patient reported outcome. Other outcomes by blinded independent physician	Low losses to follow up	Protocol not checked but reasonable range of outcomes	No	Low
Davidson et al. 2016[111]	Computer generated	Sealed opaque envelopes	Subjects and investigators were not masked to treatment group	Subjects and investigators were not masked to treatment group. PROM	31; 29 lost to follow up	None apparent but protocol not checked	Combined data from 2 RCTs	High
Fan et al. 2016[36]	No details	sealed opaque envelopes	Patients and assessors blind to randomisation	Patients and assessors blind to randomisation	2% protocol violation	No but not checked protocol	No	Low
Foadi et al. 2017[117]	Computer generated	Not described	Not described	Patient reported outcome	>70% questionnaire return	None apparent but protocol not checked	Described as pilot study	Unclear
Gao et al. 2017[37]	Random number table	Not described	Blind to patients	Blind to observers	2; 1; 0	None apparent but protocol not checked	Groups similar at baseline	Low
Ilfeld et al. 2009[108]	Computer generated	Investigators, patients, and all clinical staff were unaware of treatment group assignments	Investigators, patients, and all clinical staff were unaware of treatment group assignments	Investigators, patients, and all clinical staff were unaware of treatment group assignments	4:1 lost to follow up	No but not checked protocol	Basal infusion halved on POD1 in 10 intervention patients compared with 3 controls	High
Ilfeld et al. 2011[109]	Computer generated tables	Solutions prepared by investigational pharmacist	Yes. Intervention and control solutions indistinguishable	Patient reported outcomes. Staff masked to treatment group	11;12 did not have 4 measures out	Protocol not checked	WOMAC and WOMAC domain scores	High

For peer review only - http://bmjopen.bmj.com/site/about/guidelines.xhtml

				assignment performed all measures and assessments	of 6 up to 12 months	but seems reasonable	somewhat lower pre-intervention in extended infusion group. Authors report change scores	
Macrinici et al. 2017[38]	Computer generated	Staff performing injections blind	Anaesthesiologist, surgeons, patients and physical therapists blind to allocation	Yes	3; 4 lost to follow up. 6; 3 complications	None apparent but protocol not checked	Groups similar at baseline	Low
McDonald et al. 2016[45]	Computerised blocked	Study coordinator independent of care and surgery	Patients blind. Surgeon aware of study	Outcome assessment blind to allocation	1; 4 unexplained	None apparent, protocol not checked	Groups similar at baseline	Low
Meunier et al. 2007[51]	Computer generated	Sealed envelope	Randomisation code broken after 1 year	Yes	ITT reported except for 12 month pain outcome	No but not checked protocol	M/F ratio differed	Low
Morin et al. 2005[110]	Allocated randomly	Sealed envelope	All patients received some form of nerve block. Anaesthesiologist not blind to intervention	Observers not blinded	Per protocol analysis	No but not checked protocol	Difference between groups in anesthetist's opinion of difficulty of catheter placement. BMI differed between groups	High
Motififard et al. 2017[46]	Computer generated	Study coordinator independent of care and surgery	Patients blind. Surgeon aware of study	Outcome assessment blind to allocation	3; 7 (1; 4 unexplained)	None apparent, protocol not checked	Groups similar at baseline	Low

For peer review only - http://bmjopen.bmj.com/site/about/guidelines.xhtml

Nader et al. 2012[39]	Computer generated	Opaque envelope	No	Patient reported outcome	1:1 lost to follow up	Protocol not checked but reasonable range of outcomes	FNB group somewhat higher BMI	Low
Niemeläinen et al. 2014[47]	No details	Opaque sealed envelopes	Only research nurse preparing infiltrate aware of randomisation. All other personnel unaware until after 1 year follow up	Only research nurse preparing infiltrate aware of randomisation. All other personnel unaware until after 1 year follow up	All patients who received intervention completed follow up	No but not checked protocol	No	Low
Peng et al. 2014[40]	Computer generated	Not possible	Not possible	Patient reported outcome	31:38 lost at 12 months but ITT and per-protocol analysis	No but not checked protocol	No	Low
Perrin and Purcell 2009[107]	No details	Sealed syringe code stored in pharmacy department	yes	Yes	4 failed to complete protocol	No but not checked protocol	Pilot investigation. High risk of bias due to recruitment difficulties leading to small trial	High
Reinhardt et al. 2014[41]	Computer generated	Maintained by pharmacy department for blinding	Patients blind to intervention	Blinded research assistant and partially physical therapist	0 reported lost to follow up of those who received interventions	No but not checked protocol	No	Low
Seah et al. 2011[48]	Randomisation tables	Sealed envelopes. Anaesthetist and surgeon blind before opening of	Blinding of patients not stated	Blind outcome assessors and PROMs	No losses to follow up reported	No but not checked protocol	No	Low

For peer review only - http://bmjopen.bmj.com/site/about/guidelines.xhtml

		sealed envelope						
Shum et al. 2009[106]	No details	No details	Anaesthetist performing the blocks was not involved in the postoperative follow-up and data collection	Patient reported	14% and 20%	No but not checked protocol	Mean patient weight lower in no FNB group. More favourable mean OKS in no FNB group. Two groups combined for 2 year outcome but not for earlier	High
Spreng et al. 2012[115], Spreng et al. 2010[116]	Hospital pharmacy	Epidural catheter or sham set-up taped along the back of the patient and connected to an infusion pump covered in an opaque bag. Also sham knee catheter	Patients blind	Blind outcome assessment	13%	Limited reporting in conference abstract	Conference abstract only so limited information additional to early follow up paper	Unclear
Wang et al. 2015[112]	No details	No details	Not stated	Not stated	2:4 lost to follow up	No but not checked protocol	No	Unclear
Wegener et al. 2013[42]	No details	Opaque envelope	Patients, surgeons and researchers not blind to intervention	Patients not blinded	2:7:5 lost to follow up	No. Protocol checked	no	Low
Widmer et al. 2012[43]	Coded envelope	Coded envelope	Except for anaesthetist and surgeon	Both the investigators and patients were blinded	None reported as incomplete	No but protocol not checked	No	Low

For peer review only - http://bmjopen.bmj.com/site/about/guidelines.xhtml

Williams et al. 2013[49]	Computer generated	Not stated	Patients and assessors blind	Patients and assessors blind	3:1 of those who received treatment	No but not checked protocol	No	Low
Wu and Wong 2014[44]	Computer	Sealed envelopes	No	No	Available cases	No but not checked protocol	No	Low
Wylde et al. 2015[50]	Trials unit	Trials unit	Surgeon and anaesthetist not blind to allocation, Patients blind	Patients and research nurses blind to allocation	ITT with imputed data	No as per protocol	No	Low
Yue et al. 2013[114]	No details	No details	Surgeons and patients were double-blinded to the injection administered	surgeons and patients were double-blinded to the injection administered	Losses to follow up not reported	Limited reporting	No	Unclear
Myofascial trigger point dry needling								
Mayoral et al. 2013[118]	Computerised	Not described	Patient and other researchers apart from physical therapist blind	Patient outcomes	4: 5 loss to follow up	None apparent but protocol not checked	No	High
Tourniquet								
Abdel-Salam and Eyres 1995[119]	Card system	Not described	No	No	No losses to follow up	None apparent but protocol not checked	No	Unclear
Ejaz et al. 2014[54]	Block randomised	Sealed envelopes	Patients unaware	PROM	No losses to follow up of those who received treatments	None apparent but protocol not checked	No	Low
Huang et al. 2017[55]	Computer generated	Sealed opaque envelopes	Surgeons not blind. Patients blind to allocation	Data collector blind to allocation	No losses to follow up reported	None apparent but protocol	Groups similar at baseline	Low

						not checked		
Liu et al. 2014[56]	Excel	Not described	Patients blind	PROM	No losses	None apparent but protocol not checked.	No	Low
Mittal et al. 2012[57]	Computer generated	Sealed opaque envelopes	Patient blind	Outcome assessors blind. PROM	5:2	None apparent but protocol not checked	Study stopped because of high risk of transfusion in short tourniquet duration group	Low
Şükür et al.2016[120]	Computer generated	Not described	Possibly patients	Outcome assessors blind	No losses to follow up	KSS outcome noted in methods but not presented in results	No	High
Zhang et al. 2017[58]	Excel	Randomisation by blinded researcher.	Patients and nurses on ward blind	Not clear	No losses reported	None apparent but protocol not checked	Groups similar at baseline	Low
Zhang et al.2016[121]	Randomly allocated	Not clear	Not clear	Not clear	Not clear	HSS outcome noted in methods but not presented in results	No	High
Compression bandage								

For peer review only - http://bmjopen.bmj.com/site/about/guidelines.xhtml

Brock et al. 2017[122]	Web-based	Not specified	Not possible	No but PROMs	4; 2 of those receiving intervention	None apparent but protocol not checked	Groups similar at baseline	Low
Blood conservation								
Hourlier et al. 2015[60]	Computer generated	Opaque envelopes	Anaethsetist, surgeon and patient blind to treatment allocation	Assessors blind	No missing data	None apparent but protocol not checked	No	Low
	Computer generated	Sealed opaque envelopes	Surgeons not blind. Patients blind to allocation	Data collector blind to allocation	No losses to follow up reported	None apparent but protocol not checked	Groups similar at baseline	Low
Kim et al. 2014[61]	Computer generated	Not stated	patients blind to allocation	Clinical investigator blind to allocation	No missing data	None apparent but protocol not checked	No	Low
Kusuma et al. 2013[62]	Computer generated	Sealed envelopes	Surgeon and patient blind	Outcome assessor blind	No missing data	None apparent but protocol not checked	No	Low
Napier et al. 2014[63]	Computer generated	Sealed envelopes	Unlikely	Not stated but PROM	low losses to follow up	None apparent but protocol not checked	No	Low
Sa-Ngasoongsong et al. 2011[64]	Computer generated	Sealed envelopes	Surgeon and patient blind	Outcome assessor blind	No missing data	None apparent but	No	Low

For peer review only - http://bmjopen.bmj.com/site/about/guidelines.xhtml

						protocol not checked		
Sa-Ngasoongsong et al. 2013[65]	Computer generated	Sealed envelopes	Surgeon and patient blind	Outcome assessor blind	No missing data	None apparent but protocol not checked	Some difference between groups in pre-operative Hb	Low
Thomas et al. 2001[123]	Not described	not stated	Not reported	Not reported but PROM	Not reported but ITT	None apparent but protocol not checked	No	Unclear
Platelet rich plasma								
Aggarwal et al. 2014[124]	Not described	Opaque envelopes	Patients blind	Patients and examiners blind	No losses to follow up reported	None apparent but protocol not checked	Odd numbers in groups from randomisation	High
Cryotherapy								
Wang 2017[125]	No details	No details	No details	No details	No losses to follow up	None apparent but protocol not checked	Groups similar at baseline	Unclear
Denusomab								
Ledin et al. 2017[66]	Randomisation list produced by the study monitor	Syringes prepared independently	Investigators and patients blind	Unblinding was done after all the data had been locked	0; 2	None apparent but protocol not checked	No	Low
Continuous passive motion								
Beaupré etal. 2001[129]	Computer generated	Sealed envelopes	No	Researcher unaware and PROMs	6:8:6. Results carried	No	4 controls; 1 SB	Unclear

For peer review only - http://bmjopen.bmj.com/site/about/guidelines.xhtml

					forward for missing data		reassigned to CPM	
Bennett et al. 2005[67]	Block	Not stated	Operating surgeon blind. Patient not	Independent assessor blind	1 not included in analyses as not able to achieve 90 degree flexion	No	No	Low
Ersözlü et al. 2009[68]	Divided into groups by random selection	Not described	No	Surgeon score	A diabetic patient from the control group was excluded because of a superficial wound infection, a patient with a cardiac problem in group II due to dysrhythmia, and two patients due to insufficient follow-up.	Not apparent	No differences baseline	Unclear
Kumar et al. 1996[130]	Random number generator	Not stated	No	Not described	Large loss to follow up	Not all data clearly reported	No	High
Leach et al. 2006[126]	Allocation by date of birth	No	No	Blinded evaluation	Large loss to follow up	No	No	High
MacDonald et al. 2000[132]	Computer generated	Allocation concealed	No	Not described	Not reported	Yes, not all outcomes reported in full	No	Unclear
Pope et al. 1997[128]	Not described	Not described	Not described	Not described	No separate reporting. 8 patients (12 knees)	None apparent but protocol	No	High

					excluding 1 death	not checked		
Sahin et al. 2006[127]	Not described	Not stated	No	Followed up by treating physician	Low loss to follow up	No	No	Unclear
Worland et al. 1998[131]	Not described	Not described	No	Researcher blind	Not reported separately	None apparent but protocol not checked	No	Unclear
Electrical stimulation								
Adravanti et al. 2014[135]	Computer generated	Not described	Research assistant not involved in patient assessment	Principal investigator and all physicians in charge of clinical controls were blinded to patient allocation	78% retained at 6 months	Not apparent but protocol not checked	No	High
Avramidis et al. 2011[69]	Computer generated	Not described	No	Independent assessors blind	3 (intolerance of intervention); 3	Not apparent	Baseline similar	Low
Levine et al. 2013[134]	Drawing papers from hat	Not described	No	Not described. WOMAC PROM	5:9 for KSS pain and WOMAC	Not apparent but protocol not checked	No	Unclear
Moretti et al. 2012[70]	Computer generated	Not described	Physicians, as well as medical assessors, were blinded to the allocation of patients in the study groups	Physicians, as well as medical assessors, were blinded to the allocation of patients in the study groups	No losses reported	Not apparent but protocol not checked	No	Low
Stevens-Lapsley et al. 2012[133]	Stratified	Concealed	No	no but standardised scripts used	5; 6	Not apparent but protocol	WOMAC, BMI unequal at baseline	Unclear

For peer review only - http://bmjopen.bmj.com/site/about/guidelines.xhtml

						not checked		
Rehabilitation								
Hill et al. 2000[138]	Not described	Not stated	Not possible	Not described	No losses to follow up after initial 23222	No but protocol not checked	No	Unclear
Li et al. 2017[71]	Random number table	Not stated	Not possible	Not described but PROM	No losses to follow up	No but protocol not checked	Groups similar at baseline	Low
Liebs et al. 2012[72]	Computer generated	Allocation concealed	Not possible	No but PROM	Low losses to follow up (<20% if deaths and other explained reasons not counted)	No but protocol not checked	No	Low
Mahomed et al. 2008[137]	Block randomisation	Not stated	Not possible	PROM	No loss	No but protocol not checked	ITT gave similar results to analysis according to actual discharge destination (20 inpatient group received home based)	Low
Rahmann et al. 2009[136]	Not described	Sealed numbered envelopes	Not possible	Assessor blind to intervention. Patient reported outcome	Low losses to follow up	No but protocol not checked	TKR patients more likely to receive ward-based control intervention. THR and TKR analysed together	Unclear
Wang et al. 2014[74]	Computer generated	Surgeons did not participate	Surgery was performed by the	Postoperative evaluation was	No loss to follow up	None apparent	No baseline differences	Low

For peer review only - http://bmjopen.bmj.com/site/about/guidelines.xhtml

		in pre-operative grouping	physicians who did not participate in the preoperative grouping and postoperative evaluation	conducted by the physicians who were unaware of the grouping.		but protocol not checked		
Wound management								
Kong et al. 2014[75]	Not described	Not described	Placebo used	Patient outcome	Low loss to follow up	None apparent but protocol not checked	Similar at baseline	Low
Anabolic steroids								
Hohmann et al. 2010[76]	Internet based	Not reported	Placebo trial	Double-blind design minimized systemic error and eliminated observer and experimenter's bias	0 loss to follow up	None apparent	None apparent but small study	Low
Guided imagery								
Jacobson et al. 2016[139]	Permuted blocks	Opaque CD holders	Personnel yes, participants no	Yes	12; 10 of patients receiving treatment	None apparent but protocol not checked	No	High

For peer review only - http://bmjopen.bmj.com/site/about/guidelines.xhtml

References to all RCTs of peri-operative interventions with long-term pain or score follow up, irrespective of risk of bias assessment (numbering consistent with main article)

34. Albrecht E, Morfey D, Chan V, et al. Single-injection or continuous femoral nerve block for total knee arthroplasty? *Clin Orthop Relat Res* 2014;472:1384-93.
35. Choy WS, Lee SK, Kim KJ, et al. Two continuous femoral nerve block strategies after TKA. *Knee Surg Sports Traumatol Arthrosc* 2011;19:1901-8.
36. Fan L, Yu X, Zan P, et al. Comparison of local infiltration analgesia with femoral nerve block for total knee arthroplasty: A prospective, randomized clinical trial. *J Arthroplasty* 2016;31:1361-5.
37. Gao WL, Li H, Liu BQ, et al. Analgesic effect of femoral and sciatic nerve block under multimodal analgesia in total knee arthroplasty. *Chinese Journal of Tissue Engineering Research* 2017;21:2966-72.
38. Macrinici GI, Murphy C, Christman L, et al. Prospective, double-blind, randomized study to evaluate single-injection adductor canal nerve block versus femoral nerve block: postoperative functional outcomes after total knee arthroplasty. *Reg Anesth Pain Med* 2017;42:10-6.
39. Nader A, Kendall MC, Wixson RL, et al. A randomized trial of epidural analgesia followed by continuous femoral analgesia compared with oral opioid analgesia on short- and long-term functional recovery after total knee replacement. *Pain Med* 2012;13:937-47.
40. Peng L, Ren L, Qin P, et al. Continuous femoral nerve block versus intravenous patient controlled analgesia for knee mobility and long-term pain in patients receiving total knee replacement: a randomized controlled trial. *Evid Based Complement Alternat Med* 2014;2014:569107.
41. Reinhardt KR, Duggal S, Umunna BP, et al. Intraarticular analgesia versus epidural plus femoral nerve block after TKA: A randomized, double-blind trial. *Clin Orthop Relat Res* 2014;472:1400-8.
42. Wegener JT, van Ooij B, van Dijk CN, et al. Long-term pain and functional disability after total knee arthroplasty with and without single-injection or continuous sciatic nerve block in addition to continuous femoral nerve block: a prospective, 1-year follow-up of a randomized controlled trial. *Reg Anesth Pain Med* 2013;38:58-63.
43. Widmer BJ, Scholes CJ, Pattullo GG, et al. Is femoral nerve block necessary during total knee arthroplasty?: a randomized controlled trial. *J Arthroplasty* 2012;27:1800-5.
44. Wu JWS, Wong YC. Elective unilateral total knee replacement using continuous femoral nerve blockade versus conventional patient-controlled analgesia: perioperative patient management based on a multidisciplinary pathway. *Hong Kong Med J* 2014;20:45-51.
45. McDonald DA, Deakin AH, Ellis BM, et al. The technique of delivery of peri-operative analgesia does not affect the rehabilitation or outcomes following total knee arthroplasty. *Bone Joint J* 2016;98-B:1189-96.
46. Motififard M, Omidian A, Badiei S. Pre-emptive injection of peri-articular-multimodal drug for post-operative pain management in total knee arthroplasty: a double-blind randomized clinical trial. *Int Orthop* 2017;41:939-47.
47. Nieméläinen M, Kalliovalkama J, Aho A, et al. Single periarticular local infiltration analgesia reduces opiate consumption until 48 hours after total knee arthroplasty. A randomized placebo-controlled trial involving 56 patients. *Acta Orthop* 2014;85:614-9.
48. Seah VWT, Chin PL, Chia SL, et al. Single-dose periarticular steroid infiltration for pain management in total knee arthroplasty: a prospective, double-blind, randomised controlled trial. *Singapore Med J* 2011;52:19-23.

For peer review only - http://bmjopen.bmj.com/site/about/guidelines.xhtml

49. Williams D, Petruccelli D, Paul J, et al. Continuous infusion of bupivacaine following total knee arthroplasty: a randomized control trial pilot study. *J Arthroplasty* 2013;28:479-84.
50. Wylde V, Lenguerrand E, Gooberman-Hill R, et al. Effect of local anaesthetic infiltration on chronic postsurgical pain after total hip and knee replacement: the APEX randomised controlled trials. *Pain* 2015;156:1161-70.
51. Meunier A, Lisander B, Good L. Effects of celecoxib on blood loss, pain, and recovery of function after total knee replacement: a randomized placebo-controlled trial. *Acta Orthop* 2007;78:661-7.
52. Aveline C, Roux AL, Hetet HL, et al. Pain and recovery after total knee arthroplasty: a 12-month follow-up after a prospective randomized study evaluating Nefopam and Ketamine for early rehabilitation. *Clin J Pain* 2014;30:749-54.
53. Buvanendran A, Kroin JS, Della Valle CJ, et al. Perioperative oral pregabalin reduces chronic pain after total knee arthroplasty: a prospective, randomized, controlled trial. *Anesth Analg* 2010;110:199-207.
54. Ejaz A, Laursen AC, Kappel A, et al. Faster recovery without the use of a tourniquet in total knee arthroplasty. *Acta Orthop* 2014;85:422-6.
55. Huang Z, Xie X, Li L, et al. Intravenous and topical tranexamic acid alone are superior to tourniquet use for primary total knee arthroplasty: A prospective, randomized controlled trial. *J Bone Joint Surg (Am)* 2017;99:2053-61.
56. Liu D, Graham D, Gillies K, et al. Effects of tourniquet use on quadriceps function and pain in total knee arthroplasty. *Knee Surg Relat Res* 2014;26:207-13.
57. Mittal R, Ko V, Adie S, et al. Tourniquet application only during cement fixation in total knee arthroplasty: a double-blind, randomized controlled trial. *ANZ J Surg* 2012;82:428-33.
58. Zhang Y, Li D, Liu P, et al. Effects of different methods of using pneumatic tourniquet in patients undergoing total knee arthroplasty: a randomized control trial. *Ir Jr Med Sci* 2017;186:953-9.
59. Brock T, Sprowson A, Muller S, et al. STICKS study - Short-sTretch Inelastic Compression bandage in Knee Swelling following total knee arthroplasty - a feasibility study. *Trials* 2017;18:6.
60. Hourlier H, Reina N, Fennema P. Single dose intravenous tranexamic acid as effective as continuous infusion in primary total knee arthroplasty: a randomised clinical trial. *Arch Orthop Trauma Surg* 2015;135:465-71.
61. Kim TK, Chang CB, Kang YG, et al. Clinical value of tranexamic acid in unilateral and simultaneous bilateral TKAs under a contemporary blood-saving protocol: a randomized controlled trial. *Knee Surg Sports Traumatol Arthrosc* 2014;22:1870-8.
62. Kusuma SK, Sheridan KC, Wasielewski RC. Use of bovine thrombin to reduce blood loss in primary total knee arthroplasty: a controlled randomized trial. *J Arthroplasty* 2013;28:1278-81.
63. Napier RJ, Bennett D, McConway J, et al. The influence of immediate knee flexion on blood loss and other parameters following total knee replacement. *Bone Joint J* 2014;96-B:201-9.
64. Sa-Ngasoongsong P, Channoom T, Kawinwonggowit V, et al. Postoperative blood loss reduction in computer-assisted surgery total knee replacement by low dose intra-articular tranexamic acid injection together with 2-hour clamp drain: a prospective triple-blinded randomized controlled trial. *Orthop Rev* 2011;3:e12.
65. Sa-Ngasoongsong P, Wongsak S, Chanplakorn P, et al. Efficacy of low-dose intra-articular tranexamic acid in total knee replacement; a prospective triple-blinded randomized controlled trial. *BMC Musculoskelet Disord* 2013;14:340.
66. Ledin H, Good L, Aspenberg P. Denosumab reduces early migration in total knee replacement. *Acta Orthop* 2017;88:255-8.

For peer review only - http://bmjopen.bmj.com/site/about/guidelines.xhtml

67. Bennett LA, Brearley SC, Hart JAL, et al. A comparison of 2 continuous passive motion protocols after total knee arthroplasty: a controlled and randomized study. *J Arthroplasty* 2005;20:225-33.
68. Ersözlü S, Sahin O, Ozgür A, et al. The effects of two different continuous passive motion protocols on knee range of motion after total knee arthroplasty: a prospective analysis. *Acta Orthop Traumato* 2009;43:412-8.
69. Avramidis K, Karachalios T, Popotonasios K, et al. Does electric stimulation of the vastus medialis muscle influence rehabilitation after total knee replacement? *Orthopedics* 2011;34:175.
70. Moretti B, Notarnicola A, Moretti L, et al. I-ONE therapy in patients undergoing total knee arthroplasty: a prospective, randomized and controlled study. *BMC Musculoskelet Disord* 2012;13:88.
71. Li L, Wang Z, Yin MH, et al. Effect of early gait training on the functional rehabilitation after total knee arthroplasty. *Chinese Journal of Tissue Engineering Research* 2017;21:4288-93.
72. Liebs T, Herzberg W, Rüther W, et al. Multicenter randomized controlled trial comparing early versus late aquatic therapy after total hip or knee arthroplasty. *Arch Phys Med Rehabil* 2012;93:192-9.
73. Mahomed NN, Davis AM, Hawker G, et al. Inpatient compared with home-based rehabilitation following primary unilateral total hip or knee replacement: a randomized controlled trial. *J Bone Joint Surg (Am)* 2008;90:1673-80.
74. Wang S, Xia J, Wei Y, et al. Effect of the knee position during wound closure after total knee arthroplasty on early knee function recovery. *J Orthop Surg Res* 2014;9:79.
75. Kong CG, Kim GH, Kim DW, et al. The effect of topical scar treatment on postoperative scar pain and pruritus after total knee arthroplasty. *Arch Orthop Trauma Surg* 2014;134:555-9.
76. Hohmann E, Tetsworth K, Hohmann S, et al. Anabolic steroids after total knee arthroplasty. A double blinded prospective pilot study. *J Orthop Surg Res* 2010;5:93.
105. Bergeron SG, Kardash KJ, Huk OL, et al. Functional outcome of femoral versus obturator nerve block after total knee arthroplasty. *Clin Orthop Relat R* 2009;467:1458-62.
106. Shum CF, Lo NN, Yeo SJ, et al. Continuous femoral nerve block in total knee arthroplasty: immediate and two-year outcomes. *J Arthroplasty* 2009;24:204-9.
107. Perrin SB, Purcell AN. Intraoperative ketamine may influence persistent pain following knee arthroplasty under combined general and spinal anaesthesia: a pilot study. *Anaesth Intensive Care* 2009;37:248-53.
108. Ilfeld BM, Meyer RS, Le LT, et al. Health-related quality of life after tricompartment knee arthroplasty with and without an extended-duration continuous femoral nerve block: a prospective, 1-year follow-up of a randomized, triple-masked, placebo-controlled study. *Anesth Analg* 2009;108:1320-5.
109. Ilfeld BM, Shuster JJ, Theriaque DW, et al. Long-term pain, stiffness, and functional disability after total knee arthroplasty with and without an extended ambulatory continuous femoral nerve block: A prospective, 1-year follow-up of a multicenter, randomized, triple-masked, placebo-controlled trial. *Reg Anesth Pain Med* 2011;36:116-20.
110. Morin AM, Kratz CD, Eberhart LH, et al. Postoperative analgesia and functional recovery after total-knee replacement: comparison of a continuous posterior lumbar plexus (psoas compartment) block, a continuous femoral nerve block, and the combination of a continuous femoral and sciatic nerve block. *Reg Anesth Pain Med* 2005;30:434-45.
111. Davidson E, Machi A, Sztain J, et al. Adductor canal versus femoral continuous peripheral nerve blocks for knee arthroplasty: A one-year follow-up pilot study of two randomized, controlled clinical trials. *Reg Anesth Pain Med* 2016;41.
112. Wang F, Zhou Y, Sun J, et al. Influences of continuous femoral nerve block on knee function and quality of life in patients following total knee arthroplasty. *Int J Clin Exp Med* 2015;8:19120-5.

For peer review only - http://bmjopen.bmj.com/site/about/guidelines.xhtml

113. Anastase DM, Winckelmann J, Geiger P. Effects of regional anaesthesia techniques on patients' satisfaction after total knee arthroplasty. *Jurnalul Roman de Anestezie Terapie Intensiva* 2014;21:35-43.
114. Yue D-b, Wang B-l, Liu K-p, et al. Efficacy of multimodal cocktail periarticular injection with or without steroid in total knee arthroplasty. *Chinese Med J* 2013;126:3851-5.
115. Spreng UJ, Andersson E, Dahl V. Long-term outcome after total knee arthroplasty local infiltration analgesia (LIA) vs. Epidural analgesia. *Br J Anaesth* 2012;108:ii419.
116. Spreng UJ, Dahl V, Hjall A, et al. High-volume local infiltration analgesia combined with intravenous or local ketorolac+morphine compared with epidural analgesia after total knee arthroplasty. *Br J Anaesth* 2010;105:675-82.
117. Foadi N, Karst M, Frese-Gaul A, et al. The improved quality of postoperative analgesia after intrathecal morphine does not result in improved recovery and quality of life in the first 6 months after orthopedic surgery: a randomized controlled pilot study. *J Pain Res* 2017;10:1059-69.
118. Mayoral O, Salvat I, Martin MT, et al. Efficacy of myofascial trigger point dry needling in the prevention of pain after total knee arthroplasty: A randomized, double-blinded, placebo-controlled trial. *Evid Based Complement Alternat Med* 2013;2013:Article ID 694941.
119. Abdel-Salam A, Eyres KS. Effects of tourniquet during total knee arthroplasty. A prospective randomised study. *J Bone Joint Surg (Br)* 1995;77:250-3.
120. Sükür E, Öztürkmen Y, Akman Y, et al. The effect of tourniquet and knee position during wound closure after total knee arthroplasty on early recovery of range of motion: a prospective, randomized study. *Arch Orthop Trauma Surg* 2016;136:1773-80.
121. Zhang Q, Dong J, Gong K, et al. Effects of tourniquet use on perioperative outcome in total knee arthroplasty. *Chinese Journal of Reparative and Reconstructive Surgery* 2016;30:421-5.
122. Brock TM, Sprowson AP, Muller S, et al. STICKS study - Short-sTretch Inelastic Compression bandage in Knee Swelling following total knee arthroplasty - a feasibility study. *Trials* 2017;18:6.
123. Thomas D, Wareham K, Cohen D, et al. Autologous blood transfusion in total knee replacement surgery. *Br J Anaesth* 2001;86:669-73.
124. Aggarwal AK, Shashikanth VS, Marwaha N. Platelet-rich plasma prevents blood loss and pain and enhances early functional outcome after total knee arthroplasty: a prospective randomised controlled study. *Int Orthop* 2014;38:387-95.
125. Wang X. Impact of local compression cryotherapy combined with continuous passive motion on the early functional recovery after total knee arthroplasty. *Chinese Journal of Tissue Engineering Research* 2017;21:998-1003.
126. Leach W, Reid J, Murphy F. Continuous passive motion following total knee replacement: a prospective randomized trial with follow-up to 1 year. *Knee Surg Sports Traumatol Arthrosc* 2006;14:922-6.
127. Sahin E, Akalin E, Bircan C, et al. The effects of continuous passive motion on outcome in total knee arthroplasty. *Journal of Rheumatology and Medical Rehabilitation* 2006;17:85-90.
128. Pope RO, Corcoran S, McCaul K, et al. Continuous passive motion after primary total knee arthroplasty. Does it offer any benefits? *J Bone Joint Surg (Br)* 1997;79:914-7.
129. Beaupre LA, Davies DM, Jones CA, et al. Exercise combined with continuous passive motion or slider board therapy compared with exercise only: a randomized controlled trial of patients following total knee arthroplasty. *Physical therapy* 2001;81:1029-37.
130. Kumar PJ, McPherson EJ, Dorr LD, et al. Rehabilitation after total knee arthroplasty: a comparison of 2 rehabilitation techniques. *Clin Orthop Relat R* 1996:93-101.
131. Worland RL, Arredondo J, Angles F, et al. Home continuous passive motion machine versus professional physical therapy following total knee replacement. *J Arthroplasty* 1998;13:784-7.

For peer review only - http://bmjopen.bmj.com/site/about/guidelines.xhtml

132. MacDonald SJ, Bourne RB, Rorabeck CH, et al. Prospective randomized clinical trial of continuous passive motion after total knee arthroplasty. *Clin Orthop Relat Res* 2000:30-5.
133. Stevens-Lapsley JE, Balter JE, Wolfe P, et al. Early neuromuscular electrical stimulation to improve quadriceps muscle strength after total knee arthroplasty: a randomized controlled trial. *Physical therapy* 2012;92:210-26.
134. Levine M, McElroy K, Stakich V, et al. Comparing conventional physical therapy rehabilitation with neuromuscular electrical stimulation after TKA. *Orthopedics* 2013;36:e319-24.
135. Adravanti P, Nicoletti S, Setti S, et al. Effect of pulsed electromagnetic field therapy in patients undergoing total knee arthroplasty: a randomised controlled trial. *Int Orthop* 2014;38:397-403.
136. Rahmann AE, Brauer SG, Nitz JC. A specific inpatient aquatic physiotherapy program improves strength after total hip or knee replacement surgery: a randomized controlled trial. *Arch Phys Med Rehabil* 2009;90:745-55.
137. Mahomed NN, Davis AM, Hawker G, et al. Inpatient compared with home-based rehabilitation following primary unilateral total hip or knee replacement: a randomized controlled trial. *J Bone Joint Surg (Am)* 2008;90:1673-80.
138. Hill SP, Flynn J, Crawford EJP. Early discharge following total knee replacement -- a trial of patient satisfaction and outcomes using an orthopaedic outreach team. *J Orthop Nurs* 2000;4:121-6.
139. Jacobson AF, Umberger WA, Palmieri PA, et al. Guided imagery for total knee replacement: A randomized, placebo-controlled pilot study. *J Altern Complement Med* 2016;22:563-75.

For peer review only

For peer review only - http://bmjopen.bmj.com/site/about/guidelines.xhtml
